# Genome-wide analysis of RING-type E3 ligase family identifies potential candidates regulating high amylose starch biosynthesis in wheat (*Triticum aestivum* L.)

**DOI:** 10.1038/s41598-021-90685-7

**Published:** 2021-06-01

**Authors:** Afsana Parveen, Mohammed Saba Rahim, Ankita Sharma, Ankita Mishra, Prashant Kumar, Vikas Fandade, Pankaj Kumar, Abhishek Bhandawat, Shailender Kumar Verma, Joy Roy

**Affiliations:** 1 Agri-Food Biotechnology Division, National Agri-Food Biotechnology Institute, Mohali, 140306 Punjab India; 2 Department of Biotechnology, Panjab University, Chandigarh, 160014 Punjab India; 3 Centre for Computational Biology and Bioinformatics, School of Life Sciences, Central University of Himachal Pradesh, Kangra, 176206 Himachal Pradesh India

**Keywords:** Biotechnology, Plant sciences

## Abstract

In ubiquitin-mediated post-translational modifications, RING finger families are emerged as important E3 ligases in regulating biological processes. Amylose and amylopectin are two major constituents of starch in wheat seed endosperm. Studies have been found the beneficial effects of high amylose or resistant starch on health. The ubiquitin-mediated post-translational regulation of key enzymes for amylose/amylopectin biosynthesis (GBSSI and SBEII) is still unknown. In this study, the genome-wide analysis identified 1272 RING domains in 1255 proteins in wheat, which is not reported earlier. The identified RING domains classified into four groups—RING-H2, RING-HC, RING-v, RING-G, based on the amino acid residues (Cys, His) at metal ligand positions and the number of residues between them with the predominance of RING-H2 type. A total of 1238 RING protein genes were found to be distributed across all 21 wheat chromosomes. Among them, 1080 RING protein genes were identified to show whole genome/segmental duplication within the hexaploid wheat genome. In silico expression analysis using transcriptome data revealed 698 RING protein genes, having a possible role in seed development. Based on differential gene expression and correlation analysis of 36 RING protein genes in diverse (high and low) amylose mutants and parent, 10 potential RING protein genes found to be involved in high amylose biosynthesis and significantly associated with two starch biosynthesis genes; GBSSI and SBEIIa. Characterization of mutant lines using next-generation sequencing method identified unique mutations in 698 RING protein genes. This study signifies the putative role of RING-type E3 ligases in amylose biosynthesis and this information will be helpful for further functional validation and its role in other biological processes in wheat.

## Introduction

Bread wheat (*Triticum aestivum*) is a rich source of starch (~ 70%)^1^, that comprising of amylose (~ 25%) and amylopectin (~ 75%)^2^. High amylose starch has nutritional benefits as it is classified under type-2 resistant starch (RS) category. The resistant starch is slowly digestible in human gut, therefore it has low glycemic index. It is beneficial for combating gastrointestinal diseases, diabetes and obesity^3–6^. In wheat, the natural variation for high amylose starch is narrow. It can be improved by genetic engineering tools using the key pathway genes. It is found that the progress is limited as overexpression and knock-down/out of two key genes—GBSSI and SBEII has limited success^7–9^. This can also be done by mutational breeding approaches^10^. Many post translational studies disclosed the different aspects of starch biosynthesis pathway^11–13^ to date but the ubiquitin-mediated post translational insights of the starch biosynthesis pathway has not yet revealed and no attempt has been done for high amylose starch.

Ubiquitin-mediated post-translational modifications targeting protein degradation has emerged as a crucial process in controlling different aspects of cellular processes in eukaryotes^14,15^. This process involves the subsequent action of three enzymes, E1 (ubiquitin activating enzyme), E2 (ubiquitin conjugating enzyme), and E3 (ubiquitin ligase)^16^. In this multistep process E3 ubiquitin ligases are key determinant of target specificity for degradation by 26S proteasome system^17,18^, which are mainly classified in two groups HECT (Homologous to the E6-associated protein C terminus) and RING (Really Interesting New Gene) finger/U-box domain^17,19^. Of these known E3 ligases, RING finger domain containing proteins comprise major proportion. The RING domains are considered to be involved in protein–protein interactions and essential for E2 dependent ubiquitination, that can function as a single subunit or in multi-subunit complexes^20–22^. RING (Really Interesting New Gene/U-box)-type E3 ligases belong to the largest class of E3 ligases with > 600 members in human as the RING E3 ligases function is diverse and act as allosteric activators of the E2. Initially the RING domains were characterized as RING-H2 and RING-HC type on the basis His and Cys at fifth metal ligand position, respectively. Further 5 modified RING types RING-C2, RING S/T, RING-v, RING-D and RING-G having variation in amino acid residue at metal ligand positions were also identified in *A. thaliana*^23,24^.

The role of RING finger E3 ligases in the plant development have been extensively studied for various biological processes. RING finger containing proteins like COP1 is a well-known for its role as photomorphogenic repressor^25^, XBAT35.2 in cell death induction and pathogen response^26^, HOS1 in cold response^27^, KEG in growth and development^28^, *Capsicum annuum* CaAIRF1 in ABA and drought signaling^29^, ATL2/ATL9 in defense response^30^. Recent studies revealed that SP1, a RING type E3 ligase is involved in degradation of TOC components (translocon) present in chloroplast outer membrane and hence regulate the chloroplast protein import^31^. However, the role of this degradation pathway in amyloplast proteins turnover remains unclear. Beside the role in different biological processes the RING finger E3 ligase GW2 found to be involved in grain size and weight in rice and wheat possibly by regulating the expression of starch biosynthetic pathway genes^32–34^. The reduction in the transcripts of GW2 by RNAi in the durum wheat cultivar increased the starch content by 10–40%, width by 4–13% and surface area by 3–5%, that suggest the active role of GW2 RING finger E3 ligase in starch biosynthesis but its interacting partners need to be explored^35^. These previous studies provides substantial information to perform the current study.

In this study, we reported the genome-wide identification and characterization of RING domain containing E3 ligase family in wheat as well as identified the putative RING E3 ligases that may involve in high amylose biosynthesis based on quantitative gene expression and correlation analysis in developing wheat grains. A large set of 1255 proteins containing 1272 RING domains was first time identified in wheat and 10 potential RING protein genes found to be involved in high amylose biosynthesis and significantly associated with two starch biosynthesis genes; *GBSSI* and *SBEIIa*. Further, the transcriptome sequencing using next-generation sequencing method identified several unique induced mutations in 698 RING protein genes. Hence, this studies lay the foundation for future research to make better understanding of RING finger E3 ligases involvement in high amylose starch biosynthesis in cereal crops.

## Results

### Identification and classification of RING finger proteins in wheat

**Figure 1:**
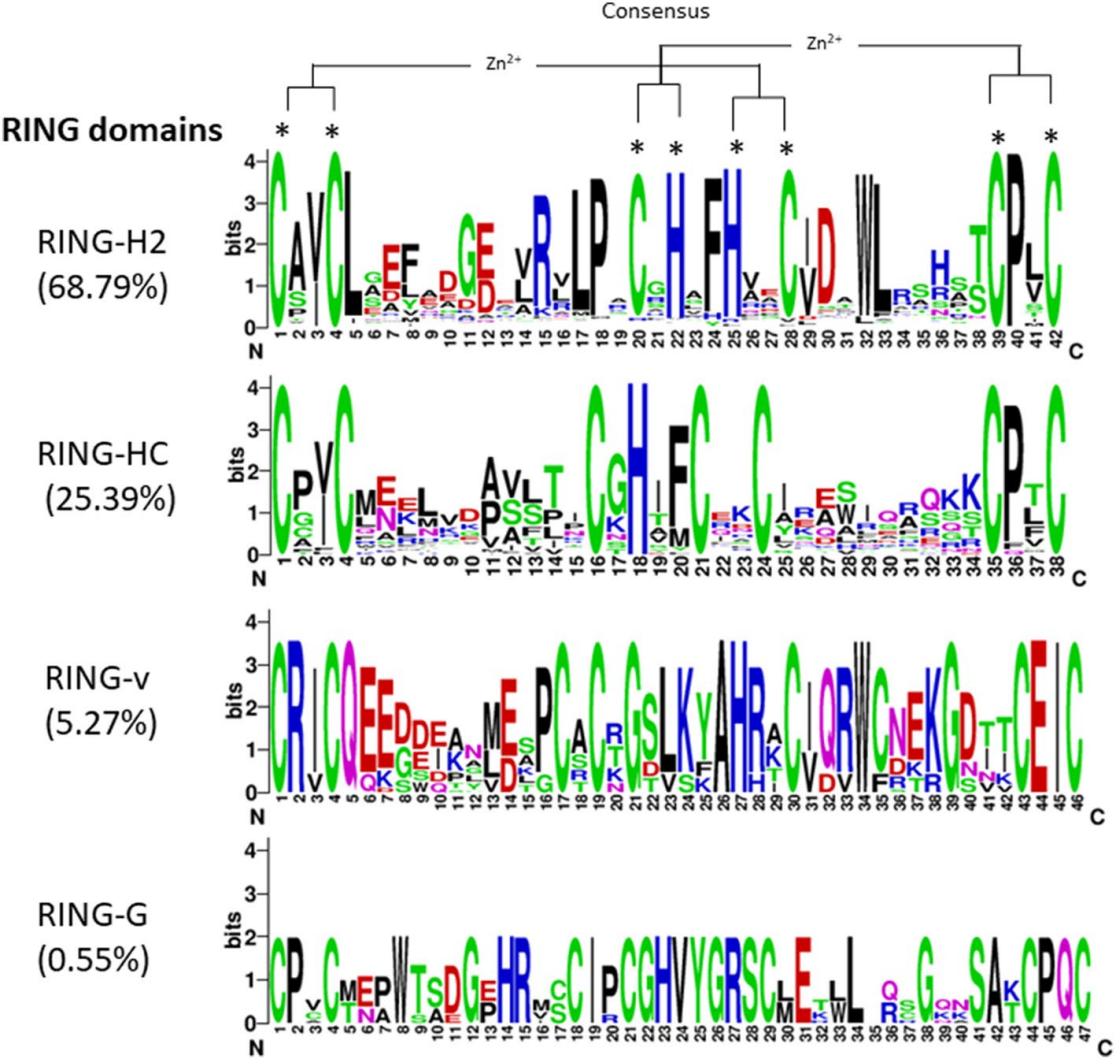
Sequence logo of overrepresented motifs found in RING-H2, RING-HC, RING-v, RING-G domains, respectively generated using WebLogo program version 2.8.2 (https://weblogo.berkeley.edu/logo.cgi)**.** The figures were generated by on-line WebLogo. The height of the letters indicates the conservation at that particular position. The asterisked letters indicate the conserved metal ligands and zinc coordinating amino acid pairs.

A total of 1272 potential RING domains in 1255 proteins were identified in wheat proteome through in silico studies (Supplementary Table S1). Among the identified proteins, 1241 proteins contained a single RING domain, 12 proteins with two RING domains and one protein each with three and four domains. Proteins with multiple RING domains were suffixed with an alphabet to their gene IDs. Predicted RING domain containing proteins size ranged from 84 to 4749 amino acids with domain size ranging from 30 to 102 amino acids. On the basis of amino acid residues at eight metal ligand positions (Cys and/or His) and number of residues between them, 1272 RING domains were classified into 4 groups according to Stone et al*.*, (2005). Maximum number of RING domains i.e. 875 domains (68.79%) were identified as RING-H2 followed by 323 RING-HC domain (25.39%), 67 RING-v domains (5.27%) and 7 RING-G domains (0.55%) (Supplementary Table S1, Supplementary Fig. S1). Representative sequence logos of protein motifs of the four identified groups are shown in Fig. 1. In RING-H2 type (out of 875) six domains were identified as RBX type having Asp instead of Cys at eighth metal ligand position (Supplementary Fig. S1). In wheat we did not identify RING-D, RING-C2 and RING-S/T type domains which were present in *Arabidopsis*, *Brassica rapa*, apple and rice. In this study, a large number of RING containing proteins were identified as compared to other plant species such as *Arabidopsis* (469), rice (488), apple (663) *B. rapa* (715) and *B. oleracea* (734) ^24,36–39^. Additionally, 117 RING domains in 94 proteins were also identified by in silico studies but due to the absence or substitution of one or more amino acid residues at metal ligand positions they were not classified in any group and considered as incomplete RING domains (Supplementary Table S2). But for the downstream analysis only complete RING protein genes were considered.

### Phylogenetic analysis

To expand the knowledge on wheat RING proteins, this study included phylogenetic analysis among 1272 RING domain sequences of 1255 wheat RING proteins. Majority of similar type of RING domains (RING-H2, RING-HC, RING-v and RING-G) were clustered together with various small subgroups (Supplementary Fig. S2). The phylogenetic relationship suggested that most of the domains from the same group were categorized together while some were found intermixed with other domains. This topology might be due to the discrepancy in domain sequences except the conserved metal ligands, during evolutionary process^40^. RING-H2 and RING-HC domains tend to be distributed within each other forming several subgroups containing small number of domains. The RING-G and RING-v domains were clustered together but lied within major RING-H2 and RING-HC domain groups.

### Additional domains in RING finger proteins

Out of the 1255 RING finger proteins, 885 proteins (70.52%) contained one or more additional domains other than RING (Supplementary Table S3). On the basis of presence/absence and organization of the additional domains, the 1255 RING domain containing proteins were grouped into 36 major groups (Table 1; Supplementary Table S3). Of 885 RING domain containing proteins, 544 (group 6.1) proteins contained additional transmembrane domain (1–14 repetitions) and 43 (group 3) proteins had only coiled coil (repetition 1–6) domain. The Zinc-Ribbon-9, signal peptide and VWA domains were present in 26, 19 and 18 RING domain containing proteins of group 13.1, 21.1 and 11.1 respectively. Some of the RING finger proteins were found to have two or more other domains along with the aforementioned domains. As with the transmembrane domain other domains, namely GIDE, IBR, PA, VWA, zinc_ribbon_9, Tmemb_185A, coiled-coil and signal peptide were also present in RING domain containing proteins. A protein–protein interacting domain VWA was simultaneously found in 20 RING proteins in combination with Vwaint domain. The Vwaint domain previously not found in *Arabidopsis* RING finger proteins^24^ but further identified in brassica^39^. The two domains Copine and NB-ARC were specifically found in wheat RING proteins, previously not found in any other plant species. Four domains ZnF_UBP, GIDE, RWD and CUE involved in ubiquitination were also identified. Nucleic acid binding domains DEXDc, HELICc, HIRAN, ZNF_C2H2, ZNF_C3H1 and ZNF_C2HC were identified in 27 RING finger proteins. Protein–protein interaction domains, Ankyrin repeats, BRCT, TPR, VWA, Coiled-coil and WD40 repeats were identified in RING finger proteins that help in substrate recognition by E3 ligases^41–43^ The two protein–protein interaction domains PHD and ZnF_ZZ from the same family of cross-brace zinc finger, from which RING domain belongs, were also identified. Presence of these various types of additional domains indicates that instead of RING finger domains these proteins share different features and are very distinct to each other.

**Table 1:** Wheat RING domain containing proteins grouped based on the presence or absence and organization of the additional domain/s in RING proteins. The possible wheat RING protein orthologs in other species are shown.

Sr. no.	Group no.	No. of proteins	Representative gene IDs	Additional domain/s	Species*	Domain description
1	1	370	TraesCS1A02G090900	RING	At, Ce, Dd, Dm, Dr, Gm, Hs, Ld, Mm, Sc	RING domain, protein binding, Zinc ion binding, E3 ubiquitin ligase activity^20^
2	2	3	TraesCS1A02G088100	ANK	At, Ce, Dr, Gm, Hs, Mm	Ankyrin repeats; protein–protein interactions^61^
3	3	43	TraesCS1A02G084200	Coiled-coil	At, Dd, Dr, Gm, Hs, Mm, Pf	Coiled-coil domain
4	4.1	2	TraesCS6A02G145300	BRAP2, ZnF_UBP	At, Ce, Dd, Dm, Dr, Gm, Hs, Ld, Mm, Pf, Sc, Sp	BRCA1-associated protein 2^62^, Ubiquitin C-terminal hydrolase-like zinc finger; ubiquitinbinding domain
5	4.2	1	TraesCS6D02G134300	Coiled-coil, BRAP2, ZnF_UBP	At, Ce, Dd, Dm, Dr, Gm, Hs, Ld, Mm, Pf, Sc, Sp	See group 3, See group 4.1
6	5	1	TraesCS5B02G111900	Copine	At, Ce, Dd, Dm, Dr, Gm, Hs, Mm	Ca(2+)-dependent phospholipid-binding proteins^63^
7	6.1	544	TraesCS1A02G126200	Transmembrane	At, Ce, Ce, Dd, Dm, Dr, Gm, Hs, Mm, Sc	Transmembrane
8	6.2	6	TraesCS7B02G442100	Transmembrane, coiled-coil	At, Dd, Dm, Dr, Gm, Hs, Mm	See group 6.1, See group 3
9	7.1	8	TraesCS2A02G546000	IBR	At, Ce, Dd, Dm, Dr, Gm, Hs, Ld, Mm, Sp	IBR, In between ring fingers, cysteine-rich (C6HC) zinc finger domain
10	7.2	1	TraesCS2D02G187100	IBR, Signal peptide	At, Ce, Dd, Dm, Dr, Gm, Hs, Mm, Sp	See group 7.1, Signal peptide
11	8	3	TraesCS1A02G177600	Coiled-coil, KISc	At, Ce, Dd, Dm, Dr, Gm, Hs, Mm	See group 3, Kinesin motor domain; ATPase activity, role in cell division and organelle transport
12	9.1	1	TraesCS1B02G074500	HELICc	At, Cd, Ce, Dd, Dm, Dr, Gm, Hs, Ld, Mm, Pf, Sc, Sco, So, Sp, Sp	Helicase superfamily c-terminal domain; helicase activity; ATP binding; nucleotide-binding^64^
13	9.2	4	TraesCS2A02G224300	DEXDc, HELICc	At, Ce, Dd, Dm, Dr, Gm, Hs, Ld, Mm	DEAD-like helicases; ATP binding See group 9.1
14	9.3	9	TraesCS2A02G145200	DEXDc, HELICc, HIRAN	At, Cd, Ce, Dd, Dm, Dr, Gm, Hs, Ld, Mm, Pf, Sc, Sp, Spn	See group 9.2, See group 9.1, found in N-terminal regions of SWI2/SNF2 ptoteins^65^
15	10	1	TraesCS1D02G002400	PHD, SRA	At, Gm, Dr, Mm, Hs, Dm, Pf, Ld, Dr, Sco	Plant homeodomain C4HC3 type^66^, SET and RING finger associated domain
16	11.1	18	TraesCS3A02G414400	VWA	At, Ce, Dd, Dr, Gm, Hs, Ld, Mm	Von Willebrand factor (vWF) type A domain
17	11.2	1	TraesCS5D02G122000	Signal peptide, VWA	At, Ce, Dd, Dr, Gm, Hs, Ld, Mm	See group 7.2, See group 11.1
18	12	1	TraesCS3D02G079000	Coiled-coil, TPR_7	At, Ce, Dd, Dm, Dr, Gm, Hs, Ld, Mm, Pf	See group 3, TPR_7
19	13.1	26	TraesCS1A02G162400	Zinc_ribbon_9	At, Ce, Dd, Dm, Dr, Gm, Hs, Mm, Pf, Sp	Zinc-binding ribbon domain
20	13.2	3	TraesCS5A02G467700	Zinc_ribbon_9, coiled-coil	At, Dd, Dm, Dr, Gm, Hs, Mm, Pf, Sp	See group 13.1, See group 3
21	13.3	2	TraesCS7A02G169600	Zinc_ribbon_9, transmembrane	At, Ce, Dd, Dm, Dr, Gm, Hs, Mm, Pf	See group 13.1, See group 6.1
22	14	1	TraesCS7A02G005100	NB-ARC	At, Gm	Signalling motif found in bacteria and eukaryotes^67^
23	15	3	TraesCS1A02G341400	DUF4792, DUF4793, signal peptide	At, Dd, Dm, Dr, Gm, Hs, Ld, Mm	Domain of unknown function approximately 70 residues, Domain of unknown function approximately 110 residues, See group 7.2
24	16	8	TraesCS1A02G029800	DUF1117, Zinc_ribbon_9	At, Dd, Dm, Dr, Gm, Hs, Mm, Pf	Domain of unknown function, See group 13.1
25	17.1	1	TraesCS2B02G577000	IBR, transmembrane	At, Ce, Dd, Dm, Dr, Gm, Hs, Ld, Mm, Sp	See group 7.1, See group 6.1
26	17.2	1	TraesCS2D02G547300	IBR, signal peptide, transmembrane	At, Ce, Dd, Dm, Dr, Gm, Hs, Ld, Mm, Sp	See group 7.1, See group 7.2, See group 6.1
27	18	3	TraesCS4A02G162000	LON_substr_bdg	At, Ce, Dm, Dr, Gm, Hs, Mm, Pa, Pf, Sh, Sp	ATP-dependent protease La (LON) domain; ATP- dependent Ser peptidases^68^
28	19	9	TraesCS4A02G117900	PHD	At, Ce, Dd, Dm, Dr, Gm, Hs, Mm, Pf, Sp	See group 10
29	20.1	1	TraesCS7B02G226400	PA, TRANSMEMBRANE	At, Ce, Dm, Dr, Gm, Hs, Mm	See group 6.1
30	20.2	5	TraesCS2B02G625300	PA, signal peptide, transmembrane	At, Ce, Dm, Dr, Gm, Hs, Mm, Sp	See group 7.2, See group 6.1
31	21.1	19	TraesCS1B02G156500	Signal peptide	At, Ce, Dm, Dr, Gm, Hs, Mm	See group 7.2
32	21.2	43	TraesCS2B02G336300	Signal peptide, TRANSMEMBRANE	At, Ce, Dd, Dm, Dr, Gm, Hs, Mm, Sp, Sp	See group 7.2, See group 6.1
33	21.3	2	TraesCS3A02G051000	Signal peptide, transmembrane, coiled-coil	At, Gm	See group 7.2, See group 6.1, See group 3
34	22	6	TraesCS1A02G025700	PHD, BRCT	At, Ce, Dd, Dm, Dr, Gm, HS, Ld, Mm, Sp	See group 10
35	23	3	TraesCS2A02G094000	PHD, coiled-coil, AT_hook	At, Dd, Dr, Gm, Hs, Mm	See group 10, See group 3
36	24.1	2	TraesCS1A02G143300	Zinc_ribbon_6	At, Ce, Dd, Dm, Dr, Gm, Hs, Mm, Pf, Sc, Sp	Zinc-ribbon finger, each pair of zinc-ligands coming from more-or-less either side of two knuckles^69^
37	24.2	8	TraesCS1D02G142100	Zinc_ribbon_6, zf-CHY	At, Ce, Dd, Dm, Dr, Gm, Hs, Pf, Sc, Sp	See group 24.1, Zinc ion binding; function unknown^70^
38	24.3	3	TraesCS1A02G374800	Zinc_ribbon_6, zf-CHY, Hemerythrin	At, Ce, Dd, Dm, Dr, Gm, Hs, Mm, Sp	See group 24.1, See group 24.2, responsible for oxygen (O2) transport in the marine invertebrate
39	25	1	TraesCS3A02G288900	ZnF_C2H2	At, Sp, Dd, Dm, Dr, Gm, Hs, Mm	C2H2-type zinc finger, binds to DNA, RNA and protein targets
40	26	9	TraesCS4A02G082400	ZnF_C3H1	At, Hs, Ce, Dd, Dm, Dr, Gm, Ld, Mm, Pf, Sc, Sp	Zinc-finger domain C- × 8-C- × 5-C- × 3-H type; nucleic acid binding^71^
41	27	3	TraesCS6B02G255500	ZnF_ZZ	At, Dd, Dm, Dr, Gm, Hs, Mm, Pf, Sp	Zinc-finger domain C- × 2-C- × 5-C- × 2-C type^72^
42	28	3	TraesCS2A02G446100	IBR, RWD, ZnF_C2HC	At, Ce, Dd, Dm, Dr, Gm, Hs, Mm, Sc, Sp	See group 7.1, function in protein interaction, See group 6.1
43	29.1	1	TraesCS1B02G007900	SRA, coiled-coil	At, Dr, Dra, Gm, Hs, Mm, Sc, Sc	See group 10, See group 3
44	29.2	1	TraesCS1A02G005700	Coiled-coil, PHD, SRA	At, Gm, Mm, Hs, Dr, Dra, Sco	See group 3, See group 10, See group 10
45	30	6	TraesCS5D02G213300	Tmemb_185A	At, Ce, Dd, Dm, Dr, Gm, Hs, Ld, Mm	Transmembrane Fragile-X-F protein, linked to FragileXF syndrome
46	31.1	20	TraesCS1A02G146700	Vwaint, VWA	At, Ce, Dd, Dm, Dr, Gm, Hs, Ma, Mm, Pf, Sc, Sh, Sp	Lies between the N-terminal VWA domain and the more C-terminal 'Vint'-type Hint domain^73^, See group 11.1
47	31.2	3	TraesCS2A02G364100	Vwaint, Signal peptide, VWA	At, Ce, Dm, Dr, Gm, Hs, Ld, Ma, Mm, Pf, Sc, So, Sp	See group 31.1, See group 7.2, See group 11.1
48	31.3	2	TraesCS4B02G344200	Coiled-coil, Vwaint, VWA	At, Dr, Gm, Hs, Ma, Mm, Sco, So, Ssp.	See group 3, See group 31.1, See group 11.1
49	32.1	4	TraesCS3B02G567600	Transmembrane, DUF1232	At, Ce, Dd, Dm, Dr, Gm, Hs, Ld, Mm, Pf, Sc	See group 6.1, Domain of unknown function representing a conserved region of approximately 60 residues
50	32.2	23	TraesCS1A02G206100	Transmembrane, DUF3675	At, Ce, Dd, Dr, Gm, Hs, Mm	See group 6.1, Domain of unknown function approximately 120 amino acids in length
51	33	2	TraesCS2A02G227400	Transmembrane, CUE	At, Ce, Dd, Dm, Dr, Gm, Hs, Ld, MM, Pf, Sc, Sp	See group 6.1, Involved in binding ubiquitin-conjugating enzymes; ubiquitin-binding motif^74^
52	34.1	3	TraesCS1A02G263800	WD40	At, Ce, Dd, Dm, Dr, Gm, Hs, Ma, Mm, Sc, Sp, Ssp.	Found as tandem repeats each containing a central Trp/Asp motif; Phospho-Ser/Thr-binding domain^75^
53	34.2	3	TraesCS6A02G326100	WD40, coiled-coil	At, Ce, Dd, Dm, Dr, Gm, Hs, Ld, Ma, Mm, Sc, Sp	See group 34.1, See group 3
54	35	2	TraesCS2D02G141100	GIDE, signal peptide	At, Dd, Dm, Dr, Gm, Hs, Ld, Mm	E3 ubiquitin ligase involved in inducing apoptosis^76^, See group 7.2
55	36	3	TraesCS4A02G011000	GIDE, transmembrane	At, Ce, Dd, Dm, Dr, Gm, HS, Ld, Mm, Pf	See group 35, See group 6.1

*Species having similar domain architecture in RING proteins determined by NCBI BLASTp search in model organisms (landmark) database only.

*At:* *Arabidopsis thaliana*, *Cd:* Clostridioides difficile, *Ce:* *C. elegans,* *Dd:* *Dictyostelium discoideum*, *Dm:* *Drosophila melanogaster*, *Dr:* *Danio rerio*, *Dra:* *Deinococcus radiodurans*, *Gm:* *Glycine max*, *Hs:* *Homo sapiens*, *Ld:* *Leishmania donovani*, *Ma:* *Microcystis aeruginosa*, *Mm:* *M. musculus*, *Pa:* *Pseudomonas aeruginosa*, *Pf:* *Plasmodium falciparum*, *Sc:* *Saccharomyces cerevisiae*, *Sco:* S*treptomyces coelicolor*, *So:* Shewanella oneidensis, *Sp:* Schizosaccharomyces pombe, *Spn:* *Streptococcus pneumonia*, *Ssp.:* *Synechocystis* sp., *Xl:* Xenopuslaevis, *Zm:* Zea mays.

### Chromosomal locations and duplication events analysis

**Figure 2:**
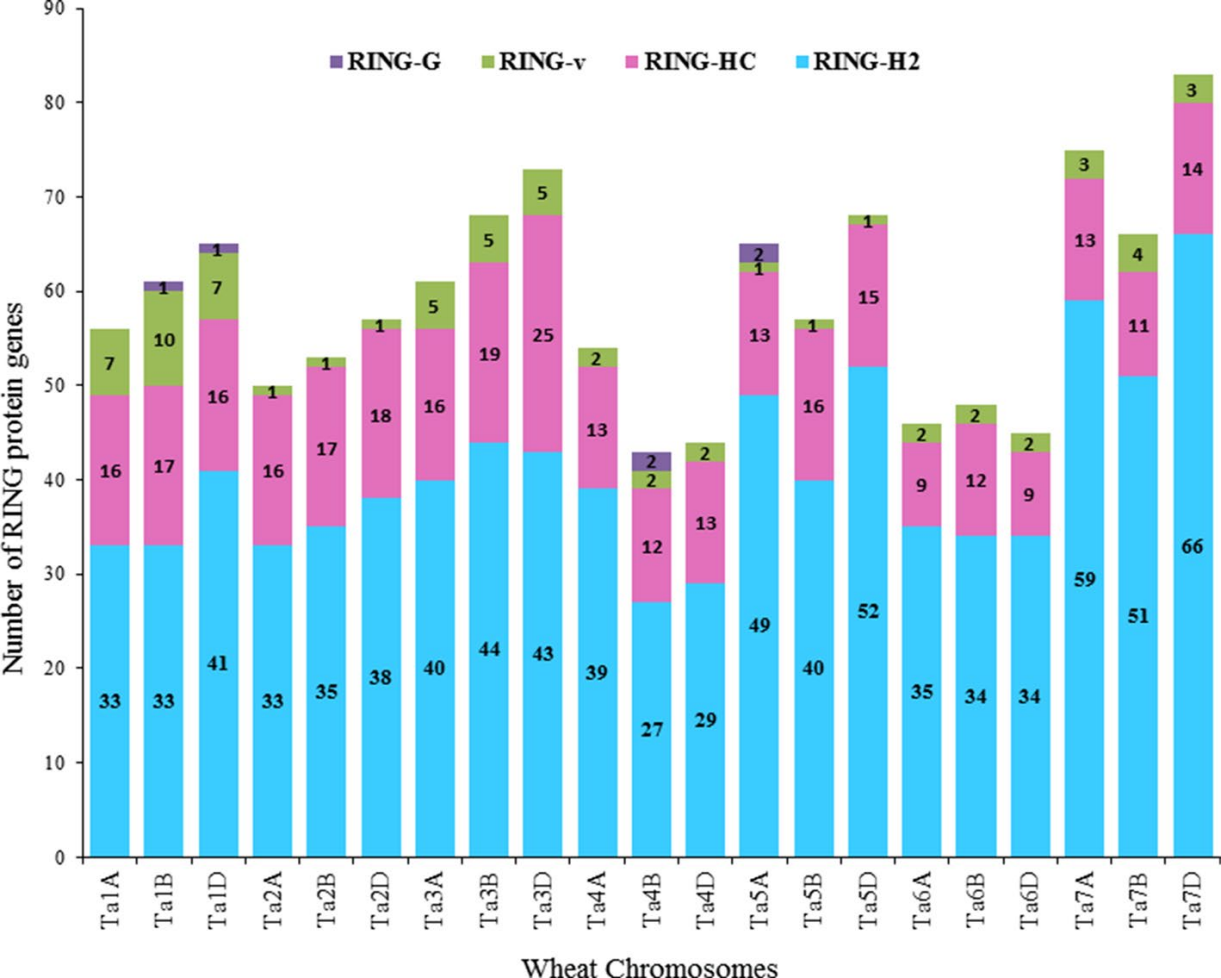
Histogram representing the distribution of RING protein genes in the identified four RING domain groups on 21 chromosomes (Ta1A to Ta7D) based on chromosomal locations from Ensembl *Triticum aestivum* database. Where, Ta stand for *Triticum aestivum* followed by chromosome name.

**Figure 3:**
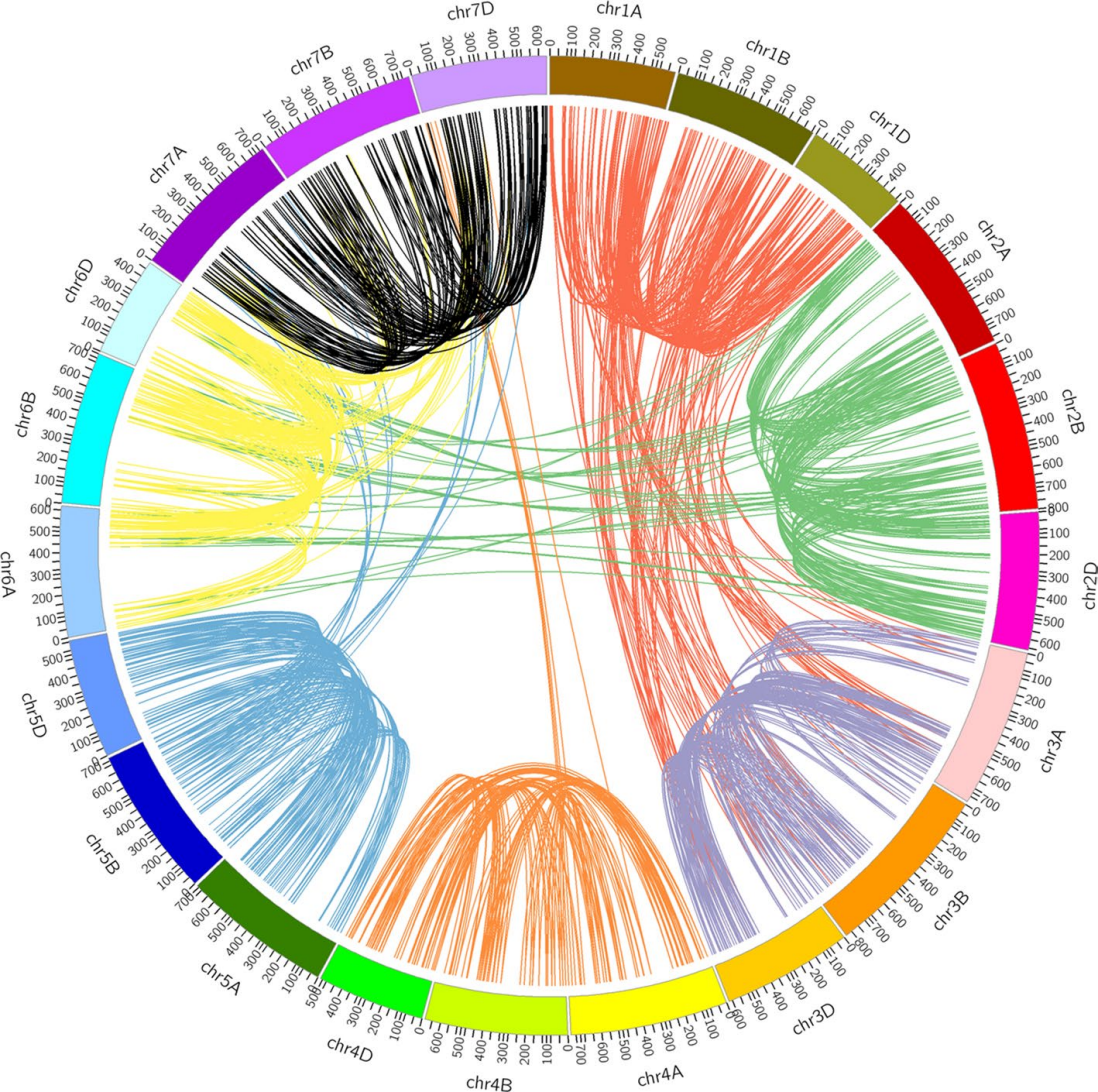
Chromosomal locations and whole genome/segmental duplication events of 1238 RING protein genes on 21 chromosomes of hexaploid wheat. The suffix after ‘chr’ represent wheat chromosome name (chr1A to chr7D) and is given a different color.

The chromosomal location of 1255 RING protein genes were extracted from *T. aestivum* genome database downloaded from Ensembl plants (ftp://ftp.Ensemblgenomes.org/pub/plants/release-46/gff3/triticum_aestivum). Out of 1255, 17 genes were mapped to unassembled chromosomal locations that were excluded in this study. The retrieved chromosomal locations of rest 1238 wheat RING protein genes were  mapped on wheat genome that were randomly distributed on all the 21 chromosomes (1A, 1B, 1D to 7A, 7B, 7D) (Supplementary Table S4). RING protein genes were found to be less distributed on 4B (3.5%) and largely distributed on 7D (6.7%) chromosome (Supplementary Fig. S3). The total number of genes in each RING class (RING-H2, RING-HC, RING-v, RING-G) were also determined and it was observed that RING-H2, RING-HC, and RING-v type RING protein genes were distributed throughout all the 21 chromosomes but genes from RING-G class were only present on chromosomes 1B, 1D, 4B and 5A (Fig. 2). Average distance between two RING protein genes ranged from 7.6 to 15.6 Mb on chromosomes 1D and 4B, respectively. The results decipher that RING protein genes are inconsistently distributed on wheat genome. Meanwhile, singleton (not duplicated) and four duplication events dispersed, proximal, tandem, WGD/segmental in 4, 49, 15, 90, and 1080 RING protein genes were identified, respectively (Supplementary Table S4). Whole genome duplicated/segmental, tandem and dispersed (except 2A and 6D) duplicated genes were distributed all over the genome. Singleton were found only on 1B, 3D, 4B, and 6D, while proximal were found on 1A, 1B, 1D, 2A, 3A, 5A, 5D, 7A and 7D. Whole genome/segmental duplicated genes from each chromosome are displayed in Fig. 3.

### Expression profiling during grain development

**Figure 4:**
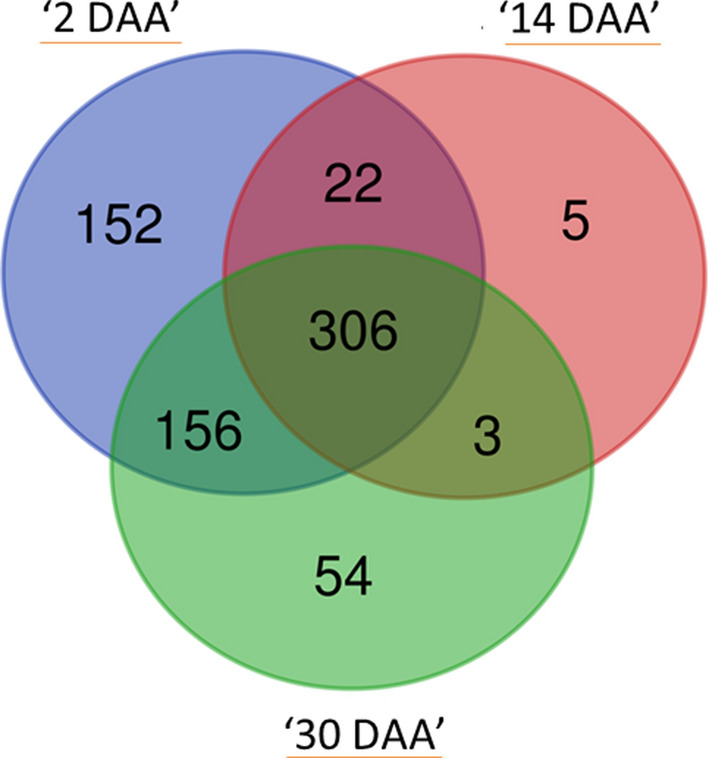
Venn diagram showing number of commonly and uniquely expressed RING finger containing genes at three seed developmental stages (2, 14 and 30 DAA). The data were taken from the publicly available RNA-seq databases of wheat (https://wheat.pw.usda.gov/GG3/node/237). DAA stands for day after anthesis.

**Figure 5:**
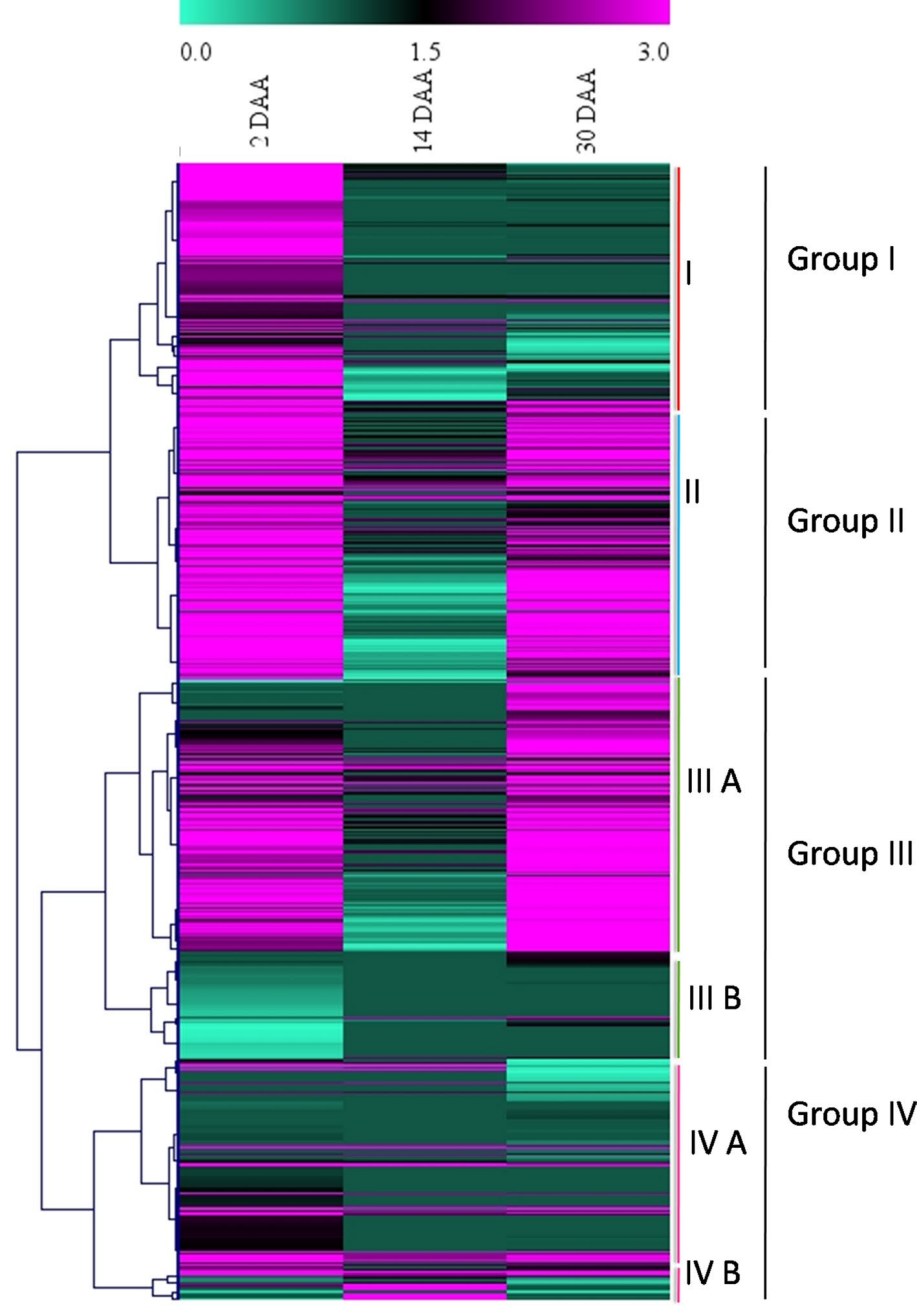
Expression profiles of 698 wheat RING protein genes at three seed developmental stages (2, 14, and 30 day after anthesis, DAA). The data was extracted from RNA-seq data set (https://wheat.pw.usda.gov/GG3/node/237), clustered expression heat map generated by MeV software version 4.9.0 and divided into four major groups according to gene expression patterns represented as color gradient. The color scale indicating largest gene expression values in pink color, intermediate values in black color and the smallest values in green color.

To explore the potential role of RING domain containing proteins during wheat grain development, gene expression values (TPM -Transcripts Per Million) of 1255 RING protein genes at different developmental stages i.e., 2 DAA, 14 DAA and 30 DAA was retrieved from publicly available wheat expression database. Out of the 1255 RING protein genes, 698 were found expressed at any of the three developmental stages suggesting their potential role during grain development. However, 557 genes that were not found expressed at any of the three stages, were considered as insignificant and excluded from this study. Of the 698 RING protein genes, 306 were consistently expressed at all the developmental stages. We found three different patterns of expression including a combination of any two days i.e., (i) 2 and 14 DAA, (ii) 14 and 30 DAA, and (iii) 2 and 30 DAA, these three combination included 22, 3, and 156 genes respectively. Days specific expression of the RING protein genes was also found involving 152, 5, and 54 genes expressed at 2, 14, and 30 DAA (Fig. 4). To analyze the pattern of expression of the identified 698 RING protein genes clustered heat map was generated (Fig. 5, Supplementary Fig. S4). Based on expression pattern genes were clustered into four major groups (Group I, II, III and IV) with two subgroups: Group III (III A, III B) and Group IV (IV A, IV B) (Fig. 5). Group I included majority of genes (123) with high expression levels at 2 DAA. A total of 194 genes with almost same expression patterns at 2 DAA and 30 DAA were clustered in group II. Group III A represented 167 genes that were mainly expressed at 30 DAA compared to 2 DAA. Simultaneously, Group III B included 66 genes that were expressed at 14 and 30 DAA but with lower expression values. Group IV A contained the 133 genes expressed at 2 DAA and 14 DAA and IV B included the 15 genes particularly expressed at 14 DAA. It was observed from this expression analysis majority of RING containing protein genes were having higher expression levels at initial (2 DAA) and late stages (30 DAA) of seed development in comparison to mid stages (14 DAA). This information briefs the involvement of RING protein genes in all stages of seed development and its functional diversity.

Further to examine the putative role of the 698 RING protein genes in amylose biosynthesis during grain development, differential gene expression (DEGs) analysis was performed using the In-house transcriptome data of high (‘TAC 75’) and low (‘TAC 6’) amylose mutant lines along with parent (‘C 306’). The DEGs were analyzed in two groups: Group 1 (‘TAC 75’ vs ‘C306’), and Group 2 (‘TAC 6’ vs ‘C306’). The DEGs were considered significantly expressed following the criteria of log_2_FC > 1 and *p*-value ≤ 0.05. Under this criteria fifteen genes were found significant in Group 1 (nine) and Group 2 (six) respectively (Supplementary Table S5). Two DEGs TraesCS6B02G286500 and TraesCS1A02G206100 in Group 1 (‘TAC 75’ vs ‘C306’) showed significant (seven fold) up- and down-regulated expression respectively. While, the DEG TraesCS2A02G099300 showed significant 11 fold down-regulated expression in group 2 (‘TAC 6’ vs ‘C306’).

The above 15 differentially expressed RING proteins genes in group 1 and group 2 might have potential role in amylose biosynthesis. So to predict the involvement of RING proteins in amylose biosynthesis all 15 DEGs were further validated by qRT-PCR in amylose mutant lines (‘TAC 75’ and ‘TAC 6’) and parent variety (‘C 306’). Considering the non-ubiquitous expression of the RING protein genes throughout the grain development, additional 21 highly expressed RING protein genes previously mined from the publicly available database (http://www.wheat-expression.com/) were also considered for qRT-PCR based expression analysis.

### Analysis and characterization of RING protein gene variants in mutant lines

**Figure 6:**
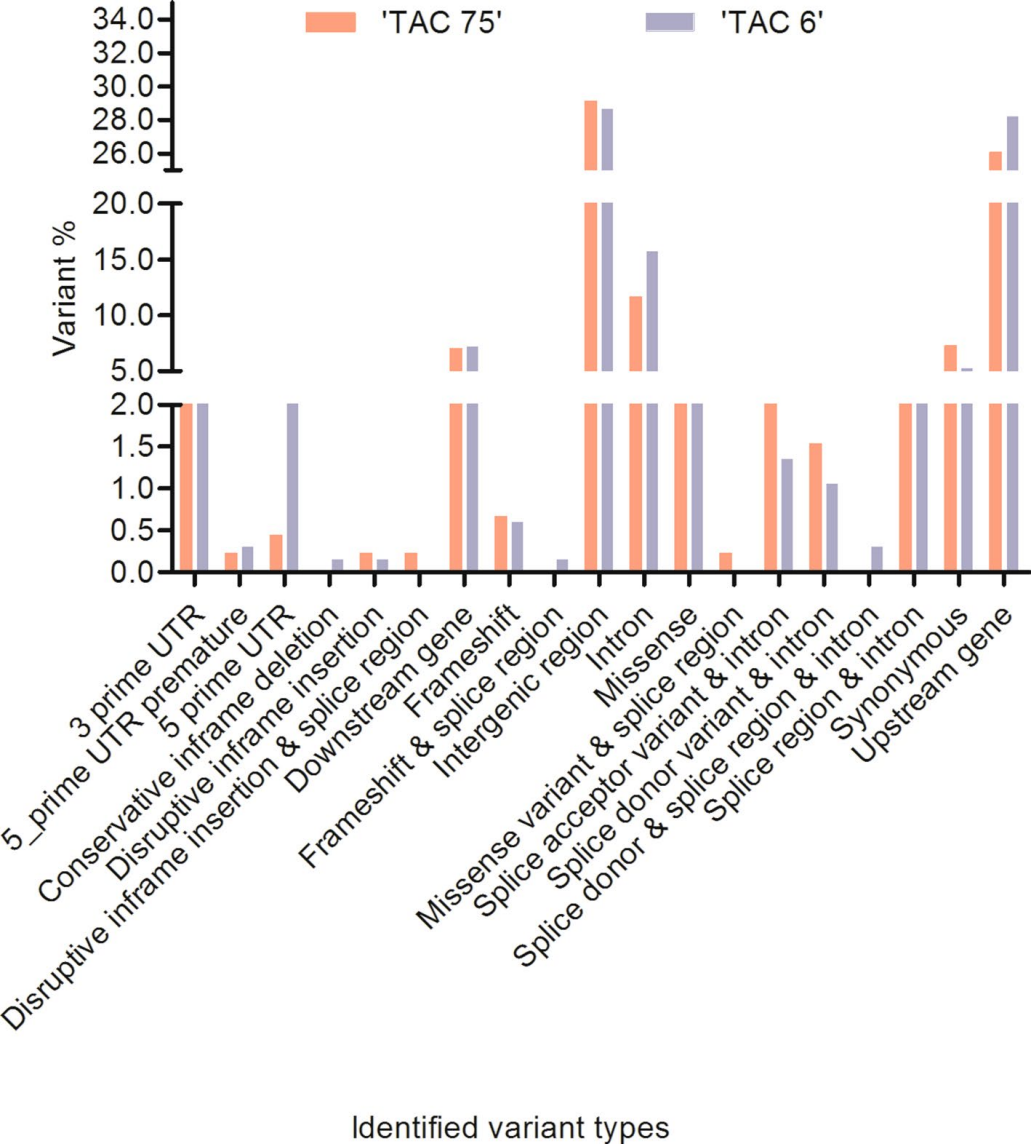
Percent distribution of different genetic variants in mutant lines ‘TAC 75’ and ‘TAC 6’.

Different genotypic variants were identified in ‘TAC 75’ and ‘TAC 6’ in reference to parent variety (‘C 306’), randomly distributed across the wheat genomes (A, B and D). This analysis revealed a total of 457 and 667 variants in 202 and 250 RING protein genes in ‘TAC 75’ and ‘TAC 6’ respectively. Highest number of mutations were located on chromosome 2B and least on 4D in both mutants. Mutations including single nucleotide polymorphisms (SNPs), insertion-deletions (InDels), and multiple nucleotide polymorphisms (MNPs) at different positions involving genic, intergenic, and 3′ and 5′ UTR were identified. The predicted effect of identified variants was characterized into high, low, moderate and modifier variants, in which 26 mutations in ‘TAC 75’ and 23 in ‘TAC 6’ exhibited high variant effect (Supplementary Table S6). Largest number of mutations were identified within intergenic regions (29.1% and 28.64% in ‘TAC 75’ and ‘TAC 6’ respectively) followed by upstream gene variants (26.04% and 28.19% in ‘TAC 75’ and ‘TAC 6’ respectively) and intronic variants (11.6% and 15.7% in ‘TAC 75’ and ‘TAC 6’ respectively), hence amino acids remained unaltered. Conservative, disruptive, splice, synonymous, missense and frameshift variants were found comparatively lower in both ‘TAC 75’ and ‘TAC 6’ (Fig. 6), but some with deleterious effect on proteins. The detail of total number of identified variants listed in Table 2. Mutant characterization indicated the presence of EMS induced specific mutations in RING protein genes identified in high (‘TAC 75’) and low (‘TAC 6’) mutant lines making them unique for the variants. One of the previous studies from our lab^6^, has also characterized the two mutant lines for key enzymes GBSSI and SBEII responsible for amylose biosynthesis and possessing the defined amylose contents.

**Table 2:** Total number of variants identified in different variant types in mutant lines ‘TAC 75’ and ‘TAC 6’.

Variant type	Number of variants	
	‘TAC 75’	‘TAC 6’
3 prime UTR	18	17
5 prime UTR premature start codon gain	1	2
5 prime UTR	2	14
Conservative inframe deletion	0	1
Disruptive inframe insertion	1	1
Disruptive inframe insertion & splice region	1	0
Downstream gene	32	48
Frameshift	3	4
Frameshift & splice region	0	1
Intergenic region	133	191
Intron	53	105
Missense	18	24
Missense & splice region	1	0
Splice acceptor & intron	16	9
Splice donor & intron	7	7
Splice donor & splice region &intron	0	2
Splice region & intron	19	18
Synonymous	33	35
Upstream gene	119	188

### qRT-PCR validation of candidate genes during seed development

**Figure 7:**
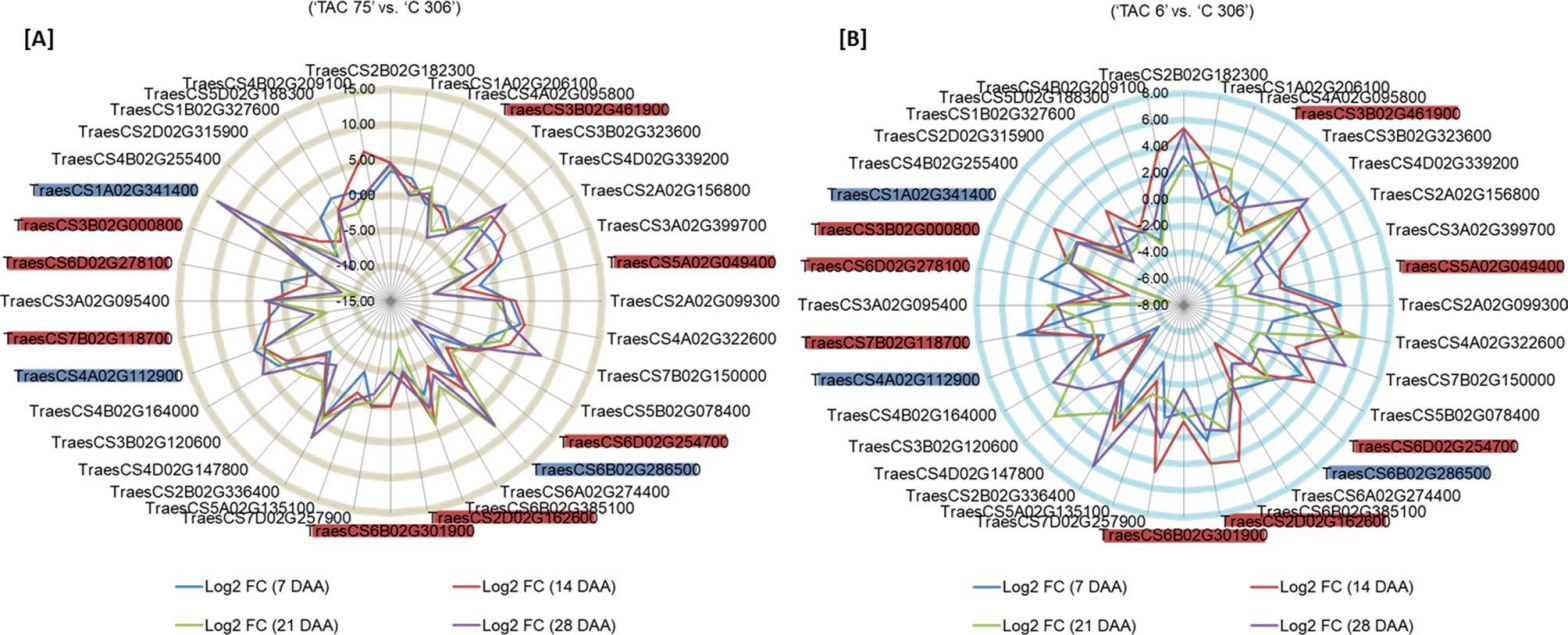
Differential gene expression (Log_2_ fold change) data of 36 RING finger protein genes in three genotypes ‘TAC 75’, ‘TAC 6’ and parent ‘C 306’ at four seed developmental stages (7, 14, 21, and 28 DAA (days after anthesis) using qRT-PCR analysis. Differential gene expression analysis was performed in two groups; (A) Group 1 (‘TAC 75’ vs. ‘C 306) and (B) Group 2 (‘TAC 6’ vs. ‘C 306’). ‘TAC 75’ and ‘TAC 6’ are high and low amylose mutant lines derived from parent variety ‘C 306’ with amylose percent ~ 65%, ~ 7%, ~ 26%, respectively. The 11 candidate RING protein genes are marked with red color (down-regulated in high amylose line) and blue color (up-regulated in high amylose line).

**Figure 8:**
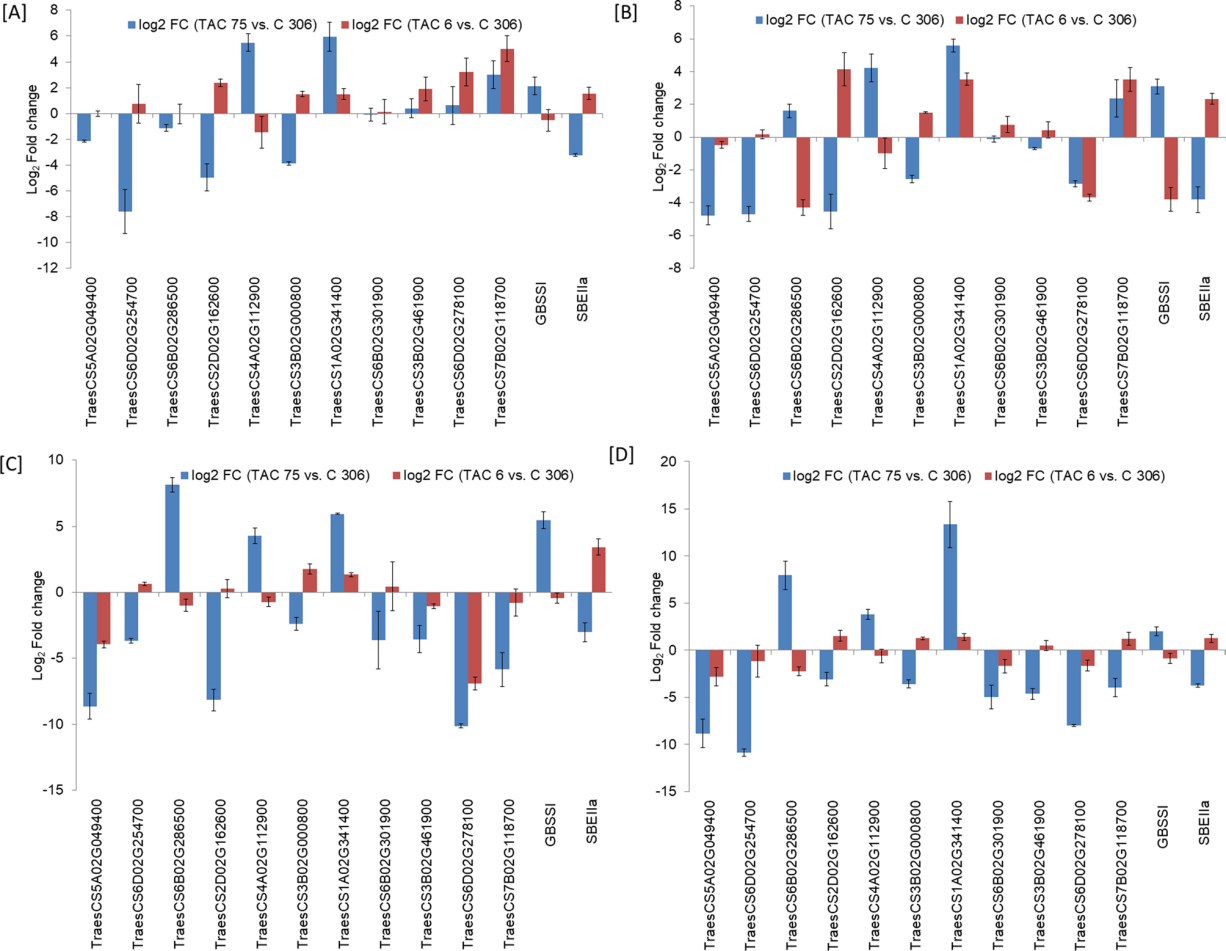
Bar graphs showing the differential gene expression of 11 candidate RING protein genes along with *GBSSI and SBEIIa* using qRT-PCR for amylose biosynthesis at (**A**) 7 DAA, (**B**) 14 DAA, (**C**) 21 DAA and (**D**) 28 DAA in ‘TAC 75’ vs. ‘C 306’ and ‘TAC 6’ vs. ‘C 306’. ‘TAC 75’ and ‘TAC 6’ are high and low amylose mutant lines derived from parent variety ‘C 306’ with amylose percent ~ 65%, ~ 7%, ~ 26%, respectively. The results were obtained using three technical and three biological replicates and are expressed as mean ± SD.

A total of 36 RING protein genes, 15 genes from In-house transcriptome data and 21 from publicly available wheat expression database were considered for qRT-PCR validation along with two key regulators (*GBSSI* and *SBEIIa*) for amylose biosynthesis. Information of gene specific primers used in the study is given in Supplementary Table S7. Gene expression analysis was performed in high amylose (‘TAC 75’), low amylose (‘TAC 6’) mutant lines and parent variety (‘C 306’) at four developmental stages (7, 14, 21 and 28 DAA) to possibly cover all stages of endosperm development. Differential gene expression was analyzed in two combinations, Group 1 (‘TAC 75’ vs. ‘C 306’), and Group 2 (‘TAC 6’ vs. ‘C 306’). All the 36 RING protein genes showed the variable pattern of differential expression among three groups (Supplementary Table S8, Fig. 7). Differential gene expression analysis showed that most of the RING protein genes were down-regulated in high amylose line, when compared to parent (Group 1). Out of the 36 RING protein genes, eight genes showed consistent down-regulated expression in high amylose line at all four developmental stages (Fig. 7). Out of the above eight RING protein genes, four genes (TraesCS6D02G254700, TraesCS2D02G162600, TraesCS3B02G000800, TraesCS5A02G049400) showed significant down-regulated expression (log_2_ FC > 2) at the later stages (21 and 28 DAA) of seed development. However the remaining four genes (TraesCS6B02G301900, TraesCS3B02G461900, TraesCS6D02G278100, TraesCS7B02G118700), were found significantly down-regulated during the early stages (7 and 14 DAA) of seed development (Fig. 8). These results suggest that the down-regulated expression of the above identified eight RING protein genes in high amylose lines, might play role in negative regulation of high amylose biosynthesis.

On the other hand six RING protein genes were found consistently up-regulated in high amylose line in all the four developmental stages (Fig. 7). Of these six genes, two genes (TraesCS4A02G112900, TraesCS1A02G341400) showed differential log_2_ FC  > 2 and at the same time were found comparatively down regulated in low amylose line comparative to the parent (Group 2) (Fig. 8). The RING protein gene TraesCS6B02G286500 was found to be extremely up-regulated at later seed developmental stages (21 and 28 DAA) in Group 1 (Fig. 8). These 3 RING protein genes were found up-regulated when amylose biosynthesis is high hence they might possibly act as positive regulators for amylose biosynthesis.

At the same time expression of two key regulatory enzymes GBSSI and SBEIIa in starch biosynthesis was also analyzed. The *GBSSI* was highly up-regulated and *SBEIIa* was highly down-regulated in high amylose line (Group 1). Whereas we found a vise-versa expression for the same (*GBSSI* and *SBEIIa*) in low amylose line (Group 2) (Fig. 8). The higher and lower expression levels of GBSSI and SBEIIa in high amylose line might be the reason for high amylose starch biosynthesis. The above identified 11 candidate (eight down-regulated and three up-regulated) RING protein genes might be involved in regulation of GBSSI and SBEIIa via ubiquitin-mediated post-translational modifications, either directly or through the modulation of other regulatory factors that has not been studied yet. As the RING proteins are involved in ubiquitin-mediated degradation pathway and can play a positive or negative role in regulation of a biosynthetic pathway^44^. So both the down regulation and up regulation of RING protein genes might be responsible for high amylose by targeting *GBSSI* and *SBEIIa*, respectively.

### Correlation analysis of RING protein genes with starch pathway genes (Pearson’s correlation)

**Figure 9:**
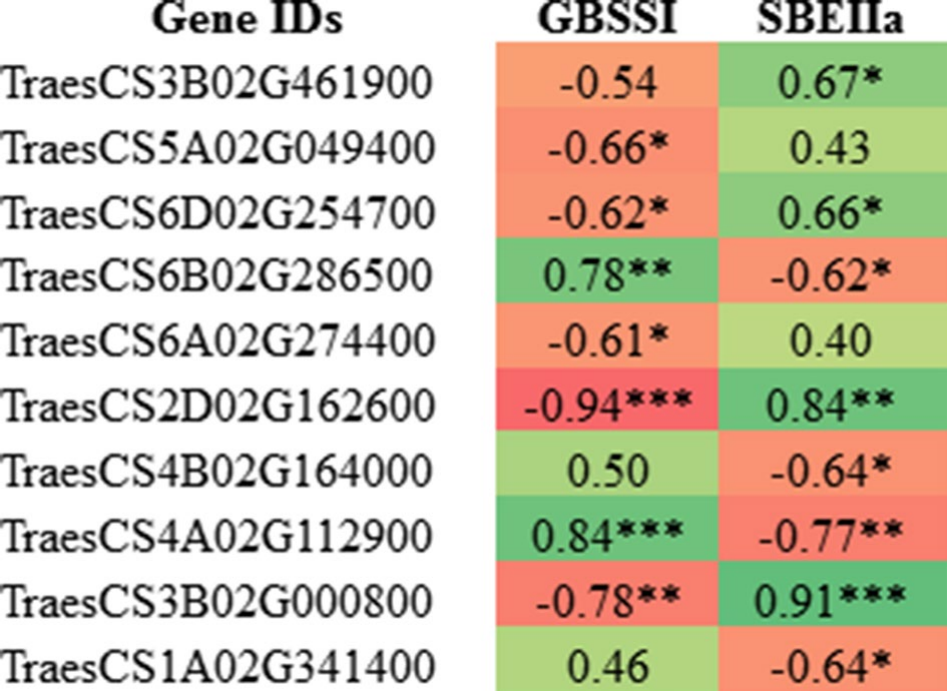
Pair wise Pearson's correlation analysis of 10 RING protein genes with two starch pathway genes *GBSSI* and *SBEIIa*. The significant values of correlation coefficient (r) labeled with *p ≤ 0. 05, **p ≤ 0. 01 and ***p ≤ 0. 001.

Correlation between the normalized gene expression data of 36 RING protein genes and two starch pathway genes *GBSSI* and *SBEIIa* was analyzed. GBSSI and SBEIIa are key enzymes regulating the amylose and amylopectin biosynthesis respectively^7,9^. Therefore, to explore the role of RING protein genes in starch biosynthesis, statistical correlation with key regulatory genes is essential. The pairwise correlation analysis revealed that 55.5% (20) RING protein genes were negatively correlated with *GBSSI*. Out of twenty, only one gene (TraesCS2D02G162600) showed very strong (*r*^2^ ≥ 0.80) and four genes (TraesCS5A02G049400, TraesCS6D02G254700, TraesCS6A02G274400, TraesCS3B02G000800) showed strong (*r*^2^ ≥ 0.60) significant negative correlation with *GBSSI* at *p*-value ≤ 0.05. While only two genes TraesCS4A02G112900 (*r*^2^ = 0.84) and TraesCS6B02G286500 (*r*^2^ = 0.78) showed significant positive correlation with GBSSI at *p*-value ≤ 0.05 (Fig. 9). These seven RING protein genes, five and two with strong negative and positive correlation might be involved in regulation of *GBSSI* expression respectively.

The correlation of 36 RING protein genes with *SBEIIa* showed 16 genes to be negatively correlated and 20 genes to be positively correlated (Fig. 9). The correlation analysis revealed 4 genes (TraesCS4A02G11290, TraesCS6B02G286500, TraesCS4B02G164000 and TraesCS1A02G341400) showing significant strong negative correlation with *SBEIIa* (*r*^2^ ≥ 0.60). However, two genes (TraesCS2D02G162600, TraesCS3B02G000800) showed very strong (*r*^2^ ≥ 0.80) and other two genes (TraesCS6D02G254700, TraesCS3B02G461900) showed significant strong (*r*^2^ ≥ 0.60) positive correlation with *SBEIIa* at *p*-value ≤ 0.05. These eight RING protein genes, four each with strong negative and positive correlation to *SBEIIa* might be involved in the regulation of *SBEIIa* expression.

From the above correlation analysis with *GBSSI* and *SBEIIa*, five RING protein genes (out of 36) were found to have significant negative or positive correlation with both the genes, hence total 10 RING protein genes found to be correlated with GBSSI and SBEIIa (Fig. 9). These results suggest the possible involvement of these RING protein genes in regulation of both GBSSI and SBEIIa at protein levels, through the post-translational modifications via direct or indirect gene regulation.

## Discussion

Many genetic analyses have shown that large number of RING proteins have been identified as positive or negative regulators of various biological processes. By considering their importance in plants, RING proteins were initially identified in *A. thaliana,* encoding ~ 36% RING protein genes out of the ~ 1400 E3 ligases^24,45,46^. In the present study by exploring the hexaploid wheat genome (*Triticum aestivum* L., 2*n* = 6*x* = 42, AABBDD) we identified 1272 potential RING domains in 1255RING finger proteins (Table S1). These potential RING domains were further classified in four major groups—RING-H2 (875), RING-HC (323), RING-v (67) and RING-G (7) (Fig. 1) illustrates that large number of identified RING proteins 79% and 25.39% are RING-H2 and RING-HC types, respectively. Previous studies in *A. thaliana* have shown the presence of ~ 2% RING protein genes with eight types of RING domains- RING-H2 (241), RING-HCa (145), RING-HCb (41), RING-D (10), RING-G (1), RING-S/T (4), RING-v (25), and RING-C2 (10)^24^. We were unable to identify some above previously identified RING domains from *Arabidopsis* and other plant species in wheat genome. We were also not able to differentiate the identified RING-HC group domains in RING-HCa and RING-HCb, all were found RING-HCa type as in rice^37^. Our analysis of additional domains in wheat RING finger proteins identified many protein–protein interaction domains, nucleic acid and ubiquitin binding domains in different organizations that have been previously identified in *Arabidopsis*, apple, *B. rapa*, and *B. oleracea*^24,36–39^. The 29.48% proteins had only RING domain, on the other hand 70.52% were having one or more additional domains other than RING (Supplementary Table S3). The transmembrane domain was most frequently present with RING domain, indicating their role as membrane integral proteins. Copine and NB-ARC domain identified in wheat RING domain containing proteins involved in lipid binding activities and nucleotide binding, respectively^47,48^. In wheat multiple domain of unknown function (DUFs), DUF1117, DUF4792, DUF4793, DUF1232, and DUF3675 were identified rather than only DUF1117 found in *Arabidopsis*, most of the time DUF domain found in combination of transmembrane domains (Supplementary Table S3). The presence of additional domains and their different organizations in RING proteins suggests the role in different biological processes that might be specific for different organisms.

Phylogenetic clustering of RING finger domains clearly demonstrated their evolutionary relationship. All the four RING domains (RING-H2, RING-HC, RING-v, RING-G) clustered with their respective group domains with some exceptions. The intermixing of a specific domain with other might be the reason of variation in protein sequences except the conserved metal ligands, during evolutionary process. This study shows that in spite of different RING types they share some similarities that confer their same origin during evolution. To look more into the evolutionary history of RING domain proteins different orthologous RING proteins can be considered for phylogeny, this will give the clear view of their origin, structural and functional characteristics.

Furthermore, our study focused to find out the genomic locations of RING protein genes and their gene duplication events within three homeologous genomes (A, B and D) in wheat. Wheat RING protein genes were mapped onto all 21 chromosomes. We observed that most of the identified RING protein genes were located on 7D (6. 7%) and less distributed on 4B (3. 5%) chromosome (Supplementary Fig. S3). Wheat (*Triticum aestivum*) is an allohexaploid (2n = 6x  = 42, genome AABBDD) originated by two isolated hybridization events 10, 000 years ago. Hence, wheat contains three genomes from closely related species, assumed having triplicate copies of genes followed by chromosomal doubling to maintain the fertility^49,50^. We identified 4 singleton (not duplicated) and 49, 15, 90 and 1080 RING protein genes as dispersed, proximal, tandem and WGD/segmental duplicated genes, respectively. The RING protein genes on homeologous chromosome 3 and 7 were found whole genome/ segmental duplicated within their A, B, D genome, no collinearity from other chromosomes found. The genes from homeologous chromosome 1 (1A, 1B and 1D) were found collinear within the chromosome and also showed collinearity in large extent with other homeologous chromosome 3 (3A, 3B, and 3D) (Fig. 3). Results of duplication events indicated that in the expansion of RING protein gene family in wheat whole genome duplication (WGD)/segmental duplications has played major role.

The analysis from RNA-seq data revealed that of 1255 RING protein genes, a set of 557 genes showed no expression at any of the three developmental stages, considered as possibly no role in seed development. The 698 RING protein genes that were preferentially expressed at any seed developmental stage (2 DAA, 14 DAA and 28 DAA), suggest possible role in developing wheat seed. The 43.84% genes were found to be expressed in all the above developmental stages, while, remaining revealed seed development specific expression (Fig. 4). The different expression patterns and expression levels of RING protein genes provide information of their functional diversity with different extent in various biological processes. If possible role of these RING proteins is determined, it will be great impact on seed quantity and quality control.

Further, the 698 seed specific RING protein genes were analyzed to illustrate the involvement in amylose biosynthesis. In seeds amylose is the main component for resistant starch, a healthy starch that is beneficial for combating gastrointestinal diseases, diabetes and obesity as well as play important role in food processing. The expression analysis from in house transcriptome data of amylose variants and qRT-PCR data of 36 wheat RING protein genes revealed that RING protein genes were expressed at both initial and late developmental stages, suggesting their putative role throughout the seed development (Supplementary Table S8, Fig. 7). Differential gene expression analysis of 36 RING protein genes in amylose variant genotypes also revealed 8 strongly down-regulated and 3 up-regulated genes in high amylose line. These RING protein genes might play potential role as positive or negative regulators of amylose biosynthesis by acting as effective E3 ligases in ubiquitin-mediated post-translational modifications. As ubiquitin-mediated post-translational modifications are involved in various biological processes including stress^51^, growth^28^, immunity^52^, photosynthesis^53^ and hormone signaling^54^, but their direct role in starch biosynthesis is not yet known. A study in wheat has been shown the role of TaGW2, a RING E3 ligase in seed size and weight. They hypothesized the increased seed weight might be a reason of higher accumulation of starch controlled by TaGW2^33^. This study suggests only the probable role of RING proteins in starch biosynthesis that need to be explored. The correlation studies of 36 RING protein genes were performed with *GBSSI and SBEIIa*, two key enzymes regulating the amylose and amylopectin biosynthesis, respectively^7,9^. Therefore, to explore the role of RING proteins in starch biosynthetic pathway, statistical correlation with key regulatory starch pathway genes was essential. The correlation studies of 36 RING protein genes with *GBSSI* and *SBEIIa* expression data showed 10 strongly correlated RING protein genes, with *GBSSI* and *SBEIIa*. These results indicate the involvement of RING protein genes in the regulation of transcripts levels and further protein levels of *GBSSI* and *SBEIIa*, hence will possibly impact on the amylose biosynthesis via direct regulation or through negative and positive regulators of these enzymes. This study has provided substantial information of RING protein genes, their correlation with starch biosynthetic genes and potential targets for wheat amylose regulation in wheat.

## Conclusion

In the present study, we identified 1255 RING proteins in wheat through in silico approaches. The RING protein genes were found to be distributed all over wheat genome with duplication across the genome. Majority of variations and expansion of RING protein gene family in wheat took place through several duplication events that has contributed for the functional diversification of RING proteins. The expression analysis of 698 RING protein genes during various seed developmental stages revealed their possible involvement in seed development. The identified mutation in RING protein genes could be the reason of amylose variation in wheat mutants. Interestingly, this study demonstrated that RING E3 ligases might play a potential role in the amylose biosynthesis as positive or negative regulators, thus imparting great knowledge for the grain quality enhancement. This whole study would be helpful to reduce the study gap of ubiquitin-mediated post translational modifications of amylose regulatory enzymes and other seed functional traits.

## Materials and methods

### Plant materials

Two mutant lines (M6 generation), ‘TAC 75’ (amylose content ~ 65%) and ‘TAC 6’ (amylose content ~ 7%) developed via EMS mutagenesis along with their parent wheat variety, ‘C 306’ (amylose content ~ 26%) were used in this study^10^.

### Genome-wide identification of RING domain containing proteins in wheat

To identify presumptive wheat RING finger domain containing proteins, two strategies were followed. In the first strategy RING finger proteins of *Oryza sativa*^37^ and *A. thaliana*^24^ were taken as query data set to identify potential wheat orthologs using Ensembl *T. aestivum* protein database (RefSeq 1.1) (https://plants.ensembl.org/Triticum_aestivum/Info/Index). The second strategy used to identify proteins containing RING [ZnF-RING (IPR001841)] and Pfam domain [zf-C3HC4 (PF00097)] of InterProScan against the same *T. aestivum* protein database^55^. To confirm the presence of RING domains in the retrieved irredundant protein sequences from both the strategies, all the sequences were analyzed by Single Modular Architecture Research Tool (SMART) database (http://smart.embl-heidelberg.de/) integrated with the Pfam domain database. Further, the confirmation of RING proteins was done by manual inspection of each protein sequences for the presence of any of the eight conserved zinc coordinating metal ligands (Cys or His) and the distance between each metal ligand. The identified RING domain containing proteins were classified into groups based on the amino acid residues at metal ligand positions and the residues number between each metal ligand^24^. Proteins confirmed by SMART database but lacking one or more metal ligands were not considered in any group and they were classified as incomplete RING domain containing proteins. Sequence logos of multiple domain alignments from each representing group were created using online WebLogo tool (https://weblogo.berkeley.edu/logo.cgi).

### Multiple sequence alignment and phylogenetic analysis of RING domains

Multiple sequence alignment was performed on the sequences of the identified RING domain that were extracted from their respective protein sequences in Clustal X (Version 2.1; http://www.clustal.org/clustal2/). The parameters for pairwise alignment were 40 gap opening and 0.8 gap extension penalty using PAM350 protein matrix and other criteria at default setting. Further, the aligned RING domain sequences were manually edited in BioEdit software to correctly align eight metal ligand residues and were used for performing phylogenetic analysis by MEGA X software (Version 10.1). Phylogenetic tree was generated by Neighbor-Joining (NJ) algorithm with 1000 bootstrap for significant evaluation of phylogenetic tree. Evolutionary distances were inferred using Jones–Taylor–Thornton (JTT) model and for gap profiling pairwise gap deletion model was used.

### Identification of additional RING domains

To identify the presence of other possible domains in RING finger proteins, SMART database considering Pfam domains and signal peptide was used. RING containing proteins were classified into different groups based on the presence or absence and organization of the identified additional domains. Species with the same protein domain architectures as that in wheat RING domain containing proteins were identified using NCBI BLASTp search in model organisms only.

### Chromosomal location of wheat RING protein genes

The chromosomal locations of wheat RING protein genes were retrieved from GFF file of *T. aestivum* genome database downloaded from Ensembl plants (ftp://ftp.ensemblgenomes.org/pub/plants/release-46/gff3/triticum_aestivum). The genes that were not mapped on any chromosome were excluded from the analysis. Chromosomal location of genes was drawn onto *T. aestivum* 21 chromosomes by Circos software (Version 0. 69-9). All duplication events such as dispersed, proximal, tandem, whole genome (WGD)/segmental and singleton (not duplicated) in RING protein genes were predicted by MCScanX tool^56^. WGD/segmental duplications are shown in comparative analysis of synteny of RING protein genes on the entire wheat chromosomes using Circos software.

### Expression analysis of RING containing genes in developing wheat seeds

The expression profiles of wheat RING containing genes were analyzed at three seed developmental stages (2, 14, and 30 DAA) in the previously reported RNA-seq data^57^ using RefSeq1.1 nucleotide database in wheat expression browser (http://www.wheat-expression.com/). Normalized transcripts per million (TPM) values were used to analyze the expression pattern at different developmental stages. Clustered heat map showing expression profile of wheat RING containing genes was generated by MeV software^58^. The hierarchical clustering was performed using uncentered pearson correlation distance matric and complete linkage clustering method. Additionally, the differential gene expression of RING protein genes was also analyzed using the in-house transcriptome data of mutant lines (‘TAC 75’ and ‘TAC 6’) and parent (‘C 306’) in three biological replicates. For the whole transcriptome data analysis CLC genomics workbench (CLC GWB, Version 20) was used. High-quality paired end reads were aligned against annotated wheat genome reference assembly (IWGSC RefSeq1.0). Differential expression analysis of genes was performed using normalized RPKM (reads per kilobase of exon model per million reads) values and genes presenting *p*-value ≤ 0.05 were considered as significant and were further used for downstream analysis. The differential gene expression values of RING containing genes were acquired by DESeq2 (v1.22.1) and genes with fold change > 2 were retained for further analysis. The RING containing genes were selected based on expression values from the public domain and in-house transcriptome data and were used for qRT-PCR validation in mutant lines along with parent.

### RING protein genes variant identification in mutant lines

For variant analysis of RING protein genes in mutants ‘TAC 75’ and ‘TAC 6’ the Illumina generated reads were used and high quality (Q30) reads were assembled and mapped against the wheat reference genome (Ensemble release 49) using the BWA-MEM tool (version 0.7.17) [17, Paolacci et al.^59^)]. This alignment file (.bam) was further used for variant identification by Samtools using parameters described by Paolacci et al.^59^. Genetic variants like SNPs, insertions, deletions and MNPs were annotated using SnpEff (version 4.3t) tool^25^ against the wheat reference genome. The in-house Perl scripts were used to analyse the distribution of variants (SNPs and Indels) in mutants ‘TAC 6’ and ‘TAC 75’ among identified 698 RING protein genes possibly involved in grain development.

### qRT-PCR validation of candidate RING protein genes during seed development

Genome-specific primers of candidate RING protein genes, limiting factor genes of starch biosynthesis (*GBSSI* and *SBEIIa*)^10^, and wheat ADP-Ribosylation Factor (ARF) as an internal control^60^ were designed using OligoCalc (http://biotools.nubic.northwestern.edu/OligoCalc.html). RNAs extracted from primary individual spikes from three biological replicate samples were used for qRT-PCR analysis. Spikes were tagged on the first day of anthesis and harvested at 7, 14, 21, and 28 day after anthesis (DAA), frozen in liquid nitrogen, and stored at − 80 °C till further use. RNA was isolated by introducing minor changes in Trizol method^20^ and cDNA was synthesized using iScriptgDNA clear cDNA synthesis kit (Bio-Rad, USA). Three biological replicates with their three technical replicates were used for qRT-PCR analysis using 1:10 diluted cDNA and Fast SYBR Green Master Mix as per manufacturer’s instruction in 7500 Fast Real-Time PCR System.

### Statistical analysis

To analyze the relationship among wheat RING protein genes and key regulatory genes of starch metabolism (*GBSSI, SBEIIa*), pair wise Pearson’s correlation coefficient (*r*) and their significance test was performed using Graph Pad Prism (version 5) with *p*-value criteria ≤ 0.05. Correlation analysis was done using their mean normalized Ct values (at four different seed developmental stages).

### Ethics declarations

All experiments were performed in accordance with relevant institutional guidelines.

## Supplementary Information

Supplementary Information 1.

Supplementary Table S3.

Supplementary Table S6.

## References

[1] Gao S, Wang W, Guo T, Han J (2003). C-N metabolic characteristics in flag leaf and starch accumulating developments in seed during grain filling stage in two winter wheat cultivars with different spike type. Acta Agron. Sin..

[2] Tester RF, Karkalas J, Qi X (2004). Starch structure and digestibility enzyme-substrate relationship. World’s Poult. Sci. J..

[3] Brouns F, Kettlitz B, Arrigoni E (2002). Resistant starch and ‘the butyrate revolution’. Trends Food Sci. Technol..

[4] Van Hung P, Maeda T, Morita N (2006). Waxy and high-amylose wheat starches and flours-characteristics, functionality and application. Trends Food Sci. Technol..

[5] Slade AJ (2012). Development of high amylose wheat through TILLING. BMC Plant Biol..

[6] Kumar P (2018). Pivotal role of bZIPs in amylose biosynthesis by genome survey and transcriptome analysis in wheat (*Triticum aestivum* L.) mutants. Sci. Rep..

[7] Regina A (2006). High-amylose wheat generated by RNA interference improves indices of large-bowel health in rats. Proc. Natl. Acad. Sci. USA..

[8] Wang Z, Li W, Qi J, Shi P, Yin Y (2014). Starch accumulation, activities of key enzyme and gene expression in starch synthesis of wheat endosperm with different starch contents. J. Food Sci. Technol..

[9] Sestili F (2010). Increasing the amylose content of durum wheat through silencing of the SBEIIa genes. BMC Plant Biol..

[10] Mishra A, Singh A, Sharma M, Kumar P, Roy J (2016). Development of EMS-induced mutation population for amylose and resistant starch variation in bread wheat (*Triticum aestivum*) and identification of candidate genes responsible for amylose variation. BMC Plant Biol..

[11] Sehnke PC, Chung HJ, Wu K, Ferl RJ (2001). Regulation of starch accumulation by granule-associated plant 14-3-3 proteins. Proc. Natl. Acad. Sci. USA..

[12] Hendriks JHM, Kolbe A, Gibon Y, Stitt M, Geigenberger P (2003). ADP-glucose pyrophosphorylase is activated by posttranslational redox-modification in response to light and to sugars in leaves of arabidopsis and other plant species. Plant Physiol..

[13] Tetlow IJ (2004). Protein phosphorylation in amyloplasts regulates starch branching enzyme activity and protein–protein interactions. Plant Cell.

[14] Hershko A (2005). The ubiquitin system for protein degradation and some of its roles in the control of the cell division cycle. Cell Death Differ..

[15] McNeilly D, Schofield A, Stone SL (2018). Degradation of the stress-responsive enzyme formate dehydrogenase by the RING-type E3 ligase Keep on Going and the ubiquitin 26S proteasome system. Plant Mol. Biol..

[16] Hershko A, Ciechanover A (1992). The ubiquitin system for protein degradation. Annu. Rev. Biochem..

[17] Seol JH (1999). Cdc53/cullin and the essential Hrt1 RING-H2 subunit of SCF define a ubiquitin ligase module that activates the E2 enzyme Cdc34. Genes Dev..

[18] Moon J, Parry G, Estelle M (2004). The ubiquitin-proteasome pathway and plant development. Plant Cell.

[19] Pickart CM (2001). Mechanisms underlying ubiquitination. Annu. Rev. Biochem..

[20] Lorick KL (1999). RING fingers mediate ubiquitin-conjugating enzyme (E2)-dependent ubiquitination. Proc. Natl. Acad. Sci. USA..

[21] Zheng N (2002). Structure of the Cul1-Rbx1-Skp1-F boxSkp2 SCF ubiquitin ligase complex. Nature.

[22] Furukawa M, He YJ, Borchers C, Xiong Y (2003). Targeting of protein ubiquitination by BTB-Cullin 3-Roc1 ubiquitin ligases. Nat. Cell Biol..

[23] Kosarev P, Mayer KFX, Hardtke CS (2002). Evaluation and classification of RING-finger domains encoded by the Arabidopsis genome. Genome Biol..

[24] Stone SL (2005). Functional analysis of the RING-type ubiquitin ligase family of Arabidopsis. Plant Physiol..

[25] McNellis TW (1994). Genetic and molecular analysis of an allelic series of cop1 mutants suggests functional roles for the multiple protein domains. Plant Cell.

[26] Liu H (2017). The RING-Type E3 Ligase XBAT35.2 is involved in cell death induction and pathogen response. Plant Physiol..

[27] Dong CH, Agarwal M, Zhang Y, Xie Q, Zhu JK (2006). The negative regulator of plant cold responses, HOS1, is a RING E3 ligase that mediates the ubiquitination and degradation of ICE1. Proc. Natl. Acad. Sci. USA..

[28] Stone SL, Williams LA, Farmer LM, Vierstra RD, Callis J (2006). KEEP ON GOING, a RING E3 ligase essential for Arabidopsis growth and development, is involved in abscisic acid signaling. Plant Cell.

[29] Lim CW, Baek W, Lee SC (2017). The pepper RING-type E3 ligase CaAIRF1 regulates ABA and drought signaling via CaADIP1 protein phosphatase degradation. Plant Physiol..

[30] Serrano M, Guzmán P (2004). Isolation and gene expression analysis of Arabidopsis thaliana mutants with constitutive expression of ATL2, an early elicitor-response RING-H2 zinc-finger gene. Genetics.

[31] Ling Q, Huang W, Baldwin A, Jarvis P (2012). Chloroplast biogenesis is regulated by direct action of the ubiquitin-proteasome system. Science.

[32] Song XJ, Huang W, Shi M, Zhu MZ, Lin HX (2007). A QTL for rice grain width and weight encodes a previously unknown RING-type E3 ubiquitin ligase. Nat. Genet..

[33] Bednarek J (2012). Down-regulation of the TaGW2 gene by RNA interference results in decreased grain size and weight in wheat. J. Exp. Bot..

[34] Geng J (2017). TaGW2-6A allelic variation contributes to grain size possibly by regulating the expression of cytokinins and starch-related genes in wheat. Planta.

[35] Sestili F (2019). Enhancing grain size in durum wheat using RNAi to knockdown GW2 genes. Theor. Appl. Genet..

[36] Li H (2011). PERSISTENT TAPETAL CELL1 encodes a PHD-finger protein that is required for tapetal cell death and pollen development in rice. Plant Physiol..

[37] Lim SD (2010). A gene family encoding RING finger proteins in rice: Their expansion, expression diversity, and co-expressed genes. Plant Mol. Biol..

[38] Alam I (2017). Genome-wide identification, evolution and expression analysis of RING finger protein genes in *Brassica rapa*. Sci. Rep..

[39] Yang YQ, Lu YH, King J (2018). Genome-wide survey, characterization, and expression analysis of RING finger protein genes in *Brassica oleracea* and their syntenic comparison to *Brassica rapa* and *Arabidopsis thaliana*. Genome.

[40] Buljan M, Bateman AG (2009). The evolution of protein domain families. Biochem. Soc. Trans..

[41] Yuan X (2013). Global analysis of ankyrin repeat domain C3HC4-type RING finger gene family in plants. PLoS ONE.

[42] Whittaker CA, Hynes RO (2002). Distribution and evolution of von Willebrand/integrin A domains: Widely dispersed domains with roles in cell adhesion and elsewhere. Mol. Biol. Cell.

[43] Mishra AK, Puranik S, Prasad M (2012). Structure and regulatory networks of WD40 protein in plants. J. Plant Biochem. Biotechnol..

[44] Duplan V, Rivas S (2014). E3 ubiquitin-ligases and their target proteins during the regulation of plant innate immunity. Front. Plant Sci..

[45] Vierstra RD (2009). The ubiquitin-26S proteasome system at the nexus of plant biology. Nat. Rev. Mol. Cell Biol..

[46] Kraft E (2005). Genome analysis and functional characterization of the E2 and RING-type E3 ligase ubiquitination enzymes of Arabidopsis. Plant Physiol..

[47] Tomsig JL, Creutz CE (2002). Copines: A ubiquitous family of Ca2+-dependent phospholipid-binding proteins. Cell. Mol. Life Sci..

[48] Tameling WIL (2002). The tomato R gene products i–2 and Mi-1 are functional ATP binding proteins with ATPase activity. Plant Cell.

[49] Wang J (2011). Variability of gene expression after polyhaploidization in wheat (*Triticum aestivum* L.). G3.

[50] Huo N (2018). Gene duplication and evolution dynamics in the homeologous regions harboring multiple prolamin and resistance gene families in hexaploid wheat. Front. Plant Sci..

[51] Lee HK (2009). Drought stress-induced Rma1H1, a RING membrane-anchor E3 ubiquitin ligase homolog, regulates aquaporin levels via ubiquitination in transgenic arabidopsis plants. Plant Cell.

[52] Zhang L, Du L, Shen C, Yang Y, Poovaiah BW (2014). Regulation of plant immunity through ubiquitin-mediated modulation of Ca2+-calmodulin-AtSR1/CAMTA3 signaling. Plant J..

[53] Agetsuma M, Furumoto T, Yanagisawa S, Izui K (2005). The ubiquitin-proteasome pathway is involved in rapid degradation of phosphoenolpyruvate carboxylase kinase for C4 photosynthesis. Plant Cell Physiol..

[54] Kelley DR, Estelle M (2012). Ubiquitin-mediated control of plant hormone signaling. Plant Physiol..

[55] Jones P (2014). InterProScan 5: Genome-scale protein function classification. Bioinformatics.

[56] Wang Y (2012). MCScanX: A toolkit for detection and evolutionary analysis of gene synteny and collinearity. Nucleic Acids Res..

[57] Choulet F (2014). Structural and functional partitioning of bread wheat chromosome 3B. Science.

[58] Howe EA, Sinha R, Schlauch D, Quackenbush J (2011). RNA-Seq analysis in MeV. Bioinformatics.

[59] Paolacci AR, Tanzarella OA, Porceddu E, Ciaffi M (2009). Identification and validation of reference genes for quantitative RT-PCR normalization in wheat. BMC Mol. Biol..

[60] Rio DC, Ares M, Hannon GJ, Nilsen TW (2010). Purification of RNA using TRIzol (TRI Reagent). Cold Spring Harb. Protoc..

[61] Bork P (1993). Hundreds of ankyrin-like repeats in functionally diverse proteins: Mobile modules that cross phyla horizontally?. Proteins Struct. Funct. Bioinform..

[62] Li S (1998). Identification of a novel cytoplasmic protein that specifically binds to nuclear localization signal motifs. J. Biol. Chem..

[63] Tomsig JL, Creutz CE (2002). Copines: A ubiquitous family of Ca 2+-dependent phospholipid-binding proteins. Cell. Mol. Life Sci..

[64] Caruthers JM, Johnson ER, McKay DB (2000). Crystal structure of yeast initiation factor 4A, a DEAD-box RNA helicase. Proc. Natl. Acad. Sci. USA.

[65] Iyer LM, Babu MM, Aravind L (2006). The HIRAN domain and recruitment of chromatin remodeling and repair activities to damaged DNA. Cell Cycle.

[66] Lu Z, Xu S, Joazeiro C, Cobb MH, Hunter T (2002). The PHD domain of MEKK1 acts as an E3 ubiquitin ligase and mediates ubiquitination and degradation of ERK1/2. Mol. Cell.

[67] van der Biezen EA, Jones JD (1998). The NB-ARC domain: A novel signalling motif shared by plant resistance gene products and regulators of cell death in animals. Curr. Biol..

[68] Van Dyck L, Pearce DA, Sherman F (1994). PIM1 encodes a mitochondrial ATP-dependent protease that is required for mitochondrial function in the yeast *Saccharomyces cerevisiae*. J. Biol. Chem..

[69] Sheng Y (2008). Molecular basis of Pirh2-mediated p53 ubiquitylation. Nat. Struct. Mol. Biol..

[70] Leng RP (2003). Pirh2, a p53-induced ubiquitin-protein ligase, promotes p53 degradation. Cell.

[71] Worthington MT, Amann BT, Nathans D, Berg JM (1996). Metal binding properties and secondary structure of the zinc-binding domain of Nup475. Proc. Natl. Acad. Sci. USA.

[72] Ponting CP, Blake DJ, Davies KE, Kendrick-Jones J, Winder SJ (1996). ZZ and TAZ: New putative zinc fingers in dystrophin and other proteins. Trends Biochem. Sci..

[73] Bürglin TR (2008). Evolution of hedgehog and hedgehog-related genes, their origin from Hog proteins in ancestral eukaryotes and discovery of a novel Hint motif. BMC Genom.s.

[74] Kang RS (2003). Solution structure of a CUE-ubiquitin complex reveals a conserved mode of ubiquitin binding. Cell.

[75] Yaffe MB, Elia AEH (2001). Phosphoserine/threonine-binding domains. Curr. Opin. Cell Biol..

[76] Zhang B (2008). GIDE is a mitochondrial E3 ubiquitin ligase that induces apoptosis and slows growth. Cell Res..

