# Supplementary material for: Genome-wide analysis of RING-type E3 ligase family identifies potential candidates regulating high amylose starch biosynthesis in wheat (Triticum aestivum L.)
Source: Sci Rep. 2021 Jun 1;11:11461. doi: 10.1038/s41598-021-90685-7 (PMC8169666; doi:10.1038/s41598-021-90685-7)
Supplement: Supplementary file 1 — Supplementary Information 1. [file 41598_2021_90685_MOESM1_ESM.pdf]

Supplemental Table S1. Analysis and classification (based on the amino acids (Cys/His) on metal ligand positions) and subcellular localization of in-silico identified 1,272 RING domains predicted by CELLO online tool.

Identifiers	RING type	Protein size (amino acids)	Domain start	Domain end	RING domain	Localization	Description
TraesCS1A02G002000	H2	377	234	274	CPICLEEFKVGNEVRGLPCAHNFHVECIDQ WLRLNVKCPRC	Plasma Membrane	
TraesCS1A02G005700b	HC	792	143	181	CVFCMKLPERPVTTPCGHNFCLKCFEKWV NSGKRTCGKC	Nuclear	SRA-YDG superfamily
TraesCS1A02G005700c	HC	792	514	570	CSICKNLMKEPLTTPCGHNFCKACLTAAFG DEGSMRERSRGGRTLRAQKIVKNCPSC	Nuclear	SRA-YDG superfamily
TraesCS1A02G025700	HC	626	475	519	CVICWTDFSSSRGILPCGHRFCYSCIQGWA DCLSSRGKVSTCPLC	Nuclear	BRCT domain superfamily
TraesCS1A02G029800	H2	399	205	245	CAVCKEPFELGAEAREMPCAHIYHEDCILP WLQLRNSCPVC	Nuclear	
TraesCS1A02G084200	HC	869	815	854	CVMCLSEEMSVVFLPCAHQVVCAKCNDLH VKQGMKECPSC	Nuclear	Zinc finger, RING/FYVE/PHD-type
TraesCS1A02G088100	HC	514	463	501	CVICLDAPVEGACIPCGHMAGCMSCLKDIE SKKWGCPIC	Nuclear	Ankyrin repeat-containing domain superfamily
TraesCS1A02G090900	H2	177	83	125	CLICFDDFVPGCYKKIRTMDCTHSFHQRCIF DWLLIDRRCPLC	Nuclear	
TraesCS1A02G107600	H2	537	488	528	CIICLEEYKHKDSLGKLKCGHDFHAGCIKK WLEVKNACPVC	Nuclear	Zinc finger, RING/FYVE/PHD-type
TraesCS1A02G112600	HC	410	349	386	CAICQEKMHTPILLQCKHIFCEDCASEWLE RERTCPLC	Plasma Membrane	

Identifiers	RING type	Protein size (amino acids)	Domain start	Domain end	RING domain	Localization	Description
TraesCS1A02G118800	H2	420	347	387	CCICLARYSNNDDLRELPCTHFFHKECVDKWLKINALCPLC	Plasma Membrane	
TraesCS1A02G126200	H2	238	151	192	CAVCLAVMVDGEAARRLPRCMHVFHRSCVDVWLREHSTCPVC	Chloroplast	
TraesCS1A02G133300	H2	114	56	93	CIICREEMTTAKKLLCGHLFHVHCLRSWRERQHTCPTC	Extracellular	Zinc finger, RING/FYVE/PHD-type
TraesCS1A02G137400	HC	453	392	429	CAICQEKMHVPVLLRCKHIFCEDCVSEWFERERTCPLC	Plasma Membrane	
TraesCS1A02G141900	HC	236	139	176	CGICLEVNAKIVLPDCTHSLCLRCFEEWNAKSKSCPFC	Cytoplasmic	Zinc finger, RING/FYVE/PHD-type
TraesCS1A02G143300	H2	173	54	96	CPVCFEYLFDSTMDISVLHCGHTIHLECLNEMRVHHHFSCPVC	Extracellular	
TraesCS1A02G146700	H2	649	64	107	CAICLGGMRPGHGHALFTAECSHKFHFRCISSNVQHGNRACPIC	Chloroplast	von Willebrand factor A-like domain superfamily
TraesCS1A02G158400	v	269	127	174	QCRVCQQKSEEPLVDLGCRCRGDLSKAHRTCIDVWFRTRGSNKCEICQ	Plasma Membrane	Zinc finger, RING/FYVE/PHD-type
TraesCS1A02G162400	H2	370	230	270	CAVCKEDFSPGDGAKQMPCKHIYHADCIVPWLELHNSCPIC	Nuclear	
TraesCS1A02G169300	HC	246	150	187	CGICMEMNSKVVLPNCTHAMCLRCYQDWNSRSQSCPFC	Nuclear	Zinc finger, RING/FYVE/PHD-type
TraesCS1A02G177600	HC	1041	995	1029	CKVCFESATAAVLLPCRHFCLCKPCALACSECPLC	Nuclear	Kinesin motor domain

Identifiers	RING type	Protein size (amino acids)	Domain start	Domain end	RING domain	Localization	Description
TraesCS1A02G192600	H2	211	124	165	CAVCLSEFAPGDAVRPLTVCRHAFHAACID SWLGAHTTCPVC	Plasma Membrane	
TraesCS1A02G198000	HC	418	170	210	CNVCFDMADDPVVTKCGHLFCWDCLYQW LHVHSNHRECPVC	Nuclear	
TraesCS1A02G201700	H2	249	161	202	CAICLTEFGEREEVRVMPQCGHGFHVDCV DTWLRSNSSCPSC	Nuclear	
TraesCS1A02G205100	H2	246	187	228	CSVCLQDLEVGESARRLPGCRHVFHAPCID RWLVRHASCPLC	Chloroplast	
TraesCS1A02G206100	v	261	16	69	QCRICHEEEGDDGCATATAMESPCGCSGSL KYAHRGCVQRWCDEKGSTVCEICL	Extracellular	Protein of unknown function DUF3675
TraesCS1A02G207000	H2	206	110	151	CPVCLEAFGDDDGVKVVPACGHVFHAPCI DRWLDVRNSCPVC	Chloroplast	
TraesCS1A02G215100	H2	370	143	184	CAVCQCVFCADDELRLLPACRHAFHSGCV DPWLRANPSCPLC	Nuclear	
TraesCS1A02G215300	HC	324	270	311	CRACRLRPATTVLLPCRHLCVCAACDPGAS ASLAAAAACPAC	Nuclear	E3 ubiquitin-protein ligase BOI-like
TraesCS1A02G225700	v	227	25	74	QCRICHEEEDESSAAMESPCACSGSLKYAH RGCVQRWCDEKGSTLCEICL	Nuclear	Protein of unknown function DUF3675
TraesCS1A02G228500	H2	187	119	160	CAVCIVEFRDGDLARLLPRCGHRFHAACV DAWLHLHSTCPLC	Extracellular	
TraesCS1A02G231700	H2	211	143	184	CAVCIVEFRDGDLARLLPRCGHRFHAACV DAWLHLHSTCPLC	Chloroplast	
TraesCS1A02G243200	H2	520	463	504	CYICLVEYEEGDCVRILPCNHEFHLTCVDK WLKEIHRVCPLC	Nuclear	
TraesCS1A02G255600	H2	290	217	259	CAICREEMAPGRGGVCVLRPCGHRFHWPC ALRWLARRNTCPCC	Plasma Membrane	

Identifiers	RING type	Protein size (amino acids)	Domain start	Domain end	RING domain	Localization	Description
TraesCS1A02G263800	HC	823	16	64	CPVCLSPFDDSSVVPRVLPCGHSLCGSCISSLPPASASAAASSLRCPLC	Plasma Membrane	WD4-repeat-containing domain superfamily
TraesCS1A02G274500	H2	201	119	160	CAICLAEFERGEQVRVLPRCNHGFHALCIDRWLAARPTCPTC	Plasma Membrane	
TraesCS1A02G280300	v	247	127	180	NCRICHLGLESAAAESGAGITLGCSCKGDLSYSHKQCAETWFKIRGNKTCEICS	Nuclear	Zinc finger, RING-CH-type
TraesCS1A02G295900	H2	193	127	168	CSICISALVAGDKVKALPPCGHCFHPECVDAWLRSQPSCPLC	Plasma Membrane	
TraesCS1A02G296100	HC	559	238	278	CNICFEMADEPVVTSCGHLFCWPCLYQWLHIHSTHKECPVC	Nuclear	
TraesCS1A02G300400	H2	192	120	161	CAICLGEFVDGEKVRVLPQCGHGFHVRCVDAWLVSHSSCPTC	Plasma Membrane	
TraesCS1A02G300500	H2	197	122	163	CAICLGEFVDGEKVRVLPQCDHGFHVRCVDAWLVSHSSCPTC	Plasma Membrane	
TraesCS1A02G300600	H2	195	120	161	CAICLGEFVDGEKVRVLPRCAHGFHVRCVDAWLVSHGSCPTC	Plasma Membrane	
TraesCS1A02G311600	H2	216	162	203	CAVCLGEVEKGETVKRLPACLHMFHQQCIDPWLHEHSTCPVC	Plasma Membrane	
TraesCS1A02G315400	HC	500	126	165	CPICLGIIRKTRTVMECLHRFCRDCIDKSMRLGNNECPAC	Nuclear	
TraesCS1A02G321800	H2	388	332	373	CPVCLEPLESDAASWPGCSSPHVFHGTCLEGTLKESEKCPIC	Nuclear	
TraesCS1A02G322000	H2	322	270	311	CPICFELLKSDIAVWPGCLRPHVFHGECLLLALKASEMCPLC	Nuclear	
TraesCS1A02G328300	v	710	32	80	QCRICRLPAEAGRPLRHPCACRGSIRFVHDDCQLRWLATRQGPRCELCN	Plasma Membrane	

Identifiers	RING type	Protein size (amino acids)	Domain start	Domain end	RING domain	Localization	Description
TraesCS1A02G329600	HC	991	16	53	CPICLSLLRSAVSVTCNHIFCNHCLTESMKS SSTCPVC	Nuclear	BRCT domain superfamily
TraesCS1A02G341400	HC	374	322	361	CVVCCDARRDCFFMPCGHSATCHACGTKI VEGDASCCPFC	Plasma Membrane	Domain of unknown function DUF4793
TraesCS1A02G346900	H2	199	124	165	CSICLCEYRDGEMQRIMPECRHRFHLMCLD AWLRRSASCPVC	Extracellular	
TraesCS1A02G373200	H2	349	302	342	CSICQEEFEASEEIGRLSCGHGYHVHCIKQW LSRKNACPLC	Nuclear	
TraesCS1A02G374800	H2	1214	1097	1138	CPICCDFLFTSSAAVRGLPCGHFMHSACFQ AYTCSHYTCPIC	Nuclear	
TraesCS1A02G378100	v	257	57	104	ECRICQEDGDETCMEAPCSCKGSLKYAHRT CVQRWCDEKGDTICEICL	Plasma Membrane	Protein of unknown function DUF3675
TraesCS1A02G398300	H2	510	462	502	CIICQEEFEARELVGTLDCGHKYHEACIKQ WLMVKNLCPIC	Nuclear	
TraesCS1A02G422300	H2	154	95	136	CAICLEALVAGAECSEVPACRHVFHRGCLA LWIKSKSTCPLC	Extracellular	
TraesCS1A02G430800	v	661	30	78	LCRICRLPAEAERPLRSPCACRGSIRFVHDE CQLRWIAARGKPLCEVCN	Plasma Membrane	Zinc finger, RING/FYVE/PHD-type
TraesCS1A02G444900	H2	393	120	161	CAVCLARFDDADLLRLLPRCRHAFHLDCV DRWLHSSASCPLC	Plasma Membrane	
TraesCS1B02G000900	H2	410	234	274	CPICLEEFKVGNEVRGLPCAHNFHVECIDQ WLRLNVKCPRC	Plasma Membrane	
TraesCS1B02G007900	HC	612	326	382	CSICKNVMKEPLTTPCGHNFCKACLTAAFG DEGSMRERSRGGRTLRAQKIVKNCPSC	Nuclear	SRA-YDG superfamily

Identifiers	RING type	Protein size (amino acids)	Domain start	Domain end	RING domain	Localization	Description
TraesCS1B02G032600	HC	628	476	520	CVICWTDFSSSRGILPCGHRFCYSCIQGWADCLSSRGKVSTCPLC	Nuclear	BRCT domain superfamily
TraesCS1B02G036900	H2	399	204	244	CAVCKEPFELGADAREMPCAHIYHEDCILPWLQLRNSCPVC	Nuclear	
TraesCS1B02G074500	HC	362	110	149	CGICHELAEDVVVTSCDHVFCKSCLMECSATLGNVSCPSC	Nuclear	P-loop containing nucleoside triphosphate hydrolase
TraesCS1B02G101700	HC	870	816	855	CVMCLSEEMSVVFLPCAHQVVCAKCNDLHDKQGMKECPSC	Nuclear	Zinc finger, RING/FYVE/PHD-type
TraesCS1B02G107100	HC	514	463	501	CVICLDAPVEGACIPCGHMAGCMSCLKDIESKKWGCPIC	Nuclear	Ankyrin repeat-containing domain superfamily
TraesCS1B02G118700	H2	178	83	125	CLICFDDFVPGCYKKIRTMDCSHSFHQRCIFDWLLIDRRCPIC	Nuclear	
TraesCS1B02G118800	H2	159	89	130	CAVCLEGFVAGGKKLRMMPCSHSFHQRCIFDLLLRSRFCPVC	Cytoplasmic	
TraesCS1B02G123100	H2	434	30	75	CSICLDAVVAASAERSTARLQCGHEFHLDCIGSAFNAKGVMQCPNC	Nuclear	
TraesCS1B02G128900	H2	183	127	168	CAVCLGEMEDGELGRLLPACSHAFHVGCIDAWLRLSSTCPVC	Extracellular	
TraesCS1B02G129600	H2	534	485	525	CIICLEEYKHKDSLGKLKCGHDFHAGCIKKWLEVKNACPVC	Nuclear	Zinc finger, RING/FYVE/PHD-type
TraesCS1B02G132800	HC	406	345	382	CAICQEKMHTPILLQCKHIFCEDCASEWLERERTCPLC	Plasma Membrane	

Identifiers	RING type	Protein size (amino acids)	Domain start	Domain end	RING domain	Localization	Description
TraesCS1B02G138300	H2	420	347	387	CCICLARYSNNDDLRELPCTHFFHKECVDKWLKINALCPLC	Plasma Membrane	
TraesCS1B02G144400	v	169	46	101	GCRICQLEDGDLPAESGSGQLVNLGCGCRGEIAAAHRRCAEAWFSVRGNRRCEICG	Extracellular	Zinc finger, RING-CH-type
TraesCS1B02G145400	H2	236	149	190	CAVCLAVMVDGQAARRLPRCMHVFHRSCVDVWLREHSTCPVC	Chloroplast	
TraesCS1B02G152000	HC	472	411	448	CAICQEKMHVPVLLRCKHIFCEDCVSEWFERERTCPLC	Plasma Membrane	
TraesCS1B02G156500	H2	105	35	76	CCVCISRFRDGEDIRSLPCGHAFHRQCVDRWLALCRRTCPLC	Extracellular	
TraesCS1B02G164800	H2	636	8	52	CAICNGDMRRGGGGGSGFTADCSHQFHYRCVSGSLASSRQACPLC	Nuclear	
TraesCS1B02G174400	v	290	127	174	QCRVCQQKSEEPLVDLGCRCRGDLSKAHRTCIDVWFRTRGSNKCEICQ	Plasma Membrane	Zinc finger, RING/FYVE/PHD-type
TraesCS1B02G179000	H2	369	230	270	CAVCKEDFSPGDGAKQMPCKHIYHADCIVPWLELHNSCPIC	Chloroplast	
TraesCS1B02G187600	HC	242	146	183	CGICMEMNSKVVLPNCTHAMCLRCYQDWNSRSQSCPFC	Nuclear	Zinc finger, RING/FYVE/PHD-type
TraesCS1B02G202600	HC	1041	995	1029	CKVCFESATAAVLLPCRHFCLCKPCALACSECPLC	Nuclear	Kinesin motor domain
TraesCS1B02G212700	HC	406	158	198	CNVCFDMADDPVVTKCGHLFCWDCLYQWLHVHSNHRECPVC	Nuclear	
TraesCS1B02G216000	H2	248	161	202	CAICLTEFGEREEVRVMPQCGHGFHVDCVDTWLRSNSSCPSC	Extracellular	

Identifiers	RING type	Protein size (amino acids)	Domain start	Domain end	RING domain	Localization	Description
TraesCS1B02G218700	H2	232	187	228	CSVCLQDLEVGESARRLPGCRHVFHAPCID RWLVRHASCPLC	Chloroplast	Zinc finger, RING/FYVE/PHD-type
TraesCS1B02G219500	G	621	129	175	CPVCMNAWTADGEHRVSCIPCGHVYGRSC LEKWLAQCGKKSATCPQC	Nuclear	
TraesCS1B02G219700	v	237	16	73	QCRICHEEERDDGCVTATATATAMESPCGC SGSLKYAHRGCVQRWCDEKGSTVCEICL	Extracellular	Protein of unknown function DUF3675
TraesCS1B02G220800	H2	206	110	151	CPVCLEAFGDDDGVKVVPACGHVFHAPCI DRWLDVRNSCPVC	Chloroplast	
TraesCS1B02G228600	H2	369	142	183	CAVCQCVFCGDDELRLLPACRHAFHSSCV DPWLRSNPSCPLC	Plasma Membrane	
TraesCS1B02G228800	HC	326	269	313	CRACRLRPATTVLLPCRHLCVCAACDPGAS ASQAGTAAAAACPAC	Nuclear	E3 ubiquitin-protein ligase BOI-like
TraesCS1B02G238900	v	235	35	84	QCRICHEEEDESSAAMESPCACSGSLKYAH RGCVQRWCDEKGSTLCEICL	Nuclear	Protein of unknown function DUF3675
TraesCS1B02G242700	H2	186	118	159	CAVCIVEFRDGDLARLLPRCGHRFHAACV DAWLHLHSTCPLC	Extracellular	
TraesCS1B02G242800	H2	187	119	160	CAVCIVEFRDGDLARLLPRCGHRFHAACV DAWLHLHSTCPLC	Extracellular	
TraesCS1B02G254700	H2	522	465	506	CYICLVEYEEGDCVRILPCNHEFHLTCVDK WLKEIHRVCPLC	Nuclear	
TraesCS1B02G258900	HC	228	173	211	CVICLTEPRDTAVFPCRHLCMCSECAQALR LQSNKCPIC	Extracellular	
TraesCS1B02G266300	H2	290	217	259	CAICREEMAPGRGGVCVLRPCGHRFHWPC ALRWLARRNTCPCC	Plasma Membrane	

Identifiers	RING type	Protein size (amino acids)	Domain start	Domain end	RING domain	Localization	Description
TraesCS1B02G274400	HC	822	16	64	CPVCLSPFDAASVVPRVLPCGHSLCGSCISSLPPASASAAASSLRCPLC	Plasma Membrane	WD4-repeat-containing domain superfamily
TraesCS1B02G284000	H2	224	142	183	CAICLAEFIRGEKVRVLPRCNHGFHAACIDRWLAARPTCPTC	Plasma Membrane	
TraesCS1B02G289200	v	246	126	179	NCRICHLGLESAAAESGAGITLGCSCKGDLSYSHKQCAETWFKIRGNKTCEICS	Nuclear	Zinc finger, RING-CH-type
TraesCS1B02G304900	HC	341	20	60	CNICFEMADEPVVTSCGHLFCWPCLYQWLHIHSTHKECPVC	Nuclear	
TraesCS1B02G310500	H2	195	120	161	CAICLGEFVDGEKVRVLPQCGHGFHVRCVDAWLVSHSSCPTC	Extracellular	
TraesCS1B02G310700	H2	196	123	164	CAICLGEFVDGEKVRVLPRCAHGFHVRCVDAWLVSHGSCPTC	Plasma Membrane	
TraesCS1B02G316300	H2	332	200	240	CSVCLDDLDIGSQAKQLPCEHKFHSPCILPWLELHSSCPVC	Nuclear	
TraesCS1B02G323200	H2	222	168	209	CAVCLVEVEKGDTVKRLPACLHMFHQQCIDPWLHEHSTCPVC	Plasma Membrane	
TraesCS1B02G323300	H2	222	168	209	CSVCLGEVEKGETVKRLPACLHMFHQQCIDPWLHEHSTCPVC	Plasma Membrane	
TraesCS1B02G327300	HC	498	124	163	CPICLGIIRKTRTVMECLHRFCRDCIDKSMRLGNNECPAC	Nuclear	
TraesCS1B02G327600	H2	127	80	122	CTICIADFRPRARVAVLPTCGHVFHRHCLREVVAGQSPRCPLC	Plasma Membrane	
TraesCS1B02G334400	H2	296	244	285	CPICFELLKSDVAVWPGCLLPHVFHGECLVLALQASEMCPLC	Cytoplasmic	
TraesCS1B02G342100	v	698	28	76	QCRICRLPAEAGRPLRHPCACRGSIRFVHDDCQLRWLATRQRPRCELCN	Plasma Membrane	

Identifiers	RING type	Protein size (amino acids)	Domain start	Domain end	RING domain	Localization	Description
TraesCS1B02G343100	HC	996	16	53	CPICLSLLRSAVSVTCNHIFCNHCLTESMKSSSTCPVC	Nuclear	BRCT domain superfamily
TraesCS1B02G354100	HC	376	325	363	CVVCCDARRDCFFMPCGHSATCHACGTKIVEGDASCPFC	Plasma Membrane	Domain of unknown function DUF4793
TraesCS1B02G361000	H2	203	128	169	CSICLCEYRDGEMQRIMPECRHRFHLMCLDAWLRRSASCPVC	Extracellular	
TraesCS1B02G393400	H2	294	247	287	CSICQEEFEASEEIGKLSCGHGYHVHCIKQWLSRKNACPLC	Nuclear	
TraesCS1B02G395000	H2	1241	1124	1165	CPICCDFLFTSSAAVRGLPCGHFMHSACFQAYTCSHYTCPIC	Nuclear	
TraesCS1B02G398900	v	258	58	105	ECRICQEDGDESCMEAPCSCKGSLKYAHRTCVQRWCDEKGDTICEICL	Plasma Membrane	Protein of unknown function DUF3675
TraesCS1B02G456800	H2	603	20	61	CAICLGAMLRGQAGFTAECSHAFHLNCISASVAHGNHDCPLC	Chloroplast	
TraesCS1B02G465300	v	787	30	79	ECRICRLPAEADRPLHRPCGCRGSMRFVHDDCQLQWIARRRKLPRCEVCR	Plasma Membrane	Zinc finger, RING/FYVE/PHD-type
TraesCS1B02G465500	v	663	32	80	LCRICRLPAEAERPLRSPCACRGSIRFVHDECQLRWIAARGKPLCEVCN	Plasma Membrane	Zinc finger, RING/FYVE/PHD-type
TraesCS1B02G465700	v	809	24	72	QCRICRHPADADRPLRRPCGCKGSMRFVHDHCQIRWLKARRQSLCEVCG	Plasma Membrane	Zinc finger, RING/FYVE/PHD-type
TraesCS1B02G479700	H2	432	128	169	CAVCLARFDDADLLRLLPRCRHAFHLDCVDRWLHSSASCPLC	Plasma Membrane	
TraesCS1D02G002400b	HC	506	144	182	CVFCMKLPERPVTTPCGHNFCLKCFEKWVNSGKRTCGKC	Nuclear	SRA-YDG superfamily

Identifiers	RING type	Protein size (amino acids)	Domain start	Domain end	RING domain	Localization	Description
TraesCS1D02G004000	H2	378	234	274	CPICLEEFKVGNEVRGLPCAHNFHVECIDQ WLRLNVKCPRC	Plasma Membrane	
TraesCS1D02G010300	H2	174	110	151	CAVCLERYADSDVVRVLPDCGHLFHRGCV DPWLRQRPTCPVC	Plasma Membrane	
TraesCS1D02G026100	HC	627	476	520	CVICWTDFSSSRGILPCGHRFCYSCIQGWA DCLSSRGKVSTCPLC	Nuclear	BRCT domain superfamily
TraesCS1D02G030300	H2	402	207	247	CAVCKEPFELGAEAREMPCAHIYHEDCILP WLQLRNSCPVC	Nuclear	
TraesCS1D02G085400	HC	870	816	855	CVMCLSEEMSVVFLPCAHQVVCAKCNDLH DKQGMKECPSC	Nuclear	Zinc finger, RING/FYVE/PHD-type
TraesCS1D02G089400	HC	552	501	539	CVICLDAPVEGACIPCGHMAGCMSCLKDIE SKKWGCPIC	Nuclear	Ankyrin repeat-containing domain superfamily
TraesCS1D02G099500	H2	148	89	130	CAVCLEGFVAGGKKLRTMPCSHSFHQRCIF DLLLRSRFCPVC	Cytoplasmic	
TraesCS1D02G101400	H2	464	410	452	CPVCWQLLEGDDLAAWPGCGKPHVFHGA CLEQVLKMSETCPLC	Nuclear	
TraesCS1D02G103800	H2	434	30	75	CSICLDAVVAASAERSMARLQCGHEFHLD CIGSAFNAKGVMQCPNC	Nuclear	
TraesCS1D02G111500	H2	536	487	527	CIICLEEYKHKDSLGKLKCGHDFHAGCIKK WLEVKNACPVC	Nuclear	Zinc finger, RING/FYVE/PHD-type
TraesCS1D02G112400	H2	190	128	169	CAVCLGEMEDGELGRMLPACSHAFHVGCI DAWLRLSSTCPVC	Plasma Membrane	
TraesCS1D02G114200	HC	411	350	387	CAICQEKMHTPILLQCKHIFCEDCASEWLE RERTCPLC	Plasma Membrane	

Identifiers	RING type	Protein size (amino acids)	Domain start	Domain end	RING domain	Localization	Description
TraesCS1D02G119700	H2	420	347	387	CCICLARYSNNDDLRELPCTHFFHKECVDKWLKINALCPLC	Plasma Membrane	
TraesCS1D02G127300	H2	239	152	193	CAVCLAVMVDGEAARRLPRCMHVFHRSCVDVWLREHSTCPVC	Chloroplast	
TraesCS1D02G128900	v	169	46	101	GCRICQLEDGDLPAESGSGQLVNLGCGCRGEIAAAHRRCAEAWFSVRGNRRCEICG	Extracellular	Zinc finger, RING-CH-type
TraesCS1D02G133300	HC	453	392	429	CAICQEKMHVPVLLRCKHIFCEDCVSEWFERERTCPLC	Plasma Membrane	
TraesCS1D02G138900	H2	109	39	80	CCVCISRFRDGEDIRSLPCGHAFHRQCVDRWLALCRRTCPLC	Extracellular	
TraesCS1D02G141000	HC	236	139	176	CGICLEVNAKIVLPDCTHSLCLRCFEEWNAKSKSCPFC	Cytoplasmic	Zinc finger, RING/FYVE/PHD-type
TraesCS1D02G142100	H2	254	135	177	CPVCFEYLFDSTMDISVLHCGHTIHLECLNEMRVHHHFSCPVC	Extracellular	
TraesCS1D02G144900	H2	683	55	99	CAICNGDMRRGGGGGSGFTADCSHQFHYRCVSGSLASSRQACPLC	Nuclear	
TraesCS1D02G155700	v	275	112	159	QCRVCQQKSEEPLVDLGCRCRGDLSKAHRTCIDVWFRTRGSNKCEICQ	Plasma Membrane	Zinc finger, RING/FYVE/PHD-type
TraesCS1D02G159200	H2	370	230	270	CAVCKEDFSPGDGAKQMPCKHIYHADCIVPWLELHNSCPIC	Nuclear	
TraesCS1D02G167300	HC	246	150	187	CGICMEMNSKVVLPNCTHAMCLRCYQDWNSRSQSCPFC	Nuclear	Zinc finger, RING/FYVE/PHD-type
TraesCS1D02G176100	HC	1041	995	1029	CKVCFESATAAVLLPCRHFCLCKPCALACSECPLC	Nuclear	Kinesin motor domain

Identifiers	RING type	Protein size (amino acids)	Domain start	Domain end	RING domain	Localization	Description
TraesCS1D02G201500	HC	400	152	192	CNVCFDMADDPVVTKCGHLFCWDCLYQWLHVHSNHRECPVC	Nuclear	
TraesCS1D02G205300	H2	248	161	202	CAICLTEFGEREEVRVMPQCGHGFHVECVDTWLRSNSSCPSC	Nuclear	
TraesCS1D02G208400	H2	232	187	228	CSVCLQDLEVGESARRLPGCRHVFHAPCIDRWLVRHASCPLC	Chloroplast	Zinc finger, RING/FYVE/PHD-type
TraesCS1D02G209200	G	554	77	123	CPVCMNAWTADGEHRVSCIPCGHVYGRSCLEKWLAQCGKKSATCPQC	Nuclear	
TraesCS1D02G209400	v	230	16	67	QCRICHEEEGDDGCATAMESPCGCSGSLKYAHRGCVQRWCDEKGSTVCEICL	Extracellular	Protein of unknown function DUF3675
TraesCS1D02G210300	H2	206	110	151	CPVCLEAFGDDDGVKVVPACGHVFHAPCIDRWLDVRNSCPVC	Chloroplast	
TraesCS1D02G217700	H2	376	149	190	CAVCQCVFCADDELRLLPACRHAFHSGCVDPWLRANPSCPLC	Nuclear	
TraesCS1D02G217800	HC	321	267	308	CRGCRLRPATTVLLPCRHLCVCDACDPGASASLAAAAACPTC	Nuclear	E3 ubiquitin-protein ligase BOI-like
TraesCS1D02G226900	v	235	34	83	QCRICHEEEDESCAAMESPCACSGSLKYAHRGCVQRWCDEKGSTLCEICL	Nuclear	Protein of unknown function DUF3675
TraesCS1D02G230300	H2	187	119	160	CAVCIVEFRDGDLARLLPRCGHRFHAACVDAWLHLHSTCPLC	Chloroplast	
TraesCS1D02G230600	H2	186	118	159	CAVCIVEFRDGDLARLLPRCGHRFHAACVDAWLHLHSTCPLC	Chloroplast	
TraesCS1D02G243200	H2	521	464	505	CYICLVEYEEGDCVRILPCNHEFHLTCVDKWLKEIHRVCPLC	Nuclear	

Identifiers	RING type	Protein size (amino acids)	Domain start	Domain end	RING domain	Localization	Description
TraesCS1D02G254900	H2	289	216	258	CAICREEMAPGRGGVCVLRPCGHRFHWPC ALRWLARRNTCPCC	Plasma Membrane	
TraesCS1D02G263900	HC	825	16	64	CPVCLSPFDAASVVPRVLPCGHSLCGSCISS LPPASASAAASSLRCPLC	Plasma Membrane	WD4-repeat-containing domain superfamily
TraesCS1D02G274500	H2	201	119	160	CAICLAEFVRGEQVRVLPRCNHGFHALCID RWLAARPTCPTC	Plasma Membrane	
TraesCS1D02G279400	v	246	126	179	NCRICHLGLESAAAESGAGITLGCSCKGDL SYSHKQCAETWFKIRGNKTCEICS	Nuclear	Zinc finger, RING-CH-type
TraesCS1D02G296100	HC	397	76	116	CNICFEMADDPVVTSCGHLFCWPCLYQWL HIHSTHKECPVC	Nuclear	
TraesCS1D02G296300	H2	195	129	170	CSICISALVAGDKVKALPPCGHCFHPECVD AWLRSQPSCPLC	Extracellular	
TraesCS1D02G296400	H2	192	127	168	CSICISALVPGDKVKALPPCGHCFHPECVDA WLRSQPSCPLC	Extracellular	
TraesCS1D02G296800	H2	215	161	202	CAVCLGEMENGDMVKRLPACLHMFHQHC IDVWLRGHSTCPVC	Plasma Membrane	
TraesCS1D02G299800	H2	183	120	161	CAICLGEFVDGEKVRVLPQCGHGFHVRCV DAWLVSHSACPTC	Plasma Membrane	
TraesCS1D02G299900	H2	191	121	162	CAICLGEFVDGEKVRVLPECCHGFHVRCVD AWLVSHSSCPTC	Plasma Membrane	
TraesCS1D02G300000	H2	198	123	164	CAICLGEFVDGEKVRVLPRCAHGFHVRCV DAWLVSHGSCPTC	Plasma Membrane	
TraesCS1D02G305300	H2	332	200	240	CSVCLDDLDIGSQAKQLPCEHKFHSPCILP WLELHSSCPVC	Nuclear	
TraesCS1D02G311600	H2	215	161	202	CAVCLGEVEKGDTMKRLPACLHMFHQQCI DPWLHEHSTCPVC	Plasma Membrane	

Identifiers	RING type	Protein size (amino acids)	Domain start	Domain end	RING domain	Localization	Description
TraesCS1D02G311700	H2	220	166	207	CAVCLGEVEKGDAVKRLPACLHLFHQQCINAWLHEHSTCPVC	Plasma Membrane	
TraesCS1D02G311900	H2	226	172	213	CPVCLGEVEKGETVKRLPACLHMFHQQCIDPWLQEHSTCPVC	Plasma Membrane	
TraesCS1D02G315600	HC	499	125	164	CPICLGIIRKTRTVMECLHRFCRDCIDKSMRLGNNECPAC	Nuclear	
TraesCS1D02G322200	H2	300	248	289	CPICFELLKSDVAVWPGCLRPHVFHGACLVQALKRSETCPLC	Cytoplasmic	
TraesCS1D02G330800	v	706	30	78	QCRICRLPAEADRPLRHPCACRGSIRFVHDDCQLRWLATRRGPRCELCN	Plasma Membrane	
TraesCS1D02G331700	HC	962	16	53	CPICLSLLRSAVSVTCNHIFCNHCLTESMKSSSTCPVC	Nuclear	BRCT domain superfamily
TraesCS1D02G343700	HC	360	308	347	CVVCCDARRDCFFMPCGHSATCHACGTKIVEGDASCCPFC	Plasma Membrane	Domain of unknown function DUF4793
TraesCS1D02G380000	H2	265	218	258	CSICQEEFEASEEVGRLSCGHGYHVHCIKQWLSRKNACPLC	Nuclear	
TraesCS1D02G382000	H2	1242	1125	1166	CPICCDFLFTSSAAVRGLPCGHFMHSACFQAYTCSHYTCPIC	Nuclear	
TraesCS1D02G405800	H2	498	450	490	CIICQEEFEARDLVGTLDCGHKYHEACIKQWLMVKNLCPIC	Nuclear	
TraesCS1D02G430400	H2	152	93	134	CAICLEALVGGAECSEVPACRHVFHRGCLALWIGSSNTCPLC	Extracellular	
TraesCS1D02G430500	H2	153	94	135	CAICLEALVGGAECSEVPACRHVFHRGCLALWIKSKSTCPLC	Extracellular	
TraesCS1D02G433500	H2	589	11	52	CAICLGGMVRGQAGFTAECSHAFHLRCISASVAHGNHDCPLC	Chloroplast	

Identifiers	RING type	Protein size (amino acids)	Domain start	Domain end	RING domain	Localization	Description
TraesCS1D02G439900	v	756	30	78	LCRICRLPAEAERPLRSPCACRGSIRFVHDE CQLRWISARGKPLCEVCN	Plasma Membrane	Zinc finger, RING/FYVE/PHD-type
TraesCS1D02G453000	H2	434	130	171	CAVCLARFDDADLLRLLPRCRHAFHLDCV DRWLHSSASCPLC	Chloroplast	
TraesCS2A02G022100	H2	701	654	694	CCICQEDYADGEDMGRVECGHYFHTACIK QWLVIKNTCPIC	Nuclear	
TraesCS2A02G039300	v	505	252	301	VCRICMVELSEGGGAMKLECSCRGELALA HTDCALKWFGIKGTRTCEVCK	Plasma Membrane	Zinc finger, RING-CH-type
TraesCS2A02G065200	H2	111	32	73	CCVCLSRIRAGEATRRLPCEHAFHRDCVDR WLALCKRTCPLC	Extracellular	
TraesCS2A02G072700	HC	1165	929	969	CPICLESTSDDPVITPCAHRMCRECLLSSWS TPAGGPCPIC	Nuclear	SNF2-related, N-terminal domain
TraesCS2A02G081500	H2	192	144	187	CPICLQEFEAGGERAGRRLPACSHVFHLECI RSWLLRKPDCPMC	Plasma Membrane	
TraesCS2A02G094000	HC	1235	815	856	CGICLSEEQRATIQGVLNCCSHYFCFACILE WSKVESRCPLC	Nuclear	
TraesCS2A02G099300	H2	162	80	121	CAVCLSGVEEGDEVRELRCRHVFHRACLD RWLATPPATCPLC	Extracellular	
TraesCS2A02G109000	H2	163	114	155	CAVCLSEFGEREAGRLLPGCGHGFHEACIA AWLRLSSTCPLC	Plasma Membrane	
TraesCS2A02G112800	HC	334	230	278	CSICLDPMFDPVSLSCGHIFCYLCCCSAASV TIVDGLKSADHRSKCPLC	Nuclear	
TraesCS2A02G126800	HC	230	134	171	CGICMEINNKVVLPTCSHAMCIKCYRDWRS RSQSCPFC	Nuclear	Zinc finger, RING/FYVE/PHD-type

Identifiers	RING type	Protein size (amino acids)	Domain start	Domain end	RING domain	Localization	Description
TraesCS2A02G145200	HC	824	575	613	CPICLSPPSTTVITSCTHIYCQTCILKILKSAS SRCPIC	Nuclear	SNF2-related, N-terminal domain
TraesCS2A02G156000	HC	233	75	119	CGVCMEPLEWVAIGPCGHREVCSDCAVRM RAGSKSQHASRRCCIC	Extracellular	
TraesCS2A02G156600	H2	176	120	161	CAVCLSLVKEGEVVRQLPACMHLFHVGCV DTWLHSHSTCPLC	Plasma Membrane	
TraesCS2A02G156800	H2	166	68	105	CAICLDAFGAGKEMPCGHRFHGECLERWL GVHGSCPVC	Cytoplasmic	Zinc finger, RING/FYVE/PHD-type
TraesCS2A02G167300	H2	268	101	142	CAVCITELAAGETARVLPRCGHGFHVDCV DMWLKSHSTCPLC	Plasma Membrane	
TraesCS2A02G168700	H2	190	140	180	CPICLEEYDYENPKILLQCNHDFHLGCIYA WMERSQSCPVC	Nuclear	
TraesCS2A02G224300	HC	859	606	645	CGICHELAEDVVVTSCDHVFCKTCLMEYS ATLGNVSCPSC	Nuclear	P-loop containing nucleoside triphosphate hydrolase
TraesCS2A02G227400	H2	529	300	341	CAICRGPMARAKKLPCDHLFHLPCLRSWL DQGLMEGYSCPTC	Plasma Membrane	
TraesCS2A02G229600	HC	500	448	486	CCICHEMQVDSLLYRCGHMCTCFNCADQL KSSSESCPIC	Nuclear	
TraesCS2A02G232200	H2	564	514	557	CVICMTTIDLSQRTSEYMVAPCEHIFHSGCL QRWMDIKMECPTC	Plasma Membrane	
TraesCS2A02G248400	H2	360	30	70	CSICLEAFCDSDPSTVTSCKHDFHLQCILEW CQRSSQCPMC	Nuclear	
TraesCS2A02G290100	HC	323	276	310	CRACGEGESCVLLLPCRHLCLCSACDAAV DTCPLC	Cytoplasmic	Zinc finger, RING/FYVE/PHD-type

Identifiers	RING type	Protein size (amino acids)	Domain start	Domain end	RING domain	Localization	Description
TraesCS2A02G299000	H2	198	114	155	CTICLSDFEAGEHVKVLPKCNHGFHVRCID RWLLARSTCPTC	Nuclear	
TraesCS2A02G311800	H2	276	107	148	CAVCLAELSEGEAARFLPKCGHGFHAECV DLWLHSHPTCPLC	Chloroplast	
TraesCS2A02G318200	H2	380	134	175	CAVCLTAFEEADDLRLLPHCSHAFHPECIDP WLEARITCPLC	Plasma Membrane	
TraesCS2A02G318300	H2	397	193	234	CSVCLGEFNDGELVRLLPKCGHAFHVPCID TWLRAHVNCPLC	Nuclear	
TraesCS2A02G352900	H2	151	49	101	CAVCLSELAGAAGCSGDSEPSAVLVRVLPG CGHGFHDECIGRWLLLRPECPLC	Extracellular	
TraesCS2A02G358400	H2	647	4	47	CAICLGGMGAGGGQAIFTAECSHTFHFHCI SASVEHGNLLCPLC	Mitochondrial	
TraesCS2A02G364100	H2	670	77	120	CAICFDSMRSGHGQALFTAECSHKFHFHCIS SNVKHGNHVCPVC	Nuclear	von Willebrand factor A-like domain superfamily
TraesCS2A02G372600	HC	258	41	91	CNICLDFAADPVVTLCGHLYCWPCIYEWLR PSVVSASGANSTSARQQCPVC	Plasma Membrane	
TraesCS2A02G385400	H2	722	676	716	CTVCLENPSFGDTIRTLPCFHKFHKECIDEW LRRKKLCPVC	Nuclear	
TraesCS2A02G395100	H2	176	112	154	CRVCLERLEPTDEVRPLGNCAHAFHRGCID RWIDVGEATCPLC	Extracellular	
TraesCS2A02G399700	H2	198	91	132	CSICLGEYTEKELLRIIPTCQHNFHRTCLDL WLQKQTTCPIC	Extracellular	Zinc finger, RING/FYVE/PHD-type
TraesCS2A02G400200	H2	253	206	246	CVICCMAYKNRDKLTTLPCQHQYHRTCVA KWLKINKVCPVC	Nuclear	

Identifiers	RING type	Protein size (amino acids)	Domain start	Domain end	RING domain	Localization	Description
TraesCS2A02G401400	H2	374	318	358	CCICISAYDDGAELRELPCGHHFHCACIDKWLHINATCPLC	Plasma Membrane	
TraesCS2A02G437200	H2	369	321	361	CSICQEEYSGGEEVGNMACKHYYHITCIQHWLRQKNWCPIC	Nuclear	
TraesCS2A02G446100a	HC	553	236	327	CMICFSEYKGIDFIKLPCRHYFCRNCMETYSRMHVKEGSVMKIVCPDNKCGGFVPPNLLKRLLGESDFERWERLILERTLDAMADVAYCPRC	Nuclear	
TraesCS2A02G446100b	HC	553	407	436	CPHCGTGITRVSGCNHMLCKNCGKLFCYGC	Nuclear	
TraesCS2A02G448700	H2	397	163	204	CSVCLGEFHDGESLRLLPKCSHAFHQQCIDTWLKSHSNCPLC	Nuclear	
TraesCS2A02G453200	H2	340	102	143	CAVCLSPFRPEAELRLLPACRHAFHAVCVDAWLRTTPTCPLC	Nuclear	
TraesCS2A02G455400	H2	190	107	148	CAICLAEFEDGEAMRVLPQCGHAFHAACVDKWLRGHSSCPSC	Extracellular	
TraesCS2A02G475800	H2	174	105	146	CAVCLGAMQDGDAVRALPGCSHAFHAGCVDVWLGAHATCPVC	Extracellular	
TraesCS2A02G501100	H2	197	111	154	CAVCLGSFDAAADELLRVLPKCRHAFHAGCVDTWLEAHSTCPVC	Plasma Membrane	
TraesCS2A02G511100	H2	237	182	222	CAICLDGLEGEPAVQLPGCPHAFHRRCISTWFSKKTTCPMC	Cytoplasmic	
TraesCS2A02G514400	H2	641	594	634	CCICQEEYVDGDDLGTLHCGHDFHASCIRQWLVVKNLCPIC	Nuclear	
TraesCS2A02G526800a	HC	526	156	205	CPMCMETAPIILKSRFGSCDDHVFCSSCVAKYVAVKQRQDVALPVECPDC	Nuclear	Zinc finger, RING/FYVE/PHD-type

Identifiers	RING type	Protein size (amino acids)	Domain start	Domain end	RING domain	Localization	Description
TraesCS2A02G545900a	HC	681	42	139	CAKCMSVVPCACIRPCFSMCDHDFCSSCVAMKLPQNVARLKCTGESSTASEKYRGVTILRPETVQVPSRIDPMFITDGNEGSPVNDISDSKRFYCAKC	Extracellular	Zinc finger, RING/FYVE/PHD-type
TraesCS2A02G545900c	HC	681	324	423	CAKCMHVVPCACIRSNFTTCDHDFCSRCVGLKLCQNVPRVKCTVDNSTVAERYHSMAMVQPETAVSSPIDPKFVTGGNEDSPLVDGNDIGDDKHFYCAKC	Extracellular	Zinc finger, RING/FYVE/PHD-type
TraesCS2A02G546000a	HC	644	229	278	CPMCITTAPIVHKSSFGSCDHHAFCSNCVAKYVAMRQREGVALPVECPDC	Extracellular	
TraesCS2A02G546000b	HC	644	321	368	CAKCMHVVPCACVRRSFTSCAHDFCSRCVSRRLQGRLGDGKHFYCTKC	Extracellular	
TraesCS2A02G556200	HC	4749	4703	4736	CRVCLNNEVNMTIVPCGHVLCNRCSSSVSRCPFC	Plasma Membrane	Zinc finger, RING/FYVE/PHD-type
TraesCS2A02G564000	H2	529	240	281	CAICLEDYTVGEQIRVLPCRHKFHAACVDMWLTSWRSFCPVC	Plasma Membrane	
TraesCS2A02G586800	HC	259	156	193	CGICMELNSRVVLPNCSHDMCIKCYRQWRSRSQSCPFC	Extracellular	Zinc finger, RING/FYVE/PHD-type
TraesCS2B02G019500	H2	222	174	215	CTICLQDMVAGETARRLPNCSHAFHQPCVDKWLFTHGSCPVC	Extracellular	
TraesCS2B02G030700	H2	701	654	694	CCICQEDYADGEDMGRVECGHYFHTACIKQWLVIKNTCPIC	Nuclear	

Identifiers	RING type	Protein size (amino acids)	Domain start	Domain end	RING domain	Localization	Description
TraesCS2B02G051700	v	494	243	292	VCRICMVELSEGGGAMKLECSCRGELALA HTDCALKWFGIKGTRTCEVCK	Plasma Membrane	Zinc finger, RING-CH-type
TraesCS2B02G077400	H2	135	54	95	CCVCLSRIRAGEATRRLPCEHAFHRDCVDR WLALCKRTCPLC	Nuclear	
TraesCS2B02G086700	HC	1181	945	985	CPICLESTSDDPVITPCAHRMCRECLLSCWS TPAGGACPIC	Nuclear	SNF2-related, N-terminal domain
TraesCS2B02G108800	HC	1238	818	859	CGICLSEEQRATIQGVLNCCSHYFCFACILE WSKVESRCPLC	Nuclear	
TraesCS2B02G115500	H2	165	80	121	CAVCLSGVGEGDEVRELRCRHVFHRACLD RWLATPPATCPLC	Extracellular	
TraesCS2B02G126700	H2	159	110	151	CAVCLSEFAEREAGRRLPGCGHGFHEACIA AWLRLNSTCPLC	Plasma Membrane	
TraesCS2B02G131600	HC	334	230	278	CSICLDTMFDPVSLSCGHIFCYLCCCSAASV TIVDGLKSADHRSKCPLC	Nuclear	
TraesCS2B02G144500a	HC	394	114	163	CPMCMELVPINLKSSFGPCGHAFCSSCVTM YVAVKQREREDVGRVKCPDC	Extracellular	Zinc finger, RING/FYVE/PHD-type
TraesCS2B02G148700	HC	234	138	175	CGICMEINNKVVLPTCSHAMCIKCYRDWRS RSQSCPFC	Extracellular	Zinc finger, RING/FYVE/PHD-type
TraesCS2B02G162000	HC	341	294	328	CVICLEQEYNAVFVPCGHMCCCMNCSSHV TNCPLC	Extracellular	E3 Ubiquitin ligase, GIDE-type
TraesCS2B02G170200	HC	824	575	613	CPICLSPPSTTVITSCTHIYCQTCILKILKSPSS RCPIC	Nuclear	SNF2-related, N-terminal domain
TraesCS2B02G181500	HC	236	78	122	CGVCMEPLEWVALGPCGHREVCSDCAVR MRVGSKSQHASRRCCIC	Extracellular	

Identifiers	RING type	Protein size (amino acids)	Domain start	Domain end	RING domain	Localization	Description
TraesCS2B02G182100	H2	176	120	161	CAVCLSLVKEGEVVRQLPACMHLFHVGCV DTWLHSHSTCPLC	Plasma Membrane	
TraesCS2B02G182300	H2	187	90	127	CAICLDAFGAGKEMPCGHRFHGECLERWL GVHGSCPVC	Cytoplasmic	
TraesCS2B02G193500	H2	267	101	142	CAVCITELAAGETARVLPRCGHGFHVDCV DMWLKSHSTCPLC	Plasma Membrane	
TraesCS2B02G195000	H2	190	140	180	CPICLEEYDYENPKVLLQCNHDFHLGCIYE WMERSQSCPVC	Extracellular	
TraesCS2B02G250800	H2	565	515	558	CVICMTAIDLSQRTSEYMVAPCEHIFHSGCL QRWMDIKMECPTC	Plasma Membrane	Zinc finger, RING/FYVE/PHD-type
TraesCS2B02G251300	HC	734	682	720	CCICHEMQVDSLLYRCGHMCTCFNCADQL KSSSGSCPIC	Nuclear	
TraesCS2B02G255300	H2	558	329	370	CAICRGPMARAKKLPCNHLFHLPCLRSWL DQGLMEGYSCPTC	Plasma Membrane	
TraesCS2B02G274500	HC	846	593	632	CGICHELAEDVVVTSCDHVFCKTCLMEYS ATLGNVSCPSC	Nuclear	P-loop containing nucleoside triphosphate hydrolase
TraesCS2B02G306600	HC	323	276	310	CRACGEGESCVLLLPCRHLCLCSACDAAV DTCPLC	Nuclear	Zinc finger, RING/FYVE/PHD-type
TraesCS2B02G315400	H2	209	125	166	CTICLSDFEAGEHVKVLPKCNHGFHVRCID RWLLARSTCPTC	Nuclear	
TraesCS2B02G328600	H2	281	107	148	CAVCLAELSDGEAARFLPKCGHGFHAECV DLWLHSHPTCPLC	Plasma Membrane	
TraesCS2B02G336300	H2	380	134	175	CAVCLTAFEEADDLRLLPHCSHAFHPECIDP WLEARITCPLC	Plasma Membrane	

Identifiers	RING type	Protein size (amino acids)	Domain start	Domain end	RING domain	Localization	Description
TraesCS2B02G336400	H2	398	195	236	CSVCLGEFSDGELVRLLPKCGHAFHVPCID TWLRAHVNCPLC	Nuclear	
TraesCS2B02G370700	H2	220	114	166	CAVCLSELVGAAEFSGDSEPSAVPVRVLPG CGHWFHDECIGRWLLLRPECPLC	Plasma Membrane	
TraesCS2B02G377900	H2	646	10	53	CAICLGGMGAGGEQAIFTAECSHTFHFHCIS ASVEHGNLLCPLC	Chloroplast	von Willebrand factor A-like domain superfamily
TraesCS2B02G382300	H2	695	76	119	CAICFDSMRSGHGQALFTAECSHKFHFHCIS SNVKHGNHVCPVC	Nuclear	von Willebrand factor A-like domain superfamily
TraesCS2B02G389600	HC	260	43	93	CNICLDFAADPVVTLCGHLYCWPCIYEWLR PSVVSASGANSTSARQQCPVC	Plasma Membrane	
TraesCS2B02G402300	H2	722	676	716	CAVCLENPSFGDTIRTLPCFHKFHKECIDEW LRRKKLCPVC	Nuclear	
TraesCS2B02G413100	H2	180	112	156	CAICLAALDVGGQLVTELCNCSHAFHGACI GYWIGSGEAGTCPLC	Extracellular	
TraesCS2B02G413200	H2	173	110	152	CRVCLERLELTDEVRPLGNCSHAFHRGCID RWIDIGEVTCPLC	Extracellular	
TraesCS2B02G413800	H2	201	103	145	CAVCLSGIGGGDEVRRLSNCRHAFHRGCL DRWMEHDQRTCPLC	Extracellular	
TraesCS2B02G417700	H2	199	92	133	CSICLGEYTEKELLRIIPTCRHNFHRTCLDL WLQKQTTCPIC	Extracellular	Zinc finger, RING/FYVE/PHD-type
TraesCS2B02G418300	H2	253	206	246	CVICCMAYKNRDKLTTLPCQHQYHRTCVA KWLKINKVCPVC	Nuclear	

Identifiers	RING type	Protein size (amino acids)	Domain start	Domain end	RING domain	Localization	Description
TraesCS2B02G419400	H2	374	318	358	CCICISAYDDGAELRELPCGHHFHCACIDK WLHINATCPLC	Plasma Membrane	
TraesCS2B02G456100	H2	373	325	365	CSICQEEYSGGEEVGNMACKHYYHIACIQH WLRQKNWCPIC	Nuclear	
TraesCS2B02G467200b	HC	553	407	436	CPHCGTGITRVSGCNHMLCKNCGKLFCYG C	Nuclear	
TraesCS2B02G469500	H2	397	163	204	CSVCLGEFDDGESLRLLPKCSHAFHQQCID TWLKSHSNCPLC	Nuclear	
TraesCS2B02G474500	H2	344	106	147	CAVCLSPFRPEAELRLLPACRHAFHAVCVD AWLRTTPTCPLC	Nuclear	
TraesCS2B02G477500	H2	184	103	144	CAICLAEFEDGEAMRVLPQCGHAFHAACV DKWLRGHSSCPSC	Extracellular	
TraesCS2B02G499500	H2	178	106	147	CAVCLGAMQDGDAVRALPGCSHAFHAGC VDVWLGAHATCPVC	Cytoplasmic	
TraesCS2B02G529100	H2	197	111	154	CAVCLGAFDAAADELLRVLPKCRHAFHAD CVDTWLEAHSTCPVC	Plasma Membrane	
TraesCS2B02G542200	H2	669	622	662	CCICQEEYVDGDDLGTLHCGHDFHASCIRQ WLVVKNLCPIC	Nuclear	
TraesCS2B02G566600	H2	164	105	145	CAVCLKDFEVDDKVATMPCDHYFHQGCIS EWLKVSCACPLC	Cytoplasmic	
TraesCS2B02G566700	H2	168	109	149	CAVCLKDFEVDDKVTTMPCDHYFHQGCIS EWLKVSCVCPLC	Nuclear	
TraesCS2B02G576800a	HC	684	42	143	CAKCMSVVACACIRPGFSMCDHEFCSSCV AMKLPQNVARVKCTGESSTGSEKYHGLTIL RPETVQLPSRIDPMFITEGNEGSLSEGKEGS LSDSKRFYCAKC	Extracellular	Zinc finger, RING/FYVE/PHD-type

Identifiers	RING type	Protein size (amino acids)	Domain start	Domain end	RING domain	Localization	Description
TraesCS2B02G576800b	HC	684	234	275	CAKCMHVVPCACIRSNFTMCDHDFCSRCV TMKLRQNIDCVKC	Extracellular	Zinc finger, RING/FYVE/PHD-type
TraesCS2B02G576800c	HC	684	328	426	CAKCMHVVPCACIRSNFTTCDHDFCSRCV GLKLCQNVPRVKCTVDNSTAAERYHSMA MVRPETVSSRIGPKFVTGGNEDSPLVDSKDI GDGKHFYCAEC	Extracellular	Zinc finger, RING/FYVE/PHD-type
TraesCS2B02G577000a	HC	601	274	322	CAKCMHVVPCACIRRSFTSCAHDFCSSCVS RRLQARRGDGGKHFYCTKC	Extracellular	
TraesCS2B02G586500	HC	4743	4697	4730	CRVCLNSEVNMTIVPCGHVLCNRCSSSVSR CPFC	Plasma Membrane	Zinc finger, RING/FYVE/PHD-type
TraesCS2B02G596600	HC	259	156	193	CGICMELNSRVVLPNCSHDMCIKCYHQWR TRSQSCPFC	Extracellular	Zinc finger, RING/FYVE/PHD-type
TraesCS2B02G625300	H2	527	240	281	CAICLEDYTVGEQIRVLPCRHKFHAACVDM WLTSWRSFCPVC	Plasma Membrane	
TraesCS2D02G005100	H2	224	176	217	CTICLQDMVAGETARRLPNCSHAFHQPCV DKWLFTHGSCPVC	Extracellular	
TraesCS2D02G022900	H2	701	654	694	CCICQEDYADGEDMGRVECGHYFHTACIK QWLVIKNTCPIC	Nuclear	
TraesCS2D02G037600	v	495	242	291	VCRICMVELSEGGGAMKLECSCRGELALA HTDCALKWFGIKGTRTCEVCK	Plasma Membrane	Zinc finger, RING-CH-type
TraesCS2D02G063300	H2	111	33	74	CCVCLSRIRAGEATRRLPCEHAFHRDCVDR WLALCKRTCPLC	Extracellular	

Identifiers	RING type	Protein size (amino acids)	Domain start	Domain end	RING domain	Localization	Description
TraesCS2D02G071800	HC	1170	934	974	CPICLESTSDDPVITPCAHRMCRECLLSCWS TPAGGACPIC	Nuclear	SNF2-related, N-terminal domain
TraesCS2D02G079500	H2	192	140	183	CPICLQEFDAGGEREGRRLPACSHVFHLECI RSWLLRKPHCPMC	Plasma Membrane	
TraesCS2D02G092600	HC	867	447	488	CGICLSEEQRATIQGVLNCCSHYFCFACILE WSKVESRCPLC	Nuclear	
TraesCS2D02G098400	H2	233	151	192	CAVCLSGVAEGDEVRELRCRHVFHRACLD RWLATPPATCPLC	Extracellular	
TraesCS2D02G109100	H2	162	113	154	CAVCLSEFGEREAGRRLPGCGHGFHEACIA AWLRLSSTCPLC	Plasma Membrane	
TraesCS2D02G125100a	HC	400	119	168	CPMCMELVPINLKSSLGPCGHAFCSSCVTT YVAVKQREREDAARVKCPDC	Extracellular	Zinc finger, RING/FYVE/PHD-type
TraesCS2D02G129300	HC	242	146	183	CGICMEINNKVVLPTCSHAMCIKCYRDWRS RSQSCPFC	Nuclear	Zinc finger, RING/FYVE/PHD-type
TraesCS2D02G141100	HC	341	294	328	CVICLEQEYNAVFVPCGHMCCCMNCSSHV TNCPLC	Extracellular	E3 Ubiquitin ligase, GIDE-type
TraesCS2D02G148900	HC	824	575	613	CPICLSPPSTTVITSCTHIYCQPCILKILKSAS SRCPIC	Nuclear	SNF2-related, N-terminal domain
TraesCS2D02G161800	HC	233	75	119	CGVCMEPLEWVAIGPCGHREVCSDCAVRM RVGSKPQHASRRCCIC	Extracellular	
TraesCS2D02G162400	H2	176	120	161	CAVCLSLVKEGEVVRQLPACMHLFHVGCV DTWLHSHSTCPLC	Plasma Membrane	
TraesCS2D02G162600	H2	187	88	125	CAICLDAFGAGKEMPCGHRFHGECLERWL GVHGSCPVC	Cytoplasmic	
TraesCS2D02G174800	H2	267	101	142	CAVCITELAAGETARVLPRCGHGFHVDCV DMWLKSHSTCPLC	Plasma Membrane	

Identifiers	RING type	Protein size (amino acids)	Domain start	Domain end	RING domain	Localization	Description
TraesCS2D02G176300	H2	174	124	164	CPICLEEYDYENPKILLQCNHDFHLGCIYA WMERSQSCPVC	Extracellular	
TraesCS2D02G187100a	HC	349	142	186	CAICMETVPGVLKLSVGSCGHAFCSSCVVQ YVAAKLGENVACVKC	Extracellular	
TraesCS2D02G230600	HC	862	609	648	CGICHELAEDVVVTSCDHVFCKTCLMEYS ATLGNVSCPSC	Nuclear	P-loop containing nucleoside triphosphate hydrolase
TraesCS2D02G232900	H2	565	515	558	CVICMTTIDLSQRTSEYMVAPCEHIFHSGCL QRWMDIKMECPTC	Plasma Membrane	Zinc finger, RING/FYVE/PHD-type
TraesCS2D02G233500	HC	757	705	743	CCICHEMQVDSLLYRCGHMCTCFNCADQL KSSSGSCPIC	Nuclear	
TraesCS2D02G235300	H2	543	329	370	CAICRGPMARAKKLPCNHLFHLPCLRSWL DQGLMEGYSCPTC	Plasma Membrane	
TraesCS2D02G250100	H2	360	30	70	CSICLEAFCDSDPSTVTSCKHDFHLQCILEW CQRSSQCPMC	Nuclear	
TraesCS2D02G288000	HC	323	276	310	CRACGEGESCVLLLPCRHLCLCSACDAAV DTCPLC	Nuclear	Zinc finger, RING/FYVE/PHD-type
TraesCS2D02G296900	H2	198	114	155	CTICLSDFEAGEHVKVLPKCNHGFHVRCID RWLLARSTCPTC	Nuclear	
TraesCS2D02G310100	H2	276	107	148	CAVCLAELSDGEAARFLPKCGHGFHAECV DLWLHSHPTCPLC	Chloroplast	
TraesCS2D02G315800	H2	373	134	175	CAVCLTAFEEADDLRLLPHCSHAFHPECIDP WLEARITCPLC	Plasma Membrane	
TraesCS2D02G315900	H2	400	196	237	CSVCLGEFNDGELVRLLPKCGHAFHVPCID TWLRAHVNCPLC	Nuclear	

Identifiers	RING type	Protein size (amino acids)	Domain start	Domain end	RING domain	Localization	Description
TraesCS2D02G351200	H2	149	47	99	CAVCLSELVGAAGYSGDSEPSSALVRVLSG CGHGFHDECIGRWLLLRPECPLC	Extracellular	
TraesCS2D02G357400	H2	646	10	53	CAICLGGMGAGGGQAIFTAECSHTFHFHCI STSVEHGNLLCPLC	Chloroplast	von Willebrand factor A-like domain superfamily
TraesCS2D02G361900	H2	692	73	116	CAICFDSMRSGHGQALFTAECSHKFHFHCIS SNVKHGNHVCPVC	Nuclear	von Willebrand factor A-like domain superfamily
TraesCS2D02G368800	HC	263	46	96	CNICLDFAADPVVTLCGHLYCWPCIYEWLR PLVVSASGANSTSARQQCPVC	Plasma Membrane	
TraesCS2D02G382100	H2	722	676	716	CAVCLENPSFGDTIRTLPCFHKFHKECIDEW LRRKKLCPVC	Nuclear	
TraesCS2D02G392500	H2	175	112	154	CRVCLERLELTDEVRPLGNCSHAFHRGCID RWIDVGEVTCPLC	Extracellular	
TraesCS2D02G392600	H2	180	112	156	CAICLAALDVGGQLVTELCNCSHAFHAACI DSWIGSGEAGTCPMC	Extracellular	
TraesCS2D02G393400	H2	204	106	148	CAVCLSGIGGGDEVRRLSNCRHAFHRGCL DRWMEHDQRTCPLC	Extracellular	
TraesCS2D02G397200	H2	199	92	133	CSICLGEYTEKELLRIIPTCQHNFHRTCLDL WLQKQTTCPIC	Extracellular	Zinc finger, RING/FYVE/PHD-type
TraesCS2D02G397800	H2	257	210	250	CVICCMAYKNRDKLTTLPCQHQYHRTCVA KWLKINKVCPVC	Nuclear	
TraesCS2D02G398700	H2	377	321	361	CCICISAYDDGAELRELPCGHHFHCACIDK WLHINATCPLC	Plasma Membrane	

Identifiers	RING type	Protein size (amino acids)	Domain start	Domain end	RING domain	Localization	Description
TraesCS2D02G432800	H2	372	324	364	CSICQEEYSGGEEVGNMACKHYYHITCIQHWLRQKNWCPIC	Nuclear	
TraesCS2D02G445000b	HC	497	351	380	CPHCGTGITRVSGCNHMLCKNCGKLFCYGC	Nuclear	
TraesCS2D02G447900	H2	398	162	203	CSVCLGEFHDGESLRLLPKCSHAFHQQCIDTWLKSHSNCPLC	Nuclear	
TraesCS2D02G452800	H2	340	102	143	CAVCLSPFRPEAELRLLPACRHAFHAVCVDAWLRTTPTCPLC	Nuclear	
TraesCS2D02G455800	H2	189	106	147	CAICLAEFEDGEAMRVLPQCGHAFHAACVDKWLRGHSSCPSC	Extracellular	
TraesCS2D02G475900	H2	166	104	145	CAVCLGAMQDGDAVRALPGCSHAFHAGCVDVWLGAHATCPVC	Chloroplast	
TraesCS2D02G479100	HC	1042	726	777	CMICAEQRKNYMDSRTGPKEPPENAVITTCCHIFCYECAQESLSEEEVCPVC	Nuclear	SNF2-related, N-terminal domain
TraesCS2D02G501500	H2	197	111	154	CAVCLGAFDAAADELLRVLPKCRHAFHAGCVDTWLEAHSTCPVC	Plasma Membrane	
TraesCS2D02G512500	H2	236	181	221	CAICLDGLEGEPAVELPACPHAFHRRCISTWFSKKTTCPIC	Cytoplasmic	
TraesCS2D02G516000	H2	669	622	662	CCICQEEYVDGDDLGTLHCGHDFHASCIRQWLVVKNLCPIC	Nuclear	
TraesCS2D02G522200	H2	167	106	146	CAVCLKDFKVDDKVTTMPCDHYFHQGCIYEWLMVSCVCPLC	Nuclear	
TraesCS2D02G537500	H2	134	75	115	CAVCLKDFEVDDKVTTMPCDHYFHQGCISEWLKVSCVCPLC	Nuclear	
TraesCS2D02G547100a	HC	496	40	81	CAKCMHVVPCACMRYNFTMCDHDFCSRCVSMKLRQNIDCVKC	Extracellular	Zinc finger, RING/FYVE/PHD-type

Identifiers	RING type	Protein size (amino acids)	Domain start	Domain end	RING domain	Localization	Description
TraesCS2D02G547100b	HC	496	140	238	CAKCMHVVPCACIRPNFTTCDHDFCSRCVGLKLCQNVPRVKCTVDNSTAAERYRSMATVRPETVSSRIDPKFVTGGNEDSPLVDGNDIGDGKHFYCAKC	Extracellular	Zinc finger, RING/FYVE/PHD-type
TraesCS2D02G547300a	HC	609	203	251	CPMCTTTAPIIHKSSFGSCGHAFCNSCVAKYIAVRQRENVALPVECPEC	Extracellular	Zinc finger, RING/FYVE/PHD-type
TraesCS2D02G547300b	HC	609	286	333	CAKCMHVVPCACIRRSFTSCAHDFCSNCVSRRLQGRRGDGKHFYCTKC	Extracellular	Zinc finger, RING/FYVE/PHD-type
TraesCS2D02G557400	HC	4749	4703	4736	CRVCLNNEVNMTIVPCGHVLCNRCSSSVSRCPFC	Plasma Membrane	Zinc finger, RING/FYVE/PHD-type
TraesCS2D02G574200	H2	527	240	281	CAICLEDYTVGEQIRVLPCRHKFHAACVDMWLTSWRSFCPVC	Plasma Membrane	
TraesCS2D02G599700	HC	259	156	193	CGICMELNSRVVLPNCSHDMCINCYRQWRSRSQSCPFC	Extracellular	Zinc finger, RING/FYVE/PHD-type
TraesCS3A02G001800	H2	119	53	108	CAICRNHIMDLCIECQANQASATSEECTVAWGVCNHAFHFHCISRWLKTRQVCPLD	Nuclear	
TraesCS3A02G022400	H2	286	98	139	CAVCLAGLEDGEEARFLPRCGHGFHTGCVDRWLASHTTCPLC	Plasma Membrane	
TraesCS3A02G025800	v	276	55	102	ECRICQEEDWDTGMEAPCACRGSLKYAHRKCIQRWCNEKGDTICEICL	Plasma Membrane	Protein of unknown function DUF3675
TraesCS3A02G051000	H2	331	124	165	CAVCLTEFEDADDLRLLPHCSHAFHPDCIDPWLQTRTTCPLC	Plasma Membrane	

Identifiers	RING type	Protein size (amino acids)	Domain start	Domain end	RING domain	Localization	Description
TraesCS3A02G087900	H2	234	134	175	CAVCLAEFERGEEARALPRCGHRFHVECID AWFRGNSTCPLC	Chloroplast	
TraesCS3A02G095400	HC	415	354	391	CAICQEKMHVPILLRCKHVFCEDCVSEWFE RERTCPLC	Plasma Membrane	
TraesCS3A02G103000	H2	519	474	514	CLVCLEEYKDNDLLGILKCRHDFHTDCIKK WLQVKNACPVC	Nuclear	Zinc finger, RING/FYVE/PHD-type
TraesCS3A02G120100	H2	410	335	375	CCICLSRFSNNEDLRELPCGHVFHMECIDK WLKINALCPLC	Plasma Membrane	
TraesCS3A02G131400	H2	136	90	131	CPVCLGAVEEGETMRALPVCRHAFHARCV DAWLRLRPTCPVC	Plasma Membrane	
TraesCS3A02G131500	H2	178	96	137	CAVCLGQLEAGEKARRLPKCAHLFHAECV DAWLRAHCTCPMC	Plasma Membrane	
TraesCS3A02G139700	H2	211	100	141	CAVCLRVVDEGETVRQLPSCKHLFHQECV DVWLLSNASCPVC	Extracellular	
TraesCS3A02G140000	H2	218	120	161	CAVCLSELADGDAVRQLPNCGHVFHVECV DAWLRTRTTCPLC	Plasma Membrane	
TraesCS3A02G176600	H2	429	282	334	CKLCLKLLKERPAWNAQDLGVVAVLLCG HVYHADCLDSLTAEAEKYDPPCPVC	Nuclear	
TraesCS3A02G179300	H2	400	292	329	CIICREEMTTAKKLLCGHLFHVHCLRSWLE RQHTCPTC	Plasma Membrane	
TraesCS3A02G182100	H2	193	105	146	CVFCLSRIDDGEEVRELRCRHVFHRECLDS WLLRPRATCPLC	Plasma Membrane	
TraesCS3A02G189200	H2	375	228	268	CAVCLEDYASGERARELPCRHRFHSQCIVP WLDMHSSCPVC	Nuclear	
TraesCS3A02G246900	H2	526	475	515	CIICQEEYEAEDCVGILDCGHRYHAVCLKQ WLTVKNLCPIC	Nuclear	

Identifiers	RING type	Protein size (amino acids)	Domain start	Domain end	RING domain	Localization	Description
TraesCS3A02G254400	H2	305	260	300	CCICLAQYREKEEVRQLPCTHMFHLKCVDRWLRIISSCPLC	Plasma Membrane	Zinc finger, RING/FYVE/PHD-type
TraesCS3A02G259800	HC	273	225	262	CCVCMVRHKGAAFIPCGHTFCRLCSRELWVSRGNCPLC	Nuclear	
TraesCS3A02G260500	H2	345	298	338	CSICQEEFEANDEMGRLGCGHSYHVYCIKQWLSQKNVCPVC	Nuclear	
TraesCS3A02G264000	v	261	52	99	ECRICQEEGDQAYMETPCSCKGSLKYAHHICIQRWCNEKGDTICEICL	Plasma Membrane	Protein of unknown function DUF3675
TraesCS3A02G271500	H2	418	373	413	CPICLDCPADGAPLRHLPCAHKFHKECIDRWLRMRTSCPVC	Nuclear	Zinc finger, RING/FYVE/PHD-type
TraesCS3A02G278900	H2	194	119	160	CSICLSEYMDGVMLRLMPECRHRFHVMCLDAWLRRSASCPVC	Plasma Membrane	
TraesCS3A02G281400	v	182	60	117	ACRICHLIPEGGDGPAAAPGSEVIRLGCCCKEELGHAHRQCAEAWFRIKGDRRCEICG	Extracellular	
TraesCS3A02G288900	H2	317	212	254	CPICFEYLFESRNDVSVLPCGHTIHEKCLKEMKEHCQFACPLC	Extracellular	RCHY1, zinc-ribbon
TraesCS3A02G293400	H2	243	116	157	CTICLGAMQEGEAVRVLPACAHVFHVPCVDTWLASSSSCPVC	Nuclear	
TraesCS3A02G293500	H2	243	116	157	CTICLGAMQEGEAVRVLPACAHVFHVPCVDTWLASSSSCPVC	Nuclear	
TraesCS3A02G309200	H2	121	32	73	CPICLGQEEAAATAWKETTCGHNFHGRCVERWLETKGTCPMC	Extracellular	
TraesCS3A02G309400	H2	122	32	72	CPICLDQERVTTAWKETVCGHIFHEQCVERWLETKGSCPMC	Nuclear	

Identifiers	RING type	Protein size (amino acids)	Domain start	Domain end	RING domain	Localization	Description
TraesCS3A02G309500	H2	123	32	72	CPICLDQEGATTAWKETVCGHIFHERCVERWLQAKGSCPMC	Nuclear	
TraesCS3A02G327900	HC	485	110	149	CPICLGIIRKTRTVMECLHRFCRDCIDKSMRLGNNECPAC	Nuclear	
TraesCS3A02G330900	H2	403	264	304	CPVCTERFEIGSEAREMPCKHLYHASCIIPWLVQHNSCPVC	Nuclear	
TraesCS3A02G339000	H2	214	164	204	CPICLEEYDYENPKIVLQCNHNFHLSCIYEWMERSQSCAVC	Extracellular	
TraesCS3A02G344600	H2	205	132	173	CAICLGEFEDGEKVRVLPRCGHEFHVRCVDTWLVSHDSCPTC	Chloroplast	
TraesCS3A02G345400	H2	126	68	110	CAICKGPLAAGGGRCRRLRACGHVYHAECVDLWLQRKPICPLC	Extracellular	
TraesCS3A02G350000	HC	567	230	270	CNICFEMAGEPVVTSCGHLFCWPCLYQWLNVYSNHKECPVC	Nuclear	
TraesCS3A02G350500	H2	181	120	161	CPICLGEFADGERVKALPRCGHCFHPECVDAWLCSHPSCPLC	Plasma Membrane	
TraesCS3A02G365100	v	252	132	185	NCRICHLGLESAAAESGAGIVLGCSCKDDLSCAHKQCAETWFKIRGNKICEICG	Extracellular	Zinc finger, RING-CH-type
TraesCS3A02G377100	H2	208	138	179	CAICLADLKQGERVRVLPRCHHGFHVRCIDRWLSARQTCPTC	Extracellular	
TraesCS3A02G391400	HC	251	155	192	CGICLETCTKMVLPNCNHAMCINCYRDWYTRSQSCPFC	Extracellular	Zinc finger, RING/FYVE/PHD-type
TraesCS3A02G399700	v	205	27	74	ECRICQEEGDEAAMDSPCACTGTLKFAHRKCIQRWCDKKGNITCEICN	Plasma Membrane	Protein of unknown function DUF3675

Identifiers	RING type	Protein size (amino acids)	Domain start	Domain end	RING domain	Localization	Description
TraesCS3A02G410600	H2	245	193	234	CSVCLQDLRPQQFVRALPQCQHIFHVRCIDDWLQRNASCPLC	Plasma Membrane	Zinc finger, RING/FYVE/PHD-type
TraesCS3A02G410900	H2	208	154	195	CTICLGEVEKGDAVKRMPVCLHMFHQQCVDPWLHEHSTCPVC	Plasma Membrane	
TraesCS3A02G414400	HC	393	350	382	CPICLVNPKDMAFGCGHQTCCECGQTLESCPIC	Extracellular	Copine
TraesCS3A02G423800	HC	255	207	244	CCVCMVRHKGAAFIPCGHTFCRLCSRELRHARGNCPLC	Nuclear	
TraesCS3A02G425600	HC	260	204	241	CPVCMNELVNASSTICGHIFCQKCIKASIQAQKKCPTC	Nuclear	Zinc finger, RING/FYVE/PHD-type
TraesCS3A02G425900	HC	240	184	221	CPFCFDELVDASSTICGHIFCHKCIKFSVQAQNRCPAC	Nuclear	Zinc finger, RING/FYVE/PHD-type
TraesCS3A02G426100	HC	282	227	264	CPVCINELVDASSTICGHIFCKKCIEASIRFQKKCPTC	Nuclear	Zinc finger, RING/FYVE/PHD-type
TraesCS3A02G426200	HC	275	220	257	CPVCMNELVDASSTICGHIFCQKCIQASIQAQSKCPTC	Nuclear	Zinc finger, RING/FYVE/PHD-type
TraesCS3A02G426400	HC	287	232	269	CPVCWNELTDASSTICGHIFCQKCIEASIQAQKKCPTC	Nuclear	Zinc finger, RING/FYVE/PHD-type
TraesCS3A02G439700	H2	229	179	219	CPTCLEEYTPENPQIITKCSHHFHLSCIYEWMERSDTCPIC	Nuclear	
TraesCS3A02G440000	H2	289	188	228	CPVCKDKFELGSEAREMPCKHLYHSDCILPWLEQHNSCPVC	Nuclear	

Identifiers	RING type	Protein size (amino acids)	Domain start	Domain end	RING domain	Localization	Description
TraesCS3A02G459400	H2	143	88	131	CAICHLDLETGDEEIVRLPCFHAHAFHTPCI KQWFYQQSKCPIC	Cytoplasmic	
TraesCS3A02G459700	H2	342	122	163	CAVCLMEFGDDDCLRLLPACPHAFHPECIG LWLAKHVTCPLC	Nuclear	
TraesCS3A02G460600	H2	313	207	249	CAICMVDEDASWDEETVALPCSHAFHSGCI LRWFHRAPTCPTC	Cytoplasmic	
TraesCS3A02G464800	H2	180	82	123	CAVCLEDLVAGGRKLRKMDCSHCFHQRCI FRWLHVSRLCPMC	Nuclear	
TraesCS3A02G467300	HC	844	792	830	CGVCFDRPKEVVITKCFHLFCSTCIQRNLEL RHRKCPGC	Cytoplasmic	
TraesCS3A02G491300	HC	268	40	82	CAICLDRIPLQETALVKGCDHAYCVTCILR WASYKQTPVCPQC	Nuclear	
TraesCS3A02G503300	HC	478	415	447	CPICLRNPKDMAFQCGHLTCRECGPTLSTC PVC	Nuclear	Copine
TraesCS3A02G503400	HC	382	338	370	CPICLTNPKDMAFQCGHLTCKECGPTLSTC PLC	Nuclear	Copine
TraesCS3A02G521400	H2	262	182	224	CRICLDEFRDGGKSGPVNLPCSHAYHAHC MLTWLDRGAICPTC	Cytoplasmic	
TraesCS3B02G000800	H2	122	56	111	CAICRNHIMDLCIECQANQASATSEECTVA WGVCNHAFHFHCISRWLKTRQVCPLD	Nuclear	
TraesCS3B02G019200	v	235	55	102	ECRICQEEDWDTGMEAPCACRGSLKYAHR KCIQRWCNEKGDTVCEICL	Plasma Membrane	Protein of unknown function DUF3675
TraesCS3B02G023000	H2	132	66	107	CAVCLAGLQDGEQARFLPRCGHGFHAGCV DTWLASRSTCPLC	Plasma Membrane	
TraesCS3B02G023300	H2	273	97	138	CAVCLAELENGEEARFLPRCGHGFHAGCV NRWLASHTTCPLC	Plasma Membrane	

Identifiers	RING type	Protein size (amino acids)	Domain start	Domain end	RING domain	Localization	Description
TraesCS3B02G060000	H2	233	132	173	CAVCLTEFEDDDDMRLLPHCSHAFHPECID PWLESRTTCPLC	Nuclear	
TraesCS3B02G103300	H2	212	130	171	CAVCLAEFERGEEARALPRCGHRFHVECID AWFRGNSTCPLC	Chloroplast	
TraesCS3B02G111000	HC	418	357	394	CAICQEKMHVPILLRCKHVFCEDCVSEWFE RERTCPLC	Plasma Membrane	
TraesCS3B02G120600	H2	498	453	493	CLVCLEEYKNNDLLGILKCRHDFHTDCIKK WLQVKNACPVC	Nuclear	Zinc finger, RING/FYVE/PHD-type
TraesCS3B02G139600	H2	410	335	375	CCICLSRFSNNEDLRELPCGHVFHMECIDK WLKINALCPLC	Plasma Membrane	
TraesCS3B02G152100	H2	127	32	73	CPICLGQEEATETTWMKTACGHIFHGRCVE RWLKMKGSCPVC	Cytoplasmic	
TraesCS3B02G152400	H2	125	32	73	CPICLGQEEAAATVWKETACGHSFHGRCV ERWLEMKGTCPMC	Cytoplasmic	
TraesCS3B02G164600	H2	238	120	161	CAVCLSELADGDTVRQLPNCGHVFHVECV DVWLRTRTTCPLC	Extracellular	Zinc finger, RING/FYVE/PHD-type
TraesCS3B02G164700	H2	219	114	155	CAVCLSELADGEKVRELPNCGHSFHVECV DAWLRSRTTCPLC	Plasma Membrane	
TraesCS3B02G164800	H2	209	98	139	CAVCLSAVDEGETVRQLPSCKHLFHQECV DVWLLSNASCPVC	Extracellular	
TraesCS3B02G164900	H2	210	128	169	CSICLCTMADGEVARQLPRCMHLFHRACV DMWLTAHTTCPVC	Extracellular	
TraesCS3B02G165000	H2	136	90	131	CPVCLGAVEEGETVRALPVCRHAFHARCV DAWLRLRPTCPVC	Extracellular	
TraesCS3B02G165100	H2	179	100	141	CAVCLGQLEAGEKARRLPKCAHLFHAECV DAWLRAHCTCPMC	Plasma Membrane	

Identifiers	RING type	Protein size (amino acids)	Domain start	Domain end	RING domain	Localization	Description
TraesCS3B02G208200	H2	429	282	334	CKLCLKLLKERSAWNAQDLGVVAVLLCG HVYHADCLDSLTAEAEKYDPPCPVC	Nuclear	
TraesCS3B02G212200	H2	193	105	146	CVFCLSRIDDGEEVRELRCRHVFHRECLDS WLLRPRATCPLC	Plasma Membrane	
TraesCS3B02G218400	H2	372	226	266	CAVCLEDYASGERARELPCRHRFHSQCIVP WLEMHSSCPVC	Nuclear	
TraesCS3B02G272400	H2	526	474	514	CIICQEEYEAEDCVGILDCGHRYHAECLKQ WLTVKNLCPIC	Nuclear	
TraesCS3B02G286400	H2	305	260	300	CCICLAQYREKEEVRQLPCTHMFHLKCVD RWLRIISSCPLC	Plasma Membrane	Zinc finger, RING/FYVE/PHD-type
TraesCS3B02G293600	H2	343	296	336	CSICQEEFEANDELGRLGCGHSYHVYCIKQ WLSQKNVCPVC	Nuclear	
TraesCS3B02G297400	v	261	52	99	ECRICQEEGDQAYMETPCSCKGSLKYAHHI CIQRWCNEKGDTICEICL	Plasma Membrane	Protein of unknown function DUF3675
TraesCS3B02G305200	H2	418	373	413	CPICLDCPADGASLRHLPCAHKFHKECINR WLRMRTSCPVC	Nuclear	Zinc finger, RING/FYVE/PHD-type
TraesCS3B02G313100	H2	184	119	160	CSICLSEYMDGEMLRLMPECRHRFHVMCL DAWLRRSASCPVC	Extracellular	
TraesCS3B02G315100	v	180	58	115	ACRICHLSPEGGDGPAAAPGSEVIRLGCCC KEELGHAHRQCAEAWFRIKGDRRCEICG	Extracellular	Zinc finger, RING/FYVE/PHD-type
TraesCS3B02G323600	H2	301	196	238	CPICFEYLFESRNDVSVLPCGHTIHEKCLRE MKEHCQFACPLC	Extracellular	RCHY1, zinc-ribbon
TraesCS3B02G328200	H2	243	116	157	CTICLGAMQEGEAVRVLPACAHVFHVPCV DTWLASSSSCPVC	Nuclear	

Identifiers	RING type	Protein size (amino acids)	Domain start	Domain end	RING domain	Localization	Description
TraesCS3B02G336200	HC	221	27	67	CNICLELAQDPVVTLCGHLFCWPCLYEWL HVHAHFPECPVC	Extracellular	
TraesCS3B02G357400	HC	420	45	84	CPICLGIIRKTRTVMECLHRFCRDCIDKSMR LGNNECPAC	Nuclear	
TraesCS3B02G361000	H2	343	205	245	CPVCTDRFEVGSEAREMPCKHLYHASCIIP WLVQHNSCPVC	Nuclear	
TraesCS3B02G370700	H2	212	162	202	CPICLEEYDYENPKVVLQCNHNFHLSCIYE WMERSQSCAVC	Extracellular	
TraesCS3B02G376500	H2	208	130	171	CAICLGEFEDGEKVRVLPRCGHEFHVRCVD TWLVSHDSCPTC	Chloroplast	
TraesCS3B02G377500	H2	125	67	109	CAICKGPLAVGGGRCRRLRACGHVYHAEC VDLWLQRKPICPLC	Extracellular	
TraesCS3B02G382600	HC	563	227	267	CNICFDMAGEPVVTSCGHLFCWPCLYQWL NVYSNHKECPVC	Nuclear	
TraesCS3B02G383200	H2	181	120	161	CPICLGEFVEGERVKALPRCGHCFHPECVD AWLCSHPSCPLC	Plasma Membrane	
TraesCS3B02G394000	H2	215	84	124	CPICLDVLEPGASAAETPCQHVYHPACLAP WLEARGTCPVC	Chloroplast	
TraesCS3B02G397000	v	210	90	143	NCRICHLGLESAAAESGGGIVLGCSCKDDL SCAHKQCAETWFKIRGNKICEICG	Extracellular	Zinc finger, RING-CH-type
TraesCS3B02G409700	H2	206	136	177	CAICLADLKQGERVRVLPRCHHGFHVRCID RWLSARQTCPTC	Extracellular	
TraesCS3B02G423300	HC	251	155	192	CGICLEACTKMVLPNCNHAMCINCYRDWY TRSQSCPFC	Extracellular	Zinc finger, RING/FYVE/PHD-type
TraesCS3B02G432700	v	214	27	74	ECRICQEEGEEAAMDSPCACTGTLKFAHRK CIQRWCDKKGNITCEICN	Plasma Membrane	Protein of unknown function DUF3675

Identifiers	RING type	Protein size (amino acids)	Domain start	Domain end	RING domain	Localization	Description
TraesCS3B02G443900	H2	245	193	234	CSVCLQDFGSRQFVRALPQCQHIFHVRCIDDWLPRNSTCPLC	Plasma Membrane	Zinc finger, RING/FYVE/PHD-type
TraesCS3B02G444100	H2	210	154	195	CTICLGKVEKGDAVKRMPACLHMFHQQCIDLWLHEHSTCPVC	Plasma Membrane	
TraesCS3B02G444200	H2	191	136	178	CTICLGEVEKGDMVKRMPACLHMFHQNMCVDSWLHEHSTCPVC	Extracellular	
TraesCS3B02G444300	H2	216	160	201	CVICLGKVKKGDAVKRMPACLHMFHQQCIDPWLHEHSTCPVC	Plasma Membrane	
TraesCS3B02G449200	HC	467	424	456	CPICLVNPKDMAFGCGHQTCCECGQTLESCPIC	Nuclear	Copine
TraesCS3B02G459700	HC	252	204	241	CCVCMARHKGAAFIPCGHTFCRLCSRELRHTRGNCPLC	Nuclear	
TraesCS3B02G461900	HC	260	204	241	CPVCMNELVNASSTICGHIFCQKCIKASIQAQKKCPTC	Nuclear	Zinc finger, RING/FYVE/PHD-type
TraesCS3B02G462000	HC	240	184	221	CPFCFDELVDPSSTMCGHIFCEKCIKASIQAQNRCPAC	Nuclear	Zinc finger, RING/FYVE/PHD-type
TraesCS3B02G462200	HC	208	153	190	CPVCMNELVDASSTICGHIFCQKCIEASIQAQSKCPTC	Nuclear	Zinc finger, RING/FYVE/PHD-type
TraesCS3B02G462300	HC	302	247	284	CPVCISELVDASSTICGHIFCKKCIEASIQFQKKCPTC	Nuclear	Zinc finger, RING/FYVE/PHD-type
TraesCS3B02G462400	HC	306	251	288	CPVCKNELVDASSTICGHIFCQKCIEASIQAQKKCPTC	Nuclear	Zinc finger, RING/FYVE/PHD-type

Identifiers	RING type	Protein size (amino acids)	Domain start	Domain end	RING domain	Localization	Description
TraesCS3B02G462500	HC	284	229	266	CPVCMNELVDASSTICGHIFCQKCIQASIQAQSKCPTC	Nuclear	Zinc finger, RING/FYVE/PHD-type
TraesCS3B02G462600	HC	287	232	269	CPVCRNELTDVSSTICGHIFCQKCIEASIQAQKKCPTC	Nuclear	Zinc finger, RING/FYVE/PHD-type
TraesCS3B02G473700	H2	229	179	219	CPTCLEEYTPENPQIITKCSHHFHLSCIYEWMERSDTCPIC	Nuclear	
TraesCS3B02G474100	H2	288	188	228	CPVCKDKFELESEAREMPCKHLYHSDCILPWLEQHNSCPVC	Nuclear	
TraesCS3B02G484400	H2	142	48	92	CPICMEELVPNSSRGFVTRLPGCLHSFHESCIAQWFCRAATCPCC	Extracellular	
TraesCS3B02G499500	H2	347	121	162	CAVCLLEFGDDDSLRLLPACPHAFHPECIGLWLAKHVTCPLC	Nuclear	
TraesCS3B02G501700	H2	319	211	253	CAICMVDEDASWDEETVALPCSHAFHTGCILTWFHRAPTCPTC	Cytoplasmic	
TraesCS3B02G509100	H2	175	80	121	CAVCLEDLVAGGRKLRKMDCSHCFHQRCIFRWLHVSRLCPMC	Nuclear	
TraesCS3B02G511800	HC	844	792	830	CGVCFDRPKEVVITKCFHLFCSTCIQRNLELRHRKCPGC	Cytoplasmic	
TraesCS3B02G552300	HC	280	43	85	CAICLDRIPLQETALVKGCDHAYCVTCILRWASYKQTPVCPQC	Nuclear	
TraesCS3B02G566700	HC	486	423	455	CPICLRNPRDMAFQCGHLTCRNCGPALSTCPVC	Plasma Membrane	
TraesCS3B02G566800	HC	379	335	367	CPICLTNPKDMAFQCGHLTCKECGPTLSTCPLC	Nuclear	Copine
TraesCS3B02G567600	HC	205	31	74	CAVCGGVDISVPHQANCSHWFCGHCIVGVWLQGSVLRPSNCPIC	Plasma Membrane	

Identifiers	RING type	Protein size (amino acids)	Domain start	Domain end	RING domain	Localization	Description
TraesCS3B02G591100	H2	270	185	227	CRICFDEFRDGGKSGSVNLPCSHAYHAHCMLTWLDRGANCPTC	Nuclear	
TraesCS3B02G595200	H2	399	201	241	CAVCKEAFELGDEAREMPCGHMYHQDCILPWLALRNSCPVC	Nuclear	
TraesCS3D02G019900	v	235	55	102	ECRICQEEDWDAGMEAPCACRGSLKYAHRKCIQRWCSEKGDTVCEICL	Plasma Membrane	Protein of unknown function DUF3675
TraesCS3D02G024200	H2	266	97	138	CAVCLAELEDGEEARFLPRCGHGFHAGCVDRWLAFHTTCPLC	Plasma Membrane	
TraesCS3D02G050600	H2	349	130	171	CAVCLTEFEDADDLRLLPHCSHAFHPDCIDPWLETRTTCPLC	Plasma Membrane	
TraesCS3D02G079000	HC	374	84	118	CMVCQALLHDASKCKPCSHKFCKACISRFKDCPLC	Nuclear	Tetratricopeptide-like helical domain superfamily
TraesCS3D02G088100	H2	215	130	171	CAVCLAEFERGEEARALPRCGHRFHVECIDAWFRGNSTCPLC	Chloroplast	
TraesCS3D02G088200	H2	212	127	168	CAVCLAEFERGEEARALPRCGHRFHVECIDAWFRGNSTCPLC	Nuclear	
TraesCS3D02G095600	HC	415	354	391	CAICQEKMHVPILLRCKHVFCEDCVSEWFERERTCPLC	Plasma Membrane	
TraesCS3D02G105300	H2	518	473	513	CLVCLEEYKDNELLGILKCRHDFHTDCIKKWLQVKNACPVC	Nuclear	Zinc finger, RING/FYVE/PHD-type
TraesCS3D02G122200	H2	410	335	375	CCICLSRFSNNEDLRELPCGHVFHMECIDKWLKINALCPLC	Plasma Membrane	
TraesCS3D02G135000	H2	121	32	73	CPICLGQEEAAATAWKETACGHSFHGRCVERWLETKGTCPMC	Cytoplasmic	
TraesCS3D02G147100	H2	217	123	164	CAVCLSELADGDTVRQLPNCGHVFHVECVDAWLRTRTTCPLC	Plasma Membrane	

Identifiers	RING type	Protein size (amino acids)	Domain start	Domain end	RING domain	Localization	Description
TraesCS3D02G147200	H2	219	114	155	CAVCLSELADGEKVRELPNCGHSFHVECV DAWLRSRTTCPLC	Plasma Membrane	
TraesCS3D02G147400	H2	204	98	139	CAVCLSAVDEGETVRQLPSCKHLFHQECV DVWLLSNASCPVC	Extracellular	
TraesCS3D02G147500	H2	203	103	144	CAVCLGALDERERVRQLPACNHLFHQGCV DVWLMSNASCPVC	Extracellular	
TraesCS3D02G147600	H2	210	128	169	CSICLCTMADGEVARQLPRCTHLFHRACVD MWLTAHTTCPVC	Extracellular	
TraesCS3D02G147700	H2	146	100	141	CPVCLGAVEEGETVRALPVCRHAFHARCV DAWLRLRPTCPVC	Extracellular	
TraesCS3D02G147800	H2	180	99	140	CAVCLGQLEAGEKARRLPKCAHLFHAECV DAWLRAHCTCPMC	Chloroplast	
TraesCS3D02G183800	H2	428	281	333	CKLCLKPLKERSAWNAQDLGVVAVLLCGH VYHADCLDSLTAEAEKYDPPCPVC	Nuclear	Zinc finger, RING/FYVE/PHD-type
TraesCS3D02G184200	H2	505	292	329	CIICREEMTTAKKLLCGHLFHVHCLRSWLE RQHTCPTC	Plasma Membrane	
TraesCS3D02G186800	H2	180	92	133	CVFCLSRIDDGEEVRELRCRHIFHRECLDS WLLCPRATCPLC	Plasma Membrane	
TraesCS3D02G192700	H2	368	223	263	CAVCLEDYACGERARELPCRHRFHSQCIVP WLEMHSSCPVC	Nuclear	
TraesCS3D02G244600	H2	528	476	516	CIICQEEYKAEDCVGILDCGHRYHAECLKQ WLTVKNLCPIC	Nuclear	
TraesCS3D02G255300	H2	305	260	300	CCICLAQYREKEEVRQLPCTHMFHLKCVD RWLRIISSCPLC	Plasma Membrane	Zinc finger, RING/FYVE/PHD-type
TraesCS3D02G259900	HC	271	223	260	CCVCMVRHKGAAFIPCGHTFCRLCSRELW VSRGNCPLC	Nuclear	

Identifiers	RING type	Protein size (amino acids)	Domain start	Domain end	RING domain	Localization	Description
TraesCS3D02G260700	H2	200	153	193	CSICQEEFEANDEMGRLGCGHSYHVYCIKQWLSQKNVCPVC	Nuclear	
TraesCS3D02G264100	v	280	71	118	ECRICQEEGDQAYMETPCSCKGSLKYAHHICIQRWCNEKGDTICEICL	Plasma Membrane	Protein of unknown function DUF3675
TraesCS3D02G271100	H2	428	383	423	CPICLDCPADGASLRHLPCAHKFHKECIDRWLRMRTSCPVC	Nuclear	Zinc finger, RING/FYVE/PHD-type
TraesCS3D02G279200	H2	194	119	160	CSICLSEYMDGEMLRLMPECRHRFHVMCLDAWLRRSASCPVC	Plasma Membrane	
TraesCS3D02G281400	v	149	60	113	ACRICHLSPEGGDGPGSEVIRLACCCKEELGHAHRQCAEAWFRIKGDRRCEICG	Extracellular	Zinc finger, RING/FYVE/PHD-type
TraesCS3D02G288700	H2	327	222	264	CPICFEYLFESRNDVSVLPCGHTIHEKCLKEMKEHCQFACPLC	Extracellular	RCHY1, zinc-ribbon
TraesCS3D02G293200	H2	243	116	157	CTICLSAMQEGEAVRVLPACAHVFHVPCVDTWLASSSSCPVC	Nuclear	
TraesCS3D02G293300	H2	243	116	157	CTICLGAMQEGEAVRVLPACAHVFHVPCVDTWLASSSSCPVC	Nuclear	
TraesCS3D02G301500	HC	221	28	68	CNICLELAQDPVVTLCGHLFCWPCLYEWLHVHAHFPECPVC	Extracellular	
TraesCS3D02G321400	HC	481	106	145	CPICLGIIRKTRTVMECLHRFCRDCIDKSMRLGNNECPAC	Nuclear	
TraesCS3D02G324300	H2	342	203	243	CPVCTDRFEVGSEAREMPCKHLYHASCIIPWLVQHNSCPVC	Nuclear	
TraesCS3D02G332600	H2	215	165	205	CPICLEEYDYENPKIVLQCNHNFHLSCIYEWMERSQSCAVC	Extracellular	
TraesCS3D02G338100	H2	195	117	158	CAICLGEFEDGEKVRVLPRCGHEFHVRCVDTWLVSHDSCPTC	Plasma Membrane	

Identifiers	RING type	Protein size (amino acids)	Domain start	Domain end	RING domain	Localization	Description
TraesCS3D02G339500	H2	127	69	111	CAICKGPLAAGGGRCRRLRACGHVYHAEC VDLWLQRKPICPLC	Extracellular	
TraesCS3D02G344000	HC	567	230	270	CNICFEMAGEPVVTSCGHLFCWPCLYQWL NVYSNHKECPVC	Nuclear	
TraesCS3D02G344500	H2	181	120	161	CPICLGEFADGERVKALPRCGHCFHPECVD AWLCSHPSCPLC	Extracellular	
TraesCS3D02G358300	v	250	130	183	NCRICHLGLESAAAESGAGIVLGCSCKDDL SCAHKQCAETWFKIRGNKICEICG	Extracellular	Zinc finger, RING-CH-type
TraesCS3D02G370200	H2	206	136	177	CAICLADLKQGERVRVLPRCHHGFHVRCID RWLSARQTCPTC	Extracellular	
TraesCS3D02G384200	HC	251	155	192	CGICLETCTKMVLPNCNHAMCINCYRDWY TRSQSCPFC	Extracellular	Zinc finger, RING/FYVE/PHD-type
TraesCS3D02G393800	v	252	27	74	ECRICQEEGEEAAMDSPCACTGTLKFAHRK CIQRWCDKKGNITCEICN	Plasma Membrane	Protein of unknown function DUF3675
TraesCS3D02G400900	HC	281	24	72	CPICLEAIEDEAYLDACLHSFCYRCITQWVS IVASKREEPLSTARCPLC	Nuclear	
TraesCS3D02G405200	H2	246	194	235	CSVCLQDFGSRQFVRALPQCQHIFHVRCID DWLQRNASCPLC	Plasma Membrane	Zinc finger, RING/FYVE/PHD-type
TraesCS3D02G405400	H2	213	157	198	CTICLGEVEKGDAVKRMPVCLHMFHRQCV DQWLHEHSTCPVC	Plasma Membrane	
TraesCS3D02G408900	HC	466	423	455	CPICLVNPKDMAFGCGHQTCCECGQTLESC PIC	Nuclear	Copine
TraesCS3D02G419000	HC	249	201	238	CCVCMVRHKGAAFIPCGHTFCRLCSRELRH TRGNCPLC	Nuclear	

Identifiers	RING type	Protein size (amino acids)	Domain start	Domain end	RING domain	Localization	Description
TraesCS3D02G420400	HC	264	208	245	CPVCMNELVNASSTICGHIFCQKCIKASIQAQKKCPTC	Nuclear	Zinc finger, RING/FYVE/PHD-type
TraesCS3D02G420500	HC	233	177	214	CPFCFEELVDAASTICGHIFCHKCIEFSIQAQSRCPSC	Nuclear	Zinc finger, RING/FYVE/PHD-type
TraesCS3D02G420700	HC	208	153	190	CPVCMNELVDASSTICGHIFCQKCIEASIQAQSKCPTC	Nuclear	Zinc finger, RING/FYVE/PHD-type
TraesCS3D02G420800	HC	214	159	196	CPVCINELVDASSTICGHIFCKKCIEASIRFQKKCPTC	Nuclear	Zinc finger, RING/FYVE/PHD-type
TraesCS3D02G420900	HC	286	231	268	CPVCINELVDASSTICGHIFCKKCIEASIRFQKKCPTC	Nuclear	
TraesCS3D02G421000	HC	275	220	257	CPVCMNELVDASSTICGHIFCQKCIQASIQAQSKCPTC	Nuclear	Zinc finger, RING/FYVE/PHD-type
TraesCS3D02G421100	HC	272	217	254	CPVCMNELVDASSTICGHIFCQNCIEASIQAQSKCPTC	Nuclear	Zinc finger, RING/FYVE/PHD-type
TraesCS3D02G421200	HC	287	232	269	CPVCWNELIDASSTICGHIFCQKCIEASIQAQKKCPTC	Nuclear	Zinc finger, RING/FYVE/PHD-type
TraesCS3D02G432500	H2	229	179	219	CPTCLEEYTPENPQIITKCSHHFHLSCIYEWMERSDTCPIC	Nuclear	
TraesCS3D02G432800	H2	289	188	228	CPVCKDKFELGSEAREMPCKHLYHSDCILPWLEQHNSCPVC	Nuclear	

Identifiers	RING type	Protein size (amino acids)	Domain start	Domain end	RING domain	Localization	Description
TraesCS3D02G451600	H2	269	197	240	CVICLLDLESEEDDEEQVRLPCSHAFHTPCIELWFDRASTCPTC	Cytoplasmic	
TraesCS3D02G452500	H2	346	121	162	CAVCLLEFGDDDSLRLLPACPHAFHPECISLWLAKHVTCPLC	Nuclear	
TraesCS3D02G453900	H2	247	189	230	CAICMSDEGTNDEETVRLPCSHVFHGRCIGPWFHRVSTCPTC	Cytoplasmic	
TraesCS3D02G454100	H2	355	205	247	CAICMVDEDASWDEETVALPCSHAFHSGCILTWFHRAPTCPTC	Cytoplasmic	
TraesCS3D02G459900	H2	176	91	132	CAVCLEDLVAGGRKLRKMDCSHCFHQRCIFRWLHVSRLCPMC	Nuclear	
TraesCS3D02G462600	HC	830	778	816	CGVCFDRPKEVVITKCFHLFCSTCIQRNLELRHRKCPGC	Cytoplasmic	
TraesCS3D02G465000	HC	165	113	151	CGVCFDRPKEVVIAKCFHLFCSTCIQRNLELRHRKCPGC	Nuclear	Zinc finger, RING/FYVE/PHD-type
TraesCS3D02G466100	HC	844	792	830	CGVCFDRPKEVVITKCFHLFCSTCIQRNLELRHRKCPGC	Cytoplasmic	
TraesCS3D02G495500	H2	386	130	171	CAVCLSEFDDDETLRLLPKCSHVFHPDCIDTWLASHITCPVC	Plasma Membrane	
TraesCS3D02G497200	HC	270	42	84	CAICLDRIPLQETALVKGCDHAYCVTCILRWASYKQTPVCPQC	Nuclear	
TraesCS3D02G509900	HC	477	415	447	CPICLRNPRDMAFQCGHLTCRECGPTLSTCPVC	Cytoplasmic	Copine
TraesCS3D02G510000	HC	344	300	332	CPICLTNPKDMAFQCGHLTCKECGPTLSTCPLC	Nuclear	Copine
TraesCS3D02G511300	HC	206	31	74	CAVCRGVDISVPHQANCSHWFCGHCIVGVWLQGSVLRPSNCPVC	Plasma Membrane	

Identifiers	RING type	Protein size (amino acids)	Domain start	Domain end	RING domain	Localization	Description
TraesCS3D02G532300	H2	386	188	228	CAVCKEAFQLGDEAREMPCSHMYHQDCILPWLALRNSCPVC	Nuclear	
TraesCS4A02G011000	HC	378	326	365	CVICLRKRRRAAFVPCGHLVCCCNCAKRVELMDEPLCPVC	Plasma Membrane	E3 Ubiquitin ligase, GIDE-type
TraesCS4A02G034600	H2	132	89	130	CAVCLTEYADGDDLRWLPRCRHAFHRSCVDEWLRRRPSCPLC	Extracellular	
TraesCS4A02G049500	H2	275	227	267	CSICLDGFYDRDELIRLRCGHRFHSTCLEPWVRKCADCPYC	Nuclear	
TraesCS4A02G053400	HC	260	46	100	CPICCESFNIVENVPYVLWCGHTMCKNCILGLQWAVVKFPTLPIQLPLFVSCPWC	Plasma Membrane	
TraesCS4A02G082400	HC	326	262	299	CFICREPFVDPVVTKCKHYFCEHCALKHHSKNKKCFVC	Nuclear	
TraesCS4A02G083500	HC	405	327	365	CVICLSEPRDTTVLPCRHMCMCSECAKVLRYQTTRCPIC	Nuclear	
TraesCS4A02G089200	HC	339	293	327	CRVCKSSEACMLLLPCRHLCLCKECESKLSFCPLC	Nuclear	E3 ubiquitin-protein ligase BOI-like
TraesCS4A02G095800	H2	280	137	177	CAVCQEAFEPGAAAREMPCKHVYHQDCILPWLSLRNSCPIC	Nuclear	
TraesCS4A02G101400b	HC	304	214	255	CPSCKSNLTNTMSLVAISTCGHVFCKKCSDKFIATDKVCLMC	Nuclear	Nitric oxide synthase-interacting protein
TraesCS4A02G104900	H2	387	165	207	CAVCLAEFADDGEKLRLLPGCCHVFHAACIDVWLAAHVTCPVC	Plasma Membrane	Zinc finger, RING/FYVE/PHD-type
TraesCS4A02G112500	H2	425	138	179	CAVCLCEFADDDRLRLLPKCSHAFHVECIDTWLLSHSTCPLC	Plasma Membrane	

Identifiers	RING type	Protein size (amino acids)	Domain start	Domain end	RING domain	Localization	Description
TraesCS4A02G112900	H2	250	198	238	CVICQMEYRRGDLQMALPCKHVYHASCVTKWLTINKVCPVC	Nuclear	Zinc finger, RING/FYVE/PHD-type
TraesCS4A02G117900	HC	871	525	566	CGICLSEDQRTTLQGVLNCCSHYFCFACIMEWSKVESRCPLC	Nuclear	
TraesCS4A02G118100	H2	84	23	76	CGICRMAFDGCCPDCKFPGDDCPIIWGACNHAFHLHCILKWVNSQTSTPLCPMC	Extracellular	
TraesCS4A02G128700	H2	167	83	125	CVICLGGMEEGDEARVLRCQHLFHRCCLDWWLAARPGATCPLC	Extracellular	
TraesCS4A02G129800	H2	324	217	257	CPVCLDEFAAGGEAKEMPCKHRFHDVCLLPWLEANSSCPVC	Nuclear	
TraesCS4A02G137300	H2	162	113	154	CAVCICELRDGDIGRRLPRCGHRFHSECVDRWFRSHATCPLC	Chloroplast	
TraesCS4A02G137800	H2	159	90	131	CAVCLAEMTDGERGRLLPRCGHRFHVECIDRWFRSNSSCPLC	Extracellular	
TraesCS4A02G156600	v	323	35	84	QCRICLETDGRDFIAPCKCKGTSKYVHRDCLDHWRAVKEGFAFSHCTTCK	Plasma Membrane	Zinc finger, RING/FYVE/PHD-type
TraesCS4A02G161100	H2	220	170	210	CPTCLEDYTSENPRIVMQCSHHFHLGCIYEWMERSEACPVC	Nuclear	
TraesCS4A02G162000	HC	480	193	230	CTLCFKLLFKPVTTPCGHTFCRSCLHQAMDHGNKCPMC	Nuclear	PUA-like superfamily
TraesCS4A02G164100	v	271	50	97	ECRICQEEDSIKNLEKPCTCNGSLKYAHRACVQRWCNEKGDITCEICH	Cytoplasmic	Protein of unknown function DUF3675
TraesCS4A02G165300	H2	364	33	73	CSICLEAFCESDPSALTGCKHEFHLQCILEWCQRSSQCPMC	Nuclear	

Identifiers	RING type	Protein size (amino acids)	Domain start	Domain end	RING domain	Localization	Description
TraesCS4A02G174400	H2	173	98	140	CPVCLADFADGEAVRVLPECLHYFHADCID TWLRAGNTSCPMC	Extracellular	
TraesCS4A02G174500	H2	151	101	142	CPVCLSDFADGEAVSVLPACMHYFHAACV GEWMRARATCPLC	Extracellular	
TraesCS4A02G174600	H2	159	98	139	CPVCLSDFADGETVRVLPACMHYFHAACV GEWLRRNTTCPVC	Extracellular	
TraesCS4A02G183700	H2	255	105	146	CVVCLAEYEDDDVLRVLPYCGHDFHVACI DIWLKQHSTCPVC	Extracellular	
TraesCS4A02G185500	H2	153	82	124	CAVCLEDLEGGDDDKLRAMPCSHCFHQRC IFRWLRVSRVCPCC	Cytoplasmic	
TraesCS4A02G185700	H2	212	105	145	CVVCLESFKDGDDIRKLPCSHGFHNSCISD WLRVSCLCPQC	Nuclear	
TraesCS4A02G194200	H2	182	124	165	CAICLGEFADGEKVRVLPRCHHGFHVRCID MWLATHTSCPNC	Plasma Membrane	Zinc finger, RING/FYVE/PHD-type
TraesCS4A02G220900	H2	183	111	152	CAVCLSAMQDGDAVRALPGCRHAFHVGC VDVWLCAQATCPVC	Chloroplast	
TraesCS4A02G222800	HC	469	139	187	CSICLNLWHDVVTVAPCFHNFCNGCFSEW LRRSSSKSRDKSQSAACPQC	Extracellular	E3 ubiquitin-protein ligase CHFR, cysteine rich domain with multizinc binding
TraesCS4A02G237400	H2	264	207	248	CSICLEGCHGASGGLIQLRCKHIFHSACLEQ WLQSRADCPYC	Nuclear	
TraesCS4A02G249200	H2	261	150	192	CPICYEYLFDSLREASVLRCGHTMHLQCFH EMLKHDKFTCPMC	Extracellular	Zinc finger, CHY-type

Identifiers	RING type	Protein size (amino acids)	Domain start	Domain end	RING domain	Localization	Description
TraesCS4A02G271200	HC	473	426	460	CRICYEGEICMVILPCRHRTLCKSCAEKCKRCPIC	Plasma Membrane	Transmembrane Fragile-X-F-associated protein
TraesCS4A02G290600	HC	237	25	65	CNICFELPQEPIVTLCGHLFCWPCLYRWLHMHANTPECPVC	Extracellular	
TraesCS4A02G311200	HC	715	663	701	CCVCCDTPIDSLLYRCGHMCTCSKCANELVRSGGKCPLC	Nuclear	
TraesCS4A02G320900	H2	259	195	233	CRVCMEAKEDTMVRLPCSHSFHRGCILPCFHKVATCPMC	Mitochondrial	
TraesCS4A02G321200	H2	209	141	184	CCVCMEDFAAAIDGDTTVRLPCSHAFHRACILPWFYKASTCPKC	Extracellular	
TraesCS4A02G322600	H2	1919	1869	1915	CPICYSILHTSNHGLPRLACKTCKHKFHGACLYKWFSTSNKSTCPLC	Plasma Membrane	Listerin, zinc finger, RING-type
TraesCS4A02G338900	H2	312	103	144	CTVCLEEYEAKDVVRVLPYCGHAFHAACIDTWLRQHPTCPIC	Extracellular	Zinc finger, RING/FYVE/PHD-type
TraesCS4A02G352200	HC	196	21	63	CSVCHDRFRIPCQANCSHWFCGECIIRVWNHGAAVQPCKCPIC	Plasma Membrane	
TraesCS4A02G385800	H2	197	106	150	CPICMEEDADEDAATGAWKETPCGHRFHGRCVERWLHAKGSCPMC	Cytoplasmic	
TraesCS4A02G385900	H2	183	112	151	CPICLEDDDTAAWKETPCGHRFHGRCVQRWLQAKGSCPMC	Cytoplasmic	
TraesCS4A02G404600	H2	355	134	175	CAVCLTEFEDSDELRVLPACCHVFHPECIDPWLAGAVTCPLC	Plasma Membrane	
TraesCS4A02G415800	H2	260	105	146	CAVCLSEFAERERVRLLPGCSHAFHIDCIDTWLQGSARCPFC	Nuclear	
TraesCS4A02G415900	H2	281	114	155	CAVCLSEFAPGERLKMLPACSHAFHIDCIDTWLHHNVSCPLC	Plasma Membrane	

Identifiers	RING type	Protein size (amino acids)	Domain start	Domain end	RING domain	Localization	Description
TraesCS4A02G441400	H2	250	202	242	CTVCLSAFRNRENIITLPCKHNYHASCISSWLKINKTCPVC	Nuclear	
TraesCS4A02G463800	H2	151	95	137	CAVCLADFHGATRVRRPHGCGHVFHRACLDRWAAYGHRTCPLC	Extracellular	
TraesCS4A02G463900	H2	163	104	146	CAVCLGNFHGAAHVRRPRGCRHVFHRACLDRWAAHGHSTCPLC	Extracellular	
TraesCS4A02G464300	H2	174	108	150	CAVCLGDFHGAARVRRPRGCRHVFHRGCLDRWAAHGHRTCPLC	Extracellular	
TraesCS4A02G465400	H2	145	86	128	CVVCLSDFHGAARVRRPRGCQHVFHFGCLDRWAAHGHRTCPLC	Extracellular	
TraesCS4A02G468600	H2	145	86	128	CVVCLSDFHGTACVRRPRRCRHVFHFGCLDRWAAHGHRTCPLC	Extracellular	
TraesCS4A02G499200	H2	335	203	243	CAVCMDDFLLGAAAKQLPCNHVFHKDCILPWLDLHSSCPVC	Nuclear	
TraesCS4B02G003100	HC	711	659	697	CCVCCDTPIDSLLYRCGHMCTCSKCANELVRSGGKCPLC	Nuclear	
TraesCS4B02G023600	HC	235	25	65	CNICFELPQEPIVTLCGHLFCWPCLYRWLHMHANTPECPVC	Extracellular	
TraesCS4B02G042900	HC	473	426	460	CRICYEGEICMVILPCRHRTLCKSCAEKCKRCPIC	Plasma Membrane	Transmembrane Fragile-X-F-associated protein
TraesCS4B02G065500	H2	302	148	190	CPICYEYLFDSLREASVLRCGHTMHLQCFHDMLKHDKFTCPMC	Extracellular	Zinc finger, CHY-type
TraesCS4B02G077700	H2	262	202	243	CSICLEGCHGASDGLIQLRCKHIFHSACLEQWLQSRADCPYC	Nuclear	Zinc finger, RING/FYVE/PHD-type
TraesCS4B02G091300	H2	219	159	200	CCICLSYLVEDQDAVRLPGCSHAFHRSCLTPWLQRAKTCPMC	Mitochondrial	

Identifiers	RING type	Protein size (amino acids)	Domain start	Domain end	RING domain	Localization	Description
TraesCS4B02G093600	HC	310	139	187	CSICLNLWHDVVTVAPCFHNFCNGCFSEW LRRSSSKSRDKSQSAACPQC	Extracellular	Zinc finger, RING/FYVE/PHD-type
TraesCS4B02G099300	H2	183	111	152	CAVCLSAMQDGDAVRALPGCRHAFHVGC VDVWLCAHATCPVC	Nuclear	Zinc finger, RING/FYVE/PHD-type
TraesCS4B02G121100	H2	186	128	169	CAICLGEFADGEKVRVLPRCHHGFHVRCID MWLATHTSCPNC	Plasma Membrane	
TraesCS4B02G134900	H2	254	105	146	CVVCLAEYEDDDVLRVLPYCGHDFHVACT DIWLKQHSTCPVC	Extracellular	Zinc finger, RING/FYVE/PHD-type
TraesCS4B02G142800	H2	152	91	132	CPVCLSDFADGETVRVLPACMHYFHAACV GEWLRRNTTCPVC	Extracellular	
TraesCS4B02G143100	H2	173	98	140	CPVCLADFADGEAVRVLPECLHYFHADCID TWLRARNTSCPMC	Extracellular	
TraesCS4B02G149000	v	272	50	97	ECRICQEEDSIKNLEKPCTCNGSLKYAHRA CVQRWCNEKGDITCEICH	Nuclear	Protein of unknown function DUF3675
TraesCS4B02G151200	HC	480	193	230	CTLCFKLLFEPVTTPCGHTFCRSCLHQSMD HGNKCPMC	Nuclear	PUA-like superfamily
TraesCS4B02G162300	H2	190	124	165	CSICISALVAGDKVKALPPCGHCFHPECVD AWLRSQPSCPLC	Extracellular	
TraesCS4B02G164000	v	323	35	84	QCRICLETDGRDFIAPCKCKGTSKYVHRDC LDHWRAVKEGFAFSHCTTCK	Plasma Membrane	Zinc finger, RING/FYVE/PHD-type
TraesCS4B02G167400	H2	159	90	131	CAVCLAEMTDGERGRLLPRCGHRFHVECI DRWFRSNSTCPLC	Extracellular	
TraesCS4B02G174800	H2	350	243	283	CPVCLDEFAAGGEAKEMPCKHRFHDVCILP WLEAHSSCPVC	Nuclear	

Identifiers	RING type	Protein size (amino acids)	Domain start	Domain end	RING domain	Localization	Description
TraesCS4B02G176000	H2	154	68	110	CVICLGGMEEGDEVRVLRCHHLFHRCCLA RWLAAWAGATCPLC	Extracellular	
TraesCS4B02G176100	H2	154	68	110	CVICLGGMEEGDEVRVLRCHHLFHRCCLA RWLAAWAGATCPLC	Extracellular	
TraesCS4B02G186500	HC	868	522	563	CGICLSEDQRTTLQGVLNCCSHYFCFACIM EWSKVESRCPLC	Nuclear	
TraesCS4B02G191300	H2	262	198	238	CVICQMEYRRGDLQMALPCKHVYHASCVT KWLTINKVCPVC	Nuclear	Zinc finger, RING/FYVE/PHD-type
TraesCS4B02G191600	H2	425	138	179	CAVCLCEFADDDRLRLLPKCSHAFHVECID TWLLSHSTCPLC	Plasma Membrane	
TraesCS4B02G199100	H2	447	190	232	CAVCLAEFADDGEKLRLLPGCCHVFHAACI DVWLAAHVTCPVC	Plasma Membrane	
TraesCS4B02G203200b	HC	304	214	255	CPSCKSNLTNTMSLVAISTCGHVFCKKCSD KFIATDKVCLMC	Nuclear	Nitric oxide synthase-interacting protein
TraesCS4B02G208600	H2	280	220	260	CSVCLEQVVAGDLLRSLPCLHQFHVNCIDP WLRQQGTCPIC	Extracellular	
TraesCS4B02G209100	H2	280	137	177	CAVCQEAFEPGAAAREMPCKHVYHQDCIL PWLSLRNSCPIC	Nuclear	
TraesCS4B02G215100	HC	339	293	327	CRVCKSSEACMLLLPCRHLCLCKECESKLS FCPLC	Nuclear	E3 ubiquitin-protein ligase BOI-like
TraesCS4B02G220700	HC	405	327	365	CVICLSEPRDTTVLPCRHMCMCSECAKVLR YQTTRCPIC	Nuclear	
TraesCS4B02G221600	HC	325	261	298	CFICREPFVDPVVTKCKHYFCEHCALKHHS KNKKCFVC	Nuclear	

Identifiers	RING type	Protein size (amino acids)	Domain start	Domain end	RING domain	Localization	Description
TraesCS4B02G251200	HC	259	46	100	CPICCESFNIVENVPYVLWCGHTMCKNCIL GLQWAVVKFPTLPIQLPLFVSCPWC	Plasma Membrane	
TraesCS4B02G255400	H2	276	227	267	CSICLDGFYDGDELIRLRCRHRFHSTCLEPW VRKCADCPYC	Nuclear	Zinc finger, RING/FYVE/PHD-type
TraesCS4B02G270600	H2	150	107	148	CSVCLAEYADGDELRWLPGCRHAFHRSCV DEWLRRRPSCPLC	Extracellular	
TraesCS4B02G293400	HC	378	326	365	CVICLRKRRRAAFVPCGHLVCCCNCAKRV ELMDEPLCPVC	Plasma Membrane	E3 Ubiquitin ligase, GIDE-type
TraesCS4B02G304400	H2	355	134	175	CAVCLTEFEDSDELRVLPACCHVFHPDCID PWLAGAVTCPLC	Plasma Membrane	
TraesCS4B02G316600	H2	261	108	149	CAVCLSEFAERERVRLLPGCSHAFHIDCIDT WLQGSARCPFC	Nuclear	
TraesCS4B02G316700	H2	393	222	263	CAVCLSEFEPGERLKMLPACSHAFHIDCIDT WLHHNVSCPLC	Plasma Membrane	
TraesCS4B02G322200	H2	210	120	161	CAICLSEYAGKEEIRVLPQCGHGFHVACVD AWLRAHSSCPSC	Extracellular	
TraesCS4B02G344100	H2	707	80	123	CAICLGSMRTGHGQALFTAECSHKFHFHCI TSNVRHGNHICPIC	Nuclear	
TraesCS4B02G344200	H2	721	10	53	CAICLGGMAAGGGQATFTAECSHTFHFNCI SASVAHGHLVCPLC	Nuclear	
TraesCS4B02G361300a	H2	475	182	223	CPVCLEPLHGRTNIMAAHECAHVFHASCIE PWLHNRGSCPVC	Extracellular	
TraesCS4B02G361300b	H2	475	249	291	CPVCLEPLLLDCGDVRAAHACGHVFHSCCI EPWLNQQGSCPVC	Extracellular	
TraesCS4B02G361300c	H2	475	327	369	CPVCLEPLRAGSGDIRAAHACGHVFHSRCI ERWVRRRGSCPVC	Extracellular	

Identifiers	RING type	Protein size (amino acids)	Domain start	Domain end	RING domain	Localization	Description
TraesCS4B02G361300d	H2	475	423	465	CPVCLEPLSVGGGDMKAAHVCGHVFHSRC IERWLRHRNSCPVC	Extracellular	
TraesCS4B02G380900a	G	497	58	104	CPCCTEPWTSDGPHRMCCIPCGHVYGRSC METLLHRSGQNSAKCPQC	Cytoplasmic	
TraesCS4B02G381000a	G	557	58	104	CPCCTEPWTSDGPHRMCCIRCGHVYGRSC METLLRRSGQNSAKCPQC	Cytoplasmic	
TraesCS4B02G381000b	G	557	320	404	CSICMEPWTSTDEHHIWRLWVHDAADVAP YDHIVQEGSLCWTFHDHLPSPTCSCIPCGH VYGRSCLIRWLRDQLSRNENLKCPQC	Cytoplasmic	
TraesCS4D02G003000	HC	674	622	660	CCVCCDTPIDSLLYRCGHMCTCSKCANELV RSGGKCPLC	Nuclear	
TraesCS4D02G021200	HC	235	25	65	CNICFELPQEPIVTLCGHLFCWPCLYRWLH MHANTPECPVC	Extracellular	
TraesCS4D02G040200	HC	473	426	460	CRICYEGEICMVILPCRHRTLCKSCAEKCKR CPIC	Plasma Membrane	Transmembrane Fragile-X-F-associated protein
TraesCS4D02G064500	H2	249	148	190	CPICYEYLFDSLRESSVLRCGHTMHLQCFH EMLKHDKFTCPMC	Extracellular	Zinc finger, CHY-type
TraesCS4D02G076100	H2	263	206	247	CSICLEGCHGASDGLIQLRCKHIFHLACLEQ WLQSRADCPYC	Extracellular	
TraesCS4D02G090400	HC	468	139	187	CSICLNLWHDVVTVAPCFHNFCNGCFSEW LRRSSSKSRDKSQSAACPQC	Extracellular	E3 ubiquitin-protein ligase CHFR, cysteine rich domain with multizinc binding
TraesCS4D02G095800	H2	183	111	152	CAVCLSAMQDGDAVRALPGCRHAFHVGC VDVWLCARATCPVC	Nuclear	

Identifiers	RING type	Protein size (amino acids)	Domain start	Domain end	RING domain	Localization	Description
TraesCS4D02G119100	H2	186	128	169	CAICLGEFADGEKVRVLPRCHHGFHVRCID MWLATHTSCPNC	Plasma Membrane	
TraesCS4D02G127900	H2	191	86	126	CVVCLESFKEGDDIRKLPCSHGFHNSCISD WLRVSCLCPQC	Nuclear	
TraesCS4D02G128100	H2	153	82	124	CAVCLEDFEGGGDDKLRAMPCSHCFHQRC IFRWLSVSRVCPCC	Cytoplasmic	
TraesCS4D02G129700	H2	255	105	146	CVVCLAEYEDDDVLRVLPYCGHDFHVACI DIWLKQHSTCPVC	Extracellular	
TraesCS4D02G141100	H2	152	91	132	CPVCLSDFADGETVRVLPACMHYFHAACV GEWLRRNTTCPVC	Extracellular	
TraesCS4D02G141200	H2	173	98	140	CPVCLADFADGEAVRVLPECLHYFHEDCID TWLRAGNTSCPMC	Extracellular	
TraesCS4D02G147800	v	275	50	97	ECRICQEEDSIKNLEKPCTCNGSLKYAHRA CVQRWCNEKGDITCEICH	Nuclear	Protein of unknown function DUF3675
TraesCS4D02G148400	H2	220	170	210	CPTCLEDYTSENPRIVMQCSHHFHLGCIYE WMERSEACPVC	Nuclear	
TraesCS4D02G152900	v	323	35	84	QCRICLETDGRDFIAPCKCKGTSKYVHRDC LDHWRAVKEGFAFSHCTTCK	Plasma Membrane	Zinc finger, RING/FYVE/PHD-type
TraesCS4D02G155700	HC	480	193	230	CTLCFKLLFEPVTTPCGHTFCRSCLHQAMD HGNKCPMC	Nuclear	PUA-like superfamily
TraesCS4D02G158400	H2	389	33	73	CSICLEAFCESDPSTLTGCKHEFHLQCILEW CQRSSQCPMC	Nuclear	
TraesCS4D02G169900	H2	159	90	131	CAVCLAEMTDGERGRLLPRCGHRFHVECI DRWFRSNSTCPLC	Extracellular	
TraesCS4D02G170000	H2	159	110	151	CAVCICELRDGDTGRRLPRCGHRFHAECVD RWFRSHATCPLC	Chloroplast	

Identifiers	RING type	Protein size (amino acids)	Domain start	Domain end	RING domain	Localization	Description
TraesCS4D02G176700	H2	348	241	281	CPVCLDDFAAGGEAKEMPCKHRFHDVCLLPWLEANSSCPVC	Nuclear	
TraesCS4D02G177900	H2	169	83	125	CVICLGGMEEGDEVRVLRCQHLFHRCCPDRWLAARPGATCPLC	Extracellular	
TraesCS4D02G187600	H2	84	23	76	CGICRMAFDGCCPDCKFPGDDCPIIWGACNHAFHLHCILKWVNSQTSIPLCPMC	Extracellular	
TraesCS4D02G187800	HC	875	529	570	CGICLSEDQRTTLQGVLNCCSHYFCFACIMEWSNVESRCPLC	Nuclear	
TraesCS4D02G192500	H2	250	198	238	CVICQMEYRRGDLQMALPCKHVYHASCVTKWLTINKVCPVC	Nuclear	Zinc finger, RING/FYVE/PHD-type
TraesCS4D02G192800	H2	418	138	179	CAVCLCEFADDDRLRLLPKCSHAFHVECIDTWLLSHSTCPLC	Plasma Membrane	
TraesCS4D02G200100	H2	414	165	207	CAVCLAEFADDGEKLRLLPGCRHVFHAACIDVWLAAHVTCPVC	Plasma Membrane	
TraesCS4D02G204100b	HC	304	214	255	CPSCKSNLTNTMSLVAISTCGHVFCKKCSDKFVATDKVCLMC	Extracellular	Nitric oxide synthase-interacting protein
TraesCS4D02G209300	H2	282	221	261	CSVCLEQVVAGDLLRSLPCLHRFHVNCIDPWLRQQGTCPIC	Extracellular	
TraesCS4D02G209500	H2	280	137	177	CAVCQEAFEPGAAAREMPCKHVYHQDCILPWLSLRNSCPIC	Nuclear	
TraesCS4D02G215600	HC	339	293	327	CRVCKSSEACMLLLPCRHLCLCKECESKLSFCPLC	Nuclear	E3 ubiquitin-protein ligase BOI-like
TraesCS4D02G221000	HC	405	327	365	CVICLSEPRDTTVLPCRHMCMCSECAKVLRYQTTRCPIC	Nuclear	

Identifiers	RING type	Protein size (amino acids)	Domain start	Domain end	RING domain	Localization	Description
TraesCS4D02G222000	HC	326	262	299	CFICREPFVDPVVTKCKHYFCEHCALKHHSKNKKCFVC	Nuclear	
TraesCS4D02G251200	HC	260	46	100	CPICCESFNIVENVPYVLWCGHTMCKNCILGLQWAVVKFPTLPIQLPLFVSCPWC	Plasma Membrane	
TraesCS4D02G255100	H2	276	227	267	CSICLDGFYDGDELIRLRCGHRFHSTCLEPWVRKCADCPYC	Nuclear	
TraesCS4D02G292000	HC	378	326	365	CVICLRKRRRAAFVPCGHLVCCCNCAKRVELMDEPLCPVC	Plasma Membrane	E3 Ubiquitin ligase, GIDE-type
TraesCS4D02G302600	H2	355	134	175	CAVCLTEFEDCDELRVLPACCHVFHPECIDPWLAGAVTCPLC	Plasma Membrane	
TraesCS4D02G313200	H2	263	109	150	CAVCLSDFAERERVRLLPGCSHAFHIDCIDTWLQGSARCPFC	Nuclear	
TraesCS4D02G313300	H2	284	114	155	CAVCLSEFAPGERLKMLPACSHAFHIDCIDTWLHHNVSCPLC	Plasma Membrane	
TraesCS4D02G318800	H2	208	117	158	CAICLSEFAGKEEIRVLPQCGHGFHVACVDAWLRAHSSCPSC	Extracellular	
TraesCS4D02G339200	H2	707	80	123	CAICLGSMRTGHGQALFTAECSHKFHFHCITSNVRHGNHICPIC	Nuclear	
TraesCS4D02G339300	H2	749	24	67	CAICLGGMAAGGGQATFTAECSHTFHFNCISASVAHGHLVCPLC	Nuclear	
TraesCS4D02G354300	HC	709	657	695	CCICCDSQIDSLLYRCGHMCTCSKCARELLRGAGKCPLC	Nuclear	
TraesCS4D02G362000	H2	91	31	71	CAVCLEDFQDGDELRTMPCSHSFHRCCIFRWLEISRVCPYC	Nuclear	
TraesCS5A02G010000	HC	236	25	65	CNICFELPQEPIVTLCGHLFCWPCLYKWLHIHSHSPECPVC	Extracellular	

Identifiers	RING type	Protein size (amino acids)	Domain start	Domain end	RING domain	Localization	Description
TraesCS5A02G027500	H2	143	85	126	CAVCLGEYDRGDVLRVLPDCAHSFHRPCV DQWLRLRPSCPVC	Chloroplast	
TraesCS5A02G028000	H2	152	99	140	CAVCLAEYADGDELRRLPGCRHAFHRRCV DEWLRRRPSCPLC	Extracellular	
TraesCS5A02G028200	H2	139	94	135	CAVCLAEYADGDELRVLPGCAHAFHRRCV DQWLRQRPSCPLC	Extracellular	
TraesCS5A02G028300	H2	163	103	144	CAVCLAQYADGDELRWLPGCRHAFHRPCV DEWLRRRPSCPLC	Extracellular	
TraesCS5A02G028400	H2	149	106	147	CAVCLAQYADGDELRWLPGCRHTFHRLCV DEWLRRRPSCPLC	Extracellular	
TraesCS5A02G028500	H2	160	102	143	CAVCLAEYADGDELRRLPGCKHAFHRLCV DEWLRRRPSCPLC	Extracellular	
TraesCS5A02G028600	H2	155	91	132	CAVCLAEYADGDELRVLPGCAHAFHRRCV DQWLRRRPSCPLC	Extracellular	
TraesCS5A02G028700	H2	151	98	139	CAVCLAEYADGDELRRLPGCRHAFHRRCV DEWLRRRPSCPLC	Extracellular	
TraesCS5A02G029200	H2	137	86	127	CAVCLADYADGDELRRLPGCRHVFHQRCV DDWLRQRTSCPLC	Extracellular	
TraesCS5A02G045900	H2	162	103	143	CSVCMEAFEEEDILRRLHCSHAFHEECISV WLGVSHLCPLC	Nuclear	
TraesCS5A02G049400	HC	228	37	84	CSICLETASEPVVTLCGHLYCWPCIFRWLTT SSRSKSRASSSARCPVC	Plasma Membrane	
TraesCS5A02G058000	HC	472	85	125	CQLCHRLLRNAATISECLHTFCRKCIYKKL NDEECDHCPVC	Nuclear	Zinc finger, RING/FYVE/PHD-type
TraesCS5A02G062400	H2	417	182	223	CAVCLLEFADGDELRALPLCAHAFHADCID VWLRAHASCPLC	Nuclear	

Identifiers	RING type	Protein size (amino acids)	Domain start	Domain end	RING domain	Localization	Description
TraesCS5A02G072600	H2	253	208	248	CSICLERCGEADGLMQLRCKHVFHSACLER WLRSHGDCPYC	Nuclear	
TraesCS5A02G095000	H2	210	86	128	CPICYEYLFDSLKGTRVLNCGHTMHMDCF AEMVDHNKYTCPIC	Extracellular	RCHY1, zinc-ribbon
TraesCS5A02G097000	H2	169	87	128	CGVCLEDFLEGGKKLRRMPCYHSFHQRCIF DWLRFNRRCPVC	Nuclear	
TraesCS5A02G129900	H2	247	110	151	CVVCLAEYEDKDVLRTLPYCGHNFHMVCI DAWLKQHSTCPVC	Plasma Membrane	
TraesCS5A02G135100	HC	738	239	285	CPICLESPLCPQITSCGHIYCFPCILHYLLMG KEDYRGECWKKCPLC	Nuclear	
TraesCS5A02G136900	H2	178	119	160	CAICLGEFDGAATLRRGSRCAHCFHAGCAE RWLRVSATCPVC	Extracellular	
TraesCS5A02G145800	H2	510	335	375	CPVCKDPMPLRTRAKQLPCMHLYHSSCILP WLSSRNTCPVC	Nuclear	
TraesCS5A02G147800	H2	209	150	192	CVICMEPYEAGGDRCRVLPACGHMFHRRC LRTWLRTSSTCPLC	Nuclear	
TraesCS5A02G147900	H2	147	85	127	CVICLAQYEGGERCSVLPGCGHVFHRGCV ATWLHTTHNTCPLC	Extracellular	
TraesCS5A02G167100	H2	162	70	110	CAVCTEEIAVADPVVRLPCAHWYHAGCIA PWLGIRSTCPMC	Plasma Membrane	
TraesCS5A02G183200	H2	413	337	377	CCICLTKYGDDDELRELPCNHLFHVQCVDK WLKINAVCPLC	Plasma Membrane	
TraesCS5A02G189000	HC	659	604	643	CIICKREEACVMLLQCAHQVLCVGCNKQH EEKGAVRCPSC	Nuclear	Zinc finger, RING/FYVE/PHD-type
TraesCS5A02G198500	H2	360	137	178	CAVCLSEFAADDRLRLLPLCGHAFHVACID TWLRSSSTCPLC	Extracellular	

Identifiers	RING type	Protein size (amino acids)	Domain start	Domain end	RING domain	Localization	Description
TraesCS5A02G207100	HC	455	397	431	CRICFERYICIVLLPCRHHVLCEPCSDKCQS CPIC	Plasma Membrane	Transmembrane Fragile-X-F-associated protein
TraesCS5A02G216000	H2	323	243	283	CAVCRENLVVDDKMQELPCKHLFHPPCLK PWLDENNSCPIC	Nuclear	Zinc finger, RING/FYVE/PHD-type
TraesCS5A02G228400	v	278	62	109	ECRICQEEDDIKNLESPCACTGSVKYAHRA CVQRWCNEKGDVTCEICH	Plasma Membrane	Protein of unknown function DUF3675
TraesCS5A02G232200	H2	448	330	370	CVVCTDPLPPSAPALRLPCGHLYHSHCIVR WLSEQNSCPIC	Nuclear	
TraesCS5A02G248100	H2	397	128	169	CAVCISEFDDDETLRLLPKCSHVFHPDCIDT WLASHVTCPVC	Plasma Membrane	
TraesCS5A02G248300	H2	356	157	198	CAVCLGEFSDGELVRLLPRCSHPFHAPCIDT WLRAHVSCPIC	Plasma Membrane	
TraesCS5A02G253700a	H2	164	96	137	CRVCLVRFEADAVVNRLPCGHLFHRACLE TWLDYDHATCPLC	Plasma Membrane	
TraesCS5A02G253700b	H2	164	95	138	DCRVCLVRFEADAVVNRLPCGHLFHRACL ETWLDYDHATCPLCR	Plasma Membrane	
TraesCS5A02G271600	H2	235	128	169	CSVCLEDLRAGELVRRLPACRHVFHVECID MWLHSHRSCPLC	Chloroplast	
TraesCS5A02G282300	HC	211	155	192	CPVCWNKLDEPATTMCGHIFCTNCIKQAIQ YQKKCPTC	Nuclear	Zinc finger, RING/FYVE/PHD-type
TraesCS5A02G283800	HC	190	142	179	CCVCMARRKGAAFVPCGHTFCRACAREVR AGRGRCPLC	Nuclear	
TraesCS5A02G308200	H2	185	120	161	CAICLSEFVDGEKVRVLPQCAHGFHVRCVD AWLVSNGSCPTC	Plasma Membrane	

Identifiers	RING type	Protein size (amino acids)	Domain start	Domain end	RING domain	Localization	Description
TraesCS5A02G308300	H2	177	112	153	CAICLSEFVDGKKVRVLPLCGHGFHVRCVNAWLVSHGSCTTC	Chloroplast	
TraesCS5A02G312800	H2	171	125	165	CVICKSSYKSLQKLIRLPCSHCYHANCITRWLKINKACPVC	Extracellular	
TraesCS5A02G325400	H2	337	285	325	CVICRVEFEEGESLVALPCNHSYHPDCINQWLQINKVCPMC	Nuclear	Zinc finger, RING/FYVE/PHD-type
TraesCS5A02G326800	H2	215	135	177	CAVCLAEYAAGDEVRVLPACRHGFHRECVDRWLLTRAPTCPVC	Cytoplasmic	
TraesCS5A02G326900	H2	181	97	139	CAVCLAEYAAGDEVRVLPACRHGFHRECVDRWLLTRAPTCPVC	Cytoplasmic	
TraesCS5A02G336400	H2	209	163	204	CPICLHEFQPGESARRLPACGHVFHLACIDSWLLWKPHCPMC	Plasma Membrane	Zinc finger, RING/FYVE/PHD-type
TraesCS5A02G354500	H2	190	110	151	CAICLAEFAGGDEARVLPTCGHGFHAACVDVWLLSNSTCPSC	Extracellular	
TraesCS5A02G354600	H2	199	111	152	CAICLAEFAPGDEVRVLPTCGHGFHAACVDVWLLSSSTCPSC	Extracellular	
TraesCS5A02G354700	H2	204	109	150	CAICLAEFTRGDEVRVLPHCGHGFHAACVDVWLLSSSTCPSC	Extracellular	
TraesCS5A02G354800	H2	192	111	152	CAICLAELARGDEVRVLPHCGHGFHAACVDVWLLSSSTCPSC	Extracellular	
TraesCS5A02G372500	H2	157	83	128	CAICLSEYGEGEAGELVRVVPACGHFYHASCEIDGWLRKRRTCPLC	Extracellular	
TraesCS5A02G372600	H2	152	78	123	CAICQWEYGEGDAGELVRVVPACGHFYHASCDVDRWLRKRHTCPLC	Extracellular	
TraesCS5A02G378600	HC	508	101	141	CPLCNRLLRDATTVSECLHTFCRKCIYKKFNDEEVESCPVC	Nuclear	

Identifiers	RING type	Protein size (amino acids)	Domain start	Domain end	RING domain	Localization	Description
TraesCS5A02G381800	HC	375	21	61	CPLCDGYFREASAFTECGHTFCRECIMKKIDEEEIEACPVC	Nuclear	Zinc finger, RING/FYVE/PHD-type
TraesCS5A02G396000	H2	247	88	129	CAVCITELAAGEAARLLPRCGHSFHVECVDMWLRSHSTCPVC	Plasma Membrane	
TraesCS5A02G426200	H2	202	135	176	CSVCLDNYGDGDVLRMLPDCGHLFHRECVDPWLRKHPTCPVC	Nuclear	
TraesCS5A02G443000	HC	403	41	81	CPLCRRLLRDAATITECLHTFCRKCISKEFIDKEICYCPTC	Nuclear	Zinc finger, RING/FYVE/PHD-type
TraesCS5A02G460200	HC	287	34	82	CPICLEGFEDEAYLDTCLHSFCYECITQWVSIVASKHEDPLSSVKCPLC	Nuclear	
TraesCS5A02G467700	H2	404	280	320	CTVCLEEFEMGTEAKEMPCQHKFHSNCILPWLELHSSCPIC	Nuclear	
TraesCS5A02G470400	H2	209	108	149	CAVCLAELADGDEARELPNCGHLFHLECVDAWLRTRTTCPLC	Plasma Membrane	
TraesCS5A02G476600	H2	84	23	76	CGICRMAFDGCCPDCKFPGDECPIIWGACNHAFHLHCILKWVNSQTSTALCPMC	Extracellular	
TraesCS5A02G494500	H2	209	117	158	CAICLSEFAEKEEIRVLPQCGHAFHVACVDAWLRAHSSCPSC	Extracellular	
TraesCS5A02G512800	H2	670	80	123	CAICLGSMRTGHGQALFTAECSHKFHFHCITSNVRHGNHICPIC	Nuclear	von Willebrand factor A-like domain superfamily
TraesCS5A02G513000	H2	740	10	53	CAICLGGMAAGGGQATFTAECSHTFHFNCISASVAHGHLVCPLC	Nuclear	
TraesCS5A02G529500	HC	718	666	704	CCICCDSQIDSLLYRCGHMCTCSKCARELLRGAGKCPLC	Nuclear	

Identifiers	RING type	Protein size (amino acids)	Domain start	Domain end	RING domain	Localization	Description
TraesCS5A02G547400a	G	477	57	103	CPACTEPWSSDGPHRMCCIPCGHVYGRSC METLLGRSGQNSAKCPQC	Cytoplasmic	
TraesCS5A02G547800	G	593	102	148	CPVCMEPWTSEGEHRISCIPCGHVYGRSCL ERWLTQRGNASATCPQC	Nuclear	
TraesCS5B02G007600	HC	234	25	65	CNICFELPQEPIVTLCGHLFCWPCLYKWLHI HSHSPECPVC	Extracellular	
TraesCS5B02G026700	H2	139	86	127	CAVCLGEYDRGDVLRVLPDCAHSFHRPCV DQWLRLRPSCPVC	Chloroplast	
TraesCS5B02G049900	H2	155	96	136	CSVCMEAFEEGEALRRMHCSHAFHEDCIS MWLSVSHLCPLC	Nuclear	
TraesCS5B02G054600	HC	256	67	114	CSICLETATEPVVTLCGHLYCWPCIFRWLTT SSKSKSRASSSARCPVC	Plasma Membrane	
TraesCS5B02G065600	HC	472	85	125	CQLCHRLLRNATTISECLHTFCRKCIYKKLN DEECDHCPVC	Nuclear	Zinc finger, RING/FYVE/PHD-type
TraesCS5B02G069700	H2	412	177	218	CAVCLLEFADGDELRALPLCAHAFHADCID VWLRAHASCPLC	Nuclear	
TraesCS5B02G078400	H2	274	224	264	CSICLERCGEADGLMQLRCKHVFHSACLER WLRSHGDCPYC	Nuclear	Zinc finger, RING/FYVE/PHD-type
TraesCS5B02G090000	H2	151	86	127	CAVCLEEFIDGGKKLRMMPCYHSFHQRCIF DWLRLNRICPVC	Cytoplasmic	
TraesCS5B02G090100	H2	169	87	128	CAVCLEDFVEGGKKLRRMPCYHSFHQRCIF DWLRFNRRCPVC	Nuclear	
TraesCS5B02G101200	H2	288	164	206	CPICYEYLFDSLKGTRVLNCGHTMHMDCF AEMVDHNKYTCPIC	Extracellular	RCHY1, zinc-ribbon
TraesCS5B02G111900	HC	241	198	230	CPVCLAKPRDMAFGCGHQTCSGCGPQVAD CPIC	Extracellular	Copine

Identifiers	RING type	Protein size (amino acids)	Domain start	Domain end	RING domain	Localization	Description
TraesCS5B02G129800	H2	275	140	181	CVVCLAEYEDKDVLRTLPYCGHNFHMVCI DAWLKQHSTCPVC	Plasma Membrane	
TraesCS5B02G133900	HC	710	210	256	CPICLESPLCPQITSCGHIYCFPCILHYLLMG KEDYRGECWKKCPLC	Nuclear	
TraesCS5B02G135600	H2	182	123	164	CAICLGEFHGVAALRRGSRCAHCFHAGCA ERWLRVSATCPVC	Extracellular	
TraesCS5B02G144700	H2	510	335	375	CPVCKDPMPIRTRAKQLPCMHLYHSSCILP WLSSRNTCPVC	Nuclear	
TraesCS5B02G164100	H2	238	147	187	CAVCTEEIAVADPVVRLPCAHWYHAGCIA PWLGIRSTCPMC	Plasma Membrane	
TraesCS5B02G181400	H2	415	339	379	CCICLTKYGDDDELRELPCNHLFHVQCVDK WLKINAVCPLC	Plasma Membrane	
TraesCS5B02G194700	HC	659	604	643	CIICKREEACVMLLQCAHQVLCVGCNKQH EEKGAVRCPSC	Nuclear	Zinc finger, RING/FYVE/PHD-type
TraesCS5B02G197200	H2	362	137	178	CAVCLSEFSPDDRLRLLPLCGHAFHVACID TWLRSSSTCPLC	Extracellular	
TraesCS5B02G205500	HC	455	397	431	CRICFERDICIVLLPCRHHVLCEPCSDKCQS CPIC	Plasma Membrane	Transmembrane Fragile-X-F-associated protein
TraesCS5B02G223400b	HC	392	346	388	CPKCKIYVERVDGCTFIACRCGHCFCYLCG ATMARTNHYCAKC	Nuclear	
TraesCS5B02G227200	v	278	62	109	ECRICQEEDDIKNLESPCACTGSVKYAHRA CVQRWCNEKGDVTCEICH	Plasma Membrane	Protein of unknown function DUF3675
TraesCS5B02G230500	H2	482	369	409	CVVCTDPLPPSAPARRLPCGHLYHSHCIVT WLSEHNSCPIC	Nuclear	
TraesCS5B02G245800	H2	396	128	169	CAVCISEFDDDETLRLLPKCSHVFHPDCIDT WLASHVTCPVC	Plasma Membrane	

Identifiers	RING type	Protein size (amino acids)	Domain start	Domain end	RING domain	Localization	Description
TraesCS5B02G246000	H2	354	155	196	CAVCLGEFSDGELVRLLPRCSHPFHAPCIDTWLRAHVSCPIC	Plasma Membrane	
TraesCS5B02G252600	H2	161	94	135	CRVCLVRFEADAVVNRLPCGHLFHRACLETWLDYDHATCPLC	Plasma Membrane	
TraesCS5B02G271900	H2	235	129	170	CSVCLEDLRAGETVRRLPACMHVFHVECIDMWLHSHRSCPLC	Plasma Membrane	
TraesCS5B02G281700	HC	335	279	316	CPVCWNKLDEPATTMCGHIFCTNCIKQAIQFQKKCPTC	Nuclear	Zinc finger, RING/FYVE/PHD-type
TraesCS5B02G282300	HC	196	148	185	CCVCMARRKGAAFVPCGHTFCRACAREVRAGRGRCPLC	Nuclear	
TraesCS5B02G308500	H2	182	118	159	CAICLSEFVDGEKVRVLPLCGHGFHVRCVDAWLVSHGSCPTC	Plasma Membrane	
TraesCS5B02G308600	H2	177	112	153	CAICLSEFVDGEKVRVLPLCGHGFHVRCVDAWLVSHGSCPTC	Plasma Membrane	
TraesCS5B02G308700	H2	182	110	151	CAICLSEFVDGEKVRVLPLCGHGFHVRCVDAWLVSHGSCPTC	Extracellular	
TraesCS5B02G313800	H2	171	125	165	CVICKSNYKSLQKLIRLPCSHCYHAGCITRWLKINKACPVC	Extracellular	
TraesCS5B02G325800	H2	378	326	366	CVICRVEFEEGESLVALPCNHSYHPDCINQWLQINKVCPMC	Nuclear	Zinc finger, RING/FYVE/PHD-type
TraesCS5B02G335400	H2	209	163	204	CPICLHEFQAGESARRLPACGHVFHLACIDSWLLWKPHCPMC	Plasma Membrane	Zinc finger, RING/FYVE/PHD-type
TraesCS5B02G356800	H2	195	113	154	CAICLAEFAPGNEVRVLPTCGHDFHAACVDVWLLSNSTCPSC	Extracellular	

Identifiers	RING type	Protein size (amino acids)	Domain start	Domain end	RING domain	Localization	Description
TraesCS5B02G356900	H2	200	112	153	CAICLVEFARGDEVRVLPTCGHGFHAACV DVWLLSSSTCPSC	Extracellular	
TraesCS5B02G357000	H2	189	113	154	CAICLAAFARGDEVRVLPRCGHGFHAACV DVWLLSSSTCPSC	Extracellular	Zinc finger, RING/FYVE/PHD-type
TraesCS5B02G373000	HC	377	273	321	CPICLDTLFNPYALSCGHLFCKGCACGAAS VYIFQGVRSAPPEAKCPVC	Nuclear	
TraesCS5B02G374600	H2	152	80	125	CAICLSEYGEGEAGELVRVVPACGHFYHAS CDVDRWLRKRRTCPLC	Extracellular	
TraesCS5B02G374700	H2	155	82	127	CAICLSEYGEGEAGELVRVVPACGHFYHAS CDVDRWLRKRRTCPLC	Extracellular	
TraesCS5B02G382100	HC	516	108	148	CPLCNRLLRDATTVSECLHTFCRKCIYKKF NDEEVESCPVC	Nuclear	
TraesCS5B02G385300	HC	400	21	61	CPLCDGYFREASAFTECGHTFCRECIMNKI DEEEIEACPVC	Nuclear	Zinc finger, RING/FYVE/PHD-type
TraesCS5B02G400800	H2	255	96	137	CAVCITELAAGEAARLLPRCGHSFHVECVD MWLRSHSTCPLC	Plasma Membrane	
TraesCS5B02G428100	H2	202	135	176	CSVCLDNYGDGDVLRMLPDCGHLFHRECV DPWLRKHPTCPVC	Nuclear	
TraesCS5B02G442500	H2	339	122	163	CAVCLLEFDDDDSLRLLPTCPHAFHPQCIGL WLEKHVTCPLC	Nuclear	
TraesCS5B02G447600	HC	391	39	79	CPLCRRLLRDAATITECLHTFCRKCISKEFID KEICYCPTC	Nuclear	Zinc finger, RING/FYVE/PHD-type
TraesCS5B02G469800	HC	312	59	107	CPICLEGFEDEAYLDTCLHSFCYECITQWVS IVASKHEEPLSNVKCPLC	Nuclear	

Identifiers	RING type	Protein size (amino acids)	Domain start	Domain end	RING domain	Localization	Description
TraesCS5B02G480100	H2	402	278	318	CTVCLEEFEMGTEAKEMPCQHKFHSNCILPWLELHSSCPIC	Nuclear	
TraesCS5B02G483000	H2	209	108	149	CAVCLAELADGDQARELPNCGHLFHLECVDAWLRTRTTCPLC	Plasma Membrane	
TraesCS5B02G489500	H2	84	23	76	CGICRMAFDGCCPDCKFPGDECPIIWGACNHAFHLHCILKWVNSQTSTPLCPMC	Extracellular	
TraesCS5B02G519400	HC	197	22	64	CSVCHDRFRIPCQANCSHWFCGECIIRVWNHGAAVQPCKCPIC	Plasma Membrane	Zinc finger, RING/FYVE/PHD-type
TraesCS5B02G522800	H2	199	83	123	CPICLDELEPGAHAAETPCQHVYHPACLATWLGARGTCPVC	Extracellular	
TraesCS5B02G548200	H2	311	103	144	CTVCLEEYEAKDVVRVLPYCGHAFHAACIDTWLRQHPTCPIC	Nuclear	Zinc finger, RING/FYVE/PHD-type
TraesCS5B02G555800	H2	1919	1869	1915	CPICYSILHTSNHGLPRLACKTCKHKFHGACLYKWFSTSNKSTCPLC	Plasma Membrane	Listerin, zinc finger, RING-type
TraesCS5B02G557600	H2	166	90	140	CPICMEDFVRDDSDTNDDSDTTVRVRLPCSHSFHCDCILPWFYKVAKCPKC	Extracellular	
TraesCS5B02G558000	H2	260	196	234	CRVCVEAKEDTMARLPCSHSFHRGCILPWFDKVATCPMC	Mitochondrial	
TraesCS5D02G014800	HC	237	26	66	CNICFELPQEPIVTLCGHLFCWPCLYKWLHIHSHSPECPVC	Extracellular	
TraesCS5D02G035300	H2	144	86	127	CAVCLGEYDRGDVLRVLPDCAHSFHRPCVDQWLRLRPSCPVC	Plasma Membrane	
TraesCS5D02G035700	H2	153	100	141	CSVCLAEYADGDELRRLPGCRHAFHRRCVDEWLRRRPSCPLC	Extracellular	

Identifiers	RING type	Protein size (amino acids)	Domain start	Domain end	RING domain	Localization	Description
TraesCS5D02G035800	H2	121	69	110	CAVCLAEYADGDELRVLPGCAHVFHRRCV DQWLRRRPSCPLC	Extracellular	Zinc finger, RING/FYVE/PHD-type
TraesCS5D02G035900	H2	154	95	136	CAVCLAEYADGDELRRLPGCKHAFHRLCV DEWLRRRPSCPLC	Extracellular	
TraesCS5D02G036200	H2	135	83	124	CAVCLAEYADGDELRVLPGCAHAFHRRCV DQWLRQRSSCPLC	Extracellular	
TraesCS5D02G036300	H2	165	106	147	CAVCLAEYADGDELRWLPGCKHAFHRLCV DEWLRRRPSCPLC	Extracellular	
TraesCS5D02G036400	H2	158	94	135	CAVCLAEYADGDELRVMPGCAHTFHRRCV DQWLRRRPSCPLC	Extracellular	
TraesCS5D02G036500	H2	152	99	140	CSVCLGEYANGDELRRLPGCRHAFHRRCV DEWLRRRPSCPLC	Extracellular	
TraesCS5D02G055800	H2	156	97	137	CSVCMEAFEEGEMLRGLECSHAFHEDCISV WLSVSHLCPLC	Mitochondrial	
TraesCS5D02G060200	HC	228	37	84	CSICLEVASEPVVTLCGHLYCWPCIFRWLT TSSKSKSRASPSARCPVC	Plasma Membrane	
TraesCS5D02G069800	HC	472	85	125	CQLCHRLLRNATTISECLHTFCRKCIYKKLN DEECDHCPVC	Nuclear	Zinc finger, RING/FYVE/PHD-type
TraesCS5D02G073600	H2	417	182	223	CAVCLLEFADGDELRALPLCAHAFHADCID VWLRAHASCPLC	Nuclear	
TraesCS5D02G085300	H2	275	225	265	CSICLERCGEADGLMQLRCKHVFHSACLER WLRSHGDCPYC	Nuclear	Zinc finger, RING/FYVE/PHD-type
TraesCS5D02G096000	H2	150	86	127	CAVCLEEFVDGGKKLRMMPCYHSFHQRCI FDWLRLNRICPVC	Cytoplasmic	

Identifiers	RING type	Protein size (amino acids)	Domain start	Domain end	RING domain	Localization	Description
TraesCS5D02G096500	H2	170	87	129	CAVCLEDFVEGGKKLRRMPCCYHSFHQRC IFDWLRFNRRCPVC	Nuclear	
TraesCS5D02G096600	H2	169	87	128	CAVCLEDFVEGGKKLRRMPCYHSFHQRCIF DWLRFNRRCPVC	Nuclear	
TraesCS5D02G107600	H2	288	164	206	CPICYEYLFDSLKGTRVLNCGHTMHMDCF AEMVDHNKYTCPIC	Extracellular	RCHY1, zinc-ribbon
TraesCS5D02G122000	HC	537	494	526	CPVCLAKPRDMAFGCGHQTCSGCGPQVVD CPIC	Nuclear	Copine
TraesCS5D02G137400	H2	245	110	151	CVVCLAEYEDKDVLRTLPYCGHNFHMVCI DAWLKQHSTCPVC	Plasma Membrane	
TraesCS5D02G139400	HC	708	208	254	CPICLESPLCPQITSCGHIYCFPCILHYLLMG KEDYRGECWKKCPLC	Nuclear	
TraesCS5D02G144900	H2	150	88	130	CVICLAQYEGGERCSVLPGCGHVFHRGCV ATWLHTTHNTCPLC	Extracellular	
TraesCS5D02G146400	H2	508	333	373	CPVCKDPMPIRTRAKQLPCMHLYHLSCILP WLSSRNTCPVC	Nuclear	
TraesCS5D02G171400	H2	162	70	110	CAVCTEEIAVADPVVRLPCAHWYHAGCIA PWLGIRSTCPMC	Plasma Membrane	
TraesCS5D02G188300	H2	413	337	377	CCICLTKYGDDDELRELPCNHLFHVQCVDK WLKINAVCPLC	Plasma Membrane	
TraesCS5D02G202000	HC	659	604	643	CIICKREEACVMLLQCAHQVLCVGCNKQH EEKGAVRCPSC	Nuclear	Zinc finger, RING/FYVE/PHD-type
TraesCS5D02G204500	H2	358	137	178	CAVCLNEFAADDRLRLLPLCGHAFHVACID TWLRSSSTCPLC	Extracellular	
TraesCS5D02G213300	HC	373	315	349	CRICFERDICIVLLPCRHHVLCEPCSDKCQS CPIC	Plasma Membrane	Transmembrane Fragile-X-F-associated protein

Identifiers	RING type	Protein size (amino acids)	Domain start	Domain end	RING domain	Localization	Description
TraesCS5D02G223800	H2	323	243	283	CAVCRENLVVDDKMQELPCKHLFHPPCLK PWLDENNSCPIC	Nuclear	Zinc finger, RING/FYVE/PHD-type
TraesCS5D02G235800	H2	487	371	411	CVVCTDPLPPSAPVRRLPCGHLYHSHCIVR WLSEQNSCPIC	Nuclear	
TraesCS5D02G239200	v	278	62	109	ECRICQEEDDIKNLESPCACTGSVKYAHRA CVQRWCNEKGDVTCEICH	Plasma Membrane	Protein of unknown function DUF3675
TraesCS5D02G254900	H2	392	126	167	CAVCLSEFDDDETLRLLPKCSHVFHPDCIDT WLASHVTCPVC	Plasma Membrane	
TraesCS5D02G255200	H2	355	156	197	CAVCLGEFSDGELVRLLPRCSHPFHAPCIDT WLRAHVSCPIC	Plasma Membrane	
TraesCS5D02G279300	H2	308	204	245	CSVCLEDLRAGELVRRLPACRHVFHVECID MWLHSHRSCPLC	Nuclear	
TraesCS5D02G289400	HC	202	146	183	CPVCWNKLDEPATTMCGHIFCTNCIKQAIQ FQKKCPTC	Nuclear	Zinc finger, RING/FYVE/PHD-type
TraesCS5D02G291000	HC	200	152	189	CCVCMARRKGAAFVPCGHTFCRACAREVR AGRGRCPLC	Nuclear	
TraesCS5D02G315200	H2	182	118	159	CAICLSEFVDGEKVRVLPLCGHGFHVRCVD TWLVSHGSCPTC	Plasma Membrane	
TraesCS5D02G315300	H2	189	118	159	CAICLSEFVDGEKVRVLPLCRHGFHVRCVD AWLVSHGSCPTC	Plasma Membrane	
TraesCS5D02G319600	H2	171	125	165	CVICKSNYKSLQKLIRLPCSHCYHAGCITR WLKINKACPVC	Extracellular	
TraesCS5D02G331600	H2	340	288	328	CVICRVEFEEGESLVALPCNHSYHPDCINQ WLQINKVCPMC	Nuclear	Zinc finger, RING/FYVE/PHD-type

Identifiers	RING type	Protein size (amino acids)	Domain start	Domain end	RING domain	Localization	Description
TraesCS5D02G333100	H2	214	134	176	CAVCLAEYAAGDEVRVLPACRHGFHRECV DRWLLTRAPTCPVC	Cytoplasmic	
TraesCS5D02G333200	H2	211	127	169	CAVCLAEYAAGDEVRVLPACRHGFHRECV DRWLLTRAPTCPVC	Extracellular	
TraesCS5D02G341100	H2	209	163	204	CPICLHEFQAGESSRRLPACGHVFHLACIDS WLLWKPHCPMC	Plasma Membrane	Zinc finger, RING/FYVE/PHD-type
TraesCS5D02G361800	H2	193	113	154	CAICLAEFAPGDEVRVLPTCGHGFHAACVD VWLLSNSTCPSC	Extracellular	
TraesCS5D02G361900	H2	200	112	153	CAICLAEFAGGDEVRVLPTCGHGFHAACV DVWLLSSSTCPSC	Extracellular	
TraesCS5D02G362000	H2	207	108	149	CAICLAEFTRGDEVRVLPTCGHGFHAACVD VWLLSSSTCPSC	Extracellular	
TraesCS5D02G362100	H2	187	111	152	CAICLAEFARGDEVRVLPHCGHGFHAACV DVWLLSSSTCPSC	Extracellular	
TraesCS5D02G380200	HC	340	236	284	CPICLDTLFNPYALSCGHLFCKGCACGAAS VYIFQGVRSAPPEAKCPVC	Nuclear	
TraesCS5D02G382000	H2	149	78	123	CAICLSEYGEGEAGELVRVVPACGHFYHAS CDVDKWLRKRRTCPLC	Extracellular	
TraesCS5D02G382100	H2	159	89	134	CALCLSDYGEGDAGELVRVVPACGHFYHA SCGVDKWLRKHRTCPLC	Extracellular	
TraesCS5D02G382200	H2	213	146	190	CAFCQSEYAKAPEEVVRVVPACGHFFHAA CDADRWIRARRTCPLC	Mitochondrial	
TraesCS5D02G388500	HC	520	112	152	CPLCNRLLRDATTVSECLHTFCRKCIYKKF NDEEVESCPVC	Nuclear	
TraesCS5D02G391100	HC	375	21	61	CPLCDGYFREASAFTECGHTFCRECIMKKI DEEEIEACPVC	Nuclear	Zinc finger, RING/FYVE/PHD-type

Identifiers	RING type	Protein size (amino acids)	Domain start	Domain end	RING domain	Localization	Description
TraesCS5D02G405300	H2	255	95	136	CAVCITELAAGDAARLLPRCGHSFHVECVDMWLRSHSTCPLC	Plasma Membrane	
TraesCS5D02G434200	H2	202	135	176	CSVCLDNYGDGDVLRMLPDCGHLFHRECVDPWLRKHPTCPVC	Nuclear	
TraesCS5D02G446700	H2	338	122	163	CAVCLLEFDDDDSLRLLPTCPHAFHPQCIGLWLEKHVTCPLC	Nuclear	
TraesCS5D02G450400	HC	399	43	83	CPLCRRLLRDAATITECLHTFCRKCISKEFIDKEICYCPTC	Nuclear	Zinc finger, RING/FYVE/PHD-type
TraesCS5D02G471300	HC	285	32	80	CPICLEGFEDEAYLDTCLHSFCYECITQWVSIVASKHEDPLSSVKCPLC	Nuclear	
TraesCS5D02G472700	H2	600	9	45	CAVCHGDMGRGHATFTAECSHSFHLRCVHHQIACPLC	Chloroplast	
TraesCS5D02G480800	H2	404	280	320	CTVCLEEFEMGTEAKEMPCQHKFHSNCILPWLELHSSCPIC	Nuclear	
TraesCS5D02G483100	H2	208	108	149	CAVCLAELADGDEARELPNCGHLFHLECVDAWLRTRTTCPLC	Plasma Membrane	
TraesCS5D02G489900	H2	84	23	76	CGICRMAFDGCCPDCKFPGDECPIIWGACNHAFHLHCILKWVNSQTSTPLCPMC	Extracellular	
TraesCS5D02G518700	HC	166	22	64	CSVCHDRFRIPCQANCSHWFCGECIIRVWNHGAAVQPCKCPIC	Extracellular	Zinc finger, RING/FYVE/PHD-type
TraesCS5D02G521500	H2	217	83	123	CPICLDELEPGAHAAETPCQHVYHPACLATWLGARGTCPVC	Cytoplasmic	
TraesCS5D02G535300	H2	313	103	144	CTVCLEEYEAKDVVRVLPYCGHAFHAACIDTWLRQHPTCPIC	Nuclear	Zinc finger, RING/FYVE/PHD-type

Identifiers	RING type	Protein size (amino acids)	Domain start	Domain end	RING domain	Localization	Description
TraesCS5D02G551700	H2	256	187	230	CCVCMEDFVAPRDSDTTVRLPCSHAFHRA CILPWLYKASTCPKC	Mitochondrial	
TraesCS5D02G553700	H2	1760	1710	1756	CPICYSILHTSNHGLPRLACKTCKHKFHGA CLYKWFSTSNKSTCPLC	Plasma Membrane	Listerin, zinc finger, RING-type
TraesCS5D02G564400	H2	253	189	227	CRVCVEAKEDAMVRLPCSHSFHRGCILPWF DKVATCPMC	Mitochondrial	
TraesCS6A02G001000	v	440	194	252	VCRICMVALLEEEEEEEGGAGDGVLKLECR CKGELALAHRRCALKWFGIKGNPNCDVCG	Plasma Membrane	Zinc finger, RING/FYVE/PHD-type
TraesCS6A02G023700	H2	162	111	158	CAICLAEFADGDAVRAMPACGHGFHACCI ERWLAVPASAARRPSCPTC	Nuclear	
TraesCS6A02G050800	H2	372	195	236	CAVCLAEFRGGETLRLLPRCAHAFHRGCID TWLRAHVNCPLC	Nuclear	
TraesCS6A02G055100	H2	224	125	166	CAVCLAGLERGEEARALPRCGHRFHVGCID AWFRGNSTCPLC	Plasma Membrane	
TraesCS6A02G084400	HC	351	304	338	CKWCGAKEAAVLVMPCRHLCLCADCDRV TDACPVC	Nuclear	E3 ubiquitin-protein ligase BOI-like
TraesCS6A02G095500	H2	220	151	192	CSLCLEDLEDGEMVRQLPSCKHIFHVDCID MWLHSHTTCIIC	Plasma Membrane	
TraesCS6A02G095600	H2	183	118	159	CSVCLEDIGDGEMVRQLPTCKHLFHVGCID MWLHSHTTCPLC	Extracellular	
TraesCS6A02G096200	v	174	84	133	QCRICLDNEGDDLIAPCNCKGTQKYVHRSC LDNWRSTKEGFAFSHCTECR	Extracellular	
TraesCS6A02G117900	HC	1029	794	833	CPICLEAFEDAVLTPCAHRLCRECILSSWQS PAAGLCPVC	Nuclear	SNF2-related, N-terminal domain
TraesCS6A02G120700	HC	263	216	250	CKGCGENAPVVVLLPCRHLSICGPCAEVAS GCPSC	Extracellular	E3 ubiquitin-protein ligase BOI-like

Identifiers	RING type	Protein size (amino acids)	Domain start	Domain end	RING domain	Localization	Description
TraesCS6A02G121400	H2	582	533	573	CSICQEEFVKGEEVGRLRCEHQYHVCCIRQ WLLQKNWCPVC	Nuclear	
TraesCS6A02G122800	H2	406	138	179	CAVCLSEFDDDDTLRLLPKCSHAFHADCID AWLASHVTCPVC	Plasma Membrane	
TraesCS6A02G145300	H2	327	175	214	CPVCLERLDQDPGGILTTICNHSFHCSCISK WTDSSCPVC	Extracellular	BRCA1-associated 2
TraesCS6A02G172100	H2	201	140	181	CAVCLGEVEKGEAVRRMPVCLHVFHTECI DRWLRSHATCPIC	Extracellular	
TraesCS6A02G202400	H2	156	56	93	CIICCEETTTAKKLLCGHLFHVHCLRSWRE RQNTCPTC	Extracellular	
TraesCS6A02G226100a	HC	451	97	136	CCVCLELLYKPVVIACGHMSCFWCVHKAM HYARESHCAIC	Nuclear	
TraesCS6A02G226100b	HC	451	236	273	CPICKELLYQPAVLNCGHVYCISCLPSVGDE ALKCQVC	Nuclear	
TraesCS6A02G227300	H2	711	83	126	CAICFDSMKPGHGQALFTAECSHMFHFHCI SSSVKHGNYVCPVC	Nuclear	
TraesCS6A02G236100	HC	242	32	79	CNICLDFAVEPVVTLCGHLYCWPCIYEWLR RDAAGGTISAAGRPCPVC	Plasma Membrane	
TraesCS6A02G239000	H2	181	128	169	CAICLSELADGERVRVLPACGHGFHGACV DGWLAARASCPTC	Nuclear	
TraesCS6A02G255700	H2	373	317	357	CCICLSVYDDGAELRELPCGHHFHCACIDK WLHINATCPLC	Plasma Membrane	
TraesCS6A02G256500	H2	208	104	145	CSICLGEYDEKEILRIMPTCRHNFHLSCIDIW LEKQTTCPIC	Plasma Membrane	Zinc finger, RING/FYVE/PHD-type
TraesCS6A02G258800	H2	207	114	156	CAVCLCGIGGCHEVRRLPNCRHVFHRGCID RWMGHGQRTCPLC	Plasma Membrane	

Identifiers	RING type	Protein size (amino acids)	Domain start	Domain end	RING domain	Localization	Description
TraesCS6A02G259200	H2	153	89	131	CIVCLERLRAADEVRRLGNCAHAFHRGCID GWIDLGRTTCPLC	Extracellular	
TraesCS6A02G261400	H2	170	94	135	CAICLAEFEDGQHMRVLPQCSHAFHAACV DAWLRSHSSCPSC	Extracellular	
TraesCS6A02G262500	H2	364	140	181	CAVCLSPFVPDAELRLLPACRHAFHAACVD AWLRNTPSCPLC	Nuclear	
TraesCS6A02G264300	H2	391	168	209	CSVCLGEFRDGESLRLLPKCSHAFHLLCIDT WLKSHSNCPLC	Nuclear	
TraesCS6A02G274400	H2	116	50	105	CAICRNHIMDLCIECQANQASATSEECTVA WGVCNHAFHFHCISRWLKTRQVCPLD	Extracellular	
TraesCS6A02G288800	H2	307	231	271	CAVCKDGIEAGEGARRLPCAHLYHDACILP WLAIRNTCPLC	Chloroplast	
TraesCS6A02G290900	H2	156	81	122	CAVCLEAFQGGDRCRVLPGCHHGFHAQCV DSWLRQSRRCPVC	Extracellular	
TraesCS6A02G291000	H2	127	67	108	CAVCLEAFMAGDRCMVLPRCEHGFHAECV SSWLRKSRLCPIC	Extracellular	
TraesCS6A02G298300	H2	363	313	353	CCICLSSYEDGVELSALPCNHHFHSTCITKW LRMHANCPLC	Plasma Membrane	
TraesCS6A02G304300	HC	395	159	197	CAICLDVCFQPSTTPCGHSFCMQCLKHAAS KCGKRCPKC	Nuclear	
TraesCS6A02G308200	H2	226	181	222	CAICLQDMVGGETARRLPNCSHAFHQLCV DKWLTSHGSCPVC	Extracellular	Zinc finger, RING/FYVE/PHD-type
TraesCS6A02G315600	H2	405	140	181	CAVCLSEFEDEDRLRLLPKCSHAFHPDCIGE WLASHVTCPVC	Nuclear	

Identifiers	RING type	Protein size (amino acids)	Domain start	Domain end	RING domain	Localization	Description
TraesCS6A02G326100	HC	700	77	114	CPICMALIKDAFLTACGHSFCYMCIVTHLNHKSDCPCC	Nuclear	WD4/YVTN repeat-like-containing domain superfamily
TraesCS6A02G330700	H2	326	202	242	CPVCKEEFELGEAARQLPCKHVYHSDCIVPWLRLHNSCPVC	Nuclear	
TraesCS6A02G346000	H2	401	330	370	CCICLAKYAHNEELRELPCTHCFHKECVDKWLKINALCPLC	Plasma Membrane	
TraesCS6A02G349700	HC	252	196	233	CPVCLNKLVEPSTTKCGHIFCAECIKQAIQFQKKCPTC	Nuclear	Zinc finger, RING/FYVE/PHD-type
TraesCS6A02G352200	H2	449	116	157	CAVCLCEFAPDDQLRLLPKCSHAFHLECIDTWLLSHSTCPLC	Nuclear	
TraesCS6A02G361600	H2	203	151	191	CAVCLEEFRAGDVLAHLPCAHRFHWACAVPWVQAASRCPFC	Nuclear	
TraesCS6A02G362000	H2	530	478	519	CHICLTEYEDGDQIRSLPCKHEFHLQCVDKWLKEIHRVCPLC	Nuclear	
TraesCS6A02G375500	H2	735	96	139	CAICLTIMKPGQGHALFTAECSHTFHFHCISANVKHGSNNCPVC	Nuclear	von Willebrand factor A-like domain superfamily
TraesCS6A02G397900	H2	285	169	210	CAVCLGEFQEGERVRLLPGCLHVFHAECIDTWLHDCANCPLC	Extracellular	
TraesCS6A02G399600	H2	171	120	160	CPICLEEYDEENPRSITKCEHHFHLCCILEWIERSETCPVC	Nuclear	
TraesCS6A02G405400	HC	766	714	752	CCVCCDTQIDSLLYRCGHMCTCSKCANELVRSGGKCPLC	Nuclear	
TraesCS6A02G420200	H2	174	81	122	CAICLADLAAGEELRVLPRCGHSFHVACVDAWLRAHATCPSC	Extracellular	

Identifiers	RING type	Protein size (amino acids)	Domain start	Domain end	RING domain	Localization	Description
TraesCS6B02G005200	v	456	211	266	VCRICMVALLEEEEEGAGDGVLKLECRCK GELALAHRRCALKWFGIKGDPNCDVCG	Nuclear	Zinc finger, RING-CH-type
TraesCS6B02G032400	H2	174	117	166	CAICLAEFADGDAVRAMPSCGHGFHARCIE RWLAVPAPAAGSRRPSCPTC	Nuclear	
TraesCS6B02G067200	H2	371	195	236	CAVCLAEFRGGETLRLLPRCGHAFHRGCID TWLRAHVNCPLC	Nuclear	
TraesCS6B02G118100	HC	349	302	336	CRWCGAKEAAVLVMPCRHLCLCADCDRV ADACPVC	Nuclear	E3 ubiquitin-protein ligase BOI-like
TraesCS6B02G123500	H2	187	118	159	CSVCLEDLEDGEMVRELPSCKHIFHVECID MWLHSHTTCPIC	Extracellular	
TraesCS6B02G123700	H2	183	118	159	CSVCLEDLGDGEMVRQLPTCKHLFHVRCID MWLHSHTTCPLC	Extracellular	
TraesCS6B02G124600	v	174	84	133	QCRICLDNEGDDLIAPCNCKGTQKYVHRSC LDNWRSTKEGFAFSHCTECR	Extracellular	
TraesCS6B02G146100	HC	1027	792	831	CPICLEAFEDAVLTPCAHRLCRECILSSWQS PAAGLCPVC	Nuclear	SNF2-related, N-terminal domain
TraesCS6B02G149100	HC	263	216	250	CKACGENAPVVVLLPCRHLSVCGPCAEAA SGCPSC	Nuclear	E3 ubiquitin-protein ligase BOI-like
TraesCS6B02G149700	H2	563	514	554	CSICQEEFVKSEEVGRLPCEHQYHVCCIRQ WLLQKNWCPVC	Nuclear	
TraesCS6B02G151000	H2	404	137	178	CAVCLSEFDDDDTLRLLPKCSHAFHADCID AWLASHVTCPVC	Plasma Membrane	
TraesCS6B02G164200	H2	123	71	111	CHVCHDDFEEGDKLRAMPCAHSFHQRCIF RALSVNRLCPVC	Nuclear	

Identifiers	RING type	Protein size (amino acids)	Domain start	Domain end	RING domain	Localization	Description
TraesCS6B02G173500	H2	322	170	209	CPVCLERLDQDPGGILTTICNHSFHCSCISK WTDSSCPVC	Extracellular	BRCA1-associated 2
TraesCS6B02G178500	H2	181	131	172	CPVCLDAARAGERWRALPACGHAFHAAC VDRWLLLSPGCPVC	Plasma Membrane	
TraesCS6B02G198800	H2	401	264	304	CAVCKDVFASGQRVVFLPCKHYFHGDCILP WLAMRSTCPVC	Nuclear	
TraesCS6B02G254400	H2	710	82	125	CAICFDSMKPGHGQALFTAECSHMFHFHCI SSSVKHGNYVCPVC	Nuclear	
TraesCS6B02G255500a	HC	392	38	77	CCVCLELLYKPVVIACGHMSCFWCVHKAM HYARESHCAIC	Nuclear	
TraesCS6B02G255500b	HC	392	177	214	CPICKELLYQPAVLNCGHVYCISCLPSVGDE ALKCQVC	Nuclear	
TraesCS6B02G264500	HC	241	31	78	CNICLDFAVEPVVTLCGHLYCWPCIYEWLR RDAAGGTISTAGRPCPVC	Plasma Membrane	
TraesCS6B02G266600	H2	203	110	152	CAVCLCGIGGGDEVRRLSNCRHVFHRGCID RWMGHQQRTCPLC	Extracellular	
TraesCS6B02G268900	H2	208	104	145	CSICLGEYDEKEILRIMPTCRHNFHLSCIDIW LEKQTTCPIC	Plasma Membrane	Zinc finger, RING/FYVE/PHD-type
TraesCS6B02G269900	H2	373	317	357	CCICLSVYDDGAELRELPCGHHFHCACIDK WLHINATCPLC	Plasma Membrane	
TraesCS6B02G274100	HC	869	817	855	CGVCRDRQKEVVITKCYHLFCNDCIQKLLR NRQRRCPSC	Nuclear	
TraesCS6B02G286500	H2	154	90	132	CIVCLERLWATDEVRRLGNCAHAFHRGCID GWIDLGRTTCPLC	Extracellular	
TraesCS6B02G288700	H2	169	93	134	CAICLAEFEDGQDMRVLPQCSHAFHTACV DAWLRSHSSCPSC	Extracellular	

Identifiers	RING type	Protein size (amino acids)	Domain start	Domain end	RING domain	Localization	Description
TraesCS6B02G289700	H2	364	143	184	CAVCLSPFVPDAELRLLPACRHAFHAACVDAWLRNTPSCPLC	Nuclear	
TraesCS6B02G291300	H2	390	169	210	CSVCLGEFQDGESLRLLPKCSHAFHLPCIDTWLKSHSNCPLC	Extracellular	
TraesCS6B02G296000	HC	325	261	298	CFICREPFVDPVVTKCKHYFCEHCALKHHSKNKKCFVC	Nuclear	
TraesCS6B02G301900	H2	116	50	105	CAICRNHIMDLCIECQANQASATSEECTVAWGVCNHAFHFHCISRWLKTRQVCPLD	Extracellular	
TraesCS6B02G310500	HC	368	206	263	CSVCLDRVLSKPTAAEKRFGLLPECDHAFCITCIRKWRSSSLTSSMDIDSTVKACPIC	Extracellular	
TraesCS6B02G318600	H2	307	228	268	CAVCKDGIEDGEGARRLPCAHLYHDACILPWLAIRNTCPLC	Chloroplast	
TraesCS6B02G321500	H2	154	80	121	CAVCLEAFQGGDRCRVLPGCHHGFHAQCVDSWLRQSRWCPVC	Extracellular	
TraesCS6B02G327800	H2	367	317	357	CCICLSSYEDGVELSALPCNHHFHSTCITKWLRMHANCPLC	Plasma Membrane	
TraesCS6B02G332400	HC	393	155	193	CAICLDICYQPSTTPCGHSFCMQCLKHAASKCGKRCPKC	Nuclear	Zinc finger, RING/FYVE/PHD-type
TraesCS6B02G336900	H2	228	183	224	CAICLQDMVAGETARRLPTCSHAFHQLCVDKWLISHGSCPVC	Extracellular	Zinc finger, RING/FYVE/PHD-type
TraesCS6B02G346000	H2	402	140	181	CAVCLSEFEDEDRLRLLPKCSHAFHPECIGVWLASHVTCPVC	Nuclear	

Identifiers	RING type	Protein size (amino acids)	Domain start	Domain end	RING domain	Localization	Description
TraesCS6B02G356400	HC	693	70	107	CPICMGLIKDAFLTACGHSFCYMCIVTHLN HKSDCPCC	Nuclear	WD4/YVTN repeat-like-containing domain superfamily
TraesCS6B02G361600	H2	337	199	239	CPVCKEEFELGEAARQLPCKHVYHSDCIVP WLCLHNSCPVC	Nuclear	
TraesCS6B02G378600	H2	402	334	374	CCICLAKYAHNEELRELPCTHCFHKECVDK WLKINALCPLC	Plasma Membrane	
TraesCS6B02G379400	H2	402	334	374	CCICLAKYAHNEELRELPCTHCFHKECVDK WLKINALCPLC	Plasma Membrane	
TraesCS6B02G382800	HC	252	196	233	CPVCLNKLVEPSTTKCGHIFCAECIKQAIQF QKKCPTC	Nuclear	Zinc finger, RING/FYVE/PHD-type
TraesCS6B02G385100	H2	451	119	160	CAVCLCEFAPDDQLRLLPKCSHAFHLECID TWLLSHSTCPLC	Nuclear	
TraesCS6B02G395000	H2	203	151	191	CAVCLEEFRGGDVLAHLPCAHRFHWACAV PWVQAASRCPFC	Nuclear	
TraesCS6B02G413100	H2	739	100	143	CAICLTIMKPGQGHALFTAECSHTFHFHCIS ANVKHGSNNCPVC	Nuclear	von Willebrand factor A-like domain superfamily
TraesCS6B02G437700	H2	282	171	212	CAVCLGEFQEGERVRLLPGCLHVFHAECID TWLHGCASCPLC	Extracellular	
TraesCS6B02G439800	H2	171	120	160	CPICLEEYDEENPRSITKCEHHFHLCCILEW MERSETCPVC	Nuclear	
TraesCS6B02G449700	H2	199	115	163	CAVCLTELQLRESVKAIPACGHVFHPPCIDR WLQSLAAAGAGHASCPLC	Nuclear	
TraesCS6B02G449800	HC	767	715	753	CCVCCDTQIDSLLYRCGHMCTCSKCANEL VRSGGKCPLC	Nuclear	

Identifiers	RING type	Protein size (amino acids)	Domain start	Domain end	RING domain	Localization	Description
TraesCS6B02G467800	H2	204	109	150	CAICLADLVAGEELRVLPRCGHSFHVACVD AWLRTCATCPSC	Plasma Membrane	
TraesCS6D02G005100	v	467	227	287	VCRICMVALLEEEEEEGGAAGAGDGVLKL ECRCKGELALAHRRCALKWFGIKGNPNCD VCG	Plasma Membrane	Zinc finger, RING/FYVE/PHD-type
TraesCS6D02G027000	H2	174	118	167	CAICLAEFAEGDAVRAMPACGHGFHARCIE RWLAVPTSAAGSRRPSCPTC	Nuclear	
TraesCS6D02G039100	H2	358	151	194	CCICAQEFAPTDMILALPACLHKFHERCIIP WIRGHAPYCCPFC	Plasma Membrane	
TraesCS6D02G086300	H2	184	119	160	CSVCLEDLGDGEMVRQLPTCKHLFHVGCI DMWLHSHTTCPLC	Extracellular	
TraesCS6D02G087300	v	272	84	133	QCRICLDNEGDDLIAPCNCKGTQKYVHRSC LDNWRSTKEGFAFSHCTECR	Plasma Membrane	
TraesCS6D02G107900	HC	1027	792	831	CPICLEAFEDAVLTPCAHRLCRECILSSWQS PSAGLCPVC	Nuclear	SNF2-related, N-terminal domain
TraesCS6D02G110700	HC	263	216	250	CKGCGENTPVAVLLPCRHLSVCGTCAEALS GCPSC	Extracellular	E3 ubiquitin-protein ligase BOI-like
TraesCS6D02G111500	H2	581	532	572	CSICQEEFVKGEEVGRLRCEHQYHVCCIRQ WLLQKNWCPVC	Nuclear	
TraesCS6D02G113000	H2	401	133	174	CAVCLSEFDDDDTLRLLPKCSHAFHVDCID AWLASHVTCPVC	Plasma Membrane	
TraesCS6D02G134300	H2	489	172	211	CPVCLERLDQDPGGILTTICNHSFHCSCISK WTDSSCPVC	Nuclear	BRCA1-associated 2
TraesCS6D02G140100	H2	183	133	174	CPVCLDAARAGERWRALPACGHAFHAAC VDRWLLLSPGCPVC	Plasma Membrane	

Identifiers	RING type	Protein size (amino acids)	Domain start	Domain end	RING domain	Localization	Description
TraesCS6D02G160300	H2	348	265	305	CAVCKDVFASGQRVVFLPCKHYFHGDCILPWLAMRSTCPVC	Nuclear	
TraesCS6D02G207700	H2	632	4	47	CAICFDSMKPGHGQALFTAECSHMFHFHCISSSVKHGNYVCPVC	Nuclear	
TraesCS6D02G209000a	HC	391	38	77	CCVCLELLYKPVVIACGHMSCFWCVHKAMHYARESHCAIC	Nuclear	
TraesCS6D02G209000b	HC	391	176	213	CPICKELLYQPTVLNCGHVYCISCLPSVGDEALKCQVC	Nuclear	
TraesCS6D02G218600	HC	241	31	78	CNICLDFAVEPVVTLCGHLYCWPCIYEWLRRDAGGGTISTAGRPCPVC	Plasma Membrane	
TraesCS6D02G221300	H2	188	135	176	CAICLSELADGERVRVLPACGHGFHGACVDGWLAARASCPTC	Nuclear	
TraesCS6D02G236900	H2	373	317	357	CCICLSVYDDGAELRELPCGHHFHCACIDKWLHINATCPLC	Plasma Membrane	
TraesCS6D02G237700	H2	208	104	145	CSICLGEYDEKEILRIMPTCRHNFHLSCIDIWLEKQTTCPIC	Plasma Membrane	Zinc finger, RING/FYVE/PHD-type
TraesCS6D02G240000	H2	205	112	154	CAVCLCSIGGGDEVRRLPNCRHVFHRGCIDRWMGHGQRTCPLC	Extracellular	
TraesCS6D02G240300	H2	154	90	132	CIVCLERLRAADEVRRLGNCAHAFHRGCIDGWIDLGRTTCPLC	Extracellular	
TraesCS6D02G242100	H2	170	94	135	CAICLAEFEDGQHMRVLPQCSHAFHAACVDAWLRSHSSCPSC	Extracellular	
TraesCS6D02G243200	H2	366	145	186	CAVCLSPFVPDAELRLLPACRHAFHAACVDAWLRNTPSCPLC	Nuclear	
TraesCS6D02G250600	H2	386	168	209	CSVCLGEFRDGESLRLLPKCSHAFHLPCIDTWLKSHSNCPLC	Nuclear	

Identifiers	RING type	Protein size (amino acids)	Domain start	Domain end	RING domain	Localization	Description
TraesCS6D02G254700	H2	116	50	105	CAICRNHIMDLCIECQANQASATSEECTVA WGVCNHAFHFHCISRWLKTRQVCPLD	Extracellular	
TraesCS6D02G262500	HC	376	204	261	CSVCLDRVLSKPTAAEKRFGLLPECDHAFC ITCIRKWRSSSLTSSMDIDSTVKACPIC	Nuclear	
TraesCS6D02G272200	H2	155	81	122	CAVCLEAFQGGDRCRVLPGCHHGFHAQCV DSWLRQSRRCPVC	Extracellular	
TraesCS6D02G272300	H2	126	66	107	CAVCLEAFNAGERCMVLPRCEHGFHAECV GSWLRKSRLCPIC	Extracellular	
TraesCS6D02G278100	H2	367	317	357	CCICLSSYEDGVELSALPCNHHFHSTCITKW LRMHANCPLC	Plasma Membrane	
TraesCS6D02G283700	HC	460	158	196	CAICLDICYQPSTTPCGHSFCMQCLKHAAS KCGKRCPKC	Nuclear	Zinc finger, RING/FYVE/PHD-type
TraesCS6D02G287400	H2	226	181	222	CAICLQDMVAGEMARRLPTCSHAFHQLCV DKWLISHGSCPVC	Extracellular	Zinc finger, RING/FYVE/PHD-type
TraesCS6D02G294800	H2	405	141	182	CAVCLSEFEDEDRLRLLPKCSHAFHPDCIGE WLASHVTCPVC	Nuclear	
TraesCS6D02G305800	HC	699	76	113	CPICMALIKDAFLTACGHSFCYMCIVTHLN HKSDCPCC	Nuclear	WD4/YVTN repeat-like-containing domain superfamily
TraesCS6D02G309800	H2	318	188	228	CPVCKEEFELGEAARQLPCKHVYHSDCIVP WLRLHNSCPVC	Nuclear	
TraesCS6D02G328700	H2	402	334	374	CCICLAKYAHNEELRELPCTHCFHKECVDK WLKINALCPLC	Plasma Membrane	

Identifiers	RING type	Protein size (amino acids)	Domain start	Domain end	RING domain	Localization	Description
TraesCS6D02G332200	HC	272	196	233	CPVCLNKLVEPSTTKCGHIFCAECIKQAIQFQKKCPTC	Nuclear	Zinc finger, RING/FYVE/PHD-type
TraesCS6D02G334900	H2	443	115	156	CAVCLCEFAPDDQLRLLPKCSHAFHLECIDTWLLSHSTCPLC	Nuclear	
TraesCS6D02G344900	H2	203	151	191	CAVCLEEFRAGDVLAHLPCAHRFHWACAVPWVQAASRCPFC	Nuclear	
TraesCS6D02G345700	H2	530	478	519	CHICLTEYEDGDQIRSLPCKHEFHLQCVDKWLKEIHRVCPLC	Nuclear	
TraesCS6D02G360000	H2	738	99	142	CAICLTIMKPGQGHALFTAECSHTFHFHCISANVKHGSNNCPVC	Nuclear	von Willebrand factor A-like domain superfamily
TraesCS6D02G382100	H2	250	137	178	CAVCLGEFQEGESVRLLPGCLHVFHAECIGTWLQGCANCPLC	Extracellular	Zinc finger, RING/FYVE/PHD-type
TraesCS6D02G382200	H2	279	163	204	CAVCLGEFQEGERVRLLPGCLHVFHAECIDTWLHGCASCPLC	Nuclear	
TraesCS6D02G383800	H2	171	120	160	CPICLEEYDEENPRSITKCEHHFHLCCILEWMERSETCPVC	Nuclear	
TraesCS6D02G389000	H2	199	117	165	CAVCLTELQLQESVKAIPACGHVFHPPCIDRWLQSLAASGAGHASCPLC	Nuclear	
TraesCS6D02G389100	HC	766	714	752	CCVCCDTQIDSLLYRCGHMCTCSKCANELVRSGGKCPLC	Nuclear	
TraesCS6D02G405300	H2	209	114	155	CAICLADLATGEELRVLPRCGHSFHVACVDAWLRTCATCPTC	Nuclear	

Identifiers	RING type	Protein size (amino acids)	Domain start	Domain end	RING domain	Localization	Description
TraesCS7A02G005100	H2	1198	1152	1192	CAICKSNFKSQQKLIRLPCSHSYHADCITRW LKINKSCPVC	Nuclear	P-loop containing nucleoside triphosphate hydrolase
TraesCS7A02G023300	H2	168	94	135	CAVCLGQVEAGEKLRRLPKCAHLFHADCV HAWLQAHSTCPMC	Extracellular	
TraesCS7A02G024600	H2	171	105	147	CAVCLGDFHGAARVRRPRGCRHVFHRGCL DRWAAHGHRTCPLC	Extracellular	
TraesCS7A02G025100	H2	163	104	146	CAVCLGDFHGAARVRRPRGCRHVLHRACL DRWAAHGHSTCPLC	Extracellular	
TraesCS7A02G025200	H2	163	101	143	CAVCLGDFHAAACVRRPRGCRHVFHRACL DRWAAHGHSTCPLC	Extracellular	
TraesCS7A02G129900	H2	362	141	182	CAVCLCEFAGDDRLRLLPLCGHAFHIDCID TWLLSNSTCPLC	Extracellular	
TraesCS7A02G144900	H2	153	97	141	CAVCLEEFAAGDVLAHLPCGHRFHWGCAL PWLEGAAAASHSCPFC	Nuclear	
TraesCS7A02G152600	H2	428	124	165	CAVCLCEFAMDDALRLLPTCGHAFHVPCID AWLLSHSTCPLC	Plasma Membrane	
TraesCS7A02G156900	H2	320	120	161	CAVCLSEVADGEKVRTLPKCGHRFHVDCI DMWFHSHDTCPLC	Plasma Membrane	
TraesCS7A02G169600	H2	319	183	223	CPVCKDEFELGETARELPCKHAYHSDCIVP WLQLHNSCPVC	Nuclear	
TraesCS7A02G176700	H2	415	152	193	CAVCLSEFEDGDQLRLLPKCSHAFHPDCIG EWLAGHVTCPVC	Nuclear	
TraesCS7A02G189900	H2	230	185	226	CPICLQDIMTGEMARRLPKCCHTFHQPCVD KWLIGHGSCPVC	Extracellular	
TraesCS7A02G190800	H2	408	167	208	CAVCLCEFDAEDRLRLLPLCGHAFHLNCID TWLLSNSTCPLC	Nuclear	

Identifiers	RING type	Protein size (amino acids)	Domain start	Domain end	RING domain	Localization	Description
TraesCS7A02G205000	H2	167	108	149	CAICLEEFQQGQPCSEVPACRHVFHRYCID VWMKSKATCPLC	Extracellular	
TraesCS7A02G210400	H2	215	117	158	CAVCLEAFESGDRCRRLPRCEHSFHAPCVD SWLKKSQCCPVC	Chloroplast	
TraesCS7A02G211800	H2	298	162	202	CAICKDDLPLAVAARRLPCGHLYHSVCIVQ WLEMHNSCPVC	Nuclear	
TraesCS7A02G212900	HC	946	38	78	CGICFTDSRRAIRGELDCCAHHFCFVCIMA WARVESRCPFC	Nuclear	
TraesCS7A02G220000	H2	491	292	329	CIICREEMTTAKKLLCGHLFHVHCLRSWLQ RQHTCPTC	Plasma Membrane	
TraesCS7A02G228200	HC	384	232	289	CSVCLDRVLSKPTAAERKFGLLSECDHPFCI SCIRNWRGNSPTSGMDVNSALRACPIC	Nuclear	
TraesCS7A02G247700	H2	269	207	247	CSVCKDGVVAGERVKMMPCSHRYHEDCIL PWLEVRNSCPLC	Chloroplast	
TraesCS7A02G253700	H2	301	104	145	CAVCLSEFQEGERLRLLPPCLHLFHIDCIDA WLHATANCPLC	Extracellular	
TraesCS7A02G256500	HC	200	142	179	CPVCLNKMELPSATSCGHVFCEKCIKAAIK AQKKCPTC	Nuclear	Zinc finger, RING/FYVE/PHD-type
TraesCS7A02G257600	HC	265	217	254	CCVCMARAKGAAFIPCGHTFCRACARELL AGRGRCPLC	Nuclear	
TraesCS7A02G268500	H2	428	21	66	CSICLDPVTGRAGGRSIAKLQCGHEFHLDCI GSAFNAKGAMQCPNC	Nuclear	
TraesCS7A02G271500	H2	288	236	276	CVICRVEFEEGESLVALPCKHSYHSDCIKQ WLQLNKVCPMC	Nuclear	Zinc finger, RING/FYVE/PHD-type

Identifiers	RING type	Protein size (amino acids)	Domain start	Domain end	RING domain	Localization	Description
TraesCS7A02G273000	H2	234	189	230	CSVCLQEFEAGEAARSLPECRHTFHMSCID GWLCRHASCPLC	Chloroplast	Zinc finger, RING/FYVE/PHD-type
TraesCS7A02G296500	HC	456	261	301	CNICLEAAKEPVVTPCGHLFCWPCLYQWL HGHSAHSECPVC	Nuclear	
TraesCS7A02G300000	H2	172	111	153	CAVCLGALEARHRVRELGNCAHAFHKACI DKWVDKGQATCPLC	Extracellular	
TraesCS7A02G308900	HC	401	356	388	CPICLTNAKDLAFGCGHMCCRECGENLDR CPIC	Chloroplast	Copine
TraesCS7A02G325800	H2	515	238	279	CAICLEDYSVGEKIRVLPCRHKFHAACVDL WLTSWRTFCPVC	Plasma Membrane	
TraesCS7A02G340400	v	903	25	73	QCRICRFPAEADRPLRRPCACSGSIRFVHDD CLLRWLATSRQSRCEVCS	Plasma Membrane	
TraesCS7A02G346400	H2	149	89	130	CAVCLGAIRVGAMAKLLPACAHVYHVECI DLWLAAHSTCPLC	Extracellular	
TraesCS7A02G346500	H2	174	124	165	CSVCLGALQVGETVRLLPACLHLYHAECID PWLDAHSTCPLC	Nuclear	
TraesCS7A02G346700	H2	155	98	139	CSVCLGALQLGDTVRLLPACLHVYHAECID PWLGAHSTCPIC	Extracellular	
TraesCS7A02G346800	H2	152	95	136	CSVCLGAFQLGETVRLLPACLHLYHVECID PWLDAHSTCPIC	Extracellular	
TraesCS7A02G346900	H2	152	95	136	CSVCLGVFQLGETVRLLPACMHLYHVECID PWLDAHSTCPIC	Mitochondrial	
TraesCS7A02G347000	H2	176	119	160	CSVCLGVFQLGETVRLLPACLHLYHVECID PWLDAHSTCPIC	Extracellular	
TraesCS7A02G347100	H2	185	128	169	CSVCLGAFLLGETVRLLPVCLHLYHVGCID PWLDAHSTCPLC	Plasma Membrane	

Identifiers	RING type	Protein size (amino acids)	Domain start	Domain end	RING domain	Localization	Description
TraesCS7A02G347200	H2	159	95	136	CSVCLGTVQAGEMVRLLPLCKHLYHVECI DMWLASHDTCPLC	Extracellular	
TraesCS7A02G347700	H2	163	100	141	CAVCLAEVRPKEVVKQLPACTHLFHDRCID EWLWSHRTCPVC	Plasma Membrane	Zinc finger, RING/FYVE/PHD-type
TraesCS7A02G347800	H2	239	175	217	CCVCLAGMREEQALRDLPRCGHRFHEKCI GKWLKTGHPTCPVC	Plasma Membrane	
TraesCS7A02G347900	H2	176	111	152	CAVCLEALSGGEKARRLPVCAHTFHVGCID MWFHSHATCPVC	Plasma Membrane	
TraesCS7A02G348000	H2	182	117	158	CAVCLEALSGREKARRLPVCAHMFHVGCI DMWFHSHVTCSVC	Plasma Membrane	
TraesCS7A02G348100	H2	200	135	176	CAVCLEDVQGGEMVRQLPPCRHLFHVDCI DMWLHTHRTCPLC	Plasma Membrane	
TraesCS7A02G348200	H2	324	122	163	CVVCLQELEDGDVVRVLPACRHFFHGSCID TWLRAHSSCPVC	Nuclear	
TraesCS7A02G352100	H2	296	114	155	CAVCLTELEDGEEARFLPRCGHGFHTECVD MWLASHTSCPLC	Plasma Membrane	
TraesCS7A02G372900	H2	216	143	184	CPVCLEDIHGGEMVRQLPACRHLFHVECID MWLDSHRTCPMC	Plasma Membrane	
TraesCS7A02G373200	H2	217	145	186	CPVCLEDIHGGEMVRQLPACRHLFHVECID MWLDSHRTCPMC	Plasma Membrane	
TraesCS7A02G381200	HC	441	398	430	CAICMDKSKDLAFGCGHQTCYDCGGKLVR CPMC	Nuclear	Copine
TraesCS7A02G381300	HC	457	414	446	CAICMDKSKDLAFGCGHQTCYDCGKNLVR CPMC	Nuclear	Copine
TraesCS7A02G391700	H2	331	257	297	CAVCKDGIVQGELATWLPCAHFYHGACIG PWLAIRNSCPVC	Cytoplasmic	

Identifiers	RING type	Protein size (amino acids)	Domain start	Domain end	RING domain	Localization	Description
TraesCS7A02G420500	HC	324	220	268	CSICLDTVFDAVSLSCGHIFCYLCCSAAASVTVVDGLESADPGSKCPIC	Extracellular	Zinc finger, RING/FYVE/PHD-type
TraesCS7A02G423300	H2	592	10	51	CAICLGGMVRGQASYTAECSHAFHLICISASVAHGNHTCPLC	Chloroplast	
TraesCS7A02G428300	H2	580	523	563	CSICQEEYIEDEEVGRMKCEHQYHVCCIQEWLRQKNWCPIC	Nuclear	
TraesCS7A02G441900	HC	316	269	303	CKGCREDAAVVLLLPCRHLCVCAPCAAAAQACPAC	Extracellular	E3 ubiquitin-protein ligase BOI-like
TraesCS7A02G442100	HC	314	267	301	CKACREGAAVVLLLPCRHLCVCAPCAAAALACPAC	Extracellular	Zinc finger, RING/FYVE/PHD-type
TraesCS7A02G442300	HC	317	270	304	CKGCREGAAVVLLLPCRHLCVCAPCAAVAQACPVC	Extracellular	E3 ubiquitin-protein ligase BOI-like
TraesCS7A02G458100	H2	135	61	102	CAVCLGQVEAGEKLRRLPKCAHLFHADCVHAWLQAHSTCPMC	Nuclear	
TraesCS7A02G463200	v	485	236	285	VCRICFVELNEGGETLKMECSCKGELALAHQDCAVKWFSIKGNKICDVCR	Nuclear	Zinc finger, RING-CH-type
TraesCS7A02G478200	H2	173	106	147	CAICLSDFADGETIRVLPVCGHRFHVLCVDRWLTSRCSCPTC	Extracellular	
TraesCS7A02G486700	HC	303	242	280	CVVCLTEPRDTTVLPCRHMCLCRECAQTLRFQTNKCPMC	Nuclear	
TraesCS7A02G503800	H2	178	112	153	CAVCLERYADSDVVRVLPDCGHLFHRGCVDAWLRRRPTCPVC	Plasma Membrane	
TraesCS7A02G524600	v	1097	47	95	VCRICRNRGDDEHPLRYPCACSGSIKFVHQDCLLQWLDHSNSRQCEVCK	Plasma Membrane	

Identifiers	RING type	Protein size (amino acids)	Domain start	Domain end	RING domain	Localization	Description
TraesCS7A02G526000	H2	250	197	238	CSVCLQDFASRQYVRALPQCRHIFHVPCID NWLQRNASCPLC	Extracellular	Zinc finger, RING/FYVE/PHD-type
TraesCS7A02G534800	H2	242	197	238	CSVCLQDFQIGEKVRSLPDCLHVFHVPCID GWLIKHGSCPLC	Plasma Membrane	
TraesCS7A02G551400	H2	244	123	164	CPVCLAAFEEGDNLRLLPHCSHVFHPECVD PWLQARATCPLC	Chloroplast	
TraesCS7A02G555800	H2	215	127	168	CAICLAEFTDGESVRVLPRCGHNFHMTCVD AWLRTCATCPSC	Nuclear	
TraesCS7A02G556000	H2	198	114	155	CAICLAEFTVGESMRVLPRCGHAFHVLCID TWLRTCTSCPFC	Extracellular	
TraesCS7A02G556100	H2	154	70	111	CAICLAEFVEGEVVRVLPRCGHTFHVLCIH AWLRTCATCPSC	Extracellular	
TraesCS7A02G556200	H2	207	118	159	CAICLADFTEGEGLRVLPRCGHHFHVGCVD AWLRTCATCPSC	Extracellular	
TraesCS7A02G556400	H2	208	121	162	CAICLAEYMEGEALRVLPRCGHGFHVACV DAWLRTCATCPSC	Extracellular	
TraesCS7A02G556500	H2	207	120	161	CAICLAEFTEGEALRVLPRCGHGFHVACVD AWLRTCATCPSC	Extracellular	
TraesCS7A02G556600	H2	161	87	128	CGICLAEFMEGEGLRVLPQCGHGFHVACV DAWLRTCATCPSC	Extracellular	
TraesCS7A02G556700	H2	211	117	158	CAICLAEFADGEAVRVLPGCGHGFHAACV DAWLRTRATCPSC	Plasma Membrane	
TraesCS7A02G563700	H2	162	99	140	CAVCLAEVRPKEVVKQLPACTHLFHDRCID EWLWSHRTCPVC	Plasma Membrane	Zinc finger, RING/FYVE/PHD-type
TraesCS7B02G029900	H2	364	142	183	CAVCLCEFAGDDRLRLLPLCGHAFHIDCID TWLLSNSTCPLC	Nuclear	

Identifiers	RING type	Protein size (amino acids)	Domain start	Domain end	RING domain	Localization	Description
TraesCS7B02G047200	H2	213	157	199	CAVCLDEFAAGDVLAHLPCGHRFHWGCALPWLEGAASHSCPFC	Nuclear	
TraesCS7B02G056400	H2	435	131	172	CAVCLCEFAMDDALRLLPTCGHAFHVPCIDAWLLSHSTCPLC	Nuclear	
TraesCS7B02G061100	H2	320	119	160	CAVCLSEVADGEKVRTLPKCGHRFHVDCIDMWFHSHDTCPLC	Plasma Membrane	
TraesCS7B02G074800	H2	322	184	224	CPVCKDEFEIGETARELPCKHAYHSDCIVPWLRLHNSCPVC	Nuclear	
TraesCS7B02G081700	H2	414	152	193	CAVCLSEFEDGDQLRLLPKCSHAFHPDCIGEWLAGHVTCPVC	Chloroplast	
TraesCS7B02G094900	H2	230	185	226	CPICLQDIVTGEMARRLPKCCHTFHQPCVDKWLIGHGSCPVC	Extracellular	
TraesCS7B02G095700	H2	410	169	210	CAVCLCEFDAEDRLRLLPLCGHAFHLNCIDTWLLSNSTCPLC	Nuclear	
TraesCS7B02G112200	H2	164	105	146	CAICLEELQQGQACSEVPVCRHVFHRDCIDAWMKSQTTCPLC	Extracellular	
TraesCS7B02G112300	H2	226	167	208	CAICQDEFLQGQMCSEVPACRHLFHRDCIEVWRKSKTTCPLC	Extracellular	
TraesCS7B02G117100	H2	180	82	123	CAVCLEAFESGDRCRRLPRCEHSFHAPCVDSWLKKSQCCPVC	Extracellular	
TraesCS7B02G118700	H2	298	162	202	CAICKDDLPLAVAARRLPCGHLYHSVCIVQWLEMHNSCPVC	Nuclear	
TraesCS7B02G127400	H2	491	292	329	CIICREEMTTAKKLLCGHLFHVHCLRSWLQRQHTCPTC	Plasma Membrane	
TraesCS7B02G134400	HC	399	356	388	CPICWSEAKNLAFGCGHQTCADCGKDLKVCPLC	Nuclear	Copine
TraesCS7B02G144100	H2	269	207	247	CSVCKDGVVAGERVKMMPCSHRYHEDCILPWLEVRNSCPLC	Chloroplast	

Identifiers	RING type	Protein size (amino acids)	Domain start	Domain end	RING domain	Localization	Description
TraesCS7B02G150000	H2	295	104	145	CAVCLSEFREGERLRLLPPCLHLFHIDCIDA WLHATANCPLC	Plasma Membrane	
TraesCS7B02G154000	HC	199	141	178	CPVCLNKMELPSATSCGHVFCEKCIKAAIK AQKKCPTC	Nuclear	Zinc finger, RING/FYVE/PHD-type
TraesCS7B02G154600	HC	263	215	252	CCVCMARAKGAAFIPCGHTFCRACARELL AGRGRCPLC	Nuclear	
TraesCS7B02G163800	H2	430	21	66	CSICLDPVAGRAGGRSIAKLQCGHEFHLDCI GSAFNAKGAMQCPNC	Nuclear	
TraesCS7B02G171000	H2	271	226	267	CSVCLQEFEAGEAARSLPECRHTFHMSCID GWLCRHASCPLC	Plasma Membrane	Zinc finger, RING/FYVE/PHD-type
TraesCS7B02G176700	v	311	54	106	CCRICLEAESEIGDELISPCMCKGTQQFVHR SCLDHWRSVKEGFAFSHCTTCK	Plasma Membrane	
TraesCS7B02G188200	H2	172	111	153	CAVCLGALEARHRVRELGNCAHAFHKACI DKWVDKGQATCPLC	Extracellular	
TraesCS7B02G189000	HC	461	266	306	CNICLEAAKEPVVTPCGHLFCWPCLYQWL HGHSAHSECPVC	Nuclear	
TraesCS7B02G191500	HC	384	232	289	CSVCLDRVLSKSTAAERKFGLLSECDHPFCI SCIRNWRGNSPTSGMDVNSALRACPIC	Nuclear	
TraesCS7B02G226400	H2	505	228	269	CAICLEDYSVGEKIRVLPCRHKFHAACVDL WLTSWRTFCPVC	Nuclear	
TraesCS7B02G234500	H2	323	123	164	CVVCLQELEDGDVVRVLPACRHFFHGSCID TWLRAHSSCPVC	Nuclear	
TraesCS7B02G234600	H2	263	127	168	CAVCLEELREGALVRMLPSCKHYFHATCV DVWLLSHATCPVC	Nuclear	

Identifiers	RING type	Protein size (amino acids)	Domain start	Domain end	RING domain	Localization	Description
TraesCS7B02G235200	H2	200	130	171	CAVCLEDVQGGEMVRQLPPCRHLFHVDCI DMWLHTHRTCPLC	Plasma Membrane	
TraesCS7B02G235300	H2	176	111	152	CAVCLEALSGGEKARRLPVCAHTFHVGCID MWFHSHATCPVC	Plasma Membrane	
TraesCS7B02G235400	H2	243	179	221	CCVCLAGMREEQALRDLPRCGHRFHEKCI GKWLKTGHPTCPVC	Plasma Membrane	
TraesCS7B02G235500	H2	182	130	171	CVVCLGEVGKGEETRRLPVCRHVFHRQCV ERWLMERSTCPVC	Plasma Membrane	
TraesCS7B02G235600	H2	171	108	149	CAVCLAEIKPKEVVKQLPACTHLFHDRCID EWLWSHRTCPVC	Extracellular	
TraesCS7B02G236000	H2	159	95	136	CSVCLGTVQAGEMVRLLPLCKHLYHVECI DMWLASHDTCPLC	Extracellular	
TraesCS7B02G236100	H2	197	140	181	CSVCLGVFQLGETVRLLPACMHLYHVECID PWLDAHSTCPIC	Nuclear	
TraesCS7B02G236200	H2	193	136	177	CSVCLGVFQLGEAVRLLPACMHLYHVECI DPWLDAHSTCPLC	Nuclear	
TraesCS7B02G236600	H2	186	136	177	CSVCLGAMQVGETVRLLPACLHLYHAECI DPWLDAHSTCPLC	Nuclear	
TraesCS7B02G236700	H2	149	89	130	CAVCLGAIRVGAMAKLLPACAHVYHVECI DLWLASHSTCPLC	Extracellular	
TraesCS7B02G252000	v	904	26	74	QCRICRFPAEADRPLRRPCACSGSIRFVHDD CLLRWLATSRQSRCEVCS	Plasma Membrane	
TraesCS7B02G253800	H2	217	145	186	CPVCLEDIHGGEMVRQLPACRHLFHVECID MWLDSHRTCPMC	Plasma Membrane	
TraesCS7B02G254300	H2	218	145	186	CPVCLEDIHGGEMVRQLPACRHLFHVECID MWLDSHRTCPMC	Plasma Membrane	
TraesCS7B02G255400	H2	218	145	186	CPVCLEDTHGGEMVRQLPACRHLFHVECID MWLDSHRTCPMC	Plasma Membrane	

Identifiers	RING type	Protein size (amino acids)	Domain start	Domain end	RING domain	Localization	Description
TraesCS7B02G276000	H2	293	111	152	CAVCLTELEDGEEARFLPRCGHGFHTECVD MWLASHTSCPLC	Plasma Membrane	
TraesCS7B02G283100	HC	443	400	432	CAICTDKSKDLAFGCGHQTCYDCGQNLVR CPMC	Nuclear	Copine
TraesCS7B02G283200	HC	456	413	445	CAICMDKSKDLAFGCGHQTCYDCGKNLVR CPMC	Nuclear	Copine
TraesCS7B02G293600	H2	330	256	296	CAVCKDGIVQGGLATRLPCAHFYHGACIGP WLAIRNSCPVC	Cytoplasmic	
TraesCS7B02G327800	H2	402	114	155	CAVCLGRFEPTEALRLLPKCRHGFHVECVD TWLDAHSTCPLC	Nuclear	
TraesCS7B02G328500	H2	578	523	563	CSICQEEYIEDEEVGRMKCEHQYHVCCIQE WLRQKNWCPIC	Nuclear	
TraesCS7B02G340500	HC	315	268	302	CKGCREGAAVVLLLPCRHLCVCASCAAAA QACPAC	Extracellular	Zinc finger, RING/FYVE/PHD-type
TraesCS7B02G340600	HC	312	265	299	CKACHDGAAVVLLLPCRHLSVCAPCAAAA QACPAC	Extracellular	E3 ubiquitin-protein ligase BOI-like
TraesCS7B02G340700	HC	312	265	299	CKGCREGAAVVLLLPCRHLCVCAPCAAAA QVCPVC	Extracellular	E3 ubiquitin-protein ligase BOI-like
TraesCS7B02G343400	H2	322	270	312	CPVCLQLLEGNDLAAWSGCDKPHVFHGSC LERVLVESKTCPMC	Cytoplasmic	
TraesCS7B02G343500	H2	388	336	378	CPICLQMLEGDDLATWPECGKPHVFHGAC LERVLVESKTCPMC	Cytoplasmic	
TraesCS7B02G363900	v	483	235	284	VCRICFVELNEGGETLKMECSCKGELALAH QDCAVKWFSIKGNKICDVCR	Nuclear	Zinc finger, RING-CH-type
TraesCS7B02G371200	H2	322	146	187	CSVCLGEFLDGELLRLLPKCGHAFHVPCIG TWLRAHVNCPLC	Nuclear	

Identifiers	RING type	Protein size (amino acids)	Domain start	Domain end	RING domain	Localization	Description
TraesCS7B02G380600	H2	228	162	203	CAICLSDFADGETIRVLPVCGHRFHVPCVD RWLASRCSCPTC	Extracellular	Zinc finger, RING/FYVE/PHD-type
TraesCS7B02G390000	HC	276	214	252	CVVCLTEPRDTTVLPCRHMCLCRECAQTLR FQTNKCPMC	Extracellular	
TraesCS7B02G442100	v	1099	47	95	VCRICRNRGDDEHPLRYPCACSGSIKFVHQ DCLLQWLDHSNSRQCEVCK	Plasma Membrane	
TraesCS7B02G443600	H2	265	212	253	CSVCLQDFGSRQFVRALPQCGHIFHVPCID NWLQRNASCPLC	Plasma Membrane	Zinc finger, RING/FYVE/PHD-type
TraesCS7B02G452400	H2	243	198	239	CSVCLQDFQIGEKVRSLPDCLHVFHVPCID GWLIKHGSCPLC	Plasma Membrane	
TraesCS7B02G475300	H2	192	97	138	CPVCLTAFVEGDSLRLLPHCSHVFHPVCIDP WLHQRATCPLC	Plasma Membrane	
TraesCS7B02G480500	H2	198	110	151	CAICLTEFTDGESVRVLPRCGHNFHVACVD AWLRTCATCPSC	Extracellular	
TraesCS7B02G480600	H2	181	97	138	CAICLAEFADGESMRVLPRCGHAFHVLCID AWLRTCATCPSC	Plasma Membrane	
TraesCS7B02G480700	H2	207	119	160	CAICLADFTEGEGLRVLPRCGHHFHVGCVD AWLRTCATCPSC	Extracellular	
TraesCS7B02G480900	H2	207	120	161	CAICLAEFTEGEALRVLPRCGHGFHVACVD AWLRTCATCPSC	Extracellular	
TraesCS7B02G481000	H2	203	115	156	CAICLAEFTEGEGLRVLPQCGHGFHVACVD AWLRSCATCPSC	Extracellular	
TraesCS7B02G481100	H2	197	104	145	CAICLAEFADGEPVRVLPGCGHGFHVACV DAWLRTRATCPSC	Plasma Membrane	

Identifiers	RING type	Protein size (amino acids)	Domain start	Domain end	RING domain	Localization	Description
TraesCS7D02G000400	H2	330	202	242	CAVCMDDFLLGAAAKQLPCNHVFHKDCIL PWLDLHSSCPVC	Nuclear	
TraesCS7D02G020500	H2	173	98	139	CAVCLGQVEAGEKLRRLPKCAHLFHADCV HAWLRAHSTCPMC	Nuclear	
TraesCS7D02G021000	H2	170	95	136	CAVCLGQVEAGEKLRRLPKCAHLFHADCV HAWLQAHSTCPMC	Extracellular	
TraesCS7D02G022600	H2	297	231	273	CAVCLGDFHGAARVRRPRGCRHVFHRGCL DRWASHGHHTCPLC	Plasma Membrane	
TraesCS7D02G023000	H2	161	101	143	CAVCFGEFHGAARVRRPRGCRHVFHRGCL DRWAAHGRRTCPLC	Extracellular	
TraesCS7D02G023100	H2	154	95	137	CAVCLGDFHGAAHVRRPRGCWHIFHRACL DRWAAYGHRTCPLC	Extracellular	
TraesCS7D02G043400	H2	159	104	146	CAVCLGDFHCAACVRRPRGCRHVFHRACL DRWAAHGHSTCPLC	Extracellular	
TraesCS7D02G049800	H2	251	203	243	CTVCLSAFRNRENIITLPCKHNYHASCISSW LKINRTCPVC	Nuclear	
TraesCS7D02G082700	H2	242	177	216	CPICMDDDEVAWKETKPCGHRFHGRCVEP WLKAKGSCPMC	Nuclear	
TraesCS7D02G125200	H2	202	111	155	CPICMEDDDDDNAATGAWKETPCGHRFHG RCVERWLRVKGSCPMC	Cytoplasmic	
TraesCS7D02G129000	H2	370	147	188	CAVCLCEFAGDDRLRLLPLCGHAFHIDCID TWLLSNSTCPLC	Nuclear	
TraesCS7D02G141500	H2	185	126	167	CVICLGLVQVGEMVRQLPACKHMFHVDCV DVWLRSHSTCPIC	Plasma Membrane	
TraesCS7D02G146300	H2	216	158	202	CAVCLDEFAAGDVLAHLPCGHRFHWGCAL PWLEGAAAASHSCPFC	Nuclear	
TraesCS7D02G154300	H2	432	127	168	CAVCLCEFAMDDALRLLPTCGHAFHVPCID AWLLSHSTCPLC	Nuclear	

Identifiers	RING type	Protein size (amino acids)	Domain start	Domain end	RING domain	Localization	Description
TraesCS7D02G157300	H2	323	119	160	CAVCLSEVADGEKVRTLPKCDHRFHVDCIDMWFHSHDTCPLC	Plasma Membrane	
TraesCS7D02G170800	H2	320	184	224	CPVCKDEFELGETARELPCKHAYHSDCIVPWLRLHNSCPVC	Extracellular	
TraesCS7D02G178000	H2	408	145	186	CAVCLSEFEDGDQLRLLPKCSHAFHPDCIGEWLAGHVTCPVC	Chloroplast	
TraesCS7D02G191100	H2	230	185	226	CPICLQDIVTGEMARRLPKCCHTFHQPCVDKWLIGHGSCPVC	Extracellular	
TraesCS7D02G191800	H2	410	169	210	CAVCLCEFDAEDRLRLLPLCGHAFHLNCIDTWLLSNSTCPLC	Nuclear	
TraesCS7D02G212200	H2	180	82	123	CAVCLEAFESGDRCRRLPRCEHSFHAPCVDSWLKKSQCCPVC	Extracellular	
TraesCS7D02G213400	H2	298	162	202	CAICKDDLPLAVAARRLPCGHLYHSVCIVQWLEMHNSCPVC	Nuclear	
TraesCS7D02G214800	HC	944	36	76	CGICFTDSRRAIRGELDCCAHHFCFVCIMAWARVESRCPFC	Nuclear	
TraesCS7D02G222100	H2	491	292	329	CIICREEMTTAKKLLCGHLFHVHCLRSWLQRQHTCPTC	Plasma Membrane	
TraesCS7D02G226400	HC	384	232	289	CSVCLDRVLSKPTAAERKFGLLSECDHPFCISCIRNWRGNSPTSGMDVNSALRACPIC	Nuclear	
TraesCS7D02G246300	H2	269	207	247	CSVCKDGVVAGERVKMMPCSHRYHEECILPWLEVRNSCPLC	Chloroplast	
TraesCS7D02G252300	H2	296	105	146	CAVCLSEFQEGERLRLLPPCLHLFHIDCIDAWLHATANCPLC	Plasma Membrane	
TraesCS7D02G257900	HC	201	143	180	CPVCLNKMELPSATSCGHVFCEKCIKAAIKAQKKCPTC	Nuclear	Zinc finger, RING/FYVE/PHD-type

Identifiers	RING type	Protein size (amino acids)	Domain start	Domain end	RING domain	Localization	Description
TraesCS7D02G258600	HC	265	217	254	CCVCMARAKGAAFIPCGHTFCRACARELF AGRGRCPLC	Nuclear	
TraesCS7D02G266300	H2	430	21	66	CSICLDPVAGRAGGRSIAKLQCGHEFHLDCI GSAFNAKGAMQCPNC	Nuclear	
TraesCS7D02G271800	H2	288	236	276	CVICRVEFEEGESLVALPCKHSYHSDCINQ WLQLNKVCPMC	Nuclear	Zinc finger, RING/FYVE/PHD-type
TraesCS7D02G273100	H2	234	189	230	CSVCLQEFEAGEAARSLPECRHTFHMSCID GWLCRHASCPLC	Chloroplast	Zinc finger, RING/FYVE/PHD-type
TraesCS7D02G282300	v	311	54	106	CCRICLEAESEIGDELISPCMCKGTQQFVHR SCLDHWRSVKEGFAFSHCTTCK	Plasma Membrane	
TraesCS7D02G293200	HC	461	266	306	CNICLEAAKEPVVTPCGHLFCWPCLYQWL HGHSAHSECPVC	Nuclear	
TraesCS7D02G295800	H2	172	111	153	CAVCLGALEARHRVRELGNCAHAFHKACI DKWVDKGQATCPLC	Extracellular	
TraesCS7D02G305600	HC	406	361	393	CPICLTNAKDLAFGCGHMCCRECGENLDR CPIC	Chloroplast	Copine
TraesCS7D02G322500	H2	515	238	279	CAICLEDYSVGEKIRVLPCRHKFHAACVDL WLTSWRTFCPVC	Nuclear	
TraesCS7D02G330600	H2	230	94	135	CAVCLEELRGGALVRMLPSCKHYFHATCV DVWLLSHATCPVC	Nuclear	
TraesCS7D02G330800	H2	323	123	164	CVVCLQELEDGDVVRVLPACRHFFHGSCID TWLRAHSSCPVC	Nuclear	
TraesCS7D02G330900	H2	205	135	176	CAVCLEDVQGGEMVRQLPPCRHLFHVDCI DMWLHTHRTCPLC	Plasma Membrane	

Identifiers	RING type	Protein size (amino acids)	Domain start	Domain end	RING domain	Localization	Description
TraesCS7D02G331000	H2	179	114	155	CAVCLEALSGGEKARRLPVCTHTFHVGCID MWFHSHATCPVC	Plasma Membrane	
TraesCS7D02G331100	H2	180	115	156	CAVCLEALSGGEKVRRLPVCAHTFHVGCID MWFHSHATCPVC	Plasma Membrane	
TraesCS7D02G331200	H2	243	179	221	CCVCLAGMREEQALRDLPRCGHRFHEKCI GKWLKTGHPTCPVC	Plasma Membrane	
TraesCS7D02G331300	H2	171	108	149	CAVCLAEIKPKEVVKQLPACTHLFHDRCID EWLWSHRTCPVC	Plasma Membrane	
TraesCS7D02G331800	H2	159	95	136	CSVCLGTVQAGEMVRLLPLCKHLYHVECI DMWLASHDTCPLC	Extracellular	
TraesCS7D02G331900	H2	186	129	170	CSVCLGAFLLGETVRLLPVCLHLYHVGCID PWLDAHSTCPLC	Plasma Membrane	
TraesCS7D02G332000	H2	155	99	140	CSVCLGAFQLGETVRLLPACLHLYHVECID PWLDAHSTCPLC	Extracellular	
TraesCS7D02G332200	H2	155	98	139	CSVCLGALQLGDTVRLLPACLHVYHAECID PWLGAHSTCPIC	Extracellular	
TraesCS7D02G332500	H2	195	136	177	CSVCLGAMQVGEMVRLLPACLHLYHTECI DPWLAAHSTCPVC	Extracellular	
TraesCS7D02G332600	H2	187	130	171	CSVCLGVLQLGETVRLLPACLHLYHAECID PWLEAHSTCPIC	Plasma Membrane	
TraesCS7D02G332700	H2	148	88	129	CAVCLAAIRVGAMAKLLPACAHVYHVECI DLWLASHSTCPLC	Extracellular	
TraesCS7D02G349700	H2	217	145	186	CPVCLEDIHGGEMVRQLPACRHLFHVECID MWLDSHRTCPMC	Plasma Membrane	
TraesCS7D02G350200	H2	210	137	178	CPVCLEDIHGGEMVRQLPACRHLFHVECID MWLDSHRTCPMC	Plasma Membrane	
TraesCS7D02G370800	H2	294	112	153	CAVCLTELEDGEEARFLPRCGHGFHTECVD MWLASHTSCPLC	Plasma Membrane	

Identifiers	RING type	Protein size (amino acids)	Domain start	Domain end	RING domain	Localization	Description
TraesCS7D02G377500	HC	454	411	443	CAICMDKSKDLAFGCGHQTCYDCGEKLVRCPMC	Nuclear	Copine
TraesCS7D02G377600	HC	457	414	446	CAICMDKSKDLAFGCGHQTCYDCGKNLVRCPMC	Nuclear	Copine
TraesCS7D02G387300	H2	326	252	292	CAVCKDGIVQGELATRLPCAHFYHGACIGPWLAIRNSCPVC	Cytoplasmic	
TraesCS7D02G413200	HC	322	218	266	CSICLDTVFDAVSLSCGHIFCYPCCSAAASVTVVDGLESADPGSKCPIC	Extracellular	Zinc finger, RING/FYVE/PHD-type
TraesCS7D02G415800a	HC	452	34	82	CPMCTTTAPIIHKSSFGSCGHAFCNSCVATYIAVRQRENAALPVECPDC	Extracellular	Zinc finger, RING/FYVE/PHD-type
TraesCS7D02G415800b	HC	452	122	169	CAKCMHVVPCACIRRSFTSCAHDFCSSCVSRRLRGRRGDGKHFYCTKC	Extracellular	Zinc finger, RING/FYVE/PHD-type
TraesCS7D02G420000	H2	401	114	155	CAVCLGRFEPTEALRLLPKCRHGFHVECVDTWLDAHSTCPLC	Chloroplast	
TraesCS7D02G420400	H2	573	518	558	CSICQEEYIEDEEVGRMKCEHQYHVCCIQEWLRQKNWCPIC	Nuclear	
TraesCS7D02G431100	HC	314	267	301	CKGCREGAAVVLLLPCRHLCVCAPCAAAAQACPAC	Extracellular	Zinc finger, RING/FYVE/PHD-type
TraesCS7D02G431200	HC	308	261	295	CKGCREDAAVVLLLPCRHLCVCAPCAAAAQACPAC	Extracellular	Zinc finger, RING/FYVE/PHD-type
TraesCS7D02G431300	HC	313	266	300	CKGCREGAAVVLLLPCRHLCVCAPCAAAAQACPVC	Extracellular	E3 ubiquitin-protein ligase BOI-like

Identifiers	RING type	Protein size (amino acids)	Domain start	Domain end	RING domain	Localization	Description
TraesCS7D02G433200	H2	384	332	374	CPICLQQLKGDDLAAWPGCGKPHVFHGAC LERVHVENKTCPMC	Cytoplasmic	
TraesCS7D02G433400	H2	452	397	441	CPVCLQLLEVEGDDLAAWPGCSKPHVFHS ACLERALVESVRCPMC	Nuclear	
TraesCS7D02G451000	v	485	236	285	VCRICFVELNEGGETLKMECSCKGELALAH QDCAVKWFSIKGNKICDVCR	Nuclear	Zinc finger, RING-CH-type
TraesCS7D02G457000	H2	324	143	184	CSVCLGEFLDGELLRLLPKCGHAFHVPCIG TWLRAHVSCPLC	Nuclear	
TraesCS7D02G465600	H2	167	101	142	CAICLSDFKDGETIRVLPVCGHRFHVPCVD RWLTSRCSCPTC	Extracellular	
TraesCS7D02G473200	HC	303	241	279	CVVCLTEPRDTTVLPCRHMCLCRECAQTLR FQTNKCPMC	Nuclear	
TraesCS7D02G513500	v	1094	44	92	VCRICRNRGDDEHPLRYPCACSGSIKFVHQ DCLLQWLDHSNSRQCEVCK	Plasma Membrane	
TraesCS7D02G514900	H2	251	198	239	CSVCLQDFGSCQFVRVLPQCQHIFHVPCID NWLQRNASCPLC	Extracellular	Zinc finger, RING/FYVE/PHD-type
TraesCS7D02G522000	H2	243	198	239	CSVCLQDFQIGEKVRSLPDCLHVFHVPCID GWLIKHGSCPLC	Plasma Membrane	
TraesCS7D02G537300	H2	213	118	159	CPVCLTAFEEGDSLRLLPHCSHVFHPECIDP WLQRRATCPLC	Plasma Membrane	
TraesCS7D02G538000	H2	256	125	166	CPVCLAVFEEGDNLRLLPHCSHVFHPECIDP WLQARATCPLC	Chloroplast	
TraesCS7D02G547300	H2	170	107	148	CAVCLAEVRPKEVVKQLPACTHLFHDRCID EWLWSHRTCPVC	Plasma Membrane	

Identifiers	RING type	Protein size (amino acids)	Domain start	Domain end	RING domain	Localization	Description
TraesCS7D02G554000	H2	198	105	146	CAICLAEFADGEAVRVLPGCGHGFHIACVD AWLRTRASCPSC	Plasma Membrane	
TraesCS7D02G554100	H2	199	116	157	CAICLAEFMEGEGLRVLPRCGHGFHVACV DAWLRTCATCPSC	Plasma Membrane	
TraesCS7D02G554200	H2	206	119	160	CAICLAEFAEGDGLRVLPLCSHRFHVACVD AWLRTCATCPSC	Extracellular	
TraesCS7D02G554300	H2	207	120	161	CAICLAEYTEGEALRVLPRCGHRFHVACVD AWLRTCATCPSC	Extracellular	
TraesCS7D02G554400	H2	205	116	157	CAICLAEFTEGEGLRVLPRCGHRFHVACVD AWLRTCATCPSC	Extracellular	
TraesCS7D02G554500	H2	199	115	156	CAICLAEFAEGEGVRVLPRCCHTFHVVCVD AWLRTCATCPSC	Nuclear	
TraesCS7D02G554600	H2	199	115	156	CAICLAEFAEGEGVRVLPRCCHTFHVVCVD AWLRTCATCPSC	Plasma Membrane	
TraesCS7D02G554700	H2	216	128	169	CAICLTEFTDGESVRVLPLCGHNFHVACVD GWLRTCATCPSC	Extracellular	
TraesCSU02G011000	H2	222	174	215	CIICLQDMVAGETARRLPNCSHTFHQPCVD KWLFTHGSCPVC	Extracellular	Zinc finger, RING/FYVE/PHD-type
TraesCSU02G056200	H2	119	53	108	CAICRNHIMDLCIECQANQASATSEECTVA WGVCNHAFHFHCISRWLKTRQVCPLD	Nuclear	
TraesCSU02G068200	H2	220	170	210	CPTCLEDYTSENPRIVMQCSHHFHLGCIYE WMERSEACPVC	Nuclear	
TraesCSU02G068700	H2	389	33	73	CSICLEAFCESDPSTLTGCKHEFHLQCILEW CQRSSQCPMC	Nuclear	
TraesCSU02G093700	H2	398	114	155	CAVCLGRFEPTEALRLLPKCRHGFHVECVD TWLDAHSTCPLC	Chloroplast	

Identifiers	RING type	Protein size (amino acids)	Domain start	Domain end	RING domain	Localization	Description
TraesCSU02G109900	H2	367	191	232	CAVCLAEFRGGETLRLLPRCAHAFHRGCIDTWLRAHVNCPLC	Nuclear	
TraesCSU02G113400	HC	351	304	338	CRLCGAKEAAVLVMPCRHLCLCADCDRVADACPVC	Nuclear	E3 ubiquitin-protein ligase BOI-like
TraesCSU02G121400	H2	223	125	166	CAVCLAGLERGEEARALPRCGHRFHVGCIDAWFRGNSTCPLC	Plasma Membrane	
TraesCSU02G152200	H2	170	99	138	CPICIEDDDSAAWKETPCEHRFHGRCLERWLQAKGSCPMC	Extracellular	
TraesCSU02G152300	H2	170	99	138	CPICIEDDDSAAWKETPCEHRFHGRCLERWLQAKGSCPMC	Extracellular	
TraesCSU02G176500	H2	135	83	124	CAVCLAEYADGDELRVLPGCAHAFHRRCVDQWLRHRPSCPLC	Extracellular	
TraesCSU02G176600	H2	135	83	124	CAVCLAEYADGDELRVLPGCAHAFHRRCVDQWLRHRPSCPLC	Extracellular	
TraesCSU02G195400	H2	149	96	137	CAVCLAEYADGDELRRLPGCRHSFHRRCVHDWLRRRPSCPLC	Extracellular	
TraesCSU02G200100	H2	156	103	144	CAVCLAEYADADELRRLPGCKHVFHRLCVDEWLRRRPSCPLC	Extracellular	
TraesCSU02G211400	H2	152	99	140	CAVCLAEYADGDELRRLPGCQHAFHRRCVDEWLRRRPSCPLC	Extracellular	
TraesCSU02G220900	H2	149	96	137	CAVCLAEYADGDELRRLPGCRHSFHRRCVHDWLRRRPSCPLC	Extracellular	
TraesCSU02G230000	H2	158	105	146	CVVCLAEYADGDELRRLPGCKHAFHRLCVDEWLRRRPSCPLC	Extracellular	

Supplemental Table S2. The 117 incomplete RING domains (not classified in any of the four groups) due the absence or substitution of any of the eight metal ligand residues.

Identifiers	RING type	Protein size (amino acids)	Domain start	Domain end	Incomplete RING domain
TraesCS1A02G005700a	ND	792	12	57	CMVCRAAAPPEVELLRCATCATPWHSPCLSKPPALADAAAWACPDC
TraesCS1A02G011700	ND	169	105	146	CAVCLERYADSDMVRVLPDCGHLFRRGCVDPWLRQRPTCPVC
TraesCS1B02G159800	ND	182	139	176	CGICLEVNAKIVLPDCTHSLCLRCFEEWNAKSKSCPFA
TraesCS1D02G002400a	ND	506	12	57	CMVCRAAAPPEVELLRCATCATPWHSPCLSRPPALADAAAWACPDC
TraesCS2A02G178500a	ND	351	144	191	CAICMETVPGTLKFSVGSCGHAFCSGCVAQYVAAKLGENVARVKCPDP
TraesCS2A02G178500b	ND	351	307	349	CPKCRMYVEKSEGCNYIKCRCGSSFCYRCASKVSALTHYCNKC
TraesCS2A02G336300	ND	347	277	318	CPICYEDLDPTDSSFLPCPCGFHLCLFCHKRILEADARCPGC
TraesCS2A02G479900	ND	1037	724	764	MDSRTGPKEPPENAVITTCCHIFCYECAQESLSEEEVCPVC
TraesCS2A02G526800b	ND	526	232	295	CAKCMHVVPCACIRPSFTSCAHDLCSSCVSQQLQGRRRQAGKLPRHQPADLLPKPKGNGDLPVD
TraesCS2A02G526800c	ND	526	303	350	CAICMELVPGTLRFRVNSCAHAFCSSCVAQYIAAKLDDMVARIECPHP
TraesCS2A02G545900b	ND	681	230	266	CAKCMHVVPCACTRYNFTMCDHNFCSRCVAMKLCQNV
TraesCS2A02G545900d	ND	681	474	521	CNICMEMVARTLKFSVNSCGHVFCSSCITQYVAAKLDNNVARVECPDP
TraesCS2A02G545900e	ND	681	637	679	CPKCQMYVEKSEGCNYIKCRCGYSFCYRCASKLSALNHFCNKC
TraesCS2A02G546000c	ND	644	374	429	CACIRPSFTSCAHDFCSSCVSRQLQGLRRRAGKMPQHQPPVDMVLFTEGNGDSPVD
TraesCS2A02G546000d	ND	644	437	480	CAICMETVPGALKFIINSCGHAFCSSCVAQYVAAKLDDNVCPIE
TraesCS2A02G546000e	ND	644	600	642	CPECQMFVEKSEGCNYIKCRCGYSFCYRCASELSAQNHHCNNC
TraesCS2A02G559700	ND	372	124	171	TDIESGHFRPQDAIVNLGCSCKNELALAHYACALKWFISHGSTVCEIC
TraesCS2A02G559800	ND	416	123	165	HLQRQDDIVNLGCSCKNELALAHYACALKWFISHGSTVCEICG
TraesCS2B02G144500b	ND	394	189	236	CAICMETVPGTLKFSVSSCGHPFCRSCVAQYVAAKLDDKVARVECPHP
TraesCS2B02G144500c	ND	394	350	392	CPKCRMFVEKSEGCNYMKCRCGNSFCYRCASKLSAQNHYCKKC
TraesCS2B02G203600a	ND	390	183	230	CPICMETVPGILKFSVASCSHAFCSNCVAQYVAAKLSENVARVKCPDP
TraesCS2B02G203600b	ND	390	346	388	CPQCKMYVEKSEGCNYIKCRCGCSFCYRCASKVSAQTHYCGMC
TraesCS2B02G205500a	ND	351	144	191	CAICMETVPGTLKFSVGSCGHAFCSGCVVRYVAAKLGENVARVKCPDP
TraesCS2B02G205500b	ND	351	307	349	CPKCRMYVEKSEGCNYIKCRCGNSFCYRCASKVSALTHYCKEC
TraesCS2B02G268800	ND	360	31	71	SICLEAFCDSDPSTVTSCKHDFHLQCILEWCQRSSQCPMCW
TraesCS2B02G345600	ND	348	277	318	CPICYEDLDPTDSSFLPCPCGFHLCLFCHKRILEADARCPGC

Identifiers	RING type	Protein size (amino acids)	Domain start	Domain end	Incomplete RING domain
TraesCS2B02G467200a	ND	553	236	283	CMICFSEYKGIDFIKLPCRHYFCRNCMETYSQMHVKEGSVMKIVCPDD
TraesCS2B02G504600	ND	1041	718	758	MDSRTGPKEPPENAVITTCCHIFCYECAQESLSEEEVCPVC
TraesCS2B02G576800d	ND	684	477	524	CNICMEMVARTLKFSINSCDHVFCSSCITQYVAAKLDNNVARVECPDP
TraesCS2B02G576800e	ND	684	640	682	CPKCQMYVEKSEGCNYIKCRCGYSFCYRCASKLSALNHFCSKC
TraesCS2B02G577000b	ND	601	328	386	CACIRPSFTSCAHDFCSSCVARPTSQAQGLRRRAGKMPQHQPPVDMLLLTEGNGDSPVD
TraesCS2B02G577000c	ND	601	394	437	CAICMETVPGALKFIINSCGHAFCSSCLAQYVAAKLDDNVCPIE
TraesCS2B02G577000d	ND	601	557	599	CPKCQMFVEKSEGCNYIKCRCGYSFCYRCASELSAQNHYCNKC
TraesCS2B02G619900	ND	406	130	172	HLQRQDDVVNLGCSCKNELALAHYACALKWFISHGSTVCEICG
TraesCS2B02G620000	ND	528	286	328	HLHPQDDIVNLGCSCKNELALAHYACALKWFISHGSTVCEICG
TraesCS2D02G125100b	ND	400	194	241	CAICMETVPGALKFSVSPCGHPFCRSCVARYVAAKLDDKVAGVACPHP
TraesCS2D02G125100c	ND	400	356	398	CPKCRMFVEKSEGCNYMKCRCGNSFCYRCASKLSAENHYCKKC
TraesCS2D02G184500a	ND	377	170	217	CAICMETVPGVRKFSVSSCGHAFCSGCVVRYVAAKLGENVPRVKCPDP
TraesCS2D02G184500b	ND	377	333	375	CPKCKMYVEKSEGCNYIKCRCGYSFCYRCAFKVSARTHFCNKC
TraesCS2D02G184700a	ND	374	192	239	CAICMETVPGVRKFSVGSCGHAFCSGCVAQYVAAKLGENVARVKCPDP
TraesCS2D02G184700b	ND	374	330	372	CPQCKMYVEKSEGCNYIKCRCGCSFCYRCASKVSAQTHYCGMC
TraesCS2D02G187100b	ND	349	305	347	CPKCRMYVEKSEGCNYIKCRCGNSFCYRCASKVSALTHYCNKC
TraesCS2D02G326600	ND	347	277	318	CPICYEDLDPTDSSFLPCPCGFHLCLFCHKRILEADARCPGC
TraesCS2D02G445000a	ND	497	180	227	CVICFSEYKGIDFIKLPCRHYFCRNCMETYSQMHVKEGSVMKIVCPDD
TraesCS2D02G547100c	ND	496	289	336	CNICMEMVARTLKFSVNSCGHVFCSSCITQYIATKLDNNVARVECPDP
TraesCS2D02G547100d	ND	496	452	494	CPKCQMYVEKSEGCNYIKCRCGYSFCYRCTTKLSALNHFCNKC
TraesCS2D02G547300c	ND	609	402	445	CAICMETVPGTLKFIINSCGHAFCSSCVAQYVAAKLDDNVCPIE
TraesCS2D02G547300d	ND	609	565	607	CPECQMFVEKSEGCNYIKCRCGYSFCYRCASELSAQNHYCNKC
TraesCS2D02G570300	ND	458	118	162	ESGHLQRQDDVVNLGCSCKNELALAHYACALKWFISHGSTVCEIC
TraesCS2D02G570400	ND	371	127	171	ESGPLHPQDDIVTLGCSCKNELALAHYACALKWFISHGSTVCEIC
TraesCS3A02G133600	ND	956	9	61	CPLCMETMDLTDKQLKPCKCGYEICLWCWHHIMEMDQKDECGGRCPGCRSIYN
TraesCS3A02G139900	ND	218	114	155	CAVCLSELADGEKVRELPNCGHSFHVECVDAWLRSRTTYPLC
TraesCS3A02G309300	ND	120	32	72	CPICFGQEGGTIAWKETVCGHIFHGRGVEKWLEMKGSCPMC

Identifiers	RING type	Protein size (amino acids)	Domain start	Domain end	Incomplete RING domain
TraesCS3A02G353800	ND	388	331	374	CPVLREQSSEDNPPMLMPCGHAVSKQSIMKLSKSSSRPFKCPYC
TraesCS3B02G023100	ND	236	45	111	TGVALSGRRRRARVRSARTAGPPARGVDPELLRSLPVTARFLPGCGHGFHAGCVDRWLAAHTTCPLC
TraesCS3B02G139400	ND	272	195	242	CCICQDQDIAAAEGGGDDEGTVRLPCSHTFHVRWLARWFEAVTTCPLC
TraesCS3B02G152500	ND	123	32	72	CPICLDQEGATAVWKETVWGHIFHGRCVERWLQTKGSCPMC
TraesCS3B02G165900	ND	959	9	61	CPLCMETMDLTDKQLKPCKCGYEICLWCWHHIMEMDQKDECGGRCPGCRSIYN
TraesCS3B02G386500	ND	386	329	372	CPVLREQSSEDNPPMLMPCGHAVSKQSIMKLSKSSSRPFKCPYC
TraesCS3D02G149900	ND	958	9	61	CPLCMETMDLTDKQLKPCKCGYEICLWCWHHIMEMDQKDECGGRCPGCRSIYN
TraesCS3D02G313600	ND	1166	873	911	CALCNDAPEDAVVTTCGHVFCNQCILEQLTGDDSICPVS
TraesCS3D02G347800	ND	386	329	372	CPVLREQSSEDNPPMLMPCGHAVSKQSIMKLSKSSSRPFKCPYC
TraesCS3D02G406200	ND	182	157	182	KCAVCLGEVEKGDTVKRLPVCLHMFH
TraesCS4A02G101400a	ND	304	43	73	CCLCLKPLIDPLACPKGHTFCKECILECLLA
TraesCS4A02G185800	ND	147	95	135	CAVCKESFEVGDKIRKMPCSRGYHESCIFRWLRISRACPLC
TraesCS4B02G203200a	ND	304	43	73	CCLCLKPLIDPLACPKGHTFCKECILECLLA
TraesCS4B02G380900b	ND	497	320	368	CSIYVEPWTSTDEHHICCIPCGHVYGRSCLRRWLRDQLSRNENLKCPQC
TraesCS4B02G381100a	ND	441	58	119	CPCCTEPWTSDGPHRMCCIPCGHVYGRSCMETLLRRSGQNSAKEINAFYEHIFVAVRATCID
TraesCS4B02G381100b	ND	441	279	330	CSICLEPWTSTGKHHICCIPCGHVYGRSCLTRWLMDQLSRNENMKEVHEYYD
TraesCS4D02G070400	ND	534	126	177	CMVCFDEFSVGAVSIMECGHYFCNDCWTEHFKASVDSGKKQIRCMGVKCPVV
TraesCS4D02G204100a	ND	304	43	73	CCLCLKPLIDPLACPKGHTFCKECILECLLA
TraesCS4D02G357700	ND	488	1	44	MEPWTSEGEHRISCIPCGHVYGRSCLERWLTQRGNASATCPQCG
TraesCS5A02G028100	ND	154	101	142	CAVCLAEYADGDELRRLPGCKHAFPRLCVDEWLRRRPSCPLC
TraesCS5A02G141300	ND	331	201	244	CEVCNEAGIKASTCSKEGCNVRIHIYCLKKKFPQRKASRACPQC
TraesCS5A02G215600a	ND	654	364	411	CGICMDTLPVFDLFHGLPCKHKFCATCMTTYVEGRIRASELPIPCPDA
TraesCS5A02G215600b	ND	654	460	527	CGVILETSGGAEPAMAPCPACKHQLCATCGMEWSPDGAAGEHDCAKGPDAALVRRLAQERQWKQCPSC
TraesCS5A02G547400b	ND	477	319	386	CSICMEPWTSTDEHHICCIPCGHVYGWSCLIRWLRDQLSRNENLKEVHEYYETKLAEFEASREECMIF
TraesCS5B02G027400	ND	134	70	111	ATVCLAEYADGDELRVLPGCAHAFHRRCVDQWLWRRPSCPLC
TraesCS5B02G139600	ND	331	201	244	CEVCNEAGIKASTCSKEGCNVRIHIYCLKKKFPQRKASRACPHC
TraesCS5B02G214100a	ND	664	367	414	CGICMDTLPILDLFHGLPCKHKFCATCMTIYVEGRIRASELPIPCPDA

Identifiers	RING type	Protein size (amino acids)	Domai n start	Domai n end	Incomplete RING domain
TraesCS5B02G214100b	ND	664	463	530	CGVILETSGQAEPAMAPCPACKHLLCATCGMEWSPDGAAGEHDCAKGPDAALVRSLAQERRWKQCPSC
TraesCS5B02G214500	ND	284	243	277	CAVCRENLVVDDKMQELPCKHLFHPPCLKPWLLTD
TraesCS5B02G223400a	ND	392	180	227	CTICMETVPGVERFRISGCRHAFCASCVRQYIAARVEENLLAIGCPEP
TraesCS5B02G279200	ND	122	84	117	CVICCMGFEEGESLVALPCKHSYHPDCINQSNKS
TraesCS5D02G151100	ND	331	201	244	CEVCNEAGIKASTCSKEGCNVRIHIYCLKKKFPQRKASRACPQC
TraesCS5D02G161900	ND	1068	12	59	CPLCAEEMDITDQQLKPCKCGYEICVWCWHHIIDMAEKEDTEGRCPAC
TraesCS5D02G223400a	ND	668	370	417	CGICMDTLPVFDLFHGLPCKHKFCATCMTTYVEGRIRASELPIPCPDA
TraesCS5D02G223400b	ND	668	466	533	CGVILETSGQAEPAMAPCPACKHLLCATCGMEWSPDGAAGEHDCAKGPDAALVRRLAQERQWKQCPSC
TraesCS5D02G286700	ND	118	84	118	EQCVICRVGFEEGESLVALPCKHSYHPDCINQSHK
TraesCS6A02G244500	ND	149	86	127	CPICLYAMEPGRAVRVLPGCNRAFHQDCVDRWLAISPRCPVC
TraesCS6A02G248600	ND	221	12	60	CSICMEPMAPSDAHRGGSGCAHAFCRACLAGHVRAKVEAGAAVVRCPGV
TraesCS6A02G251500	ND	151	86	127	CPICLDAMEPGRAVRVLPGCNRAFHQDCVDRWLAISPRCPVC
TraesCS6A02G252500	ND	386	329	370	CPVLREQGSDDNPPMLLPCGHVLSKQSTVKLSKSSSRSFKCP
TraesCS6B02G220900	ND	293	3	41	CNACWRELEGQAITTTCGHLLCTEDAKKILSNDGACPIC
TraesCS6B02G272500	ND	423	329	370	CPVLREQGSDDNPPMLLPCGHVLSKQSTAKLSKSSSRSFKCP
TraesCS6B02G273800	ND	151	86	127	CPICLDAMEPGRAVRVLPGCNRAFHQDCVDRWLAISPRCPVC
TraesCS6B02G279800	ND	149	86	127	CPICLDAMEPGRAVRVLPGCNRAFHQDCVDRWLAISPRCPVC
TraesCS6D02G182400	ND	294	3	41	CNACWRELEGQAITTTCGHVLCTEDAKKILSNDGACPIC
TraesCS6D02G233300	ND	150	85	126	CPICLDAMEPGRAVRVLPGCNRAFHQDCVDRWLAISPRCPVC
TraesCS6D02G234000	ND	423	329	370	CPVLREQGSDDNPPMLLPCGHVLSKQSTVKLSKSSSRSFKCP
TraesCS7A02G054700	ND	242	200	234	HDENRENIITLPCKHNYHASCISNWLKINRTCPVC
TraesCS7A02G274800	ND	134	76	113	CIICREETTTAKKLLCGHLFHVHCLRSWRERQHTFPTC
TraesCS7A02G367600	ND	386	329	372	CPVLREQSSDENPPMLMPCGHAVSKQSIMKLSKSSSRPFKCPYC
TraesCS7A02G444400	ND	313	256	302	KDCPICLQLLEGDDDLAAWPGCGKPHVFHGAWLESVLVDSKTCPICR
TraesCS7A02G555900	ND	160	76	117	CAICLAEFAEGESMHVLPRCGHAFHVLCIDAWLRTYASFPSC
TraesCS7B02G170500	ND	299	235	275	CVICRVEFEEGESLVALPCKHSYHSDCINQWLQLNKPLPCC
TraesCS7B02G260900	ND	396	339	382	CPVLREQSSDENPPMLMPCGHAVSKQSITKLSKSSSRPFKCPYC

Identifiers	RING type	Protein size (amino acids)	Domain start	Domain end	Incomplete RING domain
TraesCS7D02G355200	ND	396	339	382	CPVLREQSSDENPPMLMPCGHAVSKQSIMKLSKSSSRPFKCPYC
TraesCS7D02G415800c	ND	452	175	223	CACIRPSFTSCAHDFCSSCVARPTSQRQGLRRRAGKMPQHQPADLVPVD
TraesCS7D02G415800d	ND	452	245	292	CAICMETVPGTLKFSVNSCGHAFCSSCVTQYVVLKLDDKVALIECPHP
TraesCS7D02G415800e	ND	452	408	450	CPKCQMFVEKSEGCNYIKCRCGYSFCYRCASELSAQNHYCNKC
TraesCSU02G012700	ND	206	164	199	DMVAGETARRLPNCSHAFHQPCVDKWLFTHGSCPMC
TraesCSU02G080300	ND	293	3	41	CNACWRELEGQAITTTCGHLLCTEDAKKILSNDGACPIC
TraesCSU02G131500	ND	318	160	227	CSICMEPWTSTDEHHICCIPCGHVYGWSCLIRWLRDQLSRNENLKEVHEYYEAKLAEFEASREECMLF
TraesCSU02G147000	ND	137	85	125	CAVCLEEFRGGDVLAHLPCAHRFHWARAVPWVQAASRCPFC
TraesCSU02G177900	ND	161	110	151	CAVCLAEFADGDELRWLPRCRHAFHRPCVDEWLRRQPSCPLY
TraesCSU02G203500	ND	154	103	144	CAACLAEYADGDELRWLPGCRHAFHRPCLDEWLRRRSSCRTS

Supplemental Table 4. 1,238 RING protein genes with their respective chromosomal location along with detail of gene duplication types.

Identifiers	Chromosomal location	Gene duplication type
TraesCS1A02G002000	1A: 1,163,485-1,167,878	WGD/segmental
TraesCS1A02G005700	1A: 3,377,838-3,383,699	WGD/segmental
TraesCS1A02G025700	1A: 12,312,969-12,318,272	WGD/segmental
TraesCS1A02G029800	1A: 13,861,191-13,862,962	WGD/segmental
TraesCS1A02G084200	1A: 69,390,436-69,394,941	WGD/segmental
TraesCS1A02G088100	1A: 76,485,956-76,492,268	WGD/segmental
TraesCS1A02G090900	1A: 82,125,103-82,125,636	WGD/segmental
TraesCS1A02G107600	1A: 105,903,502-105,906,285	WGD/segmental
TraesCS1A02G112600	1A: 114,547,942-114,552,217	WGD/segmental
TraesCS1A02G118800	1A: 127,756,737-127,761,052	WGD/segmental
TraesCS1A02G126200	1A: 151,351,630-151,352,346	WGD/segmental
TraesCS1A02G133300	1A: 186,717,495-186,718,766	WGD/segmental
TraesCS1A02G137400	1A: 222,347,195-222,369,765	WGD/segmental
TraesCS1A02G141900	1A: 241,677,702-241,679,636	WGD/segmental
TraesCS1A02G143300	1A: 246,689,896-246,693,276	WGD/segmental
TraesCS1A02G146700	1A: 253,014,195-253,016,999	Dispersed
TraesCS1A02G158400	1A: 281,887,394-281,895,008	WGD/segmental
TraesCS1A02G162400	1A: 291,561,115-291,565,971	WGD/segmental
TraesCS1A02G169300	1A: 301,895,909-301,899,325	Proximal
TraesCS1A02G177600	1A: 320,479,860-320,494,972	WGD/segmental
TraesCS1A02G192600	1A: 347,922,420-347,923,294	Dispersed
TraesCS1A02G198000	1A: 355,720,785-355,722,734	WGD/segmental
TraesCS1A02G201700	1A: 363,006,018-363,007,340	WGD/segmental
TraesCS1A02G205100	1A: 367,381,194-367,383,318	WGD/segmental
TraesCS1A02G206100	1A: 368,711,528-368,714,085	WGD/segmental
TraesCS1A02G207000	1A: 369,083,037-369,085,306	WGD/segmental
TraesCS1A02G215100	1A: 380,261,964-380,264,364	WGD/segmental
TraesCS1A02G215300	1A: 380,889,644-380,890,816	WGD/segmental
TraesCS1A02G225700	1A: 396,105,135-396,108,083	WGD/segmental
TraesCS1A02G228500	1A: 399,825,242-399,826,597	WGD/segmental
TraesCS1A02G231700	1A: 405,092,099-405,092,734	Tandem
TraesCS1A02G243200	1A: 431,431,127-431,435,660	WGD/segmental
TraesCS1A02G255600	1A: 447,747,680-447,749,014	WGD/segmental
TraesCS1A02G263800	1A: 459,269,857-459,275,619	WGD/segmental
TraesCS1A02G274500	1A: 468,684,277-468,685,156	WGD/segmental
TraesCS1A02G280300	1A: 477,730,922-477,734,715	WGD/segmental
TraesCS1A02G295900	1A: 490,893,557-490,894,966	WGD/segmental
TraesCS1A02G296100	1A: 491,128,110-491,130,587	WGD/segmental

Identifiers	Chromosomal location	Gene duplication type
TraesCS1A02G300400	1A: 495,306,563-495,307,141	WGD/segmental
TraesCS1A02G300500	1A: 495,320,214-495,320,807	WGD/segmental
TraesCS1A02G300600	1A: 495,332,457-495,333,044	WGD/segmental
TraesCS1A02G311600	1A: 503,325,400-503,326,050	WGD/segmental
TraesCS1A02G315400	1A: 506,998,391-507,003,396	WGD/segmental
TraesCS1A02G321800	1A: 512,652,346-512,653,512	WGD/segmental
TraesCS1A02G322000	1A: 512,665,554-512,666,522	Tandem
TraesCS1A02G328300	1A: 517,408,815-517,413,636	WGD/segmental
TraesCS1A02G329600	1A: 518,662,080-518,667,158	WGD/segmental
TraesCS1A02G341400	1A: 531,047,359-531,049,493	WGD/segmental
TraesCS1A02G346900	1A: 533,162,380-533,163,003	WGD/segmental
TraesCS1A02G373200	1A: 549,418,214-549,421,078	WGD/segmental
TraesCS1A02G374800	1A: 549,837,796-549,847,148	WGD/segmental
TraesCS1A02G378100	1A: 551,049,064-551,050,825	WGD/segmental
TraesCS1A02G398300	1A: 563,678,782-563,684,009	WGD/segmental
TraesCS1A02G422300	1A: 577,886,407-577,886,871	WGD/segmental
TraesCS1A02G430800	1A: 583,301,530-583,305,437	WGD/segmental
TraesCS1A02G444900	1A: 592,829,982-592,831,432	WGD/segmental
TraesCS1B02G000900	1B: 1,208,687-1,213,090	WGD/segmental
TraesCS1B02G007900	1B: 4,340,022-4,343,180	WGD/segmental
TraesCS1B02G032600	1B: 15,869,836-15,875,064	WGD/segmental
TraesCS1B02G036900	1B: 17,622,750-17,624,788	WGD/segmental
TraesCS1B02G074500	1B: 57,771,611-57,775,063	Dispersed
TraesCS1B02G101700	1B: 111,367,935-111,372,368	WGD/segmental
TraesCS1B02G107100	1B: 119,099,065-119,104,244	WGD/segmental
TraesCS1B02G118700	1B: 139,478,753-139,479,289	WGD/segmental
TraesCS1B02G118800	1B: 139,479,746-139,481,799	WGD/segmental
TraesCS1B02G123100	1B: 148,471,521-148,478,280	WGD/segmental
TraesCS1B02G128900	1B: 158,532,587-158,533,444	WGD/segmental
TraesCS1B02G129600	1B: 159,914,450-159,924,182	WGD/segmental
TraesCS1B02G132800	1B: 167,715,268-167,719,229	WGD/segmental
TraesCS1B02G138300	1B: 178,510,837-178,515,016	WGD/segmental
TraesCS1B02G144400	1B: 196,144,519-196,148,000	WGD/segmental
TraesCS1B02G145400	1B: 198,759,282-198,759,992	WGD/segmental
TraesCS1B02G152000	1B: 240,485,424-240,504,891	WGD/segmental
TraesCS1B02G156500	1B: 264,342,104-264,342,421	WGD/segmental
TraesCS1B02G164800	1B: 287,840,829-287,843,235	WGD/segmental
TraesCS1B02G174400	1B: 313,080,958-313,101,223	Proximal
TraesCS1B02G179000	1B: 324,601,074-324,606,078	WGD/segmental
TraesCS1B02G187600	1B: 335,972,502-335,975,942	WGD/segmental

Identifiers	Chromosomal location	Gene duplication type
TraesCS1B02G202600	1B: 364,116,115-364,131,523	WGD/segmental
TraesCS1B02G212700	1B: 386,197,303-386,199,115	WGD/segmental
TraesCS1B02G216000	1B: 391,961,114-391,961,860	WGD/segmental
TraesCS1B02G218700	1B: 396,116,888-396,118,916	WGD/segmental
TraesCS1B02G219500	1B: 396,597,678-396,605,441	WGD/segmental
TraesCS1B02G219700	1B: 396,817,609-396,820,173	WGD/segmental
TraesCS1B02G220800	1B: 397,813,703-397,815,919	WGD/segmental
TraesCS1B02G228600	1B: 409,836,678-409,838,495	WGD/segmental
TraesCS1B02G228800	1B: 410,666,971-410,668,781	WGD/segmental
TraesCS1B02G238900	1B: 427,208,267-427,211,226	WGD/segmental
TraesCS1B02G242700	1B: 430,617,097-430,618,340	WGD/segmental
TraesCS1B02G242800	1B: 431,032,971-431,034,101	WGD/segmental
TraesCS1B02G254700	1B: 449,130,167-449,135,097	WGD/segmental
TraesCS1B02G258900	1B: 456,259,382-456,261,621	Dispersed
TraesCS1B02G266300	1B: 468,257,376-468,258,545	WGD/segmental
TraesCS1B02G274400	1B: 481,706,707-481,712,978	WGD/segmental
TraesCS1B02G284000	1B: 492,865,334-492,866,254	WGD/segmental
TraesCS1B02G289200	1B: 504,088,438-504,092,237	WGD/segmental
TraesCS1B02G304900	1B: 527,647,033-527,649,438	WGD/segmental
TraesCS1B02G310500	1B: 533,747,852-533,748,439	WGD/segmental
TraesCS1B02G310700	1B: 533,815,221-533,816,991	WGD/segmental
TraesCS1B02G316300	1B: 540,728,727-540,731,048	WGD/segmental
TraesCS1B02G323200	1B: 548,278,673-548,279,818	WGD/segmental
TraesCS1B02G323300	1B: 548,300,584-548,301,252	WGD/segmental
TraesCS1B02G327300	1B: 553,553,685-553,558,738	WGD/segmental
TraesCS1B02G327600	1B: 553,654,026-553,654,528	Singleton
TraesCS1B02G334400	1B: 561,551,803-561,552,693	WGD/segmental
TraesCS1B02G342100	1B: 570,270,312-570,275,099	WGD/segmental
TraesCS1B02G343100	1B: 571,436,630-571,441,697	WGD/segmental
TraesCS1B02G354100	1B: 584,102,740-584,105,121	WGD/segmental
TraesCS1B02G361000	1B: 589,488,275-589,489,188	WGD/segmental
TraesCS1B02G393400	1B: 626,224,507-626,227,842	WGD/segmental
TraesCS1B02G395000	1B: 626,704,741-626,714,325	WGD/segmental
TraesCS1B02G398900	1B: 630,060,487-630,062,606	WGD/segmental
TraesCS1B02G456800	1B: 671,225,018-671,227,057	WGD/segmental
TraesCS1B02G465300	1B: 676,147,398-676,161,697	WGD/segmental
TraesCS1B02G465500	1B: 676,200,366-676,204,120	Tandem
TraesCS1B02G465700	1B: 676,262,254-676,267,497	WGD/segmental
TraesCS1B02G479700	1B: 687,403,981-687,406,890	WGD/segmental
TraesCS1D02G002400	1D: 568,769-574,727	WGD/segmental

Identifiers	Chromosomal location	Gene duplication type
TraesCS1D02G004000	1D: 2,081,281-2,084,780	WGD/segmental
TraesCS1D02G010300	1D: 5,443,299-5,444,210	WGD/segmental
TraesCS1D02G026100	1D: 10,666,322-10,671,961	WGD/segmental
TraesCS1D02G030300	1D: 11,780,392-11,783,116	WGD/segmental
TraesCS1D02G085400	1D: 69,785,402-69,790,319	WGD/segmental
TraesCS1D02G089400	1D: 75,655,364-75,660,584	WGD/segmental
TraesCS1D02G099500	1D: 86,783,377-86,783,823	WGD/segmental
TraesCS1D02G101400	1D: 89,229,322-89,230,716	Dispersed
TraesCS1D02G103800	1D: 93,123,774-93,130,484	WGD/segmental
TraesCS1D02G111500	1D: 106,955,155-106,959,281	WGD/segmental
TraesCS1D02G112400	1D: 108,110,745-108,111,361	WGD/segmental
TraesCS1D02G114200	1D: 110,337,115-110,341,205	WGD/segmental
TraesCS1D02G119700	1D: 116,547,500-116,549,720	WGD/segmental
TraesCS1D02G127300	1D: 139,904,743-139,905,819	WGD/segmental
TraesCS1D02G128900	1D: 141,945,071-141,947,462	WGD/segmental
TraesCS1D02G133300	1D: 162,050,654-162,065,815	WGD/segmental
TraesCS1D02G138900	1D: 190,763,503-190,763,832	WGD/segmental
TraesCS1D02G141000	1D: 194,221,690-194,223,620	WGD/segmental
TraesCS1D02G142100	1D: 196,608,699-196,611,746	WGD/segmental
TraesCS1D02G144900	1D: 200,093,088-200,095,416	WGD/segmental
TraesCS1D02G155700	1D: 218,974,878-218,981,716	WGD/segmental
TraesCS1D02G159200	1D: 224,570,023-224,575,323	WGD/segmental
TraesCS1D02G167300	1D: 238,329,446-238,332,693	Proximal
TraesCS1D02G176100	1D: 250,416,019-250,430,868	WGD/segmental
TraesCS1D02G201500	1D: 284,245,274-284,247,084	WGD/segmental
TraesCS1D02G205300	1D: 290,705,084-290,706,415	WGD/segmental
TraesCS1D02G208400	1D: 294,405,087-294,407,320	WGD/segmental
TraesCS1D02G209200	1D: 295,274,493-295,280,663	WGD/segmental
TraesCS1D02G209400	1D: 295,402,549-295,405,325	WGD/segmental
TraesCS1D02G210300	1D: 296,359,185-296,364,567	WGD/segmental
TraesCS1D02G217700	1D: 302,925,500-302,927,112	WGD/segmental
TraesCS1D02G217800	1D: 303,292,768-303,294,471	WGD/segmental
TraesCS1D02G226900	1D: 315,541,113-315,544,056	Proximal
TraesCS1D02G230300	1D: 318,497,114-318,498,351	WGD/segmental
TraesCS1D02G230600	1D: 318,722,183-318,723,435	WGD/segmental
TraesCS1D02G243200	1D: 333,487,057-333,491,871	WGD/segmental
TraesCS1D02G254900	1D: 347,300,592-347,301,735	WGD/segmental
TraesCS1D02G263900	1D: 359,720,546-359,725,663	WGD/segmental
TraesCS1D02G274500	1D: 370,129,921-370,130,526	WGD/segmental
TraesCS1D02G279400	1D: 377,187,516-377,190,904	WGD/segmental

Identifiers	Chromosomal location	Gene duplication type
TraesCS1D02G296100	1D: 394,277,936-394,279,732	WGD/segmental
TraesCS1D02G296300	1D: 394,408,370-394,409,737	WGD/segmental
TraesCS1D02G296400	1D: 394,451,608-394,452,186	WGD/segmental
TraesCS1D02G296800	1D: 394,894,904-394,895,551	WGD/segmental
TraesCS1D02G299800	1D: 398,093,791-398,096,850	WGD/segmental
TraesCS1D02G299900	1D: 398,121,569-398,122,144	WGD/segmental
TraesCS1D02G300000	1D: 398,379,698-398,381,500	WGD/segmental
TraesCS1D02G305300	1D: 402,832,365-402,835,491	WGD/segmental
TraesCS1D02G311600	1D: 407,695,879-407,696,526	WGD/segmental
TraesCS1D02G311700	1D: 407,807,908-407,808,570	Tandem
TraesCS1D02G311900	1D: 407,823,302-407,824,296	Tandem
TraesCS1D02G315600	1D: 410,738,774-410,743,828	WGD/segmental
TraesCS1D02G322200	1D: 415,299,482-415,300,384	WGD/segmental
TraesCS1D02G330800	1D: 421,031,358-421,035,935	WGD/segmental
TraesCS1D02G331700	1D: 422,679,247-422,684,168	WGD/segmental
TraesCS1D02G343700	1D: 432,702,081-432,704,434	WGD/segmental
TraesCS1D02G380000	1D: 455,782,623-455,785,126	WGD/segmental
TraesCS1D02G382000	1D: 457,143,495-457,152,610	WGD/segmental
TraesCS1D02G405800	1D: 470,069,856-470,074,840	WGD/segmental
TraesCS1D02G430400	1D: 481,397,743-481,398,201	WGD/segmental
TraesCS1D02G430500	1D: 481,407,728-481,408,189	Tandem
TraesCS1D02G433500	1D: 482,896,065-482,897,873	WGD/segmental
TraesCS1D02G439900	1D: 485,714,192-485,718,393	WGD/segmental
TraesCS1D02G453000	1D: 494,140,669-494,142,864	WGD/segmental
TraesCS2A02G022100	2A: 10,596,264-10,602,559	WGD/segmental
TraesCS2A02G039300	2A: 16,418,247-16,421,651	WGD/segmental
TraesCS2A02G065200	2A: 29,593,791-29,594,126	WGD/segmental
TraesCS2A02G072700	2A: 32,266,421-32,272,626	WGD/segmental
TraesCS2A02G081500	2A: 36,880,311-36,881,178	WGD/segmental
TraesCS2A02G094000	2A: 47,905,745-47,911,177	WGD/segmental
TraesCS2A02G099300	2A: 52,132,592-52,133,693	WGD/segmental
TraesCS2A02G109000	2A: 61,290,402-61,290,893	WGD/segmental
TraesCS2A02G112800	2A: 63,794,863-63,798,396	WGD/segmental
TraesCS2A02G126800	2A: 74,873,687-74,878,327	WGD/segmental
TraesCS2A02G145200	2A: 90,219,319-90,224,636	WGD/segmental
TraesCS2A02G156000	2A: 103,097,590-103,098,291	WGD/segmental
TraesCS2A02G156600	2A: 103,225,423-103,226,293	WGD/segmental
TraesCS2A02G156800	2A: 103,354,531-103,355,459	WGD/segmental
TraesCS2A02G167300	2A: 120,098,452-120,102,342	WGD/segmental
TraesCS2A02G168700	2A: 122,133,159-122,135,251	WGD/segmental

Identifiers	Chromosomal location	Gene duplication type
TraesCS2A02G224300	2A: 223,715,437-223,723,691	WGD/segmental
TraesCS2A02G227400	2A: 240,458,425-240,484,695	WGD/segmental
TraesCS2A02G229600	2A: 261,172,024-261,184,591	WGD/segmental
TraesCS2A02G232200	2A: 275,928,473-275,935,089	WGD/segmental
TraesCS2A02G248400	2A: 371,044,432-371,047,204	WGD/segmental
TraesCS2A02G290100	2A: 499,578,683-499,580,316	WGD/segmental
TraesCS2A02G299000	2A: 514,773,850-514,774,787	WGD/segmental
TraesCS2A02G311800	2A: 535,701,410-535,702,544	WGD/segmental
TraesCS2A02G318200	2A: 543,307,998-543,309,602	WGD/segmental
TraesCS2A02G318300	2A: 543,347,568-543,349,124	WGD/segmental
TraesCS2A02G352900	2A: 593,487,294-593,488,146	WGD/segmental
TraesCS2A02G358400	2A: 602,650,886-602,653,428	WGD/segmental
TraesCS2A02G364100	2A: 607,995,741-608,000,800	WGD/segmental
TraesCS2A02G372600	2A: 615,343,674-615,345,303	WGD/segmental
TraesCS2A02G385400	2A: 631,657,145-631,662,953	WGD/segmental
TraesCS2A02G395100	2A: 646,844,854-646,845,959	WGD/segmental
TraesCS2A02G399700	2A: 652,770,741-652,776,523	WGD/segmental
TraesCS2A02G400200	2A: 653,391,710-653,396,506	WGD/segmental
TraesCS2A02G401400	2A: 655,030,200-655,033,484	WGD/segmental
TraesCS2A02G437200	2A: 688,971,773-688,974,901	WGD/segmental
TraesCS2A02G446100	2A: 695,347,720-695,352,300	WGD/segmental
TraesCS2A02G448700	2A: 698,092,579-698,093,772	WGD/segmental
TraesCS2A02G453200	2A: 702,782,530-702,783,946	WGD/segmental
TraesCS2A02G455400	2A: 704,004,768-704,005,867	WGD/segmental
TraesCS2A02G475800	2A: 716,172,503-716,173,470	WGD/segmental
TraesCS2A02G501100	2A: 729,580,854-729,581,447	WGD/segmental
TraesCS2A02G511100	2A: 735,821,591-735,822,304	WGD/segmental
TraesCS2A02G514400	2A: 738,985,582-738,991,665	WGD/segmental
TraesCS2A02G526800	2A: 746,839,499-746,841,895	Proximal
TraesCS2A02G545900	2A: 754,708,672-754,712,774	WGD/segmental
TraesCS2A02G546000	2A: 755,058,248-755,061,833	Tandem
TraesCS2A02G556200	2A: 760,564,199-760,584,162	WGD/segmental
TraesCS2A02G564000	2A: 764,321,659-764,326,432	WGD/segmental
TraesCS2A02G586800	2A: 777,254,258-777,256,719	WGD/segmental
TraesCS2B02G019500	2B: 9,016,257-9,034,173	WGD/segmental
TraesCS2B02G030700	2B: 14,045,642-14,051,486	WGD/segmental
TraesCS2B02G051700	2B: 25,256,679-25,260,416	WGD/segmental
TraesCS2B02G077400	2B: 42,957,075-42,957,482	WGD/segmental
TraesCS2B02G086700	2B: 48,797,042-48,803,165	WGD/segmental
TraesCS2B02G108800	2B: 69,409,739-69,415,222	WGD/segmental

Identifiers	Chromosomal location	Gene duplication type
TraesCS2B02G115500	2B: 78,830,147-78,831,053	WGD/segmental
TraesCS2B02G126700	2B: 94,936,565-94,937,044	WGD/segmental
TraesCS2B02G131600	2B: 98,303,727-98,307,080	WGD/segmental
TraesCS2B02G144500	2B: 110,519,549-110,521,380	WGD/segmental
TraesCS2B02G148700	2B: 114,761,658-114,763,874	WGD/segmental
TraesCS2B02G162000	2B: 134,998,354-135,004,030	WGD/segmental
TraesCS2B02G170200	2B: 143,135,998-143,140,322	WGD/segmental
TraesCS2B02G181500	2B: 156,589,558-156,590,268	WGD/segmental
TraesCS2B02G182100	2B: 157,243,309-157,243,839	WGD/segmental
TraesCS2B02G182300	2B: 157,283,950-157,284,893	WGD/segmental
TraesCS2B02G193500	2B: 169,864,360-169,865,163	WGD/segmental
TraesCS2B02G195000	2B: 172,175,263-172,175,835	WGD/segmental
TraesCS2B02G250800	2B: 260,753,164-260,759,290	WGD/segmental
TraesCS2B02G251300	2B: 262,899,486-262,906,338	WGD/segmental
TraesCS2B02G255300	2B: 291,583,780-291,619,899	WGD/segmental
TraesCS2B02G274500	2B: 377,783,748-377,790,915	WGD/segmental
TraesCS2B02G306600	2B: 438,458,570-438,460,258	WGD/segmental
TraesCS2B02G315400	2B: 450,764,928-450,765,557	WGD/segmental
TraesCS2B02G328600	2B: 471,218,015-471,219,309	WGD/segmental
TraesCS2B02G336300	2B: 480,412,023-480,413,357	WGD/segmental
TraesCS2B02G336400	2B: 480,807,946-480,809,658	WGD/segmental
TraesCS2B02G370700	2B: 528,967,262-528,968,159	WGD/segmental
TraesCS2B02G377900	2B: 540,806,348-540,808,920	WGD/segmental
TraesCS2B02G382300	2B: 546,201,659-546,207,198	WGD/segmental
TraesCS2B02G389600	2B: 553,610,630-553,614,305	WGD/segmental
TraesCS2B02G402300	2B: 571,140,809-571,146,566	WGD/segmental
TraesCS2B02G413100	2B: 588,887,494-588,888,314	Dispersed
TraesCS2B02G413200	2B: 588,928,147-588,929,511	WGD/segmental
TraesCS2B02G413800	2B: 590,918,735-590,920,136	WGD/segmental
TraesCS2B02G417700	2B: 597,545,202-597,550,951	WGD/segmental
TraesCS2B02G418300	2B: 598,970,343-598,975,131	WGD/segmental
TraesCS2B02G419400	2B: 601,175,355-601,179,411	WGD/segmental
TraesCS2B02G456100	2B: 650,789,377-650,791,283	WGD/segmental
TraesCS2B02G467200	2B: 662,793,846-662,798,312	WGD/segmental
TraesCS2B02G469500	2B: 665,857,902-665,860,311	WGD/segmental
TraesCS2B02G474500	2B: 671,592,280-671,593,835	WGD/segmental
TraesCS2B02G477500	2B: 674,722,350-674,723,350	WGD/segmental
TraesCS2B02G499500	2B: 695,517,441-695,517,977	WGD/segmental
TraesCS2B02G529100	2B: 723,446,610-723,447,203	WGD/segmental
TraesCS2B02G542200	2B: 738,679,498-738,687,119	WGD/segmental

Identifiers	Chromosomal location	Gene duplication type
TraesCS2B02G566600	2B: 757,899,827-757,900,321	WGD/segmental
TraesCS2B02G566700	2B: 757,925,773-757,926,279	Tandem
TraesCS2B02G576800	2B: 765,486,579-765,490,186	WGD/segmental
TraesCS2B02G577000	2B: 765,557,784-765,563,206	WGD/segmental
TraesCS2B02G586500	2B: 773,140,689-773,160,344	WGD/segmental
TraesCS2B02G596600	2B: 779,861,522-779,864,156	WGD/segmental
TraesCS2B02G625300	2B: 798,134,799-798,139,436	WGD/segmental
TraesCS2D02G005100	2D: 2,750,445-2,760,123	WGD/segmental
TraesCS2D02G022900	2D: 10,331,769-10,337,663	WGD/segmental
TraesCS2D02G037600	2D: 13,893,110-13,898,406	WGD/segmental
TraesCS2D02G063300	2D: 27,267,217-27,267,552	WGD/segmental
TraesCS2D02G071800	2D: 30,335,673-30,341,769	WGD/segmental
TraesCS2D02G079500	2D: 33,947,469-33,948,577	WGD/segmental
TraesCS2D02G092600	2D: 44,075,060-44,080,596	WGD/segmental
TraesCS2D02G098400	2D: 50,473,225-50,474,167	WGD/segmental
TraesCS2D02G109100	2D: 60,756,652-60,757,140	WGD/segmental
TraesCS2D02G125100	2D: 73,111,169-73,112,484	WGD/segmental
TraesCS2D02G129300	2D: 75,092,826-75,097,462	WGD/segmental
TraesCS2D02G141100	2D: 83,131,581-83,137,110	WGD/segmental
TraesCS2D02G148900	2D: 90,360,947-90,365,410	WGD/segmental
TraesCS2D02G161800	2D: 106,624,658-106,625,359	WGD/segmental
TraesCS2D02G162400	2D: 106,951,415-106,952,265	WGD/segmental
TraesCS2D02G162600	2D: 106,970,663-106,971,515	WGD/segmental
TraesCS2D02G174800	2D: 118,245,131-118,246,410	WGD/segmental
TraesCS2D02G176300	2D: 119,821,246-119,822,024	WGD/segmental
TraesCS2D02G187100	2D: 131,162,935-131,164,210	Dispersed
TraesCS2D02G230600	2D: 201,439,620-201,447,121	WGD/segmental
TraesCS2D02G232900	2D: 206,679,915-206,686,396	WGD/segmental
TraesCS2D02G233500	2D: 208,537,083-208,542,903	WGD/segmental
TraesCS2D02G235300	2D: 220,957,592-220,978,655	WGD/segmental
TraesCS2D02G250100	2D: 297,598,058-297,600,946	WGD/segmental
TraesCS2D02G288000	2D: 368,935,025-368,936,692	WGD/segmental
TraesCS2D02G296900	2D: 379,726,314-379,726,910	WGD/segmental
TraesCS2D02G310100	2D: 398,415,690-398,416,774	WGD/segmental
TraesCS2D02G315800	2D: 405,275,467-405,276,946	WGD/segmental
TraesCS2D02G315900	2D: 405,399,410-405,401,093	WGD/segmental
TraesCS2D02G351200	2D: 449,524,843-449,525,682	WGD/segmental
TraesCS2D02G357400	2D: 459,333,039-459,335,606	WGD/segmental
TraesCS2D02G361900	2D: 467,240,638-467,245,912	WGD/segmental
TraesCS2D02G368800	2D: 473,273,420-473,276,935	WGD/segmental

Identifiers	Chromosomal location	Gene duplication type
TraesCS2D02G382100	2D: 486,244,713-486,250,602	WGD/segmental
TraesCS2D02G392500	2D: 500,402,213-500,403,498	WGD/segmental
TraesCS2D02G392600	2D: 500,488,663-500,489,484	Dispersed
TraesCS2D02G393400	2D: 502,929,848-502,930,462	WGD/segmental
TraesCS2D02G397200	2D: 508,238,555-508,243,110	WGD/segmental
TraesCS2D02G397800	2D: 509,751,970-509,756,659	WGD/segmental
TraesCS2D02G398700	2D: 511,916,194-511,920,417	WGD/segmental
TraesCS2D02G432800	2D: 544,779,770-544,782,794	WGD/segmental
TraesCS2D02G445000	2D: 555,929,747-555,933,247	WGD/segmental
TraesCS2D02G447900	2D: 557,683,838-557,685,674	WGD/segmental
TraesCS2D02G452800	2D: 562,080,998-562,082,419	WGD/segmental
TraesCS2D02G455800	2D: 563,958,429-563,959,578	WGD/segmental
TraesCS2D02G475900	2D: 578,582,590-578,583,090	WGD/segmental
TraesCS2D02G479100	2D: 580,010,478-580,017,296	WGD/segmental
TraesCS2D02G501500	2D: 595,556,938-595,557,531	WGD/segmental
TraesCS2D02G512500	2D: 603,659,542-603,660,479	WGD/segmental
TraesCS2D02G516000	2D: 607,418,412-607,425,112	WGD/segmental
TraesCS2D02G522200	2D: 610,863,536-610,864,643	WGD/segmental
TraesCS2D02G537500	2D: 619,338,442-619,339,411	Tandem
TraesCS2D02G547100	2D: 623,496,884-623,499,791	WGD/segmental
TraesCS2D02G547300	2D: 623,787,597-623,792,222	WGD/segmental
TraesCS2D02G557400	2D: 630,388,082-630,407,572	WGD/segmental
TraesCS2D02G574200	2D: 638,810,379-638,815,170	WGD/segmental
TraesCS2D02G599700	2D: 650,869,233-650,871,862	WGD/segmental
TraesCS3A02G001800	3A: 1,313,066-1,315,898	WGD/segmental
TraesCS3A02G022400	3A: 12,744,053-12,744,913	WGD/segmental
TraesCS3A02G025800	3A: 13,517,881-13,521,368	WGD/segmental
TraesCS3A02G051000	3A: 27,543,553-27,544,548	WGD/segmental
TraesCS3A02G087900	3A: 56,734,766-56,735,470	WGD/segmental
TraesCS3A02G095400	3A: 60,682,662-60,686,567	WGD/segmental
TraesCS3A02G103000	3A: 66,507,059-66,511,102	WGD/segmental
TraesCS3A02G120100	3A: 93,894,059-93,897,884	WGD/segmental
TraesCS3A02G131400	3A: 107,863,262-107,863,672	WGD/segmental
TraesCS3A02G131500	3A: 107,975,081-107,977,380	WGD/segmental
TraesCS3A02G139700	3A: 118,546,170-118,546,805	Dispersed
TraesCS3A02G140000	3A: 118,587,495-118,588,381	Dispersed
TraesCS3A02G176600	3A: 197,860,295-197,865,187	WGD/segmental
TraesCS3A02G179300	3A: 207,241,844-207,246,036	WGD/segmental
TraesCS3A02G182100	3A: 211,188,157-211,188,997	WGD/segmental
TraesCS3A02G189200	3A: 231,669,384-231,671,185	WGD/segmental

Identifiers	Chromosomal location	Gene duplication type
TraesCS3A02G246900	3A: 464,904,434-464,913,424	WGD/segmental
TraesCS3A02G254400	3A: 476,087,134-476,089,068	WGD/segmental
TraesCS3A02G259800	3A: 482,092,614-482,093,752	WGD/segmental
TraesCS3A02G260500	3A: 483,449,498-483,452,912	WGD/segmental
TraesCS3A02G264000	3A: 487,857,253-487,859,848	WGD/segmental
TraesCS3A02G271500	3A: 500,969,258-500,973,693	WGD/segmental
TraesCS3A02G278900	3A: 508,216,111-508,217,471	WGD/segmental
TraesCS3A02G281400	3A: 510,180,508-510,182,295	WGD/segmental
TraesCS3A02G288900	3A: 517,074,705-517,080,156	WGD/segmental
TraesCS3A02G293400	3A: 525,297,363-525,298,746	WGD/segmental
TraesCS3A02G293500	3A: 525,628,498-525,629,229	WGD/segmental
TraesCS3A02G309200	3A: 547,329,720-547,330,085	Tandem
TraesCS3A02G309400	3A: 547,406,056-547,406,424	Tandem
TraesCS3A02G309500	3A: 547,414,326-547,414,880	Tandem
TraesCS3A02G327900	3A: 572,730,217-572,736,984	WGD/segmental
TraesCS3A02G330900	3A: 575,491,090-575,494,300	WGD/segmental
TraesCS3A02G339000	3A: 585,604,056-585,606,516	WGD/segmental
TraesCS3A02G344600	3A: 594,300,691-594,301,850	WGD/segmental
TraesCS3A02G345400	3A: 594,798,538-594,799,283	WGD/segmental
TraesCS3A02G350000	3A: 598,564,216-598,566,627	WGD/segmental
TraesCS3A02G350500	3A: 598,988,605-598,989,150	WGD/segmental
TraesCS3A02G365100	3A: 614,538,621-614,541,682	WGD/segmental
TraesCS3A02G377100	3A: 626,073,340-626,074,336	WGD/segmental
TraesCS3A02G391400	3A: 639,243,730-639,247,135	WGD/segmental
TraesCS3A02G399700	3A: 646,670,825-646,673,348	WGD/segmental
TraesCS3A02G410600	3A: 654,588,767-654,591,516	WGD/segmental
TraesCS3A02G410900	3A: 655,080,254-655,080,880	WGD/segmental
TraesCS3A02G414400	3A: 657,945,937-657,949,641	WGD/segmental
TraesCS3A02G423800	3A: 666,221,765-666,222,799	WGD/segmental
TraesCS3A02G425600	3A: 667,947,729-667,951,658	WGD/segmental
TraesCS3A02G425900	3A: 668,550,872-668,554,412	WGD/segmental
TraesCS3A02G426100	3A: 668,867,329-668,869,608	WGD/segmental
TraesCS3A02G426200	3A: 669,523,725-669,526,262	Proximal
TraesCS3A02G426400	3A: 669,565,785-669,568,156	WGD/segmental
TraesCS3A02G439700	3A: 682,972,239-682,975,088	WGD/segmental
TraesCS3A02G440000	3A: 683,070,489-683,071,959	WGD/segmental
TraesCS3A02G459400	3A: 696,050,635-696,051,066	WGD/segmental
TraesCS3A02G459700	3A: 696,280,295-696,281,350	WGD/segmental
TraesCS3A02G460600	3A: 696,641,387-696,642,646	WGD/segmental
TraesCS3A02G464800	3A: 699,521,090-699,523,037	WGD/segmental

Identifiers	Chromosomal location	Gene duplication type
TraesCS3A02G467300	3A: 700,726,173-700,737,354	WGD/segmental
TraesCS3A02G491300	3A: 718,399,151-718,401,813	WGD/segmental
TraesCS3A02G503300	3A: 726,103,263-726,108,141	WGD/segmental
TraesCS3A02G503400	3A: 726,114,798-726,119,028	Tandem
TraesCS3A02G521400	3A: 737,994,803-737,995,873	WGD/segmental
TraesCS3B02G000800	3B: 987,268-990,056	WGD/segmental
TraesCS3B02G019200	3B: 8,129,928-8,133,466	WGD/segmental
TraesCS3B02G023000	3B: 9,922,993-9,923,391	WGD/segmental
TraesCS3B02G023300	3B: 10,013,879-10,014,906	Tandem
TraesCS3B02G060000	3B: 31,799,085-31,799,805	WGD/segmental
TraesCS3B02G103300	3B: 70,092,982-70,093,620	WGD/segmental
TraesCS3B02G111000	3B: 77,003,310-77,007,424	WGD/segmental
TraesCS3B02G120600	3B: 90,470,053-90,473,802	WGD/segmental
TraesCS3B02G139600	3B: 126,000,424-126,004,003	WGD/segmental
TraesCS3B02G152100	3B: 143,639,849-143,640,232	WGD/segmental
TraesCS3B02G152400	3B: 144,238,848-144,239,225	Tandem
TraesCS3B02G164600	3B: 162,521,427-162,522,702	WGD/segmental
TraesCS3B02G164700	3B: 162,622,876-162,623,869	Tandem
TraesCS3B02G164800	3B: 162,639,215-162,639,844	WGD/segmental
TraesCS3B02G164900	3B: 162,711,952-162,712,584	WGD/segmental
TraesCS3B02G165000	3B: 162,859,432-162,859,842	WGD/segmental
TraesCS3B02G165100	3B: 163,153,434-163,154,375	WGD/segmental
TraesCS3B02G208200	3B: 244,233,190-244,238,542	WGD/segmental
TraesCS3B02G212200	3B: 251,782,184-251,782,765	WGD/segmental
TraesCS3B02G218400	3B: 261,785,782-261,787,806	WGD/segmental
TraesCS3B02G272400	3B: 438,539,032-438,549,067	WGD/segmental
TraesCS3B02G286400	3B: 458,169,718-458,171,863	WGD/segmental
TraesCS3B02G293600	3B: 472,854,362-472,857,774	WGD/segmental
TraesCS3B02G297400	3B: 476,948,704-476,951,562	WGD/segmental
TraesCS3B02G305200	3B: 490,590,831-490,594,699	WGD/segmental
TraesCS3B02G313100	3B: 503,030,628-503,031,212	WGD/segmental
TraesCS3B02G315100	3B: 506,964,655-506,966,304	WGD/segmental
TraesCS3B02G323600	3B: 523,558,221-523,563,735	WGD/segmental
TraesCS3B02G328200	3B: 529,796,955-529,798,032	WGD/segmental
TraesCS3B02G336200	3B: 542,545,417-542,546,508	WGD/segmental
TraesCS3B02G357400	3B: 567,258,816-567,265,661	WGD/segmental
TraesCS3B02G361000	3B: 572,744,096-572,745,258	WGD/segmental
TraesCS3B02G370700	3B: 582,789,662-582,792,582	WGD/segmental
TraesCS3B02G376500	3B: 592,824,443-592,825,069	WGD/segmental
TraesCS3B02G377500	3B: 594,442,026-594,442,403	WGD/segmental

Identifiers	Chromosomal location	Gene duplication type
TraesCS3B02G382600	3B: 602,364,158-602,367,037	WGD/segmental
TraesCS3B02G383200	3B: 603,374,483-603,375,028	WGD/segmental
TraesCS3B02G394000	3B: 620,823,462-620,824,109	Dispersed
TraesCS3B02G397000	3B: 627,211,213-627,214,210	WGD/segmental
TraesCS3B02G409700	3B: 646,287,974-646,288,742	WGD/segmental
TraesCS3B02G423300	3B: 660,702,688-660,707,046	WGD/segmental
TraesCS3B02G432700	3B: 671,835,516-671,838,058	WGD/segmental
TraesCS3B02G443900	3B: 684,255,024-684,257,818	WGD/segmental
TraesCS3B02G444100	3B: 684,268,262-684,268,894	WGD/segmental
TraesCS3B02G444200	3B: 684,322,145-684,328,134	Tandem
TraesCS3B02G444300	3B: 684,411,009-684,413,212	Tandem
TraesCS3B02G449200	3B: 689,685,718-689,690,284	WGD/segmental
TraesCS3B02G459700	3B: 703,296,035-703,297,327	WGD/segmental
TraesCS3B02G461900	3B: 705,002,395-705,006,306	WGD/segmental
TraesCS3B02G462000	3B: 705,135,930-705,140,149	WGD/segmental
TraesCS3B02G462200	3B: 705,153,774-705,154,953	WGD/segmental
TraesCS3B02G462300	3B: 705,184,728-705,187,057	WGD/segmental
TraesCS3B02G462400	3B: 705,453,157-705,455,060	WGD/segmental
TraesCS3B02G462500	3B: 705,546,009-705,548,565	Tandem
TraesCS3B02G462600	3B: 705,566,012-705,568,494	WGD/segmental
TraesCS3B02G473700	3B: 723,028,055-723,031,193	WGD/segmental
TraesCS3B02G474100	3B: 723,275,343-723,277,695	WGD/segmental
TraesCS3B02G484400	3B: 730,856,852-730,860,633	WGD/segmental
TraesCS3B02G499500	3B: 744,310,559-744,311,881	WGD/segmental
TraesCS3B02G501700	3B: 745,544,703-745,545,662	WGD/segmental
TraesCS3B02G509100	3B: 753,172,433-753,174,558	WGD/segmental
TraesCS3B02G511800	3B: 755,313,307-755,324,469	WGD/segmental
TraesCS3B02G552300	3B: 786,854,303-786,856,956	WGD/segmental
TraesCS3B02G566700	3B: 798,493,084-798,498,246	WGD/segmental
TraesCS3B02G566800	3B: 798,503,308-798,507,673	Tandem
TraesCS3B02G567600	3B: 799,283,130-799,286,404	WGD/segmental
TraesCS3B02G591100	3B: 815,882,719-815,883,531	WGD/segmental
TraesCS3B02G595200	3B: 818,275,834-818,277,509	WGD/segmental
TraesCS3D02G019900	3D: 6,726,405-6,730,004	WGD/segmental
TraesCS3D02G024200	3D: 7,695,031-7,696,076	WGD/segmental
TraesCS3D02G050600	3D: 19,880,521-19,881,887	WGD/segmental
TraesCS3D02G079000	3D: 39,335,597-39,339,838	Singleton
TraesCS3D02G088100	3D: 44,966,972-44,967,619	WGD/segmental
TraesCS3D02G088200	3D: 45,011,792-45,012,430	Tandem
TraesCS3D02G095600	3D: 48,448,172-48,452,581	WGD/segmental

Identifiers	Chromosomal location	Gene duplication type
TraesCS3D02G105300	3D: 57,504,916-57,509,084	WGD/segmental
TraesCS3D02G122200	3D: 79,629,742-79,633,642	WGD/segmental
TraesCS3D02G135000	3D: 94,035,701-94,036,066	WGD/segmental
TraesCS3D02G147100	3D: 111,898,862-111,900,010	WGD/segmental
TraesCS3D02G147200	3D: 111,925,047-111,929,721	Tandem
TraesCS3D02G147400	3D: 111,956,107-111,956,721	WGD/segmental
TraesCS3D02G147500	3D: 111,963,783-111,964,419	Tandem
TraesCS3D02G147600	3D: 111,977,041-111,977,673	WGD/segmental
TraesCS3D02G147700	3D: 112,321,411-112,321,851	WGD/segmental
TraesCS3D02G147800	3D: 112,332,792-112,333,799	WGD/segmental
TraesCS3D02G183800	3D: 169,423,380-169,428,670	WGD/segmental
TraesCS3D02G184200	3D: 169,882,776-169,889,380	WGD/segmental
TraesCS3D02G186800	3D: 173,394,529-173,395,461	WGD/segmental
TraesCS3D02G192700	3D: 182,593,882-182,595,749	WGD/segmental
TraesCS3D02G244600	3D: 339,848,034-339,857,172	WGD/segmental
TraesCS3D02G255300	3D: 356,955,007-356,956,796	WGD/segmental
TraesCS3D02G259900	3D: 361,776,499-361,777,314	WGD/segmental
TraesCS3D02G260700	3D: 363,051,065-363,054,397	WGD/segmental
TraesCS3D02G264100	3D: 365,909,405-365,911,365	WGD/segmental
TraesCS3D02G271100	3D: 376,157,685-376,162,066	WGD/segmental
TraesCS3D02G279200	3D: 386,776,533-386,777,117	WGD/segmental
TraesCS3D02G281400	3D: 389,211,717-389,213,101	WGD/segmental
TraesCS3D02G288700	3D: 397,777,708-397,783,443	WGD/segmental
TraesCS3D02G293200	3D: 403,352,586-403,353,317	WGD/segmental
TraesCS3D02G293300	3D: 403,692,642-403,694,635	Tandem
TraesCS3D02G301500	3D: 415,716,345-415,717,539	WGD/segmental
TraesCS3D02G321400	3D: 434,046,484-434,052,990	WGD/segmental
TraesCS3D02G324300	3D: 437,487,153-437,488,552	WGD/segmental
TraesCS3D02G332600	3D: 444,795,393-444,797,825	WGD/segmental
TraesCS3D02G338100	3D: 451,161,967-451,162,581	WGD/segmental
TraesCS3D02G339500	3D: 452,485,808-452,486,191	WGD/segmental
TraesCS3D02G344000	3D: 455,880,552-455,882,255	WGD/segmental
TraesCS3D02G344500	3D: 456,317,643-456,318,671	WGD/segmental
TraesCS3D02G358300	3D: 471,772,795-471,776,467	WGD/segmental
TraesCS3D02G370200	3D: 483,352,008-483,352,968	WGD/segmental
TraesCS3D02G384200	3D: 499,848,349-499,851,294	WGD/segmental
TraesCS3D02G393800	3D: 508,604,916-508,607,915	WGD/segmental
TraesCS3D02G400900	3D: 515,704,990-515,705,835	Dispersed
TraesCS3D02G405200	3D: 519,667,512-519,670,124	WGD/segmental
TraesCS3D02G405400	3D: 519,713,367-519,714,008	WGD/segmental

Identifiers	Chromosomal location	Gene duplication type
TraesCS3D02G408900	3D: 523,171,127-523,175,712	WGD/segmental
TraesCS3D02G419000	3D: 531,679,581-531,680,736	WGD/segmental
TraesCS3D02G420400	3D: 532,774,096-532,777,673	WGD/segmental
TraesCS3D02G420500	3D: 532,855,081-532,857,500	WGD/segmental
TraesCS3D02G420700	3D: 532,955,970-532,957,164	WGD/segmental
TraesCS3D02G420800	3D: 532,992,190-532,993,532	WGD/segmental
TraesCS3D02G420900	3D: 533,392,721-533,394,779	Tandem
TraesCS3D02G421000	3D: 533,668,357-533,670,774	WGD/segmental
TraesCS3D02G421100	3D: 533,672,905-533,675,482	WGD/segmental
TraesCS3D02G421200	3D: 533,675,918-533,678,372	Tandem
TraesCS3D02G432500	3D: 546,246,197-546,247,634	WGD/segmental
TraesCS3D02G432800	3D: 546,320,621-546,323,078	WGD/segmental
TraesCS3D02G451600	3D: 559,168,083-559,168,892	WGD/segmental
TraesCS3D02G452500	3D: 560,117,391-560,118,560	WGD/segmental
TraesCS3D02G453900	3D: 560,791,883-560,792,878	Tandem
TraesCS3D02G454100	3D: 560,854,586-560,855,653	Tandem
TraesCS3D02G459900	3D: 565,037,624-565,038,154	WGD/segmental
TraesCS3D02G462600	3D: 567,066,110-567,076,170	WGD/segmental
TraesCS3D02G465000	3D: 568,836,712-568,837,491	Tandem
TraesCS3D02G466100	3D: 569,781,286-569,790,562	Tandem
TraesCS3D02G495500	3D: 587,608,156-587,609,671	Dispersed
TraesCS3D02G497200	3D: 588,393,624-588,396,164	WGD/segmental
TraesCS3D02G509900	3D: 595,062,529-595,067,402	WGD/segmental
TraesCS3D02G510000	3D: 595,070,008-595,074,414	Tandem
TraesCS3D02G511300	3D: 596,056,968-596,061,325	WGD/segmental
TraesCS3D02G532300	3D: 608,400,960-608,402,494	WGD/segmental
TraesCS4A02G011000	4A: 6,035,206-6,038,960	WGD/segmental
TraesCS4A02G034600	4A: 26,547,130-26,547,528	WGD/segmental
TraesCS4A02G049500	4A: 40,281,761-40,285,123	WGD/segmental
TraesCS4A02G053400	4A: 45,132,071-45,135,423	WGD/segmental
TraesCS4A02G082400	4A: 85,590,082-85,592,543	WGD/segmental
TraesCS4A02G083500	4A: 87,411,847-87,415,616	WGD/segmental
TraesCS4A02G089200	4A: 95,183,338-95,188,733	WGD/segmental
TraesCS4A02G095800	4A: 103,767,064-103,768,348	WGD/segmental
TraesCS4A02G101400	4A: 113,850,637-113,852,575	WGD/segmental
TraesCS4A02G104900	4A: 118,957,247-118,958,742	WGD/segmental
TraesCS4A02G112500	4A: 137,164,112-137,166,651	WGD/segmental
TraesCS4A02G112900	4A: 137,182,501-137,188,337	WGD/segmental
TraesCS4A02G117900	4A: 144,360,330-144,365,128	WGD/segmental
TraesCS4A02G118100	4A: 144,626,448-144,626,702	WGD/segmental

Identifiers	Chromosomal location	Gene duplication type
TraesCS4A02G128700	4A: 165,712,374-165,712,877	WGD/segmental
TraesCS4A02G129800	4A: 170,156,688-170,158,391	WGD/segmental
TraesCS4A02G137300	4A: 196,614,143-196,614,700	WGD/segmental
TraesCS4A02G137800	4A: 198,183,963-198,184,442	Dispersed
TraesCS4A02G156600	4A: 312,879,555-312,883,445	WGD/segmental
TraesCS4A02G161100	4A: 349,295,917-349,310,580	WGD/segmental
TraesCS4A02G162000	4A: 352,479,696-352,513,698	WGD/segmental
TraesCS4A02G164100	4A: 376,716,802-376,728,979	WGD/segmental
TraesCS4A02G165300	4A: 389,232,569-389,251,601	WGD/segmental
TraesCS4A02G174400	4A: 444,864,195-444,864,716	WGD/segmental
TraesCS4A02G174500	4A: 445,073,127-445,073,582	Tandem
TraesCS4A02G174600	4A: 445,074,297-445,074,930	Tandem
TraesCS4A02G183700	4A: 462,201,506-462,203,307	WGD/segmental
TraesCS4A02G185500	4A: 464,989,108-464,989,569	WGD/segmental
TraesCS4A02G185700	4A: 465,006,166-465,006,874	WGD/segmental
TraesCS4A02G194200	4A: 476,065,837-476,066,397	WGD/segmental
TraesCS4A02G220900	4A: 525,110,014-525,111,081	WGD/segmental
TraesCS4A02G222800	4A: 530,172,937-530,179,589	WGD/segmental
TraesCS4A02G237400	4A: 546,256,193-546,258,841	WGD/segmental
TraesCS4A02G249200	4A: 560,316,953-560,319,838	WGD/segmental
TraesCS4A02G271200	4A: 582,524,004-582,534,805	WGD/segmental
TraesCS4A02G290600	4A: 594,283,028-594,285,283	WGD/segmental
TraesCS4A02G311200	4A: 603,451,110-603,455,621	WGD/segmental
TraesCS4A02G320900	4A: 609,132,923-609,133,711	Tandem
TraesCS4A02G321200	4A: 609,564,312-609,566,158	Tandem
TraesCS4A02G322600	4A: 610,618,336-610,629,297	Dispersed
TraesCS4A02G338900	4A: 620,778,743-620,780,555	Dispersed
TraesCS4A02G352200	4A: 627,813,928-627,816,836	Dispersed
TraesCS4A02G385800	4A: 662,960,203-662,960,796	WGD/segmental
TraesCS4A02G385900	4A: 663,202,975-663,203,526	Tandem
TraesCS4A02G404600	4A: 678,132,568-678,133,956	WGD/segmental
TraesCS4A02G415800	4A: 685,769,874-685,770,656	WGD/segmental
TraesCS4A02G415900	4A: 685,786,304-685,787,686	WGD/segmental
TraesCS4A02G441400	4A: 709,894,411-709,896,222	WGD/segmental
TraesCS4A02G463800	4A: 727,745,837-727,746,292	WGD/segmental
TraesCS4A02G463900	4A: 727,841,328-727,842,252	WGD/segmental
TraesCS4A02G464300	4A: 728,129,360-728,130,502	Tandem
TraesCS4A02G465400	4A: 728,941,731-728,942,431	Tandem
TraesCS4A02G468600	4A: 730,498,317-730,498,777	Tandem
TraesCS4A02G499200	4A: 744,274,734-744,276,074	WGD/segmental

Identifiers	Chromosomal location	Gene duplication type
TraesCS4B02G003100	4B: 1,780,942-1,785,316	WGD/segmental
TraesCS4B02G023600	4B: 17,060,305-17,062,621	WGD/segmental
TraesCS4B02G042900	4B: 30,724,599-30,734,116	WGD/segmental
TraesCS4B02G065500	4B: 58,249,079-58,251,794	WGD/segmental
TraesCS4B02G077700	4B: 74,668,204-74,672,287	WGD/segmental
TraesCS4B02G091300	4B: 94,185,365-94,186,102	Dispersed
TraesCS4B02G093600	4B: 96,186,482-96,193,135	WGD/segmental
TraesCS4B02G099300	4B: 104,740,766-104,741,547	WGD/segmental
TraesCS4B02G121100	4B: 142,697,623-142,699,398	WGD/segmental
TraesCS4B02G134900	4B: 175,563,045-175,564,693	WGD/segmental
TraesCS4B02G142800	4B: 189,081,691-189,082,149	WGD/segmental
TraesCS4B02G143100	4B: 189,151,312-189,151,833	Tandem
TraesCS4B02G149000	4B: 218,576,949-218,588,858	WGD/segmental
TraesCS4B02G151200	4B: 240,190,912-240,212,283	WGD/segmental
TraesCS4B02G162300	4B: 326,093,908-326,095,302	Dispersed
TraesCS4B02G164000	4B: 339,264,774-339,268,659	WGD/segmental
TraesCS4B02G167400	4B: 364,487,881-364,488,360	WGD/segmental
TraesCS4B02G174800	4B: 383,145,124-383,146,176	WGD/segmental
TraesCS4B02G176000	4B: 386,216,452-386,216,916	WGD/segmental
TraesCS4B02G176100	4B: 386,218,273-386,218,737	Tandem
TraesCS4B02G186500	4B: 406,457,919-406,461,341	WGD/segmental
TraesCS4B02G191300	4B: 413,829,480-413,834,394	WGD/segmental
TraesCS4B02G191600	4B: 413,874,314-413,876,393	WGD/segmental
TraesCS4B02G199100	4B: 427,742,579-427,744,076	WGD/segmental
TraesCS4B02G203200	4B: 434,470,727-434,472,874	WGD/segmental
TraesCS4B02G208600	4B: 445,842,921-445,845,979	WGD/segmental
TraesCS4B02G209100	4B: 446,342,814-446,344,246	WGD/segmental
TraesCS4B02G215100	4B: 454,695,599-454,700,622	WGD/segmental
TraesCS4B02G220700	4B: 464,156,491-464,161,145	WGD/segmental
TraesCS4B02G221600	4B: 465,777,826-465,780,475	WGD/segmental
TraesCS4B02G251200	4B: 517,491,609-517,494,849	WGD/segmental
TraesCS4B02G255400	4B: 520,239,492-520,242,190	WGD/segmental
TraesCS4B02G270600	4B: 546,733,304-546,733,756	WGD/segmental
TraesCS4B02G293400	4B: 579,097,605-579,101,980	WGD/segmental
TraesCS4B02G304400	4B: 592,672,777-592,674,053	WGD/segmental
TraesCS4B02G316600	4B: 606,181,272-606,182,758	WGD/segmental
TraesCS4B02G316700	4B: 606,552,922-606,555,581	WGD/segmental
TraesCS4B02G322200	4B: 612,876,650-612,877,884	WGD/segmental
TraesCS4B02G344100	4B: 637,623,712-637,628,173	WGD/segmental
TraesCS4B02G344200	4B: 637,722,285-637,725,028	WGD/segmental

Identifiers	Chromosomal location	Gene duplication type
TraesCS4B02G361300	4B: 650,728,588-650,730,524	Singleton
TraesCS4B02G380900	4B: 660,874,169-660,880,469	Tandem
TraesCS4B02G381000	4B: 660,904,524-660,910,478	Tandem
TraesCS4D02G003000	4D: 1,369,971-1,374,666	WGD/segmental
TraesCS4D02G021200	4D: 9,182,832-9,185,291	WGD/segmental
TraesCS4D02G040200	4D: 18,688,931-18,698,103	WGD/segmental
TraesCS4D02G064500	4D: 39,979,402-39,981,960	WGD/segmental
TraesCS4D02G076100	4D: 50,757,723-50,758,993	WGD/segmental
TraesCS4D02G090400	4D: 65,531,283-65,539,548	WGD/segmental
TraesCS4D02G095800	4D: 71,531,706-71,532,257	WGD/segmental
TraesCS4D02G119100	4D: 100,483,088-100,483,648	WGD/segmental
TraesCS4D02G127900	4D: 112,159,715-112,160,397	WGD/segmental
TraesCS4D02G128100	4D: 112,196,942-112,197,403	WGD/segmental
TraesCS4D02G129700	4D: 115,123,648-115,125,532	WGD/segmental
TraesCS4D02G141100	4D: 125,095,276-125,095,754	WGD/segmental
TraesCS4D02G141200	4D: 125,230,533-125,231,054	Tandem
TraesCS4D02G147800	4D: 140,896,655-140,909,544	WGD/segmental
TraesCS4D02G148400	4D: 147,449,869-147,453,675	WGD/segmental
TraesCS4D02G152900	4D: 189,693,811-189,697,717	WGD/segmental
TraesCS4D02G155700	4D: 208,379,393-208,422,081	WGD/segmental
TraesCS4D02G158400	4D: 221,300,756-221,303,669	WGD/segmental
TraesCS4D02G169900	4D: 295,109,176-295,109,655	WGD/segmental
TraesCS4D02G170000	4D: 295,425,573-295,428,508	Tandem
TraesCS4D02G176700	4D: 307,236,975-307,238,021	WGD/segmental
TraesCS4D02G177900	4D: 309,508,684-309,509,193	WGD/segmental
TraesCS4D02G187600	4D: 326,383,306-326,383,560	WGD/segmental
TraesCS4D02G187800	4D: 326,973,519-326,977,800	WGD/segmental
TraesCS4D02G192500	4D: 335,773,803-335,778,034	WGD/segmental
TraesCS4D02G192800	4D: 335,897,401-335,899,273	WGD/segmental
TraesCS4D02G200100	4D: 346,748,548-346,750,046	WGD/segmental
TraesCS4D02G204100	4D: 351,468,049-351,470,103	WGD/segmental
TraesCS4D02G209300	4D: 360,762,344-360,765,435	WGD/segmental
TraesCS4D02G209500	4D: 360,941,923-360,943,064	WGD/segmental
TraesCS4D02G215600	4D: 369,257,922-369,263,038	WGD/segmental
TraesCS4D02G221000	4D: 378,259,122-378,263,413	WGD/segmental
TraesCS4D02G222000	4D: 379,159,671-379,162,491	WGD/segmental
TraesCS4D02G251200	4D: 419,419,370-419,422,690	WGD/segmental
TraesCS4D02G255100	4D: 423,945,425-423,947,758	WGD/segmental
TraesCS4D02G292000	4D: 463,396,748-463,401,083	WGD/segmental
TraesCS4D02G302600	4D: 470,261,238-470,262,642	WGD/segmental

Identifiers	Chromosomal location	Gene duplication type
TraesCS4D02G313200	4D: 479,380,231-479,381,022	WGD/segmental
TraesCS4D02G313300	4D: 479,411,058-479,412,494	WGD/segmental
TraesCS4D02G318800	4D: 483,041,021-483,041,647	WGD/segmental
TraesCS4D02G339200	4D: 497,128,463-497,133,137	WGD/segmental
TraesCS4D02G339300	4D: 497,150,642-497,160,941	WGD/segmental
TraesCS4D02G354300	4D: 504,197,596-504,200,977	Dispersed
TraesCS4D02G362000	4D: 508,528,075-508,528,350	Dispersed
TraesCS5A02G010000	5A: 6,665,804-6,668,336	WGD/segmental
TraesCS5A02G027500	5A: 22,985,789-22,986,220	WGD/segmental
TraesCS5A02G028000	5A: 23,915,121-23,915,579	WGD/segmental
TraesCS5A02G028200	5A: 24,149,348-24,149,767	WGD/segmental
TraesCS5A02G028300	5A: 24,161,340-24,161,831	WGD/segmental
TraesCS5A02G028400	5A: 24,177,123-24,177,572	Tandem
TraesCS5A02G028500	5A: 24,182,186-24,182,668	Tandem
TraesCS5A02G028600	5A: 24,185,029-24,185,496	WGD/segmental
TraesCS5A02G028700	5A: 24,188,766-24,189,449	WGD/segmental
TraesCS5A02G029200	5A: 25,075,651-25,076,064	WGD/segmental
TraesCS5A02G045900	5A: 41,393,401-41,393,889	WGD/segmental
TraesCS5A02G049400	5A: 45,282,199-45,283,228	WGD/segmental
TraesCS5A02G058000	5A: 55,654,680-55,660,812	WGD/segmental
TraesCS5A02G062400	5A: 67,327,050-67,328,303	WGD/segmental
TraesCS5A02G072600	5A: 83,929,944-83,932,346	WGD/segmental
TraesCS5A02G095000	5A: 132,095,090-132,099,793	WGD/segmental
TraesCS5A02G097000	5A: 136,642,847-136,644,661	WGD/segmental
TraesCS5A02G129900	5A: 291,592,580-291,596,195	WGD/segmental
TraesCS5A02G135100	5A: 305,087,269-305,096,499	WGD/segmental
TraesCS5A02G136900	5A: 308,539,962-308,540,498	WGD/segmental
TraesCS5A02G145800	5A: 321,925,696-321,928,311	WGD/segmental
TraesCS5A02G147800	5A: 325,804,575-325,805,204	WGD/segmental
TraesCS5A02G147900	5A: 325,808,545-325,809,180	WGD/segmental
TraesCS5A02G167100	5A: 356,104,622-356,105,110	WGD/segmental
TraesCS5A02G183200	5A: 382,764,008-382,769,194	WGD/segmental
TraesCS5A02G189000	5A: 391,516,937-391,521,446	WGD/segmental
TraesCS5A02G198500	5A: 403,028,388-403,029,470	WGD/segmental
TraesCS5A02G207100	5A: 417,896,963-417,904,903	WGD/segmental
TraesCS5A02G216000	5A: 432,228,410-432,232,121	WGD/segmental
TraesCS5A02G228400	5A: 444,559,501-444,564,556	WGD/segmental
TraesCS5A02G232200	5A: 447,540,496-447,542,279	WGD/segmental
TraesCS5A02G248100	5A: 461,937,407-461,939,134	WGD/segmental
TraesCS5A02G248300	5A: 462,149,658-462,156,402	WGD/segmental

Identifiers	Chromosomal location	Gene duplication type
TraesCS5A02G253700	5A: 469,888,254-469,888,748	WGD/segmental
TraesCS5A02G271600	5A: 481,906,957-481,907,888	WGD/segmental
TraesCS5A02G282300	5A: 491,292,869-491,295,991	WGD/segmental
TraesCS5A02G283800	5A: 491,742,518-491,743,335	WGD/segmental
TraesCS5A02G308200	5A: 520,680,768-520,698,007	WGD/segmental
TraesCS5A02G308300	5A: 520,729,547-520,730,080	Dispersed
TraesCS5A02G312800	5A: 524,052,048-524,054,463	WGD/segmental
TraesCS5A02G325400	5A: 536,119,168-536,125,403	WGD/segmental
TraesCS5A02G326800	5A: 536,895,738-536,896,639	WGD/segmental
TraesCS5A02G326900	5A: 536,903,936-536,904,481	Tandem
TraesCS5A02G336400	5A: 545,129,131-545,130,038	WGD/segmental
TraesCS5A02G354500	5A: 557,076,709-557,077,548	WGD/segmental
TraesCS5A02G354600	5A: 557,081,716-557,083,099	Tandem
TraesCS5A02G354700	5A: 557,092,539-557,093,342	WGD/segmental
TraesCS5A02G354800	5A: 557,098,773-557,099,753	Proximal
TraesCS5A02G372500	5A: 570,788,586-570,789,059	WGD/segmental
TraesCS5A02G372600	5A: 570,791,269-570,791,939	Dispersed
TraesCS5A02G378600	5A: 575,687,703-575,693,006	WGD/segmental
TraesCS5A02G381800	5A: 579,435,595-579,441,101	WGD/segmental
TraesCS5A02G396000	5A: 591,081,866-591,082,624	WGD/segmental
TraesCS5A02G426200	5A: 611,542,039-611,543,335	WGD/segmental
TraesCS5A02G443000	5A: 623,207,477-623,213,471	WGD/segmental
TraesCS5A02G460200	5A: 640,681,652-640,683,873	WGD/segmental
TraesCS5A02G467700	5A: 645,466,719-645,470,140	WGD/segmental
TraesCS5A02G470400	5A: 647,168,222-647,169,122	WGD/segmental
TraesCS5A02G476600	5A: 651,437,025-651,437,279	WGD/segmental
TraesCS5A02G494500	5A: 663,189,438-663,190,648	Dispersed
TraesCS5A02G512800	5A: 676,889,751-676,894,261	Dispersed
TraesCS5A02G513000	5A: 676,949,097-676,951,841	Dispersed
TraesCS5A02G529500	5A: 688,859,604-688,863,043	Dispersed
TraesCS5A02G547400	5A: 701,298,387-701,304,757	Tandem
TraesCS5A02G547800	5A: 702,132,455-702,136,920	Tandem
TraesCS5B02G007600	5B: 8,875,269-8,877,667	WGD/segmental
TraesCS5B02G026700	5B: 25,667,853-25,668,646	WGD/segmental
TraesCS5B02G049900	5B: 55,343,656-55,344,123	WGD/segmental
TraesCS5B02G054600	5B: 59,791,457-59,792,519	WGD/segmental
TraesCS5B02G065600	5B: 73,828,030-73,833,637	WGD/segmental
TraesCS5B02G069700	5B: 79,766,085-79,767,831	WGD/segmental
TraesCS5B02G078400	5B: 94,258,854-94,261,879	WGD/segmental
TraesCS5B02G090000	5B: 115,654,500-115,655,326	WGD/segmental

Identifiers	Chromosomal location	Gene duplication type
TraesCS5B02G090100	5B: 115,868,578-115,871,863	Tandem
TraesCS5B02G101200	5B: 134,128,878-134,133,174	WGD/segmental
TraesCS5B02G111900	5B: 170,583,165-170,619,236	WGD/segmental
TraesCS5B02G129800	5B: 241,166,521-241,169,379	WGD/segmental
TraesCS5B02G133900	5B: 250,596,193-250,611,251	WGD/segmental
TraesCS5B02G135600	5B: 255,337,327-255,337,875	WGD/segmental
TraesCS5B02G144700	5B: 271,950,733-271,952,774	WGD/segmental
TraesCS5B02G164100	5B: 304,125,075-304,126,125	WGD/segmental
TraesCS5B02G181400	5B: 331,214,226-331,219,451	WGD/segmental
TraesCS5B02G194700	5B: 351,481,468-351,485,888	WGD/segmental
TraesCS5B02G197200	5B: 355,234,216-355,236,212	WGD/segmental
TraesCS5B02G205500	5B: 373,683,757-373,690,913	WGD/segmental
TraesCS5B02G223400	5B: 398,058,622-398,060,284	Dispersed
TraesCS5B02G227200	5B: 403,779,812-403,784,620	WGD/segmental
TraesCS5B02G230500	5B: 407,235,240-407,237,185	WGD/segmental
TraesCS5B02G245800	5B: 426,993,279-426,995,057	WGD/segmental
TraesCS5B02G246000	5B: 427,247,852-427,249,456	WGD/segmental
TraesCS5B02G252600	5B: 435,597,303-435,598,318	WGD/segmental
TraesCS5B02G271900	5B: 457,339,081-457,339,788	WGD/segmental
TraesCS5B02G281700	5B: 466,778,012-466,781,710	WGD/segmental
TraesCS5B02G282300	5B: 467,137,871-467,138,461	WGD/segmental
TraesCS5B02G308500	5B: 491,245,957-491,246,505	WGD/segmental
TraesCS5B02G308600	5B: 491,272,713-491,273,246	Tandem
TraesCS5B02G308700	5B: 491,280,046-491,280,594	WGD/segmental
TraesCS5B02G313800	5B: 496,267,264-496,269,755	WGD/segmental
TraesCS5B02G325800	5B: 510,293,933-510,300,065	WGD/segmental
TraesCS5B02G335400	5B: 518,570,570-518,571,885	WGD/segmental
TraesCS5B02G356800	5B: 536,384,212-536,385,111	WGD/segmental
TraesCS5B02G356900	5B: 536,476,323-536,476,925	WGD/segmental
TraesCS5B02G357000	5B: 536,505,515-536,509,118	WGD/segmental
TraesCS5B02G373000	5B: 550,844,202-550,848,058	WGD/segmental
TraesCS5B02G374600	5B: 551,739,643-551,740,400	WGD/segmental
TraesCS5B02G374700	5B: 551,798,729-551,799,396	Tandem
TraesCS5B02G382100	5B: 560,391,754-560,397,339	WGD/segmental
TraesCS5B02G385300	5B: 564,988,305-564,994,038	WGD/segmental
TraesCS5B02G400800	5B: 577,659,498-577,660,923	WGD/segmental
TraesCS5B02G428100	5B: 603,984,864-603,985,472	WGD/segmental
TraesCS5B02G442500	5B: 615,711,684-615,712,703	WGD/segmental
TraesCS5B02G447600	5B: 618,470,171-618,474,409	WGD/segmental
TraesCS5B02G469800	5B: 643,305,754-643,308,063	WGD/segmental

Identifiers	Chromosomal location	Gene duplication type
TraesCS5B02G480100	5B: 652,632,607-652,635,206	WGD/segmental
TraesCS5B02G483000	5B: 655,121,627-655,122,554	WGD/segmental
TraesCS5B02G489500	5B: 659,411,555-659,411,809	WGD/segmental
TraesCS5B02G519400	5B: 682,176,793-682,179,882	WGD/segmental
TraesCS5B02G522800	5B: 684,190,037-684,190,636	WGD/segmental
TraesCS5B02G548200	5B: 700,917,011-700,918,440	WGD/segmental
TraesCS5B02G555800	5B: 704,840,238-704,851,047	Dispersed
TraesCS5B02G557600	5B: 705,319,431-705,321,545	WGD/segmental
TraesCS5B02G558000	5B: 705,566,546-705,567,603	Tandem
TraesCS5D02G014800	5D: 8,492,429-8,495,051	WGD/segmental
TraesCS5D02G035300	5D: 34,445,628-34,446,364	WGD/segmental
TraesCS5D02G035700	5D: 35,223,135-35,223,596	WGD/segmental
TraesCS5D02G035800	5D: 35,285,941-35,286,348	WGD/segmental
TraesCS5D02G035900	5D: 35,294,300-35,305,719	WGD/segmental
TraesCS5D02G036200	5D: 35,499,405-35,499,812	Proximal
TraesCS5D02G036300	5D: 35,550,908-35,551,405	Proximal
TraesCS5D02G036400	5D: 35,553,672-35,554,148	Proximal
TraesCS5D02G036500	5D: 35,560,974-35,561,432	WGD/segmental
TraesCS5D02G055800	5D: 52,264,816-52,265,286	WGD/segmental
TraesCS5D02G060200	5D: 56,922,062-56,923,096	WGD/segmental
TraesCS5D02G069800	5D: 67,861,587-67,867,537	WGD/segmental
TraesCS5D02G073600	5D: 72,871,392-72,872,645	WGD/segmental
TraesCS5D02G085300	5D: 90,265,246-90,268,255	WGD/segmental
TraesCS5D02G096000	5D: 105,619,667-105,620,862	WGD/segmental
TraesCS5D02G096500	5D: 106,940,216-106,940,728	WGD/segmental
TraesCS5D02G096600	5D: 107,010,215-107,013,879	Tandem
TraesCS5D02G107600	5D: 121,076,624-121,086,264	WGD/segmental
TraesCS5D02G122000	5D: 175,775,498-175,788,129	WGD/segmental
TraesCS5D02G137400	5D: 218,557,426-218,561,076	WGD/segmental
TraesCS5D02G139400	5D: 222,419,558-222,429,880	WGD/segmental
TraesCS5D02G144900	5D: 231,569,235-231,569,687	WGD/segmental
TraesCS5D02G146400	5D: 233,035,135-233,038,207	WGD/segmental
TraesCS5D02G171400	5D: 268,517,194-268,518,048	WGD/segmental
TraesCS5D02G188300	5D: 290,386,100-290,391,792	WGD/segmental
TraesCS5D02G202000	5D: 306,198,113-306,203,043	WGD/segmental
TraesCS5D02G204500	5D: 309,694,008-309,697,133	WGD/segmental
TraesCS5D02G213300	5D: 322,382,978-322,390,523	WGD/segmental
TraesCS5D02G223800	5D: 331,572,260-331,575,937	WGD/segmental
TraesCS5D02G235800	5D: 344,539,254-344,540,957	WGD/segmental
TraesCS5D02G239200	5D: 347,361,848-347,366,354	Dispersed

Identifiers	Chromosomal location	Gene duplication type
TraesCS5D02G254900	5D: 361,403,532-361,405,045	WGD/segmental
TraesCS5D02G255200	5D: 361,649,578-361,651,275	WGD/segmental
TraesCS5D02G279300	5D: 381,407,190-381,408,116	WGD/segmental
TraesCS5D02G289400	5D: 388,100,580-388,104,112	WGD/segmental
TraesCS5D02G291000	5D: 388,794,132-388,794,734	WGD/segmental
TraesCS5D02G315200	5D: 409,454,528-409,455,076	WGD/segmental
TraesCS5D02G315300	5D: 409,523,271-409,542,867	WGD/segmental
TraesCS5D02G319600	5D: 412,311,925-412,314,298	WGD/segmental
TraesCS5D02G331600	5D: 422,697,010-422,702,597	WGD/segmental
TraesCS5D02G333100	5D: 423,463,576-423,464,220	WGD/segmental
TraesCS5D02G333200	5D: 423,468,788-423,469,423	WGD/segmental
TraesCS5D02G341100	5D: 429,304,293-429,305,209	WGD/segmental
TraesCS5D02G361800	5D: 440,938,427-440,939,298	WGD/segmental
TraesCS5D02G361900	5D: 440,947,896-440,948,498	WGD/segmental
TraesCS5D02G362000	5D: 440,961,355-440,962,355	WGD/segmental
TraesCS5D02G362100	5D: 440,965,632-440,966,195	Proximal
TraesCS5D02G380200	5D: 450,683,865-450,688,789	WGD/segmental
TraesCS5D02G382000	5D: 451,954,962-451,955,610	WGD/segmental
TraesCS5D02G382100	5D: 451,970,069-451,970,697	Tandem
TraesCS5D02G382200	5D: 451,972,804-451,973,574	Tandem
TraesCS5D02G388500	5D: 457,992,308-457,997,552	WGD/segmental
TraesCS5D02G391100	5D: 459,862,603-459,867,935	WGD/segmental
TraesCS5D02G405300	5D: 470,376,887-470,378,075	WGD/segmental
TraesCS5D02G434200	5D: 489,806,683-489,807,550	WGD/segmental
TraesCS5D02G446700	5D: 496,951,145-496,952,161	WGD/segmental
TraesCS5D02G450400	5D: 498,769,869-498,776,200	WGD/segmental
TraesCS5D02G471300	5D: 512,303,138-512,305,330	WGD/segmental
TraesCS5D02G472700	5D: 513,314,645-513,316,781	Dispersed
TraesCS5D02G480800	5D: 518,919,635-518,922,993	WGD/segmental
TraesCS5D02G483100	5D: 520,114,239-520,115,074	WGD/segmental
TraesCS5D02G489900	5D: 524,917,361-524,917,615	WGD/segmental
TraesCS5D02G518700	5D: 539,969,460-539,972,258	WGD/segmental
TraesCS5D02G521500	5D: 542,529,978-542,530,631	WGD/segmental
TraesCS5D02G535300	5D: 549,308,379-549,309,601	WGD/segmental
TraesCS5D02G551700	5D: 557,504,352-557,505,688	WGD/segmental
TraesCS5D02G553700	5D: 558,284,186-558,295,311	Dispersed
TraesCS5D02G564400	5D: 563,339,892-563,340,719	Proximal
TraesCS6A02G001000	6A: 637,025-639,371	WGD/segmental
TraesCS6A02G023700	6A: 11,790,646-11,791,549	WGD/segmental
TraesCS6A02G050800	6A: 26,529,406-26,530,524	WGD/segmental

Identifiers	Chromosomal location	Gene duplication type
TraesCS6A02G055100	6A: 28,643,671-28,644,597	WGD/segmental
TraesCS6A02G084400	6A: 53,071,982-53,075,458	WGD/segmental
TraesCS6A02G095500	6A: 62,130,061-62,130,723	WGD/segmental
TraesCS6A02G095600	6A: 62,717,290-62,717,841	WGD/segmental
TraesCS6A02G096200	6A: 63,584,630-63,599,250	WGD/segmental
TraesCS6A02G117900	6A: 88,763,573-88,774,341	WGD/segmental
TraesCS6A02G120700	6A: 92,575,958-92,577,116	WGD/segmental
TraesCS6A02G121400	6A: 93,797,131-93,801,770	WGD/segmental
TraesCS6A02G122800	6A: 96,451,483-96,453,055	WGD/segmental
TraesCS6A02G145300	6A: 123,455,701-123,460,492	WGD/segmental
TraesCS6A02G172100	6A: 183,218,385-183,218,990	Dispersed
TraesCS6A02G202400	6A: 338,191,609-338,194,281	Dispersed
TraesCS6A02G226100	6A: 425,741,848-425,746,917	Dispersed
TraesCS6A02G227300	6A: 428,067,382-428,074,157	WGD/segmental
TraesCS6A02G236100	6A: 445,581,013-445,582,254	WGD/segmental
TraesCS6A02G239000	6A: 448,650,675-448,651,220	WGD/segmental
TraesCS6A02G255700	6A: 473,742,844-473,747,560	WGD/segmental
TraesCS6A02G256500	6A: 477,130,508-477,134,778	WGD/segmental
TraesCS6A02G258800	6A: 481,082,445-481,083,068	WGD/segmental
TraesCS6A02G259200	6A: 482,079,152-482,080,476	WGD/segmental
TraesCS6A02G261400	6A: 484,848,038-484,848,550	WGD/segmental
TraesCS6A02G262500	6A: 485,752,114-485,753,486	WGD/segmental
TraesCS6A02G264300	6A: 487,770,179-487,771,354	WGD/segmental
TraesCS6A02G274400	6A: 501,181,099-501,183,684	WGD/segmental
TraesCS6A02G288800	6A: 520,845,844-520,847,190	WGD/segmental
TraesCS6A02G290900	6A: 522,456,856-522,457,326	WGD/segmental
TraesCS6A02G291000	6A: 522,481,346-522,481,729	Tandem
TraesCS6A02G298300	6A: 531,523,542-531,526,071	WGD/segmental
TraesCS6A02G304300	6A: 537,313,154-537,315,503	WGD/segmental
TraesCS6A02G308200	6A: 542,932,915-542,936,395	WGD/segmental
TraesCS6A02G315600	6A: 552,207,978-552,209,195	WGD/segmental
TraesCS6A02G326100	6A: 559,421,439-559,427,733	WGD/segmental
TraesCS6A02G330700	6A: 563,131,174-563,132,906	WGD/segmental
TraesCS6A02G346000	6A: 579,145,887-579,150,021	WGD/segmental
TraesCS6A02G349700	6A: 581,891,314-581,894,906	WGD/segmental
TraesCS6A02G352200	6A: 584,909,684-584,912,377	WGD/segmental
TraesCS6A02G361600	6A: 591,950,379-591,952,423	WGD/segmental
TraesCS6A02G362000	6A: 592,030,560-592,035,268	WGD/segmental
TraesCS6A02G375500	6A: 597,831,273-597,837,358	WGD/segmental
TraesCS6A02G397900	6A: 609,031,265-609,032,346	WGD/segmental

Identifiers	Chromosomal location	Gene duplication type
TraesCS6A02G399600	6A: 609,379,672-609,383,684	WGD/segmental
TraesCS6A02G405400	6A: 611,410,194-611,416,350	WGD/segmental
TraesCS6A02G420200	6A: 617,097,319-617,097,843	WGD/segmental
TraesCS6B02G005200	6B: 3,881,179-3,883,043	WGD/segmental
TraesCS6B02G032400	6B: 18,935,971-18,936,843	WGD/segmental
TraesCS6B02G067200	6B: 45,542,058-45,543,640	WGD/segmental
TraesCS6B02G118100	6B: 112,905,340-112,910,081	WGD/segmental
TraesCS6B02G123500	6B: 118,215,105-118,215,804	WGD/segmental
TraesCS6B02G123700	6B: 118,851,638-118,857,514	Tandem
TraesCS6B02G124600	6B: 119,818,964-119,823,596	WGD/segmental
TraesCS6B02G146100	6B: 146,635,998-146,648,143	WGD/segmental
TraesCS6B02G149100	6B: 149,876,709-149,877,914	WGD/segmental
TraesCS6B02G149700	6B: 150,832,055-150,836,496	WGD/segmental
TraesCS6B02G151000	6B: 152,107,612-152,109,117	WGD/segmental
TraesCS6B02G164200	6B: 171,477,774-171,478,641	Dispersed
TraesCS6B02G173500	6B: 187,838,991-187,843,501	WGD/segmental
TraesCS6B02G178500	6B: 198,448,229-198,449,275	WGD/segmental
TraesCS6B02G198800	6B: 237,731,594-237,734,398	WGD/segmental
TraesCS6B02G254400	6B: 457,419,257-457,425,431	WGD/segmental
TraesCS6B02G255500	6B: 459,560,720-459,565,834	WGD/segmental
TraesCS6B02G264500	6B: 476,110,186-476,111,002	WGD/segmental
TraesCS6B02G266600	6B: 479,299,609-479,300,813	WGD/segmental
TraesCS6B02G268900	6B: 483,603,346-483,608,665	WGD/segmental
TraesCS6B02G269900	6B: 485,869,283-485,874,164	WGD/segmental
TraesCS6B02G274100	6B: 495,424,584-495,438,618	Dispersed
TraesCS6B02G286500	6B: 516,475,910-516,476,825	WGD/segmental
TraesCS6B02G288700	6B: 519,813,378-519,814,290	WGD/segmental
TraesCS6B02G289700	6B: 521,060,464-521,061,558	WGD/segmental
TraesCS6B02G291300	6B: 523,879,963-523,883,188	WGD/segmental
TraesCS6B02G296000	6B: 531,790,670-531,797,176	Dispersed
TraesCS6B02G301900	6B: 541,151,729-541,154,548	WGD/segmental
TraesCS6B02G310500	6B: 555,855,343-555,859,616	WGD/segmental
TraesCS6B02G318600	6B: 566,342,435-566,343,733	WGD/segmental
TraesCS6B02G321500	6B: 569,387,415-569,387,879	WGD/segmental
TraesCS6B02G327800	6B: 577,365,017-577,367,692	WGD/segmental
TraesCS6B02G332400	6B: 583,626,562-583,629,091	WGD/segmental
TraesCS6B02G336900	6B: 593,263,071-593,265,921	WGD/segmental
TraesCS6B02G346000	6B: 609,854,033-609,855,241	WGD/segmental
TraesCS6B02G356400	6B: 624,795,560-624,801,969	WGD/segmental
TraesCS6B02G361600	6B: 632,966,867-632,968,058	WGD/segmental

Identifiers	Chromosomal location	Gene duplication type
TraesCS6B02G378600	6B: 653,221,446-653,223,255	WGD/segmental
TraesCS6B02G379400	6B: 653,882,038-653,885,788	Tandem
TraesCS6B02G382800	6B: 657,943,744-657,947,679	WGD/segmental
TraesCS6B02G385100	6B: 659,934,623-659,936,852	WGD/segmental
TraesCS6B02G395000	6B: 670,286,165-670,288,165	WGD/segmental
TraesCS6B02G413100	6B: 686,415,259-686,422,572	WGD/segmental
TraesCS6B02G437700	6B: 704,449,077-704,449,925	WGD/segmental
TraesCS6B02G439800	6B: 705,158,813-705,162,881	WGD/segmental
TraesCS6B02G449700	6B: 708,779,732-708,780,594	WGD/segmental
TraesCS6B02G449800	6B: 708,780,939-708,787,235	WGD/segmental
TraesCS6B02G467800	6B: 717,596,249-717,597,136	WGD/segmental
TraesCS6D02G005100	6D: 2,272,280-2,274,449	WGD/segmental
TraesCS6D02G027000	6D: 10,332,063-10,332,587	WGD/segmental
TraesCS6D02G039100	6D: 16,185,579-16,188,885	Singleton
TraesCS6D02G086300	6D: 51,778,503-51,779,057	WGD/segmental
TraesCS6D02G087300	6D: 52,694,298-52,704,440	WGD/segmental
TraesCS6D02G107900	6D: 72,017,674-72,026,514	WGD/segmental
TraesCS6D02G110700	6D: 77,172,547-77,173,785	WGD/segmental
TraesCS6D02G111500	6D: 78,078,909-78,083,972	WGD/segmental
TraesCS6D02G113000	6D: 79,819,154-79,820,860	WGD/segmental
TraesCS6D02G134300	6D: 102,122,428-102,127,013	WGD/segmental
TraesCS6D02G140100	6D: 109,275,512-109,276,582	WGD/segmental
TraesCS6D02G160300	6D: 138,384,252-138,390,390	WGD/segmental
TraesCS6D02G207700	6D: 292,964,212-292,970,120	WGD/segmental
TraesCS6D02G209000	6D: 295,271,089-295,276,039	WGD/segmental
TraesCS6D02G218600	6D: 308,879,140-308,882,081	WGD/segmental
TraesCS6D02G221300	6D: 312,068,804-312,069,735	WGD/segmental
TraesCS6D02G236900	6D: 334,342,468-334,347,300	WGD/segmental
TraesCS6D02G237700	6D: 336,825,028-336,829,242	WGD/segmental
TraesCS6D02G240000	6D: 341,116,809-341,117,426	WGD/segmental
TraesCS6D02G240300	6D: 341,998,709-341,999,703	WGD/segmental
TraesCS6D02G242100	6D: 344,299,612-344,300,520	WGD/segmental
TraesCS6D02G243200	6D: 345,302,292-345,303,392	WGD/segmental
TraesCS6D02G250600	6D: 354,368,315-354,370,771	WGD/segmental
TraesCS6D02G254700	6D: 359,523,384-359,525,961	WGD/segmental
TraesCS6D02G262500	6D: 370,795,779-370,799,059	WGD/segmental
TraesCS6D02G272200	6D: 380,996,364-380,996,831	WGD/segmental
TraesCS6D02G272300	6D: 381,004,094-381,004,474	Tandem
TraesCS6D02G278100	6D: 386,441,391-386,444,062	WGD/segmental
TraesCS6D02G283700	6D: 390,833,433-390,836,180	WGD/segmental

Identifiers	Chromosomal location	Gene duplication type
TraesCS6D02G287400	6D: 396,607,241-396,610,410	WGD/segmental
TraesCS6D02G294800	6D: 405,649,840-405,651,457	WGD/segmental
TraesCS6D02G305800	6D: 414,475,164-414,481,044	WGD/segmental
TraesCS6D02G309800	6D: 420,014,104-420,015,971	WGD/segmental
TraesCS6D02G328700	6D: 433,285,299-433,289,485	WGD/segmental
TraesCS6D02G332200	6D: 434,512,436-434,516,017	WGD/segmental
TraesCS6D02G334900	6D: 436,715,643-436,716,974	WGD/segmental
TraesCS6D02G344900	6D: 445,158,600-445,160,722	WGD/segmental
TraesCS6D02G345700	6D: 445,493,651-445,498,305	WGD/segmental
TraesCS6D02G360000	6D: 451,907,708-451,913,442	WGD/segmental
TraesCS6D02G382100	6D: 462,181,729-462,182,543	WGD/segmental
TraesCS6D02G382200	6D: 462,196,869-462,197,930	Tandem
TraesCS6D02G383800	6D: 462,536,412-462,540,932	WGD/segmental
TraesCS6D02G389000	6D: 464,830,244-464,831,016	WGD/segmental
TraesCS6D02G389100	6D: 464,832,615-464,838,474	WGD/segmental
TraesCS6D02G405300	6D: 472,568,340-472,568,969	WGD/segmental
TraesCS7A02G005100	7A: 2,693,834-2,700,660	Dispersed
TraesCS7A02G023300	7A: 9,318,501-9,319,158	WGD/segmental
TraesCS7A02G024600	7A: 9,719,138-9,720,574	WGD/segmental
TraesCS7A02G025100	7A: 9,997,094-9,998,219	WGD/segmental
TraesCS7A02G025200	7A: 10,016,989-10,017,809	Tandem
TraesCS7A02G129900	7A: 83,635,256-83,636,344	WGD/segmental
TraesCS7A02G144900	7A: 96,192,239-96,193,343	WGD/segmental
TraesCS7A02G152600	7A: 105,862,947-105,864,788	WGD/segmental
TraesCS7A02G156900	7A: 109,825,167-109,826,129	WGD/segmental
TraesCS7A02G169600	7A: 126,115,403-126,116,638	WGD/segmental
TraesCS7A02G176700	7A: 130,090,347-130,092,250	WGD/segmental
TraesCS7A02G189900	7A: 147,068,788-147,071,706	WGD/segmental
TraesCS7A02G190800	7A: 148,656,416-148,658,383	WGD/segmental
TraesCS7A02G205000	7A: 167,163,879-167,164,382	WGD/segmental
TraesCS7A02G210400	7A: 173,414,634-173,415,392	WGD/segmental
TraesCS7A02G211800	7A: 174,890,227-174,891,664	WGD/segmental
TraesCS7A02G212900	7A: 176,417,860-176,422,770	WGD/segmental
TraesCS7A02G220000	7A: 188,024,576-188,030,907	WGD/segmental
TraesCS7A02G228200	7A: 198,665,505-198,671,133	WGD/segmental
TraesCS7A02G247700	7A: 228,481,855-228,490,263	WGD/segmental
TraesCS7A02G253700	7A: 239,544,563-239,546,013	WGD/segmental
TraesCS7A02G256500	7A: 245,469,855-245,472,027	WGD/segmental
TraesCS7A02G257600	7A: 247,251,565-247,252,362	WGD/segmental
TraesCS7A02G268500	7A: 271,794,716-271,800,048	WGD/segmental

Identifiers	Chromosomal location	Gene duplication type
TraesCS7A02G271500	7A: 284,759,088-284,762,032	WGD/segmental
TraesCS7A02G273000	7A: 286,818,014-286,819,605	WGD/segmental
TraesCS7A02G296500	7A: 394,834,135-394,836,695	WGD/segmental
TraesCS7A02G300000	7A: 414,184,204-414,185,460	WGD/segmental
TraesCS7A02G308900	7A: 436,891,632-436,893,799	WGD/segmental
TraesCS7A02G325800	7A: 473,909,825-473,913,688	WGD/segmental
TraesCS7A02G340400	7A: 498,938,748-498,945,314	WGD/segmental
TraesCS7A02G346400	7A: 509,463,143-509,463,592	WGD/segmental
TraesCS7A02G346500	7A: 509,468,569-509,469,359	WGD/segmental
TraesCS7A02G346700	7A: 509,526,319-509,526,786	WGD/segmental
TraesCS7A02G346800	7A: 509,650,793-509,651,251	WGD/segmental
TraesCS7A02G346900	7A: 509,759,955-509,760,413	Tandem
TraesCS7A02G347000	7A: 509,814,511-509,815,041	Tandem
TraesCS7A02G347100	7A: 509,954,978-509,955,535	WGD/segmental
TraesCS7A02G347200	7A: 510,146,429-510,148,819	WGD/segmental
TraesCS7A02G347700	7A: 510,468,942-510,469,457	WGD/segmental
TraesCS7A02G347800	7A: 510,495,076-510,496,415	WGD/segmental
TraesCS7A02G347900	7A: 510,551,967-510,552,973	WGD/segmental
TraesCS7A02G348000	7A: 510,732,532-510,733,080	Tandem
TraesCS7A02G348100	7A: 510,824,960-510,825,884	WGD/segmental
TraesCS7A02G348200	7A: 510,905,982-510,907,349	WGD/segmental
TraesCS7A02G352100	7A: 515,226,452-515,227,559	Dispersed
TraesCS7A02G372900	7A: 546,046,166-546,046,816	WGD/segmental
TraesCS7A02G373200	7A: 546,343,203-546,343,856	Tandem
TraesCS7A02G381200	7A: 556,161,901-556,168,895	WGD/segmental
TraesCS7A02G381300	7A: 556,170,579-556,175,576	WGD/segmental
TraesCS7A02G391700	7A: 567,587,282-567,588,737	WGD/segmental
TraesCS7A02G420500	7A: 611,837,718-611,841,545	WGD/segmental
TraesCS7A02G423300	7A: 615,374,562-615,376,407	Dispersed
TraesCS7A02G428300	7A: 621,582,917-621,586,740	WGD/segmental
TraesCS7A02G441900	7A: 635,860,205-635,861,765	WGD/segmental
TraesCS7A02G442100	7A: 636,126,771-636,128,118	WGD/segmental
TraesCS7A02G442300	7A: 636,628,901-636,630,345	Tandem
TraesCS7A02G458100	7A: 652,922,228-652,922,731	Dispersed
TraesCS7A02G463200	7A: 659,552,963-659,557,530	WGD/segmental
TraesCS7A02G478200	7A: 671,419,642-671,420,545	WGD/segmental
TraesCS7A02G486700	7A: 677,694,300-677,697,668	WGD/segmental
TraesCS7A02G503800	7A: 692,331,413-692,331,949	WGD/segmental
TraesCS7A02G524600	7A: 706,905,660-706,912,181	WGD/segmental
TraesCS7A02G526000	7A: 708,229,387-708,231,828	WGD/segmental

Identifiers	Chromosomal location	Gene duplication type
TraesCS7A02G534800	7A: 712,769,538-712,771,848	WGD/segmental
TraesCS7A02G551400	7A: 725,033,817-725,034,863	WGD/segmental
TraesCS7A02G555800	7A: 727,532,471-727,533,330	WGD/segmental
TraesCS7A02G556000	7A: 727,583,730-727,584,326	WGD/segmental
TraesCS7A02G556100	7A: 727,710,478-727,710,942	Tandem
TraesCS7A02G556200	7A: 727,721,797-727,722,420	WGD/segmental
TraesCS7A02G556400	7A: 727,773,646-727,774,272	WGD/segmental
TraesCS7A02G556500	7A: 727,788,001-727,788,624	Tandem
TraesCS7A02G556600	7A: 727,800,887-727,801,372	Proximal
TraesCS7A02G556700	7A: 727,832,938-727,833,751	Dispersed
TraesCS7A02G563700	7A: 733,575,844-733,576,355	Dispersed
TraesCS7B02G029900	7B: 28,505,596-28,506,690	WGD/segmental
TraesCS7B02G047200	7B: 46,886,901-46,888,997	WGD/segmental
TraesCS7B02G056400	7B: 59,311,296-59,312,972	WGD/segmental
TraesCS7B02G061100	7B: 63,768,023-63,769,356	WGD/segmental
TraesCS7B02G074800	7B: 84,379,017-84,380,233	WGD/segmental
TraesCS7B02G081700	7B: 92,062,367-92,063,611	WGD/segmental
TraesCS7B02G094900	7B: 108,726,864-108,729,838	WGD/segmental
TraesCS7B02G095700	7B: 110,308,025-110,309,990	WGD/segmental
TraesCS7B02G112200	7B: 128,763,720-128,764,214	WGD/segmental
TraesCS7B02G112300	7B: 128,769,236-128,769,916	Tandem
TraesCS7B02G117100	7B: 136,616,034-136,616,576	WGD/segmental
TraesCS7B02G118700	7B: 138,154,545-138,156,001	WGD/segmental
TraesCS7B02G127400	7B: 152,048,035-152,054,566	WGD/segmental
TraesCS7B02G134400	7B: 164,100,668-164,103,745	WGD/segmental
TraesCS7B02G144100	7B: 185,297,565-185,300,169	WGD/segmental
TraesCS7B02G150000	7B: 199,262,006-199,263,314	WGD/segmental
TraesCS7B02G154000	7B: 204,896,297-204,897,840	WGD/segmental
TraesCS7B02G154600	7B: 206,336,634-206,337,986	WGD/segmental
TraesCS7B02G163800	7B: 226,326,629-226,332,064	WGD/segmental
TraesCS7B02G171000	7B: 243,869,170-243,870,569	WGD/segmental
TraesCS7B02G176700	7B: 251,125,433-251,155,215	WGD/segmental
TraesCS7B02G188200	7B: 318,840,602-318,841,871	WGD/segmental
TraesCS7B02G189000	7B: 322,474,987-322,478,178	WGD/segmental
TraesCS7B02G191500	7B: 329,357,979-329,362,885	WGD/segmental
TraesCS7B02G226400	7B: 426,880,963-426,885,371	WGD/segmental
TraesCS7B02G234500	7B: 438,879,663-438,881,056	WGD/segmental
TraesCS7B02G234600	7B: 438,947,586-438,948,432	Tandem
TraesCS7B02G235200	7B: 439,554,461-439,555,402	WGD/segmental
TraesCS7B02G235300	7B: 439,603,445-439,603,975	WGD/segmental

Identifiers	Chromosomal location	Gene duplication type
TraesCS7B02G235400	7B: 439,672,118-439,673,316	WGD/segmental
TraesCS7B02G235500	7B: 439,856,105-439,856,653	Dispersed
TraesCS7B02G235600	7B: 439,860,270-439,860,871	WGD/segmental
TraesCS7B02G236000	7B: 440,010,718-440,011,430	WGD/segmental
TraesCS7B02G236100	7B: 440,187,197-440,200,717	WGD/segmental
TraesCS7B02G236200	7B: 440,242,318-440,242,899	WGD/segmental
TraesCS7B02G236600	7B: 440,813,814-440,814,532	WGD/segmental
TraesCS7B02G236700	7B: 440,817,367-440,818,036	WGD/segmental
TraesCS7B02G252000	7B: 464,562,219-464,568,187	WGD/segmental
TraesCS7B02G253800	7B: 468,051,077-468,051,730	WGD/segmental
TraesCS7B02G254300	7B: 468,827,311-468,827,967	Tandem
TraesCS7B02G255400	7B: 472,783,936-472,784,592	Tandem
TraesCS7B02G276000	7B: 505,368,476-505,369,769	WGD/segmental
TraesCS7B02G283100	7B: 517,312,612-517,320,661	WGD/segmental
TraesCS7B02G283200	7B: 517,323,264-517,328,832	Tandem
TraesCS7B02G293600	7B: 530,383,315-530,384,738	WGD/segmental
TraesCS7B02G327800	7B: 582,279,282-582,280,490	WGD/segmental
TraesCS7B02G328500	7B: 583,475,392-583,479,204	WGD/segmental
TraesCS7B02G340500	7B: 595,007,303-595,008,880	WGD/segmental
TraesCS7B02G340600	7B: 595,080,502-595,081,773	WGD/segmental
TraesCS7B02G340700	7B: 595,307,293-595,308,551	WGD/segmental
TraesCS7B02G343400	7B: 599,393,042-599,394,409	WGD/segmental
TraesCS7B02G343500	7B: 599,417,148-599,418,314	Tandem
TraesCS7B02G363900	7B: 626,238,481-626,242,957	WGD/segmental
TraesCS7B02G371200	7B: 637,337,842-637,339,156	WGD/segmental
TraesCS7B02G380600	7B: 645,391,794-645,392,851	WGD/segmental
TraesCS7B02G390000	7B: 656,507,357-656,510,577	WGD/segmental
TraesCS7B02G442100	7B: 706,856,614-706,863,111	WGD/segmental
TraesCS7B02G443600	7B: 708,416,110-708,418,579	WGD/segmental
TraesCS7B02G452400	7B: 713,662,115-713,664,747	WGD/segmental
TraesCS7B02G475300	7B: 731,243,312-731,243,890	WGD/segmental
TraesCS7B02G480500	7B: 734,360,430-734,361,207	WGD/segmental
TraesCS7B02G480600	7B: 734,366,063-734,367,084	WGD/segmental
TraesCS7B02G480700	7B: 734,379,438-734,380,061	WGD/segmental
TraesCS7B02G480900	7B: 734,407,735-734,408,358	WGD/segmental
TraesCS7B02G481000	7B: 734,426,411-734,427,250	WGD/segmental
TraesCS7B02G481100	7B: 734,430,194-734,431,162	Dispersed
TraesCS7D02G000400	7D: 365,927-369,790	WGD/segmental
TraesCS7D02G020500	7D: 9,032,535-9,033,056	WGD/segmental
TraesCS7D02G021000	7D: 9,398,457-9,399,163	Tandem

Identifiers	Chromosomal location	Gene duplication type
TraesCS7D02G022600	7D: 10,593,989-10,595,471	WGD/segmental
TraesCS7D02G023000	7D: 10,804,405-10,805,408	WGD/segmental
TraesCS7D02G023100	7D: 10,810,346-10,811,875	WGD/segmental
TraesCS7D02G043400	7D: 22,475,480-22,477,656	Proximal
TraesCS7D02G049800	7D: 25,399,672-25,401,983	WGD/segmental
TraesCS7D02G082700	7D: 49,639,348-49,640,076	WGD/segmental
TraesCS7D02G125200	7D: 78,314,938-78,315,796	Tandem
TraesCS7D02G129000	7D: 80,978,567-80,980,281	WGD/segmental
TraesCS7D02G141500	7D: 90,548,668-90,549,225	Dispersed
TraesCS7D02G146300	7D: 93,683,718-93,685,989	WGD/segmental
TraesCS7D02G154300	7D: 101,806,488-101,808,173	WGD/segmental
TraesCS7D02G157300	7D: 105,210,303-105,211,274	WGD/segmental
TraesCS7D02G170800	7D: 123,162,648-123,163,855	WGD/segmental
TraesCS7D02G178000	7D: 131,268,738-131,269,964	WGD/segmental
TraesCS7D02G191100	7D: 146,387,900-146,390,718	WGD/segmental
TraesCS7D02G191800	7D: 147,410,977-147,412,844	WGD/segmental
TraesCS7D02G212200	7D: 170,582,607-170,583,149	WGD/segmental
TraesCS7D02G213400	7D: 171,835,705-171,837,236	WGD/segmental
TraesCS7D02G214800	7D: 174,407,629-174,412,960	WGD/segmental
TraesCS7D02G222100	7D: 183,318,022-183,324,728	WGD/segmental
TraesCS7D02G226400	7D: 186,410,985-186,416,209	WGD/segmental
TraesCS7D02G246300	7D: 215,226,251-215,227,329	WGD/segmental
TraesCS7D02G252300	7D: 227,543,903-227,545,067	WGD/segmental
TraesCS7D02G257900	7D: 233,648,929-233,651,179	WGD/segmental
TraesCS7D02G258600	7D: 234,347,652-234,349,027	WGD/segmental
TraesCS7D02G266300	7D: 249,368,711-249,373,796	WGD/segmental
TraesCS7D02G271800	7D: 261,865,093-261,868,004	WGD/segmental
TraesCS7D02G273100	7D: 263,831,865-263,833,593	WGD/segmental
TraesCS7D02G282300	7D: 285,674,867-285,692,862	WGD/segmental
TraesCS7D02G293200	7D: 363,038,783-363,041,009	WGD/segmental
TraesCS7D02G295800	7D: 371,268,062-371,268,580	WGD/segmental
TraesCS7D02G305600	7D: 386,906,817-386,908,952	WGD/segmental
TraesCS7D02G322500	7D: 412,672,331-412,676,428	WGD/segmental
TraesCS7D02G330600	7D: 422,155,647-422,156,339	WGD/segmental
TraesCS7D02G330800	7D: 422,210,179-422,211,562	Tandem
TraesCS7D02G330900	7D: 422,345,011-422,345,965	WGD/segmental
TraesCS7D02G331000	7D: 422,571,221-422,571,760	WGD/segmental
TraesCS7D02G331100	7D: 422,617,711-422,618,253	Tandem
TraesCS7D02G331200	7D: 422,673,072-422,674,509	WGD/segmental
TraesCS7D02G331300	7D: 422,718,401-422,718,916	WGD/segmental

Identifiers	Chromosomal location	Gene duplication type
TraesCS7D02G331800	7D: 422,952,386-422,953,079	WGD/segmental
TraesCS7D02G331900	7D: 423,326,226-423,326,786	WGD/segmental
TraesCS7D02G332000	7D: 423,551,439-423,551,906	WGD/segmental
TraesCS7D02G332200	7D: 423,642,005-423,642,472	WGD/segmental
TraesCS7D02G332500	7D: 423,709,245-423,709,832	Tandem
TraesCS7D02G332600	7D: 423,710,927-423,711,490	Tandem
TraesCS7D02G332700	7D: 423,712,801-423,713,247	WGD/segmental
TraesCS7D02G349700	7D: 449,548,060-449,548,713	WGD/segmental
TraesCS7D02G350200	7D: 451,040,506-451,041,162	WGD/segmental
TraesCS7D02G370800	7D: 479,597,566-479,598,806	WGD/segmental
TraesCS7D02G377500	7D: 488,784,279-488,790,738	WGD/segmental
TraesCS7D02G377600	7D: 488,792,548-488,796,973	Tandem
TraesCS7D02G387300	7D: 501,099,772-501,101,691	WGD/segmental
TraesCS7D02G413200	7D: 531,585,466-531,590,378	WGD/segmental
TraesCS7D02G415800	7D: 534,654,112-534,657,931	Dispersed
TraesCS7D02G420000	7D: 539,486,767-539,488,543	WGD/segmental
TraesCS7D02G420400	7D: 540,234,102-540,237,912	WGD/segmental
TraesCS7D02G431100	7D: 550,462,847-550,464,393	WGD/segmental
TraesCS7D02G431200	7D: 550,552,950-550,554,260	WGD/segmental
TraesCS7D02G431300	7D: 550,800,421-550,801,746	WGD/segmental
TraesCS7D02G433200	7D: 552,987,985-552,989,139	WGD/segmental
TraesCS7D02G433400	7D: 553,369,856-553,371,214	Tandem
TraesCS7D02G451000	7D: 570,662,870-570,668,229	WGD/segmental
TraesCS7D02G457000	7D: 575,535,921-575,537,188	WGD/segmental
TraesCS7D02G465600	7D: 579,756,244-579,757,036	WGD/segmental
TraesCS7D02G473200	7D: 586,484,621-586,488,990	WGD/segmental
TraesCS7D02G513500	7D: 614,538,679-614,545,375	WGD/segmental
TraesCS7D02G514900	7D: 615,597,931-615,600,441	WGD/segmental
TraesCS7D02G522000	7D: 619,040,369-619,043,668	WGD/segmental
TraesCS7D02G537300	7D: 628,986,543-628,987,668	WGD/segmental
TraesCS7D02G538000	7D: 629,247,782-629,248,694	Tandem
TraesCS7D02G547300	7D: 633,512,851-633,513,363	Dispersed
TraesCS7D02G554000	7D: 638,267,742-638,268,573	WGD/segmental
TraesCS7D02G554100	7D: 638,273,672-638,274,512	WGD/segmental
TraesCS7D02G554200	7D: 638,287,890-638,288,510	Tandem
TraesCS7D02G554300	7D: 638,296,335-638,296,958	Tandem
TraesCS7D02G554400	7D: 638,330,471-638,331,088	WGD/segmental
TraesCS7D02G554500	7D: 638,343,258-638,344,257	Tandem
TraesCS7D02G554600	7D: 638,354,010-638,354,609	Tandem
TraesCS7D02G554700	7D: 638,381,344-638,382,296	Proximal

Supplemental Table 5. The differential gene expression analysis of 698 RING protein genes from in-house transcriptome data of 28 DAA seed stage among three genotypes (TAC 75, TAC 6 and C 306) differing in amylose content along with the p-value for all three biological replicates.

	'TAC 75' vs. 'C 306'		'TAC 6' vs. 'C 306'	
**Identifiers**	**Log_2 fold change**	***p*-value**	**Log_2 fold change**	***p*-value**
TraesCS6A02G262500	-1.04	0.43	-0.61	0.67
TraesCS2D02G071800	-0.11	0.90	-1.20	0.26
TraesCS7B02G029900	0.50	0.65	0.11	0.93
TraesCS6B02G254400	-0.06	0.94	-0.72	0.39
TraesCS3B02G484400	-	-	-	-
TraesCS6D02G218600	-0.68	0.38	-0.17	0.83
TraesCS1B02G219500	-0.60	0.58	-0.93	0.41
TraesCS3D02G332600	0.24	0.76	-0.16	0.86
TraesCS1B02G228600	0.27	0.80	0.11	0.93
TraesCS7B02G235400	-0.46	0.70	-1.63	0.23
TraesCS6B02G346000	2.06	0.08	-1.60	0.23
TraesCS6D02G107900	0.16	0.86	-0.21	0.82
TraesCS3B02G370700	0.22	0.78	0.47	0.58
TraesCS3D02G147800	0.82	0.41	-1.17	0.26
TraesCS3D02G459900	0.73	0.58	-1.27	0.45
TraesCS7B02G276000	-1.31	0.28	-0.91	0.45
TraesCS3B02G462300	-	-	-	-
TraesCS2A02G437200	0.64	0.54	0.86	0.43
TraesCS2B02G467200	-1.21	0.21	-0.38	0.71
TraesCS7A02G300000	0.77	0.47	-0.23	0.85
TraesCS3D02G024200	1.15	0.49	-1.86	0.48
TraesCS4D02G313300	1.83	0.10	-0.45	0.69
TraesCS3A02G426100	-2.22	0.37	1.55	0.34
TraesCS4D02G192800	-0.18	0.88	-0.88	0.52
TraesCS3A02G339000	0.04	0.96	-0.49	0.61
TraesCS6A02G236100	-0.51	0.43	-0.43	0.54
TraesCS6B02G146100	-0.45	0.64	0.41	0.69
TraesCS2B02G019500	-2.22	0.37	1.32	0.43
TraesCS3B02G509100	2.19	0.13	1.18	0.39
TraesCS6A02G315600	2.23	0.12	-0.81	0.59
TraesCS6A02G050800	0.34	0.80	-0.31	0.83
TraesCS3B02G313100	-0.58	0.74	0.01	1.00
TraesCS3D02G293300	0.50	0.61	-1.04	0.33
TraesCS2D02G392600	0.69	0.43	0.84	0.30
TraesCS3D02G019900	0.50	0.62	0.17	0.87

	'TAC 75' vs. 'C 306'		'TAC 6' vs. 'C 306'	
**Identifiers**	**Log$_2$ fold change**	***p*-value**	**Log$_2$ fold change**	***p*-value**
TraesCS3B02G567600	-0.70	0.59	-1.29	0.41
TraesCS6B02G067200	-0.02	0.98	-0.82	0.47
TraesCS1B02G228800	0.89	0.44	-0.12	0.92
TraesCS7A02G024600	1.62	0.59	1.89	0.48
TraesCS7B02G188200	0.17	0.84	0.26	0.78
TraesCS7A02G346400	1.62	0.59	-	-
TraesCS1D02G217800	1.27	0.22	0.19	0.86
TraesCS7A02G273000	1.98	0.08	0.32	0.79
TraesCS6A02G096200	0.01	0.99	0.55	0.56
TraesCS6A02G117900	0.75	0.39	0.24	0.80
TraesCS5B02G197200	-0.95	0.39	-0.67	0.59
TraesCS2D02G479100	-1.00	0.21	-0.10	0.90
TraesCS2D02G392500	1.35	0.23	0.11	0.93
TraesCSU02G109900	0.24	0.83	-0.10	0.93
TraesCS3B02G165100	1.49	0.18	-2.04	0.24
TraesCS7A02G190800	0.14	0.84	-0.86	0.24
TraesCS4A02G112500	-1.68	0.09	-0.43	0.70
TraesCS2B02G413800	0.20	0.76	-0.30	0.66
TraesCS1B02G343100	1.19	0.20	0.55	0.61
TraesCS3A02G464800	0.91	0.32	-0.17	0.89
TraesCS6B02G264500	-0.41	0.61	-0.47	0.55
TraesCS2A02G446100	-0.29	0.75	-1.62	0.19
TraesCS2A02G448700	0.32	0.75	0.26	0.83
TraesCS1A02G215300	0.56	0.61	-1.27	0.31
TraesCS5B02G144700	-0.19	0.76	0.13	0.85
TraesCS2B02G576800	0.45	0.57	0.01	0.99
TraesCS3D02G421100	0.37	0.74	0.30	0.80
TraesCS3A02G293500	-0.11	0.93	-4.48	0.08
TraesCS5A02G494500	0.15	0.87	-1.50	0.19
TraesCS3A02G095400	-1.56	0.04	-0.56	0.48
TraesCS6B02G124600	-0.27	0.73	0.47	0.55
TraesCS5B02G069700	0.55	0.69	-1.86	0.28
TraesCS7D02G258600	1.70	0.17	1.47	0.20
TraesCS3A02G189200	0.59	0.63	0.95	0.52
TraesCS3B02G328200	0.95	0.32	-1.09	0.34
TraesCS5D02G391100	0.48	0.71	1.64	0.21
TraesCS6D02G207700	-0.45	0.55	-0.85	0.29
TraesCS6D02G237700	0.31	0.75	0.94	0.40

	'TAC 75' vs. 'C 306'		'TAC 6' vs. 'C 306'	
**Identifiers**	**Log_2 fold change**	***p*-value**	**Log_2 fold change**	***p*-value**
TraesCS4B02G191600	0.20	0.86	-1.66	0.31
TraesCS4D02G129700	1.07	0.33	1.25	0.28
TraesCS7D02G178000	1.18	0.35	-0.62	0.65
TraesCS6B02G318600	0.72	0.59	0.01	1.00
TraesCS6A02G352200	0.53	0.60	-0.20	0.85
TraesCS7A02G005100	1.08	0.21	0.87	0.34
TraesCS6A02G308200	0.33	0.61	0.50	0.51
TraesCS7A02G156900	0.78	0.50	-1.72	0.20
TraesCS5A02G198500	-1.12	0.43	-0.24	0.86
TraesCS7B02G154600	0.29	0.82	-1.58	0.25
TraesCS6D02G287400	0.05	0.94	0.43	0.60
TraesCS6D02G087300	0.36	0.68	0.41	0.66
TraesCS6D02G242100	1.28	0.38	1.19	0.38
TraesCS6B02G149700	0.73	0.32	0.41	0.60
TraesCS6A02G202400	-1.54	0.14	-1.47	0.22
TraesCS7A02G308900	0.99	0.31	0.14	0.89
TraesCS3B02G218400	-0.07	0.96	1.03	0.43
TraesCS2A02G545900	0.42	0.58	-0.11	0.90
TraesCS5A02G381800	0.06	0.95	-0.15	0.88
TraesCS3A02G260500	-0.58	0.53	0.50	0.63
TraesCS6B02G289700	-0.45	0.75	-1.86	0.28
TraesCS2A02G556200	0.19	0.72	0.38	0.57
TraesCS6D02G111500	0.60	0.41	0.47	0.55
TraesCS2B02G586500	0.13	0.82	0.33	0.66
TraesCS7D02G305600	0.83	0.41	-0.48	0.67
TraesCS1D02G263900	-0.25	0.72	-0.26	0.73
TraesCS6B02G291300	0.11	0.89	-0.31	0.73
TraesCS7A02G176700	0.23	0.86	-1.71	0.25
TraesCS1A02G346900	3.30	0.26	-	-
TraesCS1D02G296300	-1.25	0.11	-0.97	0.29
TraesCS6A02G304300	1.26	0.27	0.92	0.44
TraesCS3D02G511300	0.52	0.59	1.07	0.30
TraesCS6D02G250600	0.82	0.39	0.00	1.00
TraesCS3D02G421000	0.71	0.37	-1.06	0.30
TraesCS2A02G399700	0.26	0.82	0.40	0.73
TraesCS4D02G192500	0.70	0.45	-0.13	0.90
TraesCS1D02G243200	-0.58	0.48	-0.21	0.81
TraesCS3D02G192700	0.01	1.00	-1.13	0.35

	'TAC 75' vs. 'C 306'		'TAC 6' vs. 'C 306'	
**Identifiers**	**Log$_2$ fold change**	***p*-value**	**Log$_2$ fold change**	***p*-value**
TraesCS1B02G395000	0.12	0.84	0.45	0.46
TraesCS6B02G288700	0.58	0.61	-0.67	0.60
TraesCS6B02G332400	1.95	0.20	2.77	0.07
TraesCS1B02G465700	0.41	0.59	0.29	0.72
TraesCS3D02G184200	0.50	0.58	0.11	0.91
TraesCS1D02G343700	-1.17	0.19	-0.61	0.50
TraesCS6D02G243200	0.36	0.80	-1.86	0.28
TraesCS3B02G139600	0.58	0.47	0.27	0.76
TraesCS1A02G374800	-0.01	0.99	0.18	0.80
TraesCS6A02G227300	0.75	0.36	0.53	0.50
TraesCS6B02G336900	-0.15	0.81	0.26	0.72
TraesCS7A02G253700	0.07	0.95	-1.12	0.34
TraesCS6B02G286500	2.93	0.04	-0.78	0.61
TraesCS1B02G254700	0.78	0.34	-0.04	0.97
TraesCS7D02G370800	0.92	0.52	-3.43	0.19
TraesCS6A02G375500	0.00	1.00	-0.83	0.26
TraesCS6D02G334900	0.51	0.65	-0.49	0.67
TraesCS3A02G327900	-0.10	0.88	-0.76	0.28
TraesCS1B02G354100	-0.29	0.76	-1.01	0.35
TraesCS1A02G341400	0.57	0.56	-0.75	0.52
TraesCS6D02G360000	0.82	0.26	-0.49	0.55
TraesCS1A02G243200	0.38	0.63	0.37	0.67
TraesCS4A02G112900	0.77	0.40	0.53	0.60
TraesCS7D02G273100	0.61	0.55	-1.17	0.23
TraesCS6B02G266600	1.20	0.35	-1.60	0.26
TraesCS3D02G122200	0.29	0.73	-0.34	0.71
TraesCS2B02G469500	-0.21	0.78	-0.07	0.93
TraesCS4B02G191300	0.75	0.39	-0.37	0.73
TraesCS7B02G061100	-0.42	0.72	-2.47	0.07
TraesCS3A02G120100	0.58	0.57	0.50	0.65
TraesCS3D02G321400	0.60	0.39	0.32	0.67
TraesCS1A02G295900	-0.57	0.51	-0.98	0.30
TraesCS1A02G280300	0.46	0.58	-0.36	0.74
TraesCS1B02G361000	0.48	0.71	-1.10	0.45
TraesCS5A02G512800	0.35	0.64	-0.49	0.55
TraesCS2D02G447900	0.27	0.75	-0.77	0.44
TraesCS1D02G279400	0.06	0.93	-0.08	0.91
TraesCS7D02G049800	0.81	0.35	0.94	0.32

	'TAC 75' vs. 'C 306'		'TAC 6' vs. 'C 306'	
**Identifiers**	**Log$_2$ fold change**	***p*-value**	**Log$_2$ fold change**	***p*-value**
TraesCS7B02G150000	1.43	0.28	-2.17	0.17
TraesCS4D02G339200	0.47	0.55	0.26	0.76
TraesCS2B02G051700	-0.26	0.73	-0.47	0.57
TraesCS6D02G294800	2.12	0.11	-1.43	0.34
TraesCS1A02G205100	0.14	0.87	0.08	0.94
TraesCS7A02G442300	0.33	0.67	-0.45	0.56
TraesCS4B02G162300	-0.04	0.96	-0.52	0.56
TraesCS7D02G252300	1.25	0.36	-4.25	0.11
TraesCS4B02G344100	0.23	0.74	-0.11	0.88
TraesCS1A02G225700	-0.95	0.20	-0.62	0.43
TraesCS3B02G357400	0.51	0.43	0.42	0.54
TraesCS2B02G477500	-0.36	0.64	-0.29	0.71
TraesCS4D02G095800	1.95	0.18	-3.11	0.23
TraesCS4B02G164000	0.53	0.40	0.24	0.72
TraesCS1D02G226900	-0.23	0.71	-0.53	0.44
TraesCS7B02G171000	1.05	0.25	-1.51	0.08
TraesCS1D02G208400	0.79	0.32	-0.02	0.98
TraesCS1B02G036900	1.18	0.23	-1.34	0.15
TraesCS6A02G259200	1.72	0.07	-0.59	0.54
TraesCS7D02G377600	-0.32	0.56	0.29	0.65
TraesCS1B02G238900	0.20	0.75	-0.07	0.92
TraesCS1D02G030300	0.86	0.42	-0.78	0.42
TraesCS1A02G029800	0.88	0.36	-0.97	0.29
TraesCS7B02G134400	-0.45	0.39	-0.19	0.79
TraesCS3B02G462600	-0.38	0.49	0.20	0.77
TraesCS3B02G462000	-0.41	0.64	0.19	0.84
TraesCS3D02G421200	-0.35	0.52	-0.04	0.95
TraesCS3A02G425900	0.67	0.21	0.39	0.54
TraesCS3A02G426400	-0.20	0.74	0.02	0.98
TraesCS1A02G398300	0.12	0.85	0.06	0.94
TraesCS7D02G157300	0.68	0.58	-3.04	0.04
TraesCS5A02G207100	0.35	0.67	-0.08	0.93
TraesCS5A02G145800	-0.09	0.88	0.18	0.78
TraesCS5D02G036200	-	-	-	-
TraesCS6D02G283700	0.68	0.50	1.42	0.18
TraesCS2A02G156800	1.34	0.04	-0.18	0.79
TraesCS5B02G164100	0.81	0.29	-0.54	0.51
TraesCS7B02G095700	0.79	0.21	-0.24	0.74

	'TAC 75' vs. 'C 306'		'TAC 6' vs. 'C 306'	
**Identifiers**	**Log_2 fold change**	***p*-value**	**Log_2 fold change**	***p*-value**
TraesCS2D02G516000	0.55	0.34	0.24	0.73
TraesCS2D02G233500	-0.10	0.90	-0.34	0.65
TraesCS6D02G221300	0.17	0.91	0.14	0.93
TraesCS1A02G329600	0.00	1.00	-0.19	0.86
TraesCS6D02G240300	0.74	0.52	-2.47	0.05
TraesCS7D02G191800	0.26	0.72	-0.09	0.91
TraesCS1B02G242800	-0.58	0.74	0.01	1.00
TraesCS2D02G162600	2.09	0.00	1.04	0.23
TraesCS1A02G177600	-0.20	0.74	0.07	0.91
TraesCS1B02G218700	1.05	0.16	0.43	0.64
TraesCS5D02G036500	-0.58	0.74	-1.86	0.48
TraesCS3D02G260700	-0.05	0.95	-0.14	0.88
TraesCS5A02G326900	2.37	0.41	-	-
TraesCS6B02G032400	0.83	0.44	-0.89	0.45
TraesCS4A02G441400	0.55	0.56	1.09	0.32
TraesCS5A02G248300	0.08	0.90	-0.32	0.63
TraesCS4B02G134900	0.19	0.89	0.51	0.72
TraesCS5B02G054600	-0.39	0.55	-1.09	0.14
TraesCS4D02G152900	0.09	0.87	0.41	0.52
TraesCS2B02G306600	0.01	0.99	-0.98	0.21
TraesCS6B02G413100	0.14	0.83	-0.41	0.55
TraesCS2D02G037600	0.36	0.62	-0.03	0.97
TraesCS7B02G189000	0.29	0.72	0.10	0.91
TraesCS4A02G129800	-0.04	0.97	0.00	1.00
TraesCS3A02G426200	0.22	0.78	0.43	0.63
TraesCS5D02G171400	0.60	0.49	-0.88	0.35
TraesCS2D02G557400	0.20	0.71	0.35	0.62
TraesCS1D02G142100	-0.16	0.82	0.37	0.63
TraesCS7B02G340500	0.36	0.73	-0.91	0.46
TraesCS5A02G547400	-0.28	0.73	-0.81	0.37
TraesCS3D02G338100	-0.82	0.52	-2.43	0.17
TraesCS5B02G400800	-0.26	0.78	-1.72	0.15
TraesCS2B02G182300	1.52	0.01	0.36	0.60
TraesCS6B02G164200	-0.16	0.84	0.55	0.48
TraesCS5D02G235800	0.65	0.60	-4.07	0.11
TraesCS5A02G167100	1.86	0.07	-1.76	0.13
TraesCS2B02G115500	-0.95	0.39	-1.63	0.22
TraesCS2D02G397200	-0.19	0.85	-0.39	0.71

	'TAC 75' vs. 'C 306'		'TAC 6' vs. 'C 306'	
**Identifiers**	**Log_2 fold change**	***p*-value**	**Log_2 fold change**	***p*-value**
TraesCS4B02G093600	-0.31	0.62	0.09	0.90
TraesCS7A02G212900	-0.25	0.68	-0.24	0.71
TraesCS7A02G129900	1.92	0.23	0.01	1.00
TraesCS6B02G268900	-0.60	0.48	-0.29	0.76
TraesCS2D02G288000	0.43	0.55	-1.12	0.19
TraesCS7D02G214800	0.03	0.97	0.35	0.58
TraesCS7A02G347800	0.17	0.84	-1.88	0.07
TraesCS4B02G322200	1.15	0.27	-0.16	0.88
TraesCS6B02G385100	2.25	0.03	0.84	0.41
TraesCS1D02G382000	0.27	0.66	0.27	0.68
TraesCS7D02G293200	0.02	0.98	-0.30	0.72
TraesCS1D02G405800	0.09	0.89	-0.09	0.91
TraesCS4D02G155700	0.98	0.21	1.10	0.19
TraesCS4A02G220900	0.72	0.50	-0.92	0.49
TraesCS5D02G060200	-0.04	0.96	-1.35	0.08
TraesCS7D02G431100	0.77	0.35	0.42	0.63
TraesCS3A02G439700	-0.21	0.75	0.51	0.44
TraesCS2A02G145200	-1.28	0.30	-1.57	0.28
TraesCS5D02G405300	-0.20	0.82	-1.08	0.31
TraesCS3B02G376500	0.51	0.70	-1.29	0.42
TraesCS7B02G283200	-0.53	0.34	0.34	0.60
TraesCS1D02G176100	0.32	0.60	0.39	0.53
TraesCS5D02G146400	0.11	0.85	0.17	0.79
TraesCS5D02G333100	0.56	0.49	-0.86	0.30
TraesCS3A02G344600	-0.37	0.67	-1.08	0.28
TraesCS1B02G398900	0.41	0.68	0.97	0.31
TraesCS1D02G453000	-3.05	0.19	-2.65	0.29
TraesCS1B02G202600	0.07	0.90	0.22	0.72
TraesCS2A02G455400	-0.23	0.72	-0.22	0.73
TraesCS3D02G420500	0.07	0.89	-0.30	0.64
TraesCS2B02G336400	-0.14	0.89	-2.95	0.03
TraesCS2A02G318300	0.79	0.48	-2.31	0.12
TraesCS5B02G065600	0.12	0.89	0.07	0.94
TraesCS2A02G372600	0.09	0.88	-0.21	0.75
TraesCS2B02G336300	0.78	0.55	-1.08	0.48
TraesCS2B02G417700	-0.62	0.50	-0.11	0.92
TraesCS1B02G007900	0.14	0.83	-0.08	0.92
TraesCS1A02G133300	0.37	0.74	0.55	0.65

	'TAC 75' vs. 'C 306'		'TAC 6' vs. 'C 306'	
**Identifiers**	**Log_2 fold change**	***p*-value**	**Log_2 fold change**	***p*-value**
TraesCS3B02G473700	0.17	0.77	0.53	0.39
TraesCS4A02G162000	-0.07	0.92	0.42	0.57
TraesCS2A02G514400	0.48	0.39	0.15	0.83
TraesCS2D02G315800	0.17	0.88	-0.79	0.54
TraesCS5A02G058000	0.61	0.40	0.67	0.35
TraesCS3B02G111000	-0.39	0.59	0.06	0.93
TraesCS2A02G229600	-0.49	0.55	-0.91	0.32
TraesCS3B02G293600	-0.25	0.78	-0.38	0.71
TraesCS1A02G002000	0.19	0.79	0.06	0.94
TraesCS5A02G129900	0.18	0.81	-0.49	0.53
TraesCS5D02G188300	-0.06	0.93	0.29	0.67
TraesCS6A02G145300	-0.29	0.71	0.46	0.56
TraesCS1D02G230300	-0.57	0.53	-0.78	0.44
TraesCS5A02G326800	0.44	0.51	-0.61	0.42
TraesCS5A02G183200	-0.20	0.76	0.29	0.66
TraesCS3D02G105300	-0.25	0.68	-0.23	0.72
TraesCS3A02G503400	-0.27	0.64	0.06	0.92
TraesCS3D02G432500	0.36	0.60	0.34	0.65
TraesCSU02G113400	0.53	0.44	0.66	0.35
TraesCS2A02G099300	-1.54	0.13	-3.52	0.03
TraesCS2B02G274500	0.14	0.84	0.23	0.75
TraesCS2A02G227400	-0.04	0.95	0.24	0.71
TraesCS7B02G340600	1.23	0.19	0.14	0.89
TraesCS5B02G469800	0.26	0.73	-0.38	0.65
TraesCS4D02G176700	0.15	0.87	-0.11	0.91
TraesCS5B02G129800	-0.06	0.94	-0.27	0.77
TraesCS5B02G382100	0.24	0.64	0.26	0.68
TraesCS2A02G395100	1.39	0.12	-0.35	0.75
TraesCS1A02G373200	-1.03	0.28	-0.44	0.67
TraesCS2A02G039300	0.02	0.98	-1.66	0.31
TraesCS4D02G187800	0.47	0.49	-0.59	0.36
TraesCS7D02G146300	-0.39	0.59	-0.42	0.57
TraesCS3B02G591100	0.02	0.99	-1.27	0.45
TraesCS5D02G388500	0.40	0.44	0.11	0.86
TraesCS5A02G378600	0.48	0.38	0.48	0.44
TraesCS4A02G183700	-0.49	0.67	-0.29	0.81
TraesCS2A02G224300	-0.21	0.76	-0.06	0.94
TraesCS7D02G129000	-0.25	0.79	-1.02	0.36

	'TAC 75' vs. 'C 306'		'TAC 6' vs. 'C 306'	
**Identifiers**	**Log$_2$ fold change**	***p*-value**	**Log$_2$ fold change**	***p*-value**
TraesCS6B02G310500	-0.07	0.91	-0.09	0.90
TraesCS1B02G274400	0.13	0.84	0.07	0.92
TraesCS2D02G315900	0.80	0.42	-2.56	0.04
TraesCS4D02G003000	-0.01	0.98	0.16	0.81
TraesCS7A02G428300	-0.09	0.87	-0.11	0.87
TraesCS2D02G455800	-0.24	0.70	-0.52	0.44
TraesCS2B02G250800	0.54	0.39	0.79	0.21
TraesCS2D02G235300	-0.05	0.94	0.47	0.47
TraesCS4A02G161100	0.47	0.47	0.83	0.23
TraesCS5A02G049400	-0.47	0.47	-1.40	0.07
TraesCS4D02G064500	-0.68	0.44	-0.18	0.86
TraesCS5D02G254900	0.59	0.67	-3.11	0.23
TraesCS3B02G120600	0.28	0.66	-0.33	0.61
TraesCS2B02G255300	0.07	0.91	0.29	0.65
TraesCS3D02G095600	-0.29	0.69	-0.27	0.71
TraesCS1B02G289200	-0.31	0.69	-0.27	0.74
TraesCS7D02G387300	0.17	0.82	-0.57	0.49
TraesCS5B02G181400	-0.20	0.76	0.31	0.65
TraesCS5D02G069800	-0.18	0.80	0.04	0.96
TraesCS3B02G305200	-0.23	0.74	-0.62	0.42
TraesCS1D02G217700	-0.65	0.55	-1.17	0.30
TraesCS7A02G296500	-0.10	0.90	-0.53	0.55
TraesCS3B02G595200	1.11	0.19	-0.09	0.92
TraesCS1A02G201700	0.47	0.49	0.01	0.98
TraesCS6D02G345700	0.61	0.27	0.48	0.43
TraesCS6A02G121400	0.33	0.56	0.42	0.50
TraesCS3A02G103000	0.24	0.69	-0.07	0.91
TraesCS5D02G223800	0.57	0.30	0.15	0.81
TraesCS6B02G118100	0.23	0.72	-0.29	0.69
TraesCS5A02G547800	0.70	0.38	-0.10	0.92
TraesCS4A02G156600	0.48	0.39	0.16	0.80
TraesCS4B02G003100	-0.14	0.84	0.42	0.54
TraesCS3A02G414400	-0.02	0.97	0.26	0.67
TraesCS4A02G249200	-0.64	0.36	-0.18	0.80
TraesCS6D02G134300	0.16	0.80	0.22	0.75
TraesCS1D02G026100	0.41	0.50	-0.19	0.76
TraesCS5D02G137400	0.21	0.79	-0.15	0.86
TraesCS3A02G271500	0.25	0.74	0.11	0.89

	'TAC 75' vs. 'C 306'		'TAC 6' vs. 'C 306'	
**Identifiers**	**Log$_2$ fold change**	***p*-value**	**Log$_2$ fold change**	***p*-value**
TraesCS6A02G326100	0.04	0.96	0.35	0.62
TraesCS3D02G532300	0.45	0.61	-0.86	0.36
TraesCS2D02G230600	-0.03	0.96	-0.13	0.85
TraesCS3D02G509900	0.47	0.38	0.39	0.55
TraesCS5A02G010000	0.36	0.55	0.33	0.62
TraesCS1B02G242700	-0.58	0.52	-0.86	0.40
TraesCS7A02G391700	0.76	0.32	-0.28	0.75
TraesCS5B02G385300	-0.39	0.67	0.37	0.70
TraesCS2A02G290100	0.28	0.67	-0.86	0.24
TraesCS7D02G420000	1.91	0.13	0.32	0.82
TraesCS3D02G384200	-0.17	0.80	-0.82	0.30
TraesCS2D02G098400	0.99	0.33	-0.49	0.63
TraesCS3B02G208200	0.32	0.57	0.50	0.40
TraesCS4B02G151200	-0.24	0.70	0.16	0.80
TraesCS1A02G296100	-0.16	0.81	-0.56	0.43
TraesCS7D02G271800	-0.63	0.37	-0.46	0.55
TraesCS3D02G183800	0.42	0.46	0.31	0.62
TraesCS4A02G117900	0.83	0.17	-0.09	0.89
TraesCS4D02G148400	-0.09	0.88	0.23	0.72
TraesCS6A02G405400	0.25	0.69	0.16	0.81
TraesCS2A02G318200	-0.15	0.89	-0.40	0.76
TraesCSU02G093700	0.31	0.74	-0.69	0.54
TraesCS1B02G118800	0.22	0.78	0.70	0.40
TraesCS3A02G399700	-1.01	0.25	-2.05	0.04
TraesCS1D02G296100	-0.02	0.97	-0.30	0.65
TraesCS5B02G230500	0.24	0.82	-1.81	0.18
TraesCS7B02G293600	-0.15	0.84	-0.34	0.66
TraesCS4A02G053400	0.22	0.77	0.23	0.77
TraesCS5B02G090000	-0.34	0.66	0.22	0.76
TraesCS3D02G405200	0.25	0.73	-0.26	0.74
TraesCS5B02G313800	0.04	0.95	-0.21	0.76
TraesCS1A02G141900	1.06	0.11	0.03	0.97
TraesCS3A02G440000	0.15	0.82	-0.04	0.95
TraesCS7A02G144900	-0.80	0.46	-1.00	0.38
TraesCS3B02G397000	0.69	0.47	0.48	0.66
TraesCS2D02G174800	-0.28	0.83	-0.24	0.85
TraesCS5A02G248100	0.13	0.91	-4.86	0.06
TraesCS7B02G327800	0.93	0.49	0.00	1.00

	'TAC 75' vs. 'C 306'		'TAC 6' vs. 'C 306'	
**Identifiers**	**Log$_2$ fold change**	***p*-value**	**Log$_2$ fold change**	***p*-value**
TraesCS6D02G262500	0.44	0.54	0.67	0.37
TraesCS7B02G154000	0.36	0.75	-0.13	0.92
TraesCS7A02G486700	0.37	0.53	0.22	0.72
TraesCS7D02G322500	-0.12	0.83	-0.14	0.82
TraesCS2B02G251300	-0.54	0.54	-0.18	0.84
TraesCS3D02G271100	-0.17	0.81	-0.31	0.66
TraesCS3D02G358300	0.92	0.23	-0.28	0.73
TraesCS3D02G408900	0.35	0.53	0.13	0.83
TraesCS6A02G362000	0.14	0.81	0.48	0.44
TraesCS2A02G232200	-0.38	0.51	0.24	0.69
TraesCS6D02G140100	0.12	0.88	-1.51	0.11
TraesCS7A02G441900	0.37	0.66	0.00	1.00
TraesCS2B02G402300	0.16	0.79	0.70	0.26
TraesCS7A02G247700	0.35	0.57	0.35	0.60
TraesCS3B02G462500	-0.14	0.82	0.05	0.94
TraesCS1D02G201500	-0.63	0.41	0.08	0.92
TraesCS4B02G186500	1.00	0.15	-0.23	0.73
TraesCS2D02G232900	0.10	0.86	0.19	0.76
TraesCS6D02G305800	-0.45	0.48	-0.31	0.66
TraesCS5B02G245800	0.55	0.64	-1.09	0.40
TraesCS1D02G141000	0.94	0.10	-0.01	0.99
TraesCS2B02G542200	0.49	0.35	0.23	0.71
TraesCS7D02G349700	-0.71	0.58	-0.39	0.76
TraesCS4A02G311200	0.06	0.92	0.37	0.57
TraesCS6D02G389100	0.25	0.70	0.05	0.94
TraesCS6B02G356400	0.29	0.69	-0.15	0.84
TraesCS1A02G162400	0.21	0.79	-0.53	0.54
TraesCS6A02G084400	0.27	0.66	0.02	0.97
TraesCS3A02G176600	0.09	0.87	0.19	0.76
TraesCS1A02G228500	-0.70	0.40	-1.35	0.18
TraesCS3B02G474100	0.28	0.65	0.35	0.59
TraesCS4A02G095800	2.19	0.02	-0.76	0.44
TraesCS5A02G232200	1.05	0.37	-0.37	0.79
TraesCS6B02G173500	0.22	0.76	0.79	0.30
TraesCS3D02G432800	0.12	0.85	0.12	0.85
TraesCS2B02G418300	-0.62	0.47	-0.37	0.69
TraesCS3B02G443900	0.10	0.89	0.00	1.00
TraesCS6B02G274100	-0.05	0.92	-0.05	0.94

	'TAC 75' vs. 'C 306'		'TAC 6' vs. 'C 306'	
**Identifiers**	**Log_2 fold change**	***p*-value**	**Log_2 fold change**	***p*-value**
TraesCS2A02G564000	-0.16	0.85	-0.09	0.92
TraesCS7D02G451000	0.17	0.77	0.34	0.58
TraesCS1D02G002400	0.33	0.56	-0.16	0.83
TraesCS3A02G467300	-0.10	0.86	0.08	0.89
TraesCS3B02G566800	-0.04	0.94	0.35	0.56
TraesCS1A02G143300	0.03	0.96	0.13	0.85
TraesCS6B02G379400	0.06	0.92	-0.32	0.63
TraesCS7B02G363900	-0.24	0.69	-0.10	0.87
TraesCS3A02G410600	-0.66	0.38	-0.31	0.70
TraesCS4A02G118100	2.37	0.41	2.68	0.29
TraesCS3D02G462600	0.21	0.68	0.37	0.55
TraesCS1D02G315600	0.18	0.79	-0.23	0.75
TraesCS5D02G139400	0.04	0.95	0.27	0.67
TraesCS7A02G381300	-0.23	0.65	0.27	0.66
TraesCS1B02G032600	0.57	0.44	0.23	0.78
TraesCS4D02G090400	-0.14	0.82	0.32	0.64
TraesCS1B02G179000	0.23	0.76	-0.50	0.55
TraesCS2D02G574200	-0.18	0.80	-0.82	0.25
TraesCS4B02G251200	0.42	0.61	0.32	0.72
TraesCS4B02G208600	-0.04	0.95	-0.27	0.71
TraesCS3B02G499500	-0.58	0.74	0.01	1.00
TraesCS4A02G011000	-0.50	0.55	-0.26	0.77
TraesCS1B02G107100	0.07	0.91	0.07	0.92
TraesCS4D02G209300	0.06	0.93	0.08	0.91
TraesCS1A02G215100	0.16	0.88	-1.06	0.42
TraesCS1A02G137400	-0.10	0.87	-0.02	0.97
TraesCS1A02G315400	0.02	0.97	-0.26	0.69
TraesCS3D02G510000	-0.14	0.80	-0.02	0.98
TraesCS5A02G216000	0.52	0.36	0.14	0.83
TraesCS7B02G176700	-0.13	0.83	-0.07	0.92
TraesCS7B02G328500	-0.11	0.85	-0.12	0.85
TraesCS4D02G076100	0.17	0.84	0.61	0.47
TraesCS7D02G282300	0.11	0.85	-0.13	0.83
TraesCS2A02G094000	0.15	0.80	0.41	0.52
TraesCS2B02G108800	0.25	0.68	0.13	0.84
TraesCS4D02G251200	0.11	0.87	0.04	0.96
TraesCS5D02G122000	-0.32	0.56	0.06	0.92
TraesCS6B02G449800	0.64	0.29	0.28	0.66

	'TAC 75' vs. 'C 306'		'TAC 6' vs. 'C 306'	
**Identifiers**	**Log$_2$ fold change**	***p*-value**	**Log$_2$ fold change**	***p*-value**
TraesCS1A02G198000	-0.25	0.73	-0.15	0.83
TraesCS6B02G178500	-0.59	0.49	-1.55	0.11
TraesCS2B02G389600	0.06	0.91	-0.20	0.75
TraesCS1D02G155700	-0.24	0.73	0.00	1.00
TraesCS7A02G271500	-0.68	0.29	0.10	0.88
TraesCS1B02G304900	-0.10	0.87	-0.18	0.78
TraesCS4D02G292000	0.24	0.73	0.57	0.42
TraesCS5D02G107600	-0.71	0.31	-0.16	0.82
TraesCS5D02G202000	-0.08	0.90	-0.31	0.65
TraesCS3B02G423300	0.34	0.58	-0.45	0.53
TraesCS5A02G095000	-0.10	0.88	0.17	0.80
TraesCS3D02G466100	-0.07	0.89	0.24	0.70
TraesCSU02G068200	0.21	0.73	0.57	0.39
TraesCS5D02G450400	-0.15	0.84	-0.06	0.94
TraesCS4A02G237400	0.06	0.94	0.35	0.69
TraesCS5A02G135100	-0.09	0.87	0.34	0.59
TraesCS7D02G213400	0.05	0.94	0.14	0.85
TraesCS4B02G293400	-0.11	0.86	-0.11	0.87
TraesCS4D02G209500	1.31	0.16	-0.22	0.83
TraesCS3A02G246900	-0.35	0.53	0.19	0.75
TraesCS6A02G255700	-0.24	0.71	0.11	0.87
TraesCS6D02G328700	0.15	0.79	0.16	0.80
TraesCS7D02G222100	0.11	0.83	0.34	0.63
TraesCS1D02G205300	0.47	0.53	0.00	1.00
TraesCS6A02G298300	-0.29	0.67	-0.58	0.43
TraesCS1A02G005700	0.37	0.51	-0.49	0.46
TraesCS3B02G449200	0.33	0.56	0.20	0.76
TraesCS4B02G209100	1.76	0.03	-0.21	0.83
TraesCS2D02G250100	0.13	0.82	0.06	0.93
TraesCS5A02G460200	-0.08	0.91	-0.63	0.37
TraesCS1D02G099500	0.21	0.85	-0.67	0.60
TraesCS2D02G368800	-0.14	0.81	-0.62	0.34
TraesCS5D02G255200	0.79	0.32	-0.33	0.71
TraesCS5B02G101200	-0.22	0.72	0.21	0.74
TraesCS7A02G325800	-0.42	0.49	-0.27	0.67
TraesCS6D02G209000	0.18	0.75	0.07	0.92
TraesCS7A02G189900	-0.97	0.11	-0.65	0.34
TraesCS4A02G290600	0.50	0.32	0.58	0.39

	'TAC 75' vs. 'C 306'		'TAC 6' vs. 'C 306'	
**Identifiers**	**Log_2 fold change**	***p*-value**	**Log_2 fold change**	***p*-value**
TraesCS2B02G625300	-0.03	0.96	-0.73	0.34
TraesCS7B02G226400	-0.03	0.97	0.09	0.90
TraesCS7B02G144100	-0.07	0.92	-0.41	0.60
TraesCS4A02G499200	0.33	0.81	-0.50	0.73
TraesCS1D02G085400	-0.17	0.77	-0.54	0.42
TraesCS5A02G443000	-0.19	0.77	-0.52	0.46
TraesCS1D02G159200	0.06	0.95	-0.20	0.84
TraesCS4B02G077700	-0.53	0.52	-0.84	0.39
TraesCS1D02G209400	-1.42	0.26	-0.13	0.91
TraesCS2A02G400200	0.30	0.73	0.14	0.88
TraesCS1B02G212700	0.09	0.90	0.34	0.68
TraesCS1D02G380000	0.40	0.70	-0.24	0.83
TraesCS2A02G248400	0.17	0.78	-0.13	0.84
TraesCS3D02G244600	-0.13	0.81	0.36	0.57
TraesCS1B02G101700	0.16	0.78	-0.33	0.62
TraesCS7A02G228200	0.17	0.76	0.09	0.89
TraesCS1A02G088100	-0.12	0.83	0.29	0.64
TraesCS1D02G133300	0.20	0.73	0.50	0.42
TraesCS5B02G133900	0.07	0.90	0.30	0.64
TraesCS1A02G158400	0.01	0.99	0.36	0.58
TraesCS1B02G393400	0.01	0.99	-0.25	0.79
TraesCS5D02G239200	0.04	0.93	0.10	0.87
TraesCS4A02G164100	0.29	0.61	0.23	0.70
TraesCS1B02G000900	0.17	0.80	-0.27	0.73
TraesCS5A02G228400	0.07	0.89	0.15	0.81
TraesCS3B02G432700	0.02	0.98	-0.72	0.29
TraesCS4B02G149000	0.04	0.94	0.17	0.78
TraesCS2A02G022100	-0.17	0.76	0.26	0.73
TraesCS5B02G227200	-0.08	0.88	0.10	0.88
TraesCS5B02G111900	-1.26	0.14	-0.33	0.69
TraesCS5D02G096000	-0.79	0.23	-0.24	0.73
TraesCS1B02G152000	0.26	0.64	0.40	0.52
TraesCS1A02G107600	0.01	0.99	0.33	0.60
TraesCS1D02G305300	-0.15	0.78	-0.10	0.87
TraesCS7A02G463200	-0.13	0.81	0.16	0.79
TraesCS6B02G269900	-0.48	0.39	-0.18	0.79
TraesCS2D02G022900	0.05	0.94	0.35	0.67
TraesCS1B02G327300	-0.06	0.92	-0.54	0.42

	'TAC 75' vs. 'C 306'		'TAC 6' vs. 'C 306'	
**Identifiers**	**Log$_2$ fold change**	***p*-value**	**Log$_2$ fold change**	***p*-value**
TraesCS7D02G420400	0.55	0.31	0.36	0.58
TraesCS1A02G084200	0.07	0.90	-0.13	0.84
TraesCS5B02G555800	0.06	0.91	0.14	0.83
TraesCS6A02G346000	-0.17	0.77	-0.12	0.85
TraesCS2A02G385400	-0.12	0.83	0.27	0.67
TraesCS3B02G382600	0.36	0.55	0.02	0.97
TraesCS5B02G447600	-0.11	0.87	0.12	0.87
TraesCS1A02G025700	0.30	0.64	-0.47	0.50
TraesCS3A02G391400	0.20	0.75	-0.65	0.39
TraesCS5B02G246000	0.05	0.95	-0.35	0.64
TraesCS4B02G255400	0.01	0.99	0.06	0.93
TraesCS3A02G288900	0.20	0.76	0.35	0.70
TraesCS3B02G511800	-0.01	0.99	0.13	0.84
TraesCS7A02G211800	-0.08	0.90	-0.05	0.95
TraesCS7B02G191500	0.24	0.71	0.22	0.74
TraesCS4D02G204100	-0.04	0.95	0.02	0.98
TraesCS2B02G162000	-0.10	0.89	0.18	0.81
TraesCS5B02G519400	0.44	0.57	0.39	0.63
TraesCS4D02G255100	1.62	0.59	-	-
TraesCS4A02G322600	0.16	0.77	0.21	0.76
TraesCS3A02G503300	0.84	0.11	0.27	0.69
TraesCS7D02G257900	-0.14	0.84	-0.93	0.26
TraesCS4D02G158400	0.15	0.80	0.16	0.81
TraesCS5A02G312800	-0.21	0.75	-0.18	0.79
TraesCS7B02G118700	0.04	0.95	-0.09	0.90
TraesCS7D02G246300	0.30	0.64	-0.17	0.81
TraesCS3D02G393800	-0.23	0.71	-0.57	0.41
TraesCS3B02G361000	0.57	0.36	-0.05	0.94
TraesCS5A02G189000	0.29	0.62	-0.27	0.69
TraesCS7B02G094900	-0.35	0.56	-0.13	0.84
TraesCS3B02G566700	0.34	0.52	0.22	0.72
TraesCS4A02G222800	-1.45	0.11	-0.73	0.46
TraesCS2D02G382100	-0.12	0.82	0.43	0.50
TraesCS4A02G101400	-0.27	0.70	-0.44	0.55
TraesCS7A02G256500	1.05	0.18	0.52	0.55
TraesCS7D02G191100	-0.34	0.58	-0.22	0.73
TraesCS7D02G473200	-0.05	0.93	0.08	0.91
TraesCS1B02G174400	0.94	0.27	0.22	0.82

	'TAC 75' vs. 'C 306'		'TAC 6' vs. 'C 306'	
**Identifiers**	**Log_2 fold change**	***p*-value**	**Log_2 fold change**	***p*-value**
TraesCS5D02G471300	-0.55	0.44	-0.52	0.47
TraesCS4D02G147800	-0.11	0.86	0.19	0.76
TraesCS4D02G187600	0.12	0.91	-2.42	0.11
TraesCS5A02G467700	0.66	0.26	0.17	0.78
TraesCS7A02G220000	-0.21	0.70	0.12	0.84
TraesCS5D02G014800	0.41	0.48	-0.03	0.97
TraesCS4A02G049500	0.28	0.64	0.16	0.79
TraesCS1D02G089400	0.09	0.87	0.15	0.81
TraesCS6A02G349700	-0.22	0.70	-0.03	0.96
TraesCS1D02G004000	0.53	0.41	-0.03	0.96
TraesCS3D02G079000	0.13	0.82	-0.55	0.41
TraesCS5D02G331600	-0.24	0.78	-1.04	0.26
TraesCS7D02G226400	0.28	0.62	0.17	0.79
TraesCS3A02G365100	0.67	0.37	-0.65	0.40
TraesCS7B02G252000	-0.07	0.90	0.08	0.90
TraesCS1D02G111500	-0.24	0.68	-0.12	0.85
TraesCS5D02G480800	0.76	0.20	0.04	0.95
TraesCSU02G068700	0.15	0.78	-0.01	0.99
TraesCS2D02G141100	-0.17	0.77	-0.07	0.92
TraesCS2D02G092600	-0.35	0.60	0.13	0.84
TraesCS1D02G114200	0.13	0.83	0.00	1.00
TraesCS4A02G165300	0.14	0.80	0.23	0.72
TraesCS7D02G000400	0.01	0.99	-0.84	0.27
TraesCS7B02G163800	0.06	0.92	0.41	0.53
TraesCS1B02G327600	-	-	-	-
TraesCS2D02G129300	0.04	0.96	-0.15	0.82
TraesCS6A02G226100	0.17	0.76	0.31	0.63
TraesCS5B02G373000	-0.15	0.87	-0.83	0.45
TraesCS7A02G534800	-0.04	0.95	-0.48	0.53
TraesCS4D02G021200	0.50	0.37	0.28	0.69
TraesCS5D02G085300	-0.28	0.66	-0.10	0.88
TraesCS4A02G271200	-0.06	0.92	0.30	0.63
TraesCS2B02G193500	-0.70	0.59	-1.29	0.42
TraesCS6B02G382800	-0.37	0.54	0.04	0.96
TraesCS3D02G288700	0.33	0.61	0.30	0.74
TraesCS3B02G272400	-0.19	0.73	0.12	0.84
TraesCS5B02G194700	0.11	0.85	-0.12	0.85
TraesCS4A02G089200	-0.12	0.84	-0.26	0.69

	'TAC 75' vs. 'C 306'		'TAC 6' vs. 'C 306'	
**Identifiers**	**Log_2 fold change**	***p*-value**	**Log_2 fold change**	***p*-value**
TraesCS6B02G296000	-0.26	0.71	-0.79	0.30
TraesCS6D02G236900	-0.36	0.49	-0.14	0.83
TraesCS1A02G206100	-2.84	0.01	-1.07	0.28
TraesCS3D02G324300	0.69	0.24	-0.16	0.81
TraesCS1B02G258900	0.25	0.71	-0.52	0.48
TraesCS7A02G268500	-0.22	0.69	0.21	0.74
TraesCS5D02G518700	0.34	0.59	-0.56	0.44
TraesCS1A02G112600	-0.24	0.68	-0.24	0.69
TraesCS4D02G040200	0.01	0.99	0.47	0.47
TraesCS4B02G215100	-0.14	0.81	-0.17	0.78
TraesCS5B02G007600	0.54	0.47	-0.04	0.96
TraesCS1B02G219700	-1.78	0.08	-0.37	0.74
TraesCS6B02G327800	-0.15	0.83	-0.26	0.71
TraesCS1A02G118800	-0.20	0.76	-0.20	0.76
TraesCS3A02G350000	0.30	0.60	-0.68	0.29
TraesCS1B02G316300	-0.39	0.49	-0.50	0.44
TraesCS4B02G042900	0.20	0.70	0.32	0.63
TraesCS4D02G215600	0.02	0.97	-0.02	0.97
TraesCS4B02G203200	-0.23	0.71	0.10	0.88
TraesCS5A02G325400	0.41	0.50	-0.24	0.72
TraesCSU02G056200	0.03	0.96	0.34	0.60
TraesCS1B02G132800	0.12	0.82	0.10	0.87
TraesCS7A02G340400	-0.04	0.94	0.16	0.80
TraesCS6D02G278100	0.37	0.51	0.54	0.38
TraesCS7D02G522000	-0.49	0.39	-0.45	0.53
TraesCS3B02G552300	-0.02	0.97	-0.49	0.44
TraesCS1B02G138300	-0.22	0.73	-0.29	0.67
TraesCS7B02G127400	-0.16	0.76	0.20	0.77
TraesCS5B02G480100	0.37	0.52	-0.04	0.95
TraesCS1B02G129600	0.07	0.89	0.36	0.57
TraesCS5D02G380200	-0.30	0.73	0.55	0.57
TraesCS3D02G344000	-0.25	0.70	-0.48	0.46
TraesCS1D02G103800	0.10	0.85	-0.02	0.98
TraesCS2A02G364100	0.53	0.34	0.04	0.94
TraesCS2B02G382300	0.28	0.62	-0.07	0.91
TraesCS2B02G148700	-0.04	0.95	0.13	0.85
TraesCS2A02G126800	0.01	0.99	-0.47	0.50
TraesCS2B02G596600	-0.25	0.69	0.06	0.93

	'TAC 75' vs. 'C 306'		'TAC 6' vs. 'C 306'	
**Identifiers**	**Log_2 fold change**	***p*-value**	**Log_2 fold change**	***p*-value**
TraesCS5B02G489500	-0.49	0.66	0.46	0.68
TraesCS4B02G023600	0.46	0.43	0.34	0.66
TraesCS4D02G221000	-0.10	0.87	-0.17	0.80
TraesCS1A02G169300	-0.36	0.55	0.05	0.93
TraesCS5A02G253700	0.82	0.45	-0.70	0.55
TraesCS4D02G222000	0.40	0.53	0.00	1.00
TraesCS1B02G187600	0.22	0.77	0.80	0.29
TraesCS4A02G352200	0.42	0.54	0.37	0.60
TraesCS6D02G332200	-0.51	0.36	-0.27	0.66
TraesCS7D02G513500	0.11	0.83	0.23	0.76
TraesCS6B02G255500	-0.05	0.93	0.04	0.94
TraesCS5B02G281700	-0.44	0.49	-0.11	0.87
TraesCS5D02G289400	-0.93	0.17	-0.03	0.97
TraesCS2D02G599700	-0.31	0.64	-0.10	0.88
TraesCS5D02G553700	0.16	0.78	0.39	0.60
TraesCS7D02G266300	0.02	0.97	0.26	0.67
TraesCS5B02G325800	0.19	0.75	-0.28	0.67
TraesCS7B02G452400	-0.44	0.55	-0.36	0.68
TraesCS1D02G119700	-0.18	0.80	0.11	0.87
TraesCS1B02G123100	-0.02	0.97	0.15	0.81
TraesCS2D02G361900	-0.12	0.83	-0.48	0.44
TraesCS7A02G524600	0.09	0.88	0.16	0.83
TraesCS5D02G489900	-1.00	0.24	-0.25	0.78
TraesCS3A02G001800	-0.22	0.70	0.15	0.81
TraesCS4A02G082400	-0.04	0.95	-0.36	0.56
TraesCS2A02G586800	-0.41	0.59	-0.76	0.38
TraesCS3B02G323600	0.11	0.88	0.15	0.87
TraesCS2B02G030700	-0.02	0.98	0.36	0.64
TraesCS3A02G425600	0.10	0.86	-0.07	0.92
TraesCS6A02G274400	-0.54	0.46	-0.05	0.95
TraesCS3A02G330900	0.23	0.68	-0.29	0.64
TraesCS4B02G220700	0.05	0.93	-0.16	0.81
TraesCS5D02G319600	0.05	0.94	0.12	0.86
TraesCS5A02G072600	-0.89	0.20	-0.06	0.93
TraesCS6B02G301900	-0.16	0.77	0.06	0.92
TraesCS5A02G282300	-0.62	0.36	-0.02	0.97
TraesCS1D02G167300	-0.04	0.95	-0.10	0.88
TraesCS5B02G252600	0.22	0.70	-0.29	0.66

	'TAC 75' vs. 'C 306'		'TAC 6' vs. 'C 306'	
**Identifiers**	**Log_2 fold change**	***p*-value**	**Log_2 fold change**	***p*-value**
TraesCS7B02G442100	0.06	0.92	0.11	0.89
TraesCS2D02G397800	0.08	0.92	-0.45	0.64
TraesCS6D02G383800	-0.22	0.68	-0.34	0.62
TraesCS4A02G083500	0.15	0.79	-0.07	0.91
TraesCS3B02G461900	-	-	-	-
TraesCS6B02G439800	-0.37	0.49	-0.33	0.64
TraesCS3D02G497200	-0.10	0.86	0.10	0.88
TraesCS2B02G419400	-0.58	0.31	-0.47	0.48
TraesCS5A02G476600	-1.46	0.13	-1.24	0.25
TraesCS6A02G399600	-0.47	0.42	-0.09	0.89
TraesCS5B02G078400	-0.61	0.38	-0.34	0.66
TraesCS6D02G254700	-0.13	0.83	-0.20	0.76
TraesCS3D02G420400	-0.25	0.68	-0.05	0.94
TraesCS2A02G401400	-0.30	0.59	-0.24	0.70
TraesCS2D02G398700	-0.20	0.71	-0.06	0.92
TraesCS3B02G000800	0.52	0.53	-0.52	0.55
TraesCS3A02G491300	-0.23	0.66	-0.36	0.59

Supplemental Table 7. List of primer sequences used for real-time quantitative gene expression analysis (qRT-PCR) of36 RING protein genes.

Identifiers	Forward/Reverse	Primer sequences (5'-3')	Tm (°C)
TraesCS2B02G182300	Forward<br>Reverse	CTCCTTCCCTCCCTCCCA<br>CCGCTGAAGATGAAGTGGC	62.52<br>61.91
TraesCS1A02G206100	Forward<br>Reverse	GTTGCGTCGTCCGAGTTTTAG<br>TTCTAGCGGACAACATCCACTC	62.06<br>61.56
TraesCS4A02G095800	Forward<br>Reverse	TCCTGGAAGAACAAGCCC<br>TACAGAAGTGAGAACGCCG	58.72<br>57.56
TraesCS3B02G461900	Forward<br>Reverse	TGAAACCAACAGCATACCG<br>AATGGAGGCCTTGATGCATTT	58.11<br>62.90
TraesCS3B02G323600	Forward<br>Reverse	GGCTTCCTCCATATTTTGGACA<br>ACTCATCAGACTCGGAACCATTG	62.35<br>62.68
TraesCS4D02G339200	Forward<br>Reverse	GCCAACCAACTCCCTCCC<br>TGCTAGCTCCTCCCGACG	62.83<br>62.64
TraesCS2A02G156800	Forward<br>Reverse	CGCTCCCATGGACTCGAC<br>CGCCGAGGATGAAGTGAT	62.87<br>59.74
TraesCS3A02G399700	Forward<br>Reverse	CCTCTCTCTCTCTTGTTCCTTGG<br>CATCCCTCTCTCTCTTCCCCTC	60.89<br>62.82
TraesCS5A02G049400	Forward<br>Reverse	GCTGTGCAGAAAACGATGTAG<br>TTGAAACCGCGAGTAGATCACA	58.59<br>62.95
TraesCS2A02G099300	Forward<br>Reverse	GCGTCGTTGTCCACGAGA<br>GCTCGCGATATGCCGATA	62.11<br>60.85
TraesCS4A02G322600	Forward<br>Reverse	GCGGAGGCTTCCAAGGAT<br>CCATGCTCTAGTCAAGGACTCA	62.62<br>59.50
TraesCS7B02G150000	Forward<br>Reverse	CCCTCTCACGCTTTTCAATTTTA<br>TTTGTTCGATTATCTTGTTCCTT	61.71<br>57.05
TraesCS5B02G078400	Forward<br>Reverse	ACCACCACCACCACCACC<br>ATCCGACACACTGCGGTT	62.72<br>60.12
TraesCS6D02G254700	Forward<br>Reverse	GGGCGAAGTTATTTCATGGTTA<br>AGTAGCTCCCAGGGACGATTT	60.20<br>61.34
TraesCS6B02G286500	Forward<br>Reverse	GGTTTACATCATCTCAAAATTGG<br>GATCCACTAACCAAACGCTAGT	57.56<br>57.46
TraesCS6A02G274400	Forward<br>Reverse	ATCGAGTGCCAGGCGAAC<br>GTCTTGAGCCAACGGCTGAT	62.40<br>62.68
TraesCS6B02G385100	Forward<br>Reverse	TCGCCATTGTCCAACGAG<br>TGAGGGATCTGGTGGGTTTTT	61.85<br>62.85
TraesCS2D02G162600	Forward<br>Reverse	GGAGAGGCCACAGCAGGT<br>CTCTCCTCTCCTCTTCAGCAACT	61.43<br>60.68
TraesCS6B02G301900	Forward<br>Reverse	GCGAAGTTGCTTCATGGTTG<br>AGTACCCACCAAATTCACACTTC	61.75<br>59.31

Identifiers	Forward/Reverse	Primer sequences (5'-3')	Tm (°C)
TraesCS7D02G257900	Forward	CTGCCTTTTCCAAGTATTGC	57.52
	Reverse	CAGAAGGGCATGCAAATTCA	62.08
TraesCS5A02G135100	Forward	GCAAGCAACGGTGAAGTTTCT	61.71
	Reverse	TGAACATCTGCCTGAACTGAGC	62.82
TraesCS2B02G336400	Forward	CAGCAAAGAAAAGACTGCGAT	59.65
	Reverse	TTTTTGAGCTTTGGAGTGACA	58.54
TraesCS4D02G147800	Forward	AAATCGCGTGTTGATTAGACGAC	62.54
	Reverse	AACAAATCGAACTCCTTGCC	59.17
TraesCS3B02G120600	Forward	GGGTTGCTGTAGCTGTGTTATG	59.70
	Reverse	AAGGGAATTACCAGAGCAAGTAG	58.07
TraesCS4B02G164000	Forward	AGTAGAGGTTAGGGAATGGACG	60.90
	Reverse	ATGTCGGCGGGATAAGCGA	59.50
TraesCS4A02G112900	Forward	CTCGATCGTGCATGCTTGT	61.01
	Reverse	GAATGCAAGCTCATAGTCACAA	58.05
TraesCS7B02G118700	Forward	GAATTGAACTGCTTAGTGGACTG	58.07
	Reverse	TTAGCTAACAAGTTGGTCCAAAG	57.72
TraesCS3A02G095400	Forward	GGCATGCAATATTTGTAATCCTA	58.12
	Reverse	CCTTGTTCAGGAAAAAGCGA	60.36
TraesCS6D02G278100	Forward	ACTGCCCGCTTTGCAAGT	61.95
	Reverse	AGACAGGAATCACAAGGCTTCTC	61.14
TraesCS3B02G000800	Forward	TGCGCAACTAATGCTATCGTA	59.52
	Reverse	ACCACCACACTCAACATATCTCT	57.59
TraesCS1A02G341400	Forward	GAGGAAGAAGGGTCGGGG	61.93
	Reverse	TTGACCTCGATCTGACTACCTTC	59.77
TraesCS4B02G255400	Forward	AGTAGGGGAACATTACGCAACA	60.77
	Reverse	GCCCTATGAAAATGCCCT	57.55
TraesCS2D02G315900	Forward	CCAATAATGTGTTACCCGATCA	59.59
	Reverse	TTCTCTATTCTTGTTCTGGGTTG	57.56
TraesCS1B02G327600	Forward	AACAACGCGACAGAGGTTCC	62.56
	Reverse	GCTTGTAGAACCTGAAGAATCGG	61.45
TraesCS5D02G188300	Forward	GTTAGGCTTGGTTCACCAAACG	62.10
	Reverse	AGCACACAACAGAATGCCCTCA	62.10
TraesCS4B02G209100	Forward	TTTCAATTGATGATAGTGGTGAA	57.18
	Reverse	ATTTCTGAATAGTTTGCTCATGG	57.48

Supplemental Table 8.The normalized expression values (ΔCt) of 36 RING protein genes from qRT-PCR analysis at four developmental stages (7, 14, 21 and 28 days after anthesis (DAA)) in three genotypes (TAC 75, TAC 6 and C 306). The average normalized expression values of three each biological and technical replicates are given with mean ± standard deviation.

Identifiers	DAA	ΔCt (3 biological replicates average)		
		'TAC 75'	'C 306'	'TAC 6'
TraesCS2B02G182300	7	10.46 ± 0.18	13.81 ± 0.21	10.54 ± 0.29
TraesCS2B02G182300	14	11.47 ± 0.22	16.03 ± 0.35	10.68 ± 0.15
TraesCS2B02G182300	21	9.12 ± 0.7	13.22 ± 0.55	10.71 ± 0.54
TraesCS2B02G182300	28	11.71 ± 0.49	16.19 ± 0.47	11.1 ± 0.76
TraesCS1A02G206100	7	2.64 ± 0.36	5.23 ± 0.21	4.05 ± 0.27
TraesCS1A02G206100	14	3.21 ± 1.29	4.89 ± 0.69	1.67 ± 0.16
TraesCS1A02G206100	21	6.39 ± 0.66	6.99 ± 0.77	3.93 ± 0.46
TraesCS1A02G206100	28	2.46 ± 0.16	2.67 ± 0.22	2.49 ± 0.12
TraesCS4A02G095800	7	7.92 ± 0.66	7.59 ± 1.01	8.32 ± 0.47
TraesCS4A02G095800	14	5.41 ± 0.47	5.73 ± 0.55	5.13 ± 0.54
TraesCS4A02G095800	21	6.17 ± 0.41	8.39 ± 0.46	5.51 ± 0.25
TraesCS4A02G095800	28	7.72 ± 0.32	8.93 ± 0.46	7.35 ± 0.41
TraesCS3B02G461900	7	6.67 ± 0.29	7.07 ± 0.5	5.19 ± 0.52
TraesCS3B02G461900	14	6.45 ± 0.48	5.78 ± 0.44	5.34 ± 0.56
TraesCS3B02G461900	21	8.91 ± 0.41	5.36 ± 0.63	6.43 ± 0.75
TraesCS3B02G461900	28	9.79 ± 0.42	5.14 ± 0.57	4.67 ± 0.28
TraesCS3B02G323600	7	-3.38 ± 0.4	-5.15 ± 0.46	-2.26 ± 0.26
TraesCS3B02G323600	14	-2.16 ± 0.51	-4.6 ± 0.17	-3.81 ± 0.46
TraesCS3B02G323600	21	-1.67 ± 0.48	-3.27 ± 0.48	-2.11 ± 0.28
TraesCS3B02G323600	28	-0.66 ± 0.42	-3.31 ± 0.28	-5.26 ± 0.52
TraesCS4D02G339200	7	9.79 ± 0.54	11.01 ± 0.88	11.47 ± 0.36
TraesCS4D02G339200	14	5.67 ± 0.24	9.3 ± 0.46	5.78 ± 0.2
TraesCS4D02G339200	21	9.54 ± 0.88	12.73 ± 0.55	9.55 ± 0.47
TraesCS4D02G339200	28	6.39 ± 0.2	12.7 ± 0.37	8.19 ± 0.38
TraesCS2A02G156800	7	10.48 ± 1.11	12.23 ± 0.83	13.18 ± 0.85
TraesCS2A02G156800	14	5.44 ± 0.5	9.19 ± 0.42	6.02 ± 0.61
TraesCS2A02G156800	21	12.23 ± 0.38	7.05 ± 0.49	12.15 ± 0.42
TraesCS2A02G156800	28	14.96 ± 0.61	12.05 ± 0.71	13.74 ± 0.76
TraesCS3A02G399700	7	9.28 ± 0.58	11.02 ± 0.6	11.66 ± 0.49
TraesCS3A02G399700	14	6.57 ± 0.2	7.13 ± 0.26	7.27 ± 0.33
TraesCS3A02G399700	21	10.85 ± 0.47	6.89 ± 0.3	10.63 ± 0.3
TraesCS3A02G399700	28	10.88 ± 0.26	8.81 ± 0.24	10.37 ± 0.42
TraesCS5A02G049400	7	12.28 ± 0.43	10.15 ± 0.46	10.17 ± 0.48
TraesCS5A02G049400	14	11.53 ± 0.65	6.76 ± 0.3	7.24 ± 0.47
TraesCS5A02G049400	21	11.78 ± 0.48	3.15 ± 0.56	7.08 ± 0.32

Identifiers	DAA	ΔCt (3 biological replicates average)		
		'TAC 75'	'C 306'	'TAC 6'
TraesCS5A02G049400	28	13.1 ± 1.11	4.25 ± 0.42	7.09 ± 0.58
TraesCS2A02G099300	7	10.85 ± 1.59	12.93 ± 0.27	8.86 ± 0.49
TraesCS2A02G099300	14	7.1 ± 0.48	9.78 ± 0.46	6.51 ± 0.41
TraesCS2A02G099300	21	6.96 ± 0.61	6.89 ± 0.2	7.63 ± 0.22
TraesCS2A02G099300	28	10.02 ± 1.07	10.91 ± 0.83	9.48 ± 0.41
TraesCS4A02G322600	7	8.66 ± 0.21	12.4 ± 0.4	13.42 ± 0.29
TraesCS4A02G322600	14	8.78 ± 0.87	13.08 ± 0.36	8.6 ± 0.4
TraesCS4A02G322600	21	11.67 ± 0.08	13.94 ± 0.29	8.16 ± 0.21
TraesCS4A02G322600	28	8.63 ± 0.2	10.4 ± 0.2	7.46 ± 0.34
TraesCS7B02G150000	7	5.47 ± 0.13	5.33 ± 0.16	6.56 ± 0.38
TraesCS7B02G150000	14	6.08 ± 0.44	9 ± 0.31	7.82 ± 0.19
TraesCS7B02G150000	21	6.65 ± 0.24	8.25 ± 0.39	10.06 ± 0.58
TraesCS7B02G150000	28	6.44 ± 0.21	14.08 ± 0.38	8.78 ± 0.25
TraesCS5B02G078400	7	7.56 ± 0.28	6.58 ± 0.18	4.07 ± 0.33
TraesCS5B02G078400	14	4.13 ± 0.54	3.19 ± 0.35	-0.43 ± 0.14
TraesCS5B02G078400	21	4.6 ± 0.22	2.24 ± 0.22	4.4 ± 0.23
TraesCS5B02G078400	28	4.13 ± 0.37	2.17 ± 0.2	3.54 ± 0.18
TraesCS6D02G254700	7	14.04 ± 1.13	6.43 ± 0.98	5.69 ± 0.89
TraesCS6D02G254700	14	9.58 ± 0.37	4.89 ± 0.09	4.73 ± 0.21
TraesCS6D02G254700	21	10.32 ± 0.29	6.65 ± 0.23	6 ± 0.14
TraesCS6D02G254700	28	13.36 ± 0.86	2.47 ± 1.17	3.62 ± 1.53
TraesCS6B02G286500	7	10.82 ± 1.53	9.69 ± 1.79	9.72 ± 1.03
TraesCS6B02G286500	14	8.56 ± 0.41	10.18 ± 0.33	14.47 ± 0.22
TraesCS6B02G286500	21	6.13 ± 0.64	14.26 ± 0.69	15.25 ± 1.16
TraesCS6B02G286500	28	5.51 ± 1.73	13.47 ± 0.28	15.73 ± 0.2
TraesCS6A02G274400	7	13.06 ± 0.35	9.12 ± 0.32	9.78 ± 0.28
TraesCS6A02G274400	14	13.05 ± 0.51	8.62 ± 0.29	7.92 ± 0.25
TraesCS6A02G274400	21	7.71 ± 0.53	6.59 ± 0.48	7.74 ± 0.45
TraesCS6A02G274400	28	10.99 ± 0.39	8.09 ± 0.5	8.63 ± 0.42
TraesCS6B02G385100	7	12.57 ± 0.14	11.71 ± 0.17	12.1 ± 0.2
TraesCS6B02G385100	14	14.25 ± 0.33	17.35 ± 0.6	12.85 ± 0.21
TraesCS6B02G385100	21	9.05 ± 0.16	12.64 ± 0.17	10.45 ± 0.27
TraesCS6B02G385100	28	11.22 ± 0.27	12.29 ± 0.2	10.18 ± 0.43
TraesCS2D02G162600	7	11.93 ± 0.54	6.97 ± 0.49	4.58 ± 0.3
TraesCS2D02G162600	14	13.72 ± 0.22	9.19 ± 0.84	5.05 ± 0.15
TraesCS2D02G162600	21	13.62 ± 0.34	5.46 ± 0.51	5.16 ± 0.19
TraesCS2D02G162600	28	10.91 ± 0.13	7.83 ± 0.7	6.31 ± 0.24
TraesCS6B02G301900	7	8.44 ± 1.06	8.36 ± 0.59	8.23 ± 0.66
TraesCS6B02G301900	14	7.06 ± 0.67	6.94 ± 0.54	6.17 ± 0.54

Identifiers	DAA	ΔCt (3 biological replicates average)		
		'TAC 75'	'C 306'	'TAC 6'
TraesCS6B02G301900	21	13.93 ± 0.98	10.31 ± 1.22	9.85 ± 0.81
TraesCS6B02G301900	28	11.66 ± 0.82	6.66 ± 0.52	8.36 ± 0.62
TraesCS7D02G257900	7	9.83 ± 1.18	10.07 ± 0.53	9.48 ± 0.87
TraesCS7D02G257900	14	10.18 ± 0.26	10.27 ± 0.78	5.48 ± 0.41
TraesCS7D02G257900	21	6.28 ± 0.35	5.78 ± 0.95	6.51 ± 0.28
TraesCS7D02G257900	28	10.9 ± 0.6	9.25 ± 0.27	7.13 ± 0.51
TraesCS5A02G135100	7	14.43 ± 0.97	10.11 ± 0.23	13.55 ± 0.65
TraesCS5A02G135100	14	12.23 ± 0.39	11.03 ± 0.23	12.92 ± 0.22
TraesCS5A02G135100	21	11.22 ± 1.06	11.95 ± 0.43	12.81 ± 0.79
TraesCS5A02G135100	28	8.42 ± 0.37	8.43 ± 0.24	8.45 ± 0.43
TraesCS2B02G336400	7	7.28 ± 0.56	11.35 ± 0.08	9.49 ± 0.71
TraesCS2B02G336400	14	8.5 ± 0.15	12.57 ± 0.08	9.67 ± 0.17
TraesCS2B02G336400	21	6.17 ± 0.4	10.45 ± 0.55	9.08 ± 0.45
TraesCS2B02G336400	28	5.28 ± 0.5	12.64 ± 0.3	6.63 ± 0.17
TraesCS4D02G147800	7	10.47 ± 0.41	9.59 ± 0.49	10.36 ± 0.74
TraesCS4D02G147800	14	10.72 ± 0.48	10.27 ± 0.54	10.62 ± 0.5
TraesCS4D02G147800	21	10.58 ± 0.29	10.48 ± 0.53	7.79 ± 0.24
TraesCS4D02G147800	28	8.98 ± 0.47	5.83 ± 0.57	6.44 ± 0.41
TraesCS3B02G120600	7	11.65 ± 0.8	7.74 ± 0.59	13.25 ± 0.67
TraesCS3B02G120600	14	12.53 ± 0.28	9.38 ± 0.44	13.94 ± 0.87
TraesCS3B02G120600	21	9.25 ± 0.36	10.43 ± 0.22	5.4 ± 0.16
TraesCS3B02G120600	28	11.45 ± 0.48	10.01 ± 0.47	8.09 ± 0.42
TraesCS4B02G164000	7	6.64 ± 0.4	9.95 ± 0.18	9.72 ± 0.6
TraesCS4B02G164000	14	7.45 ± 0.4	9.41 ± 0.34	9.79 ± 0.38
TraesCS4B02G164000	21	7.63 ± 0.17	10.38 ± 0.41	8.79 ± 0.14
TraesCS4B02G164000	28	5.23 ± 0.3	11.07 ± 0.46	7.43 ± 0.26
TraesCS4A02G112900	7	7.56 ± 0.26	13.06 ± 0.78	14.52 ± 0.48
TraesCS4A02G112900	14	9 ± 0.38	13.22 ± 0.47	14.2 ± 0.52
TraesCS4A02G112900	21	9.52 ± 0.27	13.8 ± 0.33	14.52 ± 0.11
TraesCS4A02G112900	28	10.12 ± 0.68	13.91 ± 0.87	14.51 ± 0.19
TraesCS7B02G118700	7	8.79 ± 0.55	11.82 ± 0.55	6.8 ± 0.6
TraesCS7B02G118700	14	6.07 ± 0.74	8.44 ± 0.44	4.91 ± 0.52
TraesCS7B02G118700	21	13.82 ± 0.61	7.97 ± 0.83	8.74 ± 0.66
TraesCS7B02G118700	28	12.78 ± 0.54	8.79 ± 0.41	7.6 ± 0.7
TraesCS3A02G095400	7	7.97 ± 1	8.15 ± 0.63	10.5 ± 0.41
TraesCS3A02G095400	14	4.55 ± 0.27	6.72 ± 0.42	5 ± 0.08
TraesCS3A02G095400	21	6.06 ± 0.49	8.75 ± 0.41	6.23 ± 0.56
TraesCS3A02G095400	28	7.56 ± 0.25	10.38 ± 0.41	8.7 ± 0.3
TraesCS6D02G278100	7	11.74 ± 1.48	12.36 ± 1.15	9.14 ± 1.03

Identifiers	DAA	ΔCt (3 biological replicates average)		
		'TAC 75'	'C 306'	'TAC 6'
TraesCS6D02G278100	14	13.3 ± 0.82	10.45 ± 0.91	14.14 ± 1.04
TraesCS6D02G278100	21	14.17 ± 0.66	4.05 ± 0.79	10.97 ± 0.56
TraesCS6D02G278100	28	12.95 ± 1.08	4.95 ± 0.99	6.6 ± 0.83
TraesCS3B02G000800	7	10.63 ± 0.29	6.76 ± 0.2	5.26 ± 0.33
TraesCS3B02G000800	14	11.63 ± 0.23	9.09 ± 0.25	7.57 ± 0.28
TraesCS3B02G000800	21	11.33 ± 0.45	8.95 ± 0.3	7.16 ± 0.31
TraesCS3B02G000800	28	11.74 ± 0.37	8.14 ± 0.24	6.89 ± 0.26
TraesCS1A02G341400	7	5.84 ± 0.57	11.8 ± 0.57	10.3 ± 0.51
TraesCS1A02G341400	14	8.37 ± 0.48	13.96 ± 0.24	10.42 ± 0.41
TraesCS1A02G341400	21	4.77 ± 0.31	10.73 ± 0.26	9.38 ± 0.37
TraesCS1A02G341400	28	0.87 ± 2.41	14.21 ± 0.44	12.79 ± 0.33
TraesCS4B02G255400	7	15.03 ± 0.53	9.78 ± 0.62	10.98 ± 0.24
TraesCS4B02G255400	14	11.88 ± 0.43	10.01 ± 0.26	11.8 ± 0.43
TraesCS4B02G255400	21	11.66 ± 0.39	6.84 ± 0.43	9.12 ± 0.39
TraesCS4B02G255400	28	13.62 ± 0.26	5.59 ± 0.44	8.54 ± 0.29
TraesCS2D02G315900	7	4.76 ± 0.53	5.11 ± 0.7	6.68 ± 0.41
TraesCS2D02G315900	14	10.99 ± 1.66	6.96 ± 0.4	5.66 ± 0.39
TraesCS2D02G315900	21	9.95 ± 0.48	7 ± 0.08	9.39 ± 0.86
TraesCS2D02G315900	28	10.71 ± 0.41	5.2 ± 0.5	5.43 ± 0.42
TraesCS1B02G327600	7	8.4 ± 0.38	10.11 ± 0.14	11.62 ± 0.33
TraesCS1B02G327600	14	12.04 ± 0.46	11.85 ± 0.5	13.12 ± 0.31
TraesCS1B02G327600	21	12.88 ± 0.13	11.54 ± 0.3	12.76 ± 0.35
TraesCS1B02G327600	28	11.95 ± 0.38	11.52 ± 0.32	13.21 ± 0.24
TraesCS5D02G188300	7	6.92 ± 0.37	8.22 ± 0.74	11.03 ± 0.38
TraesCS5D02G188300	14	10.87 ± 0.3	13.29 ± 0.31	12.85 ± 0.35
TraesCS5D02G188300	21	12.44 ± 0.3	10.64 ± 0.4	13.78 ± 0.42
TraesCS5D02G188300	28	12.12 ± 0.52	11.79 ± 0.35	13.14 ± 0.38
TraesCS4B02G209100	7	8.95 ± 0.32	9.26 ± 0.49	8.19 ± 0.17
TraesCS4B02G209100	14	3.01 ± 0.3	9.51 ± 0.39	5.8 ± 0.13
TraesCS4B02G209100	21	8.69 ± 0.68	9.96 ± 0.94	9.84 ± 0.96
TraesCS4B02G209100	28	11.7 ± 0.31	12.94 ± 0.21	11.55 ± 0.35

CLUSTAL 2.1 MULTIPLE SEQUENCE ALIGNMENT

File: C:/Users/HP/Downloads/clustal alignBB1.ps **Date: Wed May 20 03:00:39 2020**


```
                    *  *                                                                             *                 *                                                                        *
TraesCS1B02G219500  CPVCMNAWTA-------------------------DGEHRVS------------------------------------CIPCG--HVY-----GR---SC-LEKWLAQ---------------------------------------------------------CGKKSATCPQC  47
TraesCS1D02G209200  CPVCMNAWTA-------------------------DGEHRVS------------------------------------CIPCG--HVY-----GR---SC-LEKWLAQ---------------------------------------------------------CGKKSATCPQC  47
TraesCS5A02G547800  CPVCMEPWTS-------------------------EGEHRIS------------------------------------CIPCG--HVY-----GR---SC-LERWLTQ---------------------------------------------------------RGNASATCPQC  47
TraesCS4B02G380900a CPCCTEPWTS-------------------------DGPHRMC------------------------------------CIPCG--HVY-----GR---SC-METLLHR---------------------------------------------------------SGQNSAKCPQC  47
TraesCS4B02G381000a CPCCTEPWTS-------------------------DGPHRMC------------------------------------CIRCG--HVY-----GR---SC-METLLRR---------------------------------------------------------SGQNSAKCPQC  47
TraesCS5A02G547400a CPACTEPWSS-------------------------DGPHRMC------------------------------------CIPCG--HVY-----GR---SC-METLLGR---------------------------------------------------------SGQNSAKCPQC  47
TraesCS4B02G381000b CSICMEPWTS-------------------------TDEHHIWRLWVHDAADVAPYDHIVQEGSLCWTFHDHLPSPTCSCIPCG--HVY-----GR---SC-LIRWLRDQ-------------------------------------------------------LSRNENLKCPQC  85
TraesCS2A02G526800a CPMCMETAPI-------------------------ILKSRFG--------------------------------------SCD-DHVF-----CS---SC-VAKYVAVKQR-----------------------------------------------------QDVALPVECPDC  50
TraesCS2A02G546000a CPMCITTAPI-------------------------VHKSSFG--------------------------------------SCD-HHAF-----CS---NC-VAKYVAMRQR-----------------------------------------------------EGVALPVECPDC  50
TraesCS2B02G144500a CPMCMELVPI-------------------------NLKSSFG--------------------------------------PCG--HAF-----CS---SC-VTMYVAVKQRER---------------------------------------------------EDVGR-VKCPDC  50
TraesCS2D02G125100a CPMCMELVPI-------------------------NLKSSLG--------------------------------------PCG--HAF-----CS---SC-VTTYVAVKQRER---------------------------------------------------EDAAR-VKCPDC  50
TraesCS2D02G187100a CAICMETVPG-------------------------VLKLSVG--------------------------------------SCG--HAF-----CS---SC-VVQYVAAKLG-----------------------------------------------------EN----VACVKC  45
TraesCS2D02G547300a CPMCTTTAPI-------------------------IHKSSFG--------------------------------------SCG--HAF-----CN---SC-VAKYIAVRQR-----------------------------------------------------ENVALPVECPEC  49
TraesCS7D02G415800a CPMCTTTAPI-------------------------IHKSSFG--------------------------------------SCG--HAF-----CN---SC-VATYIAVRQR-----------------------------------------------------ENAALPVECPDC  49
TraesCS1A02G201700  CAICLTEF-------------GEREE-----------VRVMPQ--------------------------------------CG--HGF-----HV---DC-VDTWLRSN-------------------------------------------------------------SSCPSC  42
TraesCS1B02G216000  CAICLTEF-------------GEREE-----------VRVMPQ--------------------------------------CG--HGF-----HV---DC-VDTWLRSN-------------------------------------------------------------SSCPSC  42
TraesCS1D02G205300  CAICLTEF-------------GEREE-----------VRVMPQ--------------------------------------CG--HGF-----HV---EC-VDTWLRSN-------------------------------------------------------------SSCPSC  42
TraesCS4B02G322200  CAICLSEY-------------AGKEE-----------IRVLPQ--------------------------------------CG--HGF-----HV---AC-VDAWLRAH-------------------------------------------------------------SSCPSC  42
TraesCS4D02G318800  CAICLSEF-------------AGKEE-----------IRVLPQ--------------------------------------CG--HGF-----HV---AC-VDAWLRAH-------------------------------------------------------------SSCPSC  42
TraesCS5A02G494500  CAICLSEF-------------AEKEE-----------IRVLPQ--------------------------------------CG--HAF-----HV---AC-VDAWLRAH-------------------------------------------------------------SSCPSC  42
TraesCS2B02G477500  CAICLAEF-------------EDGEA-----------MRVLPQ--------------------------------------CG--HAF-----HA---AC-VDKWLRGH-------------------------------------------------------------SSCPSC  42
TraesCS2D02G455800  CAICLAEF-------------EDGEA-----------MRVLPQ--------------------------------------CG--HAF-----HA---AC-VDKWLRGH-------------------------------------------------------------SSCPSC  42
TraesCS2A02G455400  CAICLAEF-------------EDGEA-----------MRVLPQ--------------------------------------CG--HAF-----HA---AC-VDKWLRGH-------------------------------------------------------------SSCPSC  42
TraesCS6A02G261400  CAICLAEF-------------EDGQH-----------MRVLPQ--------------------------------------CS--HAF-----HA---AC-VDAWLRSH-------------------------------------------------------------SSCPSC  42
TraesCS6D02G242100  CAICLAEF-------------EDGQH-----------MRVLPQ--------------------------------------CS--HAF-----HA---AC-VDAWLRSH-------------------------------------------------------------SSCPSC  42
TraesCS6B02G288700  CAICLAEF-------------EDGQD-----------MRVLPQ--------------------------------------CS--HAF-----HT---AC-VDAWLRSH-------------------------------------------------------------SSCPSC  42
TraesCS5A02G354600  CAICLAEF-------------APGDE-----------VRVLPT--------------------------------------CG--HGF-----HA---AC-VDVWLLSS-------------------------------------------------------------STCPSC  42
TraesCS5D02G361800  CAICLAEF-------------APGDE-----------VRVLPT--------------------------------------CG--HGF-----HA---AC-VDVWLLSN-------------------------------------------------------------STCPSC  42
TraesCS5D02G361900  CAICLAEF-------------AGGDE-----------VRVLPT--------------------------------------CG--HGF-----HA---AC-VDVWLLSS-------------------------------------------------------------STCPSC  42
TraesCS5A02G354500  CAICLAEF-------------AGGDE-----------ARVLPT--------------------------------------CG--HGF-----HA---AC-VDVWLLSN-------------------------------------------------------------STCPSC  42
TraesCS5B02G356800  CAICLAEF-------------APGNE-----------VRVLPT--------------------------------------CG--HDF-----HA---AC-VDVWLLSN-------------------------------------------------------------STCPSC  42
TraesCS5A02G354700  CAICLAEF-------------TRGDE-----------VRVLPH--------------------------------------CG--HGF-----HA---AC-VDVWLLSS-------------------------------------------------------------STCPSC  42
TraesCS5D02G362000  CAICLAEF-------------TRGDE-----------VRVLPT--------------------------------------CG--HGF-----HA---AC-VDVWLLSS-------------------------------------------------------------STCPSC  42
TraesCS5A02G354800  CAICLAEL-------------ARGDE-----------VRVLPH--------------------------------------CG--HGF-----HA---AC-VDVWLLSS-------------------------------------------------------------STCPSC  42
TraesCS5D02G362100  CAICLAEF-------------ARGDE-----------VRVLPH--------------------------------------CG--HGF-----HA---AC-VDVWLLSS-------------------------------------------------------------STCPSC  42
TraesCS5B02G356900  CAICLVEF-------------ARGDE-----------VRVLPT--------------------------------------CG--HGF-----HA---AC-VDVWLLSS-------------------------------------------------------------STCPSC  42
TraesCS5B02G357000  CAICLAAF-------------ARGDE-----------VRVLPR--------------------------------------CG--HGF-----HA---AC-VDVWLLSS-------------------------------------------------------------STCPSC  42
TraesCS6B02G467800  CAICLADL-------------VAGEE-----------LRVLPR--------------------------------------CG--HSF-----HV---AC-VDAWLRTC-------------------------------------------------------------ATCPSC  42
TraesCS6D02G405300  CAICLADL-------------ATGEE-----------LRVLPR--------------------------------------CG--HSF-----HV---AC-VDAWLRTC-------------------------------------------------------------ATCPTC  42
TraesCS6A02G420200  CAICLADL-------------AAGEE-----------LRVLPR--------------------------------------CG--HSF-----HV---AC-VDAWLRAH-------------------------------------------------------------ATCPSC  42
TraesCS7B02G480500  CAICLTEF-------------TDGES-----------VRVLPR--------------------------------------CG--HNF-----HV---AC-VDAWLRTC-------------------------------------------------------------ATCPSC  42
TraesCS7D02G554700  CAICLTEF-------------TDGES-----------VRVLPL--------------------------------------CG--HNF-----HV---AC-VDGWLRTC-------------------------------------------------------------ATCPSC  42
TraesCS7A02G555800  CAICLAEF-------------TDGES-----------VRVLPR--------------------------------------CG--HNF-----HM---TC-VDAWLRTC-------------------------------------------------------------ATCPSC  42
TraesCS7A02G556100  CAICLAEF-------------VEGEV-----------VRVLPR--------------------------------------CG--HTF-----HV---LC-IHAWLRTC-------------------------------------------------------------ATCPSC  42
TraesCS7B02G480600  CAICLAEF-------------ADGES-----------MRVLPR--------------------------------------CG--HAF-----HV---LC-IDAWLRTC-------------------------------------------------------------ATCPSC  42
TraesCS7A02G556200  CAICLADF-------------TEGEG-----------LRVLPR--------------------------------------CG--HHF-----HV---GC-VDAWLRTC-------------------------------------------------------------ATCPSC  42
TraesCS7B02G480700  CAICLADF-------------TEGEG-----------LRVLPR--------------------------------------CG--HHF-----HV---GC-VDAWLRTC-------------------------------------------------------------ATCPSC  42
TraesCS7A02G556400  CAICLAEY-------------MEGEA-----------LRVLPR--------------------------------------CG--HGF-----HV---AC-VDAWLRTC-------------------------------------------------------------ATCPSC  42
TraesCS7D02G554300  CAICLAEY-------------TEGEA-----------LRVLPR--------------------------------------CG--HRF-----HV---AC-VDAWLRTC-------------------------------------------------------------ATCPSC  42
TraesCS7A02G556500  CAICLAEF-------------TEGEA-----------LRVLPR--------------------------------------CG--HGF-----HV---AC-VDAWLRTC-------------------------------------------------------------ATCPSC  42
TraesCS7B02G480900  CAICLAEF-------------TEGEA-----------LRVLPR--------------------------------------CG--HGF-----HV---AC-VDAWLRTC-------------------------------------------------------------ATCPSC  42
TraesCS7D02G554400  CAICLAEF-------------TEGEG-----------LRVLPR--------------------------------------CG--HRF-----HV---AC-VDAWLRTC-------------------------------------------------------------ATCPSC  42
TraesCS7A02G556600  CGICLAEF-------------MEGEG-----------LRVLPQ--------------------------------------CG--HGF-----HV---AC-VDAWLRTC-------------------------------------------------------------ATCPSC  42
TraesCS7D02G554100  CAICLAEF-------------MEGEG-----------LRVLPR--------------------------------------CG--HGF-----HV---AC-VDAWLRTC-------------------------------------------------------------ATCPSC  42
TraesCS7B02G481000  CAICLAEF-------------TEGEG-----------LRVLPQ--------------------------------------CG--HGF-----HV---AC-VDAWLRSC-------------------------------------------------------------ATCPSC  42
TraesCS7D02G554500  CAICLAEF-------------AEGEG-----------VRVLPR--------------------------------------CC--HTF-----HV---VC-VDAWLRTC-------------------------------------------------------------ATCPSC  42
TraesCS7D02G554600  CAICLAEF-------------AEGEG-----------VRVLPR--------------------------------------CC--HTF-----HV---VC-VDAWLRTC-------------------------------------------------------------ATCPSC  42
TraesCS7D02G554200  CAICLAEF-------------AEGDG-----------LRVLPL--------------------------------------CS--HRF-----HV---AC-VDAWLRTC-------------------------------------------------------------ATCPSC  42
TraesCS7A02G556700  CAICLAEF-------------ADGEA-----------VRVLPG--------------------------------------CG--HGF-----HA---AC-VDAWLRTR-------------------------------------------------------------ATCPSC  42
TraesCS7D02G554000  CAICLAEF-------------ADGEA-----------VRVLPG--------------------------------------CG--HGF-----HI---AC-VDAWLRTR-------------------------------------------------------------ASCPSC  42
TraesCS7B02G481100  CAICLAEF-------------ADGEP-----------VRVLPG--------------------------------------CG--HGF-----HV---AC-VDAWLRTR-------------------------------------------------------------ATCPSC  42
TraesCS7A02G556000  CAICLAEF-------------TVGES-----------MRVLPR--------------------------------------CG--HAF-----HV---LC-IDTWLRTC-------------------------------------------------------------TSCPFC  42
TraesCS1A02G274500  CAICLAEF-------------ERGEQ-----------VRVLPR--------------------------------------CN--HGF-----HA---LC-IDRWLAAR-------------------------------------------------------------PTCPTC  42
TraesCS1D02G274500  CAICLAEF-------------VRGEQ-----------VRVLPR--------------------------------------CN--HGF-----HA---LC-IDRWLAAR-------------------------------------------------------------PTCPTC  42
TraesCS1B02G284000  CAICLAEF-------------IRGEK-----------VRVLPR--------------------------------------CN--HGF-----HA---AC-IDRWLAAR-------------------------------------------------------------PTCPTC  42
TraesCS2A02G299000  CTICLSDF-------------EAGEH-----------VKVLPK--------------------------------------CN--HGF-----HV---RC-IDRWLLAR-------------------------------------------------------------STCPTC  42
TraesCS2B02G315400  CTICLSDF-------------EAGEH-----------VKVLPK--------------------------------------CN--HGF-----HV---RC-IDRWLLAR-------------------------------------------------------------STCPTC  42
TraesCS2D02G296900  CTICLSDF-------------EAGEH-----------VKVLPK--------------------------------------CN--HGF-----HV---RC-IDRWLLAR-------------------------------------------------------------STCPTC  42
TraesCS3B02G409700  CAICLADL-------------KQGER-----------VRVLPR--------------------------------------CH--HGF-----HV---RC-IDRWLSAR-------------------------------------------------------------QTCPTC  42
TraesCS3D02G370200  CAICLADL-------------KQGER-----------VRVLPR--------------------------------------CH--HGF-----HV---RC-IDRWLSAR-------------------------------------------------------------QTCPTC  42
TraesCS3A02G377100  CAICLADL-------------KQGER-----------VRVLPR--------------------------------------CH--HGF-----HV---RC-IDRWLSAR-------------------------------------------------------------QTCPTC  42
TraesCS1A02G300400  CAICLGEF-------------VDGEK-----------VRVLPQ--------------------------------------CG--HGF-----HV---RC-VDAWLVSH-------------------------------------------------------------SSCPTC  42
TraesCS1B02G310500  CAICLGEF-------------VDGEK-----------VRVLPQ--------------------------------------CG--HGF-----HV---RC-VDAWLVSH-------------------------------------------------------------SSCPTC  42
TraesCS1A02G300500  CAICLGEF-------------VDGEK-----------VRVLPQ--------------------------------------CD--HGF-----HV---RC-VDAWLVSH-------------------------------------------------------------SSCPTC  42
TraesCS1D02G299800  CAICLGEF-------------VDGEK-----------VRVLPQ--------------------------------------CG--HGF-----HV---RC-VDAWLVSH-------------------------------------------------------------SACPTC  42
                    1.......10........20........30........40........50........60........70........80........90.......100.......110.......120.......130.......140.......150.......160.......170......
```

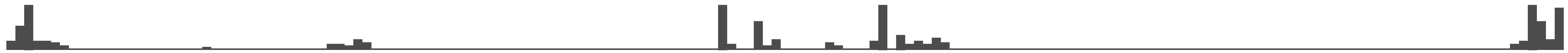

CLUSTAL 2.1 MULTIPLE SEQUENCE ALIGNMENT


```
                     *  *                                                                             *                 *                                                                       *
TraesCS1D02G299900   CAICLGEF-------------VDGEK-----------VRVLPE--------------------------------------CC--HGF-----HV---RC-VDAWLVSH------------------------------------------------------------SSCPTC   42
TraesCS1A02G300600   CAICLGEF-------------VDGEK-----------VRVLPR--------------------------------------CA--HGF-----HV---RC-VDAWLVSH------------------------------------------------------------GSCPTC   42
TraesCS1B02G310700   CAICLGEF-------------VDGEK-----------VRVLPR--------------------------------------CA--HGF-----HV---RC-VDAWLVSH------------------------------------------------------------GSCPTC   42
TraesCS1D02G300000   CAICLGEF-------------VDGEK-----------VRVLPR--------------------------------------CA--HGF-----HV---RC-VDAWLVSH------------------------------------------------------------GSCPTC   42
TraesCS5A02G308200   CAICLSEF-------------VDGEK-----------VRVLPQ--------------------------------------CA--HGF-----HV---RC-VDAWLVSN------------------------------------------------------------GSCPTC   42
TraesCS5B02G308500   CAICLSEF-------------VDGEK-----------VRVLPL--------------------------------------CG--HGF-----HV---RC-VDAWLVSH------------------------------------------------------------GSCPTC   42
TraesCS5B02G308600   CAICLSEF-------------VDGEK-----------VRVLPL--------------------------------------CG--HGF-----HV---RC-VDAWLVSH------------------------------------------------------------GSCPTC   42
TraesCS5B02G308700   CAICLSEF-------------VDGEK-----------VRVLPL--------------------------------------CG--HGF-----HV---RC-VDAWLVSH------------------------------------------------------------GSCPTC   42
TraesCS5D02G315300   CAICLSEF-------------VDGEK-----------VRVLPL--------------------------------------CR--HGF-----HV---RC-VDAWLVSH------------------------------------------------------------GSCPTC   42
TraesCS5D02G315200   CAICLSEF-------------VDGEK-----------VRVLPL--------------------------------------CG--HGF-----HV---RC-VDTWLVSH------------------------------------------------------------GSCPTC   42
TraesCS5A02G308300   CAICLSEF-------------VDGKK-----------VRVLPL--------------------------------------CG--HGF-----HV---RC-VNAWLVSH------------------------------------------------------------GSCTTC   42
TraesCS3A02G344600   CAICLGEF-------------EDGEK-----------VRVLPR--------------------------------------CG--HEF-----HV---RC-VDTWLVSH------------------------------------------------------------DSCPTC   42
TraesCS3B02G376500   CAICLGEF-------------EDGEK-----------VRVLPR--------------------------------------CG--HEF-----HV---RC-VDTWLVSH------------------------------------------------------------DSCPTC   42
TraesCS3D02G338100   CAICLGEF-------------EDGEK-----------VRVLPR--------------------------------------CG--HEF-----HV---RC-VDTWLVSH------------------------------------------------------------DSCPTC   42
TraesCS4A02G194200   CAICLGEF-------------ADGEK-----------VRVLPR--------------------------------------CH--HGF-----HV---RC-IDMWLATH------------------------------------------------------------TSCPNC   42
TraesCS4B02G121100   CAICLGEF-------------ADGEK-----------VRVLPR--------------------------------------CH--HGF-----HV---RC-IDMWLATH------------------------------------------------------------TSCPNC   42
TraesCS4D02G119100   CAICLGEF-------------ADGEK-----------VRVLPR--------------------------------------CH--HGF-----HV---RC-IDMWLATH------------------------------------------------------------TSCPNC   42
TraesCS6A02G239000   CAICLSEL-------------ADGER-----------VRVLPA--------------------------------------CG--HGF-----HG---AC-VDGWLAAR------------------------------------------------------------ASCPTC   42
TraesCS6D02G221300   CAICLSEL-------------ADGER-----------VRVLPA--------------------------------------CG--HGF-----HG---AC-VDGWLAAR------------------------------------------------------------ASCPTC   42
TraesCS7A02G478200   CAICLSDF-------------ADGET-----------IRVLPV--------------------------------------CG--HRF-----HV---LC-VDRWLTSR------------------------------------------------------------CSCPTC   42
TraesCS7D02G465600   CAICLSDF-------------KDGET-----------IRVLPV--------------------------------------CG--HRF-----HV---PC-VDRWLTSR------------------------------------------------------------CSCPTC   42
TraesCS7B02G380600   CAICLSDF-------------ADGET-----------IRVLPV--------------------------------------CG--HRF-----HV---PC-VDRWLASR------------------------------------------------------------CSCPTC   42
TraesCS1D02G230300   CAVCIVEF-------------RDGDL-----------ARLLPR--------------------------------------CG--HRF-----HA---AC-VDAWLHLH------------------------------------------------------------STCPLC   42
TraesCS1D02G230600   CAVCIVEF-------------RDGDL-----------ARLLPR--------------------------------------CG--HRF-----HA---AC-VDAWLHLH------------------------------------------------------------STCPLC   42
TraesCS1B02G242800   CAVCIVEF-------------RDGDL-----------ARLLPR--------------------------------------CG--HRF-----HA---AC-VDAWLHLH------------------------------------------------------------STCPLC   42
TraesCS1B02G242700   CAVCIVEF-------------RDGDL-----------ARLLPR--------------------------------------CG--HRF-----HA---AC-VDAWLHLH------------------------------------------------------------STCPLC   42
TraesCS1A02G231700   CAVCIVEF-------------RDGDL-----------ARLLPR--------------------------------------CG--HRF-----HA---AC-VDAWLHLH------------------------------------------------------------STCPLC   42
TraesCS1A02G228500   CAVCIVEF-------------RDGDL-----------ARLLPR--------------------------------------CG--HRF-----HA---AC-VDAWLHLH------------------------------------------------------------STCPLC   42
TraesCS4A02G137300   CAVCICEL-------------RDGDI-----------GRRLPR--------------------------------------CG--HRF-----HS---EC-VDRWFRSH------------------------------------------------------------ATCPLC   42
TraesCS4D02G170000   CAVCICEL-------------RDGDT-----------GRRLPR--------------------------------------CG--HRF-----HA---EC-VDRWFRSH------------------------------------------------------------ATCPLC   42
TraesCS2A02G167300   CAVCITEL-------------AAGET-----------ARVLPR--------------------------------------CG--HGF-----HV---DC-VDMWLKSH------------------------------------------------------------STCPLC   42
TraesCS2B02G193500   CAVCITEL-------------AAGET-----------ARVLPR--------------------------------------CG--HGF-----HV---DC-VDMWLKSH------------------------------------------------------------STCPLC   42
TraesCS2D02G174800   CAVCITEL-------------AAGET-----------ARVLPR--------------------------------------CG--HGF-----HV---DC-VDMWLKSH------------------------------------------------------------STCPLC   42
TraesCS5A02G396000   CAVCITEL-------------AAGEA-----------ARLLPR--------------------------------------CG--HSF-----HV---EC-VDMWLRSH------------------------------------------------------------STCPVC   42
TraesCS5B02G400800   CAVCITEL-------------AAGEA-----------ARLLPR--------------------------------------CG--HSF-----HV---EC-VDMWLRSH------------------------------------------------------------STCPLC   42
TraesCS5D02G405300   CAVCITEL-------------AAGDA-----------ARLLPR--------------------------------------CG--HSF-----HV---EC-VDMWLRSH------------------------------------------------------------STCPLC   42
TraesCS2B02G328600   CAVCLAEL-------------SDGEA-----------ARFLPK--------------------------------------CG--HGF-----HA---EC-VDLWLHSH------------------------------------------------------------PTCPLC   42
TraesCS2D02G310100   CAVCLAEL-------------SDGEA-----------ARFLPK--------------------------------------CG--HGF-----HA---EC-VDLWLHSH------------------------------------------------------------PTCPLC   42
TraesCS2A02G311800   CAVCLAEL-------------SEGEA-----------ARFLPK--------------------------------------CG--HGF-----HA---EC-VDLWLHSH------------------------------------------------------------PTCPLC   42
TraesCS3B02G023300   CAVCLAEL-------------ENGEE-----------ARFLPR--------------------------------------CG--HGF-----HA---GC-VNRWLASH------------------------------------------------------------TTCPLC   42
TraesCS3D02G024200   CAVCLAEL-------------EDGEE-----------ARFLPR--------------------------------------CG--HGF-----HA---GC-VDRWLAFH------------------------------------------------------------TTCPLC   42
TraesCS3A02G022400   CAVCLAGL-------------EDGEE-----------ARFLPR--------------------------------------CG--HGF-----HT---GC-VDRWLASH------------------------------------------------------------TTCPLC   42
TraesCS7B02G276000   CAVCLTEL-------------EDGEE-----------ARFLPR--------------------------------------CG--HGF-----HT---EC-VDMWLASH------------------------------------------------------------TSCPLC   42
TraesCS7D02G370800   CAVCLTEL-------------EDGEE-----------ARFLPR--------------------------------------CG--HGF-----HT---EC-VDMWLASH------------------------------------------------------------TSCPLC   42
TraesCS7A02G352100   CAVCLTEL-------------EDGEE-----------ARFLPR--------------------------------------CG--HGF-----HT---EC-VDMWLASH------------------------------------------------------------TSCPLC   42
TraesCS3B02G023000   CAVCLAGL-------------QDGEQ-----------ARFLPR--------------------------------------CG--HGF-----HA---GC-VDTWLASR------------------------------------------------------------STCPLC   42
TraesCS3B02G164600   CAVCLSEL-------------ADGDT-----------VRQLPN--------------------------------------CG--HVF-----HV---EC-VDVWLRTR------------------------------------------------------------TTCPLC   42
TraesCS3D02G147100   CAVCLSEL-------------ADGDT-----------VRQLPN--------------------------------------CG--HVF-----HV---EC-VDAWLRTR------------------------------------------------------------TTCPLC   42
TraesCS3A02G140000   CAVCLSEL-------------ADGDA-----------VRQLPN--------------------------------------CG--HVF-----HV---EC-VDAWLRTR------------------------------------------------------------TTCPLC   42
TraesCS3B02G164700   CAVCLSEL-------------ADGEK-----------VRELPN--------------------------------------CG--HSF-----HV---EC-VDAWLRSR------------------------------------------------------------TTCPLC   42
TraesCS3D02G147200   CAVCLSEL-------------ADGEK-----------VRELPN--------------------------------------CG--HSF-----HV---EC-VDAWLRSR------------------------------------------------------------TTCPLC   42
TraesCS5A02G470400   CAVCLAEL-------------ADGDE-----------ARELPN--------------------------------------CG--HLF-----HL---EC-VDAWLRTR------------------------------------------------------------TTCPLC   42
TraesCS5D02G483100   CAVCLAEL-------------ADGDE-----------ARELPN--------------------------------------CG--HLF-----HL---EC-VDAWLRTR------------------------------------------------------------TTCPLC   42
TraesCS5B02G483000   CAVCLAEL-------------ADGDQ-----------ARELPN--------------------------------------CG--HLF-----HL---EC-VDAWLRTR------------------------------------------------------------TTCPLC   42
TraesCS1D02G296300   CSICISAL-------------VAGDK-----------VKALPP--------------------------------------CG--HCF-----HP---EC-VDAWLRSQ------------------------------------------------------------PSCPLC   42
TraesCS4B02G162300   CSICISAL-------------VAGDK-----------VKALPP--------------------------------------CG--HCF-----HP---EC-VDAWLRSQ------------------------------------------------------------PSCPLC   42
TraesCS1A02G295900   CSICISAL-------------VAGDK-----------VKALPP--------------------------------------CG--HCF-----HP---EC-VDAWLRSQ------------------------------------------------------------PSCPLC   42
TraesCS1D02G296400   CSICISAL-------------VPGDK-----------VKALPP--------------------------------------CG--HCF-----HP---EC-VDAWLRSQ------------------------------------------------------------PSCPLC   42
TraesCS3A02G350500   CPICLGEF-------------ADGER-----------VKALPR--------------------------------------CG--HCF-----HP---EC-VDAWLCSH------------------------------------------------------------PSCPLC   42
TraesCS3D02G344500   CPICLGEF-------------ADGER-----------VKALPR--------------------------------------CG--HCF-----HP---EC-VDAWLCSH------------------------------------------------------------PSCPLC   42
TraesCS3B02G383200   CPICLGEF-------------VEGER-----------VKALPR--------------------------------------CG--HCF-----HP---EC-VDAWLCSH------------------------------------------------------------PSCPLC   42
TraesCS1A02G215100   CAVCQCVF-------------CADDE-----------LRLLPA--------------------------------------CR--HAF-----HS---GC-VDPWLRAN------------------------------------------------------------PSCPLC   42
TraesCS1D02G217700   CAVCQCVF-------------CADDE-----------LRLLPA--------------------------------------CR--HAF-----HS---GC-VDPWLRAN------------------------------------------------------------PSCPLC   42
TraesCS1B02G228600   CAVCQCVF-------------CGDDE-----------LRLLPA--------------------------------------CR--HAF-----HS---SC-VDPWLRSN------------------------------------------------------------PSCPLC   42
TraesCS2A02G453200   CAVCLSPF-------------RPEAE-----------LRLLPA--------------------------------------CR--HAF-----HA---VC-VDAWLRTT------------------------------------------------------------PTCPLC   42
TraesCS2B02G474500   CAVCLSPF-------------RPEAE-----------LRLLPA--------------------------------------CR--HAF-----HA---VC-VDAWLRTT------------------------------------------------------------PTCPLC   42
TraesCS2D02G452800   CAVCLSPF-------------RPEAE-----------LRLLPA--------------------------------------CR--HAF-----HA---VC-VDAWLRTT------------------------------------------------------------PTCPLC   42
TraesCS6B02G289700   CAVCLSPF-------------VPDAE-----------LRLLPA--------------------------------------CR--HAF-----HA---AC-VDAWLRNT------------------------------------------------------------PSCPLC   42
TraesCS6D02G243200   CAVCLSPF-------------VPDAE-----------LRLLPA--------------------------------------CR--HAF-----HA---AC-VDAWLRNT------------------------------------------------------------PSCPLC   42
TraesCS6A02G262500   CAVCLSPF-------------VPDAE-----------LRLLPA--------------------------------------CR--HAF-----HA---AC-VDAWLRNT------------------------------------------------------------PSCPLC   42
TraesCS1B02G479700   CAVCLARF-------------DDADL-----------LRLLPR--------------------------------------CR--HAF-----HL---DC-VDRWLHSS------------------------------------------------------------ASCPLC   42
TraesCS1D02G453000   CAVCLARF-------------DDADL-----------LRLLPR--------------------------------------CR--HAF-----HL---DC-VDRWLHSS------------------------------------------------------------ASCPLC   42
TraesCS1A02G444900   CAVCLARF-------------DDADL-----------LRLLPR--------------------------------------CR--HAF-----HL---DC-VDRWLHSS------------------------------------------------------------ASCPLC   42
TraesCS2A02G318200   CAVCLTAF-------------EEADD-----------LRLLPH--------------------------------------CS--HAF-----HP---EC-IDPWLEAR------------------------------------------------------------ITCPLC   42
TraesCS2B02G336300   CAVCLTAF-------------EEADD-----------LRLLPH--------------------------------------CS--HAF-----HP---EC-IDPWLEAR------------------------------------------------------------ITCPLC   42
                     1.......10........20........30........40........50........60........70........80........90.......100.......110.......120.......130.......140.......150.......160.......170.....
```

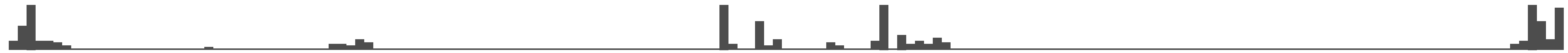

CLUSTAL 2.1 MULTIPLE SEQUENCE ALIGNMENT

File: C:/Users/HP/Downloads/clustal alignBB1.ps **Date: Wed May 20 03:00:39 2020**


```
                    *  *                                                                             *                 *                                                                        *
TraesCS2D02G315800  CAVCLTAF-------------EEADD-----------LRLLPH--------------------------------------CS--HAF-----HP---EC-IDPWLEAR-------------------------------------------------------------ITCPLC  42
TraesCS3A02G051000  CAVCLTEF-------------EDADD-----------LRLLPH--------------------------------------CS--HAF-----HP---DC-IDPWLQTR-------------------------------------------------------------TTCPLC  42
TraesCS3D02G050600  CAVCLTEF-------------EDADD-----------LRLLPH--------------------------------------CS--HAF-----HP---DC-IDPWLETR-------------------------------------------------------------TTCPLC  42
TraesCS3B02G060000  CAVCLTEF-------------EDDDD-----------MRLLPH--------------------------------------CS--HAF-----HP---EC-IDPWLESR-------------------------------------------------------------TTCPLC  42
TraesCS7A02G551400  CPVCLAAF-------------EEGDN-----------LRLLPH--------------------------------------CS--HVF-----HP---EC-VDPWLQAR-------------------------------------------------------------ATCPLC  42
TraesCS7D02G538000  CPVCLAVF-------------EEGDN-----------LRLLPH--------------------------------------CS--HVF-----HP---EC-IDPWLQAR-------------------------------------------------------------ATCPLC  42
TraesCS7B02G475300  CPVCLTAF-------------VEGDS-----------LRLLPH--------------------------------------CS--HVF-----HP---VC-IDPWLHQR-------------------------------------------------------------ATCPLC  42
TraesCS7D02G537300  CPVCLTAF-------------EEGDS-----------LRLLPH--------------------------------------CS--HVF-----HP---EC-IDPWLQRR-------------------------------------------------------------ATCPLC  42
TraesCS4A02G404600  CAVCLTEF-------------EDSDE-----------LRVLPA--------------------------------------CC--HVF-----HP---EC-IDPWLAGA-------------------------------------------------------------VTCPLC  42
TraesCS4B02G304400  CAVCLTEF-------------EDSDE-----------LRVLPA--------------------------------------CC--HVF-----HP---DC-IDPWLAGA-------------------------------------------------------------VTCPLC  42
TraesCS4D02G302600  CAVCLTEF-------------EDCDE-----------LRVLPA--------------------------------------CC--HVF-----HP---EC-IDPWLAGA-------------------------------------------------------------VTCPLC  42
TraesCS3B02G499500  CAVCLLEF-------------GDDDS-----------LRLLPA--------------------------------------CP--HAF-----HP---EC-IGLWLAKH-------------------------------------------------------------VTCPLC  42
TraesCS3D02G452500  CAVCLLEF-------------GDDDS-----------LRLLPA--------------------------------------CP--HAF-----HP---EC-ISLWLAKH-------------------------------------------------------------VTCPLC  42
TraesCS3A02G459700  CAVCLMEF-------------GDDDC-----------LRLLPA--------------------------------------CP--HAF-----HP---EC-IGLWLAKH-------------------------------------------------------------VTCPLC  42
TraesCS5B02G442500  CAVCLLEF-------------DDDDS-----------LRLLPT--------------------------------------CP--HAF-----HP---QC-IGLWLEKH-------------------------------------------------------------VTCPLC  42
TraesCS5D02G446700  CAVCLLEF-------------DDDDS-----------LRLLPT--------------------------------------CP--HAF-----HP---QC-IGLWLEKH-------------------------------------------------------------VTCPLC  42
TraesCS5A02G248100  CAVCISEF-------------DDDET-----------LRLLPK--------------------------------------CS--HVF-----HP---DC-IDTWLASH-------------------------------------------------------------VTCPVC  42
TraesCS5B02G245800  CAVCISEF-------------DDDET-----------LRLLPK--------------------------------------CS--HVF-----HP---DC-IDTWLASH-------------------------------------------------------------VTCPVC  42
TraesCS5D02G254900  CAVCLSEF-------------DDDET-----------LRLLPK--------------------------------------CS--HVF-----HP---DC-IDTWLASH-------------------------------------------------------------VTCPVC  42
TraesCS3D02G495500  CAVCLSEF-------------DDDET-----------LRLLPK--------------------------------------CS--HVF-----HP---DC-IDTWLASH-------------------------------------------------------------ITCPVC  42
TraesCS6A02G122800  CAVCLSEF-------------DDDDT-----------LRLLPK--------------------------------------CS--HAF-----HA---DC-IDAWLASH-------------------------------------------------------------VTCPVC  42
TraesCS6B02G151000  CAVCLSEF-------------DDDDT-----------LRLLPK--------------------------------------CS--HAF-----HA---DC-IDAWLASH-------------------------------------------------------------VTCPVC  42
TraesCS6D02G113000  CAVCLSEF-------------DDDDT-----------LRLLPK--------------------------------------CS--HAF-----HV---DC-IDAWLASH-------------------------------------------------------------VTCPVC  42
TraesCS6A02G315600  CAVCLSEF-------------EDEDR-----------LRLLPK--------------------------------------CS--HAF-----HP---DC-IGEWLASH-------------------------------------------------------------VTCPVC  42
TraesCS6D02G294800  CAVCLSEF-------------EDEDR-----------LRLLPK--------------------------------------CS--HAF-----HP---DC-IGEWLASH-------------------------------------------------------------VTCPVC  42
TraesCS6B02G346000  CAVCLSEF-------------EDEDR-----------LRLLPK--------------------------------------CS--HAF-----HP---EC-IGVWLASH-------------------------------------------------------------VTCPVC  42
TraesCS7A02G176700  CAVCLSEF-------------EDGDQ-----------LRLLPK--------------------------------------CS--HAF-----HP---DC-IGEWLAGH-------------------------------------------------------------VTCPVC  42
TraesCS7B02G081700  CAVCLSEF-------------EDGDQ-----------LRLLPK--------------------------------------CS--HAF-----HP---DC-IGEWLAGH-------------------------------------------------------------VTCPVC  42
TraesCS7D02G178000  CAVCLSEF-------------EDGDQ-----------LRLLPK--------------------------------------CS--HAF-----HP---DC-IGEWLAGH-------------------------------------------------------------VTCPVC  42
TraesCS4B02G191600  CAVCLCEF-------------ADDDR-----------LRLLPK--------------------------------------CS--HAF-----HV---EC-IDTWLLSH-------------------------------------------------------------STCPLC  42
TraesCS4D02G192800  CAVCLCEF-------------ADDDR-----------LRLLPK--------------------------------------CS--HAF-----HV---EC-IDTWLLSH-------------------------------------------------------------STCPLC  42
TraesCS4A02G112500  CAVCLCEF-------------ADDDR-----------LRLLPK--------------------------------------CS--HAF-----HV---EC-IDTWLLSH-------------------------------------------------------------STCPLC  42
TraesCS6A02G352200  CAVCLCEF-------------APDDQ-----------LRLLPK--------------------------------------CS--HAF-----HL---EC-IDTWLLSH-------------------------------------------------------------STCPLC  42
TraesCS6B02G385100  CAVCLCEF-------------APDDQ-----------LRLLPK--------------------------------------CS--HAF-----HL---EC-IDTWLLSH-------------------------------------------------------------STCPLC  42
TraesCS6D02G334900  CAVCLCEF-------------APDDQ-----------LRLLPK--------------------------------------CS--HAF-----HL---EC-IDTWLLSH-------------------------------------------------------------STCPLC  42
TraesCS7B02G056400  CAVCLCEF-------------AMDDA-----------LRLLPT--------------------------------------CG--HAF-----HV---PC-IDAWLLSH-------------------------------------------------------------STCPLC  42
TraesCS7D02G154300  CAVCLCEF-------------AMDDA-----------LRLLPT--------------------------------------CG--HAF-----HV---PC-IDAWLLSH-------------------------------------------------------------STCPLC  42
TraesCS7A02G152600  CAVCLCEF-------------AMDDA-----------LRLLPT--------------------------------------CG--HAF-----HV---PC-IDAWLLSH-------------------------------------------------------------STCPLC  42
TraesCS5A02G198500  CAVCLSEF-------------AADDR-----------LRLLPL--------------------------------------CG--HAF-----HV---AC-IDTWLRSS-------------------------------------------------------------STCPLC  42
TraesCS5D02G204500  CAVCLNEF-------------AADDR-----------LRLLPL--------------------------------------CG--HAF-----HV---AC-IDTWLRSS-------------------------------------------------------------STCPLC  42
TraesCS5B02G197200  CAVCLSEF-------------SPDDR-----------LRLLPL--------------------------------------CG--HAF-----HV---AC-IDTWLRSS-------------------------------------------------------------STCPLC  42
TraesCS7B02G029900  CAVCLCEF-------------AGDDR-----------LRLLPL--------------------------------------CG--HAF-----HI---DC-IDTWLLSN-------------------------------------------------------------STCPLC  42
TraesCS7D02G129000  CAVCLCEF-------------AGDDR-----------LRLLPL--------------------------------------CG--HAF-----HI---DC-IDTWLLSN-------------------------------------------------------------STCPLC  42
TraesCS7A02G129900  CAVCLCEF-------------AGDDR-----------LRLLPL--------------------------------------CG--HAF-----HI---DC-IDTWLLSN-------------------------------------------------------------STCPLC  42
TraesCS7A02G190800  CAVCLCEF-------------DAEDR-----------LRLLPL--------------------------------------CG--HAF-----HL---NC-IDTWLLSN-------------------------------------------------------------STCPLC  42
TraesCS7B02G095700  CAVCLCEF-------------DAEDR-----------LRLLPL--------------------------------------CG--HAF-----HL---NC-IDTWLLSN-------------------------------------------------------------STCPLC  42
TraesCS7D02G191800  CAVCLCEF-------------DAEDR-----------LRLLPL--------------------------------------CG--HAF-----HL---NC-IDTWLLSN-------------------------------------------------------------STCPLC  42
TraesCS2A02G318300  CSVCLGEF-------------NDGEL-----------VRLLPK--------------------------------------CG--HAF-----HV---PC-IDTWLRAH-------------------------------------------------------------VNCPLC  42
TraesCS2D02G315900  CSVCLGEF-------------NDGEL-----------VRLLPK--------------------------------------CG--HAF-----HV---PC-IDTWLRAH-------------------------------------------------------------VNCPLC  42
TraesCS2B02G336400  CSVCLGEF-------------SDGEL-----------VRLLPK--------------------------------------CG--HAF-----HV---PC-IDTWLRAH-------------------------------------------------------------VNCPLC  42
TraesCS7B02G371200  CSVCLGEF-------------LDGEL-----------LRLLPK--------------------------------------CG--HAF-----HV---PC-IGTWLRAH-------------------------------------------------------------VNCPLC  42
TraesCS7D02G457000  CSVCLGEF-------------LDGEL-----------LRLLPK--------------------------------------CG--HAF-----HV---PC-IGTWLRAH-------------------------------------------------------------VSCPLC  42
TraesCS2A02G448700  CSVCLGEF-------------HDGES-----------LRLLPK--------------------------------------CS--HAF-----HQ---QC-IDTWLKSH-------------------------------------------------------------SNCPLC  42
TraesCS2D02G447900  CSVCLGEF-------------HDGES-----------LRLLPK--------------------------------------CS--HAF-----HQ---QC-IDTWLKSH-------------------------------------------------------------SNCPLC  42
TraesCS2B02G469500  CSVCLGEF-------------DDGES-----------LRLLPK--------------------------------------CS--HAF-----HQ---QC-IDTWLKSH-------------------------------------------------------------SNCPLC  42
TraesCS6A02G264300  CSVCLGEF-------------RDGES-----------LRLLPK--------------------------------------CS--HAF-----HL---LC-IDTWLKSH-------------------------------------------------------------SNCPLC  42
TraesCS6D02G250600  CSVCLGEF-------------RDGES-----------LRLLPK--------------------------------------CS--HAF-----HL---PC-IDTWLKSH-------------------------------------------------------------SNCPLC  42
TraesCS6B02G291300  CSVCLGEF-------------QDGES-----------LRLLPK--------------------------------------CS--HAF-----HL---PC-IDTWLKSH-------------------------------------------------------------SNCPLC  42
TraesCS5B02G246000  CAVCLGEF-------------SDGEL-----------VRLLPR--------------------------------------CS--HPF-----HA---PC-IDTWLRAH-------------------------------------------------------------VSCPIC  42
TraesCS5D02G255200  CAVCLGEF-------------SDGEL-----------VRLLPR--------------------------------------CS--HPF-----HA---PC-IDTWLRAH-------------------------------------------------------------VSCPIC  42
TraesCS5A02G248300  CAVCLGEF-------------SDGEL-----------VRLLPR--------------------------------------CS--HPF-----HA---PC-IDTWLRAH-------------------------------------------------------------VSCPIC  42
TraesCS6A02G050800  CAVCLAEF-------------RGGET-----------LRLLPR--------------------------------------CA--HAF-----HR---GC-IDTWLRAH-------------------------------------------------------------VNCPLC  42
TraesCSU02G109900   CAVCLAEF-------------RGGET-----------LRLLPR--------------------------------------CA--HAF-----HR---GC-IDTWLRAH-------------------------------------------------------------VNCPLC  42
TraesCS6B02G067200  CAVCLAEF-------------RGGET-----------LRLLPR--------------------------------------CG--HAF-----HR---GC-IDTWLRAH-------------------------------------------------------------VNCPLC  42
TraesCS5A02G062400  CAVCLLEF-------------ADGDE-----------LRALPL--------------------------------------CA--HAF-----HA---DC-IDVWLRAH-------------------------------------------------------------ASCPLC  42
TraesCS5B02G069700  CAVCLLEF-------------ADGDE-----------LRALPL--------------------------------------CA--HAF-----HA---DC-IDVWLRAH-------------------------------------------------------------ASCPLC  42
TraesCS5D02G073600  CAVCLLEF-------------ADGDE-----------LRALPL--------------------------------------CA--HAF-----HA---DC-IDVWLRAH-------------------------------------------------------------ASCPLC  42
TraesCS4A02G415800  CAVCLSEF-------------AERER-----------VRLLPG--------------------------------------CS--HAF-----HI---DC-IDTWLQGS-------------------------------------------------------------ARCPFC  42
TraesCS4B02G316600  CAVCLSEF-------------AERER-----------VRLLPG--------------------------------------CS--HAF-----HI---DC-IDTWLQGS-------------------------------------------------------------ARCPFC  42
TraesCS4D02G313200  CAVCLSDF-------------AERER-----------VRLLPG--------------------------------------CS--HAF-----HI---DC-IDTWLQGS-------------------------------------------------------------ARCPFC  42
TraesCS6B02G437700  CAVCLGEF-------------QEGER-----------VRLLPG--------------------------------------CL--HVF-----HA---EC-IDTWLHGC-------------------------------------------------------------ASCPLC  42
TraesCS6D02G382200  CAVCLGEF-------------QEGER-----------VRLLPG--------------------------------------CL--HVF-----HA---EC-IDTWLHGC-------------------------------------------------------------ASCPLC  42
TraesCS6A02G397900  CAVCLGEF-------------QEGER-----------VRLLPG--------------------------------------CL--HVF-----HA---EC-IDTWLHDC-------------------------------------------------------------ANCPLC  42
TraesCS6D02G382100  CAVCLGEF-------------QEGES-----------VRLLPG--------------------------------------CL--HVF-----HA---EC-IGTWLQGC-------------------------------------------------------------ANCPLC  42
TraesCS7A02G253700  CAVCLSEF-------------QEGER-----------LRLLPP--------------------------------------CL--HLF-----HI---DC-IDAWLHAT-------------------------------------------------------------ANCPLC  42
                    1.......10........20........30........40........50........60........70........80........90.......100.......110.......120.......130.......140.......150.......160.......170......
```

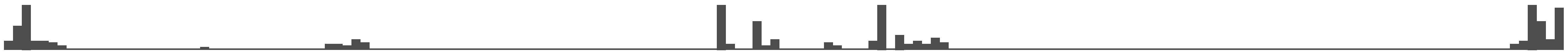

```
                   *  *                                                                             *                 *                                                                        *
TraesCS7D02G252300 CAVCLSEF-------------QEGER-----------LRLLPP--------------------------------------CL--HLF-----HI---DC-IDAWLHAT-------------------------------------------------------------ANCPLC   42
TraesCS7B02G150000 CAVCLSEF-------------REGER-----------LRLLPP--------------------------------------CL--HLF-----HI---DC-IDAWLHAT-------------------------------------------------------------ANCPLC   42
TraesCS4A02G415900 CAVCLSEF-------------APGER-----------LKMLPA--------------------------------------CS--HAF-----HI---DC-IDTWLHHN-------------------------------------------------------------VSCPLC   42
TraesCS4D02G313300 CAVCLSEF-------------APGER-----------LKMLPA--------------------------------------CS--HAF-----HI---DC-IDTWLHHN-------------------------------------------------------------VSCPLC   42
TraesCS4B02G316700 CAVCLSEF-------------EPGER-----------LKMLPA--------------------------------------CS--HAF-----HI---DC-IDTWLHHN-------------------------------------------------------------VSCPLC   42
TraesCS1B02G128900 CAVCLGEM-------------EDGEL-----------GRLLPA--------------------------------------CS--HAF-----HV---GC-IDAWLRLS-------------------------------------------------------------STCPVC   42
TraesCS1D02G112400 CAVCLGEM-------------EDGEL-----------GRMLPA--------------------------------------CS--HAF-----HV---GC-IDAWLRLS-------------------------------------------------------------STCPVC   42
TraesCS3A02G087900 CAVCLAEF-------------ERGEE-----------ARALPR--------------------------------------CG--HRF-----HV---EC-IDAWFRGN-------------------------------------------------------------STCPLC   42
TraesCS3B02G103300 CAVCLAEF-------------ERGEE-----------ARALPR--------------------------------------CG--HRF-----HV---EC-IDAWFRGN-------------------------------------------------------------STCPLC   42
TraesCS3D02G088100 CAVCLAEF-------------ERGEE-----------ARALPR--------------------------------------CG--HRF-----HV---EC-IDAWFRGN-------------------------------------------------------------STCPLC   42
TraesCS3D02G088200 CAVCLAEF-------------ERGEE-----------ARALPR--------------------------------------CG--HRF-----HV---EC-IDAWFRGN-------------------------------------------------------------STCPLC   42
TraesCS6A02G055100 CAVCLAGL-------------ERGEE-----------ARALPR--------------------------------------CG--HRF-----HV---GC-IDAWFRGN-------------------------------------------------------------STCPLC   42
TraesCSU02G121400  CAVCLAGL-------------ERGEE-----------ARALPR--------------------------------------CG--HRF-----HV---GC-IDAWFRGN-------------------------------------------------------------STCPLC   42
TraesCS4B02G167400 CAVCLAEM-------------TDGER-----------GRLLPR--------------------------------------CG--HRF-----HV---EC-IDRWFRSN-------------------------------------------------------------STCPLC   42
TraesCS4D02G169900 CAVCLAEM-------------TDGER-----------GRLLPR--------------------------------------CG--HRF-----HV---EC-IDRWFRSN-------------------------------------------------------------STCPLC   42
TraesCS4A02G137800 CAVCLAEM-------------TDGER-----------GRLLPR--------------------------------------CG--HRF-----HV---EC-IDRWFRSN-------------------------------------------------------------SSCPLC   42
TraesCS2A02G109000 CAVCLSEF-------------GEREA-----------GRLLPG--------------------------------------CG--HGF-----HE---AC-IAAWLRLS-------------------------------------------------------------STCPLC   42
TraesCS2D02G109100 CAVCLSEF-------------GEREA-----------GRRLPG--------------------------------------CG--HGF-----HE---AC-IAAWLRLS-------------------------------------------------------------STCPLC   42
TraesCS2B02G126700 CAVCLSEF-------------AEREA-----------GRRLPG--------------------------------------CG--HGF-----HE---AC-IAAWLRLN-------------------------------------------------------------STCPLC   42
TraesCS1D02G010300 CAVCLERY-------------ADSDV-----------VRVLPD--------------------------------------CG--HLF-----HR---GC-VDPWLRQR-------------------------------------------------------------PTCPVC   42
TraesCS7A02G503800 CAVCLERY-------------ADSDV-----------VRVLPD--------------------------------------CG--HLF-----HR---GC-VDAWLRRR-------------------------------------------------------------PTCPVC   42
TraesCS5A02G426200 CSVCLDNY-------------GDGDV-----------LRMLPD--------------------------------------CG--HLF-----HR---EC-VDPWLRKH-------------------------------------------------------------PTCPVC   42
TraesCS5B02G428100 CSVCLDNY-------------GDGDV-----------LRMLPD--------------------------------------CG--HLF-----HR---EC-VDPWLRKH-------------------------------------------------------------PTCPVC   42
TraesCS5D02G434200 CSVCLDNY-------------GDGDV-----------LRMLPD--------------------------------------CG--HLF-----HR---EC-VDPWLRKH-------------------------------------------------------------PTCPVC   42
TraesCS5B02G026700 CAVCLGEY-------------DRGDV-----------LRVLPD--------------------------------------CA--HSF-----HR---PC-VDQWLRLR-------------------------------------------------------------PSCPVC   42
TraesCS5D02G035300 CAVCLGEY-------------DRGDV-----------LRVLPD--------------------------------------CA--HSF-----HR---PC-VDQWLRLR-------------------------------------------------------------PSCPVC   42
TraesCS5A02G027500 CAVCLGEY-------------DRGDV-----------LRVLPD--------------------------------------CA--HSF-----HR---PC-VDQWLRLR-------------------------------------------------------------PSCPVC   42
TraesCS5A02G028300 CAVCLAQY-------------ADGDE-----------LRWLPG--------------------------------------CR--HAF-----HR---PC-VDEWLRRR-------------------------------------------------------------PSCPLC   42
TraesCS5A02G028400 CAVCLAQY-------------ADGDE-----------LRWLPG--------------------------------------CR--HTF-----HR---LC-VDEWLRRR-------------------------------------------------------------PSCPLC   42
TraesCS4B02G270600 CSVCLAEY-------------ADGDE-----------LRWLPG--------------------------------------CR--HAF-----HR---SC-VDEWLRRR-------------------------------------------------------------PSCPLC   42
TraesCS5A02G028000 CAVCLAEY-------------ADGDE-----------LRRLPG--------------------------------------CR--HAF-----HR---RC-VDEWLRRR-------------------------------------------------------------PSCPLC   42
TraesCS5A02G028700 CAVCLAEY-------------ADGDE-----------LRRLPG--------------------------------------CR--HAF-----HR---RC-VDEWLRRR-------------------------------------------------------------PSCPLC   42
TraesCS5D02G035700 CSVCLAEY-------------ADGDE-----------LRRLPG--------------------------------------CR--HAF-----HR---RC-VDEWLRRR-------------------------------------------------------------PSCPLC   42
TraesCSU02G211400  CAVCLAEY-------------ADGDE-----------LRRLPG--------------------------------------CQ--HAF-----HR---RC-VDEWLRRR-------------------------------------------------------------PSCPLC   42
TraesCS5D02G036500 CSVCLGEY-------------ANGDE-----------LRRLPG--------------------------------------CR--HAF-----HR---RC-VDEWLRRR-------------------------------------------------------------PSCPLC   42
TraesCS5A02G028500 CAVCLAEY-------------ADGDE-----------LRRLPG--------------------------------------CK--HAF-----HR---LC-VDEWLRRR-------------------------------------------------------------PSCPLC   42
TraesCS5D02G035900 CAVCLAEY-------------ADGDE-----------LRRLPG--------------------------------------CK--HAF-----HR---LC-VDEWLRRR-------------------------------------------------------------PSCPLC   42
TraesCSU02G230000  CVVCLAEY-------------ADGDE-----------LRRLPG--------------------------------------CK--HAF-----HR---LC-VDEWLRRR-------------------------------------------------------------PSCPLC   42
TraesCS5D02G036300 CAVCLAEY-------------ADGDE-----------LRWLPG--------------------------------------CK--HAF-----HR---LC-VDEWLRRR-------------------------------------------------------------PSCPLC   42
TraesCSU02G200100  CAVCLAEY-------------ADADE-----------LRRLPG--------------------------------------CK--HVF-----HR---LC-VDEWLRRR-------------------------------------------------------------PSCPLC   42
TraesCSU02G195400  CAVCLAEY-------------ADGDE-----------LRRLPG--------------------------------------CR--HSF-----HR---RC-VHDWLRRR-------------------------------------------------------------PSCPLC   42
TraesCSU02G220900  CAVCLAEY-------------ADGDE-----------LRRLPG--------------------------------------CR--HSF-----HR---RC-VHDWLRRR-------------------------------------------------------------PSCPLC   42
TraesCSU02G176500  CAVCLAEY-------------ADGDE-----------LRVLPG--------------------------------------CA--HAF-----HR---RC-VDQWLRHR-------------------------------------------------------------PSCPLC   42
TraesCSU02G176600  CAVCLAEY-------------ADGDE-----------LRVLPG--------------------------------------CA--HAF-----HR---RC-VDQWLRHR-------------------------------------------------------------PSCPLC   42
TraesCS5A02G028200 CAVCLAEY-------------ADGDE-----------LRVLPG--------------------------------------CA--HAF-----HR---RC-VDQWLRQR-------------------------------------------------------------PSCPLC   42
TraesCS5A02G028600 CAVCLAEY-------------ADGDE-----------LRVLPG--------------------------------------CA--HAF-----HR---RC-VDQWLRRR-------------------------------------------------------------PSCPLC   42
TraesCS5D02G035800 CAVCLAEY-------------ADGDE-----------LRVLPG--------------------------------------CA--HVF-----HR---RC-VDQWLRRR-------------------------------------------------------------PSCPLC   42
TraesCS5D02G036400 CAVCLAEY-------------ADGDE-----------LRVMPG--------------------------------------CA--HTF-----HR---RC-VDQWLRRR-------------------------------------------------------------PSCPLC   42
TraesCS5D02G036200 CAVCLAEY-------------ADGDE-----------LRVLPG--------------------------------------CA--HAF-----HR---RC-VDQWLRQR-------------------------------------------------------------SSCPLC   42
TraesCS5A02G029200 CAVCLADY-------------ADGDE-----------LRRLPG--------------------------------------CR--HVF-----HQ---RC-VDDWLRQR-------------------------------------------------------------TSCPLC   42
TraesCS4A02G034600 CAVCLTEY-------------ADGDD-----------LRWLPR--------------------------------------CR--HAF-----HR---SC-VDEWLRRR-------------------------------------------------------------PSCPLC   42
TraesCS4A02G183700 CVVCLAEY-------------EDDDV-----------LRVLPY--------------------------------------CG--HDF-----HV---AC-IDIWLKQH-------------------------------------------------------------STCPVC   42
TraesCS4D02G129700 CVVCLAEY-------------EDDDV-----------LRVLPY--------------------------------------CG--HDF-----HV---AC-IDIWLKQH-------------------------------------------------------------STCPVC   42
TraesCS4B02G134900 CVVCLAEY-------------EDDDV-----------LRVLPY--------------------------------------CG--HDF-----HV---AC-TDIWLKQH-------------------------------------------------------------STCPVC   42
TraesCS5B02G129800 CVVCLAEY-------------EDKDV-----------LRTLPY--------------------------------------CG--HNF-----HM---VC-IDAWLKQH-------------------------------------------------------------STCPVC   42
TraesCS5D02G137400 CVVCLAEY-------------EDKDV-----------LRTLPY--------------------------------------CG--HNF-----HM---VC-IDAWLKQH-------------------------------------------------------------STCPVC   42
TraesCS5A02G129900 CVVCLAEY-------------EDKDV-----------LRTLPY--------------------------------------CG--HNF-----HM---VC-IDAWLKQH-------------------------------------------------------------STCPVC   42
TraesCS4A02G338900 CTVCLEEY-------------EAKDV-----------VRVLPY--------------------------------------CG--HAF-----HA---AC-IDTWLRQH-------------------------------------------------------------PTCPIC   42
TraesCS5B02G548200 CTVCLEEY-------------EAKDV-----------VRVLPY--------------------------------------CG--HAF-----HA---AC-IDTWLRQH-------------------------------------------------------------PTCPIC   42
TraesCS5D02G535300 CTVCLEEY-------------EAKDV-----------VRVLPY--------------------------------------CG--HAF-----HA---AC-IDTWLRQH-------------------------------------------------------------PTCPIC   42
TraesCS7B02G234500 CVVCLQEL-------------EDGDV-----------VRVLPA--------------------------------------CR--HFF-----HG---SC-IDTWLRAH-------------------------------------------------------------SSCPVC   42
TraesCS7D02G330800 CVVCLQEL-------------EDGDV-----------VRVLPA--------------------------------------CR--HFF-----HG---SC-IDTWLRAH-------------------------------------------------------------SSCPVC   42
TraesCS7A02G348200 CVVCLQEL-------------EDGDV-----------VRVLPA--------------------------------------CR--HFF-----HG---SC-IDTWLRAH-------------------------------------------------------------SSCPVC   42
TraesCS2B02G499500 CAVCLGAM-------------QDGDA-----------VRALPG--------------------------------------CS--HAF-----HA---GC-VDVWLGAH-------------------------------------------------------------ATCPVC   42
TraesCS2D02G475900 CAVCLGAM-------------QDGDA-----------VRALPG--------------------------------------CS--HAF-----HA---GC-VDVWLGAH-------------------------------------------------------------ATCPVC   42
TraesCS2A02G475800 CAVCLGAM-------------QDGDA-----------VRALPG--------------------------------------CS--HAF-----HA---GC-VDVWLGAH-------------------------------------------------------------ATCPVC   42
TraesCS4A02G220900 CAVCLSAM-------------QDGDA-----------VRALPG--------------------------------------CR--HAF-----HV---GC-VDVWLCAQ-------------------------------------------------------------ATCPVC   42
TraesCS4D02G095800 CAVCLSAM-------------QDGDA-----------VRALPG--------------------------------------CR--HAF-----HV---GC-VDVWLCAR-------------------------------------------------------------ATCPVC   42
TraesCS4B02G099300 CAVCLSAM-------------QDGDA-----------VRALPG--------------------------------------CR--HAF-----HV---GC-VDVWLCAH-------------------------------------------------------------ATCPVC   42
TraesCS1A02G192600 CAVCLSEF-------------APGDA-----------VRPLTV--------------------------------------CR--HAF-----HA---AC-IDSWLGAH-------------------------------------------------------------TTCPVC   42
TraesCS3B02G165000 CPVCLGAV-------------EEGET-----------VRALPV--------------------------------------CR--HAF-----HA---RC-VDAWLRLR-------------------------------------------------------------PTCPVC   42
TraesCS3D02G147700 CPVCLGAV-------------EEGET-----------VRALPV--------------------------------------CR--HAF-----HA---RC-VDAWLRLR-------------------------------------------------------------PTCPVC   42
TraesCS3A02G131400 CPVCLGAV-------------EEGET-----------MRALPV--------------------------------------CR--HAF-----HA---RC-VDAWLRLR-------------------------------------------------------------PTCPVC   42
TraesCS6B02G178500 CPVCLDAA-------------RAGER-----------WRALPA--------------------------------------CG--HAF-----HA---AC-VDRWLLLS-------------------------------------------------------------PGCPVC   42
TraesCS6D02G140100 CPVCLDAA-------------RAGER-----------WRALPA--------------------------------------CG--HAF-----HA---AC-VDRWLLLS-------------------------------------------------------------PGCPVC   42
                   1.......10........20........30........40........50........60........70........80........90........100.......110.......120.......130.......140.......150.......160.......170......
```

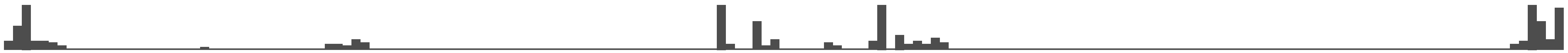

CLUSTAL 2.1 MULTIPLE SEQUENCE ALIGNMENT

File: C:/Users/HP/Downloads/clustal alignBB1.ps **Date: Wed May 20 03:00:39 2020**


```
                    *  *                                                                             *                 *                                                                        *
TraesCS6A02G290900  CAVCLEAF-------------QGGDR-----------CRVLPG--------------------------------------CH--HGF-----HA---QC-VDSWLRQS-------------------------------------------------------------RRCPVC  42
TraesCS6D02G272200  CAVCLEAF-------------QGGDR-----------CRVLPG--------------------------------------CH--HGF-----HA---QC-VDSWLRQS-------------------------------------------------------------RRCPVC  42
TraesCS6B02G321500  CAVCLEAF-------------QGGDR-----------CRVLPG--------------------------------------CH--HGF-----HA---QC-VDSWLRQS-------------------------------------------------------------RWCPVC  42
TraesCS6A02G291000  CAVCLEAF-------------MAGDR-----------CMVLPR--------------------------------------CE--HGF-----HA---EC-VSSWLRKS-------------------------------------------------------------RLCPIC  42
TraesCS6D02G272300  CAVCLEAF-------------NAGER-----------CMVLPR--------------------------------------CE--HGF-----HA---EC-VGSWLRKS-------------------------------------------------------------RLCPIC  42
TraesCS7A02G210400  CAVCLEAF-------------ESGDR-----------CRRLPR--------------------------------------CE--HSF-----HA---PC-VDSWLKKS-------------------------------------------------------------QCCPVC  42
TraesCS7B02G117100  CAVCLEAF-------------ESGDR-----------CRRLPR--------------------------------------CE--HSF-----HA---PC-VDSWLKKS-------------------------------------------------------------QCCPVC  42
TraesCS7D02G212200  CAVCLEAF-------------ESGDR-----------CRRLPR--------------------------------------CE--HSF-----HA---PC-VDSWLKKS-------------------------------------------------------------QCCPVC  42
TraesCS1A02G126200  CAVCLAVM-------------VDGEA-----------ARRLPR--------------------------------------CM--HVF-----HR---SC-VDVWLREH-------------------------------------------------------------STCPVC  42
TraesCS1D02G127300  CAVCLAVM-------------VDGEA-----------ARRLPR--------------------------------------CM--HVF-----HR---SC-VDVWLREH-------------------------------------------------------------STCPVC  42
TraesCS1B02G145400  CAVCLAVM-------------VDGQA-----------ARRLPR--------------------------------------CM--HVF-----HR---SC-VDVWLREH-------------------------------------------------------------STCPVC  42
TraesCS3B02G164900  CSICLCTM-------------ADGEV-----------ARQLPR--------------------------------------CM--HLF-----HR---AC-VDMWLTAH-------------------------------------------------------------TTCPVC  42
TraesCS3D02G147600  CSICLCTM-------------ADGEV-----------ARQLPR--------------------------------------CT--HLF-----HR---AC-VDMWLTAH-------------------------------------------------------------TTCPVC  42
TraesCS2B02G019500  CTICLQDM-------------VAGET-----------ARRLPN--------------------------------------CS--HAF-----HQ---PC-VDKWLFTH-------------------------------------------------------------GSCPVC  42
TraesCS2D02G005100  CTICLQDM-------------VAGET-----------ARRLPN--------------------------------------CS--HAF-----HQ---PC-VDKWLFTH-------------------------------------------------------------GSCPVC  42
TraesCSU02G011000   CIICLQDM-------------VAGET-----------ARRLPN--------------------------------------CS--HTF-----HQ---PC-VDKWLFTH-------------------------------------------------------------GSCPVC  42
TraesCS6B02G336900  CAICLQDM-------------VAGET-----------ARRLPT--------------------------------------CS--HAF-----HQ---LC-VDKWLISH-------------------------------------------------------------GSCPVC  42
TraesCS6D02G287400  CAICLQDM-------------VAGEM-----------ARRLPT--------------------------------------CS--HAF-----HQ---LC-VDKWLISH-------------------------------------------------------------GSCPVC  42
TraesCS6A02G308200  CAICLQDM-------------VGGET-----------ARRLPN--------------------------------------CS--HAF-----HQ---LC-VDKWLTSH-------------------------------------------------------------GSCPVC  42
TraesCS7B02G094900  CPICLQDI-------------VTGEM-----------ARRLPK--------------------------------------CC--HTF-----HQ---PC-VDKWLIGH-------------------------------------------------------------GSCPVC  42
TraesCS7D02G191100  CPICLQDI-------------VTGEM-----------ARRLPK--------------------------------------CC--HTF-----HQ---PC-VDKWLIGH-------------------------------------------------------------GSCPVC  42
TraesCS7A02G189900  CPICLQDI-------------MTGEM-----------ARRLPK--------------------------------------CC--HTF-----HQ---PC-VDKWLIGH-------------------------------------------------------------GSCPVC  42
TraesCS2A02G501100  CAVCLGSFDA-----------AADEL-----------LRVLPK--------------------------------------CR--HAF-----HA---GC-VDTWLEAH-------------------------------------------------------------STCPVC  44
TraesCS2D02G501500  CAVCLGAFDA-----------AADEL-----------LRVLPK--------------------------------------CR--HAF-----HA---GC-VDTWLEAH-------------------------------------------------------------STCPVC  44
TraesCS2B02G529100  CAVCLGAFDA-----------AADEL-----------LRVLPK--------------------------------------CR--HAF-----HA---DC-VDTWLEAH-------------------------------------------------------------STCPVC  44
TraesCS4A02G104900  CAVCLAEF-A-----------DDGEK-----------LRLLPG--------------------------------------CC--HVF-----HA---AC-IDVWLAAH-------------------------------------------------------------VTCPVC  43
TraesCS4B02G199100  CAVCLAEF-A-----------DDGEK-----------LRLLPG--------------------------------------CC--HVF-----HA---AC-IDVWLAAH-------------------------------------------------------------VTCPVC  43
TraesCS4D02G200100  CAVCLAEF-A-----------DDGEK-----------LRLLPG--------------------------------------CR--HVF-----HA---AC-IDVWLAAH-------------------------------------------------------------VTCPVC  43
TraesCS3A02G131500  CAVCLGQL-------------EAGEK-----------ARRLPK--------------------------------------CA--HLF-----HA---EC-VDAWLRAH-------------------------------------------------------------CTCPMC  42
TraesCS3B02G165100  CAVCLGQL-------------EAGEK-----------ARRLPK--------------------------------------CA--HLF-----HA---EC-VDAWLRAH-------------------------------------------------------------CTCPMC  42
TraesCS3D02G147800  CAVCLGQL-------------EAGEK-----------ARRLPK--------------------------------------CA--HLF-----HA---EC-VDAWLRAH-------------------------------------------------------------CTCPMC  42
TraesCS7A02G023300  CAVCLGQV-------------EAGEK-----------LRRLPK--------------------------------------CA--HLF-----HA---DC-VHAWLQAH-------------------------------------------------------------STCPMC  42
TraesCS7A02G458100  CAVCLGQV-------------EAGEK-----------LRRLPK--------------------------------------CA--HLF-----HA---DC-VHAWLQAH-------------------------------------------------------------STCPMC  42
TraesCS7D02G021000  CAVCLGQV-------------EAGEK-----------LRRLPK--------------------------------------CA--HLF-----HA---DC-VHAWLQAH-------------------------------------------------------------STCPMC  42
TraesCS7D02G020500  CAVCLGQV-------------EAGEK-----------LRRLPK--------------------------------------CA--HLF-----HA---DC-VHAWLRAH-------------------------------------------------------------STCPMC  42
TraesCS7D02G420000  CAVCLGRF-------------EPTEA-----------LRLLPK--------------------------------------CR--HGF-----HV---EC-VDTWLDAH-------------------------------------------------------------STCPLC  42
TraesCSU02G093700   CAVCLGRF-------------EPTEA-----------LRLLPK--------------------------------------CR--HGF-----HV---EC-VDTWLDAH-------------------------------------------------------------STCPLC  42
TraesCS7B02G327800  CAVCLGRF-------------EPTEA-----------LRLLPK--------------------------------------CR--HGF-----HV---EC-VDTWLDAH-------------------------------------------------------------STCPLC  42
TraesCS2A02G156600  CAVCLSLV-------------KEGEV-----------VRQLPA--------------------------------------CM--HLF-----HV---GC-VDTWLHSH-------------------------------------------------------------STCPLC  42
TraesCS2B02G182100  CAVCLSLV-------------KEGEV-----------VRQLPA--------------------------------------CM--HLF-----HV---GC-VDTWLHSH-------------------------------------------------------------STCPLC  42
TraesCS2D02G162400  CAVCLSLV-------------KEGEV-----------VRQLPA--------------------------------------CM--HLF-----HV---GC-VDTWLHSH-------------------------------------------------------------STCPLC  42
TraesCS7D02G141500  CVICLGLV-------------QVGEM-----------VRQLPA--------------------------------------CK--HMF-----HV---DC-VDVWLRSH-------------------------------------------------------------STCPIC  42
TraesCS3B02G328200  CTICLGAM-------------QEGEA-----------VRVLPA--------------------------------------CA--HVF-----HV---PC-VDTWLASS-------------------------------------------------------------SSCPVC  42
TraesCS3D02G293300  CTICLGAM-------------QEGEA-----------VRVLPA--------------------------------------CA--HVF-----HV---PC-VDTWLASS-------------------------------------------------------------SSCPVC  42
TraesCS3A02G293500  CTICLGAM-------------QEGEA-----------VRVLPA--------------------------------------CA--HVF-----HV---PC-VDTWLASS-------------------------------------------------------------SSCPVC  42
TraesCS3A02G293400  CTICLGAM-------------QEGEA-----------VRVLPA--------------------------------------CA--HVF-----HV---PC-VDTWLASS-------------------------------------------------------------SSCPVC  42
TraesCS3D02G293200  CTICLSAM-------------QEGEA-----------VRVLPA--------------------------------------CA--HVF-----HV---PC-VDTWLASS-------------------------------------------------------------SSCPVC  42
TraesCS3B02G164800  CAVCLSAV-------------DEGET-----------VRQLPS--------------------------------------CK--HLF-----HQ---EC-VDVWLLSN-------------------------------------------------------------ASCPVC  42
TraesCS3D02G147400  CAVCLSAV-------------DEGET-----------VRQLPS--------------------------------------CK--HLF-----HQ---EC-VDVWLLSN-------------------------------------------------------------ASCPVC  42
TraesCS3A02G139700  CAVCLRVV-------------DEGET-----------VRQLPS--------------------------------------CK--HLF-----HQ---EC-VDVWLLSN-------------------------------------------------------------ASCPVC  42
TraesCS3D02G147500  CAVCLGAL-------------DERER-----------VRQLPA--------------------------------------CN--HLF-----HQ---GC-VDVWLMSN-------------------------------------------------------------ASCPVC  42
TraesCS7B02G234600  CAVCLEEL-------------REGAL-----------VRMLPS--------------------------------------CK--HYF-----HA---TC-VDVWLLSH-------------------------------------------------------------ATCPVC  42
TraesCS7D02G330600  CAVCLEEL-------------RGGAL-----------VRMLPS--------------------------------------CK--HYF-----HA---TC-VDVWLLSH-------------------------------------------------------------ATCPVC  42
TraesCS5A02G271600  CSVCLEDL-------------RAGEL-----------VRRLPA--------------------------------------CR--HVF-----HV---EC-IDMWLHSH-------------------------------------------------------------RSCPLC  42
TraesCS5D02G279300  CSVCLEDL-------------RAGEL-----------VRRLPA--------------------------------------CR--HVF-----HV---EC-IDMWLHSH-------------------------------------------------------------RSCPLC  42
TraesCS5B02G271900  CSVCLEDL-------------RAGET-----------VRRLPA--------------------------------------CM--HVF-----HV---EC-IDMWLHSH-------------------------------------------------------------RSCPLC  42
TraesCS6A02G095500  CSLCLEDL-------------EDGEM-----------VRQLPS--------------------------------------CK--HIF-----HV---DC-IDMWLHSH-------------------------------------------------------------TTCIIC  42
TraesCS6B02G123500  CSVCLEDL-------------EDGEM-----------VRELPS--------------------------------------CK--HIF-----HV---EC-IDMWLHSH-------------------------------------------------------------TTCPIC  42
TraesCS6A02G095600  CSVCLEDI-------------GDGEM-----------VRQLPT--------------------------------------CK--HLF-----HV---GC-IDMWLHSH-------------------------------------------------------------TTCPLC  42
TraesCS6D02G086300  CSVCLEDL-------------GDGEM-----------VRQLPT--------------------------------------CK--HLF-----HV---GC-IDMWLHSH-------------------------------------------------------------TTCPLC  42
TraesCS6B02G123700  CSVCLEDL-------------GDGEM-----------VRQLPT--------------------------------------CK--HLF-----HV---RC-IDMWLHSH-------------------------------------------------------------TTCPLC  42
TraesCS7A02G348100  CAVCLEDV-------------QGGEM-----------VRQLPP--------------------------------------CR--HLF-----HV---DC-IDMWLHTH-------------------------------------------------------------RTCPLC  42
TraesCS7B02G235200  CAVCLEDV-------------QGGEM-----------VRQLPP--------------------------------------CR--HLF-----HV---DC-IDMWLHTH-------------------------------------------------------------RTCPLC  42
TraesCS7D02G330900  CAVCLEDV-------------QGGEM-----------VRQLPP--------------------------------------CR--HLF-----HV---DC-IDMWLHTH-------------------------------------------------------------RTCPLC  42
TraesCS7D02G349700  CPVCLEDI-------------HGGEM-----------VRQLPA--------------------------------------CR--HLF-----HV---EC-IDMWLDSH-------------------------------------------------------------RTCPMC  42
TraesCS7D02G350200  CPVCLEDI-------------HGGEM-----------VRQLPA--------------------------------------CR--HLF-----HV---EC-IDMWLDSH-------------------------------------------------------------RTCPMC  42
TraesCS7B02G254300  CPVCLEDI-------------HGGEM-----------VRQLPA--------------------------------------CR--HLF-----HV---EC-IDMWLDSH-------------------------------------------------------------RTCPMC  42
TraesCS7B02G253800  CPVCLEDI-------------HGGEM-----------VRQLPA--------------------------------------CR--HLF-----HV---EC-IDMWLDSH-------------------------------------------------------------RTCPMC  42
TraesCS7A02G373200  CPVCLEDI-------------HGGEM-----------VRQLPA--------------------------------------CR--HLF-----HV---EC-IDMWLDSH-------------------------------------------------------------RTCPMC  42
TraesCS7A02G372900  CPVCLEDI-------------HGGEM-----------VRQLPA--------------------------------------CR--HLF-----HV---EC-IDMWLDSH-------------------------------------------------------------RTCPMC  42
TraesCS7B02G255400  CPVCLEDT-------------HGGEM-----------VRQLPA--------------------------------------CR--HLF-----HV---EC-IDMWLDSH-------------------------------------------------------------RTCPMC  42
TraesCS7A02G156900  CAVCLSEV-------------ADGEK-----------VRTLPK--------------------------------------CG--HRF-----HV---DC-IDMWFHSH-------------------------------------------------------------DTCPLC  42
TraesCS7B02G061100  CAVCLSEV-------------ADGEK-----------VRTLPK--------------------------------------CG--HRF-----HV---DC-IDMWFHSH-------------------------------------------------------------DTCPLC  42
TraesCS7D02G157300  CAVCLSEV-------------ADGEK-----------VRTLPK--------------------------------------CD--HRF-----HV---DC-IDMWFHSH-------------------------------------------------------------DTCPLC  42
TraesCS7A02G347900  CAVCLEAL-------------SGGEK-----------ARRLPV--------------------------------------CA--HTF-----HV---GC-IDMWFHSH-------------------------------------------------------------ATCPVC  42
                    1.......10........20........30........40........50........60........70........80........90.......100.......110.......120.......130.......140.......150.......160.......170......
```

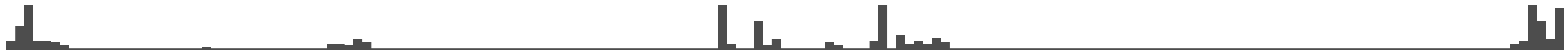

CLUSTAL 2.1 MULTIPLE SEQUENCE ALIGNMENT

File: C:/Users/HP/Downloads/clustal alignBB1.ps **Date: Wed May 20 03:00:39 2020**


```
                    *  *                                                                             *                 *                                                                        *
TraesCS7B02G235300  CAVCLEAL-------------SGGEK-----------ARRLPV--------------------------------------CA--HTF-----HV---GC-IDMWFHSH-------------------------------------------------------------ATCPVC    42
TraesCS7D02G331000  CAVCLEAL-------------SGGEK-----------ARRLPV--------------------------------------CT--HTF-----HV---GC-IDMWFHSH-------------------------------------------------------------ATCPVC    42
TraesCS7D02G331100  CAVCLEAL-------------SGGEK-----------VRRLPV--------------------------------------CA--HTF-----HV---GC-IDMWFHSH-------------------------------------------------------------ATCPVC    42
TraesCS7A02G348000  CAVCLEAL-------------SGREK-----------ARRLPV--------------------------------------CA--HMF-----HV---GC-IDMWFHSH-------------------------------------------------------------VTCSVC    42
TraesCS7A02G346400  CAVCLGAI-------------RVGAM-----------AKLLPA--------------------------------------CA--HVY-----HV---EC-IDLWLAAH-------------------------------------------------------------STCPLC    42
TraesCS7B02G236700  CAVCLGAI-------------RVGAM-----------AKLLPA--------------------------------------CA--HVY-----HV---EC-IDLWLASH-------------------------------------------------------------STCPLC    42
TraesCS7D02G332700  CAVCLAAI-------------RVGAM-----------AKLLPA--------------------------------------CA--HVY-----HV---EC-IDLWLASH-------------------------------------------------------------STCPLC    42
TraesCS7A02G346500  CSVCLGAL-------------QVGET-----------VRLLPA--------------------------------------CL--HLY-----HA---EC-IDPWLDAH-------------------------------------------------------------STCPLC    42
TraesCS7B02G236600  CSVCLGAM-------------QVGET-----------VRLLPA--------------------------------------CL--HLY-----HA---EC-IDPWLDAH-------------------------------------------------------------STCPLC    42
TraesCS7D02G332500  CSVCLGAM-------------QVGEM-----------VRLLPA--------------------------------------CL--HLY-----HT---EC-IDPWLAAH-------------------------------------------------------------STCPVC    42
TraesCS7A02G346700  CSVCLGAL-------------QLGDT-----------VRLLPA--------------------------------------CL--HVY-----HA---EC-IDPWLGAH-------------------------------------------------------------STCPIC    42
TraesCS7D02G332200  CSVCLGAL-------------QLGDT-----------VRLLPA--------------------------------------CL--HVY-----HA---EC-IDPWLGAH-------------------------------------------------------------STCPIC    42
TraesCS7D02G332600  CSVCLGVL-------------QLGET-----------VRLLPA--------------------------------------CL--HLY-----HA---EC-IDPWLEAH-------------------------------------------------------------STCPIC    42
TraesCS7A02G346800  CSVCLGAF-------------QLGET-----------VRLLPA--------------------------------------CL--HLY-----HV---EC-IDPWLDAH-------------------------------------------------------------STCPIC    42
TraesCS7D02G332000  CSVCLGAF-------------QLGET-----------VRLLPA--------------------------------------CL--HLY-----HV---EC-IDPWLDAH-------------------------------------------------------------STCPLC    42
TraesCS7A02G346900  CSVCLGVF-------------QLGET-----------VRLLPA--------------------------------------CM--HLY-----HV---EC-IDPWLDAH-------------------------------------------------------------STCPIC    42
TraesCS7B02G236100  CSVCLGVF-------------QLGET-----------VRLLPA--------------------------------------CM--HLY-----HV---EC-IDPWLDAH-------------------------------------------------------------STCPIC    42
TraesCS7A02G347000  CSVCLGVF-------------QLGET-----------VRLLPA--------------------------------------CL--HLY-----HV---EC-IDPWLDAH-------------------------------------------------------------STCPIC    42
TraesCS7B02G236200  CSVCLGVF-------------QLGEA-----------VRLLPA--------------------------------------CM--HLY-----HV---EC-IDPWLDAH-------------------------------------------------------------STCPLC    42
TraesCS7A02G347100  CSVCLGAF-------------LLGET-----------VRLLPV--------------------------------------CL--HLY-----HV---GC-IDPWLDAH-------------------------------------------------------------STCPLC    42
TraesCS7D02G331900  CSVCLGAF-------------LLGET-----------VRLLPV--------------------------------------CL--HLY-----HV---GC-IDPWLDAH-------------------------------------------------------------STCPLC    42
TraesCS7B02G236000  CSVCLGTV-------------QAGEM-----------VRLLPL--------------------------------------CK--HLY-----HV---EC-IDMWLASH-------------------------------------------------------------DTCPLC    42
TraesCS7D02G331800  CSVCLGTV-------------QAGEM-----------VRLLPL--------------------------------------CK--HLY-----HV---EC-IDMWLASH-------------------------------------------------------------DTCPLC    42
TraesCS7A02G347200  CSVCLGTV-------------QAGEM-----------VRLLPL--------------------------------------CK--HLY-----HV---EC-IDMWLASH-------------------------------------------------------------DTCPLC    42
TraesCS7A02G563700  CAVCLAEV-------------RPKEV-----------VKQLPA--------------------------------------CT--HLF-----HD---RC-IDEWLWSH-------------------------------------------------------------RTCPVC    42
TraesCS7D02G547300  CAVCLAEV-------------RPKEV-----------VKQLPA--------------------------------------CT--HLF-----HD---RC-IDEWLWSH-------------------------------------------------------------RTCPVC    42
TraesCS7A02G347700  CAVCLAEV-------------RPKEV-----------VKQLPA--------------------------------------CT--HLF-----HD---RC-IDEWLWSH-------------------------------------------------------------RTCPVC    42
TraesCS7B02G235600  CAVCLAEI-------------KPKEV-----------VKQLPA--------------------------------------CT--HLF-----HD---RC-IDEWLWSH-------------------------------------------------------------RTCPVC    42
TraesCS7D02G331300  CAVCLAEI-------------KPKEV-----------VKQLPA--------------------------------------CT--HLF-----HD---RC-IDEWLWSH-------------------------------------------------------------RTCPVC    42
TraesCS1A02G311600  CAVCLGEV-------------EKGET-----------VKRLPA--------------------------------------CL--HMF-----HQ---QC-IDPWLHEH-------------------------------------------------------------STCPVC    42
TraesCS1B02G323300  CSVCLGEV-------------EKGET-----------VKRLPA--------------------------------------CL--HMF-----HQ---QC-IDPWLHEH-------------------------------------------------------------STCPVC    42
TraesCS1D02G311900  CPVCLGEV-------------EKGET-----------VKRLPA--------------------------------------CL--HMF-----HQ---QC-IDPWLQEH-------------------------------------------------------------STCPVC    42
TraesCS1B02G323200  CAVCLVEV-------------EKGDT-----------VKRLPA--------------------------------------CL--HMF-----HQ---QC-IDPWLHEH-------------------------------------------------------------STCPVC    42
TraesCS1D02G311600  CAVCLGEV-------------EKGDT-----------MKRLPA--------------------------------------CL--HMF-----HQ---QC-IDPWLHEH-------------------------------------------------------------STCPVC    42
TraesCS1D02G311700  CAVCLGEV-------------EKGDA-----------VKRLPA--------------------------------------CL--HLF-----HQ---QC-INAWLHEH-------------------------------------------------------------STCPVC    42
TraesCS3A02G410900  CTICLGEV-------------EKGDA-----------VKRMPV--------------------------------------CL--HMF-----HQ---QC-VDPWLHEH-------------------------------------------------------------STCPVC    42
TraesCS3B02G444200  CTICLGEV-------------EKGDM-----------VKRMPA--------------------------------------CL--HMF-----HQ--NMC-VDSWLHEH-------------------------------------------------------------STCPVC    43
TraesCS3D02G405400  CTICLGEV-------------EKGDA-----------VKRMPV--------------------------------------CL--HMF-----HR---QC-VDQWLHEH-------------------------------------------------------------STCPVC    42
TraesCS3B02G444100  CTICLGKV-------------EKGDA-----------VKRMPA--------------------------------------CL--HMF-----HQ---QC-IDLWLHEH-------------------------------------------------------------STCPVC    42
TraesCS3B02G444300  CVICLGKV-------------KKGDA-----------VKRMPA--------------------------------------CL--HMF-----HQ---QC-IDPWLHEH-------------------------------------------------------------STCPVC    42
TraesCS1D02G296800  CAVCLGEM-------------ENGDM-----------VKRLPA--------------------------------------CL--HMF-----HQ---HC-IDVWLRGH-------------------------------------------------------------STCPVC    42
TraesCS6A02G172100  CAVCLGEV-------------EKGEA-----------VRRMPV--------------------------------------CL--HVF-----HT---EC-IDRWLRSH-------------------------------------------------------------ATCPIC    42
TraesCS7B02G235500  CVVCLGEV-------------GKGEE-----------TRRLPV--------------------------------------CR--HVF-----HR---QC-VERWLMER-------------------------------------------------------------STCPVC    42
TraesCS1A02G346900  CSICLCEY-------------RDGEM-----------QRIMPE--------------------------------------CR--HRF-----HL---MC-LDAWLRRS-------------------------------------------------------------ASCPVC    42
TraesCS1B02G361000  CSICLCEY-------------RDGEM-----------QRIMPE--------------------------------------CR--HRF-----HL---MC-LDAWLRRS-------------------------------------------------------------ASCPVC    42
TraesCS3B02G313100  CSICLSEY-------------MDGEM-----------LRLMPE--------------------------------------CR--HRF-----HV---MC-LDAWLRRS-------------------------------------------------------------ASCPVC    42
TraesCS3D02G279200  CSICLSEY-------------MDGEM-----------LRLMPE--------------------------------------CR--HRF-----HV---MC-LDAWLRRS-------------------------------------------------------------ASCPVC    42
TraesCS3A02G278900  CSICLSEY-------------MDGVM-----------LRLMPE--------------------------------------CR--HRF-----HV---MC-LDAWLRRS-------------------------------------------------------------ASCPVC    42
TraesCS2A02G399700  CSICLGEY-------------TEKEL-----------LRIIPT--------------------------------------CQ--HNF-----HR---TC-LDLWLQKQ-------------------------------------------------------------TTCPIC    42
TraesCS2D02G397200  CSICLGEY-------------TEKEL-----------LRIIPT--------------------------------------CQ--HNF-----HR---TC-LDLWLQKQ-------------------------------------------------------------TTCPIC    42
TraesCS2B02G417700  CSICLGEY-------------TEKEL-----------LRIIPT--------------------------------------CR--HNF-----HR---TC-LDLWLQKQ-------------------------------------------------------------TTCPIC    42
TraesCS6B02G268900  CSICLGEY-------------DEKEI-----------LRIMPT--------------------------------------CR--HNF-----HL---SC-IDIWLEKQ-------------------------------------------------------------TTCPIC    42
TraesCS6D02G237700  CSICLGEY-------------DEKEI-----------LRIMPT--------------------------------------CR--HNF-----HL---SC-IDIWLEKQ-------------------------------------------------------------TTCPIC    42
TraesCS6A02G256500  CSICLGEY-------------DEKEI-----------LRIMPT--------------------------------------CR--HNF-----HL---SC-IDIWLEKQ-------------------------------------------------------------TTCPIC    42
TraesCS5A02G136900  CAICLGEF-------------DGAAT-----------LRRGSR--------------------------------------CA--HCF-----HA---GC-AERWLRVS-------------------------------------------------------------ATCPVC    42
TraesCS5B02G135600  CAICLGEF-------------HGVAA-----------LRRGSR--------------------------------------CA--HCF-----HA---GC-AERWLRVS-------------------------------------------------------------ATCPVC    42
TraesCS5A02G147900  CVICLAQYEG-------------GER-----------CSVLPG--------------------------------------CG--HVF-----HR---GCVATWLHTT-------------------------------------------------------------HNTCPLC   43
TraesCS5D02G144900  CVICLAQYEG-------------GER-----------CSVLPG--------------------------------------CG--HVF-----HR---GCVATWLHTT-------------------------------------------------------------HNTCPLC   43
TraesCS1B02G327600  CTICIADFRP-------------RAR-----------VAVLPT--------------------------------------CG--HVF-----HR---HCLREVVAGQ-------------------------------------------------------------SPRCPLC   43
TraesCS5B02G374600  CAICLSEYGEG----------EAGEL-----------VRVVPA--------------------------------------CG--HFY-----HA---SCDVDRWLRK-------------------------------------------------------------RRTCPLC   46
TraesCS5B02G374700  CAICLSEYGEG----------EAGEL-----------VRVVPA--------------------------------------CG--HFY-----HA---SCDVDRWLRK-------------------------------------------------------------RRTCPLC   46
TraesCS5D02G382000  CAICLSEYGEG----------EAGEL-----------VRVVPA--------------------------------------CG--HFY-----HA---SCDVDKWLRK-------------------------------------------------------------RRTCPLC   46
TraesCS5D02G372500  CAICLSEYGEG----------EAGEL-----------VRVVPA--------------------------------------CG--HFY-----HA---SCEIDGWLRK-------------------------------------------------------------RRTCPLC   46
TraesCS5A02G372600  CAICQWEYGEG----------DAGEL-----------VRVVPA--------------------------------------CG--HFY-----HA---SCDVDRWLRK-------------------------------------------------------------RHTCPLC   46
TraesCS5D02G382100  CALCLSDYGEG----------DAGEL-----------VRVVPA--------------------------------------CG--HFY-----HA---SCGVDKWLRK-------------------------------------------------------------HRTCPLC   46
TraesCS5D02G382200  CAFCQSEYAK-----------APEEV-----------VRVVPA--------------------------------------CG--HFF-----HA---ACDADRWIRA-------------------------------------------------------------RRRCPLC   45
TraesCS2B02G413200  CRVCLERLEL-------------TDE-----------VRPLGN--------------------------------------CS--HAF-----HR---GC-IDRWIDIG------------------------------------------------------------EVTCPLC   43
TraesCS2D02G392500  CRVCLERLEL-------------TDE-----------VRPLGN--------------------------------------CS--HAF-----HR---GC-IDRWIDVG------------------------------------------------------------EVTCPLC   43
TraesCS2A02G395100  CRVCLERLEP-------------TDE-----------VRPLGN--------------------------------------CA--HAF-----HR---GC-IDRWIDVG------------------------------------------------------------EATCPLC   43
TraesCS6A02G259200  CIVCLERLRA-------------ADE-----------VRRLGN--------------------------------------CA--HAF-----HR---GC-IDGWIDLG------------------------------------------------------------RTTCPLC   43
TraesCS6D02G240300  CIVCLERLRA-------------ADE-----------VRRLGN--------------------------------------CA--HAF-----HR---GC-IDGWIDLG------------------------------------------------------------RTTCPLC   43
TraesCS6B02G286500  CIVCLERLWA-------------TDE-----------VRRLGN--------------------------------------CA--HAF-----HR---GC-IDGWIDLG------------------------------------------------------------RTTCPLC   43
TraesCS7B02G188200  CAVCLGALEA-------------RHR-----------VRELGN--------------------------------------CA--HAF-----HK---AC-IDKWVDKG------------------------------------------------------------QATCPLC   43
TraesCS7D02G295800  CAVCLGALEA-------------RHR-----------VRELGN--------------------------------------CA--HAF-----HK---AC-IDKWVDKG------------------------------------------------------------QATCPLC   43
TraesCS7A02G300000  CAVCLGALEA-------------RHR-----------VRELGN--------------------------------------CA--HAF-----HK---AC-IDKWVDKG------------------------------------------------------------QATCPLC   43
                    1.......10........20........30........40........50........60........70........80........90.......100.......110.......120.......130.......140.......150.......160.......170......
```

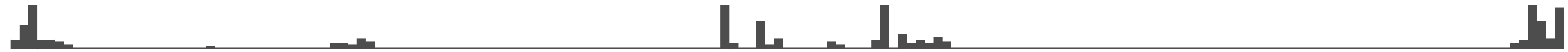

```
                    *  *                                                                             *                 *                                                                        *
TraesCS2B02G413800  CAVCLSGIGG-------------GDE-----------VRRLSN--------------------------------------CR--HAF-----HR---GC-LDRWMEHD------------------------------------------------------------QRTCPLC   43
TraesCS2D02G393400  CAVCLSGIGG-------------GDE-----------VRRLSN--------------------------------------CR--HAF-----HR---GC-LDRWMEHD------------------------------------------------------------QRTCPLC   43
TraesCS6A02G258800  CAVCLCGIGG-------------CHE-----------VRRLPN--------------------------------------CR--HVF-----HR---GC-IDRWMGHG------------------------------------------------------------QRTCPLC   43
TraesCS6D02G240000  CAVCLCSIGG-------------GDE-----------VRRLPN--------------------------------------CR--HVF-----HR---GC-IDRWMGHG------------------------------------------------------------QRTCPLC   43
TraesCS6B02G266600  CAVCLCGIGG-------------GDE-----------VRRLSN--------------------------------------CR--HVF-----HR---GC-IDRWMGHQ------------------------------------------------------------QRTCPLC   43
TraesCS4A02G463900  CAVCLGNFHG-------------AAH-----------VRRPRG--------------------------------------CR--HVF-----HR---AC-LDRWAAHG------------------------------------------------------------HSTCPLC   43
TraesCS7A02G025100  CAVCLGDFHG-------------AAR-----------VRRPRG--------------------------------------CR--HVL-----HR---AC-LDRWAAHG------------------------------------------------------------HSTCPLC   43
TraesCS7A02G025200  CAVCLGDFHA-------------AAC-----------VRRPRG--------------------------------------CR--HVF-----HR---AC-LDRWAAHG------------------------------------------------------------HSTCPLC   43
TraesCS7D02G043400  CAVCLGDFHC-------------AAC-----------VRRPRG--------------------------------------CR--HVF-----HR---AC-LDRWAAHG------------------------------------------------------------HSTCPLC   43
TraesCS4A02G464300  CAVCLGDFHG-------------AAR-----------VRRPRG--------------------------------------CR--HVF-----HR---GC-LDRWAAHG------------------------------------------------------------HRTCPLC   43
TraesCS7A02G024600  CAVCLGDFHG-------------AAR-----------VRRPRG--------------------------------------CR--HVF-----HR---GC-LDRWAAHG------------------------------------------------------------HRTCPLC   43
TraesCS7D02G022600  CAVCLGDFHG-------------AAR-----------VRRPRG--------------------------------------CR--HVF-----HR---GC-LDRWASHG------------------------------------------------------------HHTCPLC   43
TraesCS7D02G023000  CAVCFGEFHG-------------AAR-----------VRRPRG--------------------------------------CR--HVF-----HR---GC-LDRWAAHG------------------------------------------------------------RRTCPLC   43
TraesCS7D02G023100  CAVCLGDFHG-------------AAH-----------VRRPRG--------------------------------------CW--HIF-----HR---AC-LDRWAAYG------------------------------------------------------------HRTCPLC   43
TraesCS4A02G463800  CAVCLADFHG-------------ATR-----------VRRPHG--------------------------------------CG--HVF-----HR---AC-LDRWAAYG------------------------------------------------------------HRTCPLC   43
TraesCS4A02G465400  CVVCLSDFHG-------------AAR-----------VRRPRG--------------------------------------CQ--HVF-----HF---GC-LDRWAAHG------------------------------------------------------------HRTCPLC   43
TraesCS4A02G468600  CVVCLSDFHG-------------TAC-----------VRRPRR--------------------------------------CR--HVF-----HF---GC-LDRWAAHG------------------------------------------------------------HRTCPLC   43
TraesCS7A02G347800  CCVCLAGMRE-------------EQA-----------LRDLPR--------------------------------------CG--HRF-----HE---KC-IGKWLKTG------------------------------------------------------------HPTCPVC   43
TraesCS7B02G235400  CCVCLAGMRE-------------EQA-----------LRDLPR--------------------------------------CG--HRF-----HE---KC-IGKWLKTG------------------------------------------------------------HPTCPVC   43
TraesCS7D02G331200  CCVCLAGMRE-------------EQA-----------LRDLPR--------------------------------------CG--HRF-----HE---KC-IGKWLKTG------------------------------------------------------------HPTCPVC   43
TraesCS5A02G326800  CAVCLAEYAA-------------GDE-----------VRVLPA--------------------------------------CR--HGF-----HR---EC-VDRWLLTR------------------------------------------------------------APTCPVC   43
TraesCS5A02G326900  CAVCLAEYAA-------------GDE-----------VRVLPA--------------------------------------CR--HGF-----HR---EC-VDRWLLTR------------------------------------------------------------APTCPVC   43
TraesCS5D02G333100  CAVCLAEYAA-------------GDE-----------VRVLPA--------------------------------------CR--HGF-----HR---EC-VDRWLLTR------------------------------------------------------------APTCPVC   43
TraesCS5D02G333200  CAVCLAEYAA-------------GDE-----------VRVLPA--------------------------------------CR--HGF-----HR---EC-VDRWLLTR------------------------------------------------------------APTCPVC   43
TraesCS6B02G032400  CAICLAEFAD-------------GDA-----------VRAMPS--------------------------------------CG--HGF-----HA---RC-IERWLAVPAPAAGS-----------------------------------------------------RRPSCPTC   50
TraesCS6D02G027000  CAICLAEFAE-------------GDA-----------VRAMPA--------------------------------------CG--HGF-----HA---RC-IERWLAVPTSAAGS-----------------------------------------------------RRPSCPTC   50
TraesCS6A02G023700  CAICLAEFAD-------------GDA-----------VRAMPA--------------------------------------CG--HGF-----HA---CC-IERWLAVPASAA-------------------------------------------------------RRPSCPTC   48
TraesCS6D02G039100  CCICAQEFAP-------------TDM-----------ILALPA--------------------------------------CL--HKF-----HE---RC-IIPWIRGHAPYC-----------------------------------------------------------CPFC   44
TraesCS4A02G174400  CPVCLADFAD-------------GEA-----------VRVLPE--------------------------------------CL--HYF-----HA---DC-IDTWLRAG------------------------------------------------------------NTSCPMC   43
TraesCS4D02G141200  CPVCLADFAD-------------GEA-----------VRVLPE--------------------------------------CL--HYF-----HE---DC-IDTWLRAG------------------------------------------------------------NTSCPMC   43
TraesCS4B02G143100  CPVCLADFAD-------------GEA-----------VRVLPE--------------------------------------CL--HYF-----HA---DC-IDTWLRAR------------------------------------------------------------NTSCPMC   43
TraesCS4A02G174600  CPVCLSDFAD-------------GET-----------VRVLPA--------------------------------------CM--HYF-----HA---AC-VGEWLRR-------------------------------------------------------------NTTCPVC   42
TraesCS4B02G142800  CPVCLSDFAD-------------GET-----------VRVLPA--------------------------------------CM--HYF-----HA---AC-VGEWLRR-------------------------------------------------------------NTTCPVC   42
TraesCS4D02G141100  CPVCLSDFAD-------------GET-----------VRVLPA--------------------------------------CM--HYF-----HA---AC-VGEWLRR-------------------------------------------------------------NTTCPVC   42
TraesCS4A02G174500  CPVCLSDFAD-------------GEA-----------VSVLPA--------------------------------------CM--HYF-----HA---AC-VGEWMRA-------------------------------------------------------------RATCPLC   42
TraesCS1A02G205100  CSVCLQDL-------------EVGES-----------ARRLPG--------------------------------------CR--HVF-----HA---PC-IDRWLVRH-------------------------------------------------------------ASCPLC   42
TraesCS1B02G218700  CSVCLQDL-------------EVGES-----------ARRLPG--------------------------------------CR--HVF-----HA---PC-IDRWLVRH-------------------------------------------------------------ASCPLC   42
TraesCS1D02G208400  CSVCLQDL-------------EVGES-----------ARRLPG--------------------------------------CR--HVF-----HA---PC-IDRWLVRH-------------------------------------------------------------ASCPLC   42
TraesCS7A02G273000  CSVCLQEF-------------EAGEA-----------ARSLPE--------------------------------------CR--HTF-----HM---SC-IDGWLCRH-------------------------------------------------------------ASCPLC   42
TraesCS7B02G171000  CSVCLQEF-------------EAGEA-----------ARSLPE--------------------------------------CR--HTF-----HM---SC-IDGWLCRH-------------------------------------------------------------ASCPLC   42
TraesCS7D02G273100  CSVCLQEF-------------EAGEA-----------ARSLPE--------------------------------------CR--HTF-----HM---SC-IDGWLCRH-------------------------------------------------------------ASCPLC   42
TraesCS7B02G452400  CSVCLQDF-------------QIGEK-----------VRSLPD--------------------------------------CL--HVF-----HV---PC-IDGWLIKH-------------------------------------------------------------GSCPLC   42
TraesCS7D02G522000  CSVCLQDF-------------QIGEK-----------VRSLPD--------------------------------------CL--HVF-----HV---PC-IDGWLIKH-------------------------------------------------------------GSCPLC   42
TraesCS7A02G534800  CSVCLQDF-------------QIGEK-----------VRSLPD--------------------------------------CL--HVF-----HV---PC-IDGWLIKH-------------------------------------------------------------GSCPLC   42
TraesCS3B02G443900  CSVCLQDF-------------GSRQF-----------VRALPQ--------------------------------------CQ--HIF-----HV---RC-IDDWLPRN-------------------------------------------------------------STCPLC   42
TraesCS3D02G405200  CSVCLQDF-------------GSRQF-----------VRALPQ--------------------------------------CQ--HIF-----HV---RC-IDDWLQRN-------------------------------------------------------------ASCPLC   42
TraesCS3A02G410600  CSVCLQDL-------------RPQQF-----------VRALPQ--------------------------------------CQ--HIF-----HV---RC-IDDWLQRN-------------------------------------------------------------ASCPLC   42
TraesCS7B02G443600  CSVCLQDF-------------GSRQF-----------VRALPQ--------------------------------------CG--HIF-----HV---PC-IDNWLQRN-------------------------------------------------------------ASCPLC   42
TraesCS7D02G514900  CSVCLQDF-------------GSCQF-----------VRVLPQ--------------------------------------CQ--HIF-----HV---PC-IDNWLQRN-------------------------------------------------------------ASCPLC   42
TraesCS7A02G526000  CSVCLQDF-------------ASRQY-----------VRALPQ--------------------------------------CR--HIF-----HV---PC-IDNWLQRN-------------------------------------------------------------ASCPLC   42
TraesCS1A02G207000  CPVCLEAFG-------------DDDG-----------VKVVPA--------------------------------------CG--HVF-----HA---PC-IDRWLD-------------------------------------------------------------VRNSCPVC   42
TraesCS1B02G220800  CPVCLEAFG-------------DDDG-----------VKVVPA--------------------------------------CG--HVF-----HA---PC-IDRWLD-------------------------------------------------------------VRNSCPVC   42
TraesCS1D02G210300  CPVCLEAFG-------------DDDG-----------VKVVPA--------------------------------------CG--HVF-----HA---PC-IDRWLD-------------------------------------------------------------VRNSCPVC   42
TraesCS4B02G361300c CPVCLEPLRA------------GSGD-----------IRAAHA--------------------------------------CG--HVF-----HS---RC-IERWVR-------------------------------------------------------------RRGSCPVC   43
TraesCS4B02G361300d CPVCLEPLSV------------GGGD-----------MKAAHV--------------------------------------CG--HVF-----HS---RC-IERWLR-------------------------------------------------------------HRNSCPVC   43
TraesCS4B02G361300b CPVCLEPLLL------------DCGD-----------VRAAHA--------------------------------------CG--HVF-----HS---CC-IEPWLN-------------------------------------------------------------QQGSCPVC   43
TraesCS4B02G361300a CPVCLEPLH-------------GRTN-----------IMAAHE--------------------------------------CA--HVF-----HA---SC-IEPWLH-------------------------------------------------------------NRGSCPVC   42
TraesCS6B02G449700  CAVCLTELQ-------------LRES-----------VKAIPA--------------------------------------CG--HVF-----HP---PC-IDRWLQSLAAAGA------------------------------------------------------GHASCPLC   49
TraesCS6D02G389000  CAVCLTELQ-------------LQES-----------VKAIPA--------------------------------------CG--HVF-----HP---PC-IDRWLQSLAASGA------------------------------------------------------GHASCPLC   49
TraesCS1A02G422300  CAICLEAL-------------VAGAE-----------CSEVPA--------------------------------------CR--HVF-----HR---GC-LALWIKSK-------------------------------------------------------------STCPLC   42
TraesCS1D02G430500  CAICLEAL-------------VGGAE-----------CSEVPA--------------------------------------CR--HVF-----HR---GC-LALWIKSK-------------------------------------------------------------STCPLC   42
TraesCS1D02G430400  CAICLEAL-------------VGGAE-----------CSEVPA--------------------------------------CR--HVF-----HR---GC-LALWIGSS-------------------------------------------------------------NTCPLC   42
TraesCS7A02G205000  CAICLEEF-------------QQGQP-----------CSEVPA--------------------------------------CR--HVF-----HR---YC-IDVWMKSK-------------------------------------------------------------ATCPLC   42
TraesCS7B02G112200  CAICLEEL-------------QQGQA-----------CSEVPV--------------------------------------CR--HVF-----HR---DC-IDAWMKSQ-------------------------------------------------------------TTCPLC   42
TraesCS7B02G112300  CAICQDEF-------------LQGQM-----------CSEVPA--------------------------------------CR--HLF-----HR---DC-IEVWRKSK-------------------------------------------------------------TTCPLC   42
TraesCS2A02G081500  CPICLQEF-EA----------GGERA-----------GRRLPA--------------------------------------CS--HVF-----HL---EC-IRSWLLRK-------------------------------------------------------------PDCPMC   44
TraesCS2D02G079500  CPICLQEF-DA----------GGERE-----------GRRLPA--------------------------------------CS--HVF-----HL---EC-IRSWLLRK-------------------------------------------------------------PHCPMC   44
TraesCS5B02G335400  CPICLHEF-QA----------G-ESA-----------RRLPA--------------------------------------CG--HVF-----HL---AC-IDSWLLWK-------------------------------------------------------------PHCPMC   42
TraesCS5D02G341100  CPICLHEF-QA----------G-ESS------------RRLPA--------------------------------------CG--HVF-----HL---AC-IDSWLLWK-------------------------------------------------------------PHCPMC   42
TraesCS5A02G336400  CPICLHEF-QP----------G-ESA------------RRLPA--------------------------------------CG--HVF-----HL---AC-IDSWLLWK-------------------------------------------------------------PHCPMC   42
TraesCS3B02G484400  CPICMEELVPN----------SSRGF-----------VTRLPG--------------------------------------CL--HSF-----HE---SC-IAQWFCRA-------------------------------------------------------------ATCPCC   45
TraesCS4B02G091300  CCICLSYLVED----------QD--------------AVRLPG--------------------------------------CS--HAF-----HR---SC-LTPWLQRA-------------------------------------------------------------KTCPMC   42
TraesCS1A02G255600  CAICREEMA------------PGRGG-----------VCVLRP--------------------------------------CG--HRF-----HW---PC-ALRWLARR-------------------------------------------------------------NTCPCC   43
TraesCS1B02G266300  CAICREEMA------------PGRGG-----------VCVLRP--------------------------------------CG--HRF-----HW---PC-ALRWLARR-------------------------------------------------------------NTCPCC   43
TraesCS1D02G254900  CAICREEMA------------PGRGG-----------VCVLRP--------------------------------------CG--HRF-----HW---PC-ALRWLARR-------------------------------------------------------------NTCPCC   43
                    1.......10........20........30........40........50........60........70........80........90.......100.......110.......120.......130.......140.......150.......160.......170......
```

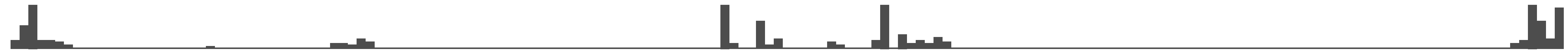

CLUSTAL 2.1 MULTIPLE SEQUENCE ALIGNMENT

File: C:/Users/HP/Downloads/clustal alignBB1.ps **Date: Wed May 20 03:00:39 2020**


```
                        *  *                                                                             *                 *                                                                        *
TraesCS3A02G521400      CRICLDEFR------------DGGKS-----------GPVNLP--------------------------------------CS--HAY-----HA---HC-MLTWLDRG-------------------------------------------------------------AICPTC    43
TraesCS3B02G591100      CRICFDEFR------------DGGKS-----------GSVNLP--------------------------------------CS--HAY-----HA---HC-MLTWLDRG-------------------------------------------------------------ANCPTC    43
TraesCS3A02G345400      CAICKGPLA------------AGGGR-----------CRRLRA--------------------------------------CG--HVY-----HA---EC-VDLWLQRK-------------------------------------------------------------PICPLC    43
TraesCS3D02G339500      CAICKGPLA------------AGGGR-----------CRRLRA--------------------------------------CG--HVY-----HA---EC-VDLWLQRK-------------------------------------------------------------PICPLC    43
TraesCS3B02G377500      CAICKGPLA------------VGGGR-----------CRRLRA--------------------------------------CG--HVY-----HA---EC-VDLWLQRK-------------------------------------------------------------PICPLC    43
TraesCS2B02G413100      CAICLAALD------------VGGQL-----------VTELCN--------------------------------------CS--HAF-----HG---AC-IGYWIGSGE-----------------------------------------------------------AGTCPLC    45
TraesCS2D02G392600      CAICLAALD------------VGGQL-----------VTELCN--------------------------------------CS--HAF-----HA---AC-IDSWIGSGE-----------------------------------------------------------AGTCPMC    45
TraesCS5A02G147800      CVICMEPYE------------AGGDR-----------CRVLPA--------------------------------------CG--HMF-----HR---RC-LRTWLRTS-------------------------------------------------------------STCPLC    43
TraesCS2A02G352900      CAVCLSELAGAA-------GCSGDSEPSA------VLVRVLPG--------------------------------------CG--HGF-----HD---EC-IGRWLLLR-------------------------------------------------------------PECPLC    53
TraesCS2D02G351200      CAVCLSELVGAA-------GYSGDSEPSS------ALVRVLSG--------------------------------------CG--HGF-----HD---EC-IGRWLLLR-------------------------------------------------------------PECPLC    53
TraesCS2B02G370700      CAVCLSELVGAA-------EFSGDSEPSA------VPVRVLPG--------------------------------------CG--HWF-----HD---EC-IGRWLLLR-------------------------------------------------------------PECPLC    53
TraesCS1A02G090900      CLICFDDF-------------VPGCY---------KKIRTMD---------------------------------------CT--HSF-----HQ---RC-IFDWLLID-------------------------------------------------------------RRCPLC    43
TraesCS1B02G118700      CLICFDDF-------------VPGCY---------KKIRTMD---------------------------------------CS--HSF-----HQ---RC-IFDWLLID-------------------------------------------------------------RRCPIC    43
TraesCS1B02G118800      CAVCLEGF-------------VAGG----------KKLRMMP---------------------------------------CS--HSF-----HQ---RC-IFDLLLRS-------------------------------------------------------------RFCPVC    42
TraesCS1D02G099500      CAVCLEGF-------------VAGG----------KKLRTMP---------------------------------------CS--HSF-----HQ---RC-IFDLLLRS-------------------------------------------------------------RFCPVC    42
TraesCS5D02G096500      CAVCLEDF-------------VEGG----------KKLRRMPC--------------------------------------CY--HSF-----HQ---RC-IFDWLRFN-------------------------------------------------------------RRCPVC    43
TraesCS5D02G096600      CAVCLEDF-------------VEGG----------KKLRRMP---------------------------------------CY--HSF-----HQ---RC-IFDWLRFN-------------------------------------------------------------RRCPVC    42
TraesCS5B02G090100      CAVCLEDF-------------VEGG----------KKLRRMP---------------------------------------CY--HSF-----HQ---RC-IFDWLRFN-------------------------------------------------------------RRCPVC    42
TraesCS5A02G097000      CGVCLEDF-------------LEGG----------KKLRRMP---------------------------------------CY--HSF-----HQ---RC-IFDWLRFN-------------------------------------------------------------RRCPVC    42
TraesCS5B02G090000      CAVCLEEF-------------IDGG----------KKLRMMP---------------------------------------CY--HSF-----HQ---RC-IFDWLRLN-------------------------------------------------------------RICPVC    42
TraesCS5D02G096000      CAVCLEEF-------------VDGG----------KKLRMMP---------------------------------------CY--HSF-----HQ---RC-IFDWLRLN-------------------------------------------------------------RICPVC    42
TraesCS3B02G509100      CAVCLEDL-------------VAGG----------RKLRKMD---------------------------------------CS--HCF-----HQ---RC-IFRWLHVS-------------------------------------------------------------RLCPMC    42
TraesCS3D02G459900      CAVCLEDL-------------VAGG----------RKLRKMD---------------------------------------CS--HCF-----HQ---RC-IFRWLHVS-------------------------------------------------------------RLCPMC    42
TraesCS3A02G464800      CAVCLEDL-------------VAGG----------RKLRKMD---------------------------------------CS--HCF-----HQ---RC-IFRWLHVS-------------------------------------------------------------RLCPMC    42
TraesCS2A02G511100      CAICLDG--------------LEGEP-----------AVQLPG--------------------------------------CP--HAF-----HR---RC-ISTWFSKK-------------------------------------------------------------TTCPMC    41
TraesCS2D02G512500      CAICLDG--------------LEGEP-----------AVELPA--------------------------------------CP--HAF-----HR---RC-ISTWFSKK-------------------------------------------------------------TTCPIC    41
TraesCS3B02G501700      CAICMVD----E-------DASWDEE-----------TVALP---------------------------------------CS--HAF-----HT---GC-ILTWFHRA-------------------------------------------------------------PTCPTC    43
TraesCS3D02G454100      CAICMVD----E-------DASWDEE-----------TVALP---------------------------------------CS--HAF-----HS---GC-ILTWFHRA-------------------------------------------------------------PTCPTC    43
TraesCS3A02G460600      CAICMVD----E-------DASWDEE-----------TVALP---------------------------------------CS--HAF-----HS---GC-ILRWFHRA-------------------------------------------------------------PTCPTC    43
TraesCS3D02G451600      CVICLLDL---E-------SEEDDEE-----------QVRLP---------------------------------------CS--HAF-----HT---PC-IELWFDRA-------------------------------------------------------------STCPTC    44
TraesCS3D02G453900      CAICMSD------------EGTNDEE-----------TVRLP---------------------------------------CS--HVF-----HG---RC-IGPWFHRV-------------------------------------------------------------STCPTC    42
TraesCS5B02G558000      CRVCVE--------------AKEDT------------MARLP---------------------------------------CS--HSF-----HR---GC-ILPWFDKV-------------------------------------------------------------ATCPMC    39
TraesCS5D02G564400      CRVCVE--------------AKEDA------------MVRLP---------------------------------------CS--HSF-----HR---GC-ILPWFDKV-------------------------------------------------------------ATCPMC    39
TraesCS4A02G320900      CRVCME--------------AKEDT------------MVRLP---------------------------------------CS--HSF-----HR---GC-ILPCFHKV-------------------------------------------------------------ATCPMC    39
TraesCS5B02G557600      CPICMED----F--------VRDDSDTNDDS--DTTVRVRLP---------------------------------------CS--HSF-----HC---DC-ILPWFYKV-------------------------------------------------------------AKCPKC    51
TraesCS4A02G321200      CCVCMED----F-------AAAIDGD----------TTVRLP---------------------------------------CS--HAF-----HR---AC-ILPWFYKA-------------------------------------------------------------STCPKC    44
TraesCS5D02G551700      CCVCMED----F-------VAPRDSD----------TTVRLP---------------------------------------CS--HAF-----HR---AC-ILPWLYKA-------------------------------------------------------------STCPKC    44
TraesCS3A02G459400      CAICHLD------------LETGDEE-----------IVRLP---------------------------------------CFHAHAF-----HT---PC-IKQWFYQQ-------------------------------------------------------------SKCPIC    44
TraesCS2B02G108800      CGICLSE--------------EQRAT----------IQGVLN---------------------------------------CCS-HYF-----CF---AC-ILEWSKVE-------------------------------------------------------------SRCPLC    42
TraesCS2D02G092600      CGICLSE--------------EQRAT----------IQGVLN---------------------------------------CCS-HYF-----CF---AC-ILEWSKVE-------------------------------------------------------------SRCPLC    42
TraesCS2A02G094000      CGICLSE--------------EQRAT----------IQGVLN---------------------------------------CCS-HYF-----CF---AC-ILEWSKVE-------------------------------------------------------------SRCPLC    42
TraesCS4A02G117900      CGICLSE--------------DQRTT----------LQGVLN---------------------------------------CCS-HYF-----CF---AC-IMEWSKVE-------------------------------------------------------------SRCPLC    42
TraesCS4B02G186500      CGICLSE--------------DQRTT----------LQGVLN---------------------------------------CCS-HYF-----CF---AC-IMEWSKVE-------------------------------------------------------------SRCPLC    42
TraesCS4D02G187800      CGICLSE--------------DQRTT----------LQGVLN---------------------------------------CCS-HYF-----CF---AC-IMEWSNVE-------------------------------------------------------------SRCPLC    42
TraesCS7A02G212900      CGICFTD--------------SRRA-----------IRGELD---------------------------------------CCA-HHF-----CF---VC-IMAWARVE-------------------------------------------------------------SRCPFC    41
TraesCS7D02G214800      CGICFTD--------------SRRA-----------IRGELD---------------------------------------CCA-HHF-----CF---VC-IMAWARVE-------------------------------------------------------------SRCPFC    41
TraesCS4A02G118100      CGICRMAFDGCCPDC-----KFPGDD-----------CPIIWGA-------------------------------------CN--HAF-----HL---HC-ILKWVNSQTS----------------------------------------------------------TPLCPMC    54
TraesCS4D02G187600      CGICRMAFDGCCPDC-----KFPGDD-----------CPIIWGA-------------------------------------CN--HAF-----HL---HC-ILKWVNSQTS----------------------------------------------------------IPLCPMC    54
TraesCS5A02G476600      CGICRMAFDGCCPDC-----KFPGDE-----------CPIIWGA-------------------------------------CN--HAF-----HL---HC-ILKWVNSQTS----------------------------------------------------------TALCPMC    54
TraesCS5B02G489500      CGICRMAFDGCCPDC-----KFPGDE-----------CPIIWGA-------------------------------------CN--HAF-----HL---HC-ILKWVNSQTS----------------------------------------------------------TPLCPMC    54
TraesCS5D02G489900      CGICRMAFDGCCPDC-----KFPGDE-----------CPIIWGA-------------------------------------CN--HAF-----HL---HC-ILKWVNSQTS----------------------------------------------------------TPLCPMC    54
TraesCS6A02G145300      CPVCLERLD-----------QDPGG--------------ILTTI-------------------------------------CN--HSF-----HC---SC-ISKWTDSS---------------------------------------------------------------CPVC    40
TraesCS6B02G173500      CPVCLERLD-----------QDPGG--------------ILTTI-------------------------------------CN--HSF-----HC---SC-ISKWTDSS---------------------------------------------------------------CPVC    40
TraesCS6D02G134300      CPVCLERLD-----------QDPGG--------------ILTTI-------------------------------------CN--HSF-----HC---SC-ISKWTDSS---------------------------------------------------------------CPVC    40
TraesCS6D02G254700      CAICRNHIMDLCIECQANQASATSEE-----------CTVAWGV-------------------------------------CN--HAF-----HF---HC-ISRWLKTR-------------------------------------------------------------QVCPLD    56
TraesCSU02G056200       CAICRNHIMDLCIECQANQASATSEE-----------CTVAWGV-------------------------------------CN--HAF-----HF---HC-ISRWLKTR-------------------------------------------------------------QVCPLD    56
TraesCS6B02G301900      CAICRNHIMDLCIECQANQASATSEE-----------CTVAWGV-------------------------------------CN--HAF-----HF---HC-ISRWLKTR-------------------------------------------------------------QVCPLD    56
TraesCS6A02G274400      CAICRNHIMDLCIECQANQASATSEE-----------CTVAWGV-------------------------------------CN--HAF-----HF---HC-ISRWLKTR-------------------------------------------------------------QVCPLD    56
TraesCS3B02G000800      CAICRNHIMDLCIECQANQASATSEE-----------CTVAWGV-------------------------------------CN--HAF-----HF---HC-ISRWLKTR-------------------------------------------------------------QVCPLD    56
TraesCS3A02G001800      CAICRNHIMDLCIECQANQASATSEE-----------CTVAWGV-------------------------------------CN--HAF-----HF---HC-ISRWLKTR-------------------------------------------------------------QVCPLD    56
TraesCS1B02G000900      CPICLEEFKV-------------G-----------NEVRGLP---------------------------------------CA--HNF-----HV---EC-IDQWLR-------------LN------------------------------------------------VKCPRC    41
TraesCS1D02G004000      CPICLEEFKV-------------G-----------NEVRGLP---------------------------------------CA--HNF-----HV---EC-IDQWLR-------------LN------------------------------------------------VKCPRC    41
TraesCS1A02G002000      CPICLEEFKV-------------G-----------NEVRGLP---------------------------------------CA--HNF-----HV---EC-IDQWLR-------------LN------------------------------------------------VKCPRC    41
TraesCS2B02G402300      CAVCLENPSF-------------G-----------DTIRTLP---------------------------------------CF--HKF-----HK---EC-IDEWLR-------------RK------------------------------------------------KLCPVC    41
TraesCS2D02G382100      CAVCLENPSF-------------G-----------DTIRTLP---------------------------------------CF--HKF-----HK---EC-IDEWLR-------------RK------------------------------------------------KLCPVC    41
TraesCS2A02G385400      CTVCLENPSF-------------G-----------DTIRTLP---------------------------------------CF--HKF-----HK---EC-IDEWLR-------------RK------------------------------------------------KLCPVC    41
TraesCS3B02G305200      CPICLDCPAD-------------G-----------ASLRHLP---------------------------------------CA--HKF-----HK---EC-INRWLR-------------MR------------------------------------------------TSCPVC    41
TraesCS3D02G271100      CPICLDCPAD-------------G-----------ASLRHLP---------------------------------------CA--HKF-----HK---EC-IDRWLR-------------MR------------------------------------------------TSCPVC    41
TraesCS3A02G271500      CPICLDCPAD-------------G-----------APLRHLP---------------------------------------CA--HKF-----HK---EC-IDRWLR-------------MR------------------------------------------------TSCPVC    41
TraesCS4B02G208600      CSVCLEQVVA-------------G-----------DLLRSLP---------------------------------------CL--HQF-----HV---NC-IDPWLR-------------QQ------------------------------------------------GTCPIC    41
TraesCS4D02G209300      CSVCLEQVVA-------------G-----------DLLRSLP---------------------------------------CL--HRF-----HV---NC-IDPWLR-------------QQ------------------------------------------------GTCPIC    41
TraesCS1A02G118800      CCICLARYSN-------------N-----------DDLRELP---------------------------------------CT--HFF-----HK---EC-VDKWLK-------------IN------------------------------------------------ALCPLC    41
TraesCS1B02G138300      CCICLARYSN-------------N-----------DDLRELP---------------------------------------CT--HFF-----HK---EC-VDKWLK-------------IN------------------------------------------------ALCPLC    41
TraesCS1D02G119700      CCICLARYSN-------------N-----------DDLRELP---------------------------------------CT--HFF-----HK---EC-VDKWLK-------------IN------------------------------------------------ALCPLC    41
TraesCS6B02G379400      CCICLAKYAH-------------N-----------EELRELP---------------------------------------CT--HCF-----HK---EC-VDKWLK-------------IN------------------------------------------------ALCPLC    41
                        1.......10........20........30........40........50........60........70........80........90.......100.......110.......120.......130.......140.......150.......160.......170......
```

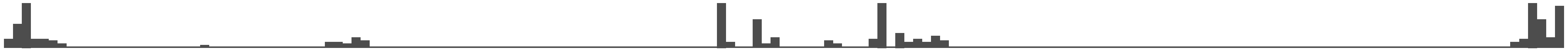

```
                    *  *                                                                             *                 *                                                                        *
TraesCS6D02G328700  CCICLAKYAH-------------N-----------EELRELP---------------------------------------CT--HCF-----HK---EC-VDKWLK-------------IN------------------------------------------------ALCPLC     41
TraesCS6B02G378600  CCICLAKYAH-------------N-----------EELRELP---------------------------------------CT--HCF-----HK---EC-VDKWLK-------------IN------------------------------------------------ALCPLC     41
TraesCS6A02G346000  CCICLAKYAH-------------N-----------EELRELP---------------------------------------CT--HCF-----HK---EC-VDKWLK-------------IN------------------------------------------------ALCPLC     41
TraesCS3B02G139600  CCICLSRFSN-------------N-----------EDLRELP---------------------------------------CG--HVF-----HM---EC-IDKWLK-------------IN------------------------------------------------ALCPLC     41
TraesCS3D02G122200  CCICLSRFSN-------------N-----------EDLRELP---------------------------------------CG--HVF-----HM---EC-IDKWLK-------------IN------------------------------------------------ALCPLC     41
TraesCS3A02G120100  CCICLSRFSN-------------N-----------EDLRELP---------------------------------------CG--HVF-----HM---EC-IDKWLK-------------IN------------------------------------------------ALCPLC     41
TraesCS5A02G183200  CCICLTKYGD-------------D-----------DELRELP---------------------------------------CN--HLF-----HV---QC-VDKWLK-------------IN------------------------------------------------AVCPLC     41
TraesCS5B02G181400  CCICLTKYGD-------------D-----------DELRELP---------------------------------------CN--HLF-----HV---QC-VDKWLK-------------IN------------------------------------------------AVCPLC     41
TraesCS5D02G188300  CCICLTKYGD-------------D-----------DELRELP---------------------------------------CN--HLF-----HV---QC-VDKWLK-------------IN------------------------------------------------AVCPLC     41
TraesCS2B02G419400  CCICISAYDD-------------G-----------AELRELP---------------------------------------CG--HHF-----HC---AC-IDKWLH-------------IN------------------------------------------------ATCPLC     41
TraesCS2D02G398700  CCICISAYDD-------------G-----------AELRELP---------------------------------------CG--HHF-----HC---AC-IDKWLH-------------IN------------------------------------------------ATCPLC     41
TraesCS2A02G401400  CCICISAYDD-------------G-----------AELRELP---------------------------------------CG--HHF-----HC---AC-IDKWLH-------------IN------------------------------------------------ATCPLC     41
TraesCS6B02G269900  CCICLSVYDD-------------G-----------AELRELP---------------------------------------CG--HHF-----HC---AC-IDKWLH-------------IN------------------------------------------------ATCPLC     41
TraesCS6D02G236900  CCICLSVYDD-------------G-----------AELRELP---------------------------------------CG--HHF-----HC---AC-IDKWLH-------------IN------------------------------------------------ATCPLC     41
TraesCS6A02G255700  CCICLSVYDD-------------G-----------AELRELP---------------------------------------CG--HHF-----HC---AC-IDKWLH-------------IN------------------------------------------------ATCPLC     41
TraesCS6B02G327800  CCICLSSYED-------------G-----------VELSALP---------------------------------------CN--HHF-----HS---TC-ITKWLR-------------MH------------------------------------------------ANCPLC     41
TraesCS6D02G278100  CCICLSSYED-------------G-----------VELSALP---------------------------------------CN--HHF-----HS---TC-ITKWLR-------------MH------------------------------------------------ANCPLC     41
TraesCS6A02G298300  CCICLSSYED-------------G-----------VELSALP---------------------------------------CN--HHF-----HS---TC-ITKWLR-------------MH------------------------------------------------ANCPLC     41
TraesCS3B02G286400  CCICLAQYRE-------------K-----------EEVRQLP---------------------------------------CT--HMF-----HL---KC-VDRWLR-------------II------------------------------------------------SSCPLC     41
TraesCS3D02G255300  CCICLAQYRE-------------K-----------EEVRQLP---------------------------------------CT--HMF-----HL---KC-VDRWLR-------------II------------------------------------------------SSCPLC     41
TraesCS3A02G254400  CCICLAQYRE-------------K-----------EEVRQLP---------------------------------------CT--HMF-----HL---KC-VDRWLR-------------II------------------------------------------------SSCPLC     41
TraesCS1B02G129600  CIICLEEYKH-------------K-----------DSLGKLK---------------------------------------CG--HDF-----HA---GC-IKKWLE-------------VK------------------------------------------------NACPVC     41
TraesCS1D02G111500  CIICLEEYKH-------------K-----------DSLGKLK---------------------------------------CG--HDF-----HA---GC-IKKWLE-------------VK------------------------------------------------NACPVC     41
TraesCS1A02G107600  CIICLEEYKH-------------K-----------DSLGKLK---------------------------------------CG--HDF-----HA---GC-IKKWLE-------------VK------------------------------------------------NACPVC     41
TraesCS3A02G103000  CLVCLEEYKD-------------N-----------DLLGILK---------------------------------------CR--HDF-----HT---DC-IKKWLQ-------------VK------------------------------------------------NACPVC     41
TraesCS3B02G120600  CLVCLEEYKN-------------N-----------DLLGILK---------------------------------------CR--HDF-----HT---DC-IKKWLQ-------------VK------------------------------------------------NACPVC     41
TraesCS3D02G105300  CLVCLEEYKD-------------N-----------ELLGILK---------------------------------------CR--HDF-----HT---DC-IKKWLQ-------------VK------------------------------------------------NACPVC     41
TraesCS1A02G373200  CSICQEEFEA-------------S-----------EEIGRLS---------------------------------------CG--HGY-----HV---HC-IKQWLS-------------RK------------------------------------------------NACPLC     41
TraesCS1B02G393400  CSICQEEFEA-------------S-----------EEIGKLS---------------------------------------CG--HGY-----HV---HC-IKQWLS-------------RK------------------------------------------------NACPLC     41
TraesCS1D02G380000  CSICQEEFEA-------------S-----------EEVGRLS---------------------------------------CG--HGY-----HV---HC-IKQWLS-------------RK------------------------------------------------NACPLC     41
TraesCS3A02G260500  CSICQEEFEA-------------N-----------DEMGRLG---------------------------------------CG--HSY-----HV---YC-IKQWLS-------------QK------------------------------------------------NVCPVC     41
TraesCS3D02G260700  CSICQEEFEA-------------N-----------DEMGRLG---------------------------------------CG--HSY-----HV---YC-IKQWLS-------------QK------------------------------------------------NVCPVC     41
TraesCS3B02G293600  CSICQEEFEA-------------N-----------DELGRLG---------------------------------------CG--HSY-----HV---YC-IKQWLS-------------QK------------------------------------------------NVCPVC     41
TraesCS1A02G398300  CIICQEEFEA-------------R-----------ELVGTLD---------------------------------------CG--HKY-----HE---AC-IKQWLM-------------VK------------------------------------------------NLCPIC     41
TraesCS1D02G405800  CIICQEEFEA-------------R-----------DLVGTLD---------------------------------------CG--HKY-----HE---AC-IKQWLM-------------VK------------------------------------------------NLCPIC     41
TraesCS3B02G272400  CIICQEEYEA-------------E-----------DCVGILD---------------------------------------CG--HRY-----HA---EC-LKQWLT-------------VK------------------------------------------------NLCPIC     41
TraesCS3D02G244600  CIICQEEYKA-------------E-----------DCVGILD---------------------------------------CG--HRY-----HA---EC-LKQWLT-------------VK------------------------------------------------NLCPIC     41
TraesCS3A02G246900  CIICQEEYEA-------------E-----------DCVGILD---------------------------------------CG--HRY-----HA---VC-LKQWLT-------------VK------------------------------------------------NLCPIC     41
TraesCS2A02G437200  CSICQEEYSG-------------G-----------EEVGNMA---------------------------------------CK--HYY-----HI---TC-IQHWLR-------------QK------------------------------------------------NWCPIC     41
TraesCS2D02G432800  CSICQEEYSG-------------G-----------EEVGNMA---------------------------------------CK--HYY-----HI---TC-IQHWLR-------------QK------------------------------------------------NWCPIC     41
TraesCS2B02G456100  CSICQEEYSG-------------G-----------EEVGNMA---------------------------------------CK--HYY-----HI---AC-IQHWLR-------------QK------------------------------------------------NWCPIC     41
TraesCS7A02G428300  CSICQEEYIE-------------D-----------EEVGRMK---------------------------------------CE--HQY-----HV---CC-IQEWLR-------------QK------------------------------------------------NWCPIC     41
TraesCS7B02G328500  CSICQEEYIE-------------D-----------EEVGRMK---------------------------------------CE--HQY-----HV---CC-IQEWLR-------------QK------------------------------------------------NWCPIC     41
TraesCS7D02G420400  CSICQEEYIE-------------D-----------EEVGRMK---------------------------------------CE--HQY-----HV---CC-IQEWLR-------------QK------------------------------------------------NWCPIC     41
TraesCS6A02G121400  CSICQEEFVK-------------G-----------EEVGRLR---------------------------------------CE--HQY-----HV---CC-IRQWLL-------------QK------------------------------------------------NWCPVC     41
TraesCS6D02G111500  CSICQEEFVK-------------G-----------EEVGRLR---------------------------------------CE--HQY-----HV---CC-IRQWLL-------------QK------------------------------------------------NWCPVC     41
TraesCS6B02G149700  CSICQEEFVK-------------S-----------EEVGRLP---------------------------------------CE--HQY-----HV---CC-IRQWLL-------------QK------------------------------------------------NWCPVC     41
TraesCS2A02G022100  CCICQEDYAD-------------G-----------EDMGRVE---------------------------------------CG--HYF-----HT---AC-IKQWLV-------------IK------------------------------------------------NTCPIC     41
TraesCS2B02G030700  CCICQEDYAD-------------G-----------EDMGRVE---------------------------------------CG--HYF-----HT---AC-IKQWLV-------------IK------------------------------------------------NTCPIC     41
TraesCS2D02G022900  CCICQEDYAD-------------G-----------EDMGRVE---------------------------------------CG--HYF-----HT---AC-IKQWLV-------------IK------------------------------------------------NTCPIC     41
TraesCS2B02G542200  CCICQEEYVD-------------G-----------DDLGTLH---------------------------------------CG--HDF-----HA---SC-IRQWLV-------------VK------------------------------------------------NLCPIC     41
TraesCS2D02G516000  CCICQEEYVD-------------G-----------DDLGTLH---------------------------------------CG--HDF-----HA---SC-IRQWLV-------------VK------------------------------------------------NLCPIC     41
TraesCS2A02G514400  CCICQEEYVD-------------G-----------DDLGTLH---------------------------------------CG--HDF-----HA---SC-IRQWLV-------------VK------------------------------------------------NLCPIC     41
TraesCS2A02G400200  CVICCMAYKN-------------R-----------DKLTTLP---------------------------------------CQ--HQY-----HR---TC-VAKWLK-------------IN------------------------------------------------KVCPVC     41
TraesCS2B02G418300  CVICCMAYKN-------------R-----------DKLTTLP---------------------------------------CQ--HQY-----HR---TC-VAKWLK-------------IN------------------------------------------------KVCPVC     41
TraesCS2D02G397800  CVICCMAYKN-------------R-----------DKLTTLP---------------------------------------CQ--HQY-----HR---TC-VAKWLK-------------IN------------------------------------------------KVCPVC     41
TraesCS4B02G191300  CVICQMEYRR-------------G-----------DLQMALP---------------------------------------CK--HVY-----HA---SC-VTKWLT-------------IN------------------------------------------------KVCPVC     41
TraesCS4D02G192500  CVICQMEYRR-------------G-----------DLQMALP---------------------------------------CK--HVY-----HA---SC-VTKWLT-------------IN------------------------------------------------KVCPVC     41
TraesCS4A02G112900  CVICQMEYRR-------------G-----------DLQMALP---------------------------------------CK--HVY-----HA---SC-VTKWLT-------------IN------------------------------------------------KVCPVC     41
TraesCS5B02G313800  CVICKSNYKS-------------L-----------QKLIRLP---------------------------------------CS--HCY-----HA---GC-ITRWLK-------------IN------------------------------------------------KACPVC     41
TraesCS5D02G319600  CVICKSNYKS-------------L-----------QKLIRLP---------------------------------------CS--HCY-----HA---GC-ITRWLK-------------IN------------------------------------------------KACPVC     41
TraesCS5A02G312800  CVICKSSYKS-------------L-----------QKLIRLP---------------------------------------CS--HCY-----HA---NC-ITRWLK-------------IN------------------------------------------------KACPVC     41
TraesCS7A02G005100  CAICKSNFKS-------------Q-----------QKLIRLP---------------------------------------CS--HSY-----HA---DC-ITRWLK-------------IN------------------------------------------------KSCPVC     41
TraesCS4A02G441400  CTVCLSAFRN-------------R-----------ENIITLP---------------------------------------CK--HNY-----HA---SC-ISSWLK-------------IN------------------------------------------------KTCPVC     41
TraesCS7D02G049800  CTVCLSAFRN-------------R-----------ENIITLP---------------------------------------CK--HNY-----HA---SC-ISSWLK-------------IN------------------------------------------------RTCPVC     41
TraesCS5B02G325800  CVICRVEFEE-------------G-----------ESLVALP---------------------------------------CN--HSY-----HP---DC-INQWLQ-------------IN------------------------------------------------KVCPMC     41
TraesCS5D02G331600  CVICRVEFEE-------------G-----------ESLVALP---------------------------------------CN--HSY-----HP---DC-INQWLQ-------------IN------------------------------------------------KVCPMC     41
TraesCS5A02G325400  CVICRVEFEE-------------G-----------ESLVALP---------------------------------------CN--HSY-----HP---DC-INQWLQ-------------IN------------------------------------------------KVCPMC     41
TraesCS7A02G271500  CVICRVEFEE-------------G-----------ESLVALP---------------------------------------CK--HSY-----HS---DC-IKQWLQ-------------LN------------------------------------------------KVCPMC     41
TraesCS7D02G271800  CVICRVEFEE-------------G-----------ESLVALP---------------------------------------CK--HSY-----HS---DC-INQWLQ-------------LN------------------------------------------------KVCPMC     41
TraesCS2B02G566700  CAVCLKDFEV-------------D-----------DKVTTMP---------------------------------------CD--HYF-----HQ---GC-ISEWLK-------------VS------------------------------------------------CVCPLC     41
TraesCS2D02G537500  CAVCLKDFEV-------------D-----------DKVTTMP---------------------------------------CD--HYF-----HQ---GC-ISEWLK-------------VS------------------------------------------------CVCPLC     41
TraesCS2B02G566600  CAVCLKDFEV-------------D-----------DKVATMP---------------------------------------CD--HYF-----HQ---GC-ISEWLK-------------VS------------------------------------------------CACPLC     41
TraesCS2D02G522200  CAVCLKDFKV-------------D-----------DKVTTMP---------------------------------------CD--HYF-----HQ---GC-IYEWLM-------------VS------------------------------------------------CVCPLC     41
TraesCS4A02G185500  CAVCLEDLEG-------------GDD---------DKLRAMP---------------------------------------CS--HCF-----HQ---RC-IFRWLM-------------VS------------------------------------------------RVCPCC     43
                    1.......10........20........30........40........50........60........70........80........90........100.......110.......120.......130.......140.......150.......160.......170.....
```

CLUSTAL 2.1 MULTIPLE SEQUENCE ALIGNMENT


```
                    *  *                                                                             *                 *                                                                        *
TraesCS4D02G128100  CAVCLEDFEG-------------GGD---------DKLRAMP---------------------------------------CS--HCF-----HQ---RC-IFRWLS-------------VS------------------------------------------------RVCPCC   43
TraesCS4D02G362000  CAVCLEDFQD-------------G-----------DELRTMP---------------------------------------CS--HSF-----HR---CC-IFRWLE-------------IS------------------------------------------------RVCPYC   41
TraesCS6B02G164200  CHVCHDDFEE-------------G-----------DKLRAMP---------------------------------------CA--HSF-----HQ---RC-IFRALS-------------VN------------------------------------------------RLCPVC   41
TraesCS4A02G185700  CVVCLESFKD-------------G-----------DDIRKLP---------------------------------------CS--HGF-----HN---SC-ISDWLR-------------VS------------------------------------------------CLCPQC   41
TraesCS4D02G127900  CVVCLESFKE-------------G-----------DDIRKLP---------------------------------------CS--HGF-----HN---SC-ISDWLR-------------VS------------------------------------------------CLCPQC   41
TraesCS5B02G049900  CSVCMEAFEE-------------G-----------EALRRMH---------------------------------------CS--HAF-----HE---DC-ISMWLS-------------VS------------------------------------------------HLCPLC   41
TraesCS5D02G055800  CSVCMEAFEE-------------G-----------EMLRGLE---------------------------------------CS--HAF-----HE---DC-ISVWLS-------------VS------------------------------------------------HLCPLC   41
TraesCS5A02G045900  CSVCMEAFEE-------------E-----------DILRRLH---------------------------------------CS--HAF-----HE---EC-ISVWLG-------------VS------------------------------------------------HLCPLC   41
TraesCS1A02G029800  CAVCKEPFEL-------------G-----------AEAREMP---------------------------------------CA--HIY-----HE---DC-ILPWLQ------------LRN------------------------------------------------S-CPVC   41
TraesCS1D02G030300  CAVCKEPFEL-------------G-----------AEAREMP---------------------------------------CA--HIY-----HE---DC-ILPWLQ------------LRN------------------------------------------------S-CPVC   41
TraesCS1B02G036900  CAVCKEPFEL-------------G-----------ADAREMP---------------------------------------CA--HIY-----HE---DC-ILPWLQ------------LRN------------------------------------------------S-CPVC   41
TraesCS3B02G595200  CAVCKEAFEL-------------G-----------DEAREMP---------------------------------------CG--HMY-----HQ---DC-ILPWLA------------LRN------------------------------------------------S-CPVC   41
TraesCS3D02G532300  CAVCKEAFQL-------------G-----------DEAREMP---------------------------------------CS--HMY-----HQ---DC-ILPWLA------------LRN------------------------------------------------S-CPVC   41
TraesCS4A02G095800  CAVCQEAFEP-------------G-----------AAAREMP---------------------------------------CK--HVY-----HQ---DC-ILPWLS------------LRN------------------------------------------------S-CPIC   41
TraesCS4B02G209100  CAVCQEAFEP-------------G-----------AAAREMP---------------------------------------CK--HVY-----HQ---DC-ILPWLS------------LRN------------------------------------------------S-CPIC   41
TraesCS4D02G209500  CAVCQEAFEP-------------G-----------AAAREMP---------------------------------------CK--HVY-----HQ---DC-ILPWLS------------LRN------------------------------------------------S-CPIC   41
TraesCS1A02G162400  CAVCKEDFSP-------------G-----------DGAKQMP---------------------------------------CK--HIY-----HA---DC-IVPWLE------------LHN------------------------------------------------S-CPIC   41
TraesCS1B02G179000  CAVCKEDFSP-------------G-----------DGAKQMP---------------------------------------CK--HIY-----HA---DC-IVPWLE------------LHN------------------------------------------------S-CPIC   41
TraesCS1D02G159200  CAVCKEDFSP-------------G-----------DGAKQMP---------------------------------------CK--HIY-----HA---DC-IVPWLE------------LHN------------------------------------------------S-CPIC   41
TraesCS3B02G361000  CPVCTDRFEV-------------G-----------SEAREMP---------------------------------------CK--HLY-----HA---SC-IIPWLV------------QHN------------------------------------------------S-CPVC   41
TraesCS3D02G324300  CPVCTDRFEV-------------G-----------SEAREMP---------------------------------------CK--HLY-----HA---SC-IIPWLV------------QHN------------------------------------------------S-CPVC   41
TraesCS3A02G330900  CPVCTERFEI-------------G-----------SEAREMP---------------------------------------CK--HLY-----HA---SC-IIPWLV------------QHN------------------------------------------------S-CPVC   41
TraesCS3A02G440000  CPVCKDKFEL-------------G-----------SEAREMP---------------------------------------CK--HLY-----HS---DC-ILPWLE------------QHN------------------------------------------------S-CPVC   41
TraesCS3D02G432800  CPVCKDKFEL-------------G-----------SEAREMP---------------------------------------CK--HLY-----HS---DC-ILPWLE------------QHN------------------------------------------------S-CPVC   41
TraesCS3B02G474100  CPVCKDKFEL-------------E-----------SEAREMP---------------------------------------CK--HLY-----HS---DC-ILPWLE------------QHN------------------------------------------------S-CPVC   41
TraesCS6A02G330700  CPVCKEEFEL-------------G-----------EAARQLP---------------------------------------CK--HVY-----HS---DC-IVPWLR------------LHN------------------------------------------------S-CPVC   41
TraesCS6D02G309800  CPVCKEEFEL-------------G-----------EAARQLP---------------------------------------CK--HVY-----HS---DC-IVPWLR------------LHN------------------------------------------------S-CPVC   41
TraesCS6B02G361600  CPVCKEEFEL-------------G-----------EAARQLP---------------------------------------CK--HVY-----HS---DC-IVPWLC------------LHN------------------------------------------------S-CPVC   41
TraesCS7A02G169600  CPVCKDEFEL-------------G-----------ETARELP---------------------------------------CK--HAY-----HS---DC-IVPWLQ------------LHN------------------------------------------------S-CPVC   41
TraesCS7D02G170800  CPVCKDEFEL-------------G-----------ETARELP---------------------------------------CK--HAY-----HS---DC-IVPWLR------------LHN------------------------------------------------S-CPVC   41
TraesCS7B02G074800  CPVCKDEFEI-------------G-----------ETARELP---------------------------------------CK--HAY-----HS---DC-IVPWLR------------LHN------------------------------------------------S-CPVC   41
TraesCS1B02G316300  CSVCLDDLDI-------------G-----------SQAKQLP---------------------------------------CE--HKF-----HS---PC-ILPWLE------------LHS------------------------------------------------S-CPVC   41
TraesCS1D02G305300  CSVCLDDLDI-------------G-----------SQAKQLP---------------------------------------CE--HKF-----HS---PC-ILPWLE------------LHS------------------------------------------------S-CPVC   41
TraesCS4A02G499200  CAVCMDDFLL-------------G-----------AAAKQLP---------------------------------------CN--HVF-----HK---DC-ILPWLD------------LHS------------------------------------------------S-CPVC   41
TraesCS7D02G000400  CAVCMDDFLL-------------G-----------AAAKQLP---------------------------------------CN--HVF-----HK---DC-ILPWLD------------LHS------------------------------------------------S-CPVC   41
TraesCS4A02G129800  CPVCLDEFAA-------------G-----------GEAKEMP---------------------------------------CK--HRF-----HD---VC-LLPWLE------------ANS------------------------------------------------S-CPVC   41
TraesCS4D02G176700  CPVCLDDFAA-------------G-----------GEAKEMP---------------------------------------CK--HRF-----HD---VC-LLPWLE------------ANS------------------------------------------------S-CPVC   41
TraesCS4B02G174800  CPVCLDEFAA-------------G-----------GEAKEMP---------------------------------------CK--HRF-----HD---VC-ILPWLE------------AHS------------------------------------------------S-CPVC   41
TraesCS5B02G480100  CTVCLEEFEM-------------G-----------TEAKEMP---------------------------------------CQ--HKF-----HS---NC-ILPWLE------------LHS------------------------------------------------S-CPIC   41
TraesCS5D02G480800  CTVCLEEFEM-------------G-----------TEAKEMP---------------------------------------CQ--HKF-----HS---NC-ILPWLE------------LHS------------------------------------------------S-CPIC   41
TraesCS5A02G467700  CTVCLEEFEM-------------G-----------TEAKEMP---------------------------------------CQ--HKF-----HS---NC-ILPWLE------------LHS------------------------------------------------S-CPIC   41
TraesCS3B02G218400  CAVCLEDYAS-------------G-----------ERARELP---------------------------------------CR--HRF-----HS---QC-IVPWLE------------MHS------------------------------------------------S-CPVC   41
TraesCS3D02G192700  CAVCLEDYAC-------------G-----------ERARELP---------------------------------------CR--HRF-----HS---QC-IVPWLE------------MHS------------------------------------------------S-CPVC   41
TraesCS3A02G189200  CAVCLEDYAS-------------G-----------ERARELP---------------------------------------CR--HRF-----HS---QC-IVPWLD------------MHS------------------------------------------------S-CPVC   41
TraesCS5B02G144700  CPVCKDPMPI-------------R-----------TRAKQLP---------------------------------------CM--HLY-----HS---SC-ILPWLS------------SRN------------------------------------------------T-CPVC   41
TraesCS5D02G146400  CPVCKDPMPI-------------R-----------TRAKQLP---------------------------------------CM--HLY-----HL---SC-ILPWLS------------SRN------------------------------------------------T-CPVC   41
TraesCS5A02G145800  CPVCKDPMPL-------------R-----------TRAKQLP---------------------------------------CM--HLY-----HS---SC-ILPWLS------------SRN------------------------------------------------T-CPVC   41
TraesCS6A02G288800  CAVCKDGIEA-------------G-----------EGARRLP---------------------------------------CA--HLY-----HD---AC-ILPWLA------------IRN------------------------------------------------T-CPLC   41
TraesCS6B02G318600  CAVCKDGIED-------------G-----------EGARRLP---------------------------------------CA--HLY-----HD---AC-ILPWLA------------IRN------------------------------------------------T-CPLC   41
TraesCS7B02G293600  CAVCKDGIVQ-------------G-----------GLATRLP---------------------------------------CA--HFY-----HG---AC-IGPWLA------------IRN------------------------------------------------S-CPVC   41
TraesCS7D02G387300  CAVCKDGIVQ-------------G-----------ELATRLP---------------------------------------CA--HFY-----HG---AC-IGPWLA------------IRN------------------------------------------------S-CPVC   41
TraesCS7A02G391700  CAVCKDGIVQ-------------G-----------ELATWLP---------------------------------------CA--HFY-----HG---AC-IGPWLA------------IRN------------------------------------------------S-CPVC   41
TraesCS7A02G247700  CSVCKDGVVA-------------G-----------ERVKMMP---------------------------------------CS--HRY-----HE---DC-ILPWLE------------VRN------------------------------------------------S-CPLC   41
TraesCS7B02G144100  CSVCKDGVVA-------------G-----------ERVKMMP---------------------------------------CS--HRY-----HE---DC-ILPWLE------------VRN------------------------------------------------S-CPLC   41
TraesCS7D02G246300  CSVCKDGVVA-------------G-----------ERVKMMP---------------------------------------CS--HRY-----HE---EC-ILPWLE------------VRN------------------------------------------------S-CPLC   41
TraesCS6B02G198800  CAVCKDVFAS-------------G-----------QRVVFLP---------------------------------------CK--HYF-----HG---DC-ILPWLA------------MRS------------------------------------------------T-CPVC   41
TraesCS6D02G160300  CAVCKDVFAS-------------G-----------QRVVFLP---------------------------------------CK--HYF-----HG---DC-ILPWLA------------MRS------------------------------------------------T-CPVC   41
TraesCS5A02G232200  CVVCTDPLPP-------------S-----------APALRLP---------------------------------------CG--HLY-----HS---HC-IVRWLS------------EQN------------------------------------------------S-CPIC   41
TraesCS5D02G235800  CVVCTDPLPP-------------S-----------APVRRLP---------------------------------------CG--HLY-----HS---HC-IVRWLS------------EQN------------------------------------------------S-CPIC   41
TraesCS5B02G230500  CVVCTDPLPP-------------S-----------APARRLP---------------------------------------CG--HLY-----HS---HC-IVTWLS------------EHN------------------------------------------------S-CPIC   41
TraesCS7A02G211800  CAICKDDLPL-------------A-----------VAARRLP---------------------------------------CG--HLY-----HS---VC-IVQWLE------------MHN------------------------------------------------S-CPVC   41
TraesCS7B02G118700  CAICKDDLPL-------------A-----------VAARRLP---------------------------------------CG--HLY-----HS---VC-IVQWLE------------MHN------------------------------------------------S-CPVC   41
TraesCS7D02G213400  CAICKDDLPL-------------A-----------VAARRLP---------------------------------------CG--HLY-----HS---VC-IVQWLE------------MHN------------------------------------------------S-CPVC   41
TraesCS5A02G216000  CAVCRENLVV-------------D-----------DKMQELP---------------------------------------CK--HLF-----HP---PC-LKPWLD------------ENN------------------------------------------------S-CPIC   41
TraesCS5D02G223800  CAVCRENLVV-------------D-----------DKMQELP---------------------------------------CK--HLF-----HP---PC-LKPWLD------------ENN------------------------------------------------S-CPIC   41
TraesCS5B02G164100  CAVCTEEIAV-------------A-----------DPVVRLP---------------------------------------CA--HWY-----HA---GC-IAPWLG------------IRS------------------------------------------------T-CPMC   41
TraesCS5D02G171400  CAVCTEEIAV-------------A-----------DPVVRLP---------------------------------------CA--HWY-----HA---GC-IAPWLG------------IRS------------------------------------------------T-CPMC   41
TraesCS5A02G167100  CAVCTEEIAV-------------A-----------DPVVRLP---------------------------------------CA--HWY-----HA---GC-IAPWLG------------IRS------------------------------------------------T-CPMC   41
TraesCS6A02G361600  CAVCLEEFRA-------------G-----------DVLAHLP---------------------------------------CA--HRF-----HW---AC-AVPWVQ------------AAS------------------------------------------------R-CPFC   41
TraesCS6D02G344900  CAVCLEEFRA-------------G-----------DVLAHLP---------------------------------------CA--HRF-----HW---AC-AVPWVQ------------AAS------------------------------------------------R-CPFC   41
TraesCS6B02G395000  CAVCLEEFRG-------------G-----------DVLAHLP---------------------------------------CA--HRF-----HW---AC-AVPWVQ------------AAS------------------------------------------------R-CPFC   41
TraesCS7A02G144900  CAVCLEEFAA-------------G-----------DVLAHLP---------------------------------------CG--HRF-----HW---GC-ALPWLEGA---------AAAS------------------------------------------------HSCPFC   45
TraesCS7D02G146300  CAVCLDEFAA-------------G-----------DVLAHLP---------------------------------------CG--HRF-----HW---GC-ALPWLEGA---------AAAS------------------------------------------------HSCPFC   45
TraesCS7B02G047200  CAVCLDEFAA-------------G-----------DVLAHLP---------------------------------------CG--HRF-----HW---GC-ALPWLEG-----------AAS------------------------------------------------HSCPFC   43
TraesCS5B02G522800  CPICLDELEP-------------G-----------AHAAETP---------------------------------------CQ--HVY-----HP---AC-LATWLG------------ARG------------------------------------------------T-CPVC   41
                    1.......10........20........30........40........50........60........70........80........90.......100.......110.......120.......130.......140.......150.......160.......170......
```

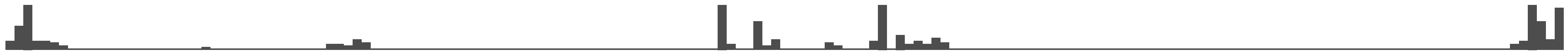

CLUSTAL 2.1 MULTIPLE SEQUENCE ALIGNMENT


```
                    *  *                                                                             *                 *                                                                        *
TraesCS5D02G521500  CPICLDELEP-------------G-----------AHAAETP---------------------------------------CQ--HVY-----HP---AC-LATWLG------------ARG------------------------------------------------T-CPVC      41
TraesCS3B02G394000  CPICLDVLEP-------------G-----------ASAAETP---------------------------------------CQ--HVY-----HP---AC-LAPWLE------------ARG------------------------------------------------T-CPVC      41
TraesCS2A02G168700  CPICLEEYDY-------------E-----------NPKILLQ---------------------------------------CN--HDF-----HL---GC-IYAWME-------------RS------------------------------------------------QSCPVC      41
TraesCS2D02G176300  CPICLEEYDY-------------E-----------NPKILLQ---------------------------------------CN--HDF-----HL---GC-IYAWME-------------RS------------------------------------------------QSCPVC      41
TraesCS2B02G195000  CPICLEEYDY-------------E-----------NPKVLLQ---------------------------------------CN--HDF-----HL---GC-IYEWME-------------RS------------------------------------------------QSCPVC      41
TraesCS3A02G339000  CPICLEEYDY-------------E-----------NPKIVLQ---------------------------------------CN--HNF-----HL---SC-IYEWME-------------RS------------------------------------------------QSCAVC      41
TraesCS3D02G332600  CPICLEEYDY-------------E-----------NPKIVLQ---------------------------------------CN--HNF-----HL---SC-IYEWME-------------RS------------------------------------------------QSCAVC      41
TraesCS3B02G370700  CPICLEEYDY-------------E-----------NPKVVLQ---------------------------------------CN--HNF-----HL---SC-IYEWME-------------RS------------------------------------------------QSCAVC      41
TraesCS3A02G439700  CPTCLEEYTP-------------E-----------NPQIITK---------------------------------------CS--HHF-----HL---SC-IYEWME-------------RS------------------------------------------------DTCPIC      41
TraesCS3B02G473700  CPTCLEEYTP-------------E-----------NPQIITK---------------------------------------CS--HHF-----HL---SC-IYEWME-------------RS------------------------------------------------DTCPIC      41
TraesCS3D02G432500  CPTCLEEYTP-------------E-----------NPQIITK---------------------------------------CS--HHF-----HL---SC-IYEWME-------------RS------------------------------------------------DTCPIC      41
TraesCS4A02G161100  CPTCLEDYTS-------------E-----------NPRIVMQ---------------------------------------CS--HHF-----HL---GC-IYEWME-------------RS------------------------------------------------EACPVC      41
TraesCS4D02G148400  CPTCLEDYTS-------------E-----------NPRIVMQ---------------------------------------CS--HHF-----HL---GC-IYEWME-------------RS------------------------------------------------EACPVC      41
TraesCSU02G068200   CPTCLEDYTS-------------E-----------NPRIVMQ---------------------------------------CS--HHF-----HL---GC-IYEWME-------------RS------------------------------------------------EACPVC      41
TraesCS6B02G439800  CPICLEEYDE-------------E-----------NPRSITK---------------------------------------CE--HHF-----HL---CC-ILEWME-------------RS------------------------------------------------ETCPVC      41
TraesCS6D02G383800  CPICLEEYDE-------------E-----------NPRSITK---------------------------------------CE--HHF-----HL---CC-ILEWME-------------RS------------------------------------------------ETCPVC      41
TraesCS6A02G399600  CPICLEEYDE-------------E-----------NPRSITK---------------------------------------CE--HHF-----HL---CC-ILEWIE-------------RS------------------------------------------------ETCPVC      41
TraesCS2A02G248400  CSICLEAFCD-------------S-----------DPSTVTS---------------------------------------CK--HDF-----HL---QC-ILEWCQ-------------RS------------------------------------------------SQCPMC      41
TraesCS2D02G250100  CSICLEAFCD-------------S-----------DPSTVTS---------------------------------------CK--HDF-----HL---QC-ILEWCQ-------------RS------------------------------------------------SQCPMC      41
TraesCS4D02G158400  CSICLEAFCE-------------S-----------DPSTLTG---------------------------------------CK--HEF-----HL---QC-ILEWCQ-------------RS------------------------------------------------SQCPMC      41
TraesCSU02G068700   CSICLEAFCE-------------S-----------DPSTLTG---------------------------------------CK--HEF-----HL---QC-ILEWCQ-------------RS------------------------------------------------SQCPMC      41
TraesCS4A02G165300  CSICLEAFCE-------------S-----------DPSALTG---------------------------------------CK--HEF-----HL---QC-ILEWCQ-------------RS------------------------------------------------SQCPMC      41
TraesCS4B02G344100  CAICLGSMRT-------------GHG---------QALFTAE---------------------------------------CS--HKF-----HF---HC-ITSNVR-----------HGN-------------------------------------------------HICPIC      44
TraesCS4D02G339200  CAICLGSMRT-------------GHG---------QALFTAE---------------------------------------CS--HKF-----HF---HC-ITSNVR-----------HGN-------------------------------------------------HICPIC      44
TraesCS5A02G512800  CAICLGSMRT-------------GHG---------QALFTAE---------------------------------------CS--HKF-----HF---HC-ITSNVR-----------HGN-------------------------------------------------HICPIC      44
TraesCS1A02G146700  CAICLGGMRP-------------GHG---------HALFTAE---------------------------------------CS--HKF-----HF---RC-ISSNVQ-----------HGN-------------------------------------------------RACPIC      44
TraesCS2A02G364100  CAICFDSMRS-------------GHG---------QALFTAE---------------------------------------CS--HKF-----HF---HC-ISSNVK-----------HGN-------------------------------------------------HVCPVC      44
TraesCS2B02G382300  CAICFDSMRS-------------GHG---------QALFTAE---------------------------------------CS--HKF-----HF---HC-ISSNVK-----------HGN-------------------------------------------------HVCPVC      44
TraesCS2D02G361900  CAICFDSMRS-------------GHG---------QALFTAE---------------------------------------CS--HKF-----HF---HC-ISSNVK-----------HGN-------------------------------------------------HVCPVC      44
TraesCS6A02G227300  CAICFDSMKP-------------GHG---------QALFTAE---------------------------------------CS--HMF-----HF---HC-ISSSVK-----------HGN-------------------------------------------------YVCPVC      44
TraesCS6B02G254400  CAICFDSMKP-------------GHG---------QALFTAE---------------------------------------CS--HMF-----HF---HC-ISSSVK-----------HGN-------------------------------------------------YVCPVC      44
TraesCS6D02G207700  CAICFDSMKP-------------GHG---------QALFTAE---------------------------------------CS--HMF-----HF---HC-ISSSVK-----------HGN-------------------------------------------------YVCPVC      44
TraesCS6A02G375500  CAICLTIMKP-------------GQG---------HALFTAE---------------------------------------CS--HTF-----HF---HC-ISANVK-----------HGS-------------------------------------------------NNCPVC      44
TraesCS6B02G413100  CAICLTIMKP-------------GQG---------HALFTAE---------------------------------------CS--HTF-----HF---HC-ISANVK-----------HGS-------------------------------------------------NNCPVC      44
TraesCS6D02G360000  CAICLTIMKP-------------GQG---------HALFTAE---------------------------------------CS--HTF-----HF---HC-ISANVK-----------HGS-------------------------------------------------NNCPVC      44
TraesCS2A02G358400  CAICLGGMGA-------------GGG---------QAIFTAE---------------------------------------CS--HTF-----HF---HC-ISASVE-----------HGN-------------------------------------------------LLCPLC      44
TraesCS2D02G357400  CAICLGGMGA-------------GGG---------QAIFTAE---------------------------------------CS--HTF-----HF---HC-ISTSVE-----------HGN-------------------------------------------------LLCPLC      44
TraesCS2B02G377900  CAICLGGMGA-------------GGE---------QAIFTAE---------------------------------------CS--HTF-----HF---HC-ISASVE-----------HGN-------------------------------------------------LLCPLC      44
TraesCS4B02G344200  CAICLGGMAA-------------GGG---------QATFTAE---------------------------------------CS--HTF-----HF---NC-ISASVA-----------HGH-------------------------------------------------LVCPLC      44
TraesCS4D02G339300  CAICLGGMAA-------------GGG---------QATFTAE---------------------------------------CS--HTF-----HF---NC-ISASVA-----------HGH-------------------------------------------------LVCPLC      44
TraesCS5A02G513000  CAICLGGMAA-------------GGG---------QATFTAE---------------------------------------CS--HTF-----HF---NC-ISASVA-----------HGH-------------------------------------------------LVCPLC      44
TraesCS1B02G456800  CAICLGAMLR-------------G-----------QAGFTAE---------------------------------------CS--HAF-----HL---NC-ISASVA-----------HGN-------------------------------------------------HDCPLC      42
TraesCS1D02G433500  CAICLGGMVR-------------G-----------QAGFTAE---------------------------------------CS--HAF-----HL---RC-ISASVA-----------HGN-------------------------------------------------HDCPLC      42
TraesCS7A02G423300  CAICLGGMVR-------------G-----------QASYTAE---------------------------------------CS--HAF-----HL---IC-ISASVA-----------HGN-------------------------------------------------HTCPLC      42
TraesCS5D02G472700  CAVCHGDMGR-------------G-----------HATFTAE---------------------------------------CS--HSF-----HL---RC-V----------------HHQ-------------------------------------------------IACPLC      37
TraesCS1B02G164800  CAICNGDMRR-------------GGGG--------GSGFTAD---------------------------------------CS--HQF-----HY---RC-VSGSLA-----------SSR-------------------------------------------------QACPLC      45
TraesCS1D02G144900  CAICNGDMRR-------------GGGG--------GSGFTAD---------------------------------------CS--HQF-----HY---RC-VSGSLA-----------SSR-------------------------------------------------QACPLC      45
TraesCS1B02G254700  CYICLVEYEE-------------GDC-----------VRILP---------------------------------------CN--HEF-----HL---TC-VDKWLK-----------EIH-------------------------------------------------RVCPLC      42
TraesCS1D02G243200  CYICLVEYEE-------------GDC-----------VRILP---------------------------------------CN--HEF-----HL---TC-VDKWLK-----------EIH-------------------------------------------------RVCPLC      42
TraesCS1A02G243200  CYICLVEYEE-------------GDC-----------VRILP---------------------------------------CN--HEF-----HL---TC-VDKWLK-----------EIH-------------------------------------------------RVCPLC      42
TraesCS6A02G362000  CHICLTEYED-------------GDQ-----------IRSLP---------------------------------------CK--HEF-----HL---QC-VDKWLK-----------EIH-------------------------------------------------RVCPLC      42
TraesCS6D02G345700  CHICLTEYED-------------GDQ-----------IRSLP---------------------------------------CK--HEF-----HL---QC-VDKWLK-----------EIH-------------------------------------------------RVCPLC      42
TraesCS2B02G625300  CAICLEDYTV-------------GEQ-----------IRVLP---------------------------------------CR--HKF-----HA---AC-VDMWLT-----------SWR-------------------------------------------------SFCPVC      42
TraesCS2D02G574200  CAICLEDYTV-------------GEQ-----------IRVLP---------------------------------------CR--HKF-----HA---AC-VDMWLT-----------SWR-------------------------------------------------SFCPVC      42
TraesCS2A02G564000  CAICLEDYTV-------------GEQ-----------IRVLP---------------------------------------CR--HKF-----HA---AC-VDMWLT-----------SWR-------------------------------------------------SFCPVC      42
TraesCS7B02G226400  CAICLEDYSV-------------GEK-----------IRVLP---------------------------------------CR--HKF-----HA---AC-VDLWLT-----------SWR-------------------------------------------------TFCPVC      42
TraesCS7D02G322500  CAICLEDYSV-------------GEK-----------IRVLP---------------------------------------CR--HKF-----HA---AC-VDLWLT-----------SWR-------------------------------------------------TFCPVC      42
TraesCS7A02G325800  CAICLEDYSV-------------GEK-----------IRVLP---------------------------------------CR--HKF-----HA---AC-VDLWLT-----------SWR-------------------------------------------------TFCPVC      42
TraesCS1B02G156500  CCVCISRFRD-------------GED-----------IRSLP---------------------------------------CG--HAF-----HR---QC-VDRWLA-----------LCR-------------------------------------------------RTCPLC      42
TraesCS1D02G138900  CCVCISRFRD-------------GED-----------IRSLP---------------------------------------CG--HAF-----HR---QC-VDRWLA-----------LCR-------------------------------------------------RTCPLC      42
TraesCS2A02G065200  CCVCLSRIRA-------------GEA-----------TRRLP---------------------------------------CE--HAF-----HR---DC-VDRWLA-----------LCK-------------------------------------------------RTCPLC      42
TraesCS2B02G077400  CCVCLSRIRA-------------GEA-----------TRRLP---------------------------------------CE--HAF-----HR---DC-VDRWLA-----------LCK-------------------------------------------------RTCPLC      42
TraesCS2D02G063300  CCVCLSRIRA-------------GEA-----------TRRLP---------------------------------------CE--HAF-----HR---DC-VDRWLA-----------LCK-------------------------------------------------RTCPLC      42
TraesCS2B02G115500  CAVCLSGVGE-------------GDE-----------VRELR---------------------------------------CR--HVF-----HR---AC-LDRWLA-----------TPP-------------------------------------------------ATCPLC      42
TraesCS2D02G098400  CAVCLSGVAE-------------GDE-----------VRELR---------------------------------------CR--HVF-----HR---AC-LDRWLA-----------TPP-------------------------------------------------ATCPLC      42
TraesCS2A02G099300  CAVCLSGVEE-------------GDE-----------VRELR---------------------------------------CR--HVF-----HR---AC-LDRWLA-----------TPP-------------------------------------------------ATCPLC      42
TraesCS3A02G182100  CVFCLSRIDD-------------GEE-----------VRELR---------------------------------------CR--HVF-----HR---EC-LDSWLL-----------RPR-------------------------------------------------ATCPLC      42
TraesCS3B02G212200  CVFCLSRIDD-------------GEE-----------VRELR---------------------------------------CR--HVF-----HR---EC-LDSWLL-----------RPR-------------------------------------------------ATCPLC      42
TraesCS3D02G186800  CVFCLSRIDD-------------GEE-----------VRELR---------------------------------------CR--HIF-----HR---EC-LDSWLL-----------CPR-------------------------------------------------ATCPLC      42
TraesCS5A02G253700b CRVCLVRFEA-------------DAV-----------VNRLP---------------------------------------CG--HLF-----HR---AC-LETWLD-----------YDH-------------------------------------------------ATCPLC      42
TraesCS5B02G252600  CRVCLVRFEA-------------DAV-----------VNRLP---------------------------------------CG--HLF-----HR---AC-LETWLD-----------YDH-------------------------------------------------ATCPLC      42
TraesCS5A02G253700a CRVCLVRFEA-------------DAV-----------VNRLP---------------------------------------CG--HLF-----HR---AC-LETWLD-----------YDH-------------------------------------------------ATCPLC      42
TraesCS4A02G128700  CVICLGGMEE-------------GDE-----------ARVLR---------------------------------------CQ--HLF-----HR---CC-LDWWLA-----------ARPG------------------------------------------------ATCPLC      43
TraesCS4D02G177900  CVICLGGMEE-------------GDE-----------VRVLR---------------------------------------CQ--HLF-----HR---CC-PDRWLA-----------ARPG------------------------------------------------ATCPLC      43
TraesCS4B02G176000  CVICLGGMEE-------------GDE-----------VRVLR---------------------------------------CH--HLF-----HR---CC-LARWLA-----------AWAG------------------------------------------------ATCPLC      43
                    1.......10........20........30........40........50........60........70........80........90........100.......110.......120.......130.......140.......150.......160.......170......
```

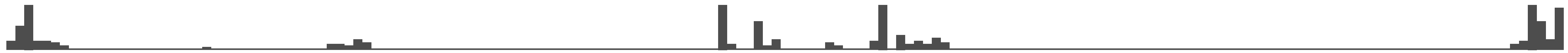

```
                    *  *                                                                             *                 *                                                                        *
TraesCS4B02G176100  CVICLGGMEE-------------GDE-----------VRVLR---------------------------------------CH--HLF-----HR---CC-LARWLA-----------AWAG------------------------------------------------ATCPLC   43
TraesCS2A02G232200  CVICMTTIDL-------------SQRT--------SEYMVAP---------------------------------------CE--HIF-----HS---GC-LQRWMD-------------IK------------------------------------------------MECPTC   44
TraesCS2D02G232900  CVICMTTIDL-------------SQRT--------SEYMVAP---------------------------------------CE--HIF-----HS---GC-LQRWMD-------------IK------------------------------------------------MECPTC   44
TraesCS2B02G250800  CVICMTAIDL-------------SQRT--------SEYMVAP---------------------------------------CE--HIF-----HS---GC-LQRWMD-------------IK------------------------------------------------MECPTC   44
TraesCS4B02G255400  CSICLDGFYD-------------G-----------DELIRLR---------------------------------------CR--HRF-----HS---TC-LEPWVR-------------KC------------------------------------------------ADCPYC   41
TraesCS4D02G255100  CSICLDGFYD-------------G-----------DELIRLR---------------------------------------CG--HRF-----HS---TC-LEPWVR-------------KC------------------------------------------------ADCPYC   41
TraesCS4A02G049500  CSICLDGFYD-------------R-----------DELIRLR---------------------------------------CG--HRF-----HS---TC-LEPWVR-------------KC------------------------------------------------ADCPYC   41
TraesCS5A02G072600  CSICLERCGE-------------A-----------DGLMQLR---------------------------------------CK--HVF-----HS---AC-LERWLR-------------SH------------------------------------------------GDCPYC   41
TraesCS5B02G078400  CSICLERCGE-------------A-----------DGLMQLR---------------------------------------CK--HVF-----HS---AC-LERWLR-------------SH------------------------------------------------GDCPYC   41
TraesCS5D02G085300  CSICLERCGE-------------A-----------DGLMQLR---------------------------------------CK--HVF-----HS---AC-LERWLR-------------SH------------------------------------------------GDCPYC   41
TraesCS4A02G237400  CSICLEGCHG-------------AS----------GGLIQLR---------------------------------------CK--HIF-----HS---AC-LEQWLQ-------------SR------------------------------------------------ADCPYC   42
TraesCS4B02G077700  CSICLEGCHG-------------AS----------DGLIQLR---------------------------------------CK--HIF-----HS---AC-LEQWLQ-------------SR------------------------------------------------ADCPYC   42
TraesCS4D02G076100  CSICLEGCHG-------------AS----------DGLIQLR---------------------------------------CK--HIF-----HL---AC-LEQWLQ-------------SR------------------------------------------------ADCPYC   42
TraesCS3A02G179300  CIICREEM---------------------------TTAKKLL---------------------------------------CG--HLF-----HV---HC-LRSWLER-------------------------------------------------------------QHTCPTC   38
TraesCS3D02G184200  CIICREEM---------------------------TTAKKLL---------------------------------------CG--HLF-----HV---HC-LRSWLER-------------------------------------------------------------QHTCPTC   38
TraesCS1A02G133300  CIICREEM---------------------------TTAKKLL---------------------------------------CG--HLF-----HV---HC-LRSWRER-------------------------------------------------------------QHTCPTC   38
TraesCS7B02G127400  CIICREEM---------------------------TTAKKLL---------------------------------------CG--HLF-----HV---HC-LRSWLQR-------------------------------------------------------------QHTCPTC   38
TraesCS7D02G222100  CIICREEM---------------------------TTAKKLL---------------------------------------CG--HLF-----HV---HC-LRSWLQR-------------------------------------------------------------QHTCPTC   38
TraesCS7A02G220000  CIICREEM---------------------------TTAKKLL---------------------------------------CG--HLF-----HV---HC-LRSWLQR-------------------------------------------------------------QHTCPTC   38
TraesCS6A02G202400  CIICCEET---------------------------TTAKKLL---------------------------------------CG--HLF-----HV---HC-LRSWRER-------------------------------------------------------------QNTCPTC   38
TraesCS2B02G255300  CAICRGPM---------------------------ARAKKLP---------------------------------------CN--HLF-----HL---PC-LRSWLDQG----------LME-----------------------------------------------GYSCPTC   42
TraesCS2D02G235300  CAICRGPM---------------------------ARAKKLP---------------------------------------CN--HLF-----HL---PC-LRSWLDQG----------LME-----------------------------------------------GYSCPTC   42
TraesCS2A02G227400  CAICRGPM---------------------------ARAKKLP---------------------------------------CD--HLF-----HL---PC-LRSWLDQG----------LME-----------------------------------------------GYSCPTC   42
TraesCS2B02G182300  CAICLDAF---------------------------GAGKEMP---------------------------------------CG--HRF-----HG---EC-LERWLGV-------------------------------------------------------------HGSCPVC   38
TraesCS2D02G162600  CAICLDAF---------------------------GAGKEMP---------------------------------------CG--HRF-----HG---EC-LERWLGV-------------------------------------------------------------HGSCPVC   38
TraesCS2A02G156800  CAICLDAF---------------------------GAGKEMP---------------------------------------CG--HRF-----HG---EC-LERWLGV-------------------------------------------------------------HGSCPVC   38
TraesCS1A02G005700b CVFCMKLP---------------------------ERPVTTP---------------------------------------CG--HNF-----CL---KC-FEKWV------------NSGK------------------------------------------------RTCGKC   39
TraesCS1D02G002400b CVFCMKLP---------------------------ERPVTTP---------------------------------------CG--HNF-----CL---KC-FEKWV------------NSGK------------------------------------------------RTCGKC   39
TraesCS1A02G005700c CSICKNLM---------------------------KEPLTTP---------------------------------------CG--HNF-----CK---AC-LTAAFGD--EGSMRERSRGGRTL----------------------------------------RAQKIVKNCPSC   57
TraesCS1B02G007900  CSICKNVM---------------------------KEPLTTP---------------------------------------CG--HNF-----CK---AC-LTAAFGD--EGSMRERSRGGRTL----------------------------------------RAQKIVKNCPSC   57
TraesCS6B02G332400  CAICLDIC---------------------------YQPSTTP---------------------------------------CG--HSF-----CM---QC-LKHAA-----------SKCG-------------------------------------------------KRCPKC   39
TraesCS6D02G283700  CAICLDIC---------------------------YQPSTTP---------------------------------------CG--HSF-----CM---QC-LKHAA-----------SKCG-------------------------------------------------KRCPKC   39
TraesCS6A02G304300  CAICLDVC---------------------------FQPSTTP---------------------------------------CG--HSF-----CM---QC-LKHAA-----------SKCG-------------------------------------------------KRCPKC   39
TraesCS4A02G162000  CTLCFKLL---------------------------FKPVTTP---------------------------------------CG--HTF-----CR---SC-LHQAM------------DHG-------------------------------------------------NKCPMC   38
TraesCS4D02G155700  CTLCFKLL---------------------------FEPVTTP---------------------------------------CG--HTF-----CR---SC-LHQAM------------DHG-------------------------------------------------NKCPMC   38
TraesCS4B02G151200  CTLCFKLL---------------------------FEPVTTP---------------------------------------CG--HTF-----CR---SC-LHQSM------------DHG-------------------------------------------------NKCPMC   38
TraesCS6B02G255500a CCVCLELL---------------------------YKPVVIA---------------------------------------CG--HMS-----CF---WC-VHKAMH----------YARES-------------------------------------------------HCAIC   40
TraesCS6D02G209000a CCVCLELL---------------------------YKPVVIA---------------------------------------CG--HMS-----CF---WC-VHKAMH----------YARES-------------------------------------------------HCAIC   40
TraesCS6A02G226100a CCVCLELL---------------------------YKPVVIA---------------------------------------CG--HMS-----CF---WC-VHKAMH----------YARES-------------------------------------------------HCAIC   40
TraesCS6A02G226100b CPICKELL---------------------------YQPAVLN---------------------------------------CG--HVY-----CI---SC-LPSVG------------DEAL-------------------------------------------------KCQVC   38
TraesCS6B02G255500b CPICKELL---------------------------YQPAVLN---------------------------------------CG--HVY-----CI---SC-LPSVG------------DEAL-------------------------------------------------KCQVC   38
TraesCS6D02G209000b CPICKELL---------------------------YQPTVLN---------------------------------------CG--HVY-----CI---SC-LPSVG------------DEAL-------------------------------------------------KCQVC   38
TraesCS2A02G112800  CSICLDPM---------------------------FDPVSLS---------------------------------------CG--HIF-----CY---LC-CCSAASV--TIVDGLKSADHR------------------------------------------------SKCPLC   49
TraesCS2B02G131600  CSICLDTM---------------------------FDPVSLS---------------------------------------CG--HIF-----CY---LC-CCSAASV--TIVDGLKSADHR------------------------------------------------SKCPLC   49
TraesCS7A02G420500  CSICLDTV---------------------------FDAVSLS---------------------------------------CG--HIF-----CY---LC-CSAAASV--TVVDGLESADPG------------------------------------------------SKCPIC   49
TraesCS7D02G413200  CSICLDTV---------------------------FDAVSLS---------------------------------------CG--HIF-----CY---PC-CSAAASV--TVVDGLESADPG------------------------------------------------SKCPIC   49
TraesCS5B02G373000  CPICLDTL---------------------------FNPYALS---------------------------------------CG--HLF-----CK---GC-ACGAASV--YIFQGVRSAPPE------------------------------------------------AKCPVC   49
TraesCS5D02G380200  CPICLDTL---------------------------FNPYALS---------------------------------------CG--HLF-----CK---GC-ACGAASV--YIFQGVRSAPPE------------------------------------------------AKCPVC   49
TraesCS2A02G556200  CRVCLNNE---------------------------VNMTIVP---------------------------------------CG--HVL-----CN---RC-SSSVS-----------------------------------------------------------------RCPFC   34
TraesCS2D02G557400  CRVCLNNE---------------------------VNMTIVP---------------------------------------CG--HVL-----CN---RC-SSSVS-----------------------------------------------------------------RCPFC   34
TraesCS2B02G586500  CRVCLNSE---------------------------VNMTIVP---------------------------------------CG--HVL-----CN---RC-SSSVS-----------------------------------------------------------------RCPFC   34
TraesCS1A02G198000  CNVCFDMA---------------------------DDPVVTK---------------------------------------CG--HLF-----CW---DC-LYQWLH----------VHSNH------------------------------------------------RECPVC   41
TraesCS1B02G212700  CNVCFDMA---------------------------DDPVVTK---------------------------------------CG--HLF-----CW---DC-LYQWLH----------VHSNH------------------------------------------------RECPVC   41
TraesCS1D02G201500  CNVCFDMA---------------------------DDPVVTK---------------------------------------CG--HLF-----CW---DC-LYQWLH----------VHSNH------------------------------------------------RECPVC   41
TraesCS1A02G296100  CNICFEMA---------------------------DEPVVTS---------------------------------------CG--HLF-----CW---PC-LYQWLH----------IHSTH------------------------------------------------KECPVC   41
TraesCS1B02G304900  CNICFEMA---------------------------DEPVVTS---------------------------------------CG--HLF-----CW---PC-LYQWLH----------IHSTH------------------------------------------------KECPVC   41
TraesCS1D02G296100  CNICFEMA---------------------------DDPVVTS---------------------------------------CG--HLF-----CW---PC-LYQWLH----------IHSTH------------------------------------------------KECPVC   41
TraesCS3A02G350000  CNICFEMA---------------------------GEPVVTS---------------------------------------CG--HLF-----CW---PC-LYQWLN----------VYSNH------------------------------------------------KECPVC   41
TraesCS3D02G344000  CNICFEMA---------------------------GEPVVTS---------------------------------------CG--HLF-----CW---PC-LYQWLN----------VYSNH------------------------------------------------KECPVC   41
TraesCS3B02G382600  CNICFDMA---------------------------GEPVVTS---------------------------------------CG--HLF-----CW---PC-LYQWLN----------VYSNH------------------------------------------------KECPVC   41
TraesCS7A02G296500  CNICLEAA---------------------------KEPVVTP---------------------------------------CG--HLF-----CW---PC-LYQWLH----------GHSAH------------------------------------------------SECPVC   41
TraesCS7B02G189000  CNICLEAA---------------------------KEPVVTP---------------------------------------CG--HLF-----CW---PC-LYQWLH----------GHSAH------------------------------------------------SECPVC   41
TraesCS7D02G293200  CNICLEAA---------------------------KEPVVTP---------------------------------------CG--HLF-----CW---PC-LYQWLH----------GHSAH------------------------------------------------SECPVC   41
TraesCS3B02G336200  CNICLELA---------------------------QDPVVTL---------------------------------------CG--HLF-----CW---PC-LYEWLH----------VHAHF------------------------------------------------PECPVC   41
TraesCS3D02G301500  CNICLELA---------------------------QDPVVTL---------------------------------------CG--HLF-----CW---PC-LYEWLH----------VHAHF------------------------------------------------PECPVC   41
TraesCS4A02G290600  CNICFELP---------------------------QEPIVTL---------------------------------------CG--HLF-----CW---PC-LYRWLH----------MHANT------------------------------------------------PECPVC   41
TraesCS4B02G023600  CNICFELP---------------------------QEPIVTL---------------------------------------CG--HLF-----CW---PC-LYRWLH----------MHANT------------------------------------------------PECPVC   41
TraesCS4D02G021200  CNICFELP---------------------------QEPIVTL---------------------------------------CG--HLF-----CW---PC-LYRWLH----------MHANT------------------------------------------------PECPVC   41
TraesCS5A02G010000  CNICFELP---------------------------QEPIVTL---------------------------------------CG--HLF-----CW---PC-LYKWLH----------IHSHS------------------------------------------------PECPVC   41
TraesCS5B02G007600  CNICFELP---------------------------QEPIVTL---------------------------------------CG--HLF-----CW---PC-LYKWLH----------IHSHS------------------------------------------------PECPVC   41
TraesCS5D02G014800  CNICFELP---------------------------QEPIVTL---------------------------------------CG--HLF-----CW---PC-LYKWLH----------IHSHS------------------------------------------------PECPVC   41
TraesCS2A02G372600  CNICLDFA---------------------------ADPVVTL---------------------------------------CG--HLY-----CW---PC-IYEWLRPSVVSASGANSTSAR------------------------------------------------QQCPVC   51
TraesCS2B02G389600  CNICLDFA---------------------------ADPVVTL---------------------------------------CG--HLY-----CW---PC-IYEWLRPSVVSASGANSTSAR------------------------------------------------QQCPVC   51
TraesCS2D02G368800  CNICLDFA---------------------------ADPVVTL---------------------------------------CG--HLY-----CW---PC-IYEWLRPLVVSASGANSTSAR------------------------------------------------QQCPVC   51
TraesCS6B02G264500  CNICLDFA---------------------------VEPVVTL---------------------------------------CG--HLY-----CW---PC-IYEWLR---RDAAGGTISTAG------------------------------------------------RPCPVC   48
                    1.......10........20........30........40........50........60........70........80........90.......100.......110.......120.......130.......140.......150.......160.......170......
```

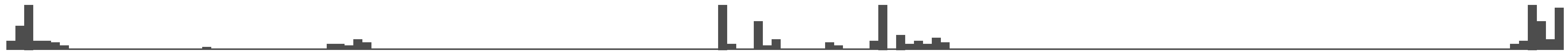

CLUSTAL 2.1 MULTIPLE SEQUENCE ALIGNMENT

File: C:/Users/HP/Downloads/clustal alignBB1.ps **Date: Wed May 20 03:00:39 2020**


```
                    *  *                                                                             *                 *                                                                        *
TraesCS6D02G218600  CNICLDFA---------------------------VEPVVTL---------------------------------------CG--HLY-----CW---PC-IYEWLR---RDAGGGTISTAG------------------------------------------------RPCPVC   48
TraesCS6A02G236100  CNICLDFA---------------------------VEPVVTL---------------------------------------CG--HLY-----CW---PC-IYEWLR---RDAAGGTISAAG------------------------------------------------RPCPVC   48
TraesCS5A02G049400  CSICLETA---------------------------SEPVVTL---------------------------------------CG--HLY-----CW---PC-IFRWLT---TSSRSKSRASSS------------------------------------------------ARCPVC   48
TraesCS5B02G054600  CSICLETA---------------------------TEPVVTL---------------------------------------CG--HLY-----CW---PC-IFRWLT---TSSKSKSRASSS------------------------------------------------ARCPVC   48
TraesCS5D02G060200  CSICLEVA---------------------------SEPVVTL---------------------------------------CG--HLY-----CW---PC-IFRWLT---TSSKSKSRASPS------------------------------------------------ARCPVC   48
TraesCS2A02G145200  CPICLSPP---------------------------STTVITS---------------------------------------CT--HIY-----CQ---TC-ILKILK----------SASS--------------------------------------------------RCPIC   39
TraesCS2D02G148900  CPICLSPP---------------------------STTVITS---------------------------------------CT--HIY-----CQ---PC-ILKILK----------SASS--------------------------------------------------RCPIC   39
TraesCS2B02G170200  CPICLSPP---------------------------STTVITS---------------------------------------CT--HIY-----CQ---TC-ILKILK----------SPSS--------------------------------------------------RCPIC   39
TraesCS5A02G135100  CPICLESP---------------------------LCPQITS---------------------------------------CG--HIY-----CF---PC-ILHYLL----------MGKEDYR----------------------------------------GEC--WKKCPLC   47
TraesCS5B02G133900  CPICLESP---------------------------LCPQITS---------------------------------------CG--HIY-----CF---PC-ILHYLL----------MGKEDYR----------------------------------------GEC--WKKCPLC   47
TraesCS5D02G139400  CPICLESP---------------------------LCPQITS---------------------------------------CG--HIY-----CF---PC-ILHYLL----------MGKEDYR----------------------------------------GEC--WKKCPLC   47
TraesCS3D02G462600  CGVCFDRP---------------------------KEVVITK---------------------------------------CF--HLF-----CS---TC-IQRNLE----------LRHR--------------------------------------------------KCPGC   39
TraesCS3D02G466100  CGVCFDRP---------------------------KEVVITK---------------------------------------CF--HLF-----CS---TC-IQRNLE----------LRHR--------------------------------------------------KCPGC   39
TraesCS3B02G511800  CGVCFDRP---------------------------KEVVITK---------------------------------------CF--HLF-----CS---TC-IQRNLE----------LRHR--------------------------------------------------KCPGC   39
TraesCS3A02G467300  CGVCFDRP---------------------------KEVVITK---------------------------------------CF--HLF-----CS---TC-IQRNLE----------LRHR--------------------------------------------------KCPGC   39
TraesCS3D02G465000  CGVCFDRP---------------------------KEVVIAK---------------------------------------CF--HLF-----CS---TC-IQRNLE----------LRHR--------------------------------------------------KCPGC   39
TraesCS6B02G274100  CGVCRDRQ---------------------------KEVVITK---------------------------------------CY--HLF-----CN---DC-IQKLLR----------NRQR--------------------------------------------------RCPSC   39
TraesCS2A02G224300  CGICHELA---------------------------EDVVVTS---------------------------------------CD--HVF-----CK---TC-LMEYSA------------TLG-----------------------------------------------NVSCPSC   40
TraesCS2B02G274500  CGICHELA---------------------------EDVVVTS---------------------------------------CD--HVF-----CK---TC-LMEYSA------------TLG-----------------------------------------------NVSCPSC   40
TraesCS2D02G230600  CGICHELA---------------------------EDVVVTS---------------------------------------CD--HVF-----CK---TC-LMEYSA------------TLG-----------------------------------------------NVSCPSC   40
TraesCS1B02G074500  CGICHELA---------------------------EDVVVTS---------------------------------------CD--HVF-----CK---SC-LMECSA------------TLG-----------------------------------------------NVSCPSC   40
TraesCS2B02G086700  CPICLESTS--------------------------DDPVITP---------------------------------------CA--HRM-----CR---EC-LLSCWS------------TPA-----------------------------------------------GGACPIC   41
TraesCS2D02G071800  CPICLESTS--------------------------DDPVITP---------------------------------------CA--HRM-----CR---EC-LLSCWS------------TPA-----------------------------------------------GGACPIC   41
TraesCS2A02G072700  CPICLESTS--------------------------DDPVITP---------------------------------------CA--HRM-----CR---EC-LLSSWS------------TPA-----------------------------------------------GGPCPIC   41
TraesCS6A02G117900  CPICLEAF---------------------------EDAVLTP---------------------------------------CA--HRL-----CR---EC-ILSSWQ------------SPA-----------------------------------------------AGLCPVC   40
TraesCS6B02G146100  CPICLEAF---------------------------EDAVLTP---------------------------------------CA--HRL-----CR---EC-ILSSWQ------------SPA-----------------------------------------------AGLCPVC   40
TraesCS6D02G107900  CPICLEAF---------------------------EDAVLTP---------------------------------------CA--HRL-----CR---EC-ILSSWQ------------SPS-----------------------------------------------AGLCPVC   40
TraesCS4A02G082400  CFICREPF---------------------------VDPVVTK---------------------------------------CK--HYF-----CE---HC-ALKHHS------------K-------------------------------------------------NKKCFVC   38
TraesCS4B02G221600  CFICREPF---------------------------VDPVVTK---------------------------------------CK--HYF-----CE---HC-ALKHHS------------K-------------------------------------------------NKKCFVC   38
TraesCS4D02G222000  CFICREPF---------------------------VDPVVTK---------------------------------------CK--HYF-----CE---HC-ALKHHS------------K-------------------------------------------------NKKCFVC   38
TraesCS6B02G296000  CFICREPF---------------------------VDPVVTK---------------------------------------CK--HYF-----CE---HC-ALKHHS------------K-------------------------------------------------NKKCFVC   38
TraesCS3B02G461900  CPVCMNEL---------------------------VNASSTI---------------------------------------CG--HIF-----CQ---KC-IKASIQ------------AQK-------------------------------------------------KCPTC   38
TraesCS3D02G420400  CPVCMNEL---------------------------VNASSTI---------------------------------------CG--HIF-----CQ---KC-IKASIQ------------AQK-------------------------------------------------KCPTC   38
TraesCS3A02G425600  CPVCMNEL---------------------------VNASSTI---------------------------------------CG--HIF-----CQ---KC-IKASIQ------------AQK-------------------------------------------------KCPTC   38
TraesCS3B02G462500  CPVCMNEL---------------------------VDASSTI---------------------------------------CG--HIF-----CQ---KC-IQASIQ------------AQS-------------------------------------------------KCPTC   38
TraesCS3D02G421000  CPVCMNEL---------------------------VDASSTI---------------------------------------CG--HIF-----CQ---KC-IQASIQ------------AQS-------------------------------------------------KCPTC   38
TraesCS3A02G426200  CPVCMNEL---------------------------VDASSTI---------------------------------------CG--HIF-----CQ---KC-IQASIQ------------AQS-------------------------------------------------KCPTC   38
TraesCS3B02G462200  CPVCMNEL---------------------------VDASSTI---------------------------------------CG--HIF-----CQ---KC-IEASIQ------------AQS-------------------------------------------------KCPTC   38
TraesCS3D02G420700  CPVCMNEL---------------------------VDASSTI---------------------------------------CG--HIF-----CQ---KC-IEASIQ------------AQS-------------------------------------------------KCPTC   38
TraesCS3D02G421100  CPVCMNEL---------------------------VDASSTI---------------------------------------CG--HIF-----CQ---NC-IEASIQ------------AQS-------------------------------------------------KCPTC   38
TraesCS3A02G426400  CPVCWNEL---------------------------TDASSTI---------------------------------------CG--HIF-----CQ---KC-IEASIQ------------AQK-------------------------------------------------KCPTC   38
TraesCS3D02G421200  CPVCWNEL---------------------------IDASSTI---------------------------------------CG--HIF-----CQ---KC-IEASIQ------------AQK-------------------------------------------------KCPTC   38
TraesCS3B02G462400  CPVCKNEL---------------------------VDASSTI---------------------------------------CG--HIF-----CQ---KC-IEASIQ------------AQK-------------------------------------------------KCPTC   38
TraesCS3B02G462600  CPVCRNEL---------------------------TDVSSTI---------------------------------------CG--HIF-----CQ---KC-IEASIQ------------AQK-------------------------------------------------KCPTC   38
TraesCS3A02G426100  CPVCINEL---------------------------VDASSTI---------------------------------------CG--HIF-----CK---KC-IEASIR------------FQK-------------------------------------------------KCPTC   38
TraesCS3D02G420800  CPVCINEL---------------------------VDASSTI---------------------------------------CG--HIF-----CK---KC-IEASIR------------FQK-------------------------------------------------KCPTC   38
TraesCS3D02G420900  CPVCINEL---------------------------VDASSTI---------------------------------------CG--HIF-----CK---KC-IEASIR------------FQK-------------------------------------------------KCPTC   38
TraesCS3B02G462300  CPVCISEL---------------------------VDASSTI---------------------------------------CG--HIF-----CK---KC-IEASIQ------------FQK-------------------------------------------------KCPTC   38
TraesCS3A02G425900  CPFCFDEL---------------------------VDASSTI---------------------------------------CG--HIF-----CH---KC-IKFSVQ------------AQN-------------------------------------------------RCPAC   38
TraesCS3B02G462000  CPFCFDEL---------------------------VDPSSTM---------------------------------------CG--HIF-----CE---KC-IKASIQ------------AQN-------------------------------------------------RCPAC   38
TraesCS3D02G420500  CPFCFEEL---------------------------VDAASTI---------------------------------------CG--HIF-----CH---KC-IEFSIQ------------AQS-------------------------------------------------RCPSC   38
TraesCS5B02G281700  CPVCWNKL---------------------------DEPATTM---------------------------------------CG--HIF-----CT---NC-IKQAIQ------------FQK-------------------------------------------------KCPTC   38
TraesCS5D02G289400  CPVCWNKL---------------------------DEPATTM---------------------------------------CG--HIF-----CT---NC-IKQAIQ------------FQK-------------------------------------------------KCPTC   38
TraesCS5A02G282300  CPVCWNKL---------------------------DEPATTM---------------------------------------CG--HIF-----CT---NC-IKQAIQ------------YQK-------------------------------------------------KCPTC   38
TraesCS6A02G349700  CPVCLNKL---------------------------VEPSTTK---------------------------------------CG--HIF-----CA---EC-IKQAIQ------------FQK-------------------------------------------------KCPTC   38
TraesCS6B02G382800  CPVCLNKL---------------------------VEPSTTK---------------------------------------CG--HIF-----CA---EC-IKQAIQ------------FQK-------------------------------------------------KCPTC   38
TraesCS6D02G332200  CPVCLNKL---------------------------VEPSTTK---------------------------------------CG--HIF-----CA---EC-IKQAIQ------------FQK-------------------------------------------------KCPTC   38
TraesCS7B02G154000  CPVCLNKM---------------------------ELPSATS---------------------------------------CG--HVF-----CE---KC-IKAAIK------------AQK-------------------------------------------------KCPTC   38
TraesCS7D02G257900  CPVCLNKM---------------------------ELPSATS---------------------------------------CG--HVF-----CE---KC-IKAAIK------------AQK-------------------------------------------------KCPTC   38
TraesCS7A02G256500  CPVCLNKM---------------------------ELPSATS---------------------------------------CG--HVF-----CE---KC-IKAAIK------------AQK-------------------------------------------------KCPTC   38
TraesCS6A02G326100  CPICMALI---------------------------KDAFLTA---------------------------------------CG--HSF-----CY---MC-IVTHLN------------HKS-------------------------------------------------DCPCC   38
TraesCS6D02G305800  CPICMALI---------------------------KDAFLTA---------------------------------------CG--HSF-----CY---MC-IVTHLN------------HKS-------------------------------------------------DCPCC   38
TraesCS6B02G356400  CPICMGLI---------------------------KDAFLTA---------------------------------------CG--HSF-----CY---MC-IVTHLN------------HKS-------------------------------------------------DCPCC   38
TraesCS1A02G329600  CPICLSLLR---------------------------SAVSVT---------------------------------------CN--HIF-----CN---HC-LTESMK------------SSS-------------------------------------------------TCPVC   38
TraesCS1B02G343100  CPICLSLLR---------------------------SAVSVT---------------------------------------CN--HIF-----CN---HC-LTESMK------------SSS-------------------------------------------------TCPVC   38
TraesCS1D02G331700  CPICLSLLR---------------------------SAVSVT---------------------------------------CN--HIF-----CN---HC-LTESMK------------SSS-------------------------------------------------TCPVC   38
TraesCS2D02G479100  CMICAEQRKN-------------YMDSRTGPKEPPENAVITT---------------------------------------CC--HIF-----CY---EC-AQESLS------------EEE-------------------------------------------------VCPVC   52
TraesCS3A02G259800  CCVCMVRH---------------------------KGAAFIP---------------------------------------CG--HTF-----CR---LC-SRELWV------------SRG-------------------------------------------------NCPLC   38
TraesCS3D02G259900  CCVCMVRH---------------------------KGAAFIP---------------------------------------CG--HTF-----CR---LC-SRELWV------------SRG-------------------------------------------------NCPLC   38
TraesCS3A02G423800  CCVCMVRH---------------------------KGAAFIP---------------------------------------CG--HTF-----CR---LC-SRELRH------------ARG-------------------------------------------------NCPLC   38
TraesCS3D02G419000  CCVCMVRH---------------------------KGAAFIP---------------------------------------CG--HTF-----CR---LC-SRELRH------------TRG-------------------------------------------------NCPLC   38
TraesCS3B02G459700  CCVCMARH---------------------------KGAAFIP---------------------------------------CG--HTF-----CR---LC-SRELRH------------TRG-------------------------------------------------NCPLC   38
TraesCS5A02G283800  CCVCMARR---------------------------KGAAFVP---------------------------------------CG--HTF-----CR---AC-AREVRA------------GRG-------------------------------------------------RCPLC   38
TraesCS5B02G282300  CCVCMARR---------------------------KGAAFVP---------------------------------------CG--HTF-----CR---AC-AREVRA------------GRG-------------------------------------------------RCPLC   38
TraesCS5D02G291000  CCVCMARR---------------------------KGAAFVP---------------------------------------CG--HTF-----CR---AC-AREVRA------------GRG-------------------------------------------------RCPLC   38
                    1.......10........20........30........40........50........60........70........80........90.......100.......110.......120.......130.......140.......150.......160.......170......
```

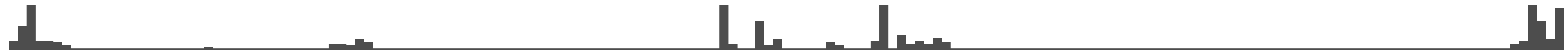

CLUSTAL 2.1 MULTIPLE SEQUENCE ALIGNMENT

File: C:/Users/HP/Downloads/clustal alignBB1.ps **Date: Wed May 20 03:00:39 2020**


```
                     *  *                                                                             *                 *                                                                        *
TraesCS7A02G257600   CCVCMARA---------------------------KGAAFIP---------------------------------------CG--HTF-----CR---AC-ARELLA------------GRG-------------------------------------------------RCPLC 38
TraesCS7B02G154600   CCVCMARA---------------------------KGAAFIP---------------------------------------CG--HTF-----CR---AC-ARELLA------------GRG-------------------------------------------------RCPLC 38
TraesCS7D02G258600   CCVCMARA---------------------------KGAAFIP---------------------------------------CG--HTF-----CR---AC-ARELFA------------GRG-------------------------------------------------RCPLC 38
TraesCS1B02G132800   CAICQEKM---------------------------HTPILLQ---------------------------------------CK--HIF-----CE---DC-ASEWLE------------RER-------------------------------------------------TCPLC 38
TraesCS1D02G114200   CAICQEKM---------------------------HTPILLQ---------------------------------------CK--HIF-----CE---DC-ASEWLE------------RER-------------------------------------------------TCPLC 38
TraesCS1A02G112600   CAICQEKM---------------------------HTPILLQ---------------------------------------CK--HIF-----CE---DC-ASEWLE------------RER-------------------------------------------------TCPLC 38
TraesCS1B02G152000   CAICQEKM---------------------------HVPVLLR---------------------------------------CK--HIF-----CE---DC-VSEWFE------------RER-------------------------------------------------TCPLC 38
TraesCS1D02G133300   CAICQEKM---------------------------HVPVLLR---------------------------------------CK--HIF-----CE---DC-VSEWFE------------RER-------------------------------------------------TCPLC 38
TraesCS1A02G137400   CAICQEKM---------------------------HVPVLLR---------------------------------------CK--HIF-----CE---DC-VSEWFE------------RER-------------------------------------------------TCPLC 38
TraesCS3B02G111000   CAICQEKM---------------------------HVPILLR---------------------------------------CK--HVF-----CE---DC-VSEWFE------------RER-------------------------------------------------TCPLC 38
TraesCS3D02G095600   CAICQEKM---------------------------HVPILLR---------------------------------------CK--HVF-----CE---DC-VSEWFE------------RER-------------------------------------------------TCPLC 38
TraesCS3A02G095400   CAICQEKM---------------------------HVPILLR---------------------------------------CK--HVF-----CE---DC-VSEWFE------------RER-------------------------------------------------TCPLC 38
TraesCS1A02G141900   CGICLEVN---------------------------AKIVLPD---------------------------------------CT--HSL-----CL---RC-FEEWNA------------KSK-------------------------------------------------SCPFC 38
TraesCS1D02G141000   CGICLEVN---------------------------AKIVLPD---------------------------------------CT--HSL-----CL---RC-FEEWNA------------KSK-------------------------------------------------SCPFC 38
TraesCS1A02G169300   CGICMEMN---------------------------SKVVLPN---------------------------------------CT--HAM-----CL---RC-YQDWNS------------RSQ-------------------------------------------------SCPFC 38
TraesCS1B02G187600   CGICMEMN---------------------------SKVVLPN---------------------------------------CT--HAM-----CL---RC-YQDWNS------------RSQ-------------------------------------------------SCPFC 38
TraesCS1D02G167300   CGICMEMN---------------------------SKVVLPN---------------------------------------CT--HAM-----CL---RC-YQDWNS------------RSQ-------------------------------------------------SCPFC 38
TraesCS2B02G148700   CGICMEIN---------------------------NKVVLPT---------------------------------------CS--HAM-----CI---KC-YRDWRS------------RSQ-------------------------------------------------SCPFC 38
TraesCS2D02G129300   CGICMEIN---------------------------NKVVLPT---------------------------------------CS--HAM-----CI---KC-YRDWRS------------RSQ-------------------------------------------------SCPFC 38
TraesCS2A02G126800   CGICMEIN---------------------------NKVVLPT---------------------------------------CS--HAM-----CI---KC-YRDWRS------------RSQ-------------------------------------------------SCPFC 38
TraesCS2A02G586800   CGICMELN---------------------------SRVVLPN---------------------------------------CS--HDM-----CI---KC-YRQWRS------------RSQ-------------------------------------------------SCPFC 38
TraesCS2D02G599700   CGICMELN---------------------------SRVVLPN---------------------------------------CS--HDM-----CI---NC-YRQWRS------------RSQ-------------------------------------------------SCPFC 38
TraesCS2B02G596600   CGICMELN---------------------------SRVVLPN---------------------------------------CS--HDM-----CI---KC-YHQWRT------------RSQ-------------------------------------------------SCPFC 38
TraesCS3A02G391400   CGICLETC---------------------------TKMVLPN---------------------------------------CN--HAM-----CI---NC-YRDWYT------------RSQ-------------------------------------------------SCPFC 38
TraesCS3D02G384200   CGICLETC---------------------------TKMVLPN---------------------------------------CN--HAM-----CI---NC-YRDWYT------------RSQ-------------------------------------------------SCPFC 38
TraesCS3B02G423300   CGICLEAC---------------------------TKMVLPN---------------------------------------CN--HAM-----CI---NC-YRDWYT------------RSQ-------------------------------------------------SCPFC 38
TraesCS1A02G322000   CPICFELLK------------------------SDIAVWPG----------------------------------------CLRPHVF-----HG---EC-LLLALK-------------AS------------------------------------------------EMCPLC 42
TraesCS1B02G334400   CPICFELLK------------------------SDVAVWPG----------------------------------------CLLPHVF-----HG---EC-LVLALQ-------------AS------------------------------------------------EMCPLC 42
TraesCS1D02G322200   CPICFELLK------------------------SDVAVWPG----------------------------------------CLRPHVF-----HG---AC-LVQALK-------------RS------------------------------------------------ETCPLC 42
TraesCS1A02G321800   CPVCLEPLE------------------------SDAASWPG----------------------------------------CSSPHVF-----HG---TC-LEGTLK-------------ES------------------------------------------------EKCPIC 42
TraesCS7B02G343500   CPICLQMLE-----------------------GDDLATWPE----------------------------------------CGKPHVF-----HG---AC-LERVLV-------------ES------------------------------------------------KTCPMC 43
TraesCS7D02G433200   CPICLQQLK-----------------------GDDLAAWPG----------------------------------------CGKPHVF-----HG---AC-LERVHV-------------EN------------------------------------------------KTCPMC 43
TraesCS7B02G343400   CPVCLQLLE-----------------------GNDLAAWSG----------------------------------------CDKPHVF-----HG---SC-LERVLV-------------ES------------------------------------------------KTCPMC 43
TraesCS1D02G101400   CPVCWQLLE-----------------------GDDLAAWPG----------------------------------------CGKPHVF-----HG---AC-LEQVLK-------------MS------------------------------------------------ETCPLC 43
TraesCS7D02G433400   CPVCLQLLEV-------------E--------GDDLAAWPG----------------------------------------CSKPHVF-----HS---AC-LERALV-------------ES------------------------------------------------VRCPMC 45
TraesCS3A02G309200   CPICLGQE------------------------EAAATAWKET-T-------------------------------------CG--HNF-----HG---RC-VERWLE-------------TK------------------------------------------------GTCPMC 42
TraesCS3D02G135000   CPICLGQE------------------------EAAATAWKET-A-------------------------------------CG--HSF-----HG---RC-VERWLE-------------TK------------------------------------------------GTCPMC 42
TraesCS3B02G152400   CPICLGQE------------------------EAAATVWKET-A-------------------------------------CG--HSF-----HG---RC-VERWLE-------------MK------------------------------------------------GTCPMC 42
TraesCS3B02G152100   CPICLGQE------------------------EATETTWMKT-A-------------------------------------CG--HIF-----HG---RC-VERWLK-------------MK------------------------------------------------GSCPVC 42
TraesCS3A02G309400   CPICLDQE-------------------------RVTTAWKET-V-------------------------------------CG--HIF-----HE---QC-VERWLE-------------TK------------------------------------------------GSCPMC 41
TraesCS3A02G309500   CPICLDQE-------------------------GATTAWKET-V-------------------------------------CG--HIF-----HE---RC-VERWLQ-------------AK------------------------------------------------GSCPMC 41
TraesCS4A02G385800   CPICMEEDAD-------------E--------DAATGAWKET-P-------------------------------------CG--HRF-----HG---RC-VERWLH-------------AK------------------------------------------------GSCPMC 45
TraesCS7D02G125200   CPICMEDDDD-------------D--------NAATGAWKET-P-------------------------------------CG--HRF-----HG---RC-VERWLR-------------VK------------------------------------------------GSCPMC 45
TraesCSU02G152200    CPICIEDD--------------------------DSAAWKET-P-------------------------------------CE--HRF-----HG---RC-LERWLQ-------------AK------------------------------------------------GSCPMC 40
TraesCSU02G152300    CPICIEDD--------------------------DSAAWKET-P-------------------------------------CE--HRF-----HG---RC-LERWLQ-------------AK------------------------------------------------GSCPMC 40
TraesCS4A02G385900   CPICLEDD--------------------------DTAAWKET-P-------------------------------------CG--HRF-----HG---RC-VQRWLQ-------------AK------------------------------------------------GSCPMC 40
TraesCS7D02G082700   CPICMDDD--------------------------E-VAWKETKP-------------------------------------CG--HRF-----HG---RC-VEPWLK-------------AK------------------------------------------------GSCPMC 40
TraesCS1A02G143300   CPVCFEYLFD-----------STMDI------------SVLH---------------------------------------CG--HTI-----HL---EC-LNEMR-VHHHFS-----------------------------------------------------------CPVC 43
TraesCS1D02G142100   CPVCFEYLFD-----------STMDI------------SVLH---------------------------------------CG--HTI-----HL---EC-LNEMR-VHHHFS-----------------------------------------------------------CPVC 43
TraesCS3A02G288900   CPICFEYLFE-----------SRNDV------------SVLP---------------------------------------CG--HTI-----HE---KC-LKEMK-EHCQFA-----------------------------------------------------------CPLC 43
TraesCS3D02G288700   CPICFEYLFE-----------SRNDV------------SVLP---------------------------------------CG--HTI-----HE---KC-LKEMK-EHCQFA-----------------------------------------------------------CPLC 43
TraesCS3B02G323600   CPICFEYLFE-----------SRNDV------------SVLP---------------------------------------CG--HTI-----HE---KC-LREMK-EHCQFA-----------------------------------------------------------CPLC 43
TraesCS4A02G249200   CPICYEYLFD-----------SLREA------------SVLR---------------------------------------CG--HTM-----HL---QC-FHEML-KHDKFT-----------------------------------------------------------CPMC 43
TraesCS4B02G065500   CPICYEYLFD-----------SLREA------------SVLR---------------------------------------CG--HTM-----HL---QC-FHDML-KHDKFT-----------------------------------------------------------CPMC 43
TraesCS4D02G064500   CPICYEYLFD-----------SLRES------------SVLR---------------------------------------CG--HTM-----HL---QC-FHEML-KHDKFT-----------------------------------------------------------CPMC 43
TraesCS5B02G101200   CPICYEYLFD-----------SLKGT------------RVLN---------------------------------------CG--HTM-----HM---DC-FAEMV-DHNKYT-----------------------------------------------------------CPIC 43
TraesCS5D02G107600   CPICYEYLFD-----------SLKGT------------RVLN---------------------------------------CG--HTM-----HM---DC-FAEMV-DHNKYT-----------------------------------------------------------CPIC 43
TraesCS5A02G095000   CPICYEYLFD-----------SLKGT------------RVLN---------------------------------------CG--HTM-----HM---DC-FAEMV-DHNKYT-----------------------------------------------------------CPIC 43
TraesCS3B02G208200   CKLCLKLLKERSAW-------NAQDLGV---------VAVLL---------------------------------------CG--HVY-----HA---DC-LDSLTAEAEKYDP---------------------------------------------------------PCPVC 53
TraesCS3D02G183800   CKLCLKPLKERSAW-------NAQDLGV---------VAVLL---------------------------------------CG--HVY-----HA---DC-LDSLTAEAEKYDP---------------------------------------------------------PCPVC 53
TraesCS3A02G176600   CKLCLKLLKERPAW-------NAQDLGV---------VAVLL---------------------------------------CG--HVY-----HA---DC-LDSLTAEAEKYDP---------------------------------------------------------PCPVC 53
TraesCS1B02G274400   CPVCLS-PFD-----------AASVVP-----------RVLP---------------------------------------CG--HSL-----CG---SC-ISSLPPASASAAASSLR------------------------------------------------------CPLC 49
TraesCS1D02G263900   CPVCLS-PFD-----------AASVVP-----------RVLP---------------------------------------CG--HSL-----CG---SC-ISSLPPASASAAASSLR------------------------------------------------------CPLC 49
TraesCS1A02G263800   CPVCLS-PFD-----------DSSVVP-----------RVLP---------------------------------------CG--HSL-----CG---SC-ISSLPPASASAAASSLR------------------------------------------------------CPLC 49
TraesCS4B02G251200   CPICCE-SFN-----------IVENVP-----------YVLW---------------------------------------CG--HTM-----CK---NC-ILGLQWAVVKFPTLPIQLPLF------------------------------------------------VSCPWC 55
TraesCS4D02G251200   CPICCE-SFN-----------IVENVP-----------YVLW---------------------------------------CG--HTM-----CK---NC-ILGLQWAVVKFPTLPIQLPLF------------------------------------------------VSCPWC 55
TraesCS4A02G053400   CPICCE-SFN-----------IVENVP-----------YVLW---------------------------------------CG--HTM-----CK---NC-ILGLQWAVVKFPTLPIQLPLF------------------------------------------------VSCPWC 55
TraesCS1A02G374800   CPICCDFLFT-----------SSAAV------------RGLP---------------------------------------CG--HFM-----HS---AC-FQAYT--CSHYT-----------------------------------------------------------CPIC 42
TraesCS1B02G395000   CPICCDFLFT-----------SSAAV------------RGLP---------------------------------------CG--HFM-----HS---AC-FQAYT--CSHYT-----------------------------------------------------------CPIC 42
TraesCS1D02G382000   CPICCDFLFT-----------SSAAV------------RGLP---------------------------------------CG--HFM-----HS---AC-FQAYT--CSHYT-----------------------------------------------------------CPIC 42
TraesCS4A02G101400b  CPSCKSNLTN-----------TMSLV------------AIST---------------------------------------CG--HVF-----CK---KC-SDKFI--ATDKV-----------------------------------------------------------CLMC 42
TraesCS4B02G203200b  CPSCKSNLTN-----------TMSLV------------AIST---------------------------------------CG--HVF-----CK---KC-SDKFI--ATDKV-----------------------------------------------------------CLMC 42
TraesCS4D02G204100b  CPSCKSNLTN-----------TMSLV------------AIST---------------------------------------CG--HVF-----CK---KC-SDKFV--ATDKV-----------------------------------------------------------CLMC 42
TraesCS2A02G545900a  CAKCMSVVP--------------CAC-----------IRPCFSM-------------------------------------CD--HDF-----CS---SC-VAMKLPQNVARLKCTGESSTASEKYRGVTILRPETVQVPSRIDPMFITDGNEGSPVN----DISDS-KRFYCAKC 98
TraesCS2B02G576800a  CAKCMSVVA--------------CAC-----------IRPGFSM-------------------------------------CD--HEF-----CS---SC-VAMKLPQNVARVKCTGESSTGSEKYHGLTILRPETVQLPSRIDPMFITEGNEGSLSEGKEGSLSDS-KRFYCAKC 102
                     1.......10........20........30........40........50........60........70........80........90.......100.......110.......120.......130.......140.......150.......160.......170......
```

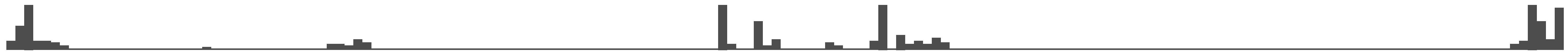

CLUSTAL 2.1 MULTIPLE SEQUENCE ALIGNMENT

File: C:/Users/HP/Downloads/clustal alignBB1.ps **Date: Wed May 20 03:00:39 2020**


```
                    *  *                                                                             *                 *                                                                        *
TraesCS2A02G545900c CAKCMHVVP--------------CAC-----------IRSNFTT-------------------------------------CD--HDF-----CS---RC-VGLKLCQNVPRVKCTVDNSTVAERYHSMAMVQPETA-VSSPIDPKFVTGGNEDSPLVDGN-DIGDD-KHFYCAKC  100
TraesCS2D02G547100b CAKCMHVVP--------------CAC-----------IRPNFTT-------------------------------------CD--HDF-----CS---RC-VGLKLCQNVPRVKCTVDNSTAAERYRSMATVRPET--VSSRIDPKFVTGGNEDSPLVDGN-DIGDG-KHFYCAKC   99
TraesCS2B02G576800c CAKCMHVVP--------------CAC-----------IRSNFTT-------------------------------------CD--HDF-----CS---RC-VGLKLCQNVPRVKCTVDNSTAAERYHSMAMVRPET--VSSRIGPKFVTGGNEDSPLVDSK-DIGDG-KHFYCAEC   99
TraesCS2B02G576800b CAKCMHVVP--------------CAC-----------IRSNFTM-------------------------------------CD--HDF-----CS---RC-VTMKLRQNID-------------------------------------------------------------CVKC   42
TraesCS2D02G547100a CAKCMHVVP--------------CAC-----------MRYNFTM-------------------------------------CD--HDF-----CS---RC-VSMKLRQNID-------------------------------------------------------------CVKC   42
TraesCS2D02G547300b CAKCMHVVP--------------CAC-----------IRRSFTS-------------------------------------CA--HDF-----CS---NC-VSRRLQGRR------------------------------------------------------GDG-KHFYCTKC   48
TraesCS7D02G415800b CAKCMHVVP--------------CAC-----------IRRSFTS-------------------------------------CA--HDF-----CS---SC-VSRRLRGRR------------------------------------------------------GDG-KHFYCTKC   48
TraesCS2A02G546000b CAKCMHVVP--------------CAC-----------VRRSFTS-------------------------------------CA--HDF-----CS---RC-VSRRLQGRL------------------------------------------------------GDG-KHFYCTKC   48
TraesCS2B02G577000a CAKCMHVVP--------------CAC-----------IRRSFTS-------------------------------------CA--HDF-----CS---SC-VSRRLQARR------------------------------------------------------GDGGKHFYCTKC   49
TraesCS6B02G310500  CSVCLDRVLSKP---------TAAEK----------RFGLLPE--------------------------------------CD--HAF-----CI---TC-IRKWRSSSLTSS--------------------------------------------------MDIDSTVKACPIC   58
TraesCS6D02G262500  CSVCLDRVLSKP---------TAAEK----------RFGLLPE--------------------------------------CD--HAF-----CI---TC-IRKWRSSSLTSS--------------------------------------------------MDIDSTVKACPIC   58
TraesCS7A02G228200  CSVCLDRVLSKP---------TAAER----------KFGLLSE--------------------------------------CD--HPF-----CI---SC-IRNWRGNSPTSG--------------------------------------------------MDVNSALRACPIC   58
TraesCS7D02G226400  CSVCLDRVLSKP---------TAAER----------KFGLLSE--------------------------------------CD--HPF-----CI---SC-IRNWRGNSPTSG--------------------------------------------------MDVNSALRACPIC   58
TraesCS7B02G191500  CSVCLDRVLSKS---------TAAER----------KFGLLSE--------------------------------------CD--HPF-----CI---SC-IRNWRGNSPTSG--------------------------------------------------MDVNSALRACPIC   58
TraesCS1B02G032600  CVICWTDF---------------------------SSSRGILP--------------------------------------CG--HRF-----CY---SC-IQGWADCLSSRG-------------------------------------------------------KVSTCPLC   45
TraesCS1D02G026100  CVICWTDF---------------------------SSSRGILP--------------------------------------CG--HRF-----CY---SC-IQGWADCLSSRG-------------------------------------------------------KVSTCPLC   45
TraesCS1A02G025700  CVICWTDF---------------------------SSSRGILP--------------------------------------CG--HRF-----CY---SC-IQGWADCLSSRG-------------------------------------------------------KVSTCPLC   45
TraesCS3A02G327900  CPICLGII---------------------------RKTRTVME--------------------------------------CL--HRF-----CR---DC-IDKSMRLG-----------------------------------------------------------NNE-CPAC   40
TraesCS3B02G357400  CPICLGII---------------------------RKTRTVME--------------------------------------CL--HRF-----CR---DC-IDKSMRLG-----------------------------------------------------------NNE-CPAC   40
TraesCS3D02G321400  CPICLGII---------------------------RKTRTVME--------------------------------------CL--HRF-----CR---DC-IDKSMRLG-----------------------------------------------------------NNE-CPAC   40
TraesCS1D02G315600  CPICLGII---------------------------RKTRTVME--------------------------------------CL--HRF-----CR---DC-IDKSMRLG-----------------------------------------------------------NNE-CPAC   40
TraesCS1B02G327300  CPICLGII---------------------------RKTRTVME--------------------------------------CL--HRF-----CR---DC-IDKSMRLG-----------------------------------------------------------NNE-CPAC   40
TraesCS1A02G315400  CPICLGII---------------------------RKTRTVME--------------------------------------CL--HRF-----CR---DC-IDKSMRLG-----------------------------------------------------------NNE-CPAC   40
TraesCS5B02G065600  CQLCHRLL---------------------------RNATTISE--------------------------------------CL--HTF-----CR---KC-IYKKLNDE-----------------------------------------------------------ECDHCPVC   41
TraesCS5D02G069800  CQLCHRLL---------------------------RNATTISE--------------------------------------CL--HTF-----CR---KC-IYKKLNDE-----------------------------------------------------------ECDHCPVC   41
TraesCS5A02G058000  CQLCHRLL---------------------------RNAATISE--------------------------------------CL--HTF-----CR---KC-IYKKLNDE-----------------------------------------------------------ECDHCPVC   41
TraesCS5A02G378600  CPLCNRLL---------------------------RDATTVSE--------------------------------------CL--HTF-----CR---KC-IYKKFNDE-----------------------------------------------------------EVESCPVC   41
TraesCS5B02G382100  CPLCNRLL---------------------------RDATTVSE--------------------------------------CL--HTF-----CR---KC-IYKKFNDE-----------------------------------------------------------EVESCPVC   41
TraesCS5D02G388500  CPLCNRLL---------------------------RDATTVSE--------------------------------------CL--HTF-----CR---KC-IYKKFNDE-----------------------------------------------------------EVESCPVC   41
TraesCS5A02G443000  CPLCRRLL---------------------------RDAATITE--------------------------------------CL--HTF-----CR---KC-ISKEFIDK-----------------------------------------------------------EICYCPTC   41
TraesCS5B02G447600  CPLCRRLL---------------------------RDAATITE--------------------------------------CL--HTF-----CR---KC-ISKEFIDK-----------------------------------------------------------EICYCPTC   41
TraesCS5D02G450400  CPLCRRLL---------------------------RDAATITE--------------------------------------CL--HTF-----CR---KC-ISKEFIDK-----------------------------------------------------------EICYCPTC   41
TraesCS5A02G381800  CPLCDGYF---------------------------REASAFTE--------------------------------------CG--HTF-----CR---EC-IMKKIDEE-----------------------------------------------------------EIEACPVC   41
TraesCS5D02G391100  CPLCDGYF---------------------------REASAFTE--------------------------------------CG--HTF-----CR---EC-IMKKIDEE-----------------------------------------------------------EIEACPVC   41
TraesCS5B02G385300  CPLCDGYF---------------------------REASAFTE--------------------------------------CG--HTF-----CR---EC-IMNKIDEE-----------------------------------------------------------EIEACPVC   41
TraesCS4A02G222800  CSICLNLW---------------------------HDVVTVAP--------------------------------------CF--HNF-----CN---GC-FSEWLRRSSSKSR---------------------------------------------------DKSQSAACPQC   49
TraesCS4B02G093600  CSICLNLW---------------------------HDVVTVAP--------------------------------------CF--HNF-----CN---GC-FSEWLRRSSSKSR---------------------------------------------------DKSQSAACPQC   49
TraesCS4D02G090400  CSICLNLW---------------------------HDVVTVAP--------------------------------------CF--HNF-----CN---GC-FSEWLRRSSSKSR---------------------------------------------------DKSQSAACPQC   49
TraesCS3D02G079000  CMVCQALL---------------------------HDASKCKP--------------------------------------CS--HKF-----CK---AC-ISRFKD-----------------------------------------------------------------CPLC   35
TraesCS5A02G460200  CPICLEGF---------------------------EDEAYLDT--------------------------------------CL--HSF-----CY---EC-ITQWVSIVASKHE---------------------------------------------------DPLSSVKCPLC   49
TraesCS5D02G471300  CPICLEGF---------------------------EDEAYLDT--------------------------------------CL--HSF-----CY---EC-ITQWVSIVASKHE---------------------------------------------------DPLSSVKCPLC   49
TraesCS5B02G469800  CPICLEGF---------------------------EDEAYLDT--------------------------------------CL--HSF-----CY---EC-ITQWVSIVASKHE---------------------------------------------------EPLSNVKCPLC   49
TraesCS3D02G400900  CPICLEAI---------------------------EDEAYLDA--------------------------------------CL--HSF-----CY---RC-ITQWVSIVASKRE---------------------------------------------------EPLSTARCPLC   49
TraesCS3B02G567600  CAVCGGVD---------------------------ISVPHQAN--------------------------------------CS--HWF-----CG---HC-IVGVWLQG--------------------------------------------------------SVLRPSNCPIC   44
TraesCS3D02G511300  CAVCRGVD---------------------------ISVPHQAN--------------------------------------CS--HWF-----CG---HC-IVGVWLQG--------------------------------------------------------SVLRPSNCPVC   44
TraesCS4A02G352200  CSVCHDR----------------------------FRIPCQAN--------------------------------------CS--HWF-----CG---EC-IIRVWNHG--------------------------------------------------------AAVQPCKCPIC   43
TraesCS5B02G519400  CSVCHDR----------------------------FRIPCQAN--------------------------------------CS--HWF-----CG---EC-IIRVWNHG--------------------------------------------------------AAVQPCKCPIC   43
TraesCS5D02G518700  CSVCHDR----------------------------FRIPCQAN--------------------------------------CS--HWF-----CG---EC-IIRVWNHG--------------------------------------------------------AAVQPCKCPIC   43
TraesCS1B02G123100  CSICLDAVVA-----------------------ASAERSTARLQ-------------------------------------CG--HEF-----HL---DC-IGSAFNAKGV-----------------------------------------------------------MQCPNC   46
TraesCS1D02G103800  CSICLDAVVA-----------------------ASAERSMARLQ-------------------------------------CG--HEF-----HL---DC-IGSAFNAKGV-----------------------------------------------------------MQCPNC   46
TraesCS7B02G163800  CSICLDPVAG-----------------------RAGGRSIAKLQ-------------------------------------CG--HEF-----HL---DC-IGSAFNAKGA-----------------------------------------------------------MQCPNC   46
TraesCS7D02G266300  CSICLDPVAG-----------------------RAGGRSIAKLQ-------------------------------------CG--HEF-----HL---DC-IGSAFNAKGA-----------------------------------------------------------MQCPNC   46
TraesCS7A02G268500  CSICLDPVTG-----------------------RAGGRSIAKLQ-------------------------------------CG--HEF-----HL---DC-IGSAFNAKGA-----------------------------------------------------------MQCPNC   46
TraesCS2B02G467200b CPHCGTGITR-----------------------VSG---------------------------------------------CN--HML-----CK---NC---------GK-----------------------------------------------------------LFCYGC   30
TraesCS2D02G445000b CPHCGTGITR-----------------------VSG---------------------------------------------CN--HML-----CK---NC---------GK-----------------------------------------------------------LFCYGC   30
TraesCS2A02G446100b CPHCGTGITR-----------------------VSG---------------------------------------------CN--HML-----CK---NC---------GK-----------------------------------------------------------LFCYGC   30
TraesCS3A02G491300  CAICLDRIPL-----------------------QETALVKG----------------------------------------CD--HAY-----CV---TC-ILRWASYKQT-----------------------------------------------------------PVCPQC   43
TraesCS3B02G552300  CAICLDRIPL-----------------------QETALVKG----------------------------------------CD--HAY-----CV---TC-ILRWASYKQT-----------------------------------------------------------PVCPQC   43
TraesCS3D02G497200  CAICLDRIPL-----------------------QETALVKG----------------------------------------CD--HAY-----CV---TC-ILRWASYKQT-----------------------------------------------------------PVCPQC   43
TraesCS5B02G555800  CPICYSIL-------------------------HTSNHGLPRLACKT----------------------------------CK--HKF-----HG---AC-LYKWFSTSNK-----------------------------------------------------------STCPLC   47
TraesCS5D02G553700  CPICYSIL-------------------------HTSNHGLPRLACKT----------------------------------CK--HKF-----HG---AC-LYKWFSTSNK-----------------------------------------------------------STCPLC   47
TraesCS4A02G322600  CPICYSIL-------------------------HTSNHGLPRLACKT----------------------------------CK--HKF-----HG---AC-LYKWFSTSNK-----------------------------------------------------------STCPLC   47
TraesCS1A02G158400  CRVCQQ----------K----------------SEEPLVDLG---------------------------------------CR--CRGDLSKAHR---TC-IDVWFRTRGS-----------------------------------------------------------NKCEIC   46
TraesCS1B02G174400  CRVCQQ----------K----------------SEEPLVDLG---------------------------------------CR--CRGDLSKAHR---TC-IDVWFRTRGS-----------------------------------------------------------NKCEIC   46
TraesCS1D02G155700  CRVCQQ----------K----------------SEEPLVDLG---------------------------------------CR--CRGDLSKAHR---TC-IDVWFRTRGS-----------------------------------------------------------NKCEIC   46
TraesCS1B02G289200  CRICHL---GLESAAAE----------------SGAGIT-LG---------------------------------------CS--CKGDLSYSHK---QC-AETWFKIRGN-----------------------------------------------------------KTCEIC   52
TraesCS1D02G279400  CRICHL---GLESAAAE----------------SGAGIT-LG---------------------------------------CS--CKGDLSYSHK---QC-AETWFKIRGN-----------------------------------------------------------KTCEIC   52
TraesCS1A02G280300  CRICHL---GLESAAAE----------------SGAGIT-LG---------------------------------------CS--CKGDLSYSHK---QC-AETWFKIRGN-----------------------------------------------------------KTCEIC   52
TraesCS3A02G365100  CRICHL---GLESAAAE----------------SGAGIV-LG---------------------------------------CS--CKDDLSCAHK---QC-AETWFKIRGN-----------------------------------------------------------KICEIC   52
TraesCS3D02G358300  CRICHL---GLESAAAE----------------SGAGIV-LG---------------------------------------CS--CKDDLSCAHK---QC-AETWFKIRGN-----------------------------------------------------------KICEIC   52
TraesCS3B02G397000  CRICHL---GLESAAAE----------------SGGGIV-LG---------------------------------------CS--CKDDLSCAHK---QC-AETWFKIRGN-----------------------------------------------------------KICEIC   52
TraesCS3A02G281400  CRICHLIPEGGDGPAAA----------------PGSEVIRLG---------------------------------------CC--CKEELGHAHR---QC-AEAWFRIKGD-----------------------------------------------------------RRCEIC   56
TraesCS3B02G315100  CRICHLSPEGGDGPAAA----------------PGSEVIRLG---------------------------------------CC--CKEELGHAHR---QC-AEAWFRIKGD-----------------------------------------------------------RRCEIC   56
TraesCS3D02G281400  CRICHLSPEGGDG--------------------PGSEVIRLA---------------------------------------CC--CKEELGHAHR---QC-AEAWFRIKGD-----------------------------------------------------------RRCEIC   52
TraesCS1B02G144400  CRICQLE--DGDLPAES----------------GSGQLVNLG---------------------------------------CG--CRGEIAAAHR---RC-AEAWFSVRGN-----------------------------------------------------------RRCEIC   54
                    1.......10........20........30........40........50........60........70........80........90........100.......110.......120.......130.......140.......150.......160.......170.....
```

```
                   *  *                                                                             *                 *                                                                        *
TraesCS1D02G128900 CRICQLE--DGDLPAES----------------GSGQLVNLG---------------------------------------CG--CRGEIAAAHR---RC-AEAWFSVRGN-----------------------------------------------------------RRCEIC   54
TraesCS1A02G206100 CRICHEEEGDDGCATAT----------------A----MESP---------------------------------------CG--CSGSLKYAHR---GC-VQRWCDEKGS-----------------------------------------------------------TVCEIC   52
TraesCS1D02G209400 CRICHEEEGDDGCATA----------------------MESP---------------------------------------CG--CSGSLKYAHR---GC-VQRWCDEKGS-----------------------------------------------------------TVCEIC   50
TraesCS1B02G219700 CRICHEEERDDGCVTAT----------------ATATAMESP---------------------------------------CG--CSGSLKYAHR---GC-VQRWCDEKGS-----------------------------------------------------------TVCEIC   56
TraesCS1A02G225700 CRICHEEE--DESSAA----------------------MESP---------------------------------------CA--CSGSLKYAHR---GC-VQRWCDEKGS-----------------------------------------------------------TLCEIC   48
TraesCS1B02G238900 CRICHEEE--DESSAA----------------------MESP---------------------------------------CA--CSGSLKYAHR---GC-VQRWCDEKGS-----------------------------------------------------------TLCEIC   48
TraesCS1D02G226900 CRICHEEE--DESCAA----------------------MESP---------------------------------------CA--CSGSLKYAHR---GC-VQRWCDEKGS-----------------------------------------------------------TLCEIC   48
TraesCS1A02G378100 CRICQEDG----DETC----------------------MEAP---------------------------------------CS--CKGSLKYAHR---TC-VQRWCDEKGD-----------------------------------------------------------TICEIC   46
TraesCS1B02G398900 CRICQEDG----DESC----------------------MEAP---------------------------------------CS--CKGSLKYAHR---TC-VQRWCDEKGD-----------------------------------------------------------TICEIC   46
TraesCS3A02G264000 CRICQEEG----DQAY----------------------METP---------------------------------------CS--CKGSLKYAHH---IC-IQRWCNEKGD-----------------------------------------------------------TICEIC   46
TraesCS3B02G297400 CRICQEEG----DQAY----------------------METP---------------------------------------CS--CKGSLKYAHH---IC-IQRWCNEKGD-----------------------------------------------------------TICEIC   46
TraesCS3D02G264100 CRICQEEG----DQAY----------------------METP---------------------------------------CS--CKGSLKYAHH---IC-IQRWCNEKGD-----------------------------------------------------------TICEIC   46
TraesCS3A02G025800 CRICQEED----WDTG----------------------MEAP---------------------------------------CA--CRGSLKYAHR---KC-IQRWCNEKGD-----------------------------------------------------------TICEIC   46
TraesCS3B02G019200 CRICQEED----WDTG----------------------MEAP---------------------------------------CA--CRGSLKYAHR---KC-IQRWCNEKGD-----------------------------------------------------------TVCEIC   46
TraesCS3D02G019900 CRICQEED----WDAG----------------------MEAP---------------------------------------CA--CRGSLKYAHR---KC-IQRWCSEKGD-----------------------------------------------------------TVCEIC   46
TraesCS4A02G164100 CRICQEED----SIKN----------------------LEKP---------------------------------------CT--CNGSLKYAHR---AC-VQRWCNEKGD-----------------------------------------------------------ITCEIC   46
TraesCS4B02G149000 CRICQEED----SIKN----------------------LEKP---------------------------------------CT--CNGSLKYAHR---AC-VQRWCNEKGD-----------------------------------------------------------ITCEIC   46
TraesCS4D02G147800 CRICQEED----SIKN----------------------LEKP---------------------------------------CT--CNGSLKYAHR---AC-VQRWCNEKGD-----------------------------------------------------------ITCEIC   46
TraesCS5B02G227200 CRICQEED----DIKN----------------------LESP---------------------------------------CA--CTGSVKYAHR---AC-VQRWCNEKGD-----------------------------------------------------------VTCEIC   46
TraesCS5D02G239200 CRICQEED----DIKN----------------------LESP---------------------------------------CA--CTGSVKYAHR---AC-VQRWCNEKGD-----------------------------------------------------------VTCEIC   46
TraesCS5A02G228400 CRICQEED----DIKN----------------------LESP---------------------------------------CA--CTGSVKYAHR---AC-VQRWCNEKGD-----------------------------------------------------------VTCEIC   46
TraesCS3B02G432700 CRICQEEG----EEAA----------------------MDSP---------------------------------------CA--CTGTLKFAHR---KC-IQRWCDKKGN-----------------------------------------------------------ITCEIC   46
TraesCS3D02G393800 CRICQEEG----EEAA----------------------MDSP---------------------------------------CA--CTGTLKFAHR---KC-IQRWCDKKGN-----------------------------------------------------------ITCEIC   46
TraesCS3A02G399700 CRICQEEG----DEAA----------------------MDSP---------------------------------------CA--CTGTLKFAHR---KC-IQRWCDKKGN-----------------------------------------------------------ITCEIC   46
TraesCS2A02G039300 CRICMVELSE------------------------GGGAMKLE---------------------------------------CS--CRGELALAHT---DC-ALKWFGIKGT-----------------------------------------------------------RTCEVC   48
TraesCS2B02G051700 CRICMVELSE------------------------GGGAMKLE---------------------------------------CS--CRGELALAHT---DC-ALKWFGIKGT-----------------------------------------------------------RTCEVC   48
TraesCS2D02G037600 CRICMVELSE------------------------GGGAMKLE---------------------------------------CS--CRGELALAHT---DC-ALKWFGIKGT-----------------------------------------------------------RTCEVC   48
TraesCS7A02G463200 CRICFVELNE------------------------GGETLKME---------------------------------------CS--CKGELALAHQ---DC-AVKWFSIKGN-----------------------------------------------------------KICDVC   48
TraesCS7B02G363900 CRICFVELNE------------------------GGETLKME---------------------------------------CS--CKGELALAHQ---DC-AVKWFSIKGN-----------------------------------------------------------KICDVC   48
TraesCS7D02G451000 CRICFVELNE------------------------GGETLKME---------------------------------------CS--CKGELALAHQ---DC-AVKWFSIKGN-----------------------------------------------------------KICDVC   48
TraesCS6A02G001000 CRICMVALLEEEEEE--E-------------GGAGDGVLKLE---------------------------------------CR--CKGELALAHR---RC-ALKWFGIKGN-----------------------------------------------------------PNCDVC   57
TraesCS6B02G005200 CRICMVALLEEEEE------------------GAGDGVLKLE---------------------------------------CR--CKGELALAHR---RC-ALKWFGIKGD-----------------------------------------------------------PNCDVC   54
TraesCS6D02G005100 CRICMVALLEEEEEEGGA-------------AGAGDGVLKLE---------------------------------------CR--CKGELALAHR---RC-ALKWFGIKGN-----------------------------------------------------------PNCDVC   59
TraesCS4A02G156600 CRICLETD---------------------------GRDFIAP---------------------------------------CK--CKGTSKYVHR---DC-LDHWRAVKEGF--------------------------------------------------------AFSHCTTC   48
TraesCS4B02G164000 CRICLETD---------------------------GRDFIAP---------------------------------------CK--CKGTSKYVHR---DC-LDHWRAVKEGF--------------------------------------------------------AFSHCTTC   48
TraesCS4D02G152900 CRICLETD---------------------------GRDFIAP---------------------------------------CK--CKGTSKYVHR---DC-LDHWRAVKEGF--------------------------------------------------------AFSHCTTC   48
TraesCS6A02G096200 CRICLDNE---------------------------GDDLIAP---------------------------------------CN--CKGTQKYVHR---SC-LDNWRSTKEGF--------------------------------------------------------AFSHCTEC   48
TraesCS6B02G124600 CRICLDNE---------------------------GDDLIAP---------------------------------------CN--CKGTQKYVHR---SC-LDNWRSTKEGF--------------------------------------------------------AFSHCTEC   48
TraesCS6D02G087300 CRICLDNE---------------------------GDDLIAP---------------------------------------CN--CKGTQKYVHR---SC-LDNWRSTKEGF--------------------------------------------------------AFSHCTEC   48
TraesCS7B02G176700 CRICLEAESEI------------------------GDELISP---------------------------------------CM--CKGTQQFVHR---SC-LDHWRSVKEGF--------------------------------------------------------AFSHCTTC   51
TraesCS7D02G282300 CRICLEAESEI------------------------GDELISP---------------------------------------CM--CKGTQQFVHR---SC-LDHWRSVKEGF--------------------------------------------------------AFSHCTTC   51
TraesCS1A02G328300 CRICRLPA-------------------------EAGRPLRHP---------------------------------------CA--CRGSIRFVHD---DC-QLRWLATRQG-----------------------------------------------------------PRCELC   47
TraesCS1B02G342100 CRICRLPA-------------------------EAGRPLRHP---------------------------------------CA--CRGSIRFVHD---DC-QLRWLATRQR-----------------------------------------------------------PRCELC   47
TraesCS1D02G330800 CRICRLPA-------------------------EADRPLRHP---------------------------------------CA--CRGSIRFVHD---DC-QLRWLATRRG-----------------------------------------------------------PRCELC   47
TraesCS7A02G340400 CRICRFPA-------------------------EADRPLRRP---------------------------------------CA--CSGSIRFVHD---DC-LLRWLATSRQ-----------------------------------------------------------SRCEVC   47
TraesCS7B02G252000 CRICRFPA-------------------------EADRPLRRP---------------------------------------CA--CSGSIRFVHD---DC-LLRWLATSRQ-----------------------------------------------------------SRCEVC   47
TraesCS1A02G430800 CRICRLPA-------------------------EAERPLRSP---------------------------------------CA--CRGSIRFVHD---EC-QLRWIAARGK-----------------------------------------------------------PLCEVC   47
TraesCS1B02G465500 CRICRLPA-------------------------EAERPLRSP---------------------------------------CA--CRGSIRFVHD---EC-QLRWIAARGK-----------------------------------------------------------PLCEVC   47
TraesCS1D02G439900 CRICRLPA-------------------------EAERPLRSP---------------------------------------CA--CRGSIRFVHD---EC-QLRWISARGK-----------------------------------------------------------PLCEVC   47
TraesCS1A02G465700 CRICRHPA-------------------------DADRPLRRP---------------------------------------CG--CKGSMRFVHD---HC-QIRWLKARRQ-----------------------------------------------------------SLCEVC   47
TraesCS1B02G465300 CRICRLPA-------------------------EADRPLHRP---------------------------------------CG--CRGSMRFVHD---DC-QLQWIARRRKL----------------------------------------------------------PRCEVC   48
TraesCS7B02G442100 CRICRNRG-------------------------DDEHPLRYP---------------------------------------CA--CSGSIKFVHQ---DC-LLQWLDHSNS-----------------------------------------------------------RQCEVC   47
TraesCS7D02G513500 CRICRNRG-------------------------DDEHPLRYP---------------------------------------CA--CSGSIKFVHQ---DC-LLQWLDHSNS-----------------------------------------------------------RQCEVC   47
TraesCS7A02G524600 CRICRNRG-------------------------DDEHPLRYP---------------------------------------CA--CSGSIKFVHQ---DC-LLQWLDHSNS-----------------------------------------------------------RQCEVC   47
TraesCS1B02G101700 CVMCLSEE-----------------------------MSVVFLP-------------------------------------CA--HQVV----CA---KC-NDLHDKQ------------------------------------------------------------GMKECPSC   40
TraesCS1D02G085400 CVMCLSEE-----------------------------MSVVFLP-------------------------------------CA--HQVV----CA---KC-NDLHDKQ------------------------------------------------------------GMKECPSC   40
TraesCS1A02G084200 CVMCLSEE-----------------------------MSVVFLP-------------------------------------CA--HQVV----CA---KC-NDLHVKQ------------------------------------------------------------GMKECPSC   40
TraesCS5B02G194700 CIICKREE-----------------------------ACVMLLQ-------------------------------------CA--HQVL----CV---GC-NKQHEEK------------------------------------------------------------GAVRCPSC   40
TraesCS5D02G202000 CIICKREE-----------------------------ACVMLLQ-------------------------------------CA--HQVL----CV---GC-NKQHEEK------------------------------------------------------------GAVRCPSC   40
TraesCS5A02G189000 CIICKREE-----------------------------ACVMLLQ-------------------------------------CA--HQVL----CV---GC-NKQHEEK------------------------------------------------------------GAVRCPSC   40
TraesCS1A02G177600 CKVCFESA-----------------------------TAAVLLP-------------------------------------CR--HFCL----CK---PC-AL-----------------------------------------------------------------ACSECPLC   35
TraesCS1B02G202600 CKVCFESA-----------------------------TAAVLLP-------------------------------------CR--HFCL----CK---PC-AL-----------------------------------------------------------------ACSECPLC   35
TraesCS1D02G176100 CKVCFESA-----------------------------TAAVLLP-------------------------------------CR--HFCL----CK---PC-AL-----------------------------------------------------------------ACSECPLC   35
TraesCS6A02G120700 CKGCGENA-----------------------------PVVVLLP-------------------------------------CR--HLSI----CG---PC-AE-----------------------------------------------------------------VASGCPSC   35
TraesCS6B02G149100 CKACGENA-----------------------------PVVVLLP-------------------------------------CR--HLSV----CG---PC-AE-----------------------------------------------------------------AASGCPSC   35
TraesCS6D02G110700 CKGCGENT-----------------------------PVAVLLP-------------------------------------CR--HLSV----CG---TC-AE-----------------------------------------------------------------ALSGCPSC   35
TraesCS7A02G441900 CKGCREDA-----------------------------AVVLLLP-------------------------------------CR--HLCV----CA---PC-AA-----------------------------------------------------------------AAQACPAC   35
TraesCS7D02G431200 CKGCREDA-----------------------------AVVLLLP-------------------------------------CR--HLCV----CA---PC-AA-----------------------------------------------------------------AAQACPAC   35
TraesCS7D02G431100 CKGCREGA-----------------------------AVVLLLP-------------------------------------CR--HLCV----CA---PC-AA-----------------------------------------------------------------AAQACPAC   35
TraesCS7B02G340500 CKGCREGA-----------------------------AVVLLLP-------------------------------------CR--HLCV----CA---SC-AA-----------------------------------------------------------------AAQACPAC   35
TraesCS7A02G442300 CKGCREGA-----------------------------AVVLLLP-------------------------------------CR--HLCV----CA---PC-AA-----------------------------------------------------------------VAQACPVC   35
TraesCS7D02G431300 CKGCREGA-----------------------------AVVLLLP-------------------------------------CR--HLCV----CA---PC-AA-----------------------------------------------------------------AAQACPVC   35
TraesCS7B02G340700 CKGCREGA-----------------------------AVVLLLP-------------------------------------CR--HLCV----CA---PC-AA-----------------------------------------------------------------AAQVCPVC   35
TraesCS7A02G442100 CKACREGA-----------------------------AVVLLLP-------------------------------------CR--HLCV----CA---PC-AA-----------------------------------------------------------------AALACPAC   35
TraesCS7B02G340600 CKACHDGA-----------------------------AVVLLLP-------------------------------------CR--HLSV----CA---PC-AA-----------------------------------------------------------------AAQACPAC   35
                   1.......10........20........30........40........50........60........70........80........90.......100.......110.......120.......130.......140.......150.......160.......170......
```

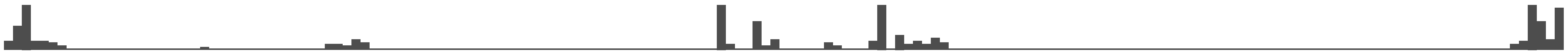

CLUSTAL 2.1 MULTIPLE SEQUENCE ALIGNMENT


```
                    *  *                                                                             *                 *                                                                        *
TraesCS2A02G290100  CRACGEGE-----------------------------SCVLLLP-------------------------------------CR--HLCL----CS---AC-DA-----------------------------------------------------------------AVDTCPLC  35
TraesCS2B02G306600  CRACGEGE-----------------------------SCVLLLP-------------------------------------CR--HLCL----CS---AC-DA-----------------------------------------------------------------AVDTCPLC  35
TraesCS2D02G288000  CRACGEGE-----------------------------SCVLLLP-------------------------------------CR--HLCL----CS---AC-DA-----------------------------------------------------------------AVDTCPLC  35
TraesCS4B02G215100  CRVCKSSE-----------------------------ACMLLLP-------------------------------------CR--HLCL----CK---EC-ES-----------------------------------------------------------------KLSFCPLC  35
TraesCS4D02G215600  CRVCKSSE-----------------------------ACMLLLP-------------------------------------CR--HLCL----CK---EC-ES-----------------------------------------------------------------KLSFCPLC  35
TraesCS4A02G089200  CRVCKSSE-----------------------------ACMLLLP-------------------------------------CR--HLCL----CK---EC-ES-----------------------------------------------------------------KLSFCPLC  35
TraesCS6B02G118100  CRWCGAKE-----------------------------AAVLVMP-------------------------------------CR--HLCL----CA---DC-DR-----------------------------------------------------------------VADACPVC  35
TraesCSU02G113400   CRLCGAKE-----------------------------AAVLVMP-------------------------------------CR--HLCL----CA---DC-DR-----------------------------------------------------------------VADACPVC  35
TraesCS6A02G084400  CKWCGAKE-----------------------------AAVLVMP-------------------------------------CR--HLCL----CA---DC-DR-----------------------------------------------------------------VTDACPVC  35
TraesCS4B02G042900  CRICYEGE-----------------------------ICMVILP-------------------------------------CR--HRTL----CK---SC-AE-----------------------------------------------------------------KCKRCPIC  35
TraesCS4D02G040200  CRICYEGE-----------------------------ICMVILP-------------------------------------CR--HRTL----CK---SC-AE-----------------------------------------------------------------KCKRCPIC  35
TraesCS4A02G271200  CRICYEGE-----------------------------ICMVILP-------------------------------------CR--HRTL----CK---SC-AE-----------------------------------------------------------------KCKRCPIC  35
TraesCS5B02G205500  CRICFERD-----------------------------ICIVLLP-------------------------------------CR--HHVL----CE---PC-SD-----------------------------------------------------------------KCQSCPIC  35
TraesCS5D02G213300  CRICFERD-----------------------------ICIVLLP-------------------------------------CR--HHVL----CE---PC-SD-----------------------------------------------------------------KCQSCPIC  35
TraesCS5A02G207100  CRICFERY-----------------------------ICIVLLP-------------------------------------CR--HHVL----CE---PC-SD-----------------------------------------------------------------KCQSCPIC  35
TraesCS1A02G215300  CRACRLRP-----------------------------ATTVLLP-------------------------------------CR--HLCV----CA---AC-DPGASASLA----------------------------------------------------------AAAACPAC  42
TraesCS1B02G228800  CRACRLRP-----------------------------ATTVLLP-------------------------------------CR--HLCV----CA---AC-DPGASASQAGTA-------------------------------------------------------AAAACPAC  45
TraesCS1D02G217800  CRGCRLRP-----------------------------ATTVLLP-------------------------------------CR--HLCV----CD---AC-DPGASASLA----------------------------------------------------------AAAACPTC  42
TraesCS1A02G088100  CVICLDAP-----------------------------VEGACIP-------------------------------------CG--HMAG----CM---SC--LKDIESKKW------------------------------------------------------------GCPIC  39
TraesCS1B02G107100  CVICLDAP-----------------------------VEGACIP-------------------------------------CG--HMAG----CM---SC--LKDIESKKW------------------------------------------------------------GCPIC  39
TraesCS1D02G089400  CVICLDAP-----------------------------VEGACIP-------------------------------------CG--HMAG----CM---SC--LKDIESKKW------------------------------------------------------------GCPIC  39
TraesCS1A02G341400  CVVCCDAR-----------------------------RDCFFMP-------------------------------------CG--HSAT----CH---AC-GTKIVEGDAS------------------------------------------------------------CCPFC  40
TraesCS1D02G343700  CVVCCDAR-----------------------------RDCFFMP-------------------------------------CG--HSAT----CH---AC-GTKIVEGDAS------------------------------------------------------------CCPFC  40
TraesCS1B02G354100  CVVCCDAR-----------------------------RDCFFMP-------------------------------------CG--HSAT----CH---AC-GTKIVEGDAS-------------------------------------------------------------CPFC  39
TraesCS2B02G162000  CVICLEQE-----------------------------YNAVFVP-------------------------------------CG--HMCC----CM---NC-SSHVTN-----------------------------------------------------------------CPLC  35
TraesCS2D02G141100  CVICLEQE-----------------------------YNAVFVP-------------------------------------CG--HMCC----CM---NC-SSHVTN-----------------------------------------------------------------CPLC  35
TraesCS4A02G011000  CVICLRKR-----------------------------RRAAFVP-------------------------------------CG--HLVC----CC---NC-AKRVELMDEP------------------------------------------------------------LCPVC  40
TraesCS4B02G293400  CVICLRKR-----------------------------RRAAFVP-------------------------------------CG--HLVC----CC---NC-AKRVELMDEP------------------------------------------------------------LCPVC  40
TraesCS4D02G292000  CVICLRKR-----------------------------RRAAFVP-------------------------------------CG--HLVC----CC---NC-AKRVELMDEP------------------------------------------------------------LCPVC  40
TraesCS2A02G156000  CGVCMEPL-----------------------------EWVAIGP-------------------------------------CG--HREV----CS---DC-AVRMRAGSKSQH-------------------------------------------------------ASRRCCIC  45
TraesCS2D02G161800  CGVCMEPL-----------------------------EWVAIGP-------------------------------------CG--HREV----CS---DC-AVRMRVGSKPQH-------------------------------------------------------ASRRCCIC  45
TraesCS2B02G181500  CGVCMEPL-----------------------------EWVALGP-------------------------------------CG--HREV----CS---DC-AVRMRVGSKSQH-------------------------------------------------------ASRRCCIC  45
TraesCS4B02G220700  CVICLSEP-----------------------------RDTTVLP-------------------------------------CR--HMCM----CS---EC-AKVLRYQTTR-------------------------------------------------------------CPIC  39
TraesCS4D02G221000  CVICLSEP-----------------------------RDTTVLP-------------------------------------CR--HMCM----CS---EC-AKVLRYQTTR-------------------------------------------------------------CPIC  39
TraesCS4A02G083500  CVICLSEP-----------------------------RDTTVLP-------------------------------------CR--HMCM----CS---EC-AKVLRYQTTR-------------------------------------------------------------CPIC  39
TraesCS1B02G258900  CVICLTEP-----------------------------RDTAVFP-------------------------------------CR--HLCM----CS---EC-AQALRLQSNK-------------------------------------------------------------CPIC  39
TraesCS7A02G486700  CVVCLTEP-----------------------------RDTTVLP-------------------------------------CR--HMCL----CR---EC-AQTLRFQTNK-------------------------------------------------------------CPMC  39
TraesCS7B02G390000  CVVCLTEP-----------------------------RDTTVLP-------------------------------------CR--HMCL----CR---EC-AQTLRFQTNK-------------------------------------------------------------CPMC  39
TraesCS7D02G473200  CVVCLTEP-----------------------------RDTTVLP-------------------------------------CR--HMCL----CR---EC-AQTLRFQTNK-------------------------------------------------------------CPMC  39
TraesCS2B02G251300  CCICHEMQ-----------------------------VDSLLYR-------------------------------------CG--HMCT----CF---NC-ADQLKSSSGS-------------------------------------------------------------CPIC  39
TraesCS2D02G233500  CCICHEMQ-----------------------------VDSLLYR-------------------------------------CG--HMCT----CF---NC-ADQLKSSSGS-------------------------------------------------------------CPIC  39
TraesCS2A02G229600  CCICHEMQ-----------------------------VDSLLYR-------------------------------------CG--HMCT----CF---NC-ADQLKSSSES-------------------------------------------------------------CPIC  39
TraesCS4B02G003100  CCVCCDTP-----------------------------IDSLLYR-------------------------------------CG--HMCT----CS---KC-ANELVRSGGK-------------------------------------------------------------CPLC  39
TraesCS4D02G003000  CCVCCDTP-----------------------------IDSLLYR-------------------------------------CG--HMCT----CS---KC-ANELVRSGGK-------------------------------------------------------------CPLC  39
TraesCS4A02G311200  CCVCCDTP-----------------------------IDSLLYR-------------------------------------CG--HMCT----CS---KC-ANELVRSGGK-------------------------------------------------------------CPLC  39
TraesCS6B02G449800  CCVCCDTQ-----------------------------IDSLLYR-------------------------------------CG--HMCT----CS---KC-ANELVRSGGK-------------------------------------------------------------CPLC  39
TraesCS6D02G389100  CCVCCDTQ-----------------------------IDSLLYR-------------------------------------CG--HMCT----CS---KC-ANELVRSGGK-------------------------------------------------------------CPLC  39
TraesCS6A02G405400  CCVCCDTQ-----------------------------IDSLLYR-------------------------------------CG--HMCT----CS---KC-ANELVRSGGK-------------------------------------------------------------CPLC  39
TraesCS4D02G354300  CCICCDSQ-----------------------------IDSLLYR-------------------------------------CG--HMCT----CS---KC-ARELLRGAGK-------------------------------------------------------------CPLC  39
TraesCS5A02G529500  CCICCDSQ-----------------------------IDSLLYR-------------------------------------CG--HMCT----CS---KC-ARELLRGAGK-------------------------------------------------------------CPLC  39
TraesCS3B02G449200  CPICLVNP-----------------------------KD-MAFG-------------------------------------CG--HQT-----CC---EC-GQT-----------------------------------------------------------------LESCPIC  33
TraesCS3D02G408900  CPICLVNP-----------------------------KD-MAFG-------------------------------------CG--HQT-----CC---EC-GQT-----------------------------------------------------------------LESCPIC  33
TraesCS3A02G414400  CPICLVNP-----------------------------KD-MAFG-------------------------------------CG--HQT-----CC---EC-GQT-----------------------------------------------------------------LESCPIC  33
TraesCS3A02G503300  CPICLRNP-----------------------------KD-MAFQ-------------------------------------CG--HLT-----CR---EC-GPT-----------------------------------------------------------------LSTCPVC  33
TraesCS3D02G509900  CPICLRNP-----------------------------RD-MAFQ-------------------------------------CG--HLT-----CR---EC-GPT-----------------------------------------------------------------LSTCPVC  33
TraesCS3B02G566700  CPICLRNP-----------------------------RD-MAFQ-------------------------------------CG--HLT-----CR---NC-GPA-----------------------------------------------------------------LSTCPVC  33
TraesCS3B02G566800  CPICLTNP-----------------------------KD-MAFQ-------------------------------------CG--HLT-----CK---EC-GPT-----------------------------------------------------------------LSTCPLC  33
TraesCS3D02G510000  CPICLTNP-----------------------------KD-MAFQ-------------------------------------CG--HLT-----CK---EC-GPT-----------------------------------------------------------------LSTCPLC  33
TraesCS3A02G503400  CPICLTNP-----------------------------KD-MAFQ-------------------------------------CG--HLT-----CK---EC-GPT-----------------------------------------------------------------LSTCPLC  33
TraesCS7A02G308900  CPICLTNA-----------------------------KD-LAFG-------------------------------------CG--HMC-----CR---EC-GEN-----------------------------------------------------------------LDRCPIC  33
TraesCS7D02G305600  CPICLTNA-----------------------------KD-LAFG-------------------------------------CG--HMC-----CR---EC-GEN-----------------------------------------------------------------LDRCPIC  33
TraesCS7A02G381200  CAICMDKS-----------------------------KD-LAFG-------------------------------------CG--HQT-----CY---DC-GGK-----------------------------------------------------------------LVRCPMC  33
TraesCS7D02G377500  CAICMDKS-----------------------------KD-LAFG-------------------------------------CG--HQT-----CY---DC-GEK-----------------------------------------------------------------LVRCPMC  33
TraesCS7B02G283200  CAICMDKS-----------------------------KD-LAFG-------------------------------------CG--HQT-----CY---DC-GKN-----------------------------------------------------------------LVRCPMC  33
TraesCS7D02G377600  CAICMDKS-----------------------------KD-LAFG-------------------------------------CG--HQT-----CY---DC-GKN-----------------------------------------------------------------LVRCPMC  33
TraesCS7A02G381300  CAICMDKS-----------------------------KD-LAFG-------------------------------------CG--HQT-----CY---DC-GKN-----------------------------------------------------------------LVRCPMC  33
TraesCS7B02G283100  CAICTDKS-----------------------------KD-LAFG-------------------------------------CG--HQT-----CY---DC-GQN-----------------------------------------------------------------LVRCPMC  33
TraesCS7B02G134400  CPICWSEA-----------------------------KN-LAFG-------------------------------------CG--HQT-----CA---DC-GKD-----------------------------------------------------------------LKVCPLC  33
TraesCS5B02G111900  CPVCLAKP-----------------------------RD-MAFG-------------------------------------CG--HQT-----CS---GC-GPQ-----------------------------------------------------------------VADCPIC  33
TraesCS5D02G122000  CPVCLAKP-----------------------------RD-MAFG-------------------------------------CG--HQT-----CS---GC-GPQ-----------------------------------------------------------------VVDCPIC  33
TraesCS5B02G223400b CPKCKIYVER-----------VDG-------------CTFIACR-------------------------------------CG--HCF-----CY---LC-GATMARTN-------------------------------------------------------------HYCAKC  43
TraesCS2A02G446100a CMICFSEYKG-------------------------IDFIKLP---------------------------------------CR--HYF-----CR---NC-METYSRMHVKEGSVMKIVCPDNKCGG---------FVPPNLLKRLLGESDFERWERLILERTLDAMADVAYCPRC  92
                    1.......10........20........30........40........50........60........70........80........90........100.......110.......120.......130.......140.......150.......160.......170......
```

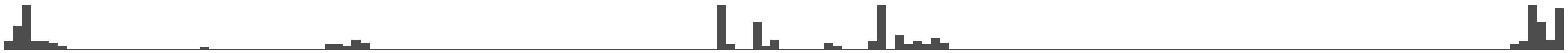

Supplementary Fig. S1. Multiple sequence alignment of 1, 272 wheat RING finger domains.

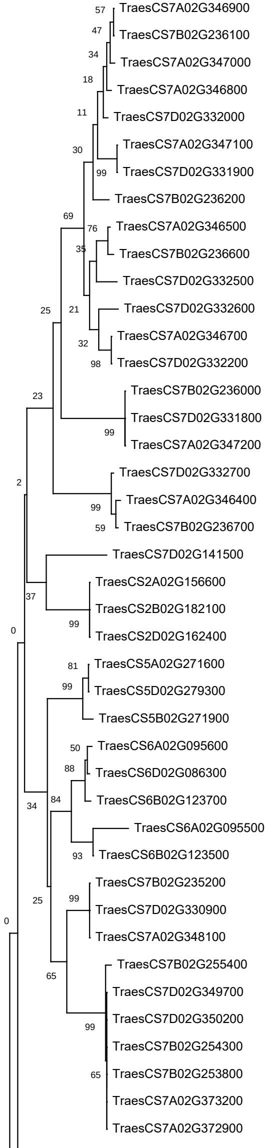

57
TraesCS7A02G346900
47
TraesCS7B02G236100
34
TraesCS7A02G347000
18
TraesCS7A02G346800
11
TraesCS7D02G332000
30
TraesCS7A02G347100
99
TraesCS7D02G331900
TraesCS7B02G236200
69
76
TraesCS7A02G346500
35
TraesCS7B02G236600
TraesCS7D02G332500
25
21
TraesCS7D02G332600
32
TraesCS7A02G346700
98
TraesCS7D02G332200
23
TraesCS7B02G236000
TraesCS7D02G331800
99
TraesCS7A02G347200
2
TraesCS7D02G332700
99
TraesCS7A02G346400
59
TraesCS7B02G236700
TraesCS7D02G141500
TraesCS2A02G156600
37
TraesCS2B02G182100
99
TraesCS2D02G162400
0
81
TraesCS5A02G271600
99
TraesCS5D02G279300
TraesCS5B02G271900
50
TraesCS6A02G095600
88
TraesCS6D02G086300
84
TraesCS6B02G123700
34
TraesCS6A02G095500
93
TraesCS6B02G123500
TraesCS7B02G235200
25
99
TraesCS7D02G330900
0
TraesCS7A02G348100
TraesCS7B02G255400
65
TraesCS7D02G349700
TraesCS7D02G350200
99
TraesCS7B02G254300
65
TraesCS7B02G253800
TraesCS7A02G373200
TraesCS7A02G372900

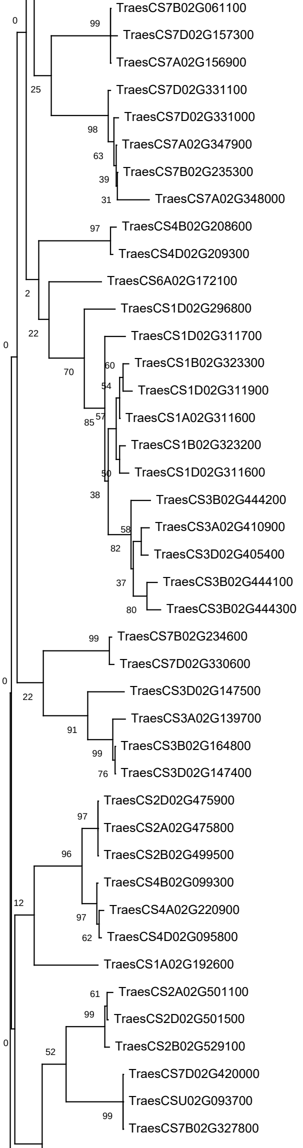
TraesCS7B02G061100
TraesCS7D02G157300
TraesCS7A02G156900
TraesCS7D02G331100
TraesCS7D02G331000
TraesCS7A02G347900
TraesCS7B02G235300
TraesCS7A02G348000
TraesCS4B02G208600
TraesCS4D02G209300
TraesCS6A02G172100
TraesCS1D02G296800
TraesCS1D02G311700
TraesCS1B02G323300
TraesCS1D02G311900
TraesCS1A02G311600
TraesCS1B02G323200
TraesCS1D02G311600
TraesCS3B02G444200
TraesCS3A02G410900
TraesCS3D02G405400
TraesCS3B02G444100
TraesCS3B02G444300
TraesCS7B02G234600
TraesCS7D02G330600
TraesCS3D02G147500
TraesCS3A02G139700
TraesCS3B02G164800
TraesCS3D02G147400
TraesCS2D02G475900
TraesCS2A02G475800
TraesCS2B02G499500
TraesCS4B02G099300
TraesCS4A02G220900
TraesCS4D02G095800
TraesCS1A02G192600
TraesCS2A02G501100
TraesCS2D02G501500
TraesCS2B02G529100
TraesCS7D02G420000
TraesCSU02G093700
TraesCS7B02G327800

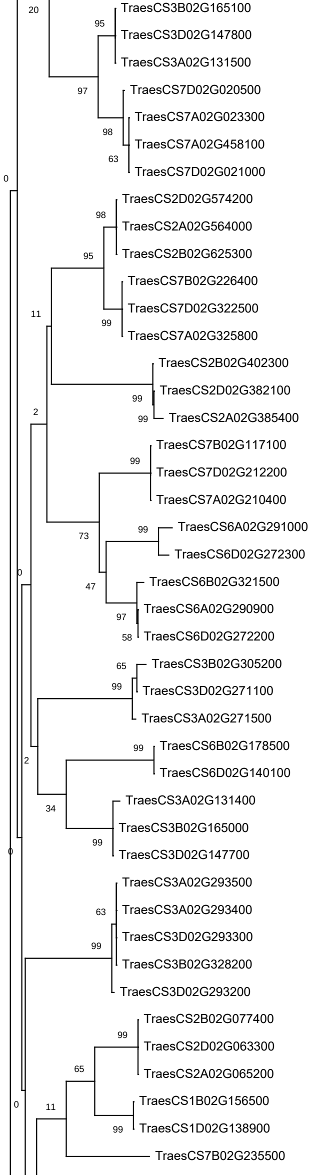
20
95
TraesCS3B02G165100
TraesCS3D02G147800
TraesCS3A02G131500
97
TraesCS7D02G020500
TraesCS7A02G023300
98
TraesCS7A02G458100
63
TraesCS7D02G021000
0
TraesCS2D02G574200
98
TraesCS2A02G564000
95
TraesCS2B02G625300
TraesCS7B02G226400
11
TraesCS7D02G322500
99
TraesCS7A02G325800
TraesCS2B02G402300
TraesCS2D02G382100
99
2
99
TraesCS2A02G385400
TraesCS7B02G117100
99
TraesCS7D02G212200
TraesCS7A02G210400
99
TraesCS6A02G291000
73
TraesCS6D02G272300
0
TraesCS6B02G321500
47
TraesCS6A02G290900
97
58
TraesCS6D02G272200
65
TraesCS3B02G305200
99
TraesCS3D02G271100
TraesCS3A02G271500
99
TraesCS6B02G178500
2
TraesCS6D02G140100
TraesCS3A02G131400
34
TraesCS3B02G165000
99
0
TraesCS3D02G147700
TraesCS3A02G293500
63
TraesCS3A02G293400
TraesCS3D02G293300
99
TraesCS3B02G328200
TraesCS3D02G293200
TraesCS2B02G077400
99
TraesCS2D02G063300
65
TraesCS2A02G065200
TraesCS1B02G156500
0
11
99
TraesCS1D02G138900
TraesCS7B02G235500

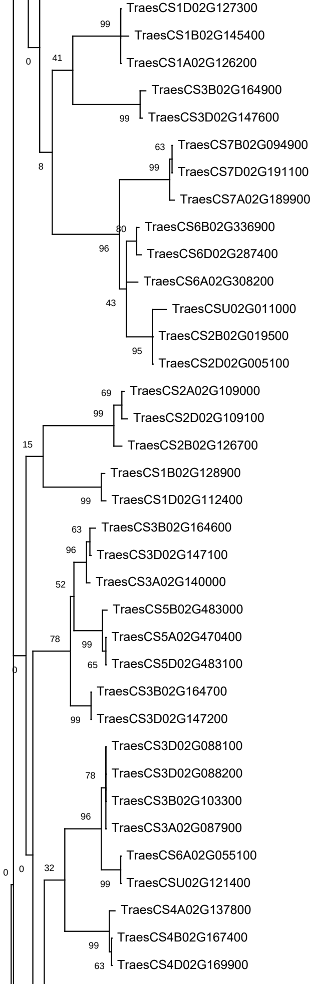
TraesCS1D02G127300
TraesCS1B02G145400
TraesCS1A02G126200
TraesCS3B02G164900
TraesCS3D02G147600
TraesCS7B02G094900
TraesCS7D02G191100
TraesCS7A02G189900
TraesCS6B02G336900
TraesCS6D02G287400
TraesCS6A02G308200
TraesCSU02G011000
TraesCS2B02G019500
TraesCS2D02G005100
TraesCS2A02G109000
TraesCS2D02G109100
TraesCS2B02G126700
TraesCS1B02G128900
TraesCS1D02G112400
TraesCS3B02G164600
TraesCS3D02G147100
TraesCS3A02G140000
TraesCS5B02G483000
TraesCS5A02G470400
TraesCS5D02G483100
TraesCS3B02G164700
TraesCS3D02G147200
TraesCS3D02G088100
TraesCS3D02G088200
TraesCS3B02G103300
TraesCS3A02G087900
TraesCS6A02G055100
TraesCSU02G121400
TraesCS4A02G137800
TraesCS4B02G167400
TraesCS4D02G169900

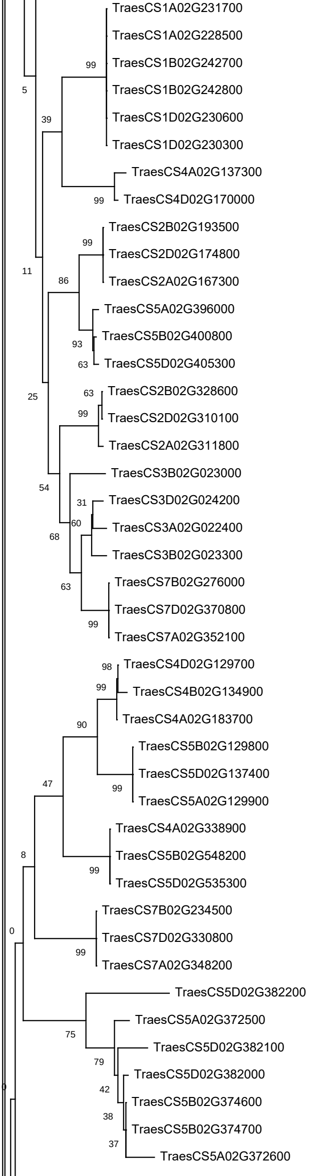
TraesCS1A02G231700
TraesCS1A02G228500
TraesCS1B02G242700
TraesCS1B02G242800
TraesCS1D02G230600
TraesCS1D02G230300
TraesCS4A02G137300
TraesCS4D02G170000
TraesCS2B02G193500
TraesCS2D02G174800
TraesCS2A02G167300
TraesCS5A02G396000
TraesCS5B02G400800
TraesCS5D02G405300
TraesCS2B02G328600
TraesCS2D02G310100
TraesCS2A02G311800
TraesCS3B02G023000
TraesCS3D02G024200
TraesCS3A02G022400
TraesCS3B02G023300
TraesCS7B02G276000
TraesCS7D02G370800
TraesCS7A02G352100
TraesCS4D02G129700
TraesCS4B02G134900
TraesCS4A02G183700
TraesCS5B02G129800
TraesCS5D02G137400
TraesCS5A02G129900
TraesCS4A02G338900
TraesCS5B02G548200
TraesCS5D02G535300
TraesCS7B02G234500
TraesCS7D02G330800
TraesCS7A02G348200
TraesCS5D02G382200
TraesCS5A02G372500
TraesCS5D02G382100
TraesCS5D02G382000
TraesCS5B02G374600
TraesCS5B02G374700
TraesCS5A02G372600

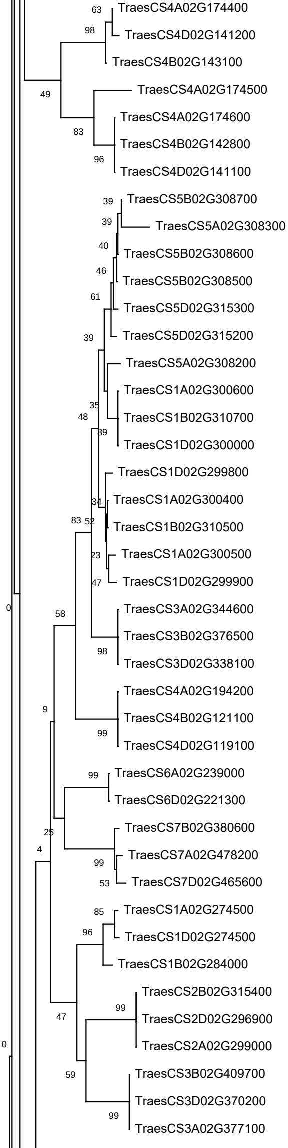
TraesCS4A02G174400
TraesCS4D02G141200
TraesCS4B02G143100
TraesCS4A02G174500
TraesCS4A02G174600
TraesCS4B02G142800
TraesCS4D02G141100
TraesCS5B02G308700
TraesCS5A02G308300
TraesCS5B02G308600
TraesCS5B02G308500
TraesCS5D02G315300
TraesCS5D02G315200
TraesCS5A02G308200
TraesCS1A02G300600
TraesCS1B02G310700
TraesCS1D02G300000
TraesCS1D02G299800
TraesCS1A02G300400
TraesCS1B02G310500
TraesCS1A02G300500
TraesCS1D02G299900
TraesCS3A02G344600
TraesCS3B02G376500
TraesCS3D02G338100
TraesCS4A02G194200
TraesCS4B02G121100
TraesCS4D02G119100
TraesCS6A02G239000
TraesCS6D02G221300
TraesCS7B02G380600
TraesCS7A02G478200
TraesCS7D02G465600
TraesCS1A02G274500
TraesCS1D02G274500
TraesCS1B02G284000
TraesCS2B02G315400
TraesCS2D02G296900
TraesCS2A02G299000
TraesCS3B02G409700
TraesCS3D02G370200
TraesCS3A02G377100

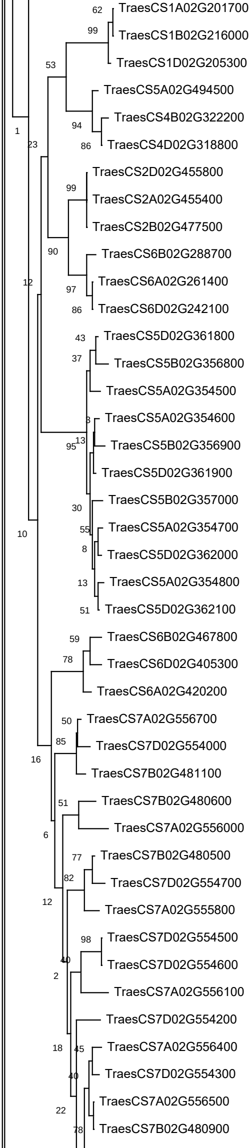
TraesCS1A02G201700
TraesCS1B02G216000
TraesCS1D02G205300
TraesCS5A02G494500
TraesCS4B02G322200
TraesCS4D02G318800
TraesCS2D02G455800
TraesCS2A02G455400
TraesCS2B02G477500
TraesCS6B02G288700
TraesCS6A02G261400
TraesCS6D02G242100
TraesCS5D02G361800
TraesCS5B02G356800
TraesCS5A02G354500
TraesCS5A02G354600
TraesCS5B02G356900
TraesCS5D02G361900
TraesCS5B02G357000
TraesCS5A02G354700
TraesCS5D02G362000
TraesCS5A02G354800
TraesCS5D02G362100
TraesCS6B02G467800
TraesCS6D02G405300
TraesCS6A02G420200
TraesCS7A02G556700
TraesCS7D02G554000
TraesCS7B02G481100
TraesCS7B02G480600
TraesCS7A02G556000
TraesCS7B02G480500
TraesCS7D02G554700
TraesCS7A02G555800
TraesCS7D02G554500
TraesCS7D02G554600
TraesCS7A02G556100
TraesCS7D02G554200
TraesCS7A02G556400
TraesCS7D02G554300
TraesCS7A02G556500
TraesCS7B02G480900

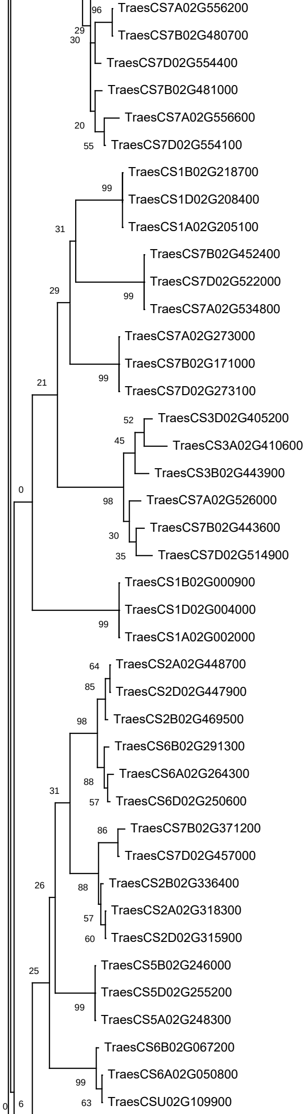
96
TraesCS7A02G556200
29
30
TraesCS7B02G480700
TraesCS7D02G554400
TraesCS7B02G481000
TraesCS7A02G556600
20
55
TraesCS7D02G554100
TraesCS1B02G218700
99
TraesCS1D02G208400
31
TraesCS1A02G205100
TraesCS7B02G452400
TraesCS7D02G522000
29
99
TraesCS7A02G534800
TraesCS7A02G273000
TraesCS7B02G171000
21
99
TraesCS7D02G273100
52
TraesCS3D02G405200
45
TraesCS3A02G410600
TraesCS3B02G443900
0
98
TraesCS7A02G526000
TraesCS7B02G443600
30
35
TraesCS7D02G514900
TraesCS1B02G000900
TraesCS1D02G004000
99
TraesCS1A02G002000
64
TraesCS2A02G448700
85
TraesCS2D02G447900
98
TraesCS2B02G469500
TraesCS6B02G291300
88
TraesCS6A02G264300
31
57
TraesCS6D02G250600
86
TraesCS7B02G371200
TraesCS7D02G457000
26
88
TraesCS2B02G336400
57
TraesCS2A02G318300
60
TraesCS2D02G315900
TraesCS5B02G246000
25
TraesCS5D02G255200
99
TraesCS5A02G248300
TraesCS6B02G067200
TraesCS6A02G050800
99
0
6
63
TraesCSU02G109900

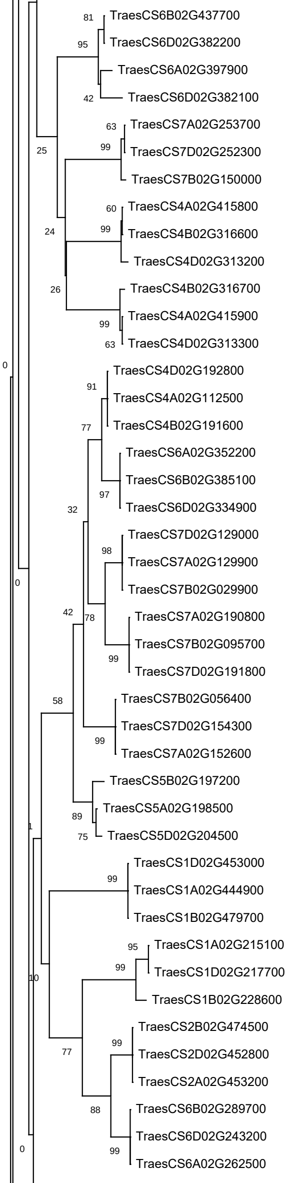
81
TraesCS6B02G437700
95
TraesCS6D02G382200
TraesCS6A02G397900
42
TraesCS6D02G382100
63
TraesCS7A02G253700
99
TraesCS7D02G252300
25
TraesCS7B02G150000
60
TraesCS4A02G415800
24
99
TraesCS4B02G316600
TraesCS4D02G313200
26
TraesCS4B02G316700
TraesCS4A02G415900
99
63
TraesCS4D02G313300
0
TraesCS4D02G192800
91
TraesCS4A02G112500
77
TraesCS4B02G191600
TraesCS6A02G352200
TraesCS6B02G385100
97
32
TraesCS6D02G334900
TraesCS7D02G129000
98
TraesCS7A02G129900
0
TraesCS7B02G029900
42
78
TraesCS7A02G190800
TraesCS7B02G095700
99
TraesCS7D02G191800
58
TraesCS7B02G056400
TraesCS7D02G154300
99
TraesCS7A02G152600
TraesCS5B02G197200
TraesCS5A02G198500
89
1
75
TraesCS5D02G204500
TraesCS1D02G453000
99
TraesCS1A02G444900
TraesCS1B02G479700
95
TraesCS1A02G215100
99
TraesCS1D02G217700
10
TraesCS1B02G228600
TraesCS2B02G474500
99
77
TraesCS2D02G452800
TraesCS2A02G453200
88
TraesCS6B02G289700
TraesCS6D02G243200
0
99
TraesCS6A02G262500

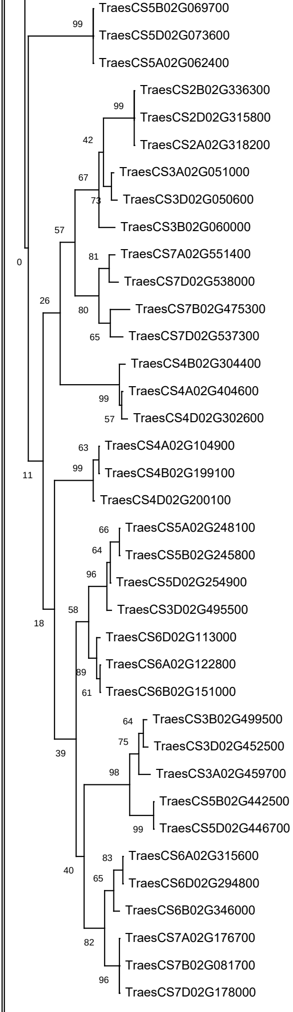

TraesCS5B02G069700
99
TraesCS5D02G073600
TraesCS5A02G062400
TraesCS2B02G336300
99
TraesCS2D02G315800
42
TraesCS2A02G318200
TraesCS3A02G051000
67
73
TraesCS3D02G050600
TraesCS3B02G060000
57
81
TraesCS7A02G551400
0
TraesCS7D02G538000
26
80
TraesCS7B02G475300
65
TraesCS7D02G537300
TraesCS4B02G304400
TraesCS4A02G404600
99
57
TraesCS4D02G302600
63
TraesCS4A02G104900
99
TraesCS4B02G199100
11
TraesCS4D02G200100
66
TraesCS5A02G248100
64
TraesCS5B02G245800
96
TraesCS5D02G254900
58
TraesCS3D02G495500
18
TraesCS6D02G113000
TraesCS6A02G122800
89
61
TraesCS6B02G151000
64
TraesCS3B02G499500
75
TraesCS3D02G452500
39
98
TraesCS3A02G459700
TraesCS5B02G442500
99
TraesCS5D02G446700
83
TraesCS6A02G315600
40
65
TraesCS6D02G294800
TraesCS6B02G346000
82
TraesCS7A02G176700
TraesCS7B02G081700
96
TraesCS7D02G178000

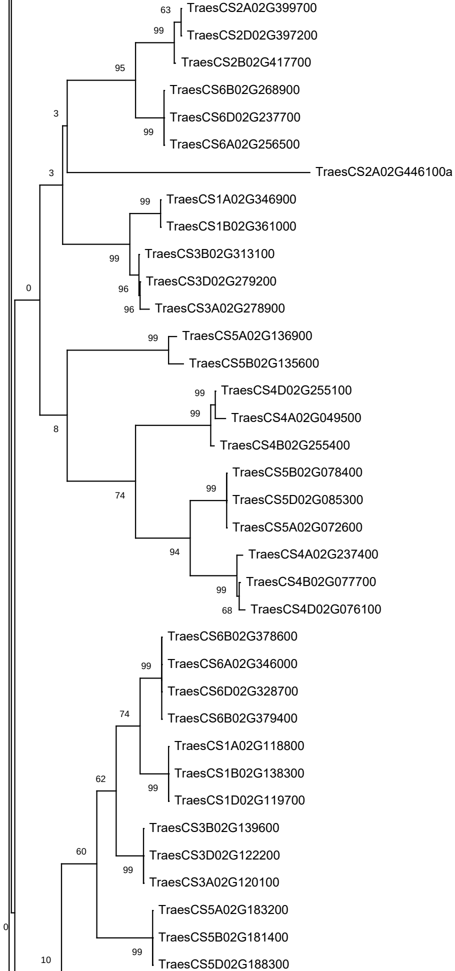
TraesCS2A02G399700
TraesCS2D02G397200
TraesCS2B02G417700
TraesCS6B02G268900
TraesCS6D02G237700
TraesCS6A02G256500
TraesCS2A02G446100a
TraesCS1A02G346900
TraesCS1B02G361000
TraesCS3B02G313100
TraesCS3D02G279200
TraesCS3A02G278900
TraesCS5A02G136900
TraesCS5B02G135600
TraesCS4D02G255100
TraesCS4A02G049500
TraesCS4B02G255400
TraesCS5B02G078400
TraesCS5D02G085300
TraesCS5A02G072600
TraesCS4A02G237400
TraesCS4B02G077700
TraesCS4D02G076100
TraesCS6B02G378600
TraesCS6A02G346000
TraesCS6D02G328700
TraesCS6B02G379400
TraesCS1A02G118800
TraesCS1B02G138300
TraesCS1D02G119700
TraesCS3B02G139600
TraesCS3D02G122200
TraesCS3A02G120100
TraesCS5A02G183200
TraesCS5B02G181400
TraesCS5D02G188300

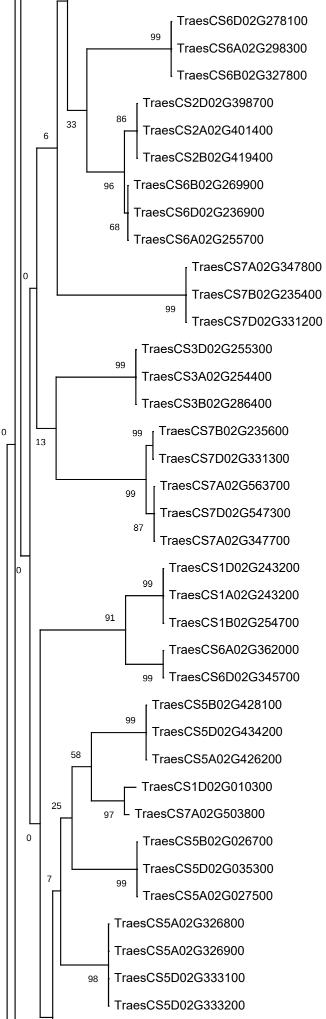

TraesCS6D02G278100
TraesCS6A02G298300
TraesCS6B02G327800
TraesCS2D02G398700
TraesCS2A02G401400
TraesCS2B02G419400
TraesCS6B02G269900
TraesCS6D02G236900
TraesCS6A02G255700
TraesCS7A02G347800
TraesCS7B02G235400
TraesCS7D02G331200
TraesCS3D02G255300
TraesCS3A02G254400
TraesCS3B02G286400
TraesCS7B02G235600
TraesCS7D02G331300
TraesCS7A02G563700
TraesCS7D02G547300
TraesCS7A02G347700
TraesCS1D02G243200
TraesCS1A02G243200
TraesCS1B02G254700
TraesCS6A02G362000
TraesCS6D02G345700
TraesCS5B02G428100
TraesCS5D02G434200
TraesCS5A02G426200
TraesCS1D02G010300
TraesCS7A02G503800
TraesCS5B02G026700
TraesCS5D02G035300
TraesCS5A02G027500
TraesCS5A02G326800
TraesCS5A02G326900
TraesCS5D02G333100
TraesCS5D02G333200
99
33
86
6
96
68
0
99
99
0
13
99
99
87
0
99
91
99
99
58
25
97
0
7
99
98

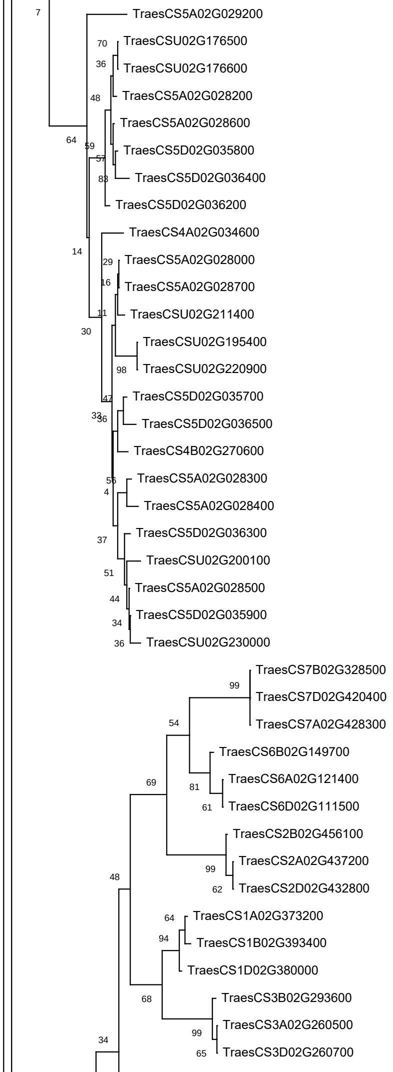

TraesCS5A02G029200
TraesCSU02G176500
TraesCSU02G176600
TraesCS5A02G028200
TraesCS5A02G028600
TraesCS5D02G035800
TraesCS5D02G036400
TraesCS5D02G036200
TraesCS4A02G034600
TraesCS5A02G028000
TraesCS5A02G028700
TraesCSU02G211400
TraesCSU02G195400
TraesCSU02G220900
TraesCS5D02G035700
TraesCS5D02G036500
TraesCS4B02G270600
TraesCS5A02G028300
TraesCS5A02G028400
TraesCS5D02G036300
TraesCSU02G200100
TraesCS5A02G028500
TraesCS5D02G035900
TraesCSU02G230000
TraesCS7B02G328500
TraesCS7D02G420400
TraesCS7A02G428300
TraesCS6B02G149700
TraesCS6A02G121400
TraesCS6D02G111500
TraesCS2B02G456100
TraesCS2A02G437200
TraesCS2D02G432800
TraesCS1A02G373200
TraesCS1B02G393400
TraesCS1D02G380000
TraesCS3B02G293600
TraesCS3A02G260500
TraesCS3D02G260700

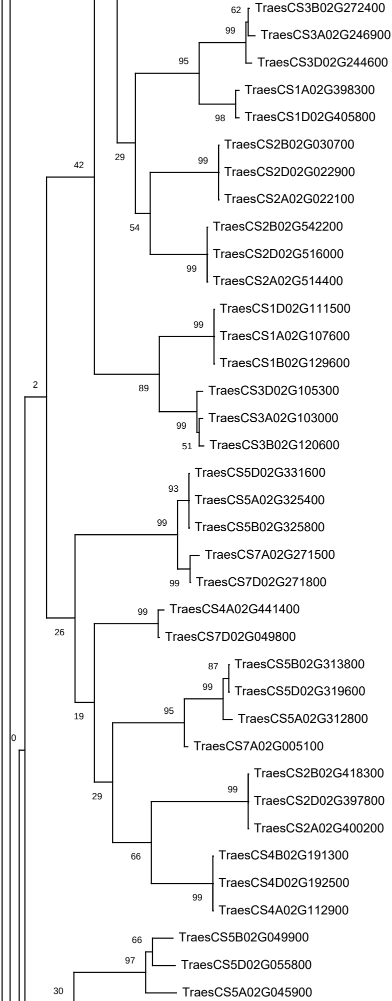
62
TraesCS3B02G272400
99
TraesCS3A02G246900
95
TraesCS3D02G244600
TraesCS1A02G398300
98
TraesCS1D02G405800
42
29
TraesCS2B02G030700
99
TraesCS2D02G022900
TraesCS2A02G022100
54
TraesCS2B02G542200
TraesCS2D02G516000
99
TraesCS2A02G514400
TraesCS1D02G111500
99
TraesCS1A02G107600
TraesCS1B02G129600
2
89
TraesCS3D02G105300
TraesCS3A02G103000
99
51
TraesCS3B02G120600
TraesCS5D02G331600
93
TraesCS5A02G325400
99
TraesCS5B02G325800
TraesCS7A02G271500
99
TraesCS7D02G271800
99
TraesCS4A02G441400
26
TraesCS7D02G049800
87
TraesCS5B02G313800
99
TraesCS5D02G319600
19
95
TraesCS5A02G312800
0
TraesCS7A02G005100
TraesCS2B02G418300
99
29
TraesCS2D02G397800
TraesCS2A02G400200
66
TraesCS4B02G191300
TraesCS4D02G192500
99
TraesCS4A02G112900
66
TraesCS5B02G049900
97
TraesCS5D02G055800
30
TraesCS5A02G045900

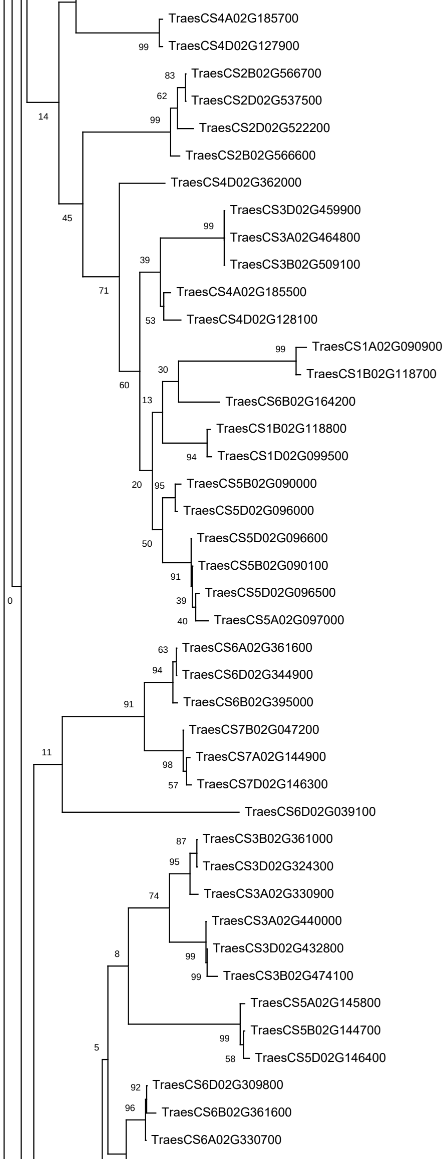

TraesCS4A02G185700
99
TraesCS4D02G127900
83
TraesCS2B02G566700
62
TraesCS2D02G537500
14
99
TraesCS2D02G522200
TraesCS2B02G566600
TraesCS4D02G362000
45
TraesCS3D02G459900
99
TraesCS3A02G464800
TraesCS3B02G509100
39
71
TraesCS4A02G185500
53
TraesCS4D02G128100
99
TraesCS1A02G090900
30
TraesCS1B02G118700
60
13
TraesCS6B02G164200
TraesCS1B02G118800
94
TraesCS1D02G099500
20
95
TraesCS5B02G090000
TraesCS5D02G096000
50
TraesCS5D02G096600
TraesCS5B02G090100
91
TraesCS5D02G096500
0
39
40
TraesCS5A02G097000
63
TraesCS6A02G361600
94
TraesCS6D02G344900
TraesCS6B02G395000
91
TraesCS7B02G047200
11
TraesCS7A02G144900
98
57
TraesCS7D02G146300
TraesCS6D02G039100
87
TraesCS3B02G361000
95
TraesCS3D02G324300
TraesCS3A02G330900
74
TraesCS3A02G440000
TraesCS3D02G432800
8
99
99
TraesCS3B02G474100
TraesCS5A02G145800
TraesCS5B02G144700
99
5
58
TraesCS5D02G146400
92
TraesCS6D02G309800
96
TraesCS6B02G361600
TraesCS6A02G330700

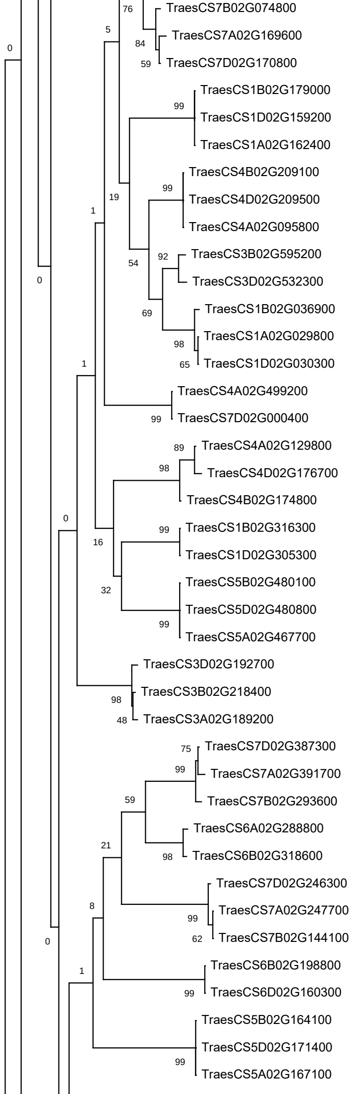

TraesCS7B02G074800
TraesCS7A02G169600
TraesCS7D02G170800
TraesCS1B02G179000
TraesCS1D02G159200
TraesCS1A02G162400
TraesCS4B02G209100
TraesCS4D02G209500
TraesCS4A02G095800
TraesCS3B02G595200
TraesCS3D02G532300
TraesCS1B02G036900
TraesCS1A02G029800
TraesCS1D02G030300
TraesCS4A02G499200
TraesCS7D02G000400
TraesCS4A02G129800
TraesCS4D02G176700
TraesCS4B02G174800
TraesCS1B02G316300
TraesCS1D02G305300
TraesCS5B02G480100
TraesCS5D02G480800
TraesCS5A02G467700
TraesCS3D02G192700
TraesCS3B02G218400
TraesCS3A02G189200
TraesCS7D02G387300
TraesCS7A02G391700
TraesCS7B02G293600
TraesCS6A02G288800
TraesCS6B02G318600
TraesCS7D02G246300
TraesCS7A02G247700
TraesCS7B02G144100
TraesCS6B02G198800
TraesCS6D02G160300
TraesCS5B02G164100
TraesCS5D02G171400
TraesCS5A02G167100

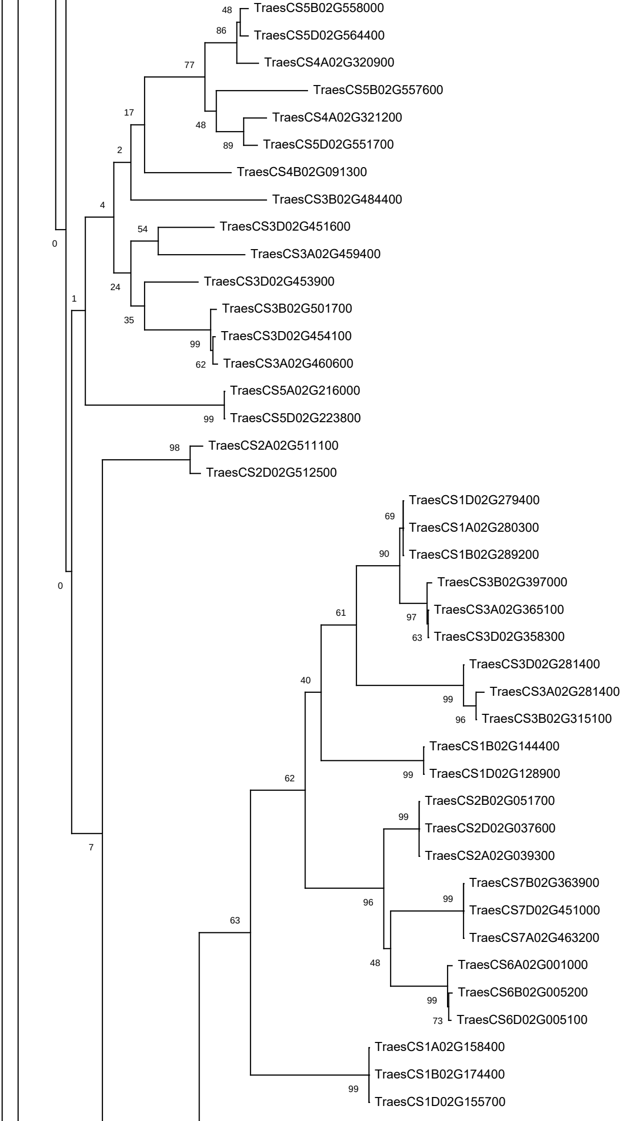
TraesCS5B02G558000
TraesCS5D02G564400
TraesCS4A02G320900
TraesCS5B02G557600
TraesCS4A02G321200
TraesCS5D02G551700
TraesCS4B02G091300
TraesCS3B02G484400
TraesCS3D02G451600
TraesCS3A02G459400
TraesCS3D02G453900
TraesCS3B02G501700
TraesCS3D02G454100
TraesCS3A02G460600
TraesCS5A02G216000
TraesCS5D02G223800
TraesCS2A02G511100
TraesCS2D02G512500
TraesCS1D02G279400
TraesCS1A02G280300
TraesCS1B02G289200
TraesCS3B02G397000
TraesCS3A02G365100
TraesCS3D02G358300
TraesCS3D02G281400
TraesCS3A02G281400
TraesCS3B02G315100
TraesCS1B02G144400
TraesCS1D02G128900
TraesCS2B02G051700
TraesCS2D02G037600
TraesCS2A02G039300
TraesCS7B02G363900
TraesCS7D02G451000
TraesCS7A02G463200
TraesCS6A02G001000
TraesCS6B02G005200
TraesCS6D02G005100
TraesCS1A02G158400
TraesCS1B02G174400
TraesCS1D02G155700

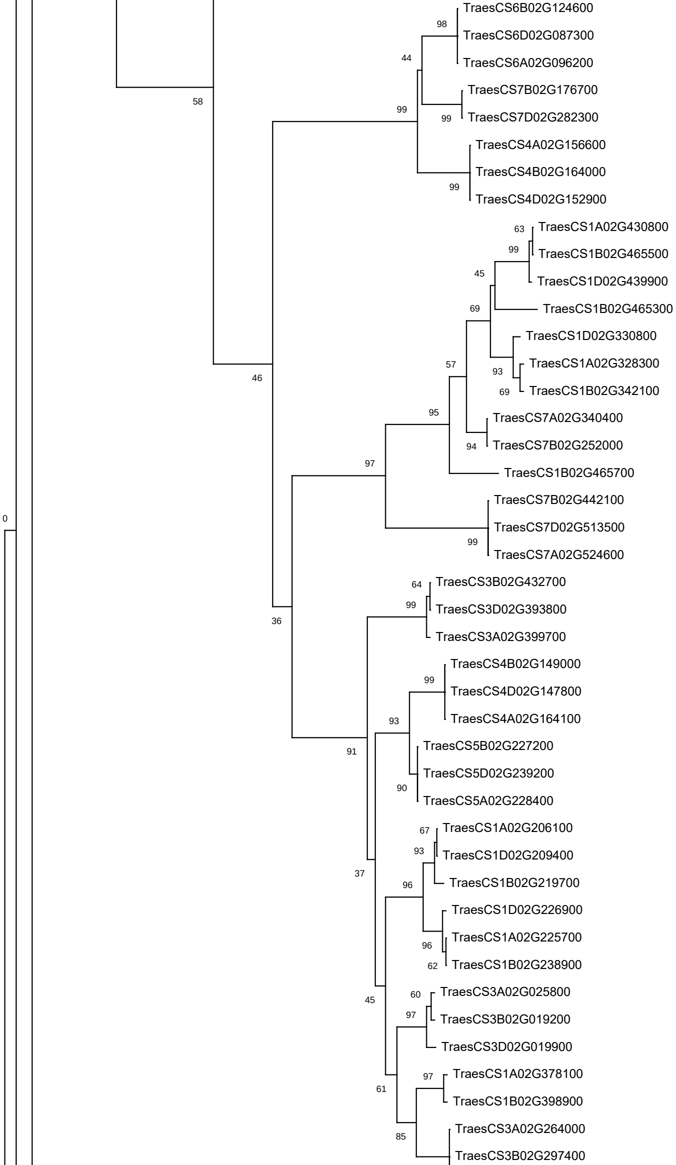

TraesCS6B02G124600
TraesCS6D02G087300
TraesCS6A02G096200
TraesCS7B02G176700
TraesCS7D02G282300
TraesCS4A02G156600
TraesCS4B02G164000
TraesCS4D02G152900
TraesCS1A02G430800
TraesCS1B02G465500
TraesCS1D02G439900
TraesCS1B02G465300
TraesCS1D02G330800
TraesCS1A02G328300
TraesCS1B02G342100
TraesCS7A02G340400
TraesCS7B02G252000
TraesCS1B02G465700
TraesCS7B02G442100
TraesCS7D02G513500
TraesCS7A02G524600
TraesCS3B02G432700
TraesCS3D02G393800
TraesCS3A02G399700
TraesCS4B02G149000
TraesCS4D02G147800
TraesCS4A02G164100
TraesCS5B02G227200
TraesCS5D02G239200
TraesCS5A02G228400
TraesCS1A02G206100
TraesCS1D02G209400
TraesCS1B02G219700
TraesCS1D02G226900
TraesCS1A02G225700
TraesCS1B02G238900
TraesCS3A02G025800
TraesCS3B02G019200
TraesCS3D02G019900
TraesCS1A02G378100
TraesCS1B02G398900
TraesCS3A02G264000
TraesCS3B02G297400

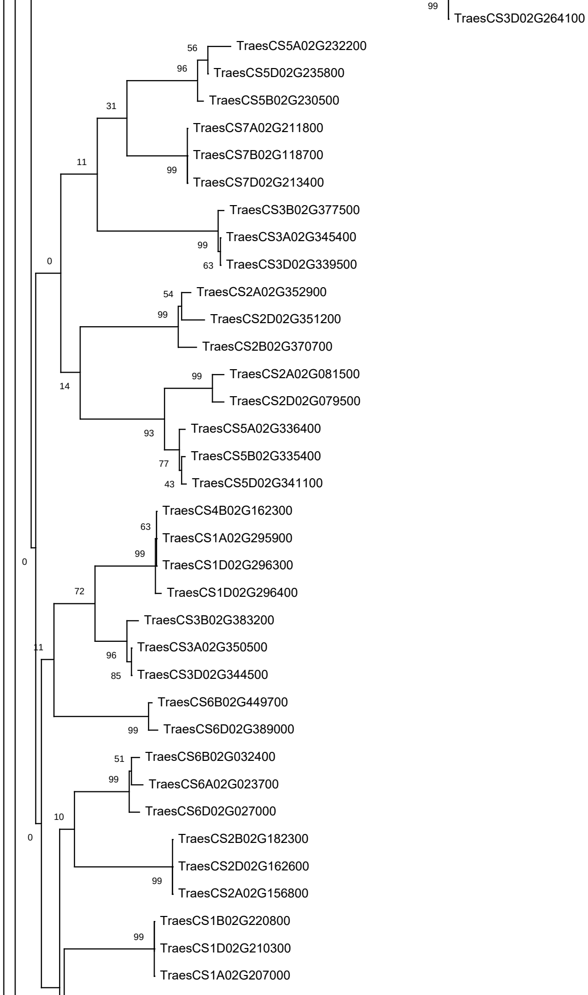
99
TraesCS3D02G264100
56
TraesCS5A02G232200
96
TraesCS5D02G235800
TraesCS5B02G230500
31
TraesCS7A02G211800
TraesCS7B02G118700
11
99
TraesCS7D02G213400
TraesCS3B02G377500
TraesCS3A02G345400
99
63
TraesCS3D02G339500
0
54
TraesCS2A02G352900
99
TraesCS2D02G351200
TraesCS2B02G370700
99
TraesCS2A02G081500
14
TraesCS2D02G079500
TraesCS5A02G336400
93
TraesCS5B02G335400
77
43
TraesCS5D02G341100
TraesCS4B02G162300
63
TraesCS1A02G295900
99
0
TraesCS1D02G296300
TraesCS1D02G296400
72
TraesCS3B02G383200
11
TraesCS3A02G350500
96
85
TraesCS3D02G344500
TraesCS6B02G449700
99
TraesCS6D02G389000
51
TraesCS6B02G032400
99
TraesCS6A02G023700
TraesCS6D02G027000
10
0
TraesCS2B02G182300
TraesCS2D02G162600
99
TraesCS2A02G156800
TraesCS1B02G220800
99
TraesCS1D02G210300
TraesCS1A02G207000

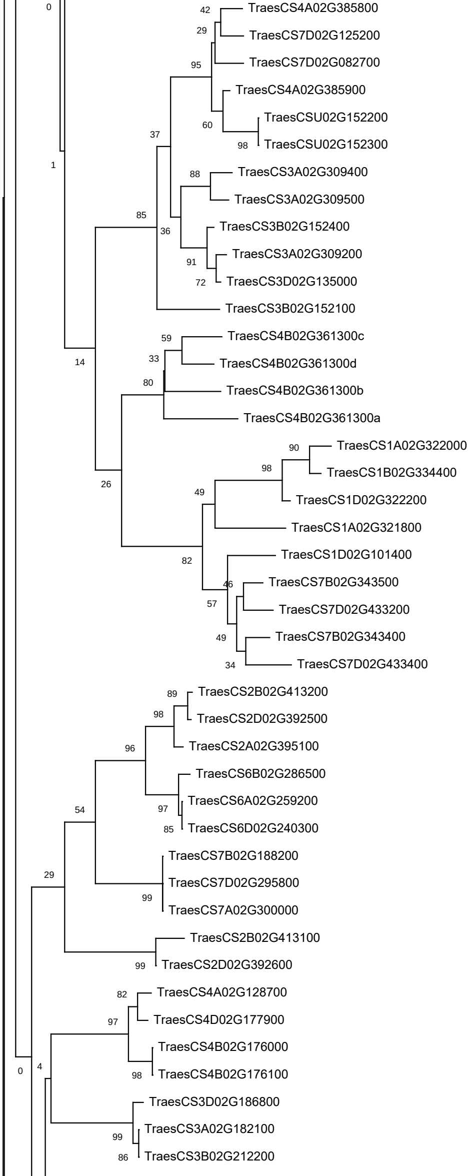
0
42
TraesCS4A02G385800
29
TraesCS7D02G125200
TraesCS7D02G082700
95
TraesCS4A02G385900
TraesCSU02G152200
60
37
98
TraesCSU02G152300
1
88
TraesCS3A02G309400
TraesCS3A02G309500
85
TraesCS3B02G152400
36
TraesCS3A02G309200
91
72
TraesCS3D02G135000
TraesCS3B02G152100
59
TraesCS4B02G361300c
14
33
TraesCS4B02G361300d
80
TraesCS4B02G361300b
TraesCS4B02G361300a
90
TraesCS1A02G322000
98
TraesCS1B02G334400
26
49
TraesCS1D02G322200
TraesCS1A02G321800
TraesCS1D02G101400
82
TraesCS7B02G343500
46
57
TraesCS7D02G433200
TraesCS7B02G343400
49
34
TraesCS7D02G433400
89
TraesCS2B02G413200
98
TraesCS2D02G392500
TraesCS2A02G395100
96
TraesCS6B02G286500
TraesCS6A02G259200
97
54
85
TraesCS6D02G240300
TraesCS7B02G188200
29
TraesCS7D02G295800
99
TraesCS7A02G300000
TraesCS2B02G413100
99
TraesCS2D02G392600
82
TraesCS4A02G128700
97
TraesCS4D02G177900
TraesCS4B02G176000
0
4
98
TraesCS4B02G176100
TraesCS3D02G186800
TraesCS3A02G182100
99
86
TraesCS3B02G212200

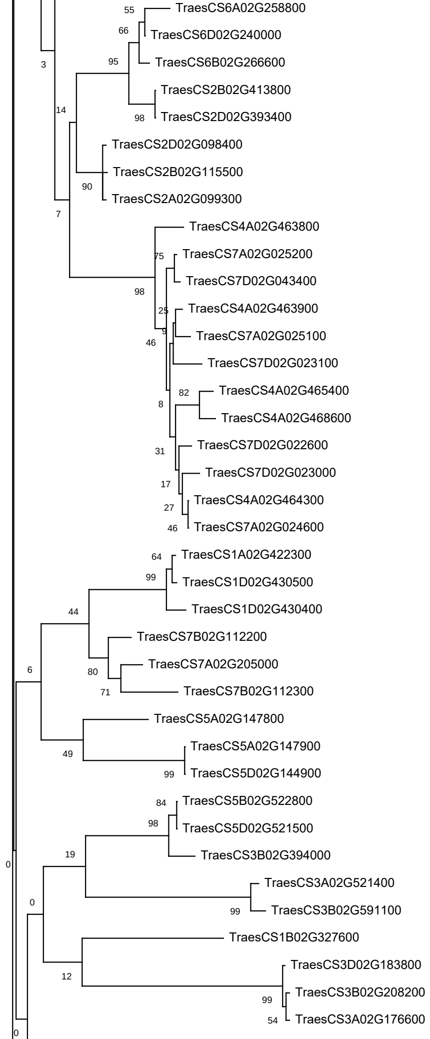
TraesCS6A02G258800
TraesCS6D02G240000
TraesCS6B02G266600
TraesCS2B02G413800
TraesCS2D02G393400
TraesCS2D02G098400
TraesCS2B02G115500
TraesCS2A02G099300
TraesCS4A02G463800
TraesCS7A02G025200
TraesCS7D02G043400
TraesCS4A02G463900
TraesCS7A02G025100
TraesCS7D02G023100
TraesCS4A02G465400
TraesCS4A02G468600
TraesCS7D02G022600
TraesCS7D02G023000
TraesCS4A02G464300
TraesCS7A02G024600
TraesCS1A02G422300
TraesCS1D02G430500
TraesCS1D02G430400
TraesCS7B02G112200
TraesCS7A02G205000
TraesCS7B02G112300
TraesCS5A02G147800
TraesCS5A02G147900
TraesCS5D02G144900
TraesCS5B02G522800
TraesCS5D02G521500
TraesCS3B02G394000
TraesCS3A02G521400
TraesCS3B02G591100
TraesCS1B02G327600
TraesCS3D02G183800
TraesCS3B02G208200
TraesCS3A02G176600

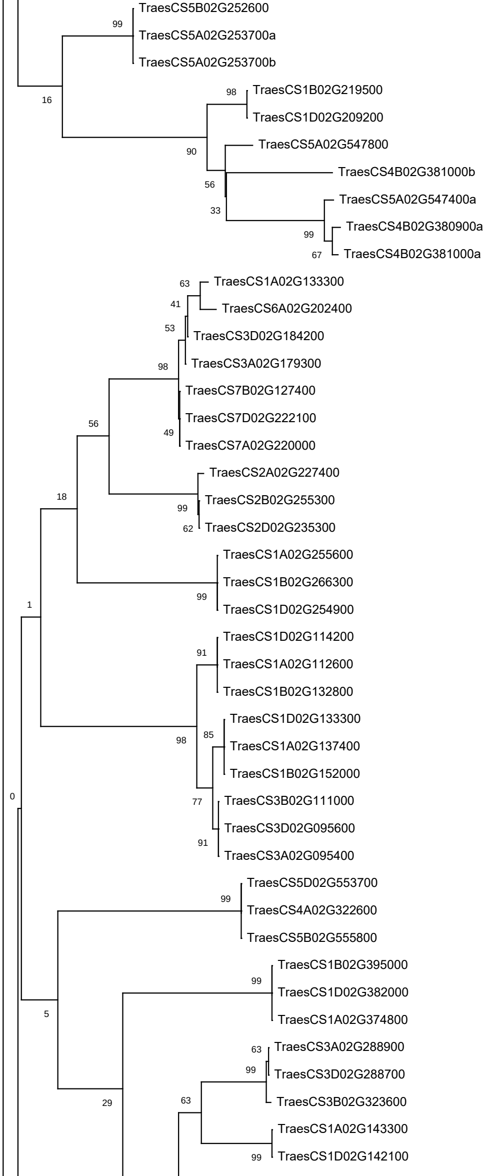
TraesCS5B02G252600
TraesCS5A02G253700a
TraesCS5A02G253700b
TraesCS1B02G219500
TraesCS1D02G209200
TraesCS5A02G547800
TraesCS4B02G381000b
TraesCS5A02G547400a
TraesCS4B02G380900a
TraesCS4B02G381000a
TraesCS1A02G133300
TraesCS6A02G202400
TraesCS3D02G184200
TraesCS3A02G179300
TraesCS7B02G127400
TraesCS7D02G222100
TraesCS7A02G220000
TraesCS2A02G227400
TraesCS2B02G255300
TraesCS2D02G235300
TraesCS1A02G255600
TraesCS1B02G266300
TraesCS1D02G254900
TraesCS1D02G114200
TraesCS1A02G112600
TraesCS1B02G132800
TraesCS1D02G133300
TraesCS1A02G137400
TraesCS1B02G152000
TraesCS3B02G111000
TraesCS3D02G095600
TraesCS3A02G095400
TraesCS5D02G553700
TraesCS4A02G322600
TraesCS5B02G555800
TraesCS1B02G395000
TraesCS1D02G382000
TraesCS1A02G374800
TraesCS3A02G288900
TraesCS3D02G288700
TraesCS3B02G323600
TraesCS1A02G143300
TraesCS1D02G142100

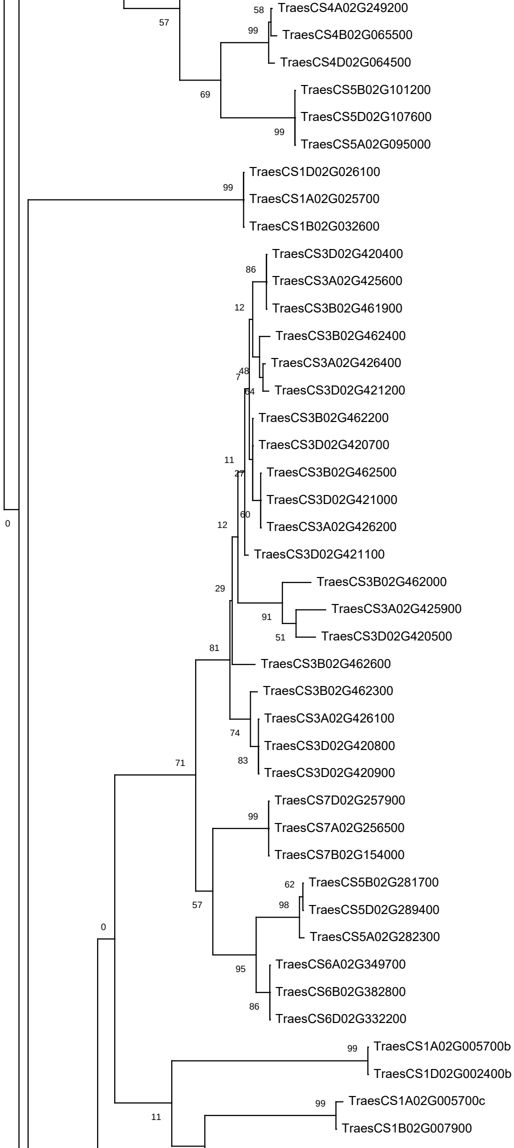
58
TraesCS4A02G249200
57
99
TraesCS4B02G065500
TraesCS4D02G064500
69
TraesCS5B02G101200
TraesCS5D02G107600
99
TraesCS5A02G095000
TraesCS1D02G026100
99
TraesCS1A02G025700
TraesCS1B02G032600
TraesCS3D02G420400
86
TraesCS3A02G425600
12
TraesCS3B02G461900
TraesCS3B02G462400
48
TraesCS3A02G426400
7
64
TraesCS3D02G421200
TraesCS3B02G462200
TraesCS3D02G420700
11
27
TraesCS3B02G462500
TraesCS3D02G421000
0
60
12
TraesCS3A02G426200
TraesCS3D02G421100
29
TraesCS3B02G462000
TraesCS3A02G425900
91
51
TraesCS3D02G420500
81
TraesCS3B02G462600
TraesCS3B02G462300
TraesCS3A02G426100
74
TraesCS3D02G420800
83
71
TraesCS3D02G420900
TraesCS7D02G257900
99
TraesCS7A02G256500
TraesCS7B02G154000
62
TraesCS5B02G281700
57
98
TraesCS5D02G289400
0
TraesCS5A02G282300
TraesCS6A02G349700
95
TraesCS6B02G382800
86
TraesCS6D02G332200
99
TraesCS1A02G005700b
TraesCS1D02G002400b
99
TraesCS1A02G005700c
11
TraesCS1B02G007900

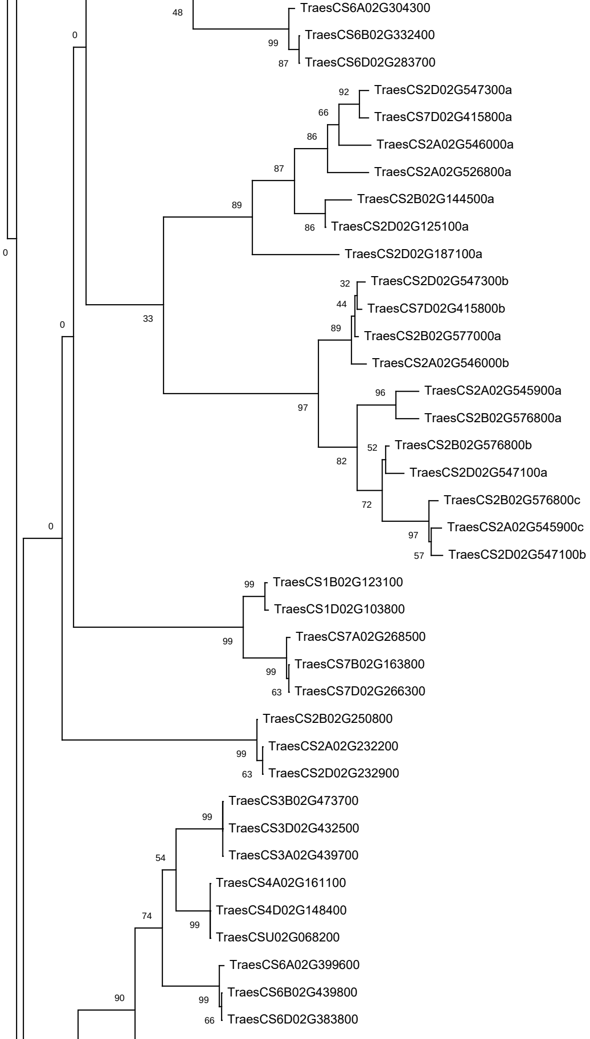
48
TraesCS6A02G304300
0
TraesCS6B02G332400
99
87
TraesCS6D02G283700
92
TraesCS2D02G547300a
66
TraesCS7D02G415800a
86
TraesCS2A02G546000a
87
TraesCS2A02G526800a
89
TraesCS2B02G144500a
86
TraesCS2D02G125100a
0
TraesCS2D02G187100a
32
TraesCS2D02G547300b
44
TraesCS7D02G415800b
33
0
89
TraesCS2B02G577000a
TraesCS2A02G546000b
96
TraesCS2A02G545900a
97
TraesCS2B02G576800a
52
TraesCS2B02G576800b
82
TraesCS2D02G547100a
72
TraesCS2B02G576800c
0
97
TraesCS2A02G545900c
57
TraesCS2D02G547100b
99
TraesCS1B02G123100
TraesCS1D02G103800
99
TraesCS7A02G268500
99
TraesCS7B02G163800
63
TraesCS7D02G266300
TraesCS2B02G250800
99
TraesCS2A02G232200
63
TraesCS2D02G232900
TraesCS3B02G473700
99
TraesCS3D02G432500
54
TraesCS3A02G439700
TraesCS4A02G161100
74
TraesCS4D02G148400
99
TraesCSU02G068200
TraesCS6A02G399600
90
99
TraesCS6B02G439800
66
TraesCS6D02G383800

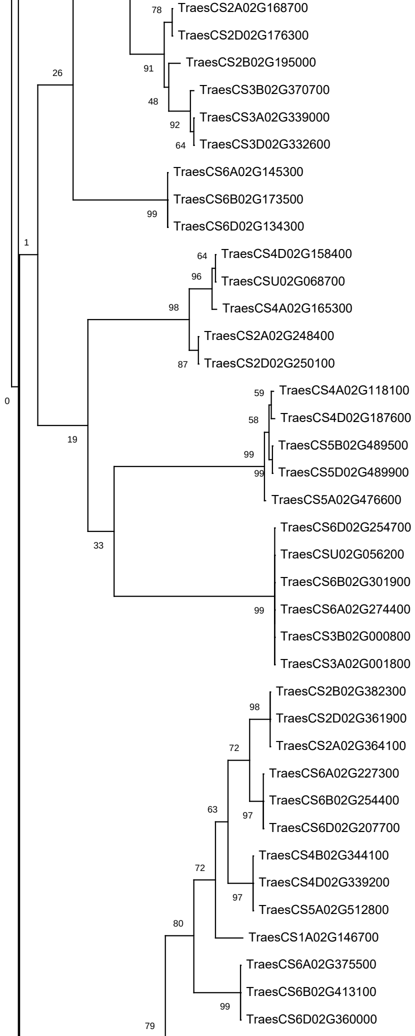

78
TraesCS2A02G168700
TraesCS2D02G176300
91
TraesCS2B02G195000
26
TraesCS3B02G370700
48
TraesCS3A02G339000
92
64
TraesCS3D02G332600
TraesCS6A02G145300
TraesCS6B02G173500
99
TraesCS6D02G134300
1
64
TraesCS4D02G158400
96
TraesCSU02G068700
98
TraesCS4A02G165300
TraesCS2A02G248400
87
TraesCS2D02G250100
59
TraesCS4A02G118100
0
58
TraesCS4D02G187600
19
TraesCS5B02G489500
99
99
TraesCS5D02G489900
TraesCS5A02G476600
TraesCS6D02G254700
33
TraesCSU02G056200
TraesCS6B02G301900
99
TraesCS6A02G274400
TraesCS3B02G000800
TraesCS3A02G001800
TraesCS2B02G382300
98
TraesCS2D02G361900
72
TraesCS2A02G364100
TraesCS6A02G227300
TraesCS6B02G254400
63
97
TraesCS6D02G207700
TraesCS4B02G344100
72
TraesCS4D02G339200
97
TraesCS5A02G512800
80
TraesCS1A02G146700
TraesCS6A02G375500
TraesCS6B02G413100
99
TraesCS6D02G360000
79

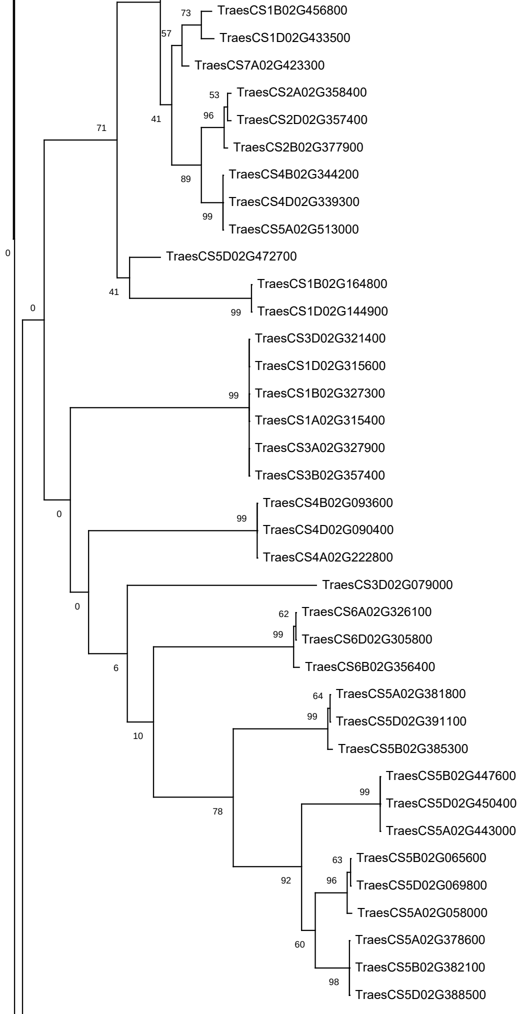

TraesCS1B02G456800
TraesCS1D02G433500
TraesCS7A02G423300
TraesCS2A02G358400
TraesCS2D02G357400
TraesCS2B02G377900
TraesCS4B02G344200
TraesCS4D02G339300
TraesCS5A02G513000
TraesCS5D02G472700
TraesCS1B02G164800
TraesCS1D02G144900
TraesCS3D02G321400
TraesCS1D02G315600
TraesCS1B02G327300
TraesCS1A02G315400
TraesCS3A02G327900
TraesCS3B02G357400
TraesCS4B02G093600
TraesCS4D02G090400
TraesCS4A02G222800
TraesCS3D02G079000
TraesCS6A02G326100
TraesCS6D02G305800
TraesCS6B02G356400
TraesCS5A02G381800
TraesCS5D02G391100
TraesCS5B02G385300
TraesCS5B02G447600
TraesCS5D02G450400
TraesCS5A02G443000
TraesCS5B02G065600
TraesCS5D02G069800
TraesCS5A02G058000
TraesCS5A02G378600
TraesCS5B02G382100
TraesCS5D02G388500
73
57
53
41
96
71
89
99
0
41
99
0
99
99
0
0
62
99
6
64
99
10
99
78
63
96
92
60
98

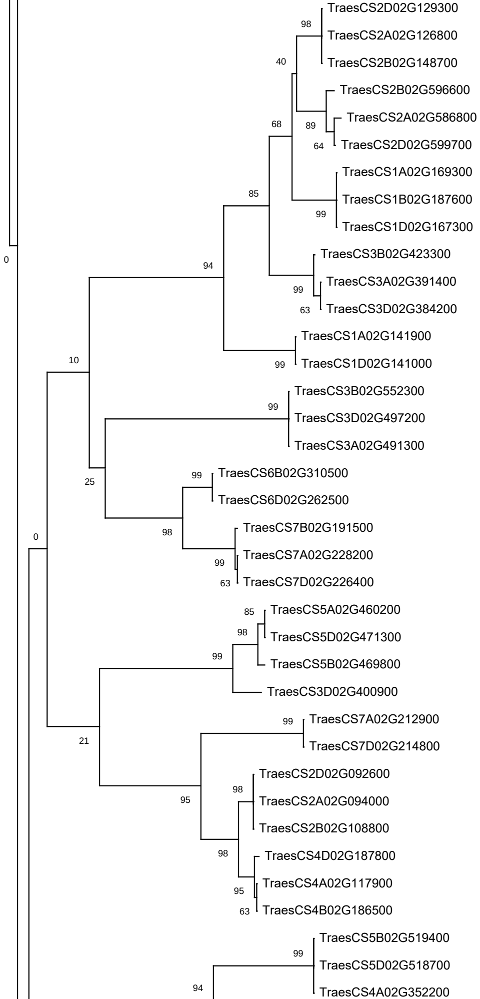
TraesCS2D02G129300
TraesCS2A02G126800
TraesCS2B02G148700
TraesCS2B02G596600
TraesCS2A02G586800
TraesCS2D02G599700
TraesCS1A02G169300
TraesCS1B02G187600
TraesCS1D02G167300
TraesCS3B02G423300
TraesCS3A02G391400
TraesCS3D02G384200
TraesCS1A02G141900
TraesCS1D02G141000
TraesCS3B02G552300
TraesCS3D02G497200
TraesCS3A02G491300
TraesCS6B02G310500
TraesCS6D02G262500
TraesCS7B02G191500
TraesCS7A02G228200
TraesCS7D02G226400
TraesCS5A02G460200
TraesCS5D02G471300
TraesCS5B02G469800
TraesCS3D02G400900
TraesCS7A02G212900
TraesCS7D02G214800
TraesCS2D02G092600
TraesCS2A02G094000
TraesCS2B02G108800
TraesCS4D02G187800
TraesCS4A02G117900
TraesCS4B02G186500
TraesCS5B02G519400
TraesCS5D02G518700
TraesCS4A02G352200

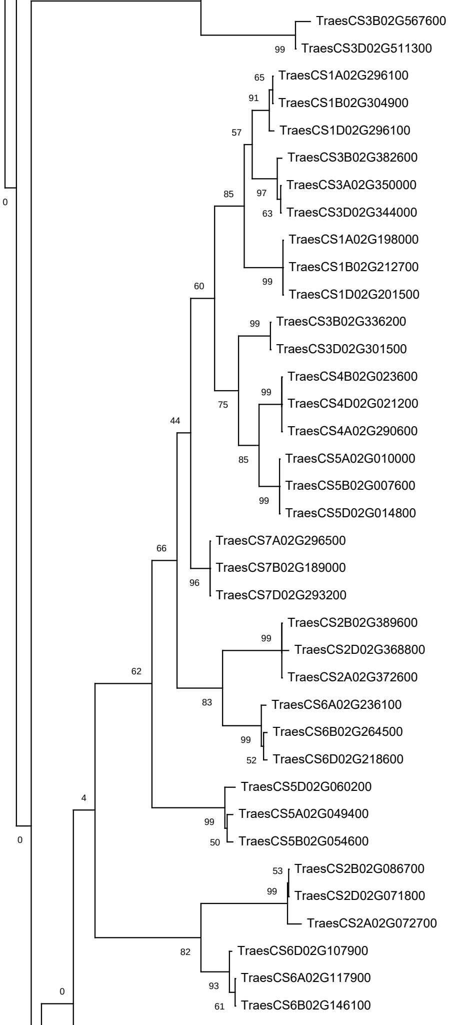
TraesCS3B02G567600
TraesCS3D02G511300
TraesCS1A02G296100
TraesCS1B02G304900
TraesCS1D02G296100
TraesCS3B02G382600
TraesCS3A02G350000
TraesCS3D02G344000
TraesCS1A02G198000
TraesCS1B02G212700
TraesCS1D02G201500
TraesCS3B02G336200
TraesCS3D02G301500
TraesCS4B02G023600
TraesCS4D02G021200
TraesCS4A02G290600
TraesCS5A02G010000
TraesCS5B02G007600
TraesCS5D02G014800
TraesCS7A02G296500
TraesCS7B02G189000
TraesCS7D02G293200
TraesCS2B02G389600
TraesCS2D02G368800
TraesCS2A02G372600
TraesCS6A02G236100
TraesCS6B02G264500
TraesCS6D02G218600
TraesCS5D02G060200
TraesCS5A02G049400
TraesCS5B02G054600
TraesCS2B02G086700
TraesCS2D02G071800
TraesCS2A02G072700
TraesCS6D02G107900
TraesCS6A02G117900
TraesCS6B02G146100

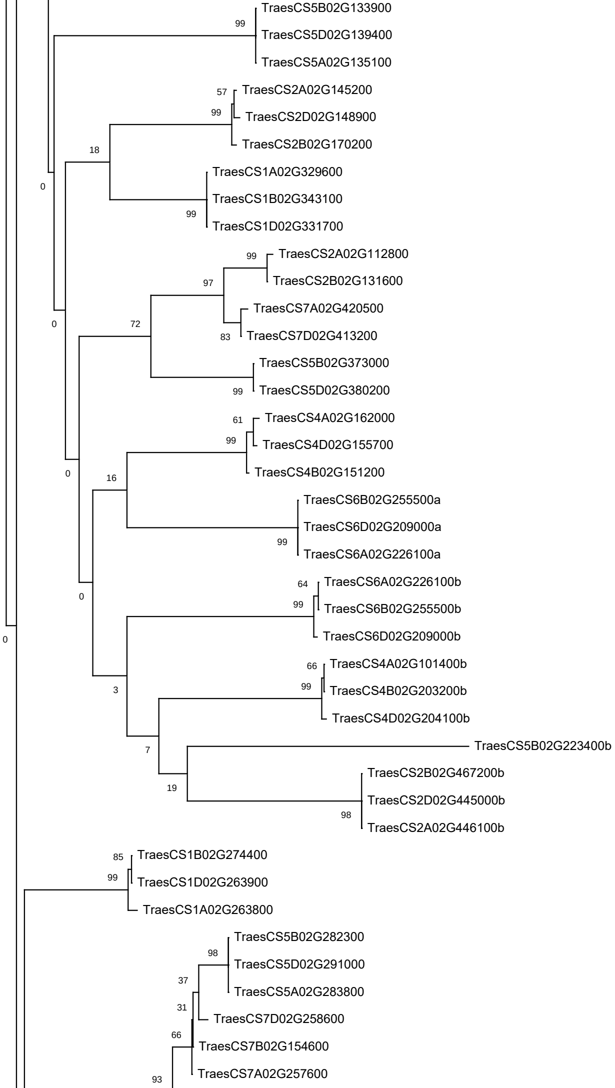
TraesCS5B02G133900
99
TraesCS5D02G139400
TraesCS5A02G135100
57
TraesCS2A02G145200
99
TraesCS2D02G148900
TraesCS2B02G170200
18
TraesCS1A02G329600
0
TraesCS1B02G343100
99
TraesCS1D02G331700
99
TraesCS2A02G112800
TraesCS2B02G131600
97
TraesCS7A02G420500
0
72
83
TraesCS7D02G413200
TraesCS5B02G373000
99
TraesCS5D02G380200
61
TraesCS4A02G162000
99
TraesCS4D02G155700
TraesCS4B02G151200
0
16
TraesCS6B02G255500a
TraesCS6D02G209000a
99
TraesCS6A02G226100a
64
TraesCS6A02G226100b
0
99
TraesCS6B02G255500b
0
TraesCS6D02G209000b
66
TraesCS4A02G101400b
99
TraesCS4B02G203200b
3
TraesCS4D02G204100b
TraesCS5B02G223400b
7
TraesCS2B02G467200b
19
TraesCS2D02G445000b
98
TraesCS2A02G446100b
85
TraesCS1B02G274400
99
TraesCS1D02G263900
TraesCS1A02G263800
TraesCS5B02G282300
98
TraesCS5D02G291000
37
TraesCS5A02G283800
31
TraesCS7D02G258600
66
TraesCS7B02G154600
TraesCS7A02G257600
93

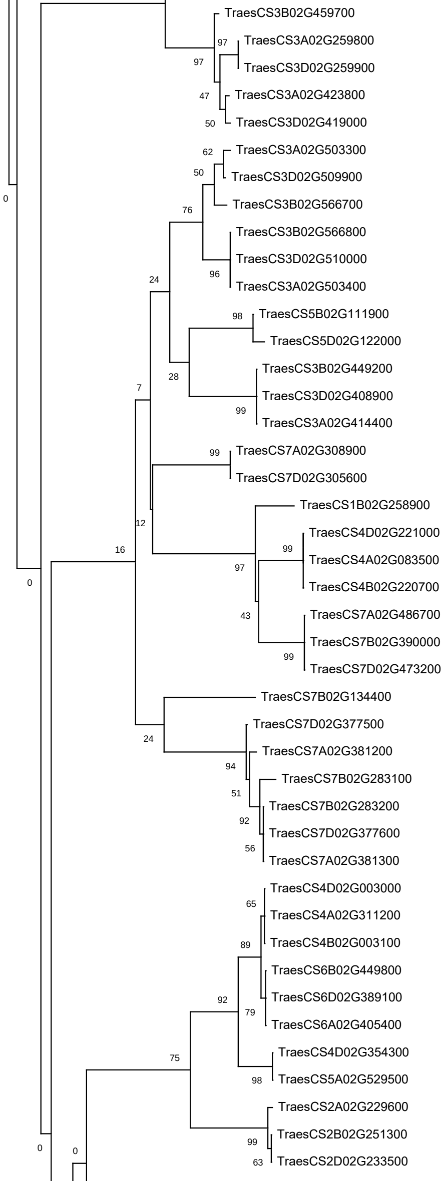
TraesCS3B02G459700
TraesCS3A02G259800
TraesCS3D02G259900
TraesCS3A02G423800
TraesCS3D02G419000
TraesCS3A02G503300
TraesCS3D02G509900
TraesCS3B02G566700
TraesCS3B02G566800
TraesCS3D02G510000
TraesCS3A02G503400
TraesCS5B02G111900
TraesCS5D02G122000
TraesCS3B02G449200
TraesCS3D02G408900
TraesCS3A02G414400
TraesCS7A02G308900
TraesCS7D02G305600
TraesCS1B02G258900
TraesCS4D02G221000
TraesCS4A02G083500
TraesCS4B02G220700
TraesCS7A02G486700
TraesCS7B02G390000
TraesCS7D02G473200
TraesCS7B02G134400
TraesCS7D02G377500
TraesCS7A02G381200
TraesCS7B02G283100
TraesCS7B02G283200
TraesCS7D02G377600
TraesCS7A02G381300
TraesCS4D02G003000
TraesCS4A02G311200
TraesCS4B02G003100
TraesCS6B02G449800
TraesCS6D02G389100
TraesCS6A02G405400
TraesCS4D02G354300
TraesCS5A02G529500
TraesCS2A02G229600
TraesCS2B02G251300
TraesCS2D02G233500

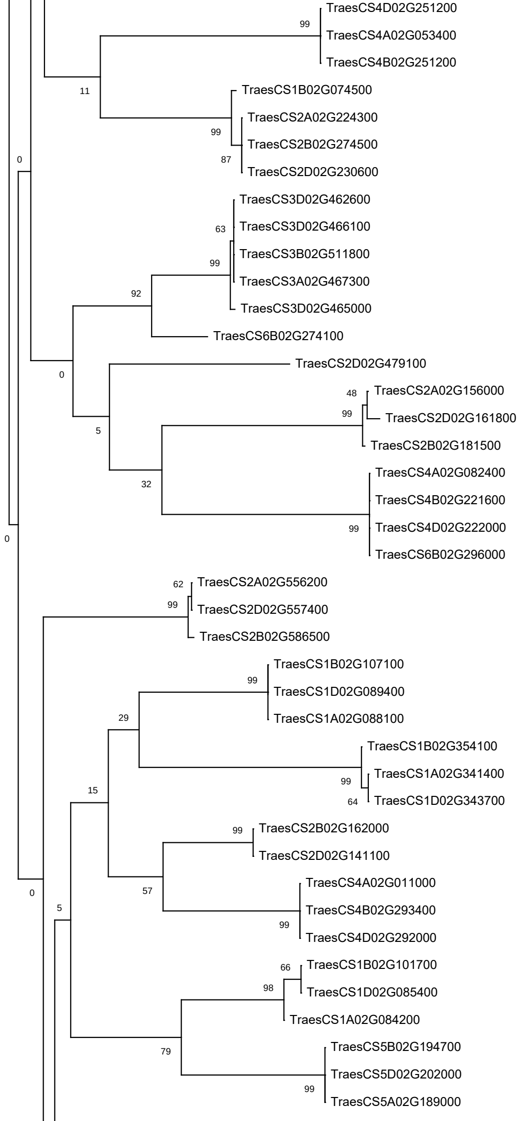
TraesCS4D02G251200
99
TraesCS4A02G053400
TraesCS4B02G251200
11
TraesCS1B02G074500
TraesCS2A02G224300
99
TraesCS2B02G274500
0
87
TraesCS2D02G230600
TraesCS3D02G462600
TraesCS3D02G466100
63
TraesCS3B02G511800
99
TraesCS3A02G467300
92
TraesCS3D02G465000
TraesCS6B02G274100
TraesCS2D02G479100
0
48
TraesCS2A02G156000
99
TraesCS2D02G161800
5
TraesCS2B02G181500
TraesCS4A02G082400
32
TraesCS4B02G221600
99
TraesCS4D02G222000
0
TraesCS6B02G296000
62
TraesCS2A02G556200
99
TraesCS2D02G557400
TraesCS2B02G586500
TraesCS1B02G107100
99
TraesCS1D02G089400
29
TraesCS1A02G088100
TraesCS1B02G354100
TraesCS1A02G341400
99
15
64
TraesCS1D02G343700
99
TraesCS2B02G162000
TraesCS2D02G141100
TraesCS4A02G011000
57
0
TraesCS4B02G293400
99
5
TraesCS4D02G292000
66
TraesCS1B02G101700
98
TraesCS1D02G085400
TraesCS1A02G084200
79
TraesCS5B02G194700
TraesCS5D02G202000
99
TraesCS5A02G189000

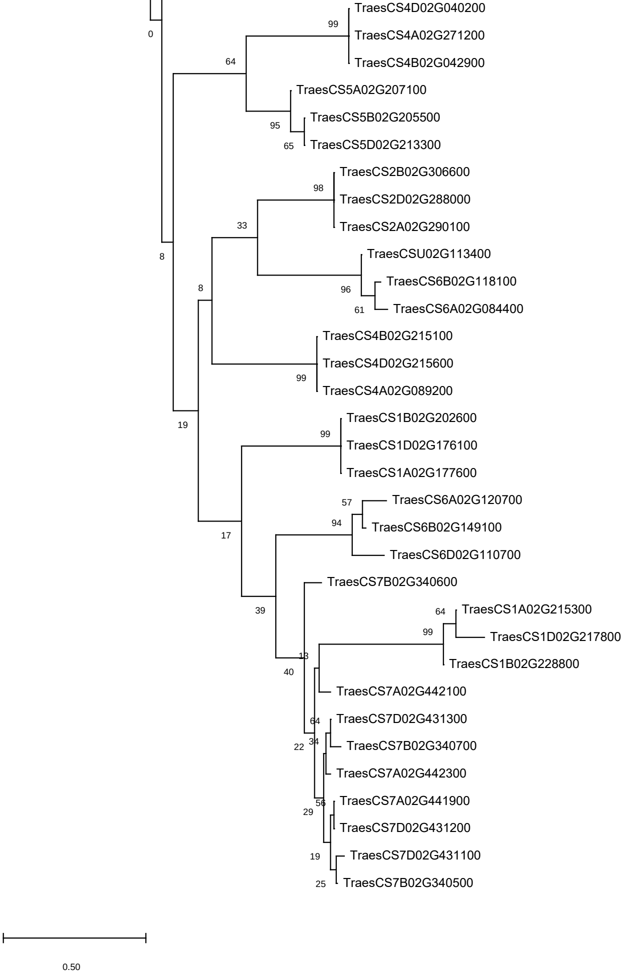

Supplementary Fig. S2. Phylogenetic tree generated by multiple sequence alignment of 1, 272 wheat RING finger domains. The bootstrap values for each node during 1000 replications of phylogenetic tree reconstruction are shown next to the branches.

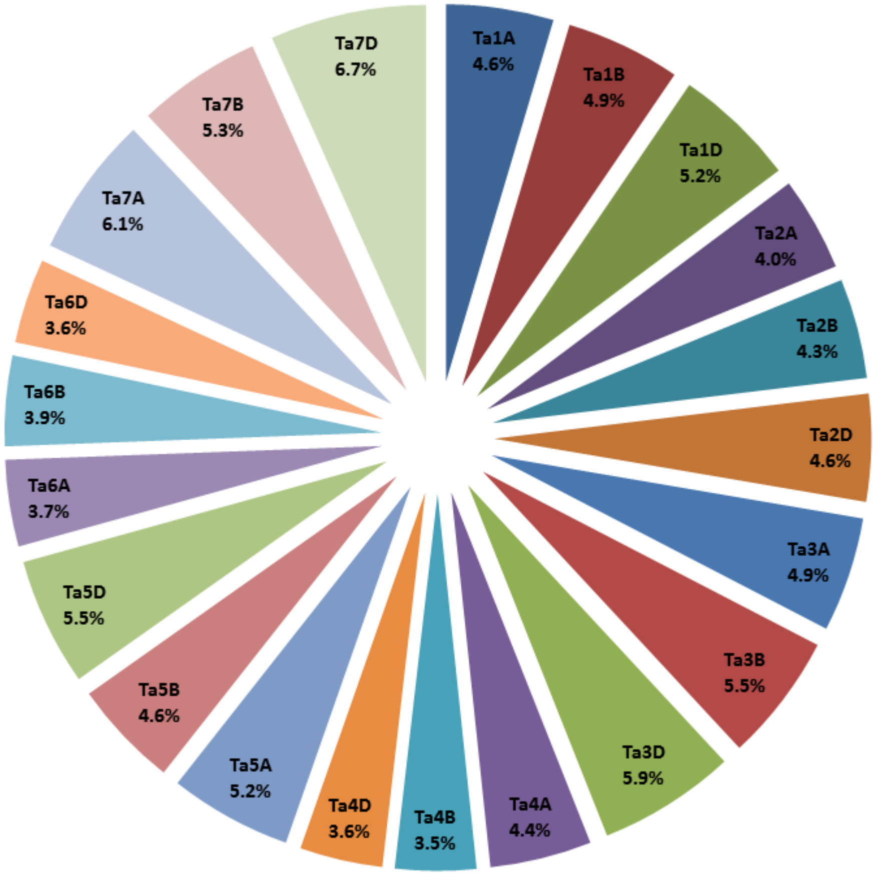

Supplementary Fig. S3. Percent distribution of RING protein genes on wheat genome on the basis of their chromosomal locations. Each chromosome is represented with a unique color and suffix after 'Ta' represents wheat chromosome name (Ta1A to Ta7D).

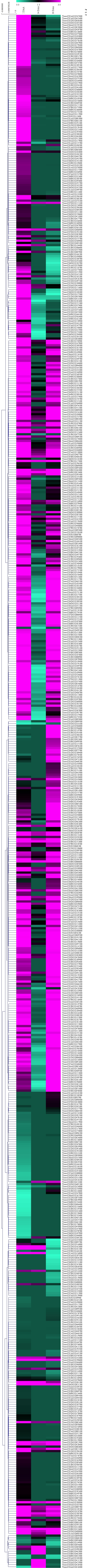

Supplementary Fig. S4. Clustered expression heat map of 698 wheat RING protein genes at three seed developmental stages- 2 DAA, 14 DAA and 30 DAA generated by MeV software version 4. 9. 0.
